# Supplementary material for: Facile access to bicyclo[2.1.1]hexanes by Lewis acid-catalyzed formal cycloaddition between silyl enol ethers and bicyclo[1.1.0]butanes
Source: Nat Commun. 2024 Jul 20;15:6128. doi: 10.1038/s41467-024-50434-6 (PMC11271461; doi:10.1038/s41467-024-50434-6)
Supplement: Supplementary file 1 — Supplementary Information [file 41467_2024_50434_MOESM1_ESM.pdf]

Facile access to bicyclo[2.1.1]hexanes by Lewis acid-catalyzed formal cycloaddition of silyl enol ethers and bicyclo[1.1.0]butanes

Sai Hu,[1,2,3†] Yuming Pan,[2,3†] Dongshun Ni,[2,3*] and Li Deng[2,3*]
[1]Department of Chemistry, Zhejiang University, Hangzhou 310027, China
[2]Key Laboratory of Precise Synthesis of Functional Molecules of Zhejiang Province, Department of Chemistry, School of Science and Research Center for Industries of the Future, Westlake University, 600 Dunyu Road, Hangzhou, 310030, Zhejiang Province, China
[3]Institute of Natural Sciences, Westlake Institute for Advanced Study, Hangzhou 310024, China.
†These authors contributed equally to this work.
*Corresponding Author(s): nidongshun@westlake.edu.cn; dengli@westlake.edu.cn.

Supplementary information

Table of Content

1. Supplementary Methods 2
1.1 General Information 2
1.2 Preparation of Substrates....3
1.2.1 Preparation of silyl enol ethers 3
1.2.2 Preparation of bicyclo[1.1.0]butanes....11
1.3 Reaction Optimization....16
1.4 Characterization of Products 17
1.5 Applications 52
2. Supplementary Discussion....59
2.1 Diastereoselectivity issues....59
2.2 Regioselectivity issues: 63
2.3 ^{13}C Kinetic Isotope Effect Study. 64
3. X-Ray Crystallography 66
4. Supplementary NMR Spectra 70
5. Supplementary References....178

1. Supplementary Methods

1.1 General Information

Unless otherwise noted, all reagents were purchased from commercial suppliers (Adamas-beta, J&K Scientific, Alfa Aesar, Leyan, Bide, Acros Organics, Energy Chemical and TCI) and used without further purification. NMR spectra were recorded on a Bruker AVANCE NEO 600 MHz NMR spectrometer (600.23 MHz for proton frequency) with a QCI-F Cryoprobe, Bruker AVANCE NEO 500 MHz NMR spectrometer (500.3 MHz for proton frequency) with a BBO Cryoprobe, Bruker AVANCE NEO 500 MHz NMR spectrometer (500.16 MHz for proton frequency) with a BBFO SMART probe. For ^{1}H NMR spectra, chemical shifts are reported in ppm from tetramethylsilane with the residual solvent resonance as the internal standard ($CDCl_3$: 7.26 ppm,). Data are reported as follows: chemical shift, multiplicity (s = singlet, d = doublet, t = triplet, q = quartet, br = broad, m = multiplet), coupling constants (Hz), and integration. All ^{13}C NMR spectra were recorded with proton decoupling unless otherwise noted. Chemical shifts are reported in ppm from tetramethylsilane with solvent resonance as the internal standard ($CDCl_3$: 77.16 ppm). ^{19}F NMR spectra were recorded on a Bruker AVANCE NEO 600 MHz NMR spectrometer (600.23 MHz for proton frequency) with a QCI-F Cryoprobe. High-resolutions spectrometric analysis was performed on ESI-HRMS (waters,synapt-XS) and EI-HRMS (Agilent, 8890GC-7250QTOF). Infrared spectra were recorded on Perkin Elmer FT-IR spectrometer and Thermo Scientific Nicolet iS50 FTIR Spectrometer are reported in frequency of absorption, ν_{max} in cm^{-1}. Bands are characterized as broad (br), strong (s), medium (m), and weak (w). All the pure compound is directly used for measurement without any treatment. Thin-layer chromatography (TLC) was performed on Silica Gel F254 glass plates and visualized with UV and/or standard potassium permanganate, phosphomolybdic acid staining techniques. Standard column chromatography techniques using ZEOprep 60/40-63 µm silica gel were used for purification. For difficult separations medium pressure liquid chromatography (MPLC) was performed using a Biotage instrument.

1.2 Preparation of Substrates

1.2.1 Preparation of silyl enol ethers

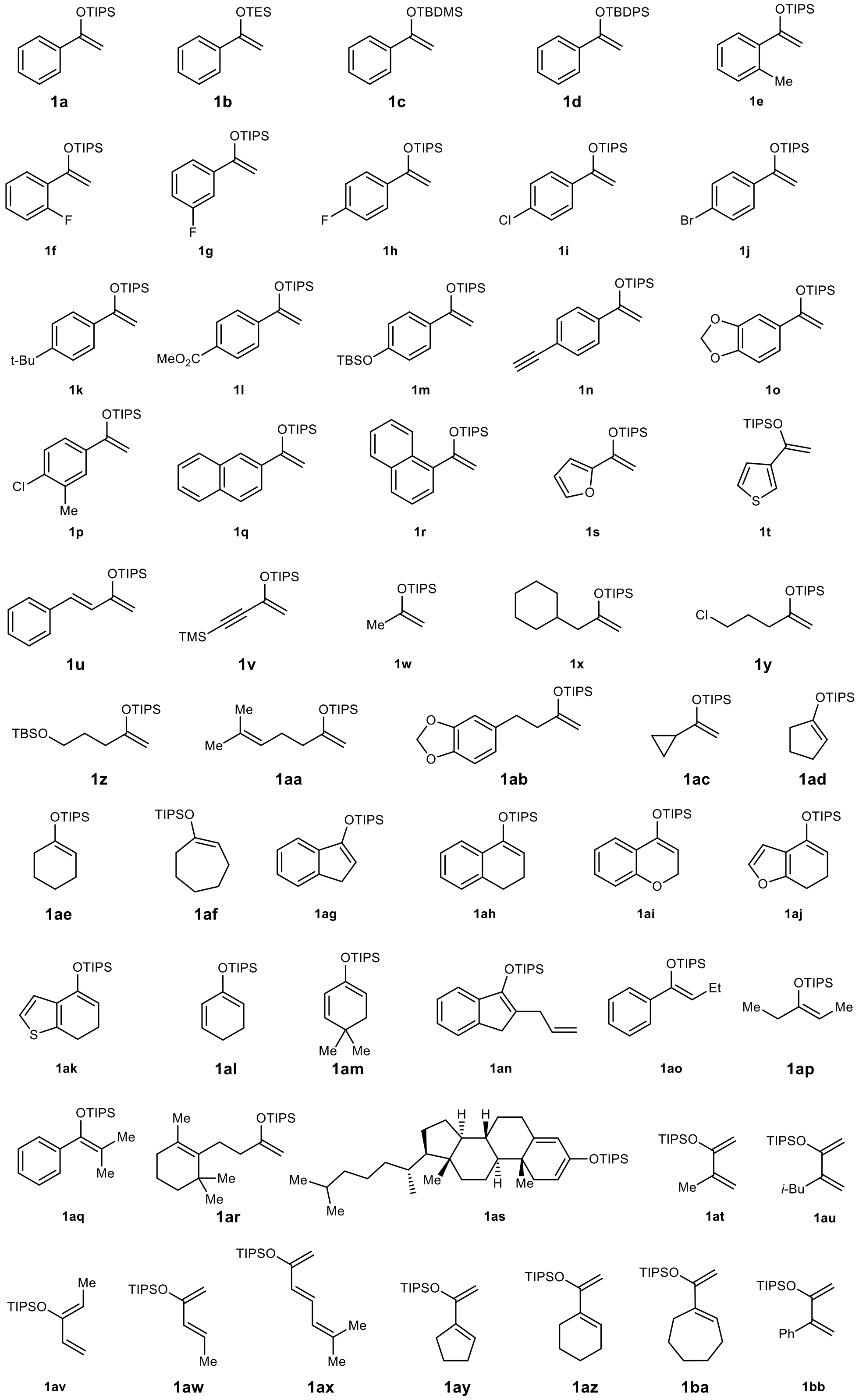

Supplementary Figure 1. Silyl enol ethers

The following compounds (**1a**[1], **1b**[1], **1c**[1], **1d**[8], **1e**[1], **1f**[1], **1g**[1], **1h**[1], **1i**[1], **1j**[1], **1k**[1], **1l**[2], **1o**[3], **1q**[1], **1r**[2], **1s**[4], **1t**[4], **1u**[2], **1w**[9], **1ad**[10], **1ae**[2], **1af**[10], **1ag**[1], **1ah**[2], **1ai**[6], **1al**[5], **1am**[7], **1ao**[3], **1ap**[2], **1aq**[1], **1as**[7], **1at**[16], **1av**[17], **1aw**[17], **1ay**[18], **1az**[18]) are known compounds and all the spectral data are consistent with reported literature. The compounds (**1m**, **1n**, **1p**, **1v**, **1x**, **1y**, **1z**, **1aa**, **1ab**, **1ac**, **1aj**, **1ak**, **1an**, **1ar**, **1ax**, **1ba** and **1bb**) were prepared according to known procedures in the literature (see the General Procedure A and B below for details)

General Procedure A: Synthesis of Silyl Enol Ethers

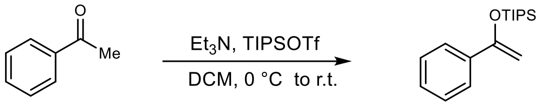

Et_3N (1.25 mL, 9 mmol, 1.8 equiv) was added to the solution of ketone (5 mmol, 1.0 equiv) in anhydrous DCM (20 mL) at 0 °C under N_2 atmosphere, followed by the addition of TIPSOTf (1.61 mL, 6 mmol, 1.2 equiv). The mixture was stirred at room temperature until the ketone was completely consumed monitored by TLC. $NaHCO_3$ (aq.) was added to quench the reaction. The solution was extracted with DCM. The combined organic layers were washed with brine, dried over Na_2SO_4, filtered, and concentrated on rotary evaporator under reduced pressure. The residue was purified by silica-gel (pre-treated with 5% Et_3N in petroleum ether) flash column chromatography to afford silyl enol ether product.

Follow the Procedure A, **1m**, **1n**, **1p**, **1v**, **1ac**, **1aj, 1ak, 1an, 1ax, 1ba** and **1bb** were prepared and characterized as follows:

tert-Butyldimethyl(4-(1-((triisopropylsilyl)oxy)vinyl)phenoxy)silane (1m)

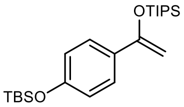

Colorless oil, 1.83 g (5 mmol scale), 90% yield, Rf = 0.85 (PE: EA = 30:1).

^{1}H NMR (500 MHz, $CDCl_3$) δ 7.54 (d, J = 8.7 Hz, 2H), 6.81 (d, J = 8.7 Hz, 2H), 4.75 (d, J = 1.7 Hz, 1H), 4.34 (d, J = 1.7 Hz, 1H), 1.37 – 1.25 (m, 3H), 1.15 (d, J = 7.4 Hz, 18H), 1.01 (s, 9H), 0.22 (s, 6H).

^{13}C NMR (125 MHz, $CDCl_3$) δ 156.1, 155.9, 131.3, 126.7, 119.7, 88.6, 25.8, 18.3, 13.0, -4.3.

ATR-IR ν (cm-1): 2945 (m), 2866 (m), 1605 (m), 1507 (m), 1254 (s), 1012 (m), 912 (s), 779 (s), 677 (m).

HRMS (ESI): Calcd for $C_{23}H_{43}O_2Si_2^+$(M+H$^+$): 407.2802, Found: 407.2809.

((1-(4-Ethynylphenyl)vinyl)oxy)triisopropylsilane (1n)

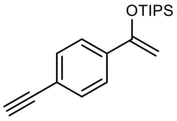

Colorless oil, 1.13 g (5 mmol scale), 75% yield, Rf = 0.9 (PE: EA = 30:1).

^{1}H NMR (600 MHz, $CDCl_3$) δ 7.66 – 7.61 (m, 2H), 7.52 – 7.46 (m, 2H), 4.91 (dq, J

= 3.6, 1.7 Hz, 1H), 4.50 (dq, J = 3.6, 1.7 Hz, 1H), 3.17 – 3.09 (m, 1H), 1.37 – 1.29 (m, 3H), 1.24 – 1.09 (m, 18H).
^{13}C NMR (150 MHz, $CDCl_3$) δ 155.5, 138.4, 132.0, 125.3, 121.8, 121.8, 91.2, 83.8, 77.9, 18.2, 12.9.
ATR-IR ν (cm-1): 3300 (w), 2944 (m), 2866 (m), 1609 (m), 1463 (m), 1311 (s), 1107 (s), 1012 (s), 845 (m), 642 (m).
HRMS (ESI): Calcd for $C_{19}H_{29}OSi^+$(M+H$^+$): 301.1988, Found: 301.1986.

((1-(4-Chloro-3-methylphenyl)vinyl)oxy)triisopropylsilane (1p)

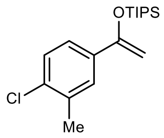

Colorless oil, 1.14 g (5 mmol scale), 70% yield, Rf = 0.9 (PE:EA = 30:1).
^{1}H NMR (600 MHz, $CDCl_3$) δ 7.52 (t, J = 2.8 Hz, 1H), 7.42 (d, J = 8.5 Hz, 1H), 7.29 (dd, J = 8.4, 2.5 Hz, 1H), 4.86 – 4.80 (m, 1H), 4.45 – 4.40 (m, 1H), 2.39 (d, J = 2.8 Hz, 3H), 1.39 – 1.26 (m, 3H), 1.14 (dd, J = 7.7, 3.8 Hz, 18H).
^{13}C NMR (150 MHz, $CDCl_3$) δ 155.4, 136.6, 135.6, 134.2, 128.8, 127.9, 124.2, 90.3, 20.4, 18.2, 12.9.
ATR-IR ν (cm-1): 2944 (m), 2866 (m), 1612 (w), 1464 (m), 1308 (s), 1113 (m), 1014 (s), 881 (m), 814 (m), 679 (s).
HRMS (ESI): Calcd for $C_{18}H_{39}ClOSi^+$(M+H$^+$): 325.1754, Found: 325.1758.

Triisopropyl((4-(trimethylsilyl)but-1-en-3-yn-2-yl)oxy)silane (1v)

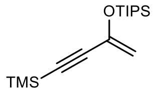

Colorless oil, 1.26 g (5 mmol scale), 85% yield, Rf = 0.9 (PE:EA = 30:1).
^{1}H NMR (600 MHz, $CDCl_3$) δ 4.74 (s, 1H), 4.69 (s, 1H), 1.30 – 1.20 (m, 3H), 1.17 – 1.07 (m, 18H), 0.18 (s, 9H).
^{13}C NMR (150 MHz, $CDCl_3$) δ 139.8, 103.2, 102.5, 92.5, 18.0, 12.7, -0.3.
ATR-IR ν (cm-1): 2945 (m), 2867 (m), 1600 (m), 1464 (m) 1265 (s), 1032 (m), 838 (s), 680 (m).
HRMS (ESI): Calcd for $C_{16}H_{33}OSi_2^+$(M+H$^+$): 297.2070, Found: 297.2074.

((1-Cyclopropylvinyl)oxy)triisopropylsilane (1ac)

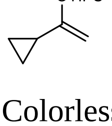

Colorless oil, 876 mg (5 mmol scale), 73% yield, Rf = 0.95 (PE).
^{1}H NMR (500 MHz, $CDCl_3$) δ 4.10 (d, *J* = 1.0 Hz, 1H), 3.96 (d, *J* = 1.0 Hz, 1H), 1.44 (tt, *J* = 8.2, 5.0 Hz, 1H), 1.26 – 1.14 (m, 3H), 1.07 (d, *J* = 7.7 Hz, 18H), 0.74 – 0.67 (m, 2H), 0.58 – 0.50 (m, 2H).
^{13}C NMR (125 MHz, $CDCl_3$) δ 159.1, 86.7, 18.2, 16.0, 12.8, 4.6.

ATR-IR ν (cm-1): 2944 (s), 2867 (s), 1649 (m), 1464 (m), 1281 (s), 1013 (s), 881 (s), 794 (s).
HRMS (ESI): Calcd for $C_{14}H_{29}OSi^{+}(M+H^{+})$: 241.1988, Found: 241.1989.

((6,7-Dihydrobenzofuran-4-yl)oxy)triisopropylsilane (1aj)

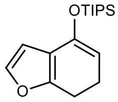

Yellow oil, 788 mg (5 mmol scale), 54% yield, Rf = 0.7 (PE).
^{1}H NMR (500 MHz, CDCl$_3$) δ 7.26 – 7.23 (m, 1H), 6.44 (t, *J* = 1.9 Hz, 1H), 4.72 (t, *J* = 2.4 Hz, 1H), 2.74 (t, *J* = 9.2 Hz, 2H), 2.50 (dd, *J* = 9.3, 4.4 Hz, 2H), 1.34 – 1.21 (m, 3H), 1.20 – 0.99 (m, 18H).
^{13}C NMR (125 MHz, CDCl$_3$) δ 153.5, 146.5, 140.6, 118.2, 106.3, 96.3, 23.2, 21.8, 18.1, 12.7.
ATR-IR ν (cm-1): 2943 (s), 2866 (s), 1634 (s), 1313 (s), 1187 (s), 1041 (s), 997 (s), 881 (s).
HRMS (ESI): Calcd for $C_{17}H_{29}O_2Si^{+}(M+H^{+})$: 293.1937, Found: 293.1946.

((6,7-Dihydrobenzo[b]thiophen-4-yl)oxy)triisopropylsilane (1ak)

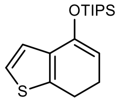

Colorless oil, 1.28 g (5 mmol scale), 83% yield, Rf = 0.7 (PE).
^{1}H NMR (500 MHz, CDCl$_3$) δ 7.09 (t, *J* = 4.1 Hz, 1H), 7.00 – 6.95 (m, 1H), 4.89 – 4.83 (m, 1H), 2.78 (td, *J* = 9.1, 8.6, 2.3 Hz, 2H), 2.45 – 2.37 (m, 2H), 1.32 – 1.19 (m, 3H), 1.18 – 1.05 (m, 18H).
^{13}C NMR (125 MHz, CDCl$_3$) δ 147.5, 137.2, 135.8, 123.4, 120.9, 98.5, 23.9, 23.5, 18.2, 12.9.
ATR-IR ν (cm-1): 2942 (s), 2865 (s), 1629 (s), 1309 (s), 1209 (s), 964 (s), 881 (s)
HRMS (ESI): Calcd for $C_{17}H_{29}OSSi^{+}(M+H^{+})$: 309.1708, Found: 309.1712.

((2-Allyl-1H-inden-3-yl)oxy)triisopropylsilane (1an)

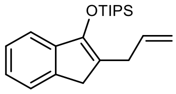

Yellow oil, 220 mg (0.87 mmol scale), 77% yield, Rf = 0.5 (PE).
^{1}H NMR (500 MHz, CDCl$_3$) δ 7.31 (dd, *J* = 7.7, 1.3 Hz, 2H), 7.27 – 7.23 (m, 1H), 7.13 (td, *J* = 7.3, 1.2 Hz, 1H), 5.88 (ddt, *J* = 16.7, 10.0, 6.7 Hz, 1H), 5.11 (dd, *J* = 17.1, 1.8 Hz, 1H), 5.04 (dt, *J* = 10.0, 1.6 Hz, 1H), 3.21 (dt, *J* = 6.7, 1.3 Hz, 2H), 3.18 (s, 2H), 1.37 – 1.24 (m, 3H), 1.14 (d, *J* = 7.4 Hz, 18H).
^{13}C NMR (125 MHz, CDCl$_3$) δ 148.4, 142.8, 141.3, 136.7, 126.1, 124.4, 123.6, 120.5, 117.8, 115.7, 36.2, 31.6, 18.2, 13.8.
ATR-IR ν (cm-1): 2944 (s), 2866 (s), 1625 (s), 1463 (m), 1360 (s), 1301 (m), 1144 (s), 993 (s), 882 (s), 856 (s), 763 (s)

HRMS (ESI): Calcd for $C_{21}H_{33}OSi^+(M+H^+)$: 329.2301, Found: 329.2299.

Triisopropyl((5-methyl-3-methylenehex-1-en-2-yl)oxy)silane (1au)

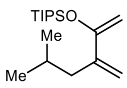

Colorless oil, 541 mg (3.0 mmol scale), 64% yield, Rf = 0.8 (PE).
^{1}H NMR (500 MHz, CDCl$_3$) δ 5.54 (d, *J* = 2.5 Hz, 1H), 4.93 – 4.89 (m, 1H), 4.43 (d, *J* = 1.4 Hz, 1H), 4.30 (d, *J* = 1.6 Hz, 1H), 2.08 (dd, *J* = 7.0, 1.0 Hz, 2H), 1.86 – 1.75 (m, 1H), 1.24 (tt, *J* = 8.7, 6.7 Hz, 3H), 1.10 (d, *J* = 7.4 Hz, 18H), 0.89 (d, *J* = 6.6 Hz, 6H).
^{13}C NMR (125 MHz, CDCl$_3$) δ 156.6, 143.6, 113.9, 91.5, 43.1, 27.2, 22.8, 18.2, 13.0.
ATR-IR ν (cm-1): 2947 (s), 2868 (s), 1588 (m), 1464 (m), 1183 (m), 1017 (s), 883 (m), 684 (m).
HRMS (ESI): Calcd for $C_{17}H_{35}OSi^+(M+H^+)$: 283.2457, Found: 283.2451.

(E)-Triisopropyl((6-methylhepta-1,3,5-trien-2-yl)oxy)silane (1ax)

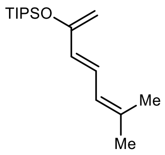

Yellow oil, 1.29 g (5 mmol scale), 92% yield, Rf = 0.9 (PE).
^{1}H NMR (500 MHz, CDCl$_3$) δ 6.84 (dd, *J* = 14.9, 11.4 Hz, 1H), 5.99 – 5.84 (m, 2H), 4.27 (d, *J* = 17.9 Hz, 2H), 1.80 (d, *J* = 12.6 Hz, 6H), 1.31 – 1.22 (m, 3H), 1.13 (d, *J* = 7.4 Hz, 18H).
^{13}C NMR (125 MHz, CDCl$_3$) δ 156.0, 137.4, 127.4, 126.4, 124.8, 94.3, 26.3, 18.5, 18.2, 12.9.
ATR-IR ν (cm-1): 2944 (m), 2866 (m), 1573 (m), 1464 (m), 1315 (s), 1022 (s), 882 (s), 676 (s).
HRMS (ESI): Calcd for $C_{17}H_{33}OSi^+$ $(M+H^+)$: 281.2301, Found: 281.2299.

((1-(Cyclohept-1-en-1-yl)vinyl)oxy)triisopropylsilane (1ba)

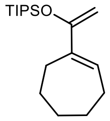

dark orange oil, 492 mg (1.9 mmol scale), 88% yield, Rf = 0.9 (PE).
^{1}H NMR (500 MHz, CDCl$_3$) δ 6.43 (t, *J* = 7.0 Hz, 1H), 4.42 (d, *J* = 1.4 Hz, 1H), 4.21 (d, *J* = 1.3 Hz, 1H), 2.36 – 2.30 (m, 2H), 2.25 – 2.18 (m, 2H), 1.80 – 1.71 (m, 2H), 1.50 (tdd, *J* = 11.5, 7.6, 5.4 Hz, 4H), 1.23 (ddd, *J* = 14.7, 8.4, 6.6 Hz, 3H), 1.10 (d, *J* = 7.3 Hz, 18H).
^{13}C NMR (125 MHz, CDCl$_3$) δ 158.1, 141.1, 129.9, 89.6, 32.6, 29.0, 28.3, 26.7, 26.5, 18.3, 13.0.
ATR-IR ν (cm-1): 2921 (s), 2866 (m), 1587 (m), 1463 (m), 1293 (s), 1202 (m), 1015

(s), 680 (s).
HRMS (ESI): Calcd for $C_{18}H_{35}OSi^+$ ($M+H^+$): 295.2457, Found: 295.2455.

Triisopropyl((3-phenylbuta-1,3-dien-2-yl)oxy)silane (1bb)

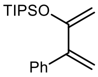

Light yellow oil. 302 mg (4.1 mmol scale), 24% yield., Rf = 0.9 (PE).
^{1}H NMR (500 MHz, CDCl$_3$) δ 7.35 – 7.24 (m, 5H), 5.75 (d, *J* = 2.2 Hz, 1H), 5.14 (d, *J* = 1.9 Hz, 1H), 4.47 (d, *J* = 1.4 Hz, 1H), 4.25 (d, *J* = 1.2 Hz, 1H), 1.32 – 1.23 (m, 3H), 1.14 (d, *J* = 7.5 Hz, 18H).
^{13}C NMR (125 MHz, CDCl$_3$) δ 156.6, 147.3, 141.0, 129.1, 127.9, 127.4, 115.2, 96.1, 18.3, 18.3, 13.0.
ATR-IR ν (cm-1): 2944 (m), 2867 (m), 1582 (m), 1463 (m), 1216 (s), 1019 (s), 882 (s), 788 (m).
HRMS (ESI): Calcd for $C_{19}H_{31}OSi^+$($M+H^+$): 303.2144, Found: 303.2143.

General Procedure B: Synthesis of Silyl Enol Ethers

The Silyl enol ethers **1x**, **1y**, **1z**, **1aa**, **1ab** and **1ar** were prepared according to a literature procedure from the corresponding ketone.[11]

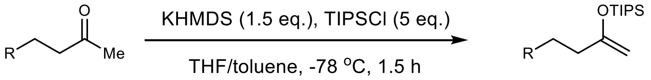

A solution of KHMDS in THF (1 M, 7.5 mmol, 7.5 mL, 1.5 equiv) was added to a solution of TIPSCl (5.3 mL, 25 mmol, 5.0 equiv) in a mixture solvent of THF (75 mL) and toluene (60 mL) at -78 °C. To the resulting mixture was added dropwise a solution of corresponding ketone (5 mmol) in toluene (15 mL) at -78°C over 30 min. After stirring at that temperature for 1.5 h, triethylamine (45 mL) was added followed by saturated $NaHCO_3$ aqueous solution (100 mL). The mixture was stirred at room temperature for 20 min and extracted with hexanes (3 × 200 mL). The organic layer was washed with water, dried over Na_2SO_4, filtered, and concentrated. The residue was purified by flash chromatography on silica gel (column was pretreated with 5% trietylamine in hexanes) using hexanes as eluent to give the TIPS silyl enol ether.

((3-Cyclohexylprop-1-en-2-yl)oxy)triisopropylsilane (1x)

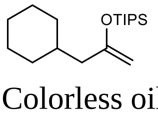

Colorless oil, 1.33 g (5 mmol scale), 90% yield, Rf = 0.9 (PE).
^{1}H NMR (500 MHz, CDCl$_3$) δ 4.02 (s, 1H), 3.94 (s, 1H), 1.92 (d, *J* = 7.0 Hz, 2H), 1.79 – 1.55 (m, 6H), 1.19 (ddd, *J* = 13.8, 6.2, 4.0 Hz, 6H), 1.08 (d, *J* = 7.2 Hz, 18H), 0.88 (ddd, *J* = 12.0, 3.3 Hz, 2H).
^{13}C NMR (125 MHz, CDCl$_3$) δ 158.5, 89.8, 44.9, 35.3, 33.3, 26.8, 26.5, 18.2, 12.8.
ATR-IR ν (cm-1): 2925 (s), 2867 (m), 1654 (w), 1614 (w), 1464 (m), 1294 (m), 1020 (m), 883 (m).
HRMS (ESI): Calcd for $C_{18}H_{37}OSi^+$($M+H^+$): 297.2614, Found: 297.2615.

((5-Chloropent-1-en-2-yl)oxy)triisopropylsilane (1y)

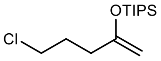

Light yellow oil, 1.01 g (5 mmol scale), 73% yield, Rf = 0.9 (PE: EA = 30:1).
Note: there are small amounts of regioisomers in the spectra.
^{1}H NMR (500 MHz, CDCl$_3$) δ 4.06 (d, J = 1.0 Hz, 1H), 4.04 (d, J = 1.0 Hz, 1H), 3.57 (t, J = 6.6 Hz, 2H), 2.22 (t, J = 7.2 Hz, 2H), 1.98 (dq, J = 8.3, 6.6 Hz, 2H), 1.25 – 1.16 (m, 3H), 1.12 – 1.06 (m, 18H).
^{13}C NMR (125 MHz, CDCl$_3$) δ 158.0, 90.0, 44.6, 34.0, 30.1, 18.2, 12.7.
ATR-IR ν (cm-1): 2944 (m), 2867 (m), 1624 (w), 1464 (m), 1272 (m), 1013 (s), 882 (s), 676 (s).
HRMS (ESI): Calcd for $C_{14}H_{30}ClOSi^+$(M+H$^+$): 277.1754, Found: 277.1748.

10,10-Diisopropyl-2,2,3,3,11-pentamethyl-8-methylene-4,9-dioxa-3,10-disiladodecane (1z)

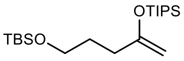

Light yellow oil, 1.64 g (5 mmol scale), 88% yield, Rf = 0.9 (PE).
Note: there are small amounts of regioisomers in the spectra.
^{1}H NMR (500 MHz, CDCl$_3$) δ 4.01 (d, *J* = 10.9 Hz, 2H), 3.63 (t, *J* = 6.5 Hz, 2H), 2.11 (dd, *J* = 8.7, 6.5 Hz, 2H), 1.79 – 1.68 (m, 2H), 1.24 – 1.16 (m, 3H), 1.09 (t, *J* = 7.9 Hz, 18H), 0.89 (s, 9H), 0.05 (s, 6H).
^{13}C NMR (125 MHz, CDCl$_3$) δ 157.3, 86.9, 60.6, 31.0, 28.2, 23.9, 16.0, 15.6, 10.5, -7.4.
ATR-IR ν (cm-1): 2945 (m), 2866 (m), 1656 (w), 1618 (w), 1464 (m), 1255 (m), 1103 (s), 1016 (m), 882 (m), 835 (s), 774 (m), 679 (m).
HRMS (ESI): Calcd for $C_{20}H_{45}O_2Si_2^+$(M+H$^+$): 373.2958, Found: 373.2950.

Triisopropyl((6-methylhepta-1,5-dien-2-yl)oxy)silane (1aa)

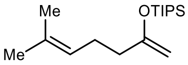

Colorless oil, 1.34 g (7 mmol scale), 68% yield, Rf = 0.6 (PE).
^{1}H NMR (500 MHz, CDCl$_3$) δ 5.04 (tt, *J* = 6.9, 1.5 Hz, 1H), 3.92 (s, 1H), 3.90 (s, 1H), 2.09 (dd, *J* = 7.5 Hz, 2H), 1.97 (dd, *J* = 9.1, 6.3 Hz, 2H), 1.59 (s, 3H), 1.51 (s, 3H), 1.15 – 1.04 (m, 3H), 0.99 (d, *J* = 7.4 Hz, 18H).
^{13}C NMR (125 MHz, CDCl$_3$) δ 159.7, 131.8, 124.2, 89.0, 37.0, 26.0, 25.8, 18.2, 17.8, 12.8.
ATR-IR ν (cm-1): 2944 (s), 2867 (s), 1655 (m), 1617 (m), 1464 (m), 1272 (s), 1048 (s), 1013 (s), 882 (s).
HRMS (ESI): Calcd for $C_{17}H_{35}OSi^+$(M+H$^+$): 283.2457, Found: 283.2467.

((4-(Benzo[d][1,3]dioxol-5-yl)but-1-en-2-yl)oxy)triisopropylsilane (1ab)

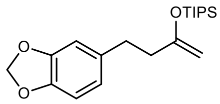

Colorless oil, 1.57 g (5 mmol scale), 90% yield, Rf = 0.5 (PE: EA = 30:1).

Note: there are small amounts of regioisomers in the spectra.

^{1}H NMR (500 MHz, $CDCl_3$) δ 6.75 – 6.70 (m, 2H), 6.66 (dd, J = 7.8, 1.7 Hz, 1H), 5.92 (s, 2H), 4.06 (d, J = 0.9 Hz, 1H), 4.02 (s, 1H), 2.81 – 2.74 (m, 2H), 2.37 – 2.28 (m, 2H), 1.28 – 1.19 (m, 3H), 1.16 – 1.11 (m, 18H).

^{13}C NMR (125 MHz, $CDCl_3$) δ 158.9, 147.6, 145.7, 136.0, 121.2, 109.0, 108.2, 100.8, 89.5, 39.2, 33.5, 18.2, 12.8.

ATR-IR ν (cm-1): 2944 (m), 2866 (m), 1624 (w), 1490 (m), 1243 (s), 1040 (s), 882 (m), 806 (m), 677 (m).

HRMS (ESI): Calcd for $C_{20}H_{33}O_3Si^+$(M+H$^+$): 349.2199, Found: 349.2196.

Triisopropyl((4-(2,6,6-trimethylcyclohex-1-en-1-yl)but-1-en-2-yl)oxy)silane (1ar)

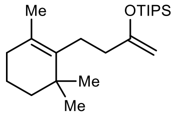

Colorless oil, 1.31 g (5 mmol scale), 75% yield, Rf = 0.6 (PE).

^{1}H NMR (500 MHz, $CDCl_3$) δ 4.01 (d, *J* = 5.1 Hz, 2H), 2.26 – 2.19 (m, 2H), 2.14 – 2.07 (m, 2H), 1.91 (t, *J* = 6.3 Hz, 2H), 1.62 (s, 3H), 1.60 – 1.53 (m, 2H), 1.45 – 1.39 (m, 2H), 1.26 – 1.16 (m, 3H), 1.10 (d, *J* = 7.2 Hz, 18H), 1.00 (s, 6H).

^{13}C NMR (125 MHz, $CDCl_3$) δ 160.6, 137.0, 127.5, 88.2, 40.0, 37.7, 35.2, 32.9, 28.7, 27.0, 19.9, 19.7, 18.2, 12.9.

ATR-IR ν (cm-1): 2943 (s), 2867 (s), 1655 (m), 1616 (m), 1464 (m), 1288 (m), 1014 (m), 883 (m).

HRMS (ESI): Calcd for $C_{22}H_{43}OSi^+$(M+H$^+$): 351.3083, Found: 351.3081.

1.2.2 Preparation of bicyclo[1.1.0]butanes

Bicyclo[1.1.0]butanes (**2a**, **2b**, **2c**, **2d**, **2e**, **2f**, **2g** and **2h**) are known compounds and were prepared based on the reported procedure (see General Procedure C and D below)[12-15]. All the spectral data matched with that reported in the literature.

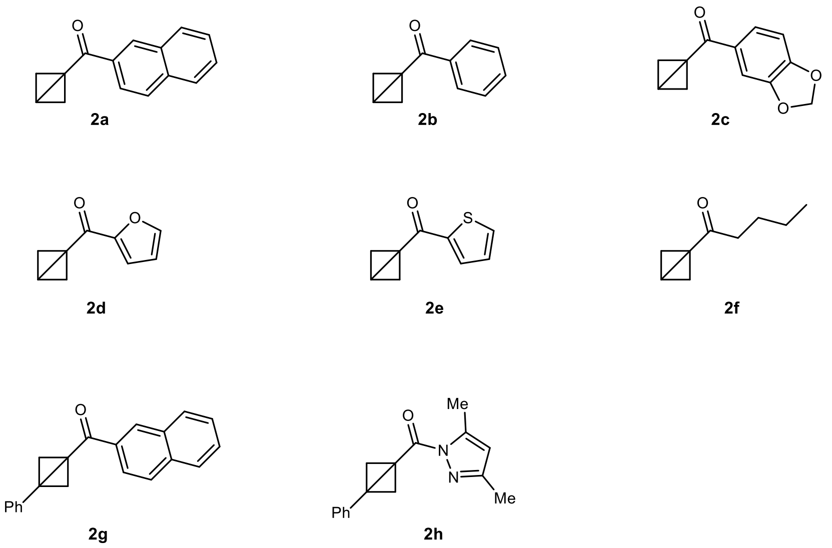

Supplementary Figure 2. Bicyclo[1.1.0]butanes

The detail for the preparation of the following monosubstituted bicyclo[1.1.0]butanes (**2a-2f**) was illustrated on the General Procedure C.

General Procedure C: Synthesis of monosubstituted bicyclo[1.1.0]butanes

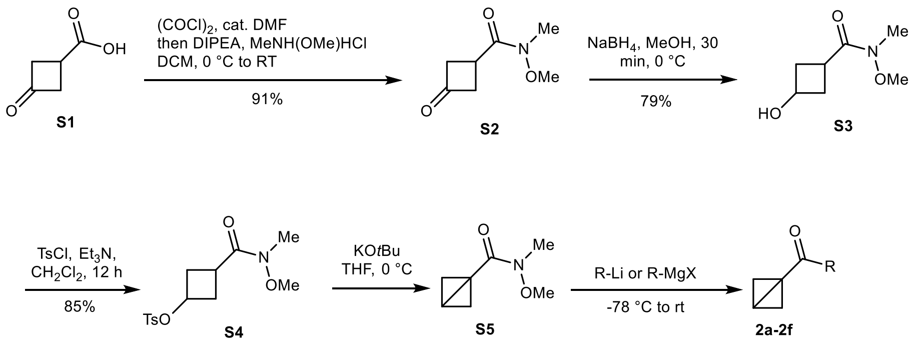

An oven-dried 1 L flask with a stir bar was charged with 3-oxocyclobutane carboxylic acid **S1** (11.4 g, 100 mmol). The flask was sealed with a septum, evacuated and backfilled with N_2 (× 3). Under N_2 atmosphere, anhydrous CH_2Cl_2 (400 mL) was added. The solution was cooled to 0 °C under ice/water bath. Then oxalyl chloride (9.4 mL, 110 mmol) was added dropwise followed by the addition of 20 drops of DMF. The resulting mixture was allowed to stir at room temperature for overnight. After being cooled to 0 °C again, MeNH(OMe)·HCl (10.7 g, 110 mmol) and DIPEA (61.0 mL, 350 mL) were added. The mixture was stirred at room temperature for 12 h. Aqueous saturated $NaHCO_3$ was added to quench the reaction. The solution was extracted with CH_2Cl_2. The combined organic phases were washed with brine, dried

over Na_2SO_4, filtered and concentrated on rotary evaporator. The crude residue was purified by silica-gel flash column chromatography (PE: EA = 1:1 to 1:2) to give Weinreb amide **S2** (14.3 g, 91% yield) as light yellow oil.

To a solution of Weinreb amide **S2** (5.10 g, 32.4 mmol) in methanol (108 mL) was added $NaBH_4$ (1.22 g, 32.4 mmol) in portions on ice/water bath. The mixture was stirred at same temperature for 30 min. Then aqueous saturated NH_4Cl solution was added to quench the reaction. The most methanol was removed on rotavapor under reduced pressure. The aqueous solution was extracted with ethyl acetate, washed with brine, dried over Na_2SO_4, filtered and concentrated on rotavapor. The crude alcohol **S3** was used directly for the next reaction without further purification.

The crude alcohol **S3** was dissolved in anhydrous DCM and the solution was cooled to 0 ℃ at ice/water bath. Then Et_3N was added followed by the addition of TsCl. The resulting mixture was allowed to stir at room temperature for 24 h before adding water to quench the reaction. The mixture was extracted with DCM, washed with brine, dried over Na_2SO_4, filtered and concentrated on rotavapor under reduced pressure. The crude residue was purified by flash column chromatography (50%~60% ethyl acetate in petroleum ether) to give the tosylate **S4** (6.9 g, 68% for two steps) as light yellow solid.

An oven-dried 100-mL Schlenk flask was charged with tosylate **S4** (3.13 g, 10.0 mmol). Anhydrous THF (67 mL) was added to dissolve the solid. The solution was cooled to 0 ℃ in an ice/water bath. A solution of KO*t*Bu (10.5 mL, 1.00 M in THF) was added. During the addition, the solution would become viscous suspension. The suspension was stirred vigorously for 15 min before the addition of aqueous saturated NH_4Cl solution. The mixture was extracted with ethyl acetate, washed with brine, dried over Na_2SO_4, filtered and concentrated on rotavapor under reduced pressure. The crude residue was purified by silica-gel flash column chromatography to give the BCB Weinreb **S5** (1.0 g, 71% yield) as a colorless oil. (Note: the BCB Weinreb **S5** would be taken away under vacuum.)

Under N_2 atmosphere, the BCB Weinreb **S5** (5.0 mmol, 1 equiv.) was dissolved in anhydrous THF (15 mL, 0.33 mmol/mL). After being cooled to -78 ℃, a solution of organolithium or Grignard reagent (6.0 mmol, 1.2 equiv.) was added dropwise. The resulting mixture was allowed to stir at same temperature for 30 min and then warmed to room temperature for another 1.5 h. Aqueous NH_4Cl solution was added to quench the reaction. The mixture was extracted with ethyl acetate, washed with brine, dried over Na_2SO_4, filtered and concentrated on rotavapor under reduced pressure. The crude residue was purified by flash column chromatography to give the corresponding monosubstituted BCB.

General Procedure D: Synthesis of disubstituted bicyclo[1.1.0]butanes

Preparation of naphthalen-2-yl(3-phenylbicyclo[1.1.0]butan-1-yl)methanone (**2g**)

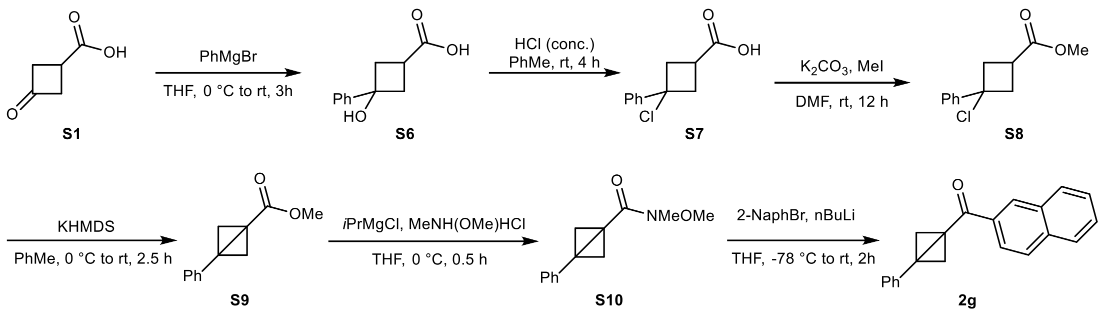

An oven-dried 100 mL round bottom flask equipped with a stir bar was charged with 3-oxocyclobutanecarboxylic acid **S1** (2.3 g, 20 mmol, 1.0 equiv.). After backfilled with N_2 (× 3) and capped with a septum, THF (50 mL) were added. The reaction was cooled to 0 °C in an ice/water bath and PhMgBr (16 mL, 3.0 M in ether, 48 mmol, 2.4 equiv.) was added to the solution. The ice/water bath was then removed and the reaction was stirred for 2 h at room temperature before quenched with saturated NH_4Cl solution (50 mL). The aqueous layer was extracted with EtOAc (3 × 50 mL). The combined organic layers were washed with brine (100 mL), dried over anhydrous Na_2SO_4, filtered and concentrated on rotavapor under reduced pressure. The crude acid **S6** was directly used in next reaction without further purification. A 100 mL round bottom flask equipped with a magnetic stir bar was charged with above crude acid **S6** (assuming 20.0 mmol, 1.0 equiv.), concentrated HCl solution (25 mL) and PhMe (25 mL). The flask was capped with a septum. The reaction was vigorously stirred for 4 h at room temperature. The aqueous layer was extracted with Et_2O (3 × 50 mL). The combined organic layers were washed with brine (100 mL), dried over anhydrous Na_2SO_4, filtered and concentrated by rotary evaporation. The crude acid **S7** was directly used in next reaction without further purification.

A 250 mL round bottom flask equipped with a magnetic stir bar was charged with above crude acid **S7** (assuming 20.0 mmol, 1.0 equiv.) and DMF (100 mL). K_2CO_3 (5.52 g, 40.0 mmol, 2.00 equiv.) and MeI (1.87 mL, 30.0 mmol, 1.50 eq.) was added sequentially to the solution. The flask was capped with a septum. The reaction was stirred at room temperature for overnight. The solution was then diluted with H_2O (50 mL) and extracted with Et_2O (3 × 50 mL). The combined organic layers were washed with brine (100 mL), dried over anhydrous Na_2SO_4, filtered and concentrated on rotavapor under reduced pressure. The crude residue was purified by flash column chromatography (10%~12% ethyl acetate in petroleum ether) to afford ester **S8** (3.0 g, 67% yield over 3 steps) as a colorless oil. All spectral data matched that reported in the literature.

An oven-dried 100 mL round bottom flask equipped with a stir bar was charged with **S8** (1.12 g, 5.0 mmol, 1.0 equiv.). After backfilled with N_2 (× 3) and capped with a septum, THF (20 mL) were added. Then the solution was cooled to 0 °C, and NaHMDS (6.0 mL, 6.0 mmol, 1.2 equiv.) was added. The resulting mixture was stirred for 30 min before the addition of saturated NH_4Cl solution (20 mL). The aqueous layer was extracted with Et_2O (3 × 50 mL). The combined organic layers were washed with brine (100 mL), dried over anhydrous Na_2SO_4, filtered and concentrated by rotary evaporation. The crude residue was purified by flash column

chromatography (15% ethyl acetate in petroleum ether) to afford ester **S9** (683 mg, 72% yield) as a white solid.

An oven-dried 25 mL round bottom flask equipped with a stir bar was charged with ester **S9** (328 mg, 1.74 mmol, 1.0 equiv.). After backfilled with N_2 (× 3) and capped with a septum, THF (10 mL) were added. The reaction was cooled to 0 °C. MeNH(OMe)·HCl (204 mg, 2.1 mmol, 1.2 eq.) and *i*PrMgCl (3.2 mL, 1.3 M in THF, 4.2 mmol, 2.40 equiv.) were sequentially added to the solution. After stirred at the same temperature for 0.5 h, the reaction was quenched by saturated NH_4Cl solution (20 mL). The aqueous layer was extracted with EtOAc (3 × 10 mL). The combined organic layers were washed with brine (25 mL), dried over anhydrous Na_2SO_4, filtered and concentrated by rotary evaporation. The crude residue was purified by flash column chromatography (30% ethyl acetate in petroleum ether) to afford amide **S10** (125 mg, 33% yield) as a light yellow solid.

An oven-dried 10 mL vial equipped with a stir bar was charged with 2-NaphBr (180 mg, 0.87 mmol, 1.50 equiv.). After backfilled with N_2 (× 3) and capped with a septum, THF (4.8 mL) were added. The solution was cooled to -78 °C in the dry ice/acetone bath. and *n*BuLi (0.50 mL, 1.6 M in Hex, 0.81 mmol, 1.40 equiv.) was added. After stirred at the same temperature for 0.5 h, a solution of amide **S10** (125 mg, 0.58 mmol, 1.0 equiv.) in THF (1 mL) was added. After 0.5 h, the dry ice/acetone bath was removed, and the reaction was stirred at room temperature for 1.5 h before quenched with saturated NH_4Cl solution (5 mL). The aqueous layer was extracted with EtOAc (3 × 10 mL), washed with brine (10 mL), dried over anhydrous Na_2SO_4, filtered and concentrated on rotavapor under reduced pressure. The crude residue was purified by flash column chromatography (10-14% ethyl acetate in petroleum ether) to afford ketone **2g** (140 mg, 56% yield) as a white solid. All spectral data matched that reported in the literature (ref. 14).

Preparation of (3,5-dimethyl-1H-pyrazol-1-yl)(3-phenylbicyclo[1.1.0]butan-1-yl) methanone (**2h**)

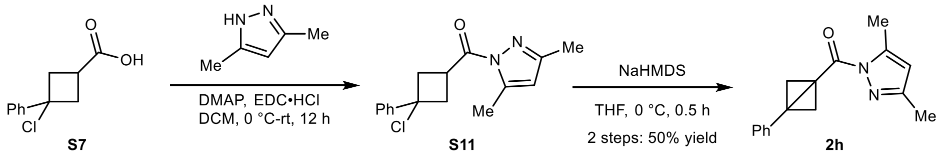

To a 250 mL round-bottomed flask the carboxylic acid **S7** (16.6 mmol, 3.5 g, 1.00 equiv.) was added followed by 3,5-dimethyl-1H-pyrazole (18.3 mmol, 1.76 g, 1.10 equiv.), DMAP (16.6 mmol, 2.03 g, 1.00 equiv.), and DCM (50 mL), and the solution was cooled to 0 °C. EDC hydrochloride (19.9 mmol, 3.82 g, 1.20 equiv.) was added and the solution was stirred at room temperature overnight. The product mixture was transferred to a separatory funnel with DCM (10 mL), and then washed with 1N HCl solution (2 × 20 mL) and brine (2 × 40 mL). The organic phase was dried over anhydrous $NaSO_4$, filtered and concentrated to dryness by rotary evaporation to afford a crude solid **S11** which was used directly in next step.

The above solid **S11** (assuming 16.6 mmol) was dissolved with THF (50 mL) and the solution was cooled to 0 °C. NaHMDS (1.0 M, 16.6 mL) was added dropwise to

this solution at 0 °C. After being stirred at 0 °C for 0.5 h, quenched with saturated NH_4Cl (20 mL) and water (20 mL), and extracted with EtOAc (3 × 50 mL). The combined organic layers were dried over anhydrous Na_2SO_4, filtered and concentrated by rotary evaporation. The crude mixture was purified by flash column chromatography (0-10% ethyl acetate in hexane) to afford **2h** (2.1 g, 50% yield over 2 steps) as a white solid. All spectral data matched that reported in the literature (ref. 15).

Unsuccessful substrates:

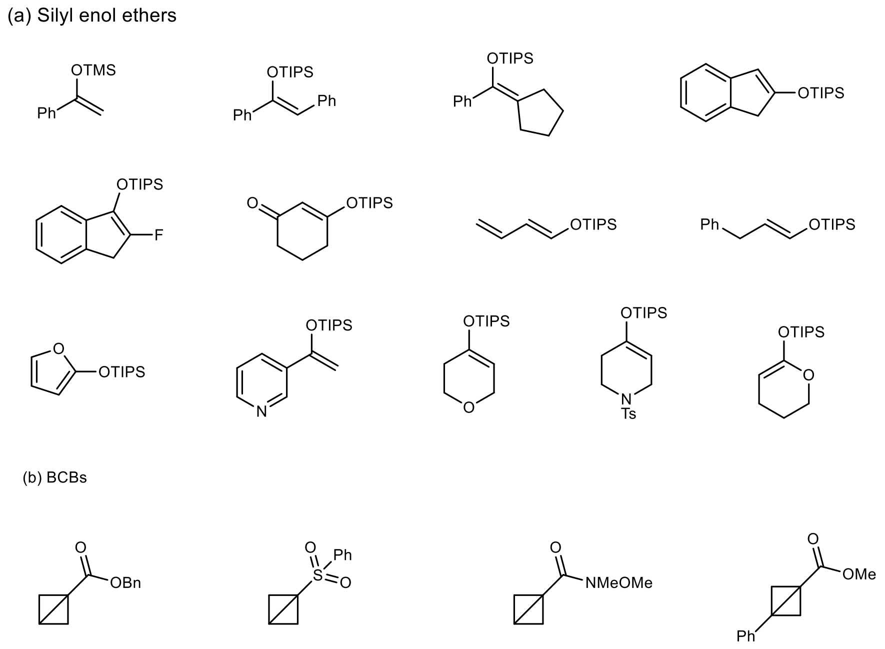

Supplementary Figure 3. Unsuccessful substrates. **a** Silyl enol ethers. **b** BCBs

1.3 Reaction Optimization

General Procedure E for reaction evaluation:

A 1-dram screw-cap vial with a stir bar was charged with silyl enol ether **1a** (0.1 mmol), BCB **2a** (0.13 mmol) and catalyst (0.01 mmol). Then the vial was sealed with a septum, evacuated, and backfilled with N_2 (× 3). Under N_2 atmosphere, dry solvent (1 mL) was added rapidly. The septum was quickly replaced with a screw cap. The reaction was stirred for 2 – 8 h. The reaction mixture was filtered through a pad of silica-gel and washed with DCM. The filtrate was concentrated on rotary evaporator and the residue was analyzed by ^{1}H NMR with 1,1,2,2-tetrachloroethane or 1,3,5-trimethoxybenzene as the internal standard.

Supplementary Table 1 Optimization of reaction conditions[a]

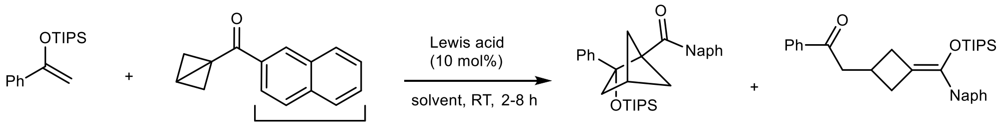

entry	Lewis acid	solvent	time	results[b]
**1**	$Cu(OTf)_2$	DCM	6 h	**3a** (5%), **4** (45%)
**2**	$Zn(OTf)_2$	DCM	6 h	**3a** (14%), **4** (57%)
**3**	$Ni(OTf)_2$	DCM	6 h	**3a** (3%), **4** (45%)
**4**	AgOTf	DCM	6 h	**3a** (6%), **4** (46%)
**5**	$Sc(OTf)_3$	DCM	2 h	**3a** (56%)
**6**	$B(C_6F_5)_3$	DCM	4 h	**4** (43%)
**7**	$Ce(OTf)_3$	DCM	8 h	**3a** (42%)
**8**	$Eu(OTf)_3$	DCM	3 h	**3a** (84%)
**9**	$Gd(OTf)_3$	DCM	3 h	**3a** (74%)
**10**	$Tm(OTf)_3$	DCM	2 h	**3a** (92%)
**11**	$Lu(OTf)_3$	DCM	2 h	**3a** (90%)
**12**	$Yb(OTf)_3$	DCM	2 h	**3a** (97%)(96%)[c]
**13**	$YbCl_3$	DCM	8 h	n.r.
**14**	$Yb(OAc)_3$	DCM	8 h	n.r.
**15**	$Yb(OTf)_3$	toluene	2 h	**3a** (98%)(99%)[c]
**16**	$Yb(OTf)_3$	THF	2 h	**3a** (42%)
**17**	$Yb(OTf)_3$	MeCN	2 h	**3a** (63%)
**18**	-	DCM	2 h	n.r.

[a]Reactions were run on a 0.10 mmol scale, with 0.10 mmol silyl enol ethers **1a**, 0.13 mmol BCB **2a,** and 0.01 mmol Lewis acid. [b]Yields were determined by ^{1}H NMR analysis of the unpurified reaction mixture with 1,1,2,2-tetrachloroethane or 1,3,5-trimethoxybenzene as an internal standard. [c]Yield of the reaction with 5 mol% of $Yb(OTf)_3$.

Investigation of byproduct **4**

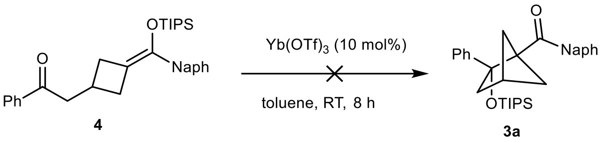

We treated the byproduct **4** with Lewis acid, however, no cycloadduct was observed.

General Procedure F for substrate scope:

A 1-dram screw-cap vial with a stir bar was charged with silyl enol ether **1** (0.2 mmol), BCB **2** (0.26 mmol) and $Yb(OTf)_3$ (0.01 mmol). Then the vial was sealed with a septum, evacuated, and backfilled with N_2 (× 3). Under N_2 atmosphere, dry DCM or toluene (2 mL) was added rapidly. The septum was quickly replaced with a screw cap. The reaction was stirred for 2 – 4 h until silyl enol ether was completely consumed. The reaction mixture was filtered through a pad of silica-gel and washed with DCM. The filtrate was concentrated on rotary evaporator and the residue was directly purified by silica-gel flash column chromatography to afford the product **3**. If silyl enol ether or BCB was liquid, a solution of silyl enol ether or/and BCB solution in DCM or toluene was added to the vial with $Yb(OTf)_3$ to make reaction mixture.

General Procedure G for substrate scope:

A 1-dram screw-cap vial with a stir bar was charged with silyl dienol ether **1** (0.2 mmol), BCB **2** (0.26 mmol). Then the vial was sealed with a septum, evacuated, and backfilled with N_2 (× 3). Under N_2 atmosphere, dry DCM or toluene (2 mL) was added rapidly. When fully dissolved, $Sc(OTf)_3$ (0.02 mmol) was added in one portion, and the septum was quickly replaced with a screw cap. The reaction was stirred for 30 minutes until silyl dienol ether was completely consumed. After quenching with triethylamine (0.2 mL), the reaction mixture was filtered through a pad of deactivated silica-gel and washed with DCM. The filtrate was concentrated on rotary evaporator and the residue was directly purified by silica-gel flash column chromatography to afford the product **5**.

1.4 Characterization of Products

Naphthalen-2-yl(2-phenyl-2((triisopropylsilyl)oxy)bicyclo[2.1.1]hexan-1-yl)methanone (3a)

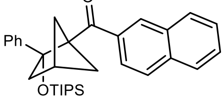

The title compound was prepared according to the General Procedure F with silyl enol ether **1a** and BCB **2a** in toluene within 2 h. Purification by flash column chromatography (0-10% ethyl acetate in hexane) provided the cycloaddition product **3a** (90 mg, 93% yield) as a colorless oil.

^{1}H NMR (500 MHz, $CDCl_3$) δ 8.21 (s, 1H), 7.92 (dd, J = 8.7, 1.7 Hz, 1H), 7.86 – 7.75 (m, 3H), 7.55 (td, J = 6.9, 1.3 Hz, 1H), 7.49 (td, J = 7.4, 1.3 Hz, 1H), 7.42 – 7.34

(m, 2H), 7.24 – 7.14 (m, 3H), 3.10 (dt, J = 11.7, 2.1 Hz, 1H), 2.86 (td, J = 6.8, 2.3 Hz, 1H), 2.57 (s, 1H), 2.34 – 2.26 (m, 1H), 2.22 (d, J = 11.8 Hz, 1H), 1.92 – 1.81 (m, 2H), 1.03 – 0.91 (m, 3H), 0.89 (d, J = 7.1 Hz, 9H), 0.83 (d, J = 7.2 Hz, 9H).
^{13}C NMR (125 MHz, $CDCl_3$) (one peak on aromatic area is missing) δ 201.7, 143.4, 135.6, 135.3, 132.4, 131.8, 129.7, 128.1, 127.8, 127.7, 127.6, 127.5, 126.3, 125.3, 86.4, 70.2, 44.7, 43.0, 42.6, 33.8, 18.4, 18.3, 13.5.
ATR-IR ν (cm-1): 2945 (s), 2866 (s), 1655 (s), 1464 (m), 1306 (m), 1055 (m), 780 (s), 702 (m).
HRMS (ESI): Calcd for $C_{32}H_{40}O_2SiNa^+$ (M+Na$^+$): 507.2695, Found: 507.2693.

2-(3-(Naphthalen-2-yl((triisopropylsilyl)oxy)methylene)cyclobutyl)-1-phenylethan-1-one (4)

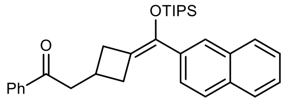

^{1}H NMR (600 MHz, $CDCl_3$) δ 7.98 – 7.92 (m, 2H), 7.82 – 7.74 (m, 4H), 7.58 – 7.51 (m, 2H), 7.48 – 7.41 (m, 4H), 3.30 – 3.16 (m, 4H), 2.98 – 2.88 (m, 1H), 2.70 – 2.57 (m, 2H), 1.14 – 1.01 (m, 21H).
^{13}C NMR (150 MHz, $CDCl_3$) (one peak on aromatic area is missing) δ 199.6, 143.3, 137.1, 135.8, 133.2, 132.5, 128.7, 128.2, 128.2, 127.7, 127.4, 126.1, 125.8, 125.1, 124.9, 118.7, 45.3, 37.2, 35.8, 27.4, 18.1, 13.4.
ATR-IR ν (cm-1): 2944 (m), 2866 (m), 1686 (s), 1448 (m), 1289 (m), 1240 (m), 1129 (m), 883 (m), 749 (m), 688 (m).
HRMS (ESI): Calcd for $C_{32}H_{40}O_2SiNa^+$ (M+Na$^+$): 507.2695, Found: 507.2692.

Naphthalen-2-yl(2-phenyl-2-((triethylsilyl)oxy)bicyclo[2.1.1]hexan-1-yl)methanone (3b)

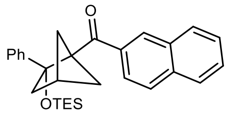

The title compound was prepared according to the General Procedure F with silyl enol ether **1b** and BCB **2a** in DCM for 2 h. Purification by flash column chromatography (0-10% ethyl acetate in hexane) provided the cycloaddition product **3b** (40 mg, 45% yield) as a colorless oil.
^{1}H NMR (500 MHz, $CDCl_3$) δ 8.64 – 8.60 (m, 1H), 8.06 (dd, *J* = 8.6, 1.7 Hz, 1H), 7.91 (dd, *J* = 8.1, 1.2 Hz, 1H), 7.86 (dd, *J* = 8.3, 1.2 Hz, 1H), 7.83 (d, *J* = 8.6 Hz, 1H), 7.58 (ddd, *J* = 8.2, 6.8, 1.4 Hz, 1H), 7.53 (ddd, *J* = 8.1, 6.8, 1.4 Hz, 1H), 7.31 – 7.27 (m, 2H), 7.23 – 7.16 (m, 3H), 3.06 (ddd, *J* = 11.9, 2.8, 1.7 Hz, 1H), 2.81 – 2.72 (m, 1H), 2.59 – 2.54 (m, 1H), 2.28 (dt, *J* = 6.2, 2.9 Hz, 1H), 2.19 – 2.12 (m, 1H), 1.86 – 1.77 (m, 2H), 0.75 (t, *J* = 7.9 Hz, 9H), 0.51 – 0.31 (m, 6H).
^{13}C NMR (125 MHz, $CDCl_3$) δ 201.7, 143.3, 135.4, 135.2, 132.6, 132.5, 129.8, 128.2, 127.8, 127.7, 127.7, 127.6, 127.5, 126.4, 125.5, 85.8, 70.3, 45.2, 43.1, 41.9, 33.9, 7.0, 6.3.
ATR-IR ν (cm-1): 2955 (m), 2875 (m), 1655 (s), 1464 (m), 1306 (m), 1118 (m), 1080

(m), 781 (s), 701 (s).
HRMS (ESI): Calcd for $C_{29}H_{35}O_2Si^+$ (M+H$^+$): 443.2406, Found: 443.2406.

(2-((tert-Butyldimethylsilyl)oxy)-2-phenylbicyclo[2.1.1]hexan-1-yl)(naphthalen-2-yl)methanone (3c)

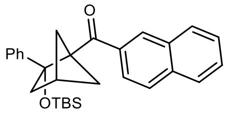

The title compound was prepared according to the General Procedure F with silyl enol ether **1c** and BCB **2a** in DCM for 2 h. Purification by flash column chromatography (0-10% ethyl acetate in hexane) provided the cycloaddition product **3c** (70 mg, 79% yield) as a colorless oil.
^{1}H NMR (500 MHz, CDCl$_3$) δ 8.39 – 8.35 (m, 1H), 8.04 (dd, *J* = 8.7, 1.7 Hz, 1H), 7.88 – 7.83 (m, 2H), 7.81 (d, *J* = 8.6 Hz, 1H), 7.57 (ddd, *J* = 8.2, 6.9, 1.3 Hz, 1H), 7.50 (ddd, *J* = 8.1, 6.9, 1.2 Hz, 1H), 7.35 – 7.27 (m, 2H), 7.24 – 7.16 (m, 3H), 3.06 (ddd, *J* = 11.9, 2.8, 1.6 Hz, 1H), 2.77 (dd, *J* = 9.2, 6.6 Hz, 1H), 2.56 (ddd, *J* = 2.9, 1.5 Hz, 1H), 2.27 (dt, *J* = 6.2, 2.9 Hz, 1H), 2.21 (ddd, *J* = 11.9, 3.4, 1.4 Hz, 1H), 1.87 – 1.76 (m, 2H), 0.88 (s, 9H), -0.09 (s, 3H), -0.37 (s, 3H).
^{13}C NMR (125 MHz, CDCl$_3$) δ 201.8, 142.8, 135.4, 135.3, 132.5, 131.8, 129.9, 128.2, 128.2, 127.8, 127.7, 127.6, 127.5, 126.4, 125.6, 86.4, 70.1, 44.8, 42.5, 42.4, 33.9, 26.5, 18.8, -2.6, -2.8.
ATR-IR ν (cm-1): 2957 (m), 2928 (m), 2857 (m), 1655 (s), 1306 (m), 1257 (s), 1054 (m), 833 (s), 776 (s), 702 (m).
HRMS (ESI): Calcd for $C_{29}H_{35}O_2Si^+$ (M+H$^+$): 443.2406, Found: 443.2410.

(2-((tert-Butyldiphenylsilyl)oxy)-2-phenylbicyclo[2.1.1]hexan-1-yl)(naphthalen-2-yl)methanone (3d)

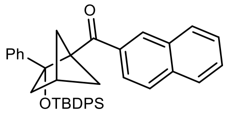

The title compound was prepared according to the General Procedure F with silyl enol ether **1d** and BCB **2a** in DCM for 2 h. Purification by flash column chromatography (0-10% ethyl acetate in hexane) provided the cycloaddition product **3d** (100 mg, 88% yield) as a colorless oil.
^{1}H NMR (500 MHz, CDCl$_3$) δ 8.37 (d, *J* = 1.7 Hz, 1H), 8.04 (dd, *J* = 8.6, 1.7 Hz, 1H), 7.89 (dd, *J* = 8.4, 4.3 Hz, 2H), 7.83 (d, *J* = 8.2 Hz, 1H), 7.59 (ddd, *J* = 8.1, 6.8, 1.3 Hz, 1H), 7.50 (ddd, *J* = 8.1, 6.8, 1.3 Hz, 1H), 7.35 – 7.27 (m, 3H), 7.16 (dt, *J* = 10.3, 7.5 Hz, 3H), 7.09 – 6.99 (m, 5H), 6.92 (t, *J* = 7.5 Hz, 4H), 2.94 (dd, *J* = 9.6, 6.5 Hz, 1H), 2.68 (dt, *J* = 12.5, 2.1 Hz, 1H), 2.49 – 2.44 (m, 1H), 2.35 (dt, *J* = 6.1, 2.8 Hz, 1H), 2.30 (ddd, *J* = 12.1, 3.6, 1.3 Hz, 1H), 1.85 – 1.78 (m, 1H), 1.68 (dd, *J* = 9.6, 7.8 Hz, 1H), 0.99 (s, 9H).
^{13}C NMR (125 MHz, CDCl$_3$) δ 202.0, 142.6, 136.5, 136.2, 136.0, 135.4, 134.6, 134.2, 132.5, 131.5, 129.9, 129.3, 128.9, 128.2, 128.1, 128.0, 127.8, 127.2, 127.1, 126.9, 126.5, 125.6, 87.6, 70.1, 44.8, 42.8, 42.3, 34.1, 27.8, 19.7.

ATR-IR ν (cm-1): 2965 (m), 2857 (m), 1656 (s), 1427 (m), 1306 (m), 1110 (s), 1053 (m), 781 (m), 699 (s).
HRMS (ESI): Calcd for $C_{39}H_{39}O_2Si^+$ ($M+H^+$): 567.2719, Found: 567.2729.

Naphthalen-2-yl(2-(o-tolyl)-2-((triisopropylsilyl)oxy)bicyclo[2.1.1]hexan-1-yl)methanone (3e)

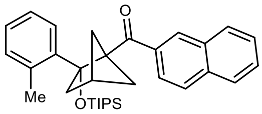

The title compound was prepared according to the General Procedure F with silyl enol ether **1e** and BCB **2a** in DCM within 3 h. Purification by flash column chromatography (0-10% ethyl acetate in hexane) provided the cycloaddition product **3e** (62 mg, 62% yield) as a colorless oil.
^{1}H NMR (500 MHz, $CDCl_3$) δ 8.03 – 7.78 (m, 2H), 7.78 (dd, *J* = 24.6, 8.4 Hz, 2H), 7.62 (d, *J* = 8.1 Hz, 2H), 7.54 (ddd, *J* = 8.2, 6.8, 1.3 Hz, 1H), 7.48 – 7.42 (m, 1H), 7.25 – 7.18 (m, 1H), 7.14 (td, *J* = 7.3, 1.2 Hz, 1H), 6.86 (d, *J* = 7.5 Hz, 1H), 3.37 (dt, *J* = 12.3, 2.3 Hz, 1H), 2.80 (dd, *J* = 9.7, 6.9 Hz, 1H), 2.51 (d, *J* = 1.7 Hz, 1H), 2.29 (ddd, *J* = 12.2, 3.6, 1.3 Hz, 1H), 2.14 (dd, *J* = 6.8, 3.2 Hz, 1H), 2.06 (s, 3H), 1.97 (dd, *J* = 9.7, 7.7 Hz, 1H), 1.86 – 1.79 (m, 1H), 1.19 – 1.08 (m, 3H), 1.02 (d, *J* = 7.4 Hz, 9H), 0.97 (d, *J* = 7.4 Hz, 9H).
^{13}C NMR (125 MHz, $CDCl_3$) δ 201.7, 140.2, 139.8, 135.2, 134.7, 133.0, 132.3, 131.5, 129.8, 128.3, 128.2, 127.7, 127.7, 127.7, 126.3, 125.1, 124.5, 88.4, 69.5, 44.8, 44.7, 44.0, 33.0, 23.5, 18.7, 18.4, 14.3.
ATR-IR ν (cm-1): 2945 (m), 2866 (m), 1655 (s), 1464 (m), 1307 (m), 1042 (m), 907 (m), 771 (s), 732 (s).
HRMS (ESI): Calcd for $C_{33}H_{42}O_2SiNa^+$ ($M+Na^+$): 521.2852, Found: 521.2841.

(2-(2-Fluorophenyl)-2-((triisopropylsilyl)oxy)bicyclo[2.1.1]hexan-1-yl)(naphthalen-2-yl)methanone (3f)

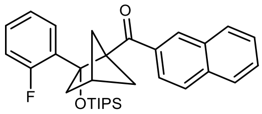

The title compound was prepared according to the General Procedure F with silyl enol ether **1f** and BCB **2a** in DCM within 3 h. Purification by flash column chromatography (0-10% ethyl acetate in hexane) provided the cycloaddition product **3f** (80 mg, 80% yield) as a colorless oil.
^{1}H NMR (500 MHz, $CDCl_3$) δ 8.48 (s, 1H), 8.08 (d, *J* = 8.2 Hz, 1H), 7.89 – 7.80 (m, 3H), 7.56 (ddd, *J* = 8.1, 6.8, 1.4 Hz, 1H), 7.53 – 7.48 (m, 1H), 7.42 (s, 1H), 7.20 (dd, *J* = 6.9, 6.1 Hz, 1H), 7.01 (s, 1H), 6.95 – 6.85 (m, 1H), 3.40 (s, 1H), 2.78 (dd, *J* = 9.6, 6.6 Hz, 1H), 2.55 (dt, *J* = 3.3, 1.6 Hz, 1H), 2.28 – 2.14 (m, 2H), 1.93 – 1.76 (m, 2H), 1.04 – 0.96 (m, 3H), 0.93 (d, *J* = 7.1 Hz, 9H), 0.88 (d, *J* = 7.2 Hz, 9H).
^{13}C NMR (125 MHz, $CDCl_3$) δ 200.4, 160.0 (d, *J* = 243.1 Hz), 134.3, 134.2, 131.5, 130.7, 129.2, 128.8 (d, *J* = 8.9 Hz), 128.7, 127.1, 126.8, 126.8, 125.3, 124.5, 122.2 (d, *J* = 3.5 Hz), 116.1, 115.9, 84.2 (d, *J* = 3.2 Hz), 69.0, 42.9, 42.8, 42.3, 32.5, 17.4, 17.4,

12.7.
^{19}F NMR (565 MHz, $CDCl_3$) δ -98.5, -110.7. (two peaks speculated from hindered rotation).
ATR-IR ν (cm-1): 2945 (s), 2867 (s), 1657 (s), 1308 (m), 1121 (m), 1052 (m), 780 (s).
HRMS (ESI): Calcd for $C_{32}H_{40}FO_2Si^+$ (M+H$^+$): 503.2782, Found: 503.2773.

(2-(3-Fluorophenyl)-2-((triisopropylsilyl)oxy)bicyclo[2.1.1]hexan-1-yl)(naphthalen-2-yl)methanone (3g)

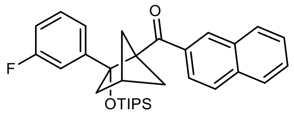

The title compound was prepared according to the General Procedure F with silyl enol ether **1g** and BCB **2a** in DCM within 3 h. Purification by flash column chromatography (0-10% ethyl acetate in hexane) provided the cycloaddition product **3g** (80 mg, 80% yield) as a white soild.
m.p.: 52 ℃.
^{1}H NMR (500 MHz, $CDCl_3$) δ 8.28 (s, 1H), 7.92 (dt, J = 8.7, 1.6 Hz, 1H), 7.87 – 7.77 (m, 3H), 7.56 (t, J = 7.5 Hz, 1H), 7.50 (t, J = 7.5 Hz, 1H), 7.19 – 7.12 (m, 2H), 7.08 (dd, J = 11.2, 2.4 Hz, 1H), 6.92 – 6.83 (m, 1H), 3.00 (d, J = 12.0 Hz, 1H), 2.85 (dd, J = 9.0, 6.7 Hz, 1H), 2.62 – 2.55 (m, 1H), 2.38 – 2.28 (m, 1H), 2.24 (dd, J = 12.0, 3.1 Hz, 1H), 1.93 – 1.79 (m, 2H), 1.01 – 0.91 (m, 3H), 0.88 (d, J = 7.2 Hz, 9H), 0.82 (d, J = 7.2 Hz, 9H).
^{13}C NMR (125 MHz, $CDCl_3$) δ 201.4, 162.3 (d, *J* = 245.0 Hz), 146.4 (d, *J* = 6.4 Hz), 135.6, 135.4, 132.4, 131.7, 129.7, 128.9 (d, *J* = 8.2 Hz), 128.2, 128.0, 127.8, 126.5, 125.1, 123.8 (d, *J* = 2.7 Hz), 115.3 (d, *J* = 22.6 Hz), 114.4 (d, *J* = 21.0 Hz), 86.1 (d, *J* = 1.8 Hz), 70.2, 44.7, 43.3, 42.5, 33.8, 18.4, 18.3, 13.5.
^{19}F NMR (471MHz, $CDCl_3$) δ -113.69.
ATR-IR ν (cm-1): 2946 (s), 2866 (s), 1655 (s), 1465 (m), 1310 (m), 1262 (m), 1119 (m), 882 (m), 784 (s), 697 (m).
HRMS (ESI): Calcd for $C_{32}H_{40}FO_2Si^+$ (M+H$^+$): 503.2782, Found: 503.2787.

(2-(4-Fluorophenyl)-2-((triisopropylsilyl)oxy)bicyclo[2.1.1]hexan-1-yl)(naphthalen-2-yl)methanone (3h)

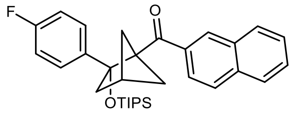

The title compound was prepared according to the General Procedure F with silyl enol ether **1h** and BCB **2a** in DCM within 2 h. Purification by flash column chromatography (0-10% ethyl acetate in hexane) provided the cycloaddition product **3h** (82mg, 82% yield) as a light yellow solid.
m.p.: 116 ℃.
^{1}H NMR (500 MHz, $CDCl_3$) δ 8.31 (d, J = 1.6 Hz, 1H), 7.93 (dd, J = 8.6, 1.7 Hz, 1H), 7.87 – 7.78 (m, 3H), 7.57 (ddd, J = 8.1, 6.8, 1.3 Hz, 1H), 7.51 (ddd, J = 8.2, 6.9, 1.3 Hz, 1H), 7.37 – 7.29 (m, 2H), 6.88 (t, J = 8.7 Hz, 2H), 3.09 – 2.99 (m, 1H), 2.84 (dd, J

= 9.5, 6.6 Hz, 1H), 2.57 (tt, J = 3.0, 1.5 Hz, 1H), 2.35 – 2.27 (m, 1H), 2.23 (ddd, J = 12.0, 3.5, 1.4 Hz, 1H), 1.91 – 1.83 (m, 1H), 1.80 (dd, J = 9.5, 7.9 Hz, 1H), 0.93 (ddd, J = 9.9, 6.8, 5.5 Hz, 3H), 0.88 (d, J = 6.9 Hz, 9H), 0.81 (d, J = 7.2 Hz, 9H).
^{13}C NMR (125 MHz, CDCl$_3$) δ 201.6, 162.2 (d, *J* = 246.6 Hz), 139.3 (d, *J* = 3.1 Hz), 135.4, 135.4, 132.5, 131.8, 129.8 (d, *J* = 8.1 Hz), 129.7, 128.3, 127.9, 127.8, 126.5, 125.2, 114.3 (d, *J* = 21.1 Hz), 86.1, 70.2, 44.6, 43.3, 42.7, 33.8, 18.4, 18.3, 13.5.
^{19}F NMR (471 MHz, CDCl$_3$) δ -115.45.
ATR-IR ν (cm-1): 2946 (s), 2867 (s), 1656 (s), 1509 (m), 1464 (m), 1312 (m), 1091 (m), 1053 (m), 779 (s).
HRMS (ESI): Calcd for $C_{32}H_{40}FO_2Si^+$ (M+H$^+$): 503.2782, Found: 503.2781.

(2-(4-Chlorophenyl)-2-((triisopropylsilyl)oxy)bicyclo[2.1.1]hexan-1-yl)(naphthalen-2-yl)methanone (3i)

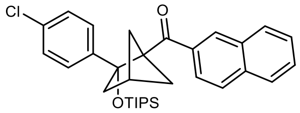

The title compound was prepared according to the General Procedure F with silyl enol ether **1i** and BCB **2a** in DCM within 2 h. Purification by flash column chromatography (0-10% ethyl acetate in hexane) provided the cycloaddition product **3i** (95 mg, 90% yield) as a colorless oil.
^{1}H NMR (500 MHz, CDCl$_3$) δ 8.31 (d, J = 1.6 Hz, 1H), 7.94 (dd, J = 8.6, 1.7 Hz, 1H), 7.88 – 7.77 (m, 3H), 7.57 (ddd, J = 8.1, 6.8, 1.3 Hz, 1H), 7.52 (ddd, J = 8.2, 6.9, 1.3 Hz, 1H), 7.29 (d, J = 8.7 Hz, 2H), 7.17 (d, J = 8.7 Hz, 2H), 3.03 (dt, J = 12.1, 1.6 Hz, 1H), 2.85 (dd, J = 9.6, 6.7 Hz, 1H), 2.59 – 2.55 (m, 1H), 2.35 – 2.27 (m, 1H), 2.23 (ddd, J = 12.0, 3.5, 1.4 Hz, 1H), 1.91 – 1.83 (m, 1H), 1.79 (dd, J = 9.6, 7.9 Hz, 1H), 0.98 – 0.91 (m, 3H), 0.88 (d, J = 7.0 Hz, 9H), 0.82 (d, J = 7.1 Hz, 9H).
^{13}C NMR (125 MHz, CDCl$_3$) (one peak on aromatic area is missing) δ 201.52, 142.02, 135.38, 133.49, 132.41, 131.86, 129.68, 129.44, 128.30, 127.95, 127.79, 127.60, 126.53, 125.15, 86.08, 70.20, 44.59, 43.14, 42.56, 33.81, 18.42, 18.31, 13.48.
ATR-IR ν (cm-1): 2944 (s), 2865 (m), 1655 (s), 1464 (m), 1260 (m), 1094 (s), 1053 (m), 914 (m), 809 (m), 673 (m).
HRMS (ESI): Calcd for $C_{32}H_{40}ClO_2Si^+$ (M+H$^+$): 519.2486, Found: 519.2482.

(2-(4-Bromophenyl)-2-((triisopropylsilyl)oxy)bicyclo[2.1.1]hexan-1-yl)(naphthalen-2-yl)methanone (3j)

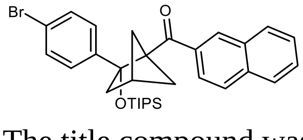

The title compound was prepared according to the General Procedure F with silyl enol ether **1j** and BCB **2a** in DCM within 2 h. Purification by flash column chromatography (0-10% ethyl acetate in hexane) provided the cycloaddition product **3j** (104 mg, 93% yield) as a colorless oil.
^{1}H NMR (500 MHz, CDCl$_3$) δ 8.29 (d, J = 1.6 Hz, 1H), 7.93 (dd, J = 8.6, 1.7 Hz, 1H), 7.87 – 7.78 (m, 3H), 7.57 (ddd, J = 8.2, 6.8, 1.3 Hz, 1H), 7.52 (ddd, J = 8.1, 6.8, 1.3

Hz, 1H), 7.32 (d, J = 8.6 Hz, 2H), 7.23 (d, J = 8.7 Hz, 2H), 3.06 – 2.97 (m, 1H), 2.84 (dd, J = 9.6, 6.8 Hz, 1H), 2.61 – 2.54 (m, 1H), 2.34 – 2.27 (m, 1H), 2.22 (ddd, J = 12.0, 3.5, 1.4 Hz, 1H), 1.90 – 1.83 (m, 1H), 1.79 (dd, J = 9.6, 7.9 Hz, 1H), 0.98 – 0.90 (m, 3H), 0.88 (d, J = 6.9 Hz, 9H), 0.81 (d, J = 7.1 Hz, 9H).
^{13}C NMR (125 MHz, $CDCl_3$) (one peak on aromatic area is missing) δ 201.5, 142.5, 135.4, 132.4, 131.9, 130.6, 129.8, 129.7, 128.3, 128.0, 127.8, 126.5, 125.1, 121.8, 86.1, 70.2, 44.6, 43.1, 42.6, 33.8, 18.4, 18.3, 13.5.
ATR-IR ν (cm-1): 2945 (s), 2866 (s), 1655 (s), 1464 (m), 1080 (s), 882 (m), 808 (m), 673 (m).
HRMS (ESI): Calcd for $C_{32}H_{39}BrO_2SiNa^+$ (M+Na$^+$): 585.1801, Found: 585.1827.

(2-(4-(tert-Butyl)phenyl)-2-((triisopropylsilyl)oxy)bicyclo[2.1.1]hexan-1-yl)(naphthalen-2-yl)methanone (3k)

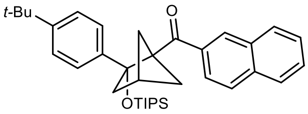

The title compound was prepared according to the General Procedure F with silyl enol ether **1k** and BCB **2a** in DCM within 3 h. Purification by flash column chromatography (0-10% ethyl acetate in hexane) provided the cycloaddition product **3k** (47mg, 44% yield) as a colorless oil.
^{1}H NMR (600 MHz, $CDCl_3$) δ 8.12 – 8.06 (m, 1H), 7.86 (dd, J = 8.6, 1.7 Hz, 1H), 7.80 (dd, J = 8.1, 1.2 Hz, 1H), 7.76 – 7.71 (m, 2H), 7.53 (ddd, J = 8.2, 6.8, 1.3 Hz, 1H), 7.46 (ddd, J = 8.1, 6.8, 1.2 Hz, 1H), 7.26 (d, 2H), 7.14 (d, J = 8.5 Hz, 2H), 3.07 (ddd, J = 11.9, 2.8, 1.6 Hz, 1H), 2.86 – 2.77 (m, 1H), 2.59 – 2.50 (m, 1H), 2.29 – 2.21 (m, 1H), 2.21 – 2.13 (m, 1H), 1.90 – 1.85 (m, 2H), 1.18 (s, 9H), 0.98 – 0.90 (m, 3H), 0.85 (d, J = 7.4 Hz, 9H), 0.82 (d, J = 7.4 Hz, 9H).
^{13}C NMR (150 MHz, $CDCl_3$) δ 201.9, 150.4, 140.3, 135.6, 135.2, 132.4, 131.7, 129.8, 128.0, 127.8, 127.7, 127.7, 126.2, 125.3, 124.3, 86.2, 70.0, 44.6, 43.1, 42.8, 34.4, 33.8, 31.4, 18.4, 18.3, 13.6.
ATR-IR ν (cm-1): 2961 (s), 2866 (s), 1656 (s), 1464 (m), 1306 (m), 1115 (m), 1056 (m), 788 (s), 674 (m).
HRMS (ESI): Calcd for $C_{36}H_{49}O_2Si^+$ (M+H$^+$): 541.3502, Found: 541.3495.

Methyl-4-(1-(2-naphthoyl)-2((triisopropylsilyl)oxy)bicyclo[2.1.1]hexan-2-yl)benzoate (3l)

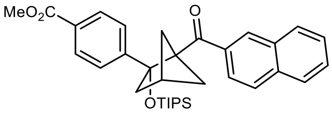

The title compound was prepared according to the General Procedure F with silyl enol ether **1l** and BCB **2a** in DCM within 2 h. Purification by flash column chromatography (5-10% ethyl acetate in hexane) provided the cycloaddition product **3l** (78 mg, 72% yield) as a white solid.
m.p.: 133 ℃.
^{1}H NMR (500 MHz, $CDCl_3$) δ 8.32 (d, J = 1.7 Hz, 1H), 7.93 (dd, J = 8.6, 1.8 Hz, 1H),

7.90 – 7.77 (m, 5H), 7.60 – 7.53 (m, 1H), 7.54 – 7.46 (m, 1H), 7.43 (d, J = 8.3 Hz, 2H), 3.86 (s, 3H), 3.10 (dt, J = 11.9, 2.2 Hz, 1H), 2.87 (dd, J = 9.3, 6.6 Hz, 1H), 2.62 – 2.57 (m, 1H), 2.33 (dt, J = 6.3, 2.9 Hz, 1H), 2.25 (dd, J = 11.9, 3.3 Hz, 1H), 1.93 – 1.75 (m, 2H), 0.97 – 0.87 (m, 3H), 0.86 (d, J = 7.1 Hz, 9H), 0.78 (d, J = 7.2 Hz, 9H).
^{13}C NMR (125 MHz, CDCl$_3$) δ 201.4, 167.0, 148.6, 135.4, 135.4, 132.4, 131.9, 129.7, 129.2, 128.8, 128.3, 128.1, 128.0, 127.8, 126.5, 125.1, 86.3, 70.3, 52.1, 44.6, 43.1, 42.4, 33.9, 18.4, 18.3, 13.5.
ATR-IR ν (cm-1): 2941 (s), 2865 (s), 1655 (s), 1464 (m), 1308 (m), 1125 (m), 882 (m), 782 (s), 674 (m).
HRMS (ESI): Calcd for $C_{34}H_{43}O_4Si^+$ (M+H$^+$): 543.2931, Found: 543.2938.

(2-(4-((tert-Butyldimethylsilyl)oxy)phenyl)-2-((triisopropylsilyl)oxy)bicyclo[2.1.1]hexan-1-yl)(naphthalen-2-yl)methanone (3m)

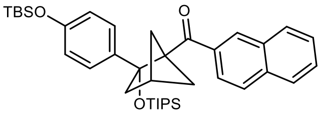

The title compound was prepared according to the General Procedure F with silyl enol ether **1m** and BCB **2a** in DCM within 2 h. Purification by flash column chromatography (0-10% ethyl acetate in hexane) provided the cycloaddition product **3m** (100 mg, 81% yield) as a colorless oil.
^{1}H NMR (600 MHz, CDCl$_3$) δ 8.25 (d, J = 1.7 Hz, 1H), 7.86 (dd, J = 8.6, 1.7 Hz, 1H), 7.83 – 7.79 (m, 2H), 7.76 (d, J = 8.6 Hz, 1H), 7.54 (ddd, J = 8.3, 6.8, 1.2 Hz, 1H), 7.48 (ddd, J = 7.9, 6.8, 1.2 Hz, 1H), 7.22 (d, J = 8.7 Hz, 2H), 6.64 (d, J = 8.7 Hz, 2H), 3.06 – 2.98 (m, 1H), 2.82 (ddd, J = 8.8, 6.5, 1.0 Hz, 1H), 2.58 – 2.49 (m, 1H), 2.30 – 2.21 (m, 1H), 2.17 (ddd, J = 12.0, 3.2, 1.4 Hz, 1H), 1.90 – 1.80 (m, 2H), 0.97 – 0.90 (m, 12H), 0.88 (d, J = 6.9 Hz, 9H), 0.82 (d, J = 7.2 Hz, 9H), 0.08 (d, J = 1.4 Hz, 6H).
^{13}C NMR (150 MHz, CDCl$_3$) δ 201.8, 155.0, 136.4, 135.6, 135.2, 132.5, 131.7, 129.8, 129.3, 128.1, 127.8, 127.7, 126.4, 125.3, 119.0, 86.1, 70.1, 44.7, 43.2, 42.7, 33.8, 25.8, 18.5, 18.4, 18.3, 13.5, -4.4, -4.4.
ATR-IR ν (cm-1): 2949 (s), 2865 (s), 1656 (s), 1509 (s), 1463 (m), 1254 (s), 1053 (m), 914 (s), 839 (m), 778 (s).
HRMS (ESI): Calcd for $C_{38}H_{54}O_3Si_2Na^+$ (M+Na$^+$): 637.3509, Found: 637.3516.

(2-(4-Ethynylphenyl)-2-((triisopropylsilyl)oxy)bicyclo[2.1.1]hexan-1-yl)(naphthalen-2-yl)methanone (3n)

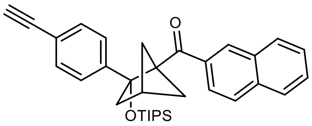

The title compound was prepared according to the General Procedure F with silyl enol ether **1n** and BCB **2a** in DCM within 2 h. Purification by flash column chromatography (3-10% ethyl acetate in hexane) provided the cycloaddition product **3n** (79 mg, 78% yield) as a white solid.
m.p.: 136 ℃.

^{1}H NMR (600 MHz, CDCl$_3$) δ 8.33 – 8.28 (m, 1H), 7.92 (dd, J = 8.6, 1.7 Hz, 1H), 7.87 – 7.82 (m, 2H), 7.80 (d, J = 8.6 Hz, 1H), 7.57 (ddd, J = 8.1, 6.9, 1.3 Hz, 1H), 7.51 (ddd, J = 8.1, 6.8, 1.2 Hz, 1H), 7.36 – 7.30 (m, 4H), 3.06 (ddd, J = 12.0, 2.8, 1.6 Hz, 1H), 3.02 (s, 1H), 2.85 (dd, J = 9.4, 6.6 Hz, 1H), 2.59 – 2.54 (m, 1H), 2.33 – 2.27 (m, 1H), 2.22 (ddd, J = 12.0, 3.5, 1.4 Hz, 1H), 1.88 – 1.82 (m, 1H), 1.80 (dd, J = 9.5, 7.9 Hz, 1H), 0.97 – 0.89 (m, 3H), 0.87 (d, J = 7.2 Hz, 9H), 0.81 (d, J = 7.3 Hz, 9H).
^{13}C NMR (150 MHz, CDCl$_3$) δ 201.5, 144.2, 135.4, 135.4, 132.4, 131.8, 131.3, 129.7, 128.3, 128.0, 127.9, 127.8, 126.5, 125.2, 121.3, 86.3, 83.6, 77.4, 70.2, 44.7, 43.0, 42.5, 33.8, 18.4, 18.3, 13.5.
ATR-IR ν (cm-1): 2946 (s), 2866 (s), 1655 (s), 1464 (m), 1315 (m), 1093 (m), 1054 (s), 882 (m), 789 (m), 674 (m).
HRMS (ESI): Calcd for $C_{34}H_{40}O_2SiNa^+$ (M+Na$^+$): 531.2695, Found: 531.2697.

(2-(Benzo[d][1,3]dioxol-5-yl)-2-((triisopropylsilyl)oxy)bicyclo[2.1.1]hexan-1-yl)(naphthalen-2-yl)methanone (3o)

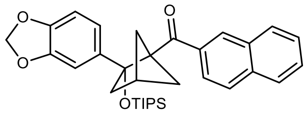

The title compound was prepared according to the General Procedure F with silyl enol ether **1o** and BCB **4** in DCM within 2 h. Purification by flash column chromatography (3-10% ethyl acetate in hexane) provided the cycloaddition product **3o** (55 mg, 52% yield) as a white solid.
m.p.: 102 ℃.
^{1}H NMR (500 MHz, CDCl$_3$) δ 8.23 (d, J = 1.7 Hz, 1H), 7.93 (dd, J = 8.6, 1.7 Hz, 1H), 7.81 (q, J = 8.4 Hz, 3H), 7.55 (ddd, J = 8.1, 6.8, 1.3 Hz, 1H), 7.49 (ddd, J = 8.1, 6.8, 1.3 Hz, 1H), 6.87 (dd, J = 8.2, 2.0 Hz, 1H), 6.79 (d, J = 1.9 Hz, 1H), 6.64 (d, J = 8.2 Hz, 1H), 5.85 (d, J = 1.6 Hz, 1H), 5.69 (d, J = 1.6 Hz, 1H), 2.97 (ddd, J = 11.9, 2.8, 1.6 Hz, 1H), 2.86 – 2.76 (m, 1H), 2.58 – 2.51 (m, 1H), 2.31 – 2.24 (m, 1H), 2.20 (dt, J = 11.9, 2.1 Hz, 1H), 1.91 – 1.84 (m, 2H), 1.00 – 0.91 (m, 3H), 0.89 (d, J = 7.0 Hz, 9H), 0.83 (d, J = 7.2 Hz, 9H).
^{13}C NMR (125 MHz, CDCl$_3$) δ 201.7, 147.1, 146.8, 137.7, 135.6, 135.3, 132.5, 131.8, 129.7, 128.1, 127.9, 127.8, 126.4, 125.2, 121.3, 109.2, 107.0, 101.0, 86.5, 70.4, 44.6, 43.5, 42.7, 33.7, 18.5, 18.4, 13.6.
ATR-IR ν (cm-1): 2944 (m), 2866 (s), 1655 (s), 1488 (s), 1236 (s), 1117 (m), 1042 (s), 883 (m), 778 (m).
HRMS (ESI): Calcd for $C_{33}H_{40}O_4SiNa^+$ (M+Na$^+$): 551.2593, Found: 551.2591.

(2-(4-Chloro-3-methylphenyl)-2-((triisopropylsilyl)oxy)bicyclo[2.1.1]hexan-1-yl)(naphthalen-2-yl)methanone (3p)

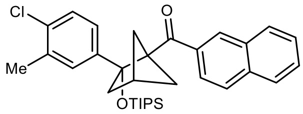

The title compound was prepared according to the General Procedure F with silyl enol ether **1p** and BCB **2a** in DCM within 2 h. Purification by flash column

chromatography (0-10% ethyl acetate in hexane) provided the cycloaddition product **3p** (90 mg, 84% yield) as a white solid.
m.p.: 103 °C.
^{1}H NMR (500 MHz, CDCl$_3$) δ 8.23 (d, J = 1.7 Hz, 1H), 7.91 (dd, J = 8.6, 1.7 Hz, 1H), 7.86 – 7.76 (m, 3H), 7.57 (ddd, J = 8.2, 6.8, 1.3 Hz, 1H), 7.51 (ddd, J = 8.1, 6.8, 1.3 Hz, 1H), 7.19 – 7.14 (m, 2H), 7.12 (dd, J = 8.4, 2.4 Hz, 1H), 3.01 (ddd, J = 11.9, 2.8, 1.7 Hz, 1H), 2.84 (dd, J = 9.5, 6.6 Hz, 1H), 2.60 – 2.53 (m, 1H), 2.34 – 2.27 (m, 1H), 2.20 (ddd, J = 11.9, 3.5, 1.4 Hz, 1H), 2.13 (s, 3H), 1.92 – 1.85 (m, 1H), 1.81 (dd, J = 9.5, 7.8 Hz, 1H), 0.95 – 0.88 (m, 3H), 0.86 (d, J = 6.6 Hz, 9H), 0.80 (d, J = 7.0 Hz, 9H).
^{13}C NMR (125 MHz, CDCl$_3$) δ 201.6, 141.9, 135.7, 135.3, 134.8, 133.5, 132.4, 131.8, 131.2, 129.7, 128.2, 127.9, 127.9, 127.8, 126.6, 126.5, 125.1, 86.0, 70.3, 44.5, 43.1, 42.5, 33.8, 20.0, 18.4, 18.3, 13.5.
ATR-IR ν (cm-1): 2945 (m), 2866 (s), 1655 (s), 1465 (m), 1311 (m), 1118 (s), 1047 (m), 789 (s), 673 (m).
HRMS (ESI): Calcd for $C_{33}H_{41}ClO_2Si^+$ (M+H$^+$): 533.2643, Found: 533.2620.

Naphthalen-2-yl(2-(naphthalen-2-yl)-2-((triisopropylsilyl)oxy)bicyclo[2.1.1]hexan-1-yl)methanone (3q)

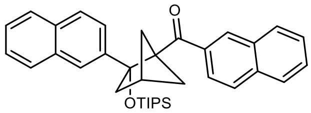

The title compound was prepared according to the General Procedure F with silyl enol ether **1q** and BCB **2a** in toluene within 2 h. Purification by flash column chromatography (0-10% ethyl acetate in hexane) provided the cycloaddition product **3q** (74 mg, 69% yield) as a white foam.
^{1}H NMR (600 MHz, CDCl$_3$) δ 8.24 (d, J = 1.7 Hz, 1H), 7.94 (dd, J = 8.6, 1.7 Hz, 1H), 7.88 (d, J = 1.9 Hz, 1H), 7.81 (dd, J = 8.2, 1.2 Hz, 1H), 7.77 (d, J = 8.3 Hz, 2H), 7.70 (dd, J = 7.7, 1.6 Hz, 1H), 7.65 (d, J = 8.2 Hz, 1H), 7.58 (d, J = 8.7 Hz, 1H), 7.53 (ddd, J = 8.1, 6.8, 1.2 Hz, 1H), 7.46 – 7.39 (m, 4H), 3.26 (dt, J = 12.1, 2.0 Hz, 1H), 2.92 (dd, J = 9.5, 6.5 Hz, 1H), 2.67 – 2.60 (m, 1H), 2.37 – 2.27 (m, 2H), 1.93 (dd, J = 9.6, 7.7 Hz, 1H), 1.90 – 1.84 (m, 1H), 1.00 – 0.90 (m, 3H), 0.87 (d, J = 7.4 Hz, 9H), 0.80 (d, J = 7.4 Hz, 9H).
^{13}C NMR (150 MHz, CDCl$_3$) δ 201.8, 140.8, 135.6, 135.3, 132.8, 132.6, 132.4, 131.8, 129.6, 128.4, 128.1, 127.8, 127.7, 127.6, 127.1, 127.0, 126.3, 126.1, 126.0, 126.0, 125.3, 86.7, 70.3, 44.7, 43.1, 42.7, 33.8, 18.5, 18.4, 13.5.
ATR-IR ν (cm-1): 2945 (m), 2866 (m), 1656 (s), 1465 (m), 1308 (m), 1194 (m), 1118 (m), 807 (m), 744 (m), 674 (m).
HRMS (ESI): Calcd for $C_{36}H_{42}O_2SiNa^+$ (M+Na$^+$): 557.2852, Found: 557.2847.

(2-(Naphthalen-1-yl)-2-((triisopropylsilyl)oxy)bicyclo[2.1.1]hexan-1-yl)(naphthalen-2-yl)methanone (3r)

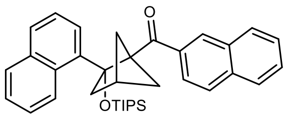

The title compound was prepared according to the General Procedure F with silyl enol ether **1r** and BCB **2a** in DCM within 4 h. Purification by flash column chromatography (0-10% ethyl acetate in hexane) provided the cycloaddition product **3r** (78 mg, 73% yield) as a white solid.

m.p.: 135 ℃.

^{1}H NMR (600 MHz, CDCl$_3$) δ 8.49 (d, J = 8.8 Hz, 1H), 8.08 – 7.68 (m, 5H), 7.68 – 7.57 (bd, 2H), 7.56 – 7.48 (m, 2H), 7.46 (t, J = 7.7 Hz, 1H), 7.44 – 7.40 (m, 1H), 7.05 (t, J = 7.4 Hz, 1H), 6.75 (t, J = 7.8 Hz, 1H), 3.53 (dt, J = 12.2, 2.3 Hz, 1H), 2.97 (dd, J = 9.6, 6.7 Hz, 1H), 2.62 – 2.56 (m, 1H), 2.37 (ddd, J = 12.3, 3.6, 1.4 Hz, 1H), 2.26 (dt, J = 6.5, 2.9 Hz, 1H), 1.98 (dd, J = 9.7, 7.7 Hz, 1H), 1.88 (dt, J = 7.3, 3.3 Hz, 1H), 1.01 – 0.88 (m, 12H), 0.80 (d, J = 6.8 Hz, 9H).

^{13}C NMR (150 MHz, CDCl$_3$) δ 201.6, 138.5, 135.0, 135.0, 134.5, 133.0, 132.2, 131.2, 130.1, 129.7, 129.6, 128.2, 128.0, 127.6, 127.5, 126.2, 126.0, 125.3, 125.0, 124.0, 123.9, 88.7, 69.8, 45.3, 45.0, 44.5, 33.0, 18.5, 18.3, 14.0.

ATR-IR ν (cm-1): 2945 (m), 2866 (s), 1655 (s), 1464 (m), 1302 (m), 1062 (m), 883 (w), 773 (s), 677 (m).

HRMS (ESI): Calcd for $C_{36}H_{43}O_2Si^+$ (M+H$^+$): 535.3032, Found: 535.3022.

(2-(Furan-2-yl)-2-((triisopropylsilyl)oxy)bicyclo[2.1.1]hexan-1-yl)(naphthalen-2-yl)methanone (3s)

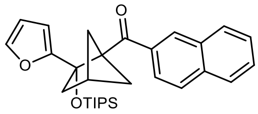

The title compound was prepared according to the General Procedure F with silyl enol ether **1s** and BCB **2a** in toluene within 2 h. Purification by flash column chromatography (0-10% ethyl acetate in hexane) provided the cycloaddition product **3s** (80 mg, 84% yield) as a light yellow oil.

^{1}H NMR (600 MHz, CDCl$_3$) δ 8.21 (d, J = 1.7 Hz, 1H), 7.92 (dd, J = 8.6, 1.7 Hz, 1H), 7.85 – 7.81 (m, 2H), 7.78 (d, J = 8.6 Hz, 1H), 7.55 (ddd, J = 8.0, 6.8, 1.3 Hz, 1H), 7.49 (ddd, J = 8.2, 6.8, 1.2 Hz, 1H), 7.08 (dd, J = 1.8, 0.9 Hz, 1H), 6.32 – 6.24 (m, 2H), 2.79 – 2.70 (m, 2H), 2.56 – 2.47 (m, 1H), 2.31 – 2.24 (m, 1H), 2.20 (ddd, J = 11.6, 3.6, 1.5 Hz, 1H), 2.01 – 1.95 (m, 1H), 1.85 (dd, J = 9.6, 7.5 Hz, 1H), 0.89 (d, J = 6.9 Hz, 9H), 0.87 (d, J = 6.7 Hz, 9H), 0.85 – 0.80 (m, 3H).

^{13}C NMR (150 MHz, CDCl$_3$) δ 201.4, 155.8, 141.5, 135.5, 135.2, 132.5, 131.3, 129.7, 128.0, 127.7, 127.7, 126.3, 125.3, 110.3, 108.7, 81.5, 69.0, 45.6, 43.7, 43.5, 33.6, 18.3, 18.2, 13.1.

ATR-IR ν (cm-1): 2946 (m), 2866 (s),1658 (s), 1465 (m), 1311 (m), 1262 (m), 1155 (m), 1023 (m), 777 (s), 675 (m).

HRMS (ESI): Calcd for $C_{30}H_{39}O_3Si^+$ (M+H$^+$): 475.2668, Found: 475.2670.

Naphthalen-2-yl(2-(thiophen-3-yl)-2-((triisopropylsilyl)oxy)bicyclo[2.1.1]hexan-

1-yl)methanone (3t)

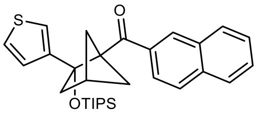

The title compound was prepared according to the General Procedure F with silyl enol ether **1t** and BCB **2a** in toluene within 2 h. Purification by flash column chromatography (0-10% ethyl acetate in hexane) provided the cycloaddition product **3t** (85 mg, 86% yield) as a colorless oil.

^{1}H NMR (500 MHz, $CDCl_3$) δ 8.22 (d, J = 1.7 Hz, 1H), 7.93 (dd, J = 8.6, 1.7 Hz, 1H), 7.85 – 7.77 (m, 3H), 7.56 (ddd, J = 8.1, 6.8, 1.3 Hz, 1H), 7.50 (ddd, J = 8.1, 6.8, 1.2 Hz, 1H), 7.14 (dd, J = 2.9, 1.4 Hz, 1H), 7.04 (dd, J = 5.0, 2.9 Hz, 1H), 6.84 (dd, J = 5.0, 1.4 Hz, 1H), 2.84 (ddd, J = 11.6, 2.7, 1.6 Hz, 1H), 2.79 (dd, J = 9.5, 6.7 Hz, 1H), 2.57 – 2.52 (m, 1H), 2.32 – 2.24 (m, 2H), 1.95 – 1.90 (m, 1H), 1.86 (dd, J = 9.5, 7.6 Hz, 1H), 0.98 – 0.91 (m, 3H), 0.88 (d, J = 6.8 Hz, 9H), 0.83 (d, J = 7.1 Hz, 9H).

^{13}C NMR (125 MHz, $CDCl_3$) δ 201.8, 146.1, 135.4, 135.3, 132.4, 131.9, 129.8, 128.9, 128.2, 127.8, 127.7, 126.4, 125.2, 124.7, 122.2, 84.0, 69.5, 45.7, 44.1, 43.4, 33.7, 18.4, 18.2, 13.4.

ATR-IR ν (cm-1): 2945 (m), 2866 (s), 1655 (s), 1465 (m), 1308 (m), 1118 (m), 1056 (m), 782 (s), 655 (m).

HRMS (ESI): Calcd for $C_{30}H_{39}O_2SSi^+$ (M+H$^+$): 491.2440, Found: 491.2437.

Naphthalen-2-yl(2-styryl-2-((triisopropylsilyl)oxy)bicyclo[2.1.1]hexan-1-yl)methanone (3u)

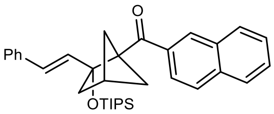

The title compound was prepared according to the General Procedure F with silyl enol ether **1u** and BCB **2a** in toluene within 3 h. Purification by flash column chromatography (0-10% ethyl acetate in hexane) provided the cycloaddition product **3u** (81 mg, 79% yield) as a colorless oil.

^{1}H NMR (500 MHz, $CDCl_3$) δ 8.49 (d, *J* = 1.6 Hz, 1H), 8.06 (dd, *J* = 8.7, 1.7 Hz, 1H), 7.84 (t, *J* = 9.0 Hz, 2H), 7.79 (d, *J* = 8.1 Hz, 1H), 7.56 (ddd, *J* = 8.2, 6.7, 1.2 Hz, 1H), 7.50 – 7.44 (m, 1H), 7.29 – 7.16 (m, 5H), 6.62 (d, *J* = 16.3 Hz, 1H), 6.34 (d, *J* = 16.3 Hz, 1H), 2.76 (dt, *J* = 11.3, 2.2 Hz, 1H), 2.71 (dd, *J* = 9.5, 6.7 Hz, 1H), 2.50 (t, *J* = 3.0 Hz, 1H), 2.26 (dt, *J* = 6.2, 2.9 Hz, 1H), 2.07 – 1.95 (m, 2H), 1.90 (dd, *J* = 9.5, 7.2 Hz, 1H), 1.04 – 0.90 (m, 21H).

^{13}C NMR (125 MHz, $CDCl_3$) δ 202.1, 136.8, 135.6, 135.3, 132.8, 132.5, 131.8, 130.0, 129.7, 128.7, 128.2, 127.9, 127.8, 127.7, 126.8, 126.5, 125.2, 84.2, 68.9, 44.0, 43.2, 42.1, 33.7, 18.4, 18.4, 13.2.

ATR-IR ν (cm-1): 2944 (s), 2864 (s), 1654 (s), 1626 (m), 1463 (s), 1310 (s), 1195 (s), 1118 (s), 1093 (s), 882 (s), 743 (s).

HRMS (ESI): Calcd for $C_{34}H_{43}O_2Si^+$ (M+H$^+$): 511.3032, Found: 511.3020.

Naphthalen-2-yl(2-((triisopropylsilyl)oxy)-2-

((trimethylsilyl)ethynyl)bicyclo[2.1.1]hexan-1-yl)methanone (3v)

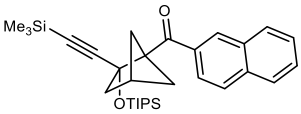

The title compound was prepared according to the General Procedure F with silyl enol ether **1v** and BCB **2a** in toluene within 12 h. Purification by flash column chromatography (0-10% ethyl acetate in hexane) provided the cycloaddition product **3v** (90 mg, 89% yield) as a colorless oil.

^{1}H NMR (500 MHz, CDCl$_3$) δ 8.48 – 8.44 (m, 1H), 8.03 (dd, J = 8.6, 1.7 Hz, 1H), 7.89 (d, J = 8.1 Hz, 1H), 7.82 (dd, J = 15.7, 8.4 Hz, 2H), 7.59 – 7.53 (m, 1H), 7.53 – 7.48 (m, 1H), 2.54 (ddd, J = 10.8, 2.8, 1.5 Hz, 1H), 2.47 (td, J = 3.1, 1.5 Hz, 1H), 2.43 (dd, J = 9.2, 7.0 Hz, 1H), 2.27 – 2.11 (m, 4H), 1.18 – 1.07 (m, 3H), 0.99 (d, J = 7.5 Hz, 9H), 0.95 (d, J = 7.5 Hz, 9H), -0.06 (s, 9H).

^{13}C NMR (125 MHz, CDCl$_3$) δ 200.1, 135.7, 135.4, 132.4, 131.8, 129.8, 128.1, 127.8, 127.6, 126.3, 125.5, 108.1, 91.3, 76.3, 69.4, 51.4, 45.0, 41.9, 34.2, 18.5, 18.4, 12.9, -0.5.

ATR-IR ν (cm-1): 2945 (m), 2865 (m), 1660 (s), 1464 (m), 1312 (m), 1249 (m), 1118 (s), 843 (s), 776 (m).

HRMS (ESI): Calcd for $C_{31}H_{44}O_2Si_2Na^+$ (M+Na$^+$): 527.2778, Found: 527.2759.

(2-Methyl-2-((triisopropylsilyl)oxy)bicyclo[2.1.1]hexan-1-yl)(naphthalen-2-yl)methanone (3w)

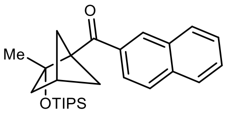

The title compound was prepared according to the General Procedure F with silyl enol ether **1w** and BCB **2a** in toluene within 2 h. Purification by flash column chromatography (0-10% ethyl acetate in hexane) provided the cycloaddition product **3w** (76 mg, 90% yield) as a colorless oil.

^{1}H NMR (500 MHz, CDCl$_3$) δ 8.53 (d, J = 1.6 Hz, 1H), 8.07 (dd, J = 8.7, 1.7 Hz, 1H), 7.90 (d, J = 8.1 Hz, 1H), 7.83 (t, J = 9.2 Hz, 2H), 7.56 (t, J = 7.5 Hz, 1H), 7.52 (t, J = 7.5 Hz, 1H), 2.69 (dd, J = 9.6, 6.4 Hz, 1H), 2.42 (t, J = 3.2 Hz, 1H), 2.24 – 2.18 (m, 1H), 2.11 – 2.01 (m, 2H), 2.00 – 1.94 (m, 1H), 1.88 (dd, J = 9.6, 7.5 Hz, 1H), 1.58 (s, 3H), 1.04 – 0.88 (m, 21H).

^{13}C NMR (125 MHz, CDCl$_3$) δ 203.0, 135.4, 135.2, 132.3, 131.7, 129.5, 128.0, 127.7, 127.6, 126.3, 125.0, 82.2, 68.6, 47.6, 44.8, 43.1, 33.9, 25.7, 18.3, 13.3.

ATR-IR ν (cm-1): 2944 (s), 2865 (s), 1653 (s), 1464 (m), 1310 (m), 1168 (m), 1041 (s), 882 (m), 757 (s), 674 (m).

HRMS (ESI): Calcd for $C_{27}H_{39}O_2Si^+$ (M+H$^+$): 423.2719, Found: 423.2740.

(2-(Cyclohexylmethyl)-2-((triisopropylsilyl)oxy)bicyclo[2.1.1]hexan-1-yl)(naphthalen-2-yl)methanone (3x)

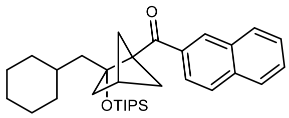

The title compound was prepared according to the General Procedure F with silyl enol ether **1x** and BCB **2a** in toluene within 3 h. Purification by flash column chromatography (0-10% ethyl acetate in hexane) provided the cycloaddition product **3x** (74 mg, 73% yield) as a colorless oil.

^{1}H NMR (500 MHz, CDCl$_3$) δ 8.45 (s, 1H), 8.00 (dd, *J* = 8.6, 1.8 Hz, 1H), 7.89 (d, *J* = 8.0 Hz, 1H), 7.83 (dd, *J* = 12.2, 8.3 Hz, 2H), 7.58 – 7.47 (m, 2H), 2.69 – 2.63 (m, 1H), 2.41 (s, 1H), 2.33 (d, *J* = 11.1 Hz, 1H), 2.15 (dt, *J* = 6.1, 2.9 Hz, 1H), 1.98 (d, *J* = 7.7 Hz, 3H), 1.84 (dt, *J* = 14.6, 4.9 Hz, 2H), 1.79 – 1.55 (m, 6H), 1.27 – 1.12 (m, 3H), 1.08 – 0.86 (m, 23H).

^{13}C NMR (125 MHz, CDCl$_3$) δ 203.5, 136.3, 135.3, 132.5, 131.3, 129.5, 128.1, 127.8, 127.8, 126.5, 125.4, 85.9, 69.3, 47.9, 45.5, 44.0, 43.9, 35.6, 35.5, 34.8, 33.8, 26.7, 26.6, 26.5, 18.8, 18.8, 14.2.

ATR-IR ν (cm-1): 2922 (s), 2865 (s), 1654 (s), 1464 (m), 1308 (m), 1116 (s), 882 (m), 672 (s).

HRMS (ESI): Calcd for $C_{33}H_{48}O_2SiNa^+$ (M+Na$^+$): 527.3322, Found: 527.3317.

(2-(3-Chloropropyl)-2-((triisopropylsilyl)oxy)bicyclo[2.1.1]hexan-1-yl)(naphthalen-2-yl)methanone (3y)

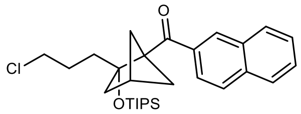

The title compound was prepared according to the General Procedure F with silyl enol ether **1y** and BCB **2a** in toluene within 4 h. Purification by flash column chromatography (0-10% ethyl acetate in hexane) provided the cycloaddition product **3y** (46 mg, 47% yield) with isomers from regioisomeric silyl enol ethers of 12:1:1 ratio as a yellow oil.

^{1}H NMR (600 MHz, CDCl$_3$) δ 8.51 – 8.48 (m, 1H), 8.03 (dd, *J* = 8.6, 1.7 Hz, 1H), 7.90 (d, *J* = 8.1 Hz, 1H), 7.84 (t, *J* = 8.7 Hz, 2H), 7.57 (ddd, *J* = 8.2, 6.9, 1.3 Hz, 1H), 7.54 – 7.49 (m, 1H), 3.51 – 3.41 (m, 2H), 2.70 (dd, *J* = 9.6, 6.5 Hz, 1H), 2.45 – 2.38 (m, 1H), 2.25 – 2.18 (m, 1H), 2.16 – 2.00 (m, 4H), 1.99 – 1.89 (m, 3H), 1.83 – 1.72 (m, 1H), 1.07 (h, *J* = 7.1, 6.7 Hz, 3H), 1.01 – 0.95 (m, 18H).

^{13}C NMR (150 MHz, CDCl$_3$) δ 204.1, 135.5, 135.4, 132.4, 131.8, 129.6, 128.3, 128.0, 127.8, 126.6, 125.2, 85.0, 68.7, 46.5, 45.9, 44.2, 44.1, 37.6, 33.6, 28.6, 18.7, 18.6, 13.9.

ATR-IR ν (cm-1): 2945 (s), 2866 (s), 1652 (s), 1464 (m), 1311 (m), 1096 (m), 1037 (m), 882 (m), 673 (s).

HRMS (ESI): Calcd for $C_{29}H_{41}ClO_2SiNa^+$ (M+Na$^+$): 507.2462, Found: 507.2454.

(2-(3-((tert-Butyldimethylsilyl)oxy)propyl)-2-((triisopropylsilyl)oxy)bicyclo[2.1.1]hexan-1-yl)(naphthalen-2-yl)methanone (3z)

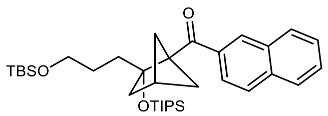

The title compound was prepared according to the General Procedure F with silyl enol ether **1z** and BCB **2a** in toluene within 3 h. Purification by flash column chromatography (0-10% ethyl acetate in hexane) provided the cycloaddition product **3z** (82 mg, 71% yield) with isomers from regioisomeric silyl enol ethers of 12:1:1 ratio as a yellow oil.

^{1}H NMR (500 MHz, CDCl$_3$) δ 8.24 (s, 1H), 7.79 (dd, *J* = 8.6, 1.7 Hz, 1H), 7.65 (d, *J* = 8.1 Hz, 1H), 7.58 (dd, *J* = 10.4, 8.3 Hz, 2H), 7.31 (t, *J* = 7.4 Hz, 1H), 7.26 (t, *J* = 7.5 Hz, 1H), 3.29 (t, *J* = 6.2 Hz, 2H), 2.46 (dd, *J* = 9.0, 6.4 Hz, 1H), 2.15 (d, *J* = 3.0 Hz, 1H), 1.94 (dt, *J* = 6.0, 2.7 Hz, 1H), 1.89 – 1.78 (m, 2H), 1.76 – 1.67 (m, 4H), 1.49 – 1.40 (m, 1H), 1.34 – 1.22 (m, 1H), 0.89 – 0.77 (m, 3H), 0.75 – 0.70 (m, 18H), 0.62 (s, 9H).

^{13}C NMR (125 MHz, CDCl$_3$) δ 203.9, 135.8, 135.4, 132.5, 131.6, 129.6, 128.1, 127.8, 127.8, 126.4, 125.4, 85.4, 68.9, 63.6, 46.2, 44.2, 44.1, 36.5, 33.6, 29.0, 26.1, 18.7, 18.4, 13.9, -5.2.

ATR-IR ν (cm-1): 2945 (m), 2866 (m), 1656 (w), 1618 (w), 1463 (m), 1255 (m), 1103 (s), 1016 (m), 882 (m), 835 (s), 774 (m), 679 (m).

HRMS (ESI): Calcd for $C_{35}H_{57}O_3Si_2^+$ (M+H$^+$): 581.3846, Found: 581.3849.

(2-(4-Methylpent-3-en-1-yl)-2-((triisopropylsilyl)oxy)bicyclo[2.1.1]hexan-1-yl)(naphthalen-2-yl)methanone (3aa)

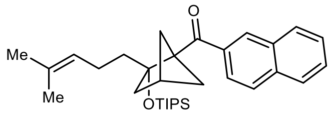

The title compound was prepared according to the General Procedure F with silyl enol ether **1aa** and BCB **2a** in toluene within 3 h. Purification by flash column chromatography (0-10% ethyl acetate in hexane) provided the cycloaddition product **3aa** (82 mg, 84% yield,) with isomers from regioisomeric silyl enol ether of 16:1:1 ratio as a yellow oil.

^{1}H NMR (500 MHz, CDCl$_3$) δ 8.49 (s, 1H), 8.05 (dd, *J* = 8.6, 1.7 Hz, 1H), 7.93 – 7.88 (m, 1H), 7.86 – 7.79 (m, 2H), 7.53 (dddd, *J* = 24.9, 8.2, 7.0, 1.3 Hz, 2H), 5.03 – 4.95 (m, 1H), 2.71 (dd, *J* = 9.5, 6.4 Hz, 1H), 2.41 (t, *J* = 1.6 Hz, 1H), 2.21 (dd, *J* = 6.2, 3.0 Hz, 1H), 2.13 – 2.02 (m, 3H), 2.01 – 1.88 (m, 5H), 1.64 (s, 3H), 1.54 (s, 3H), 1.13 – 1.01 (m, 3H), 0.99 – 0.90 (m, 18H).

^{13}C NMR (125 MHz, CDCl$_3$) δ 204.0, 135.8, 135.4, 132.5, 131.7, 131.7, 129.6, 128.1, 127.9, 127.8, 126.4, 125.4, 124.4, 85.4, 68.8, 46.8, 44.2, 44.0, 39.9, 33.6, 25.8, 24.3, 18.6, 18.6, 17.9, 13.9.

ATR-IR ν (cm-1): 2942 (s), 2865 (s), 1651 (s), 1464 (s), 1309 (s), 1121 (s), 1031 (s), 883 (s).

HRMS (ESI): Calcd for $C_{32}H_{46}O_2SiNa^+$ (M+Na$^+$): 513.3165, Found: 513.3163.

(2-(2-(Benzo[d][1,3]dioxol-5-yl)ethyl)-2-

((triisopropylsilyl)oxy)bicyclo[2.1.1]hexan-1-yl)(naphthalen-2-yl)methanone (3ab)

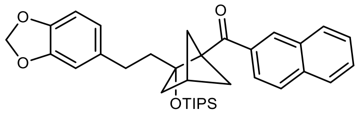

The title compound was prepared according to the General Procedure F with silyl enol ether **1ab** and BCB **2a** in toluene within 6 h. Purification by flash column chromatography (3%-10% ethyl acetate in hexane) provided the cycloaddition product **3ab** (95 mg, 85% yield) as a white solid.

^{1}H NMR (600 MHz, CDCl$_3$) δ 8.55 – 8.51 (m, 1H), 8.08 (dd, J = 8.6, 1.7 Hz, 1H), 7.91 (dd, J = 8.3, 1.2 Hz, 1H), 7.87 – 7.82 (m, 2H), 7.57 (ddd, J = 8.1, 6.8, 1.3 Hz, 1H), 7.52 (ddd, J = 8.1, 6.8, 1.3 Hz, 1H), 6.68 (d, J = 7.9 Hz, 1H), 6.65 (d, J = 1.7 Hz, 1H), 6.56 (dd, J = 7.9, 1.7 Hz, 1H), 5.89 (s, 2H), 2.73 (dd, J = 9.6, 6.5 Hz, 1H), 2.62 (td, J = 13.0, 5.4 Hz, 1H), 2.50 (td, J = 13.3, 4.0 Hz, 1H), 2.45 – 2.41 (m, 1H), 2.29 – 2.22 (m, 2H), 2.21 – 2.12 (m, 2H), 2.09 – 2.00 (m, 2H), 1.96 (dd, J = 9.6, 7.7 Hz, 1H), 1.14 – 1.07 (m, 3H), 0.99 (dd, J = 9.6, 7.4 Hz, 18H).

^{13}C NMR (150 MHz, CDCl$_3$) δ 204.2, 147.6, 145.5, 136.6, 135.7, 135.4, 132.5, 131.8, 129.6, 128.2, 128.0, 127.8, 126.5, 125.4, 121.1, 109.0, 108.3, 100.8, 85.1, 68.8, 47.1, 44.3, 44.0, 42.3, 33.5, 31.5, 18.7, 18.7, 14.0.

ATR-IR ν (cm-1): 2945 (s), 2866 (s), 1651 (m), 1489 (s), 1309 (m), 1244 (s), 1093 (m), 1042 (s), 673 (m).

HRMS (ESI): Calcd for $C_{35}H_{45}O_4Si^+$ (M+H$^+$): 557.3087, Found: 557.3089.

(2-Cyclopropyl-2-((triisopropylsilyl)oxy)bicyclo[2.1.1]hexan-1-yl)(naphthalen-2-yl)methanone (3ac)

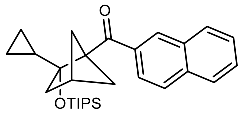

The title compound was prepared according to the General Procedure F with silyl enol ether **1ac** and BCB **2a** in toluene within 4 h. Purification by flash column chromatography (0-10% ethyl acetate in hexane) provided the cycloaddition product **3ac** (61 mg, 68% yield) as a colorless oil.

^{1}H NMR (600 MHz, CDCl$_3$) δ 8.56 – 8.52 (m, 1H), 8.06 (dd, *J* = 8.6, 1.7 Hz, 1H), 7.91 (dd, *J* = 8.1, 1.3 Hz, 1H), 7.84 (dd, *J* = 9.9, 8.2 Hz, 2H), 7.57 (ddd, *J* = 8.2, 6.8, 1.3 Hz, 1H), 7.52 (ddd, *J* = 8.1, 6.8, 1.3 Hz, 1H), 2.64 (dd, *J* = 9.7, 6.5 Hz, 1H), 2.43 – 2.35 (m, 1H), 2.28 (dd, *J* = 9.8, 7.2 Hz, 1H), 2.11 (ddt, *J* = 6.3, 3.0, 1.4 Hz, 1H), 1.98 (ddt, *J* = 7.1, 3.3, 1.8 Hz, 1H), 1.66 (q, *J* = 1.6 Hz, 2H), 1.08 – 1.01 (m, 3H), 0.98 (d, *J* = 7.2 Hz, 9H), 0.94 (d, *J* = 7.3 Hz, 9H), 0.90 – 0.82 (m, 1H), 0.76 – 0.68 (m, 2H), 0.54 – 0.48 (m, 1H), 0.48 – 0.42 (m, 1H).

^{13}C NMR (150 MHz, CDCl$_3$) δ 202.3, 136.0, 135.4, 132.5, 131.6, 129.6, 128.2, 127.9, 127.9, 126.5, 125.2, 85.7, 70.4, 44.3, 43.7, 39.1, 33.9, 18.9, 18.7, 18.7, 14.0, 6.1, 2.2.

ATR-IR ν (cm-1): 2967 (s), 2865 (s), 1655 (s), 1464 (m), 1314 (s), 1138 (s), 1060 (s), 762 (s).

HRMS (ESI): Calcd for $C_{29}H_{41}O_2Si^+$ (M+H$^+$): 449.2876, Found: 449.2865.

(Rac-)Naphthalen-2-yl((3aR,6aS)-6a-((triisopropylsilyl)oxy)hexahydro-1,3-methanopentalen-1(2H)-yl)methanone (3ad)

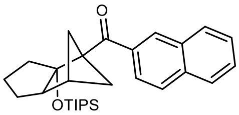

The title compound was prepared according to the General Procedure F with silyl enol ether **1ad** and BCB **2a** in toluene within 2 h. Purification by flash column chromatography (0-10% ethyl acetate in hexane) provided the cycloaddition product **3ad** (70 mg, 78% yield) as a colorless oil.

^{1}H NMR (500 MHz, CDCl$_3$) δ 8.47 (d, J = 1.6 Hz, 1H), 8.01 (dd, J = 8.6, 1.7 Hz, 1H), 7.90 (d, J = 8.1 Hz, 1H), 7.84 (t, J = 8.7 Hz, 2H), 7.59 – 7.49 (m, 2H), 2.74 (dd, J = 9.7, 6.4 Hz, 1H), 2.55 (dt, J = 8.5, 4.1 Hz, 1H), 2.43 – 2.32 (m, 2H), 2.28 (q, J = 2.7 Hz, 1H), 2.19 – 2.01 (m, 3H), 1.97 – 1.87 (m, 2H), 1.79 – 1.69 (m, 1H), 1.43 – 1.33 (m, 1H), 1.01 – 0.85 (m, 21H).

^{13}C NMR (125 MHz, CDCl$_3$) δ 203.4, 135.9, 135.3, 132.5, 131.5, 129.6, 128.2, 127.8, 127.8, 126.5, 125.1, 97.2, 67.0, 58.0, 46.6, 41.1, 38.5, 35.6, 29.1, 29.0, 18.6, 18.5, 13.7.

ATR-IR ν (cm-1): 2945 (s), 2866 (s), 1652 (s), 1465 (m), 1316 (m), 1192 (m), 1089 (m), 882 (m), 677 (m).

HRMS (ESI): Calcd for $C_{29}H_{41}O_2Si^+$ (M+H$^+$): 449.2876, Found: 449.2874.

(Rac-)Naphthalen-2-yl((3aR,7aS)-7a-((triisopropylsilyl)oxy)octahydro-1H-1,3-methanoinden-1-yl)methanone (3ae)

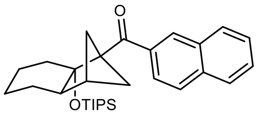

The title compound was prepared according to the General Procedure F with silyl enol ether **1ae** and BCB **2a** in toluene within 2 h. Purification by flash column chromatography (0-10% ethyl acetate in hexane) provided the cycloaddition product **3ae** (69 mg, 75% yield) as a white solid.

m.p.: 75 °C.

^{1}H NMR (500 MHz, CDCl$_3$) δ 8.44 (d, *J* = 1.6 Hz, 1H), 8.00 (dd, *J* = 8.7, 1.7 Hz, 1H), 7.90 (d, *J* = 8.0 Hz, 1H), 7.84 (dd, *J* = 12.1, 8.4 Hz, 2H), 7.57 (ddd, *J* = 8.3, 6.8, 1.4 Hz, 1H), 7.52 (ddd, *J* = 8.1, 6.8, 1.3 Hz, 1H), 2.58 (dd, *J* = 9.7, 6.4 Hz, 1H), 2.25 – 2.07 (m, 5H), 1.98 (dt, *J* = 7.6, 2.8 Hz, 1H), 1.93 – 1.83 (m, 1H), 1.69 – 1.53 (m, 5H), 1.41 – 1.28 (m, 1H), 1.05 – 0.95 (m, 12H), 0.93 – 0.87 (m, 9H).

^{13}C NMR (125 MHz, CDCl$_3$) δ 202.7, 136.0, 135.3, 132.5, 131.5, 129.6, 128.3, 127.9, 127.8, 126.5, 125.2, 83.9, 68.8, 50.5, 42.8, 42.0, 39.5, 31.4, 25.8, 20.1, 19.1, 18.7, 18.7, 14.3.

ATR-IR ν (cm-1): 2941 (s), 2865 (s), 1652 (s), 1464 (m), 1306 (m), 1063 (s), 883 (m), 676 (m).

HRMS (ESI): Calcd for $C_{30}H_{43}O_2Si^+$ (M+H$^+$): 463.3032, Found: 463.3025.

(Rac-)Naphthalen-2-yl((3aR,8aS)-8a-((triisopropylsilyl)oxy)octahydro-1,3-

methanoazulen-1(2H)-yl)methanone (3af)

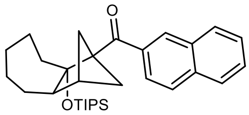

The title compound was prepared according to the General Procedure F with silyl enol ether **1af** and BCB **2a** in toluene within 2 h. Purification by flash column chromatography (0-10% ethyl acetate in hexane) provided the cycloaddition product **3af** (75 mg, 79% yield) as a white solid.

m.p.: 140 ℃.

^{1}H NMR (500 MHz, CDCl$_3$) δ 8.38 (d, J = 1.7 Hz, 1H), 8.00 – 7.90 (m, 2H), 7.85 (t, J = 7.8 Hz, 2H), 7.62 – 7.50 (m, 2H), 2.61 (dd, J = 9.7, 6.6 Hz, 1H), 2.36 – 2.25 (m, 2H), 2.21 – 2.16 (m, 1H), 2.14 (td, J = 3.1, 1.3 Hz, 1H), 2.08 (dd, J = 6.6, 3.1 Hz, 1H), 2.00 – 1.77 (m, 5H), 1.66 – 1.55 (m, 2H), 1.53 – 1.43 (m, 1H), 1.43 – 1.33 (m, 1H), 1.32 – 1.21 (m, 1H), 1.17 – 1.07 (m, 3H), 1.05 (d, J = 7.1 Hz, 9H), 0.99 (d, J = 7.2 Hz, 9H).

^{13}C NMR (125 MHz, CDCl$_3$) δ 201.6, 136.3, 135.3, 132.5, 131.2, 129.7, 128.3, 128.1, 127.8, 126.6, 125.2, 89.6, 69.5, 57.8, 43.2, 42.7, 40.4, 37.1, 33.5, 32.1, 28.8, 25.6, 18.9, 18.9, 14.8.

ATR-IR ν (cm-1): 2922 (s), 2863 (s), 1654 (s), 1463 (m), 1307 (m), 1136 (s), 882 (m), 673 (s).

HRMS (ESI): Calcd for $C_{31}H_{44}O_2SiNa^+$ (M+Na$^+$): 499.3008, Found: 499.2991.

(Rac-)Naphthalen-2-yl((3aR,8aR)-3a-((triisopropylsilyl)oxy)-1,3a,8,8a-tetrahydro-1,3-methanocyclopenta[a]inden-3(2H)-yl)methanone (3ag)

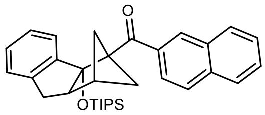

The title compound was prepared according to the General Procedure F with silyl enol ether **1ag** and BCB **2a** in toluene within 2 h. Purification by flash column chromatography (0-10% ethyl acetate in hexane) provided the cycloaddition product **3ag** (89 mg, 93% yield) as a colorless oil.

^{1}H NMR (600 MHz, CDCl$_3$) δ 8.47 (d, J = 1.7 Hz, 1H), 8.11 (dd, J = 8.6, 1.7 Hz, 1H), 7.89 – 7.82 (m, 3H), 7.61 – 7.55 (m, 1H), 7.55 – 7.49 (m, 1H), 7.28 – 7.21 (m, 1H), 7.17 (d, J = 7.6 Hz, 1H), 7.07 (t, J = 7.4 Hz, 1H), 6.97 (d, J = 7.6 Hz, 1H), 3.34 (dd, J = 17.1, 9.2 Hz, 1H), 2.93 (dd, J = 9.0, 2.7 Hz, 1H), 2.85 (dd, J = 9.6, 6.5 Hz, 1H), 2.62 (dd, J = 17.1, 3.1 Hz, 1H), 2.51 (q, J = 2.7 Hz, 1H), 2.40 (dd, J = 6.5, 3.0 Hz, 1H), 1.83 (dt, J = 7.9, 2.8 Hz, 1H), 1.50 (dd, J = 9.6, 7.9 Hz, 1H), 0.82 (t, J = 2.3 Hz, 12H), 0.71 (dd, J = 5.3, 2.3 Hz, 9H).

^{13}C NMR (150 MHz, CDCl$_3$) δ 201.6, 145.2, 142.5, 135.4, 135.3, 132.3, 131.9, 129.5, 128.8, 128.0, 127.7, 127.6, 126.3, 126.2, 125.9, 125.3, 124.4, 96.8, 67.8, 53.7, 44.3, 39.9, 39.3, 34.6, 18.1, 17.8, 13.0.

ATR-IR ν (cm-1): 2944 (s), 2865 (s), 1655 (s), 1460 (m), 1124 (s), 883 (m), 824 (m), 676 (m).

HRMS (ESI): Calcd for $C_{33}H_{41}O_2Si^+$ (M+H$^+$): 497.2876, Found: 497.2878.

(Rac-)Naphthalen-2-yl((3aR,9bR)-9b-((triisopropylsilyl)oxy)-2,3,3a,4,5,9b-hexahydro-1H-1,3-methanocyclopenta[a]naphthalen-1-yl)methanone (3ah)

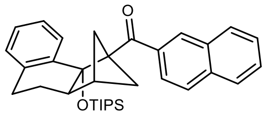

The title compound was prepared according to the General Procedure F with silyl enol ether **1ah** and BCB **2a** in toluene within 4 h. Purification by flash column chromatography (0-10% ethyl acetate in hexane) provided the cycloaddition product **3ah** (62 mg, 61% yield) as a colorless oil.

^{1}H NMR (500 MHz, CDCl$_3$) δ 8.16 (d, J = 1.7 Hz, 1H), 7.92 (dd, J = 8.6, 1.7 Hz, 1H), 7.82 (d, J = 8.2 Hz, 1H), 7.77 (dd, J = 10.5, 8.3 Hz, 2H), 7.55 (ddd, J = 8.1, 6.8, 1.3 Hz, 1H), 7.48 (ddd, J = 8.1, 6.8, 1.3 Hz, 1H), 7.16 – 7.07 (m, 3H), 6.91 (td, J = 7.4, 1.9 Hz, 1H), 3.02 – 2.90 (m, 1H), 2.85 – 2.70 (m, 3H), 2.41 (td, J = 3.0, 1.1 Hz, 1H), 2.28 – 2.15 (m, 2H), 2.13 – 2.04 (m, 1H), 1.88 (dd, J = 9.9, 8.1 Hz, 1H), 1.75 (dt, J = 8.1, 2.7 Hz, 1H), 1.01 – 0.92 (m, 3H), 0.89 (d, J = 7.3 Hz, 9H), 0.76 (d, J = 7.3 Hz, 9H).

^{13}C NMR (125 MHz, CDCl$_3$) δ 201.5, 140.0, 137.4, 135.6, 135.3, 132.4, 132.0, 130.0, 129.8, 128.5, 128.1, 127.7, 127.7, 127.3, 126.3, 125.6, 125.4, 82.8, 70.9, 50.1, 44.9, 40.8, 40.4, 28.0, 24.2, 18.5, 18.0, 13.6.

ATR-IR ν (cm-1): 2941 (s), 2865 (s), 1655 (s), 1464 (m), 1311 (m), 1100 (m), 881 (m), 795 (s), 674 (m).

HRMS (ESI): Calcd for $C_{34}H_{43}O_2Si^+$ (M+H$^+$): 511.3027, Found: 511.3032.

(Rac-)Naphthalen-2-yl((3aS,9bR)-9b-((triisopropylsilyl)oxy)-2,3,3a,9b-tetrahydro-1,3-methanocyclopenta[c]chromen-1(4H)-yl)methanone (3ai)

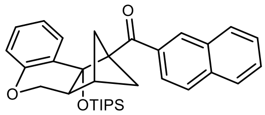

The title compound was prepared according to the General Procedure F with silyl enol ether **1ai** and BCB **2a** in toluene within 3 h. Purification by flash column chromatography (3-10% ethyl acetate in hexane) provided the cycloaddition product **3ai** (32 mg, 31% yield) as a white solid.

m.p.: 125 ℃.

^{1}H NMR (600 MHz, CDCl$_3$) δ 7.87 (s, 1H), 7.84 (dd, J = 8.6, 1.7 Hz, 1H), 7.80 (d, J = 8.2 Hz, 1H), 7.75 (d, J = 8.6 Hz, 1H), 7.66 (d, J = 8.2 Hz, 1H), 7.54 (dd, J = 8.1, 6.8 Hz, 1H), 7.46 (t, J = 7.5 Hz, 1H), 7.10 (td, J = 7.7, 1.6 Hz, 1H), 7.04 (dd, J = 7.8, 1.7 Hz, 1H), 6.94 (d, J = 8.1 Hz, 1H), 6.63 – 6.57 (m, 1H), 4.39 (d, J = 11.6 Hz, 1H), 4.31 (dd, J = 11.6, 4.6 Hz, 1H), 2.76 – 2.67 (m, 2H), 2.56 – 2.50 (m, 1H), 2.27 – 2.18 (m, 2H), 1.88 – 1.81 (m, 1H), 1.07 – 0.98 (m, 3H), 0.95 (d, J = 7.3 Hz, 9H), 0.84 (d, J = 7.3 Hz, 9H).

^{13}C NMR (150 MHz, CDCl$_3$) δ 200.4, 154.8, 135.3, 135.3, 132.3, 131.8, 129.8, 129.7, 129.0, 128.4, 128.2, 127.8, 127.7, 126.3, 125.1, 121.2, 117.3, 79.5, 70.4, 67.5, 52.9, 44.5, 40.5, 39.3, 18.5, 18.1, 13.6.

ATR-IR ν (cm-1): 2944 (m), 2865 (m), 1655 (s), 1485 (m), 1239 (m), 1098 (m), 881 (m), 757 (s), 675 (m).
HRMS (ESI): Calcd for $C_{33}H_{41}O_3Si^+$ ($M+H^+$): 513.2825, Found: 513.2823.

(Rac-)Naphthalen-2-yl((5aR,8aR)-8a-((triisopropylsilyl)oxy)-4,5,5a,6,7,8a-hexahydro-8H-6,8-methanoindeno[5,4-b]furan-8-yl)methanone (3aj)

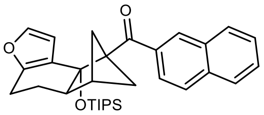

The title compound was prepared according to the General Procedure F with silyl enol ether **1aj** and BCB **2a** in toluene within 2 h. Purification by flash column chromatography (0-10% ethyl acetate in hexane) provided the cycloaddition product **3aj** (90 mg, 90% yield) as a colorless oil.
^{1}H NMR (500 MHz, CDCl$_3$) δ 8.45 (d, J = 1.6 Hz, 1H), 8.06 (dd, J = 8.6, 1.7 Hz, 1H), 7.90 – 7.79 (m, 3H), 7.57 (ddd, J = 8.1, 6.8, 1.3 Hz, 1H), 7.51 (ddd, J = 8.1, 6.8, 1.3 Hz, 1H), 7.13 (d, J = 1.9 Hz, 1H), 6.00 (d, J = 1.9 Hz, 1H), 2.78 – 2.70 (m, 2H), 2.69 – 2.60 (m, 1H), 2.61 – 2.55 (m, 1H), 2.35 – 2.31 (m, 1H), 2.31 – 2.22 (m, 2H), 2.06 – 1.97 (m, 1H), 1.84 – 1.79 (m, 1H), 1.74 (dd, J = 9.7, 7.8 Hz, 1H), 0.98 – 0.91 (m, 3H), 0.89 (d, J = 6.0 Hz, 9H), 0.77 (d, J = 6.9 Hz, 9H).
^{13}C NMR (125 MHz, CDCl$_3$) δ 201.8, 152.3, 139.8, 135.4, 135.4, 132.5, 132.1, 129.7, 128.2, 127.8, 127.8, 126.5, 125.5, 120.9, 110.2, 81.5, 69.6, 51.0, 43.7, 41.8, 40.0, 24.8, 20.9, 18.4, 18.0, 13.3.
ATR-IR ν (cm-1): 2942 (m), 2865 (s), 1655 (s), 1464 (m), 1310 (m), 1087 (m), 1066 (s), 882 (m), 779 (m), 676 (m).
HRMS (ESI): Calcd for $C_{32}H_{41}O_3Si^+$ ($M+H^+$): 501.2825, Found: 501.2835.

(Rac-)Naphthalen-2-yl((5aR,8aS)-8a-((triisopropylsilyl)oxy)-4,5,5a,6,7,8a-hexahydro-8H-6,8-methanoindeno[5,4-b]thiophen-8-yl)methanone (3ak)

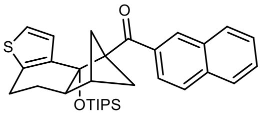

The title compound was prepared according to the General Procedure F with silyl enol ether **1ak** and BCB **2a** in toluene within 2 h. Purification by flash column chromatography (0-10% ethyl acetate in hexane) provided the cycloaddition product **3ak** (52 mg, 50% yield) as a yellow oil.
^{1}H NMR (500 MHz, CDCl$_3$) δ 8.21 – 8.17 (m, 1H), 7.97 (dd, J = 8.6, 1.7 Hz, 1H), 7.83 (d, J = 7.4 Hz, 1H), 7.79 (d, J = 8.4 Hz, 2H), 7.56 (ddd, J = 8.1, 6.8, 1.3 Hz, 1H), 7.49 (ddd, J = 8.1, 6.8, 1.3 Hz, 1H), 6.79 (dd, J = 5.3, 0.8 Hz, 1H), 6.59 (d, J = 5.3 Hz, 1H), 2.93 (ddd, J = 16.4, 6.5, 2.8 Hz, 1H), 2.82 (ddd, J = 16.7, 11.7, 6.2 Hz, 1H), 2.71 (dd, J = 9.3, 6.6 Hz, 1H), 2.67 – 2.58 (m, 1H), 2.38 (td, J = 2.9, 1.2 Hz, 1H), 2.27 – 2.16 (m, 2H), 2.16 – 2.08 (m, 1H), 1.88 – 1.78 (m, 2H), 0.99 – 0.91 (m, 3H), 0.90 (d, J = 6.6 Hz, 9H), 0.77 (d, J = 7.1 Hz, 9H).
^{13}C NMR (125 MHz, CDCl$_3$) δ 201.4, 139.4, 138.0, 135.4, 135.4, 132.4, 132.1, 129.8, 128.3, 128.2, 127.8, 127.8, 126.4, 125.4, 120.6, 81.9, 70.4, 50.1, 44.0, 41.4, 40.1, 25.4,

23.0, 18.4, 17.9, 13.5.
ATR-IR ν (cm-1): 2941 (m), 2864 (s), 1654 (s), 1463 (m), 1308 (m), 1125 (m), 882 (m), 782 (s), 674 (m).
HRMS (ESI): Calcd for $C_{32}H_{40}O_2SSiNa^+$ ($M+Na^+$): 539.2416, Found: 539.2402.

(Rac-)Naphthalen-2-yl((3aR,7aS)-7a-((triisopropylsilyl)oxy)-2,3,3a,4,5,7a-hexahydro-1H-1,3-methanoinden-1-yl)methanone (3al)

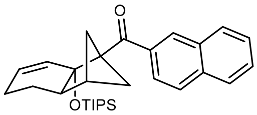

The title compound was prepared according to the General Procedure F with silyl enol ether **1al** and BCB **2a** in toluene within 2 h. Purification by flash column chromatography (0-10% ethyl acetate in hexane) provided the cycloaddition product **3al** (85 mg, 90% yield) as a colorless oil.
^{1}H NMR (600 MHz, CDCl$_3$) δ 8.53 (d, J = 1.8 Hz, 1H), 8.05 (dt, J = 8.6, 1.4 Hz, 1H), 7.90 (d, J = 8.2 Hz, 1H), 7.84 (dd, J = 15.9, 8.4 Hz, 2H), 7.61 – 7.54 (m, 1H), 7.55 – 7.49 (m, 1H), 6.08 (dt, J = 10.3, 4.1 Hz, 1H), 5.89 (dt, J = 10.5, 2.1 Hz, 1H), 2.62 (dd, J = 9.7, 6.5 Hz, 1H), 2.51 – 2.45 (m, 1H), 2.27 – 2.22 (m, 1H), 2.17 (dd, J = 6.5, 3.0 Hz, 1H), 2.16 – 2.08 (m, 2H), 2.08 – 1.99 (m, 1H), 1.85 – 1.79 (m, 1H), 1.79 – 1.72 (m, 1H), 1.23 – 1.15 (m, 1H), 1.00 – 0.86 (m, 21H).
^{13}C NMR (150 MHz, CDCl$_3$) δ 202.0, 135.3, 135.2, 132.3, 131.9, 131.9, 130.8, 129.5, 128.1, 127.7, 127.6, 126.3, 125.1, 79.4, 69.1, 49.5, 43.2, 41.9, 39.7, 23.8, 22.3, 18.4, 18.3, 13.3.
ATR-IR ν (cm-1): 2941 (s), 2864 (s), 1653 (s), 1464 (m), 1308 (m), 1139 (s), 1052 (s), 882 (m), 677 (m).
HRMS (ESI): Calcd for $C_{30}H_{41}O_2Si^+$ ($M+H^+$): 461.2876, Found: 461.2869.

(Rac-)((3aR,7aS)-5,5-Dimethyl-7a-((triisopropylsilyl)oxy)-2,3,3a,4,5,7a-hexahydro-1H-1,3-methanoinden-1-yl)(naphthalen-2-yl)methanone (3am)

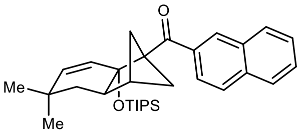

The title compound was prepared according to the General Procedure F with silyl enol ether **1am** and BCB **2a** in toluene within 2 h. Purification by flash column chromatography (0-10% ethyl acetate in hexane) provided the cycloaddition product **3am** (77 mg, 78% yield) as a colorless solid.
^{1}H NMR (500 MHz, CDCl$_3$) δ 8.47 (d, J = 1.6 Hz, 1H), 8.02 (dd, J = 8.7, 1.7 Hz, 1H), 7.91 (d, J = 8.1 Hz, 1H), 7.84 (dd, J = 11.9, 8.4 Hz, 2H), 7.62 – 7.54 (m, 1H), 7.56 – 7.49 (m, 1H), 5.79 – 5.71 (m, 2H), 2.63 (dd, J = 9.8, 6.1 Hz, 1H), 2.56 – 2.48 (m, 1H), 2.24 – 2.17 (m, 2H), 2.11 (dd, J = 9.8, 7.4 Hz, 1H), 1.98 (dd, J = 14.3, 8.3 Hz, 1H), 1.93 – 1.88 (m, 1H), 1.52 (dd, J = 14.3, 6.0 Hz, 1H), 1.11 (s, 6H), 1.00 – 0.85 (m, 21H).
^{13}C NMR (125 MHz, CDCl$_3$) δ 201.8, 141.2, 135.6, 135.4, 132.5, 131.8, 129.7, 128.3,

127.9, 127.5, 126.5, 125.3, 80.0, 67.1, 48.6, 43.2, 43.1, 40.0, 39.7, 31.5, 31.1, 30.5, 18.6, 18.6, 13.9.

ATR-IR ν (cm-1): 2947 (m), 2864 (s), 1655 (s), 1463 (m), 1308 (m), 1106 (s), 882 (m), 749 (s), 677 (m).

HRMS (ESI): Calcd for $C_{32}H_{45}O_2Si^+$ ($M+H^+$): 489.3189, Found: 489.3183.

(Rac-)((3aR,8aR)-8a-Allyl-3a-((triisopropylsilyl)oxy)-1,3a,8,8a-tetrahydro-1,3-methanocyclopenta[a]inden-3(2H)-yl)(naphthalen-2-yl)methanone (3an)

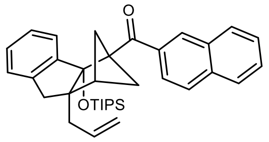

The title compound was prepared according to the General Procedure F with silyl enol ether **1an** and BCB **2a** in toluene within 3 h. Purification by flash column chromatography (0-10% ethyl acetate in hexane) provided the cycloaddition product **3an** (52 mg, 48% yield) as a yellow oil.

^{1}H NMR (500 MHz, CDCl$_3$) δ 8.23 (d, *J* = 1.6 Hz, 1H), 8.02 (dd, *J* = 8.6, 1.7 Hz, 1H), 7.82 (dd, *J* = 16.1, 8.4 Hz, 2H), 7.73 (d, *J* = 8.1 Hz, 1H), 7.56 (ddd, *J* = 8.2, 6.7, 1.3 Hz, 1H), 7.49 (ddd, *J* = 8.1, 6.7, 1.2 Hz, 1H), 7.25 – 7.20 (m, 1H), 7.16 (d, *J* = 7.5 Hz, 1H), 7.02 (d, *J* = 7.6 Hz, 1H), 6.97 (t, *J* = 7.4 Hz, 1H), 5.96 (ddt, *J* = 17.2, 10.1, 7.2 Hz, 1H), 5.21 – 5.11 (m, 2H), 3.13 (d, *J* = 17.2 Hz, 1H), 2.83 (dd, *J* = 9.9, 7.2 Hz, 1H), 2.80 – 2.72 (m, 2H), 2.44 (t, *J* = 3.1 Hz, 1H), 2.40 (dd, *J* = 13.8, 7.3 Hz, 1H), 2.18 (dd, *J* = 7.2, 2.9 Hz, 1H), 1.79 (dd, *J* = 7.8, 3.4 Hz, 1H), 1.46 (dd, *J* = 9.7, 8.0 Hz, 1H), 0.96 – 0.83 (m, 12H), 0.74 (d, *J* = 6.4 Hz, 9H).

^{13}C NMR (125 MHz, CDCl$_3$) δ 200.8, 144.3, 142.1, 135.8, 135.3, 135.0, 132.3, 132.2, 129.7, 128.9, 128.2, 127.7, 127.7, 126.9, 126.4, 125.7, 125.3, 124.1, 117.2, 98.0, 67.2, 56.2, 41.9, 41.6, 41.2, 40.0, 39.8, 18.7, 18.3, 14.3.

ATR-IR ν (cm-1): 2944 (s), 2865 (s), 1653 (s), 1627 (m), 1460 (m), 1305 (s), 1222 (s), 1121 (s), 882 (s), 778 (s).

HRMS (ESI): Calcd for $C_{36}H_{45}O_2Si^+$ ($M+H^+$): 537.3189, Found: 537.3195.

(Rac-)((2R,3S)-3-ethyl-2-phenyl-2-((triisopropylsilyl)oxy)bicyclo[2.1.1]hexan-1-yl)(naphthalen-2-yl)methanone (3ao)

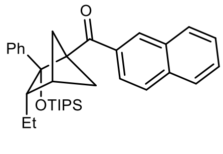

The title compound was prepared according to the General Procedure F with silyl enol ether **1ao** and BCB **2a** in DCM within 6 h. Purification by flash column chromatography (0-10% ethyl acetate in hexane) provided the cycloaddition product **3ao** (58 mg, 57% yield, 13:1 dr) as a white solid.

m.p.: 108 ℃.

^{1}H NMR (500 MHz, CDCl$_3$) δ 8.14 (d, J = 1.7 Hz, 1H), 7.88 – 7.80 (m, 2H), 7.77 (t, J = 8.0 Hz, 2H), 7.55 (ddd, J = 8.2, 6.8, 1.3 Hz, 1H), 7.51 – 7.42 (m, 3H), 7.21 – 7.14

(m, 3H), 2.98 – 2.90 (m, 1H), 2.80 (dd, J = 9.5, 7.1 Hz, 1H), 2.51 – 2.42 (m, 1H), 2.10 – 2.04 (m, 1H), 1.97 – 1.83 (m, 2H), 1.67 – 1.56 (m, 2H), 1.10 (t, J = 7.4 Hz, 3H), 0.91 (d, J = 6.9 Hz, 9H), 0.90 – 0.81 (m, 3H), 0.73 (d, J = 7.1 Hz, 9H).
^{13}C NMR (150 MHz, $CDCl_3$) (one peak on aromatic area is missing) δ 202.1, 143.8, 135.5, 135.0, 132.3, 131.5, 129.6, 128.9, 128.1, 127.6, 127.6, 127.6, 126.3, 125.3, 89.1, 69.0, 51.8, 41.8, 41.8, 36.1, 22.6, 19.0, 18.4, 14.8, 13.2.
ATR-IR ν (cm-1): 2947 (s), 2866 (s), 1655 (m), 1464 (m), 1296 (m), 1116 (s), 884 (m), 782 (m), 672 (m).
HRMS (ESI): Calcd for $C_{34}H_{45}O_2Si^+$ (M+H$^+$): 513.3189, Found: 513.3172.

(Rac-)((2S,3S)-2-ethyl-3-methyl-2-((triisopropylsilyl)oxy)bicyclo[2.1.1]hexan-1-yl)(naphthalen-2-yl)methanone (3ap)

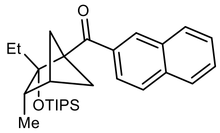

The title compound was prepared according to the General Procedure F with silyl enol ether **1ap** and BCB **2a** in toluene within 3 h. Purification by flash column chromatography (0-10% ethyl acetate in hexane) provided the cycloaddition product **3ap** (80 mg, 89% yield, 1.3:1 dr) as a colorless oil.
^{1}H NMR (500 MHz, $CDCl_3$) δ 8.41 (d, *J* = 1.7 Hz, 1H), 7.99 (dd, *J* = 8.6, 1.7 Hz, 1H), 7.91 (d, *J* = 8.1 Hz, 1H), 7.85 (t, *J* = 8.7 Hz, 2H), 7.61 – 7.56 (m, 1H), 7.56 – 7.50 (m, 1H), 2.64 (dd, *J* = 9.6, 6.9 Hz, 1H), 2.32 (dd, *J* = 7.0 Hz, 1H), 2.17 – 1.99 (m, 5H), 1.73 (dq, *J* = 14.4, 7.3 Hz, 1H), 1.13 (d, *J* = 6.9 Hz, 3H), 1.07 (t, *J* = 7.4 Hz, 3H), 1.05 – 0.93 (m, 21H).
^{13}C NMR (125 MHz, $CDCl_3$) δ 201.9, 136.0, 135.3, 132.5, 131.4, 129.7, 128.4, 128.0, 127.8, 126.6, 125.3, 87.5, 69.3, 45.2, 45.1, 40.5, 39.9, 32.3, 19.0, 19.0, 15.5, 14.7, 10.0.
ATR-IR ν (cm-1): 2943 (s), 2865 (s), 1652 (s), 1462 (m), 1310 (s), 1165 (s), 1060 (s), 881 (s).
HRMS (ESI): Calcd for $C_{29}H_{43}O_2Si^+$ (M+H$^+$): 451.3032, Found: 451.3031.

(Rac-)((2S,3R)-2-ethyl-3-methyl-2-((triisopropylsilyl)oxy)bicyclo[2.1.1]hexan-1-yl)(naphthalen-2-yl)methanone (3ap')

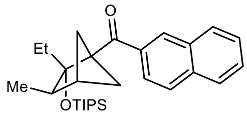

^{1}H NMR (500 MHz, $CDCl_3$) δ 8.50 (s, 1H), 8.05 (dd, *J* = 8.5, 1.5 Hz, 1H), 7.90 (d, *J* = 8.1 Hz, 1H), 7.82 (t, *J* = 9.0 Hz, 2H), 7.58 – 7.53 (m, 1H), 7.50 (t, *J* = 7.0 Hz, 1H), 2.67 (dd, *J* = 9.4, 6.3 Hz, 1H), 2.38 (dd, *J* = 6.5 Hz, 1H), 2.17 – 2.12 (m, 2H), 2.07 – 2.00 (m, 1H), 1.97 – 1.87 (m, 2H), 1.75 (dq, *J* = 15.0, 7.6 Hz, 1H), 1.12 (d, *J* = 7.2 Hz, 3H), 1.11 – 1.02 (m, 3H), 0.95 (dd, *J* = 14.2, 7.3 Hz, 18H), 0.78 (t, *J* = 7.3 Hz, 3H).
^{13}C NMR (125 MHz, $CDCl_3$) δ 204.4, 135.8, 135.4, 132.5, 131.7, 129.6, 128.1, 127.9,

127.8, 126.4, 125.6, 87.0, 68.9, 50.6, 42.9, 40.6, 39.5, 27.3, 18.8, 18.5, 15.1, 13.8, 9.4.
ATR-IR ν (cm-1): 2945 (s), 2866 (s), 1653 (s), 1464 (s), 1301 (s), 1045 (s), 780 (s).
HRMS (ESI): Calcd for $C_{29}H_{42}O_2SiNa^+$ ($M+Na^+$): 473.2852, Found: 473.2847.

(3,3-Dimethyl-2-phenyl-2-((triisopropylsilyl)oxy)bicyclo[2.1.1]hexan-1-yl)(naphthalen-2-yl)methanone (3aq)

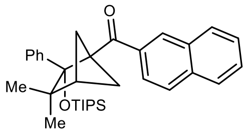

The title compound was prepared according to the General Procedure F with silyl enol ether **1aq** and BCB **2a** in DCM within 6 h. Purification by flash column chromatography (0-10% ethyl acetate in hexane) provided the cycloaddition product **3aq** (44 mg, 43% yield) as a colorless solid.
^{1}H NMR (500 MHz, $CDCl_3$) δ 8.38 (s, 1H), 7.98 – 7.93 (m, 1H), 7.92 – 7.86 (m, 3H), 7.63 – 7.53 (m, 2H), 7.50 – 7.44 (m, 2H), 7.24 – 7.13 (m, 3H), 2.81 (dd, J = 9.9, 7.0 Hz, 1H), 2.39 (dd, J = 7.6, 3.2 Hz, 1H), 2.28 – 2.20 (m, 2H), 2.02 (t, J = 3.2 Hz, 1H), 1.42 (s, 3H), 1.10 – 1.00 (m, 3H), 0.93 (d, J = 7.4 Hz, 9H), 0.86 (d, J = 7.4 Hz, 9H), 0.69 (s, 3H).
^{13}C NMR (150 MHz, $CDCl_3$) (one peak on aromatic area is missing) δ 202.1, 143.2, 137.6, 134.5, 132.4, 129.2, 129.2, 128.6, 128.1, 127.9, 127.7, 127.1, 126.7, 124.9, 89.8, 66.3, 49.7, 47.1, 42.1, 40.4, 28.1, 24.8, 19.1, 18.9, 14.6.
ATR-IR ν (cm-1): 2945 (m), 2864 (m), 1669 (m), 1464 (m), 1295 (m), 1103 (s), 882 (m), 810 (m), 665 (m).
HRMS (ESI): Calcd for $C_{35}H_{45}O_2Si^+$ ($M+H^+$): 513.3189, Found: 513.3186.

Phenyl(2-phenyl-2-((triisopropylsilyl)oxy)bicyclo[2.1.1]hexan-1-yl)methanone (3ba)

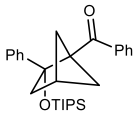

The title compound was prepared according to the General Procedure F with silyl enol ether **1a** and BCB **2b** in DCM within 2 h. Purification by flash column chromatography (0-10% ethyl acetate in hexane) provided the cycloaddition product **3ba** (61 mg, 70% yield) as a white solid.
m.p.: 78 °C.
^{1}H NMR (600 MHz, $CDCl_3$) δ 7.87 – 7.81 (m, 2H), 7.48 – 7.42 (m, 1H), 7.35 – 7.29 (m, 4H), 7.21 – 7.16 (m, 3H), 3.06 (ddd, J = 11.8, 2.7, 1.6 Hz, 1H), 2.74 (ddd, J = 8.3, 6.7, 1.6 Hz, 1H), 2.53 (tt, J = 3.0, 1.5 Hz, 1H), 2.27 – 2.19 (m, 1H), 2.16 (ddd, J = 12.0, 3.2, 1.4 Hz, 1H), 1.83 – 1.72 (m, 2H), 1.01 – 0.87 (m, 12H), 0.83 (d, J = 6.9 Hz, 9H).
^{13}C NMR (150 MHz, $CDCl_3$) (one peak on aromatic area is missing) δ 201.9, 143.2, 138.2, 132.3, 129.8, 128.0, 127.5, 127.4, 86.3, 70.0, 44.5, 42.9, 42.4, 33.7, 18.5, 18.3, 13.4.

ATR-IR ν (cm-1): 2945 (m), 2866 (m), 1662 (s), 1447 (m), 1301 (m), 1136 (m), 1055 (m), 701 (s).
HRMS (ESI): Calcd for $C_{28}H_{38}O_2SiNa^+$ (M+Na$^+$): 457.2539, Found: 457.2524.

(Rac-)Phenyl((3aR,8aR)-3a-((triisopropylsilyl)oxy)-1,3a,8,8a-tetrahydro-1,3-methanocyclopenta[a]inden-3(2H)-yl)methanone (3bb)

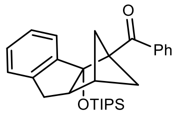

The title compound was prepared according to the General Procedure F with silyl enol ether **1ag** and BCB **2b** in DCM within 2 h. Purification by flash column chromatography (0-10% ethyl acetate in hexane) provided the cycloaddition product **3bb** (58 mg, 65% yield) as a clolorless oil.
^{1}H NMR (500 MHz, CDCl$_3$) δ 8.02 (dd, J = 8.3, 1.4 Hz, 2H), 7.55 – 7.48 (m, 1H), 7.39 (t, J = 7.8 Hz, 2H), 7.22 (td, J = 7.4, 1.2 Hz, 1H), 7.14 (dt, J = 7.7, 1.0 Hz, 1H), 7.08 (td, J = 7.4, 1.1 Hz, 1H), 6.95 – 6.89 (m, 1H), 3.31 (dd, J = 17.2, 9.2 Hz, 1H), 2.96 – 2.83 (m, 1H), 2.72 (dd, J = 9.7, 6.7 Hz, 1H), 2.58 (dd, J = 17.1, 3.1 Hz, 1H), 2.47 (td, J = 3.0, 1.8 Hz, 1H), 2.33 (dd, J = 6.7, 3.0 Hz, 1H), 1.85 – 1.71 (m, 1H), 1.42 (dd, J = 9.7, 7.9 Hz, 1H), 0.93 – 0.78 (m, 12H), 0.71 (d, 9H).
^{13}C NMR (125 MHz, CDCl$_3$) δ 201.9, 145.4, 142.7, 138.3, 132.6, 130.0, 128.9, 128.0, 126.4, 125.8, 124.5, 96.8, 67.8, 53.8, 44.3, 39.8, 39.4, 34.7, 18.4, 17.9, 13.1.
ATR-IR ν (cm-1): 2946 (m), 2866 (m), 1660 (s), 1458 (m), 1248 (m), 1114 (m), 881 (m), 711 (m).
HRMS (ESI): Calcd for $C_{29}H_{39}O_2Si^+$ (M+H$^+$): 447.2719, Found: 447.2733.

(Rac-)Benzo[d][1,3]dioxol-5-yl((3aR,8aR)-3a-((triisopropylsilyl)oxy)-1,3a,8,8a-tetrahydro-1,3-methanocyclopenta[a]inden-3(2H)-yl)methanone (3bc)

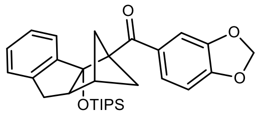

The title compound was prepared according to the General Procedure F with silyl enol ether **1ag** and BCB **2c** in DCM within 3 h. Purification by flash column chromatography (0-10% ethyl acetate in hexane) provided the cycloaddition product **3bc** (52 mg, 53% yield) as a white solid.
m.p.: 88 °C.
^{1}H NMR (600 MHz, CDCl$_3$) δ 7.65 (dd, *J* = 8.2, 1.7 Hz, 1H), 7.53 (d, *J* = 1.6 Hz, 1H), 7.21 (t, *J* = 7.4 Hz, 1H), 7.13 (d, *J* = 7.6 Hz, 1H), 7.09 (t, *J* = 7.4 Hz, 1H), 6.92 (d, *J* = 7.6 Hz, 1H), 6.78 (d, *J* = 8.1 Hz, 1H), 6.04 – 6.00 (m, 2H), 3.30 (dd, *J* = 17.1, 9.2 Hz, 1H), 2.88 – 2.82 (m, 1H), 2.66 (dd, *J* = 9.7, 6.5 Hz, 1H), 2.59 – 2.53 (m, 1H), 2.47 – 2.42 (m, 1H), 2.29 (dd, *J* = 6.6, 3.0 Hz, 1H), 1.72 (dt, *J* = 8.1, 2.9 Hz, 1H), 1.38 (dd, *J* = 9.6, 8.0 Hz, 1H), 0.88 – 0.79 (m, 12H), 0.76 – 0.69 (m, 9H).
^{13}C NMR (150 MHz, CDCl$_3$) δ 199.7, 151.3, 147.7, 145.4, 142.7, 133.1, 129.0, 126.8, 126.4, 125.9, 124.6, 109.7, 107.5, 101.7, 96.7, 67.7, 53.8, 44.5, 39.9, 39.3, 34.7, 18.4, 17.9, 13.2.

ATR-IR ν (cm-1): 2943 (s), 2865 (s), 1653 (m), 1439 (s), 1249 (s), 1115 (m), 1040 (m), 769 (m), 676 (m).
HRMS (ESI): Calcd for $C_{30}H_{39}O_4Si+$ ($M+H^+$): 491.2617, Found: 491.2603.

(Rac-)Furan-2-yl((3aR,8aR)-3a-((triisopropylsilyl)oxy)-1,3a,8,8a-tetrahydro-1,3-methanocyclopenta[a]inden-3(2H)-yl)methanone (3bd)

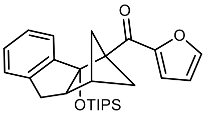

The title compound was prepared according to the General Procedure F with silyl enol ether **1ag** and BCB **2d** in toluene within 3 h. Purification by flash column chromatography (0-10% ethyl acetate in hexane) provided the cycloaddition product **3bd** (61 mg, 70% yield) as a colorless oil.
^{1}H NMR (500 MHz, $CDCl_3$) δ 7.53 (d, *J* = 1.7 Hz, 1H), 7.21 (td, *J* = 7.4, 1.2 Hz, 1H), 7.16 – 7.06 (m, 3H), 7.03 – 6.98 (m, 1H), 6.48 (dd, *J* = 3.5, 1.7 Hz, 1H), 3.29 (dd, *J* = 17.1, 9.2 Hz, 1H), 2.82 (dq, *J* = 9.1, 2.4 Hz, 1H), 2.67 (dd, *J* = 9.6, 6.6 Hz, 1H), 2.56 (dd, *J* = 17.2, 3.1 Hz, 1H), 2.47 (td, *J* = 3.1, 1.8 Hz, 1H), 2.29 (dd, *J* = 6.7, 3.0 Hz, 1H), 1.73 (dt, *J* = 8.0, 2.8 Hz, 1H), 1.37 (dd, *J* = 9.6, 7.9 Hz, 1H), 0.88 – 0.78 (m, 12H), 0.77 – 0.69 (m, 9H).
^{13}C NMR (125 MHz, $CDCl_3$) δ 190.0, 153.5, 146.2, 145.3, 142.6, 129.0, 126.4, 125.7, 124.5, 119.3, 111.9, 96.8, 66.3, 53.5, 43.4, 39.5, 38.8, 34.6, 18.3, 17.8, 13.1.
ATR-IR ν (cm-1): 2943 (s), 2865 (s), 1653 (s), 1562 (m), 1463 (s), 1248 (s), 1119 (s), 882 (s), 842 (s), 751 (s)
HRMS (ESI): Calcd for $C_{27}H_{36}O_3SiNa^+$ ($M+Na^+$): 459.2332, Found: 459.2344.

(Rac-)Thiophen-2-yl((3aR,8aR)-3a-((triisopropylsilyl)oxy)-1,3a,8,8a-tetrahydro-1,3-methanocyclopenta[a]inden-3(2H)-yl)methanone (3be)

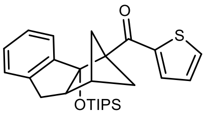

The title compound was prepared according to the General Procedure F with silyl enol ether **1ag** and BCB **2e** in toluene within 2 h. Purification by flash column chromatography (0-10% ethyl acetate in hexane) provided the cycloaddition product **3be** (70 mg, 78% yield) as a white solid.
m.p.: 80 °C.
^{1}H NMR (500 MHz, $CDCl_3$) δ 7.67 (dd, J = 3.8, 1.1 Hz, 1H), 7.60 (dd, J = 5.0, 1.1 Hz, 1H), 7.22 (td, J = 7.4, 1.3 Hz, 1H), 7.16 – 7.08 (m, 2H), 7.05 (dd, J = 4.9, 3.8 Hz, 1H), 7.00 (d, J = 7.6 Hz, 1H), 3.30 (dd, J = 17.2, 9.3 Hz, 1H), 2.91 – 2.80 (m, 1H), 2.70 (dd, J = 9.6, 6.5 Hz, 1H), 2.57 (dd, J = 17.1, 3.1 Hz, 1H), 2.48 (td, J = 3.1, 1.9 Hz, 1H), 2.33 (dd, J = 6.6, 3.1 Hz, 1H), 1.77 (dt, J = 7.9, 2.8 Hz, 1H), 1.39 (dd, J = 9.7, 7.9 Hz, 1H), 0.92 – 0.77 (m, 12H), 0.77 – 0.66 (m, 9H).
^{13}C NMR (125 MHz, $CDCl_3$) δ 194.1, 145.4, 145.4, 142.5, 134.4, 133.4, 129.0, 127.8, 126.4, 125.9, 124.5, 96.6, 67.3, 53.7, 43.8, 39.4, 39.2, 34.6, 18.3, 17.8, 13.2.
ATR-IR ν (cm-1): 2944 (s), 2865 (s), 1639 (s), 1415 (s), 1248 (m), 1135 (s), 882 (m),

790 (m), 716 (s).

HRMS (ESI): Calcd for $C_{27}H_{37}O_2SSi^+$ (M+H$^+$): 453.2284, Found: 453.2283.

(Rac-)1-((3aR,8aR)-3a-((Triisopropylsilyl)oxy)-1,3a,8,8a-tetrahydro-1,3-methanocyclopenta[a]inden-3(2H)-yl)pentan-1-one (3bf)

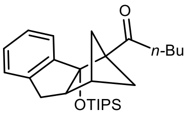

The title compound was prepared according to the General Procedure F with silyl enol ether **1ag** and BCB **2f** in toluene within 4 h. Purification by flash column chromatography (0-10% ethyl acetate in hexane) provided the cycloaddition product **3bf** (67 mg, 78% yield) as a white solid.

m.p.: 93 °C.

^{1}H NMR (500 MHz, CDCl$_3$) δ 7.20 (td, *J* = 7.3, 1.5 Hz, 1H), 7.15 (t, *J* = 7.4 Hz, 1H), 7.10 (dt, *J* = 7.6, 1.6 Hz, 2H), 3.24 (dd, *J* = 17.2, 9.2 Hz, 1H), 2.84 (ddd, *J* = 18.2, 9.3, 5.7 Hz, 1H), 2.76 (dq, *J* = 9.3, 2.6 Hz, 1H), 2.58 (ddd, *J* = 18.2, 9.3, 5.9 Hz, 1H), 2.49 (dd, *J* = 17.2, 3.1 Hz, 1H), 2.45 – 2.34 (m, 2H), 2.01 (dd, *J* = 6.6, 3.0 Hz, 1H), 1.66 – 1.50 (m, 3H), 1.41 – 1.28 (m, 2H), 1.12 (dd, *J* = 9.7, 8.0 Hz, 1H), 1.00 – 0.77 (m, 24H).

^{13}C NMR (125 MHz, CDCl$_3$) δ 212.2, 145.2, 142.8, 128.9, 126.6, 125.2, 124.5, 96.2, 67.5, 53.1, 42.2, 41.6, 38.6, 37.3, 34.4, 25.4, 22.6, 18.3, 18.1, 14.1, 13.1.

ATR-IR ν (cm-1): 2944 (s), 2866 (s), 1698 (s), 1459 (m), 1249 (s), 1123 (s), 1065 (s), 883 (s), 825 (m), 676 (s).

HRMS (ESI): Calcd for $C_{27}H_{43}O_2Si^+$ (M+H$^+$): 427.3032, Found: 427.3029.

1-(2-(4-Methylpent-3-en-1-yl)-2-((triisopropylsilyl)oxy)bicyclo[2.1.1]hexan-1-yl)pentan-1-one (3bg)

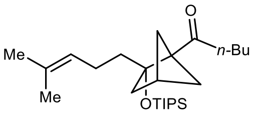

The title compound was prepared according to the General Procedure F with silyl enol ether **1aa** and BCB **2f** in DCM within 4 h. Purification by flash column chromatography (0-10% ethyl acetate in hexane) provided the cycloaddition product **3bg** (54 mg, 64% yield) with isomers from regioisomeric silyl enol ethers of 10:1:1 ratio as a yellow oil.

^{1}H NMR (500 MHz, CDCl$_3$) δ 4.99 (t, *J* = 7.0 Hz, 1H), 2.71 (ddd, *J* = 18.1, 9.4, 5.8 Hz, 1H), 2.48 (ddd, *J* = 17.9, 9.2, 5.8 Hz, 1H), 2.28 (d, *J* = 3.2 Hz, 1H), 2.25 (dd, *J* = 9.7, 6.4 Hz, 1H), 2.15 – 2.04 (m, 1H), 1.96 – 1.87 (m, 3H), 1.85 – 1.79 (m, 2H), 1.73 (dd, *J* = 7.1, 3.3 Hz, 2H), 1.65 (s, 3H), 1.56 (s, 3H), 1.54 – 1.45 (m, 3H), 1.29 (dd, *J* = 14.8, 7.4 Hz, 2H), 1.11 (dd, *J* = 5.9, 3.4 Hz, 21H), 0.89 (t, *J* = 7.3 Hz, 3H).

^{13}C NMR (125 MHz, CDCl$_3$) δ 213.7, 131.9, 124.2, 84.6, 68.7, 46.3, 42.5, 40.9, 40.8, 40.2, 32.6, 25.8, 25.5, 23.9, 22.6, 18.8, 18.8, 17.9, 14.2, 13.9.

ATR-IR ν (cm-1): 2944 (s), 2867 (s), 1696 (s), 1463 (m), 1379 (m), 1096 (m), 1036 (s), 882 (m), 672 (m).

HRMS (ESI): Calcd for $C_{26}H_{49}O_2Si^+$ (M+H$^+$): 421.3502, Found: 421.3505.

(2-Methyl-4-phenyl-2-((triisopropylsilyl)oxy)bicyclo[2.1.1]hexan-1-yl)(naphthalen-2-yl)methanone (3bh)

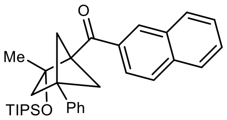

The title compound was prepared according to the General Procedure F with silyl enol ether **1w** (0.2 mmol) and BCB **2g** (0.1 mmol) in DCM (1 mL) within 24 h. Purification by flash column chromatography (0-6% ethyl acetate in hexane) provided the cycloaddition product **3bh** (27 mg, 54% yield) as a colorless oil.

^{1}H NMR (600 MHz, CDCl$_3$) δ 8.63 – 8.60 (m, 1H), 8.13 (dd, *J* = 8.6, 1.7 Hz, 1H), 7.95 (dd, *J* = 8.1, 1.3 Hz, 1H), 7.88 (t, *J* = 8.1 Hz, 2H), 7.64 – 7.59 (m, 1H), 7.59 – 7.53 (m, 1H), 7.40 – 7.34 (m, 2H), 7.28 (ddd, *J* = 10.1, 7.0, 1.5 Hz, 3H), 3.22 (dd, *J* = 9.5, 6.3 Hz, 1H), 2.44 (dd, *J* = 6.3, 2.7 Hz, 1H), 2.42 – 2.35 (m, 2H), 2.32 (dd, *J* = 10.9, 2.7 Hz, 1H), 2.20 (dd, *J* = 7.3, 3.5 Hz, 1H), 1.69 (s, 3H), 1.08 – 0.94 (m, 21H).

^{13}C NMR (150 MHz, CDCl$_3$) δ 202.3, 142.4, 135.5, 135.5, 132.5, 132.0, 129.7, 128.5, 128.3, 127.9, 127.9, 126.6, 126.6, 126.0, 125.2, 83.2, 65.3, 53.5, 48.4, 48.2, 47.0, 25.7, 18.5, 13.4, 1.2.

ATR-IR ν (cm-1): 2943 (m), 2865 (m), 1653 (m), 1464 (m), 1225 (m), 1031 (s), 1014 (s), 756 (s).

HRMS (ESI): Calcd for $C_{33}H_{42}O_2SiNa^+$ (M+Na$^+$): 521.2852, Found: 521.2839.

(3,5-Dimethyl-1H-pyrazol-1-yl)(2,4-diphenyl-2-((triisopropylsilyl)oxy)bicyclo-[2.1.1]hexan-1-yl)methanone (3bi)

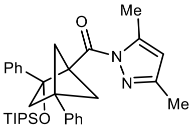

The title compound was prepared according to the General Procedure F with silyl enol ether **1a** and BCB **2h** in DCM within 4 h. Purification by flash column chromatography (0-10% ethyl acetate in hexane) provided the cycloaddition product **3bi** (45 mg, 43% yield) as a colorless oil.

^{1}H NMR (500 MHz, CDCl$_3$) δ 7.41 – 7.31 (m, 6H), 7.30 – 7.23 (m, 1H), 7.16 – 7.08 (m, 3H), 5.75 (s, 1H), 3.20 (dd, *J* = 9.5, 7.1 Hz, 1H), 3.16 (dd, *J* = 11.7, 3.0 Hz, 1H), 2.51 (d, *J* = 1.0 Hz, 3H), 2.44 (dd, *J* = 9.5, 8.0 Hz, 1H), 2.37 – 2.31 (m, 2H), 2.17 (dd, *J* = 8.0, 3.6 Hz, 1H), 1.82 (s, 3H), 1.00 – 0.89 (m, 21H).

^{13}C NMR (125 MHz, CDCl$_3$) δ 172.1, 150.6, 143.4, 143.4, 142.7, 128.6, 127.2, 127.1, 127.1, 126.6, 126.2, 110.3, 87.4, 62.4, 48.4, 48.0, 47.7, 47.0, 18.4, 18.3, 13.6, 13.6.

ATR-IR ν (cm-1): 2944 (m), 2866 (m), 1705 (s), 1374 (s), 1354 (s), 1131 (m), 885 (m).

HRMS (ESI): Calcd for $C_{33}H_{44}N_2O_2SiNa^+$(M+Na$^+$): 551.3069, Found: 551.3076.

Characterization of formal (4+3) cycloaddition products

Naphthalen-2-yl(9-((triisopropylsilyl)oxy)-1,2,3,4,4a,6,7,8-octahydro-5H-5,7-methanobenzo[7]annulen-5-yl)methanone (5a)

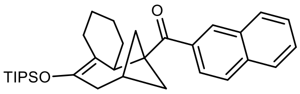

The title compound was prepared according to the General Procedure F with silyl enol ether **1az** and BCB **2a** in toluene for 3 h. Purification by flash column chromatography (0-3% ethyl acetate in hexane) provided the cycloaddition product **5a** (58 mg, 65% yield) as a white solid.
m.p.: 125 ℃.
^{1}H NMR (600 MHz, $CDCl_3$) δ 8.36 (d, *J* = 1.8 Hz, 1H), 7.94 (dd, *J* = 8.6, 1.7 Hz, 1H), 7.91 (d, *J* = 8.1 Hz, 1H), 7.87 (d, *J* = 8.5 Hz, 2H), 7.59 (ddd, *J* = 8.2, 6.8, 1.3 Hz, 1H), 7.54 (ddd, *J* = 8.1, 6.8, 1.3 Hz, 1H), 3.34 (ddd, *J* = 12.9, 2.7 Hz, 1H), 2.88 – 2.81 (m, 2H), 2.64 (dd, *J* = 10.6, 8.4 Hz, 1H), 2.59 (q, *J* = 2.2, 1.6 Hz, 2H), 2.45 (dddd, *J* = 24.1, 10.7, 4.8, 1.7 Hz, 2H), 1.83 (ddd, *J* = 11.6, 5.3, 1.7 Hz, 1H), 1.69 – 1.62 (m, 2H), 1.55 – 1.46 (m, 1H), 1.32 – 1.25 (m, 3H), 1.25 – 1.12 (m, 21H), 1.11 – 1.04 (m, 1H).
^{13}C NMR (150 MHz, $CDCl_3$) δ 204.4, 140.6, 135.3, 132.5, 132.5, 130.7, 129.7, 128.4, 128.3, 127.8, 126.7, 125.4, 117.9, 55.3, 47.4, 40.6, 36.2, 29.9, 29.7, 29.4, 27.2, 26.8, 26.5, 18.5, 18.4, 13.7.
ATR-IR ν (cm-1): 2925 (s), 2864 (m), 1669 (s), 1463 (m), 1170 (s), 999 (m), 882 (m), 760 (m).
HRMS (ESI): Calcd for $C_{33}H_{45}O_2Si^+$ ($M+H^+$): 489.3189, Found: 489.3188.

Naphthalen-2-yl(8-((triisopropylsilyl)oxy)-2,3,3a,5,6,7-hexahydro-4,6-methanoazulen-4(1H)-yl)methanone (5b)

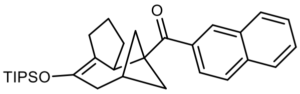

The title compound was prepared according to the General Procedure G with silyl enol ether **1ay** and BCB **2a** in toluene for 30 min. Purification by flash column chromatography (0-10% ethyl acetate in hexane) provided the cycloaddition product **5b** (74 mg, 77% yield) as a pale yellow foam.
^{1}H NMR (500 MHz, $CDCl_3$) δ 8.38 (d, *J* = 1.7 Hz, 1H), 7.97 (dd, *J* = 8.6, 1.7 Hz, 1H), 7.93 (d, *J* = 8.0 Hz, 1H), 7.87 (dd, *J* = 8.4, 4.3 Hz, 2H), 7.59 (ddd, *J* = 8.2, 6.8, 1.4 Hz, 1H), 7.54 (ddd, *J* = 8.2, 6.8, 1.4 Hz, 1H), 3.06 – 2.98 (m, 1H), 2.88 (t, *J* = 9.8 Hz, 1H), 2.66 (ddd, *J* = 23.1, 14.5, 8.4 Hz, 3H), 2.55 – 2.45 (m, 2H), 2.35 – 2.23 (m, 2H), 2.01 – 1.93 (m, 1H), 1.72 – 1.63 (m, 1H), 1.42 – 1.32 (m, 1H), 1.25 – 1.17 (m, 5H), 1.14 (d, *J* = 6.5 Hz, 18H).
^{13}C NMR (125 MHz, $CDCl_3$) δ 203.9, 140.7, 135.4, 132.8, 132.5, 130.6, 129.7, 128.4, 128.4, 127.9, 126.7, 125.2, 121.7, 52.4, 50.7, 40.1, 39.2, 29.3, 28.6, 28.5, 26.5, 22.6, 18.4, 18.4, 13.5.
ATR-IR ν (cm-1): 2943 (s), 2865 (m), 1671 (s), 1464 (m), 1275 (m), 1203 (m), 1150

(s), 883 (m), 679 (m).
HRMS (ESI): Calcd for $C_{31}H_{43}O_2Si^+$ (M+H$^+$): 475.3032, Found: 475.3032.

Naphthalen-2-yl(5-((triisopropylsilyl)oxy)-3,4,6,7,8,9,10,10a-octahydro-1,3-methanoheptalen-1(2H)-yl)methanone (5c)

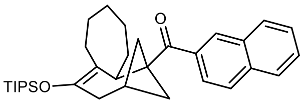

The title compound was prepared according to the General Procedure G with silyl enol ether **1ba** and BCB **2a** in toluene for 30 min. Purification by flash column chromatography (0-10% ethyl acetate in hexane) provided the cycloaddition product **5c** (74 mg, 73% yield) as a white solid.
m.p.: 130 ℃.
^{1}H NMR (500 MHz, CDCl$_3$) δ 8.35 (d, *J* = 1.6 Hz, 1H), 7.92 (dd, *J* = 8.6, 1.8 Hz, 2H), 7.85 (d, *J* = 8.5 Hz, 2H), 7.57 (ddd, *J* = 8.2, 6.8, 1.4 Hz, 1H), 7.52 (ddd, *J* = 8.0, 6.7, 1.3 Hz, 1H), 3.10 (t, *J* = 6.0 Hz, 1H), 2.84 (td, *J* = 11.7, 7.6 Hz, 2H), 2.66 – 2.50 (m, 3H), 2.44 (ddd, *J* = 11.2, 5.5, 1.8 Hz, 2H), 2.08 – 1.98 (m, 1H), 1.81 – 1.74 (m, 1H), 1.72 – 1.62 (m, 1H), 1.51 – 1.31 (m, 6H), 1.25 – 1.18 (m, 3H), 1.15 (dd, *J* = 7.1, 2.1 Hz, 18H), 1.13 – 1.06 (m, 1H).
^{13}C NMR (125 MHz, CDCl$_3$) δ 204.2, 142.2, 135.2, 132.6, 132.5, 130.6, 129.7, 128.3, 128.2, 127.8, 126.7, 125.4, 118.3, 55.8, 46.5, 40.7, 35.6, 32.4, 31.7, 30.0, 29.5, 26.9, 26.4, 25.9, 18.5, 13.8.
ATR-IR ν (cm-1): 2924 (s), 2865 (m), 1671 (m), 1463 (m), 1163 (m), 882 (m) 677 (m).
HRMS (ESI): Calcd for $C_{33}H_{47}O_2Si^+$ (M+H$^+$): 503.3345, Found: 503.3339.

3-Methyl-4-((triisopropylsilyl)oxy)bicyclo[4.1.1]oct-3-en-1-yl)(naphthalen-2-yl)methanone (5d)

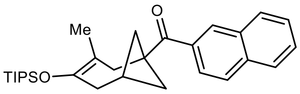

The title compound was prepared according to the General Procedure G with silyl enol ether **1at** and BCB **2a** in DCM for 30 min. Purification by flash column chromatography (0-10% ethyl acetate in hexane) provided the cycloaddition product **5d** (63 mg, 70% yield) as a white solid.
m.p.: 105 ℃.
^{1}H NMR (500 MHz, CDCl$_3$) δ 8.36 (d, *J* = 1.7 Hz, 1H), 7.96 (dd, *J* = 8.6, 1.8 Hz, 1H), 7.91 (d, *J* = 8.1 Hz, 1H), 7.86 (dd, *J* = 8.4, 5.8 Hz, 2H), 7.59 (ddd, *J* = 8.2, 6.8, 1.4 Hz, 1H), 7.54 (ddd, *J* = 8.1, 6.7, 1.3 Hz, 1H), 2.74 (td, *J* = 8.8, 2.8 Hz, 2H), 2.60 (d, *J* = 3.8 Hz, 4H), 2.50 – 2.44 (m, 1H), 2.17 – 2.11 (m, 2H), 1.75 (s, 3H), 1.27 – 1.19 (m, 3H), 1.15 (d, *J* = 6.8 Hz, 18H).
^{13}C NMR (125 MHz, CDCl$_3$) δ 204.7, 142.9, 135.4, 132.5, 131.9, 130.9, 129.7, 128.5, 128.3, 127.8, 126.8, 125.2, 110.4, 51.1, 42.9, 40.1, 34.5, 26.0, 19.1, 18.4, 13.6.
ATR-IR ν (cm-1): 2926 (s), 2866 (m), 1673 (s), 1464 (m), 1157 (s), 885 (m).

HRMS (ESI): Calcd for $C_{29}H_{41}O_2Si^+$ (M+H$^+$): 449.2876, Found: 449.2870.

Naphthalen-2-yl(3-phenyl-4-((triisopropylsilyl)oxy)bicyclo[4.1.1]oct-3-en-1-yl)methanone (5e)

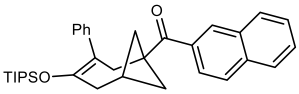

The title compound was prepared according to the General Procedure G with silyl enol ether **1bb** and BCB **2a** in toluene for 30 min. Purification by flash column chromatography (0-10% ethyl acetate in hexane) provided the cycloaddition product **5e** (75 mg, 52% yield) as a colorless oil.

^{1}H NMR (500 MHz, $CDCl_3$) δ 8.37 (d, *J* = 1.7 Hz, 1H), 7.97 – 7.89 (m, 2H), 7.85 (dd, *J* = 8.4, 3.8 Hz, 2H), 7.58 (ddd, *J* = 8.2, 6.8, 1.4 Hz, 1H), 7.53 (ddd, *J* = 8.2, 6.8, 1.3 Hz, 1H), 7.29 – 7.22 (m, 4H), 7.16 – 7.09 (m, 1H), 2.90 – 2.80 (m, 4H), 2.76 – 2.69 (m, 2H), 2.60 – 2.52 (m, 1H), 2.35 – 2.29 (m, 2H), 0.99 – 0.90 (m, 21H).
^{13}C NMR (125 MHz, $CDCl_3$) δ 204.5, 144.0, 143.3, 135.4, 132.5, 131.9, 130.9, 129.7, 129.7, 128.5, 128.3, 128.0, 127.8, 126.7, 125.9, 125.1, 116.5, 51.4, 43.7, 40.5, 34.6, 26.0, 18.1, 13.5.
ATR-IR ν (cm-1): 2942 (s), 2865 (s), 1668 (s), 1464 (m), 1184 (s), 1149 (s), 883 (m).
HRMS (ESI): Calcd for $C_{34}H_{43}O_2Si^+$ (M+H$^+$): 511.3032, Found: 511.3032.

(3-Isobutyl-4-((triisopropylsilyl)oxy)bicyclo[4.1.1]oct-3-en-1-yl)(naphthalen-2-yl)methanone (5f)

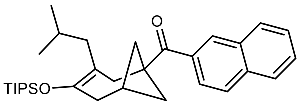

The title compound was prepared according to the General Procedure G with silyl enol ether **1au** and BCB **2a** in toluene for 30 min. Purification by flash column chromatography (0-10% ethyl acetate in hexane) provided the cycloaddition product **5f** (75 mg, 76% yield) as a colorless oil.
^{1}H NMR (500 MHz, $CDCl_3$) δ 8.38 (d, *J* = 1.7 Hz, 1H), 7.96 (dd, *J* = 8.7, 1.7 Hz, 1H), 7.89 (dt, *J* = 15.8, 7.8 Hz, 3H), 7.59 (ddd, *J* = 8.2, 6.7, 1.4 Hz, 1H), 7.54 (ddd, *J* = 8.1, 6.8, 1.3 Hz, 1H), 2.78 – 2.70 (m, 2H), 2.63 – 2.59 (m, 4H), 2.50 – 2.43 (m, 1H), 2.15 – 2.09 (m, 2H), 2.06 (d, *J* = 7.7 Hz, 2H), 1.89 – 1.79 (m, 1H), 1.25 – 1.11 (m, 21H), 0.90 (d, *J* = 6.6 Hz, 6H).
^{13}C NMR (125 MHz, $CDCl_3$) δ 204.7, 143.6, 135.4, 132.6, 131.8, 131.0, 129.7, 128.5, 128.3, 127.8, 126.8, 125.2, 113.4, 51.0, 41.3, 41.1, 40.2, 34.6, 27.2, 26.1, 22.5, 18.5, 13.8.
ATR-IR ν (cm-1): 2946 (s), 2865 (s), 1668 (s), 1463 (m), 1151 (s), 883 (m), 776 (m), 679 (m).
HRMS (ESI): Calcd for $C_{32}H_{47}O_2Si^+$ (M+H$^+$): 491.3345, Found: 491.3349.

5-Methyl-4-((triisopropylsilyl)oxy)bicyclo[4.1.1]oct-3-en-1-yl)(naphthalen-2-yl)methanone (5g)

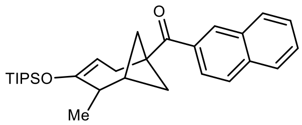

The title compound was prepared according to the General Procedure G with silyl enol ether **1av** and BCB **2a** in DCM for 30 min. Purification by flash column chromatography (0-10% ethyl acetate in hexane) provided the cycloaddition product **5g** (45 mg, 50% yield, 10:1 rr) as a white foam. (rr: the ratio of (4+3) cycloadduct to (2+3) cycloadduct)

^{1}H NMR (500 MHz, CDCl$_3$) δ 8.35 (d, *J* = 1.7 Hz, 1H), 7.96 – 7.90 (m, 2H), 7.86 (dd, *J* = 8.4, 4.6 Hz, 2H), 7.58 (ddd, *J* = 8.2, 6.8, 1.4 Hz, 1H), 7.53 (ddd, *J* = 8.2, 6.8, 1.4 Hz, 1H), 4.85 (t, *J* = 4.1 Hz, 1H), 2.74 (dd, *J* = 11.1, 8.9 Hz, 1H), 2.69 – 2.59 (m, 2H), 2.56 – 2.49 (m, 2H), 2.27 (tdt, *J* = 6.5, 4.0, 2.0 Hz, 1H), 2.21 (ddd, *J* = 11.4, 4.9, 1.9 Hz, 1H), 2.14 (ddd, *J* = 11.1, 5.2, 1.9 Hz, 1H), 1.26 – 1.17 (m, 6H), 1.13 (dd, *J* = 7.2, 4.8 Hz, 18H).

^{13}C NMR (125 MHz, CDCl$_3$) δ 205.0, 153.2, 135.4, 132.5, 132.0, 130.8, 129.7, 128.5, 128.3, 127.9, 126.8, 125.2, 101.8, 51.4, 41.8, 36.2, 33.6, 32.4, 32.0, 18.3, 16.9, 12.9.

ATR-IR ν (cm-1): 2942 (m), 2865 (m), 1667 (m), 1463 (m), 1279 (m), 1171 (s), 881 (s), 760 (m), 680 (m).

HRMS (ESI): Calcd for $C_{29}H_{41}O_2Si^+$ (M+H$^+$): 449.2876, Found: 449.2868.

2-Methyl-4-((triisopropylsilyl)oxy)bicyclo[4.1.1]oct-3-en-1-yl)(naphthalen-2-yl)methanone (5h)

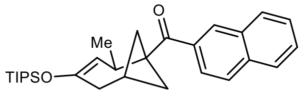

The title compound was prepared according to the General Procedure G with silyl enol ether **1aw** and BCB **2a** in DCM for 30 min. Purification by flash column chromatography (0-3% ethyl acetate in hexane) provided the cycloaddition product **5h** (43 mg, 48% yield) as a colorless oil.

^{1}H NMR (500 MHz, CDCl$_3$) δ 8.36 (s, 1H), 7.95 (dd, *J* = 8.6, 1.7 Hz, 1H), 7.91 (d, *J* = 8.1 Hz, 1H), 7.86 (dd, *J* = 8.4, 3.6 Hz, 2H), 7.58 (t, *J* = 7.4 Hz, 1H), 7.54 (t, *J* = 7.6 Hz, 1H), 4.90 (d, *J* = 4.1 Hz, 1H), 3.18 – 3.11 (m, 1H), 2.85 (dd, *J* = 11.9, 8.5 Hz, 1H), 2.57 – 2.40 (m, 4H), 1.93 – 1.86 (m, 1H), 1.24 – 1.15 (m, 3H), 1.13 (dd, *J* = 7.2, 2.3 Hz, 18H), 0.78 (d, *J* = 6.8 Hz, 3H).

^{13}C NMR (125 MHz, CDCl$_3$) δ 203.9, 148.1, 135.3, 132.5, 132.5, 130.7, 129.7, 128.4, 128.3, 127.8, 126.7, 125.3, 111.8, 54.8, 39.5, 38.2, 36.6, 28.6, 26.2, 18.2, 17.0, 12.8.

ATR-IR ν (cm-1): 2943 (m), 2866 (m), 1670 (s), 1463 (m), 1172 (s), 883 (m), 754 (m).

HRMS (ESI): Calcd for $C_{29}H_{41}O_2Si^+$ (M+H$^+$): 449.2876, Found: 449.2876.

2-(2-Methylprop-1-en-1-yl)-4-((triisopropylsilyl)oxy)bicyclo[4.1.1]oct-3-en-1-

yl)(naphthalen-2-yl)methanone (5i)

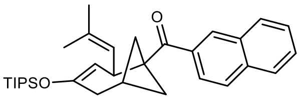

The title compound was prepared according to the General Procedure G with silyl enol ether **1ax** and BCB **2a** in toluene for 30 min. Purification by flash column chromatography (0-10% ethyl acetate in hexane) provided the cycloaddition product **5i** (40 mg, 41% yield) as a colorless oil.

^{1}H NMR (500 MHz, CDCl$_3$) δ 8.28 (s, 1H), 7.91 (d, *J* = 7.6 Hz, 1H), 7.87 – 7.82 (m, 3H), 7.55 (dddd, *J* = 21.9, 8.2, 6.9, 1.4 Hz, 2H), 4.96 (dt, *J* = 10.1, 1.5 Hz, 1H), 4.76 (d, *J* = 4.2 Hz, 1H), 3.86 – 3.79 (m, 1H), 2.89 (dd, *J* = 11.5, 8.4 Hz, 1H), 2.74 (dd, *J* = 10.8, 8.3 Hz, 1H), 2.57 – 2.37 (m, 4H), 2.03 – 1.96 (m, 1H), 1.48 (d, *J* = 1.4 Hz, 3H), 1.22 – 1.13 (m, 3H), 1.12 (dd, *J* = 7.0, 2.8 Hz, 18H), 0.87 (d, *J* = 1.4 Hz, 3H).

^{13}C NMR (125 MHz, CDCl$_3$) δ 203.7, 148.5, 135.1, 133.2, 132.5, 132.5, 130.2, 129.6, 128.2, 128.1, 127.8, 126.6, 125.3, 122.7, 109.4, 54.5, 43.0, 39.5, 36.4, 29.2, 26.2, 25.8, 18.2, 17.6, 12.9.

ATR-IR ν (cm-1): 2926 (s), 2865 (m), 1672 (s), 1464 (m), 1382 (m), 1170 (s), 898 (m), 757 (s).

HRMS (ESI): Calcd for $C_{32}H_{45}O_2Si^+$ (M+H$^+$): 489.3189, Found: 489.3183.

Phenyl(9-((triisopropylsilyl)oxy)-1,2,3,4,4a,6,7,8-octahydro-5H-5,7-methanobenzo[7]annulen-5-yl)methanone (5j)

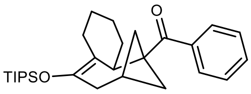

The title compound was prepared according to the General Procedure F with silyl enol ether **1az** and BCB **2b** in toluene for 3 h. Purification by flash column chromatography (0-3% ethyl acetate in hexane) provided the cycloaddition product **5j** (73 mg, 80% yield, 14:1 rr) as a colorless oil. (rr: the ratio of (4+3) cycloadduct to (2+3) cycloadduct)

^{1}H NMR (600 MHz, CDCl$_3$) δ 7.83 (dd, *J* = 8.4, 1.3 Hz, 1H), 7.53 – 7.48 (m, 1H), 7.44 – 7.38 (m, 1H), 3.34 – 3.28 (m, 1H), 2.77 (dd, *J* = 11.7, 8.8 Hz, 1H), 2.75 – 2.71 (m, 1H), 2.56 – 2.49 (m, 3H), 2.42 (ddt, *J* = 9.0, 3.5, 1.6 Hz, 1H), 2.31 – 2.25 (m, 1H), 1.77 (dd, *J* = 11.4, 3.6 Hz, 1H), 1.70 – 1.61 (m, 2H), 1.48 (tq, *J* = 13.4, 2.6, 2.1 Hz, 1H), 1.28 – 1.17 (m, 6H), 1.13 (dd, *J* = 7.2, 2.9 Hz, 18H), 1.07 – 1.00 (m, 1H).

^{13}C NMR (150 MHz, CDCl$_3$) δ 204.5, 140.5, 135.3, 132.4, 129.3, 128.4, 117.7, 55.1, 47.2, 40.6, 35.9, 29.9, 29.7, 29.3, 27.2, 26.8, 26.4, 18.4, 18.4, 13.6.

ATR-IR ν (cm-1): 2926 (s), 2865 (m), 1672 (s), 1446 (m), 1242 (m), 1171 (s), 1134 (m), 998 (m), 882 (m), 678 (m).

HRMS (ESI): Calcd for $C_{28}H_{43}O_2Si^+$ (M+H$^+$): 439.3032, Found: 439.3033.

Benzo[d][1,3]dioxol-5-yl(9-((triisopropylsilyl)oxy)-1,2,3,4,4a,6,7,8-octahydro-5H-5,7-methanobenzo[7]annulen-5-yl)methanone (5k)

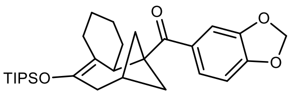

The title compound was prepared according to the General Procedure G with silyl enol ether **1az** and BCB **2c** in DCM for 3 h. Purification by flash column chromatography (0-3% ethyl acetate in hexane) provided the (4+3) cycloaddition product **5k** (42 mg, 43% yield) as a white solid.
^{1}H NMR (500 MHz, CDCl$_3$) δ 7.44 (dd, *J* = 8.2, 1.7 Hz, 1H), 7.34 (d, *J* = 1.6 Hz, 1H), 6.80 (d, *J* = 8.1 Hz, 1H), 6.02 (s, 2H), 3.31 (dd, *J* = 12.9, 2.3 Hz, 1H), 2.75 – 2.67 (m, 2H), 2.53 – 2.45 (m, 3H), 2.43 – 2.34 (m, 1H), 2.23 (dd, *J* = 10.8, 4.6 Hz, 1H), 1.76 (dd, *J* = 11.7, 4.6 Hz, 1H), 1.69 – 1.64 (m, 2H), 1.48 (td, *J* = 13.3, 11.6, 5.2 Hz, 1H), 1.27 – 1.08 (m, 25H).
^{13}C NMR (125 MHz, CDCl$_3$) δ 202.6, 151.2, 147.9, 140.5, 129.7, 125.4, 117.7, 109.4, 107.8, 101.8, 54.9, 47.6, 40.6, 36.1, 30.0, 29.7, 29.6, 27.2, 26.9, 26.4, 18.4, 18.4, 13.6.
ATR-IR ν (cm-1): 2926 (s), 2865 (m), 1667 (m), 1487 (m), 1437 (m), 1251 (s), 1172 (s), 1134 (m), 1040 (m), 882 (m).
HRMS (ESI): Calcd for $C_{29}H_{43}O_4Si^+$ (M+H$^+$): 483.2931, Found: 483.2923.

Thiophen-2-yl(9-((triisopropylsilyl)oxy)-1,2,3,4,4a,6,7,8-octahydro-5H-5,7-methanobenzo[7]annulen-5-yl)methanone (5l)

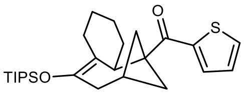

The title compound was prepared according to the General Procedure G with silyl enol ether **1az** and BCB **2e** in DCM for 3 h. Purification by flash column chromatography (0-3% ethyl acetate in hexane) provided the (4+3) cycloaddition product **5l** (40 mg, 45% yield) as a colorless oil.
^{1}H NMR (500 MHz, CDCl$_3$) δ 7.61 – 7.55 (m, 2H), 7.09 (dd, *J* = 4.9, 3.8 Hz, 1H), 3.36 – 3.28 (m, 1H), 2.79 – 2.73 (m, 1H), 2.67 (dd, *J* = 11.6, 8.6 Hz, 1H), 2.52 (dt, *J* = 3.9, 1.9 Hz, 2H), 2.48 (dd, *J* = 10.7, 8.6 Hz, 1H), 2.44 – 2.37 (m, 1H), 2.24 – 2.17 (m, 1H), 1.77 (ddd, *J* = 11.5, 5.2, 1.6 Hz, 1H), 1.73 – 1.65 (m, 2H), 1.55 – 1.45 (m, 1H), 1.40 – 1.33 (m, 1H), 1.29 – 1.20 (m, 3H), 1.22 – 1.09 (m, 21H).
^{13}C NMR (126 MHz, CDCl$_3$) δ 197.8, 141.8, 140.6, 132.9, 132.2, 127.9, 117.7, 54.9, 48.0, 40.5, 35.6, 30.0, 29.7, 29.4, 27.2, 26.9, 26.3, 18.4, 18.4, 13.6.
ATR-IR ν (cm-1): 2926 (s), 2864 (m), 1652 (s), 1412 (m), 1239 (m), 1171 (s), 998 (s), 882 (m), 679 (m).
HRMS (ESI): Calcd for $C_{26}H_{41}O_2Si^+$ (M+H$^+$): 445.2596, Found: 445.2587.

1-(9-((Triisopropylsilyl)oxy)-1,2,3,4,4a,6,7,8-octahydro-5H-5,7-methanobenzo[7]annulen-5-yl)pentan-1-one (5m)

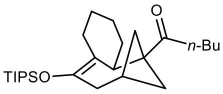

The title compound was prepared according to the General Procedure F with silyl enol

ether **1az** and BCB **2f** in toluene for 3 h. Purification by flash column chromatography (0-3% ethyl acetate in hexane) provided the cycloaddition product **5m** (42 mg, 36% yield) as a colorless oil.

^{1}H NMR (500 MHz, CDCl$_3$) δ 3.33 – 3.24 (m, 1H), 2.55 (dd, *J* = 11.5, 8.7 Hz, 1H), 2.48 – 2.43 (m, 3H), 2.40 – 2.25 (m, 3H), 2.02 (dd, *J* = 10.7, 8.5 Hz, 1H), 1.82 (ddd, *J* = 10.7, 5.1, 1.6 Hz, 1H), 1.74 (ddt, *J* = 17.7, 15.1, 3.7 Hz, 2H), 1.58 – 1.45 (m, 4H), 1.39 – 1.23 (m, 3H), 1.21 – 1.13 (m, 6H), 1.10 (d, *J* = 6.8 Hz, 18H), 0.90 (t, *J* = 7.4 Hz, 3H).

^{13}C NMR (125 MHz, CDCl$_3$) δ 213.7, 140.5, 117.4, 55.6, 46.3, 40.5, 36.0, 33.7, 30.1, 29.9, 27.2, 27.1, 27.0, 26.1, 25.2, 22.7, 18.4, 14.1, 13.6.

ATR-IR ν (cm-1): 2926 (s), 2865 (m), 1704 (m), 1655 (m), 1464 (m), 1172 (s), 1135 (m), 997 (m), 883 (m).

HRMS (ESI): Calcd for $C_{26}H_{47}O_2Si^+$ (M+H$^+$): 419.3345, Found: 419.3344.

Unsuccessful (4+3) cycloaddition with 3-substituted BCB

The cycloaddition of silyl dienol ether **1az** and 3-phenyl BCB **2g** did not give desired cycloadduct, and only minor byproduct cyclobutene **S12** was identified.

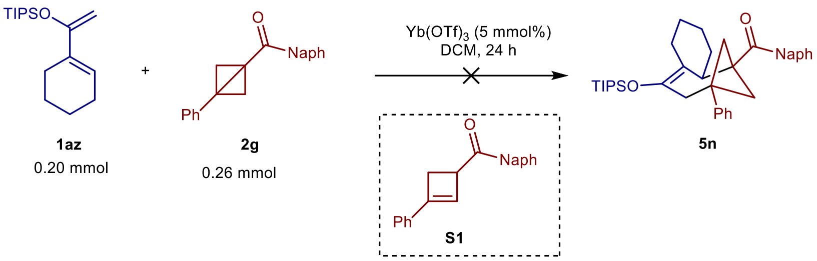

Naphthalen-2-yl(3-phenylcyclobut-2-en-1-yl)methanone (S1)

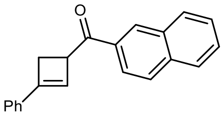

^{1}H NMR (500 MHz, CDCl$_3$) δ 8.55 (d, *J* = 1.7 Hz, 1H), 8.08 (dd, *J* = 8.6, 1.7 Hz, 1H), 8.00 (d, *J* = 8.1 Hz, 1H), 7.92 (dd, *J* = 15.2, 8.3 Hz, 2H), 7.60 (dddd, *J* = 22.4, 8.2, 6.9, 1.4 Hz, 2H), 7.43 – 7.38 (m, 2H), 7.37 – 7.32 (m, 2H), 7.31 – 7.26 (m, 1H), 6.54 (d, *J* = 1.3 Hz, 1H), 4.65 (dd, *J* = 3.5, 1.6 Hz, 1H), 3.33 – 3.18 (m, 2H).

^{13}C NMR (125 MHz, CDCl$_3$) δ 199.4, 147.9, 135.8, 134.0, 133.6, 132.8, 129.9, 129.7, 128.7, 128.6, 128.5, 128.4, 128.0, 126.9, 125.9, 124.9, 124.2, 46.1, 32.8.

ATR-IR ν (cm-1): 2914 (m), 1673 (s), 1626 (m), 1178 (m), 822 (m), 747 (s).

HRMS (ESI): Calcd for $C_{21}H_{17}O^+$ (M+H$^+$): 285.1279, Found: 285.1281.

1.5 Applications

Wittig reaction:

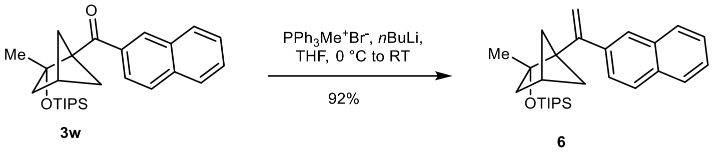

Triisopropyl((2-methyl-1-(1-(naphthalen-2-yl)vinyl)bicyclo[2.1.1]hexan-2-yl)oxy)silane (6): In an oven dried 100 mL Schlenk tube purged with nitrogen, $Ph_3P^+MeBr^-$ (632 mg, 1.67 mmol, 1.50 equiv.) was stirred in dry THF (10.0 mL). The suspension was cooled to -78 °C, and *n*BuLi (1.60 M solution in hexane, 1.00 mL, 1.51 mmol, 1.40 equiv.) was added dropwise. The mixture turned yellow and was stirred for 30 minutes at the same temperature. To the mixture, the ketone **3w** (450 mg, 1.06 mmol, 1.00 equiv.) solution in THF (2.0 mL) was subsequently added dropwise. The reaction was then warmed to room temperature and stirred for 3 hours. The reaction was quenched with saturated NH_4Cl aqueous solution (10 mL) and extracted with DCM (6 × 10 mL). The combined organic phase was washed with brine (20 mL), dried with anhydrous Na_2SO_4, filtered, concentrated, and purified by flash column chromatography (SiO_2, 0 – 20% gradient EtOAc in petroleum ether) to deliver the alkene **6** (410 mg, 92% yield) as a colorless oil.

^{1}H NMR (500 MHz, $CDCl_3$) δ 7.91 – 7.81 (m, 3H), 7.78 (d, *J* = 8.5 Hz, 1H), 7.59 (dd, *J* = 8.5, 1.8 Hz, 1H), 7.48 (tt, *J* = 7.0, 5.2 Hz, 2H), 5.43 (d, *J* = 2.1 Hz, 1H), 5.21 (d, *J* = 2.1 Hz, 1H), 2.47 – 2.39 (m, 2H), 2.09 – 1.99 (m, 2H), 1.96 – 1.90 (m, 2H), 1.79 (dd, *J* = 9.8, 6.7 Hz, 1H), 1.32 (s, 3H), 1.11 – 0.94 (m, 21H).

^{13}C NMR (125 MHz, $CDCl_3$) δ 149.6, 140.9, 133.5, 132.6, 128.2, 127.6, 127.2, 126.6, 126.2, 125.9, 125.5, 116.6, 80.6, 65.0, 47.9, 44.6, 42.7, 33.5, 26.0, 18.6, 18.6, 13.5.

ATR-IR ν (cm-1): 2965 (s), 2865 (s), 1463 (m), 1375 (m), 1242 (m), 1168 (m), 1045 (s), 882 (m), 751 (m), 675 (m).

HRMS (ESI): Calcd for $C_{28}H_{41}OSi^+$ ($M+H^+$): 421.2927, Found: 421.2930.

Desilylation:

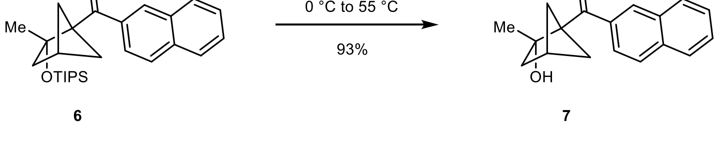

2-Methyl-1-(1-(naphthalen-2-yl)vinyl)bicyclo[2.1.1]hexan-2-ol (7): To a solution of silyl ether **5** (300 mg, 0.713 mmol, 1.00 equiv.) in THF (7.0 mL) at 0 °C, TBAF (1.0 M solution in THF, 3.50 mL, 3.50 mmol, 4.90 equiv.) was added. The reaction mixture was then slowly heated to 55 °C and was stirred for 18 hours. The reaction was cooled to room temperature, quenched with NH_4Cl aqueous solution (5.0 mL), extracted with DCM (6 × 10 mL), dried over anhydrous Na_2SO_4, filtered, concentrated, and purified by flash column chromatography (SiO_2, 0-20% gradient EtOAc in petroleum ether) to deliver the alcohol **7** (175 mg, 93% yield) as a colorless foam.

^{1}H NMR (500 MHz, $CDCl_3$) δ 7.87 – 7.78 (m, 4H), 7.53 – 7.45 (m, 3H), 5.39 (d, *J* =

1.9 Hz, 1H), 5.15 (d, J = 1.9 Hz, 1H), 2.44 (tt, J = 3.0, 1.5 Hz, 1H), 2.32 (dd, J = 9.8, 6.4 Hz, 1H), 2.08 (dt, J = 7.0, 3.7 Hz, 1H), 1.98 (dt, J = 5.7, 2.5 Hz, 1H), 1.90 – 1.83 (m, 2H), 1.75 (dd, J = 9.8, 6.9 Hz, 1H), 1.26 (s, 1H), 1.25 (s, 3H).
^{13}C NMR (125 MHz, $CDCl_3$) δ 149.5, 140.0, 133.4, 132.7, 128.2, 127.8, 127.7, 126.2, 126.1, 126.1, 126.0, 116.2, 78.6, 63.8, 45.5, 44.0, 42.7, 33.4, 25.5.
ATR-IR ν (cm-1): 3346 (br), 2971 (s), 2872 (m), 1609 (m), 1376 (m), 1095 (m), 1049 (m), 818 (m), 750 (s).
HRMS (ESI): Calcd for $C_{19}H_{21}O^+$ (M+H$^+$): 265.1692, Found: 265.1696.

Dehydration:

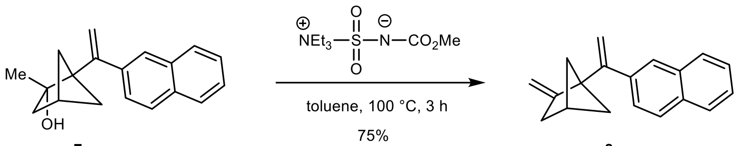

7 8

2-Methylene-1-(1-(naphthalen-2-yl)vinyl)bicyclo[2.1.1]hexane (8): An oven dried vial was charged with Burgess reagent (124 mg, 0.520 mmol) and sealed with septum. The vial was evacuated and backfilled with N_2 for 3 times. Then 2 mL toluene was added into the vial, followed by the addition of a solution of tertiary alcohol **6** (69 mg, 0.26 mmol) in dry toluene (1 mL), and the solution was stirred at 100 ℃ for 3 h. The mixture was cooled, diluted with EtOAc, washed with brine, dried over Mg_2SO_4, filtered, and concentrated. The residue was purified by preparative thin layer chromatography (0-5% EtOAc in petroleum ether) to deliver diene **8** (48 mg, 75%) as a colorless oil.
^{1}H NMR (500 MHz, $CDCl_3$) δ 7.84 (d, J = 1.9 Hz, 1H), 7.79 (dt, J = 6.6, 3.0 Hz, 2H), 7.75 (d, J = 8.6 Hz, 1H), 7.58 (dd, J = 8.6, 1.8 Hz, 1H), 7.47 – 7.38 (m, 2H), 5.60 (d, J = 1.4 Hz, 1H), 5.15 (d, J = 1.4 Hz, 1H), 4.84 – 4.79 (m, 2H), 2.57 – 2.52 (m, 1H), 2.49 – 2.44 (m, 2H), 1.98 – 1.89 (m, 2H), 1.75 (dd, J = 4.0, 2.0 Hz, 2H).
^{13}C NMR (125 MHz, $CDCl_3$) δ 154.9, 148.9, 137.7, 133.4, 132.8, 128.4, 127.6, 127.6, 126.1, 125.9, 125.8, 125.5, 114.1, 102.5, 63.7, 45.8, 36.9, 32.6.
ATR-IR ν (cm-1): 2978 (m), 2929 (m), 1666 (m), 1615 (w), 1505 (w), 1437 (w), 1261 (m), 893 (m), 818 (m), 750 (s).
HRMS (EI): Calcd for $C_{19}H_{19}^+$ (M+H$^+$): 247.1487, Found: 247.1485.

Fluorination:

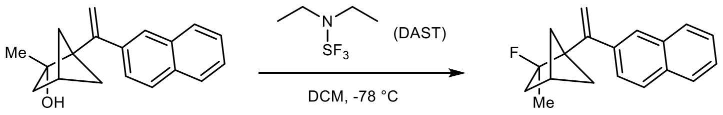

7 9

2-Fluoro-2-methyl-1-(1-(naphthalen-2-yl)vinyl)bicyclo[2.1.1]hexane (9): A blow-dried Schlenk tube purged with nitrogen was charged with DCM (1.0 mL) and cooled to -78 °C. To the solvent, diethylaminosulfur trifluoride (100 μL, 0.757 mmol, 7.50 equiv.) was added dropwise, followed by a solution of alcohol **6** (26.6 mg, 0.100 mmol, 1.00 equiv.) in DCM (1.0 mL). After 30 minutes, the reaction mixture was

quenched with saturated $NaHCO_3$ aqueous solution (5.0 mL), extracted with DCM (6 × 5 mL), dried with anhydrous Na_2SO_4, filtered, concentrated, and purified by preparative thin layer chromatography (10% EtOAc in petroleum ether) to deliver the fluorinated product **9** (19 mg, 71%) as a colorless oil.
^{1}H NMR (500 MHz, $CDCl_3$) δ 7.84 – 7.79 (m, 3H), 7.76 (d, *J* = 8.6 Hz, 1H), 7.53 – 7.41 (m, 3H), 5.41 (d, *J* = 1.8 Hz, 1H), 5.15 (d, *J* = 1.8 Hz, 1H), 2.42 (tt, *J* = 3.3, 1.7 Hz, 1H), 2.23 (ddd, *J* = 9.8, 6.6, 1.1 Hz, 1H), 2.12 – 2.03 (m, 1H), 2.03 – 1.99 (m, 1H), 1.97 – 1.88 (m, 2H), 1.65 (ddd, *J* = 9.6, 7.0, 2.4 Hz, 1H), 1.40 (d, *J* = 22.2 Hz, 3H).
^{13}C NMR (125 MHz, $CDCl_3$) δ 148.77 (d, *J* = 1.5 Hz), 139.7, 133.4, 132.7, 128.3, 127.7, 127.4, 126.2, 126.2 (d, *J* = 4.8 Hz), 126.0, 125.8, 116.4, 102.04 (d, *J* = 186.9 Hz), 62.98 (d, *J* = 21.7 Hz), 44.74 (d, *J* = 4.6 Hz), 44.44 (d, *J* = 21.4 Hz), 42.48 (d, *J* = 3.8 Hz), 32.87 (d, *J* = 1.8 Hz), 22.02 (d, *J* = 29.1 Hz).
^{19}F NMR (471 MHz, $CDCl_3$) δ -139.31 (td, *J* = 24.3, 23.7, 17.0 Hz).
ATR-IR ν (cm-1): 2980 (s), 2929 (m), 1614 (m), 1380 (m), 1151 (m), 918 (m), 918 (m), 830 (m), 742 (s).
HRMS (EI): Calcd for $C_{19}H_{20}F$ (M): 266.1471, Found: 266.1461.

Isocyanation reaction:

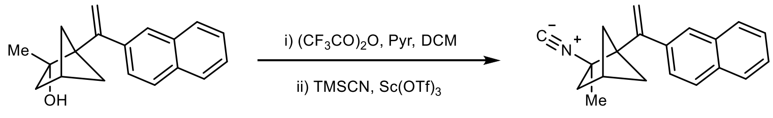

7 10

2-Isocyano-2-methyl-1-(1-(naphthalen-2-yl)vinyl)bicyclo[2.1.1]hexane (10):[19] A solution of the tertiary alcohol **6** (50 mg, 0.19 mmol, 1.0 equiv.) in dry DCM (0.5 mL) was cooled to 0 °C and treated with dry pyridine (100 μL, 1.24 mmol, 6.50 equiv.), followed by dropwise addition of trifluoroacetic anhydride (100 μL, 0.719 mmol, 3.80 equiv.). The reaction mixture was stirred for 30 minutes at the same temperature before being quenched with 1 N aqueous HCl (1 mL). The mixture was then stirred for 3 minutes and extracted with hexanes (6 × 2 mL). The combined organic layers were washed with saturated $NaHCO_3$ aqueous solution, dried over anhydrous Na_2SO_4, filtered, and concentrated under reduced pressure. The crude residue was directly used for the next step without further purification.
The resulting crude ester was dissolved in TMSCN (0.500 mL, 4.00 mmol, 21.0 equiv.) at room temperature. $Sc(OTf)_3$ (4.9 mg, 10 μmol, 0.050 equiv.) was then added to the solution. The resulting mixture was stirred for 24 hours at room temperature and concentrated under reduced pressure. The crude product was purified by preparative thin layer chromatography (10% EtOAc in petroleum ether 60-90) to deliver the isonitrile **10** (21.3 mg, 0.077 mmol, 41 % yield) as white solid.
m.p.: 49 ℃.
^{1}H NMR (500 MHz, $CDCl_3$) δ 7.87 – 7.73 (m, 4H), 7.51 – 7.40 (m, 3H), 5.42 (d, *J* = 1.5 Hz, 1H), 5.24 (d, *J* = 1.5 Hz, 1H), 2.51 (tt, *J* = 3.1, 1.4 Hz, 1H), 2.27 (dt, *J* = 11.6, 2.6 Hz, 1H), 2.20 – 2.10 (m, 2H), 2.06 (dt, *J* = 7.2, 3.0 Hz, 1H), 1.91 (ddd, *J* = 11.5, 2.9, 1.4 Hz, 1H), 1.72 (dd, *J* = 10.0, 7.4 Hz, 1H), 1.23 – 1.19 (m, 3H).

^{13}**C NMR (125 MHz, CDCl$_3$)** δ 154.0 (t, $^1J_{C\text{-}N}$ = 4.8 Hz), 147.2, 139.4, 133.4, 132.8, 128.2, 127.8, 127.7, 126.3, 126.3, 126.1, 126.0, 118.1, 64.2 (t, $^1J_{C\text{-}N}$ = 5.8 Hz), 63.3, 47.0, 43.4, 41.7, 34.2, 25.1.
ATR-IR ν (cm-1): 2979 (m), 2879 (m), **2129 (s)**, 1617 (m), 1504 (m), 1446 (m), 1273 (m), 1107 (m), 899 (m), 860 (m), 821 (s), 751 (s).
HRMS (ESI): Calcd for $C_{20}H_{20}N^+$ (M+H$^+$): 274.1596, Found: 274.1592.

Derivatization of (4+3) cycloadducts
Desilylation

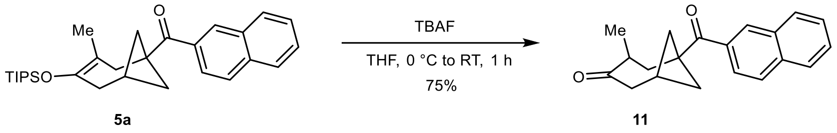

6-(2-naphthoyl)-4-methylbicyclo[4.1.1]octan-3-one (11): To a stirred solution of silyl enol ether (0.05 mmol, 23 mg) in THF (1 mL) was added TBAF (0.075 mmol, 1.5 eq. 0.08 mL) at 0 ^{o}C. After stirred for 5 min, the resulting solution was allowed warm to room temperature and stirred for 1 h. The solvent was removed under reduced pressure. The residue was purified by flash column chromatography (SiO$_2$, 5-10% EtOAc in petroleum ether) to deliver ketone **11** (11 mg, 75%) as a white solid.
^{1}**H NMR (600 MHz, CDCl$_3$)** δ 8.27 (s, 1H), 7.93 (d, *J* = 8.1 Hz, 1H), 7.88 (d, *J* = 1.4 Hz, 2H), 7.87 (d, *J* = 9.2 Hz, 1H), 7.60 (ddd, *J* = 8.1, 6.8, 1.3 Hz, 1H), 7.55 (ddd, *J* = 8.1, 6.9, 1.3 Hz, 1H), 3.30 (dp, *J* = 12.7, 6.4 Hz, 1H), 2.96 (dd, *J* = 12.2, 8.9 Hz, 1H), 2.90 (dd, *J* = 11.6, 7.8 Hz, 1H), 2.73 (t, *J* = 4.1 Hz, 2H), 2.56 (dd, *J* = 11.0, 5.7 Hz, 2H), 2.17 (ddd, *J* = 13.8, 6.2, 1.5 Hz, 1H), 1.84 (dd, *J* = 11.3, 6.1 Hz, 1H), 1.78 (dd, *J* = 13.7, 11.6 Hz, 1H), 1.14 (d, *J* = 6.4 Hz, 3H).
^{13}**C NMR (150 MHz, CDCl$_3$)** δ 213.6, 203.8, 135.5, 132.5, 131.5, 130.8, 129.8, 128.7, 128.6, 127.9, 127.0, 124.9, 52.2, 48.8, 43.3, 41.6, 36.5, 33.7, 25.2, 15.6.
ATR-IR ν (cm-1): 2930 (m), 1704 (s), 1668 (s), 1279 (m), 1122 (m), 762 (m).
HRMS (ESI): Calcd for $C_{20}H_{21}O2^+$(M+H$^+$): 293.1541, Found: 293.1545.

Dihydroxylation of silyl enol ether

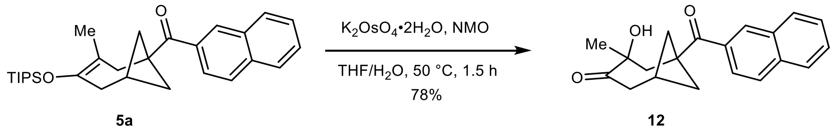

6-(2-Naphthoyl)-4-hydroxy-4-methylbicyclo[4.1.1]octan-3-one (12): An oven dried vial was charged with silyl enol ether (0.05 mmol, 23 mg), K_2OsO_4•$2H_2O$ (0.0025 mmol, 5% eq. 0.8 mg) and NMO (0.25 mmol, 5 eq. 29.3 mg). Then 0.9 mL THF and 0.3 mL H_2O was added into the vial, and the solution was stirred at 50 ℃ for 1.5 h. The mixture was cooled, diluted with DCM, washed with H_2O and brine, dried over Mg_2SO_4, filtered, and concentrated. The residue was purified by flash column chromatography (SiO$_2$, 5-10% EtOAc in petroleum ether) to deliver α-hydroxyketone **12** (12 mg, 78%) as a white solid.

^{1}H NMR (500 MHz, $CDCl_3$) δ 8.33 (s, 1H), 7.96 – 7.83 (m, 4H), 7.60 (t, *J* = 7.5 Hz, 1H), 7.55 (t, *J* = 7.5 Hz, 1H), 3.51 (s, 1H), 3.18 (dd, *J* = 14.1, 4.5 Hz, 1H), 2.93 – 2.79 (m, 3H), 2.58 – 2.52 (m, 1H), 2.48 (dd, *J* = 11.9, 6.0 Hz, 1H), 2.33 – 2.17 (m, 3H), 1.64 (s, 3H).
^{13}C NMR (125 MHz, $CDCl_3$) δ 212.9, 203.4, 135.5, 132.6, 131.2, 130.9, 129.8, 128.7, 128.6, 127.9, 126.9, 124.9, 80.0, 51.8, 47.1, 43.8, 35.3, 34.4, 28.9, 24.8.
ATR-IR ν (cm-1): 3474 (br), 2932 (m), 1704 (s), 1664 (s), 1281 (m), 1121 (m), 762 (m).
HRMS (ESI): Calcd for $C_{20}H_{21}O3^+$(M+H$^+$): 309.1491, Found: 309.1493.

Late-stage modification of natural products:

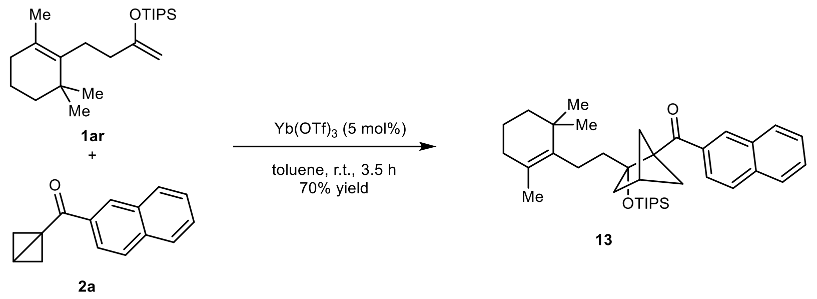

Naphthalen-2-yl(2-((triisopropylsilyl)oxy)-2-(2-(2,6,6-trimethylcyclohex-1-en-1-yl)ethyl)bicyclo[2.1.1]hexan-1-yl)methanone (13): A 1-dram screw-cap vial with a stir bar was charged with silyl enol ether **1ar** (0.2 mmol, 70.1 mg), BCB **2a** (0.26 mmol, 54.1 mg) and $Yb(OTf)_3$ (0.01 mmol, 6.2 mg). Then the vial was sealed with a septum, evacuated and backfilled with N_2 (× 3). Under N_2 atmosphere, dry toluene (2 mL) was added rapidly. The septum was quickly replaced with a screw cap. The reaction was stirred for 3.5 h until silyl enol ether was completely consumed. The reaction mixture was filtered through a pad of silica-gel and washed with DCM. The filtrate was concentrated on rotary evaporator and the residue was directly purified by silica-gel flash column chromatography (0 – 10% ethyl acetate in hexane) afforded the product **10** (78 mg, 70% yield) as a colorless oil.
^{1}H NMR (500 MHz, $CDCl_3$) δ 8.46 (d, *J* = 1.6 Hz, 1H), 8.02 (dd, *J* = 8.5, 1.7 Hz, 1H), 7.89 (d, *J* = 8.1 Hz, 1H), 7.82 (dd, *J* = 11.9, 8.3 Hz, 2H), 7.61 – 7.47 (m, 2H), 2.78 (dd, *J* = 9.8, 6.2 Hz, 1H), 2.48 (td, *J* = 12.6, 12.1, 4.4 Hz, 1H), 2.42 (t, *J* = 3.2 Hz, 1H), 2.34 (dd, *J* = 11.8, 2.3 Hz, 1H), 2.24 – 2.17 (m, 1H), 2.13 – 2.07 (m, 2H), 2.03 (td, *J* = 13.0, 4.3 Hz, 1H), 1.99 – 1.95 (m, 1H), 1.94 – 1.84 (m, 4H), 1.63 (s, 3H), 1.58 – 1.52 (m, 2H), 1.44 – 1.38 (m, 2H), 1.04 (s, 3H), 1.01 (s, 3H), 0.98 – 0.88 (m, 21H).
^{13}C NMR (125 MHz, $CDCl_3$) (one peak of aromatic carbon was missing) δ 203.8, 137.5, 136.3, 135.3, 132.5, 131.4, 129.5, 128.0, 127.8, 127.3, 126.4, 125.3, 85.8, 69.1, 44.8, 43.7, 43.4, 41.3, 40.4, 35.2, 33.9, 33.1, 28.9, 28.9, 26.4, 20.2, 19.7, 18.6, 14.0.
ATR-IR ν (cm-1): 2942 (s), 2865 (s), 1651 (s), 1464 (s), 1309 (s), 1121 (s), 1031 (s), 883 (s).
HRMS (ESI): Calcd for $C_{37}H_{55}O_2Si^+$ (M+H$^+$): 559.3972, Found: 559.3990.

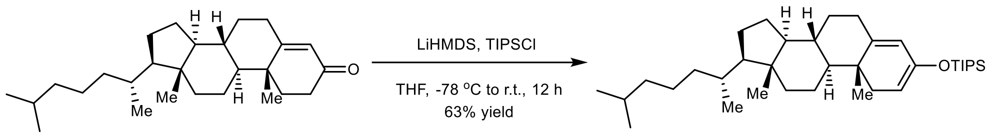

4-Cholesten-3-one **1as**

(((8S,9S,10R,13R,14S,17R)-10,13-dimethyl-17-((R)-6-methylheptan-2-yl)-6,7,8,-9,10,11,12,13,14,15,16,17-dodecahydro-1H-cyclopenta[a]phenanthren-3-yl)oxy)-triisopropylsilane (1as): To a stirred solution of 4-Cholesten-3-one (2.6 mmol, 1 g) in THF (20 mL) was slowly added LiHMDS (3.12 mmol, 1.2 eq. 3.12 mL) at -78 °C under N_2. After stirred for 5 min, TIPSCl (3.9 mmol, 1.5 eq., 0.83 mL) was added slowly at the same temperature. The resulting solution was allowed warm to room temperature. After stirred for 12 h, quenched with sat. aq. NH_4Cl. The resulting mixture was extracted with EtOAc (× 3). The combined organic layers were washed with brine, dried over Na_2SO_4, and concentrated under reduced pressure. Purification by column silica gel chromatography afforded the silyl enol ether **1as** (886 mg, 63% yield) as colorless oil. The characterization is consistent with the reported reference.[7]

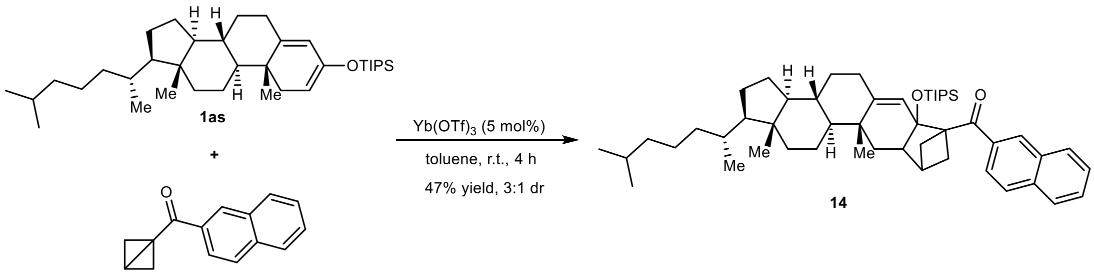

((1R,3aS,3bS,10aR,10bS,12aR)-10a,12a-dimethyl-1-((R)-6-methylheptan-2-yl)-6a-((triisopropylsilyl)oxy)-2,3,3a,3b,4,5,6a,8,9,9a,10,10a,10b,11,12,12a-hexadeca-hydro-7,9-methanodicyclopenta[a,h]phenanthren-7(1H)-yl)(naphthalen-2-yl)-methanone (14): A 1-dram screw-cap vial with a stir bar was charged with silyl enol ether **1as** (0.3 mmol, 162.3 mg), BCB **2a** (0.2 mmol, 41.6 mg) and $Yb(OTf)_3$ (0.01 mmol, 6.2 mg). Then the vial was sealed with a septum, evacuated and backfilled with N_2 (× 3). Under N_2 atmosphere, dry toluene (2 mL) was added rapidly. The septum was quickly replaced with a screw cap. The reaction was stirred for 4 h until silyl enol ether was completely consumed. The reaction mixture was filtered through a pad of silica-gel and washed with DCM. The filtrate was concentrated on rotary evaporator and the residue was directly purified by silica-gel flash column chromatography (0-10% ethyl acetate in hexane) afforded the product **11** (106 mg, 47% yield, 3:1 dr) as a colorless oil.

Major isomer

^{1}H NMR (600 MHz, $CDCl_3$) δ 8.41 (d, J = 1.7 Hz, 1H), 8.01 (dd, J = 8.6, 1.7 Hz, 1H), 7.87 (dd, J = 10.1, 8.2 Hz, 2H), 7.83 (d, J = 8.6 Hz, 1H), 7.58 (ddd, J = 8.1, 6.8, 1.3 Hz, 1H), 7.53 (ddd, J = 8.1, 6.7, 1.3 Hz, 1H), 5.41 (d, J = 2.0 Hz, 1H), 2.64 (dd, J = 9.6, 6.6 Hz, 1H), 2.49 – 2.43 (m, 1H), 2.27 – 2.15 (m, 3H), 2.15 – 2.07 (m, 2H), 2.06 – 1.95 (m, 3H), 1.90 – 1.81 (m, 1H), 1.77 – 1.70 (m, 1H), 1.64 (dddd, J = 10.8,

8.1, 6.3, 3.6 Hz, 1H), 1.56 – 1.49 (m, 2H), 1.47 – 1.32 (m, 5H), 1.27 (t, *J* = 7.2 Hz, 1H), 1.22 (dd, *J* = 14.2, 10.2 Hz, 1H), 1.18 – 0.98 (m, 9H), 1.16 (s, 3H), 0.97 – 0.83 (m, 31H), 0.70 (s, 3H).

^{13}C NMR (150 MHz, CDCl$_3$) δ 201.3, 148.1, 135.5, 135.4, 132.4, 132.1, 129.6, 128.3, 127.9, 127.8, 126.6, 125.3, 122.8, 80.6, 68.0, 56.4, 56.4, 55.2, 46.6, 43.4, 43.0, 42.5, 41.2, 40.1, 39.9, 39.7, 36.5, 36.3, 36.3, 36.0, 33.3, 32.5, 28.4, 28.2, 24.4, 24.0, 23.0, 22.7, 21.3, 20.0, 18.8, 18.7, 18.5, 14.1, 12.1.

ATR-IR ν (cm-1): 2939 (s), 2865 (m), 1655 (m), 1464 (m), 1308 (m), 1116 (m), 907 (m), 732 (s), 677 (m).

HRMS (ESI): Calcd for $C_{51}H_{77}O_2Si^+$ (M+H$^+$): 749.5693, Found: 749.5686.

Minor isomer

^{1}H NMR (600 MHz, CDCl$_3$) δ 8.50 (s, 1H), 8.04 (dd, *J* = 8.6, 1.6 Hz, 1H), 7.88 (d, *J* = 8.1 Hz, 1H), 7.85 (d, *J* = 8.1 Hz, 1H), 7.81 (d, *J* = 8.6 Hz, 1H), 7.57 (t, *J* = 7.8 Hz, 1H), 7.51 (t, *J* = 7.7 Hz, 1H), 5.47 (s, 1H), 2.53 – 2.41 (m, 3H), 2.20 – 2.16 (m, 3H), 2.13 – 2.08 (m, 2H), 2.05 – 1.98 (m, 1H), 1.91 (dd, *J* = 14.8, 8.5 Hz, 1H), 1.85 – 1.76 (m, 3H), 1.72 (dd, *J* = 11.2, 4.6 Hz, 1H), 1.54 – 1.44 (m, 4H), 1.37 – 1.22 (m, 4H), 1.18 – 1.05 (m, 6H), 1.15 (s, 3H), 0.98 – 0.77 (m, 34H), 0.71 (s, 3H).

^{13}C NMR (150 MHz, CDCl$_3$) δ 202.1, 147.2, 135.6, 135.3, 132.4, 132.2, 129.7, 128.2, 127.8, 127.7, 126.4, 125.4, 123.7, 80.7, 67.2, 56.7, 56.6, 56.3, 49.7, 45.0, 43.1, 42.7, 40.3, 39.9, 39.7, 38.6, 36.3, 36.2, 35.9, 35.4, 33.4, 32.8, 28.4, 28.2, 24.4, 23.9, 23.0, 22.7, 21.8, 21.5, 18.8, 18.7, 18.5, 13.5, 12.2.

ATR-IR ν (cm-1): 2944 (s), 2866 (m), 1655 (m), 1466 (m), 1258 (m), 1119 (m), 805 (m), 680 (m).

HRMS (ESI): Calcd for $C_{51}H_{77}O_2Si^+$ (M+H$^+$): 749.5693, Found: 749.5693.

2. General Procedure F Discussion

2.1 Diastereoselectivity issues

The substituent geminal to the OTIPS group is crucial for the control of diastereoselectivity in the cycloaddition of bicyclo[1.1.0]butanes and alkenes. The reaction with silyl enol ether **1ao** (Z/E = 10:1) would deliver **3ao** in high diasteroselectivity (~13:1 dr) (Supplementary Figures 1a, 2 and 3). However, the reaction with silyl enol ether **1ap** (Z/E = 7.4:1) would afford a mixture of cycloadducts **3ap** with 1.3:1 dr (Supplementary Figures 1b, 4 and 5). These experiments demonstrate that the R group of silyl enol ether **1** is critical for the control of diastereoselectivity, which arises from the differences in substituents adjacent to the oxocarbenium ion of **Int 5**.

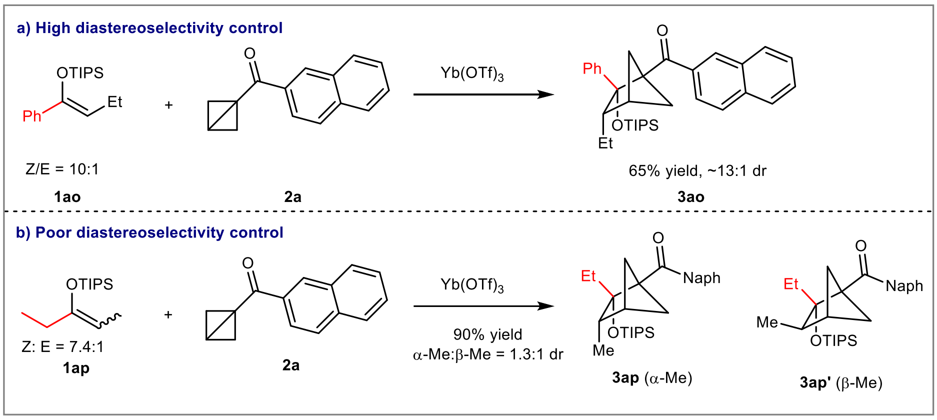

Supplementary Figure 4. Formal cycloaddition of trisubstituted silyl dienol ethers and BCBs. **a** Reaction with high diastereoselectivity. **b** Reaction with poor diastereoselectivity

^{1}H NMR (600 MHz, CDCl3)

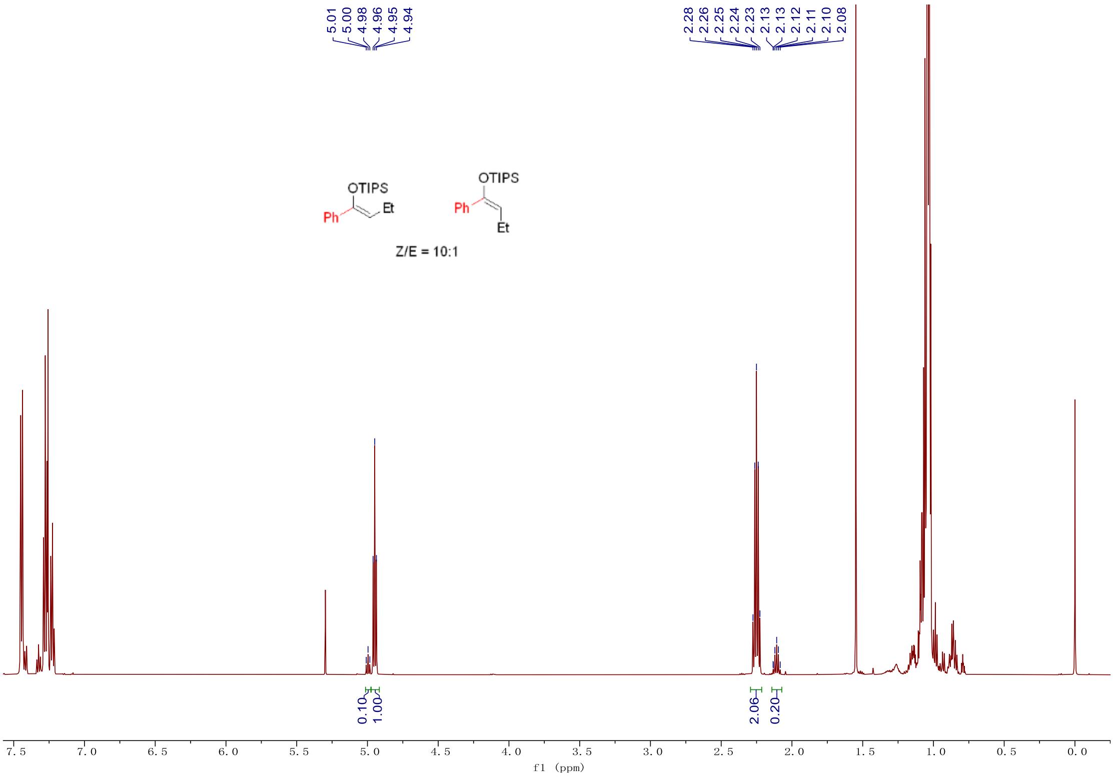

Supplementary Figure 5. ^{1}H NMR spectrum of **1ao**

^{1}H NMR (500 MHz, CDCl3)

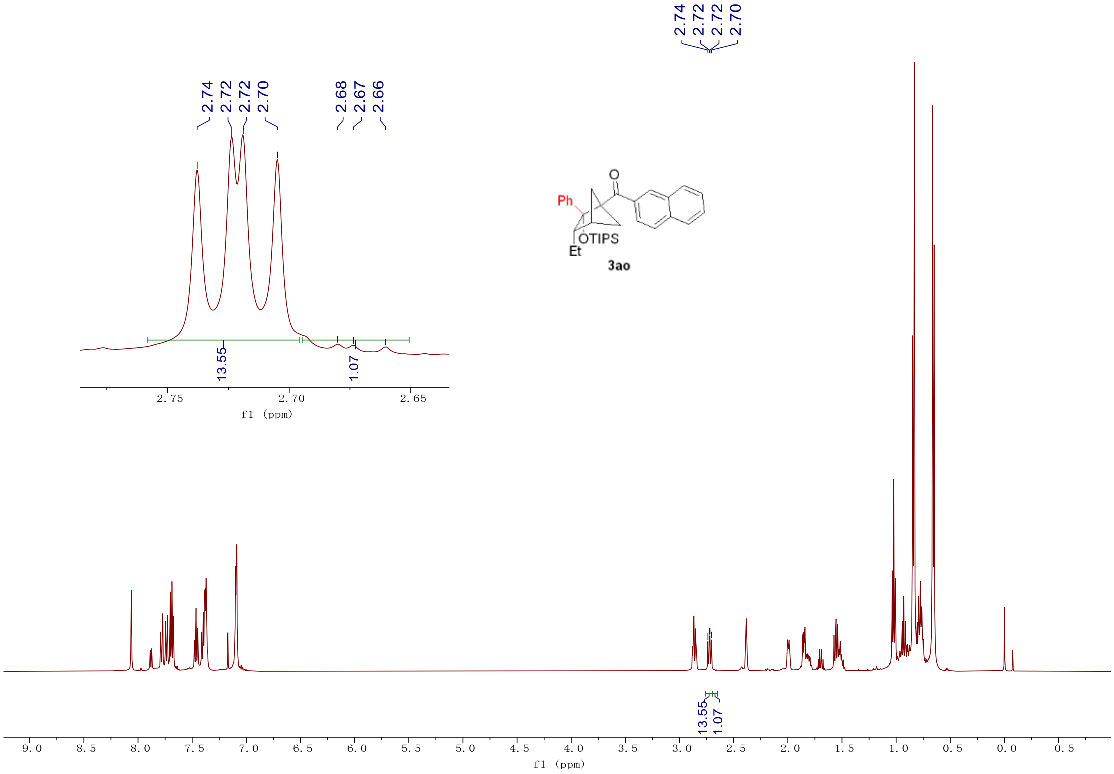

Supplementary Figure 6. ^{1}H NMR spectrum of **3ao**

¹H NMR (500 MHz, $CDCl_3$)

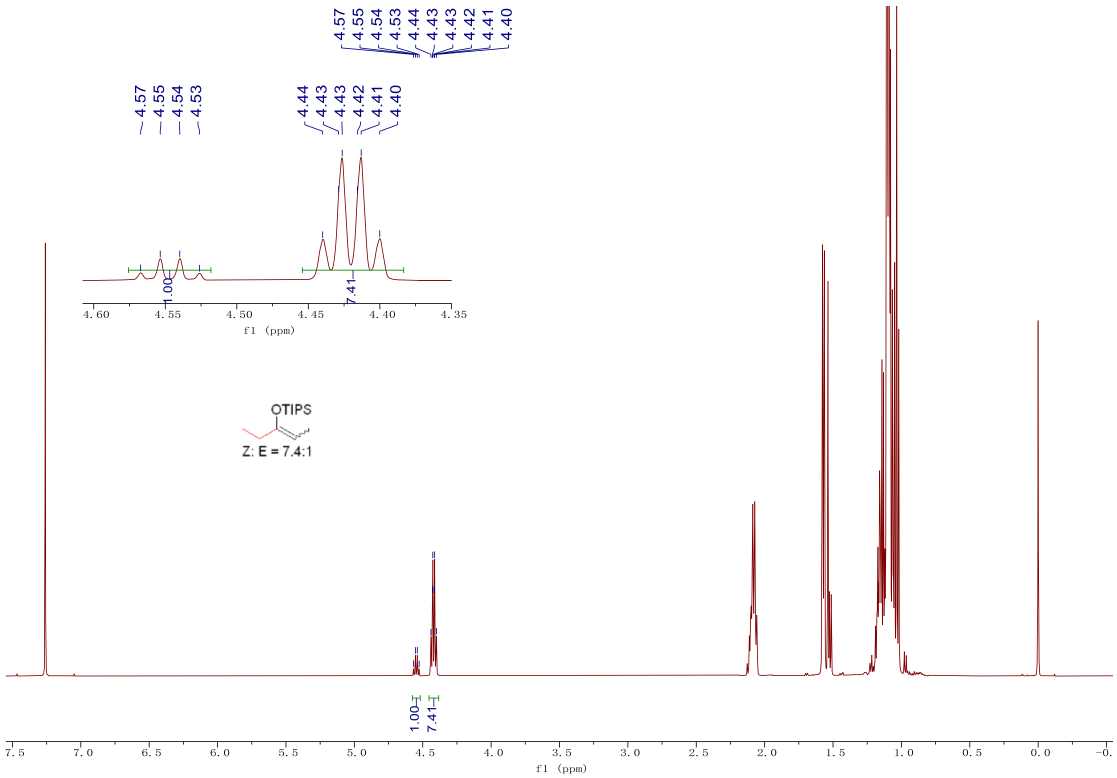

Supplementary Figure 7. ¹H NMR spectrum of **1ap**

¹H NMR (500 MHz, $CDCl_3$)

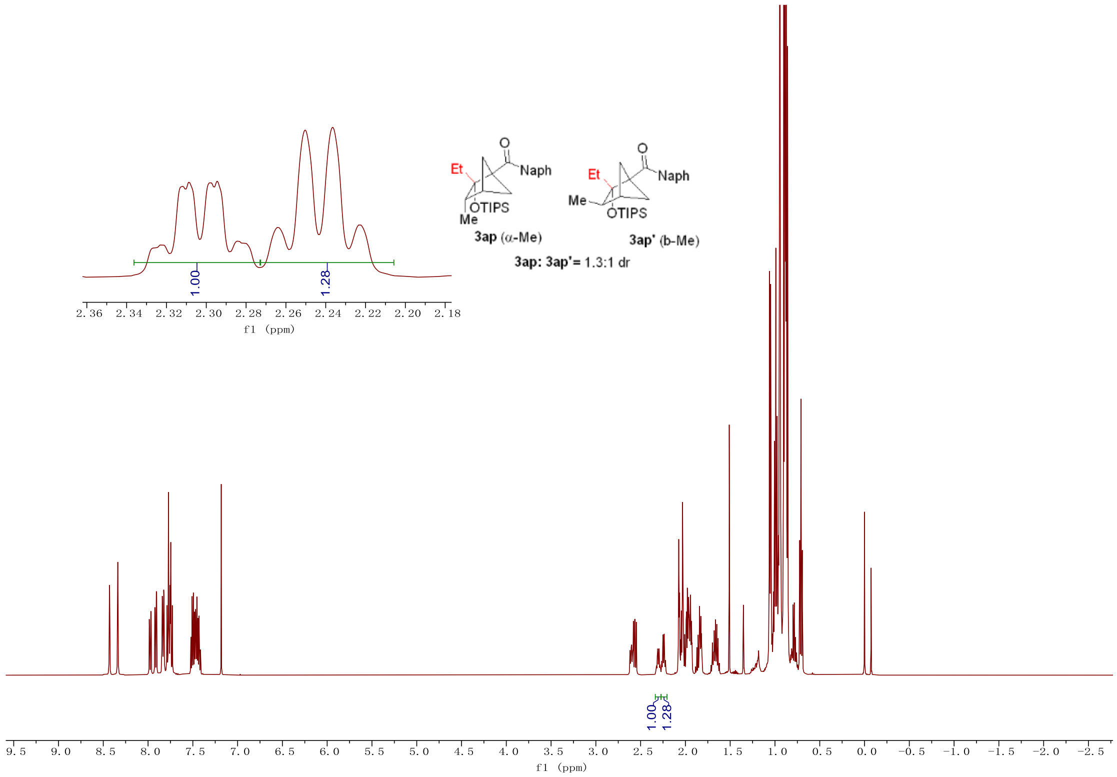

Supplementary Figure 8. ¹H NMR spectrum of **3ap**

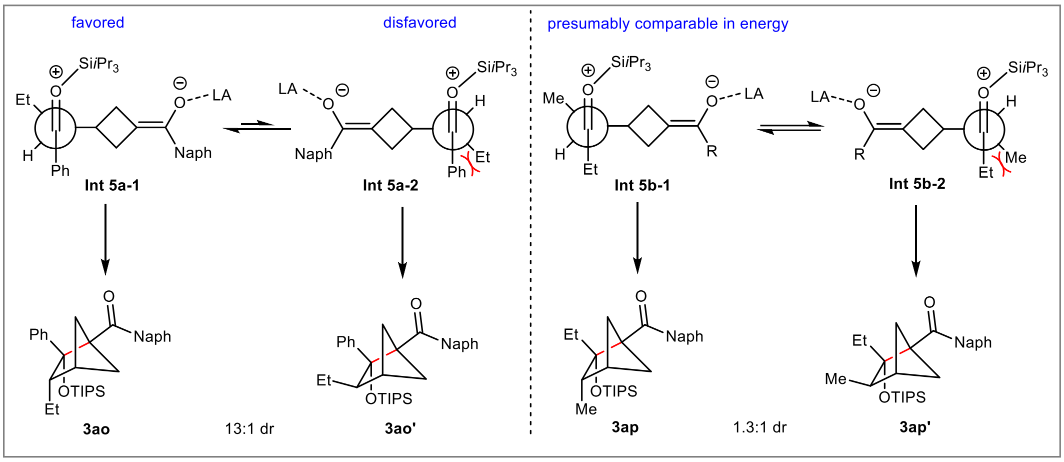

Supplementary Figure 9. Felkin-Anh type model of **Int 5a** and **Int 5b**

We proposed a Felkin-Anh type model of the zwitterionic intermediate (**int 5a** and **int 5b**) to explain the diastereoselectivity (Supplementary Figure 9). In the reaction of silyl enol ether **1ao** and BCB **2a** to access **3ao**, a zwitterionic intermediate **Int 5a** was formed after the nucleophilic addition of silyl enol ether to BCB. According to the Felkin-Anh model, the large substituent of the α-carbon of carbonyl compound prefers a perpendicular orientation relative to the carbonyl group, which gave two possible conformations (**Int 5a-1** and **Int 5a-2)**. The stronger gauche interaction between the ethyl and phenyl group versus that between the hydrogen and phenyl group rendered **Int 5a-1** a more favorable conformation. **Int 5a-1** led to a high diastereoselectivity in favor of diastereomer **3ao**.

In the reaction of silyl enol ether **1ap** and BCB **2a** to access **3ap**, a zwitterionic intermediate **int 5b** was formed after the nucleophilic addition of silyl enol ether to BCB. The steric interaction of methyl and ethyl group is not as pronounced, possibly resulting in low diastereoselectivity.

2.2 Regioselectivity issues:

The regioselectivity of formal (3+2) and (4+3) cycloaddition with silyl dienol ether appears to be substrate-dependent. As demonstrated by the formation of (3+2) and (4+3) cycloadducts (**3u, 3al**, **3am**, **5a-5h**), the steric and electronic properties of the silyl dienol ethers have a significant influence on which pathway is favored. For example, silyl dienol ethers bearing a 3-alkyl/phenyl or 1-alkyl substituent in silyl dienol ether would favor the formation of (4+3) cycloadduct. The silyl dienol ethers with less bulky substituent such as methyl and alkenyl on the 4 position would favor the (4+3) cycloaddition (as a comparison of **3u**, **5g** and **5h**). The reaction of cyclic silyl dienol ether **1al** and BCB **2a** would provide (3+2) product **3al** exclusively. The (4+3) pathway would lead to the formation of **3al-1**, which is much more sterically congested than **3al**. Therefore, this (4+3) pathway is energetically unfavored.

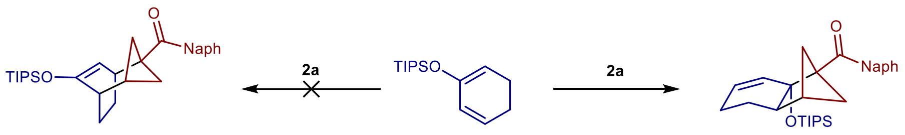

3al-1 sterically congested | **1al** | **3al** 90% yield

Supplementary Figure 10. Reaction between silyl dienol ether **1al** and BCB **2a**

2.3 ^{13}C Kinetic Isotope Effect Study.

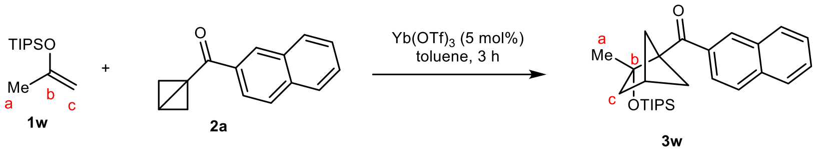

Standard sample (100% conversion based on 1w):

A 1-dram screw-cap vial with a stir bar was charged with silyl enol ether **1w** (0.5 mmol), BCB **2a** (0.5 mmol) and $Yb(OTf)_3$ (0.025 mmol, 15.5 mg). Then the vial was sealed with a septum, evacuated, and backfilled with N_2 (× 3). Under N_2 atmosphere, dry toluene (5 mL) was added rapidly. The septum was quickly replaced with a screw cap. The reaction was stirred for 3 h until BCB **2a** was completely consumed. The reaction mixture was filtered through a pad of silica-gel and washed with DCM. The filtrate was concentrated on rotary evaporator and the residue was directly purified by silica-gel flash column chromatography to afford the product **3w** (186 mg, 88% yield) as light yellow oil.

Sample prepared by excess 1w (10% conversion based on 1w):

A 1-dram screw-cap vial with a stir bar was charged with silyl enol ether **1w** (5.0 mmol), BCB **2a** (0.5 mmol). Then the vial was sealed with a septum, evacuated, and backfilled with N_2 (× 3). Under N_2 atmosphere, dry toluene (5 mL) was added rapidly and $Yb(OTf)_3$ (0.025 mmol, 15.5 mg). The septum was quickly replaced with a screw cap. The reaction was stirred for 2.5 h until BCB **2a** was completely consumed. The reaction mixture was filtered through a pad of silica-gel and washed with DCM. The filtrate was concentrated on rotary evaporator and the residue was directly purified by silica-gel flash column chromatography to afford the product **3w** (146 mg, 69% yield) as light yellow oil.

The ^{13}C NMR analyses of the standard sample and the sample prepared by excess **1w** were performed following Singleton's ^{13}C NMR method[20-22]. All of the NMR samples were prepared identically as follows: a 5 mm high precision NMR tube was charged with **3w** (100.0 mg, 0.24 mmol), then $CDCl_3$ (0.5 mL) was added. The ^{13}C NMR spectra were recorded with H-decoupling and 45 degree pulses on 500 MHz NMR spectrometer. A 30 s delay between pulses was imposed to minimize T1 variations (d1 = 30 s, at = 5.0 s, nt = 512). Polynomial fit zeroth-order baseline correction was applied. Integrations were determined by using a range of ±7 Hz around each peak. The carbon **a**, which isotopic composition can be safely assumed not to change during the reaction, was employed as an 'internal standard' for ^{13}C integrations. The average ^{13}C integration ratio of the standard sample and sample prepared by excess **1w** is shown in the following Supplementary Table 2.

Supplementary Table 2 ^{13}C integration of the samples of 3w

1st run

C#	Standard	Sample 1 (excess **1w**)	Standard/Sample 1	change (%)
a	1.000	1.000	1.000	0
b	1.009	1.010	0.999	-0.1
c	0.978	0.958	1.021	2.1

2nd run

C#	Standard	Sample 2 (excess **1w**)	Standard/Sample 2	change (%)
a	1.000	1.000	1.000	0
b	1.009	1.003	1.005	0.5
c	0.978	0.964	1.015	1.5

3rd run

C#	Standard	Sample 3 (excess **1w**)	Standard/Sample 3	change (%)
a	1.000	1.000	1.000	0
b	1.009	1.004	1.005	0.5
c	0.978	0.962	1.017	1.7

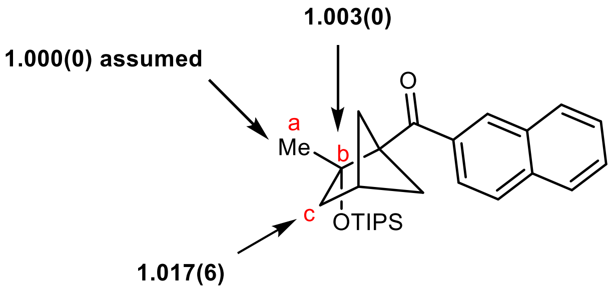

Supplementary Figure 11 ^{13}C kinetic isotope effect result of **3w**

The pronounced carbon isotope effect was only observed on the carbon **c** of the silyl enol ether **1w** when the ^{13}C integration ratio of the standard sample was compared to that prepared with excess **1w** (^{13}C(standard)/^{13}C(sample) at C(c) = 1.017, average of three runs) (Supplementary Figure 11). These results are consistent with the stepwise pathway, and the nucleophilic addition is the rate determining step.

3. X-Ray Crystallography

Supplementary Table 3 Crystal data and structure refinement of compound 3r

Crystallographic files (CDCC: 2329559). There is one B-alert, see CIF/checkCIF due to short intramolecular H-H contact between H5 and H14A. The B alert can be justified by the rigid nature of this molecular structure.

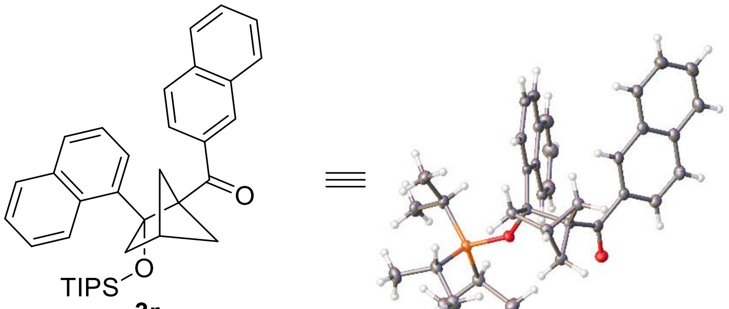

3r

(CCDC 2329559)

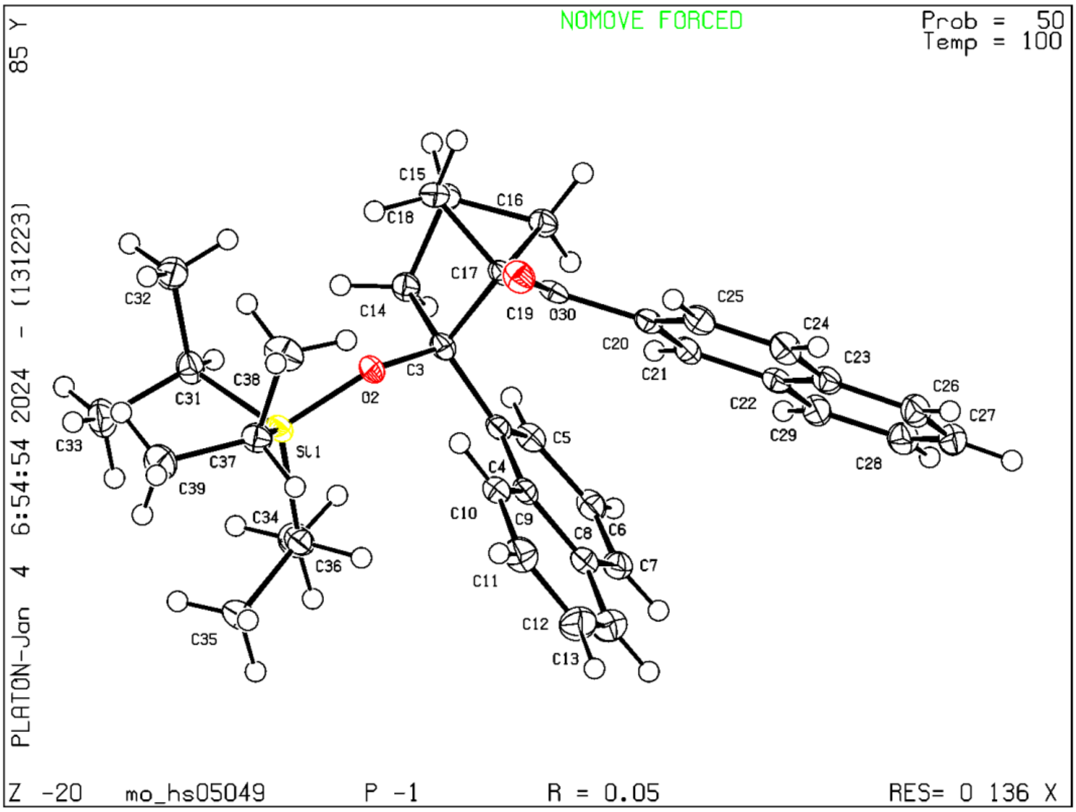

Alert level B

```
PLAT410_ALERT_2_B Short Intra H...H Contact  H5        ..H14A     .        1.88 Ang.
                                                       x,y,z  =       1_555 Check
```

Author Response: the position of hydrogen atoms has been carefully checked, this is due to the structure of this molecular

Empirical formula	$C_{36}H_{42}O_2Si$
Formula weight	534.78
Temperature/K	100.00
Crystal system	triclinic
Space group	P-1
a/Å	7.6693(3)
b/Å	11.6378(5)
c/Å	17.2328(7)
α/°	94.718(2)
β/°	98.046(2)
γ/°	105.210(2)
Volume/Å^3	1458.17(10)
Z	2
ρ_{calc}g/cm^3	1.218
μ/mm^{-1}	0.112
F(000)	576.0
Crystal size/mm^3	0.3 × 0.2 × 0.03
Radiation	MoKα (λ = 0.71073)
2Θ range for data collection/°	4.118 to 52.744
Index ranges	$-9 \leq h \leq 9$, $-14 \leq k \leq 14$, $-21 \leq l \leq 21$
Reflections collected	51402
Independent reflections	5967 [R_{int} = 0.0911, R_{sigma} = 0.0467]
Data/restraints/parameters	5967/0/358
Goodness-of-fit on F^2	1.049
Final R indexes [I>=2σ (I)]	R_1 = 0.0505, wR_2 = 0.1202
Final R indexes [all data]	R_1 = 0.0697, wR_2 = 0.1323
Largest diff. peak/hole / e Å^{-3}	0.30/-0.38

Supplementary Table 4 Crystal data and structure refinement of compound 5a

Crystallographic files (CDCC: 2358618). There are no A-alerts and B-alerts, see CIF/checkCIF).

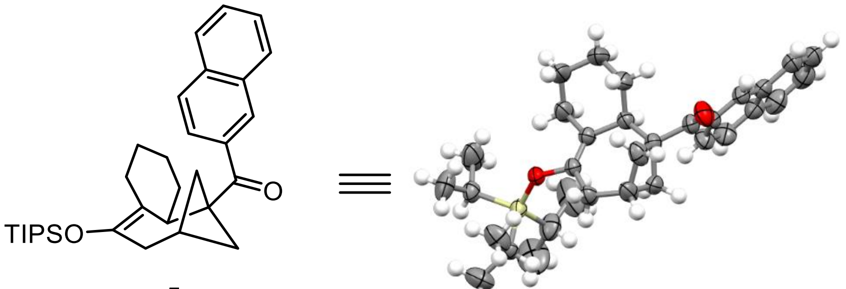

5a

(CCDC 2358618)

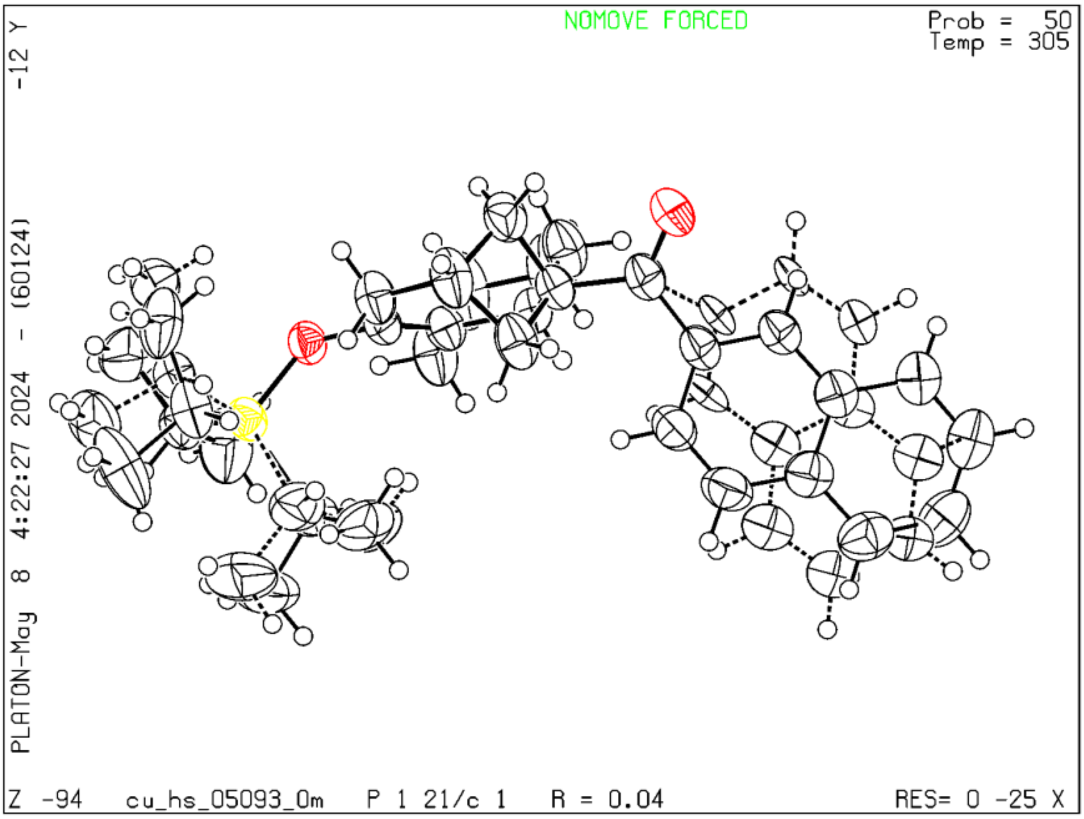

Empirical formula	$C_{32}H_{44}O_2Si$
Formula weight	488.76
Temperature/K	305.00
Crystal system	monoclinic
Space group	$P2_1/c$
a/Å	7.3861(4)
b/Å	11.2687(5)
c/Å	34.5299(14)
α/°	90
β/°	92.882(3)
γ/°	90

Volume/Å^3	2870.3(2)
Z	4
ρ_{calc}g/cm^3	1.131
μ/mm^{-1}	0.904
F(000)	1064.0
Crystal size/mm^3	0.1 × 0.1 × 0.1
Radiation	CuKα (λ = 1.54178)
2Θ range for data collection/°	5.124 to 136.774
Index ranges	-8 ≤ h ≤ 8, -13 ≤ k ≤ 13, -41 ≤ l ≤ 41
Reflections collected	33027
Independent reflections	5256 [R_{int} = 0.0437, R_{sigma} = 0.0306]
Data/restraints/parameters	5256/179/473
Goodness-of-fit on F^2	1.037
Final R indexes [I>=2σ (I)]	R_1 = 0.0430, wR_2 = 0.1222
Final R indexes [all data]	R_1 = 0.0499, wR_2 = 0.1287
Largest diff. peak/hole / e Å^{-3}	0.22/-0.24

4. Supplementary NMR Spectra

Compound 1m

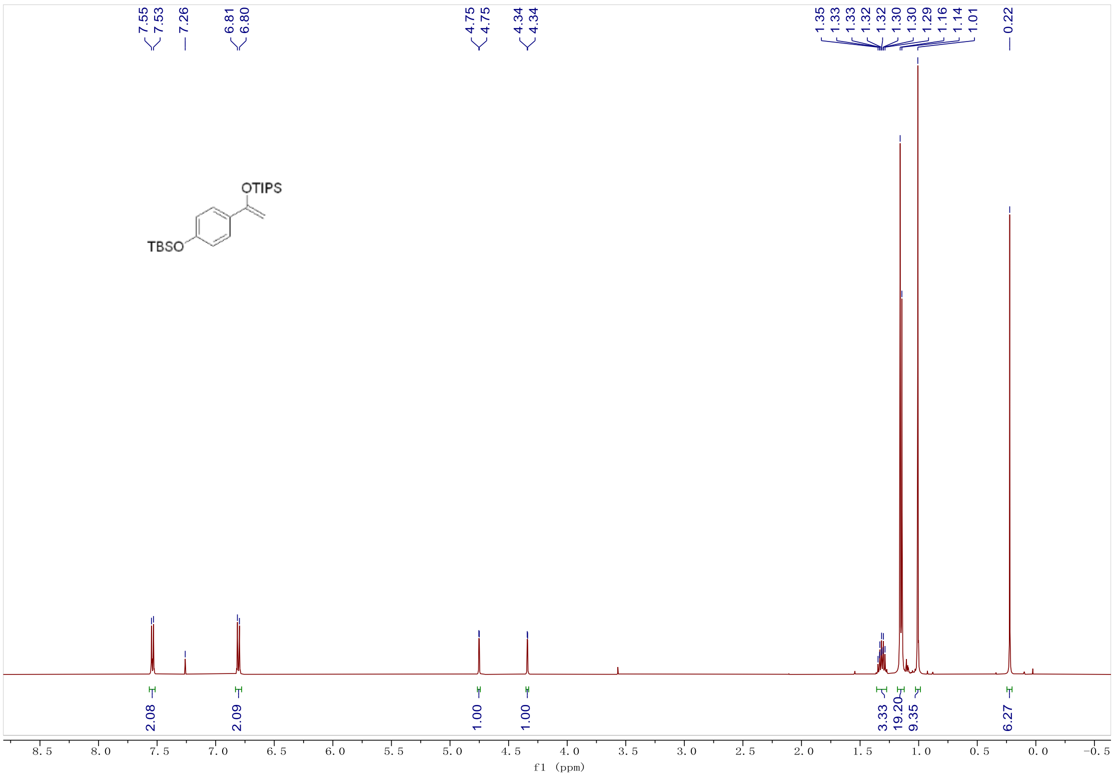

Supplementary Figure 12. **^{1}H NMR** spectrum (500 MHz, $CDCl_3$) of **1m**

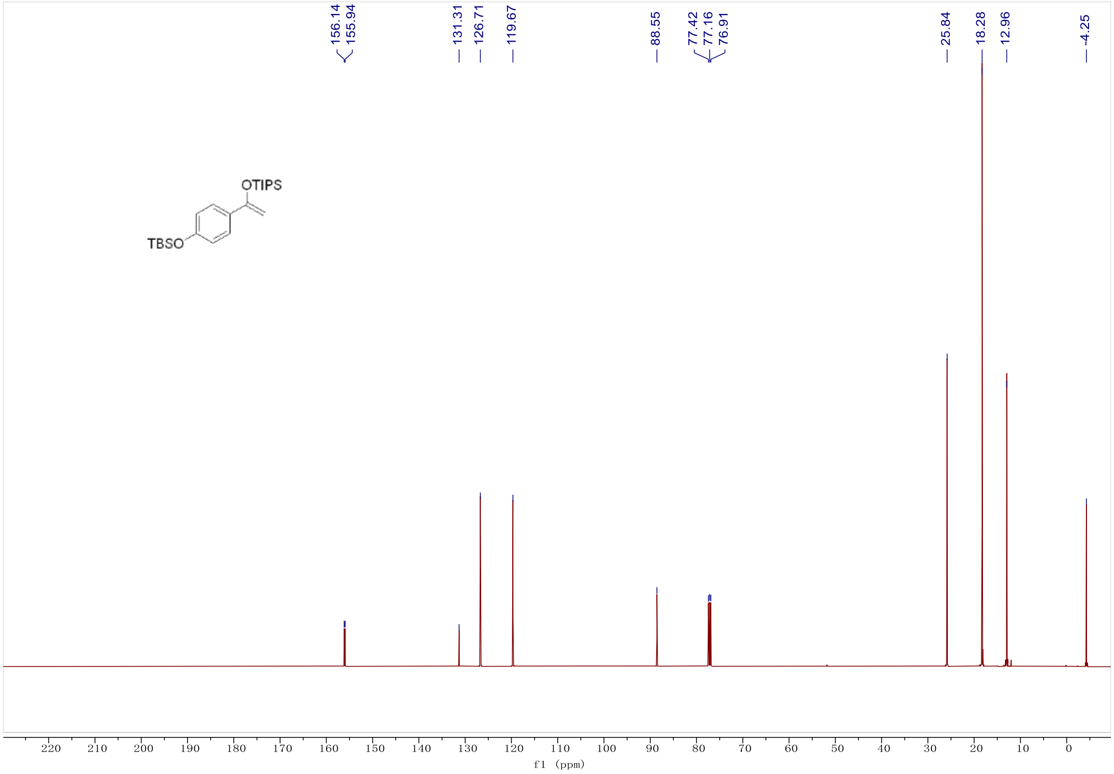

Supplementary Figure 13. **^{13}C NMR** spectrum (125 MHz, $CDCl_3$) of **1m**

Compound 1n

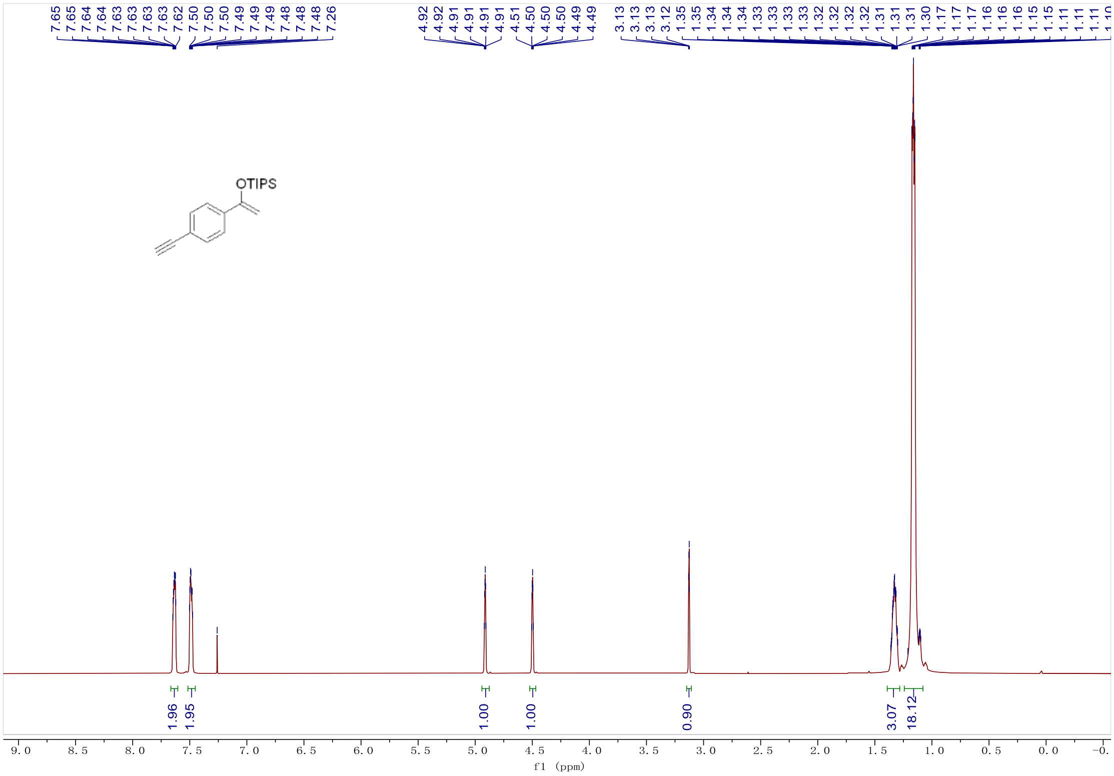

Supplementary Figure 14. ^{1}H NMR spectrum (600 MHz, $CDCl_3$) of **1n**

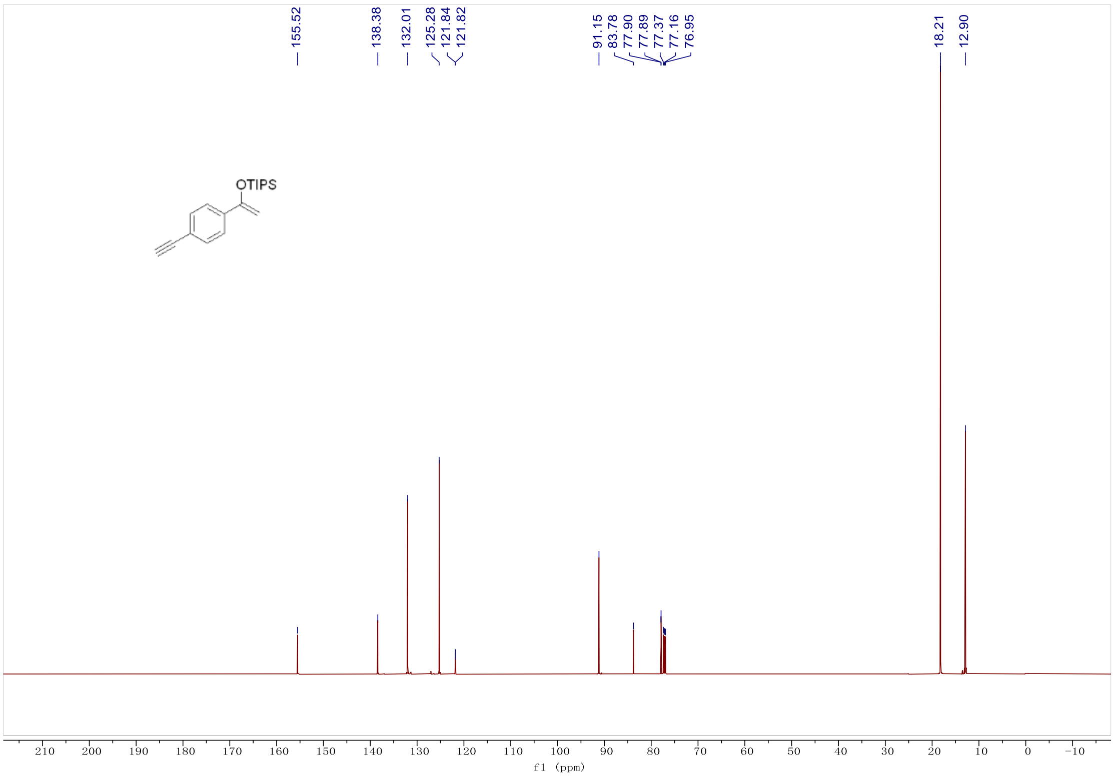

Supplementary Figure 15. ^{13}C NMR spectrum (150 MHz, $CDCl_3$) of **1n**

Compound 1p

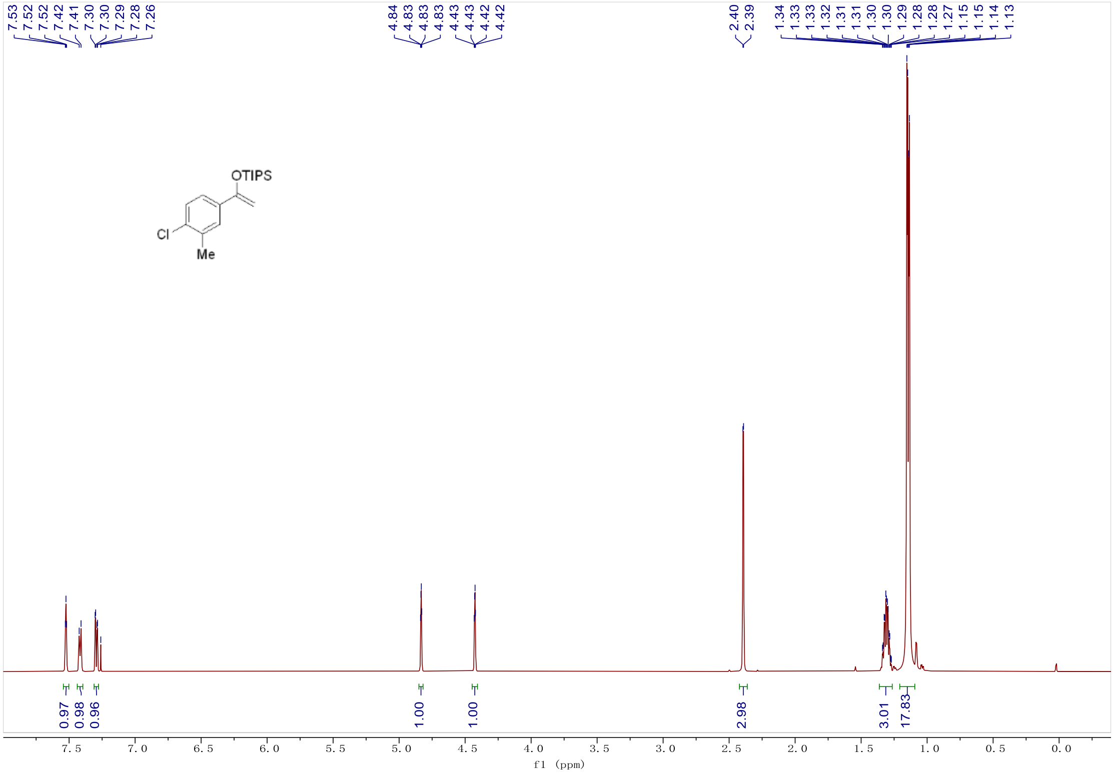

Supplementary Figure 16. **^{1}H NMR** spectrum (600 MHz, $CDCl_3$) of **1p**

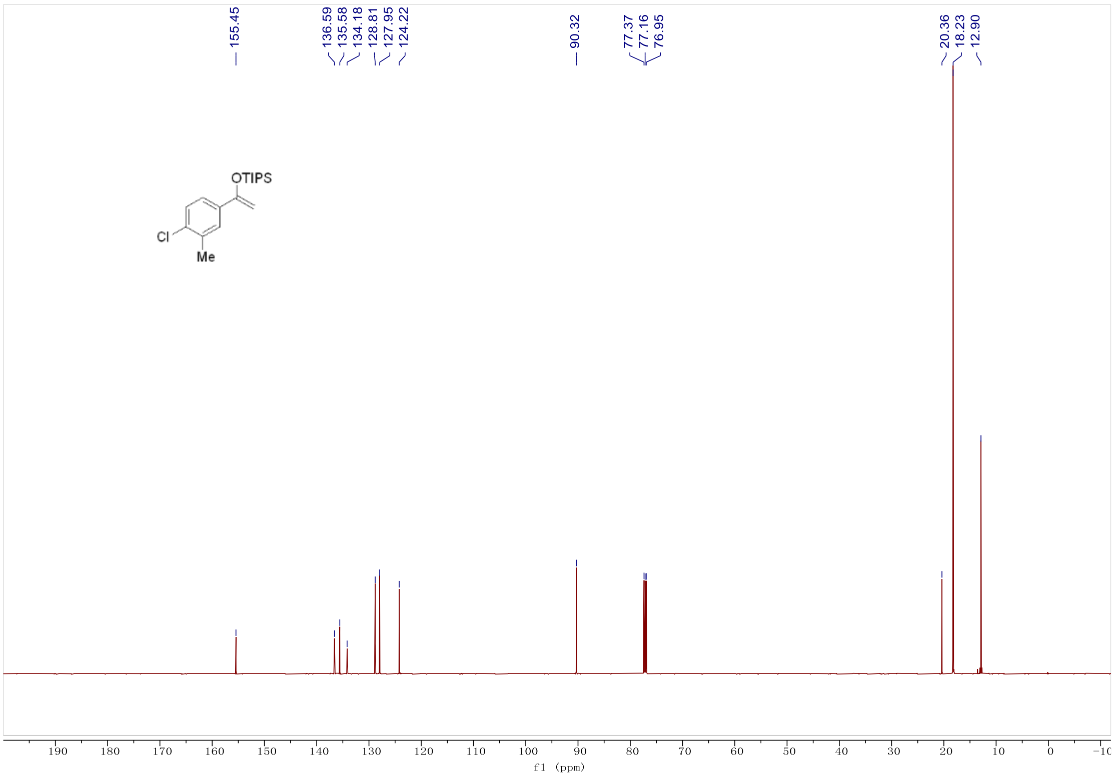

Supplementary Figure 17. **^{13}C NMR** spectrum (150 MHz, $CDCl_3$) of **1p**

Compound 1v

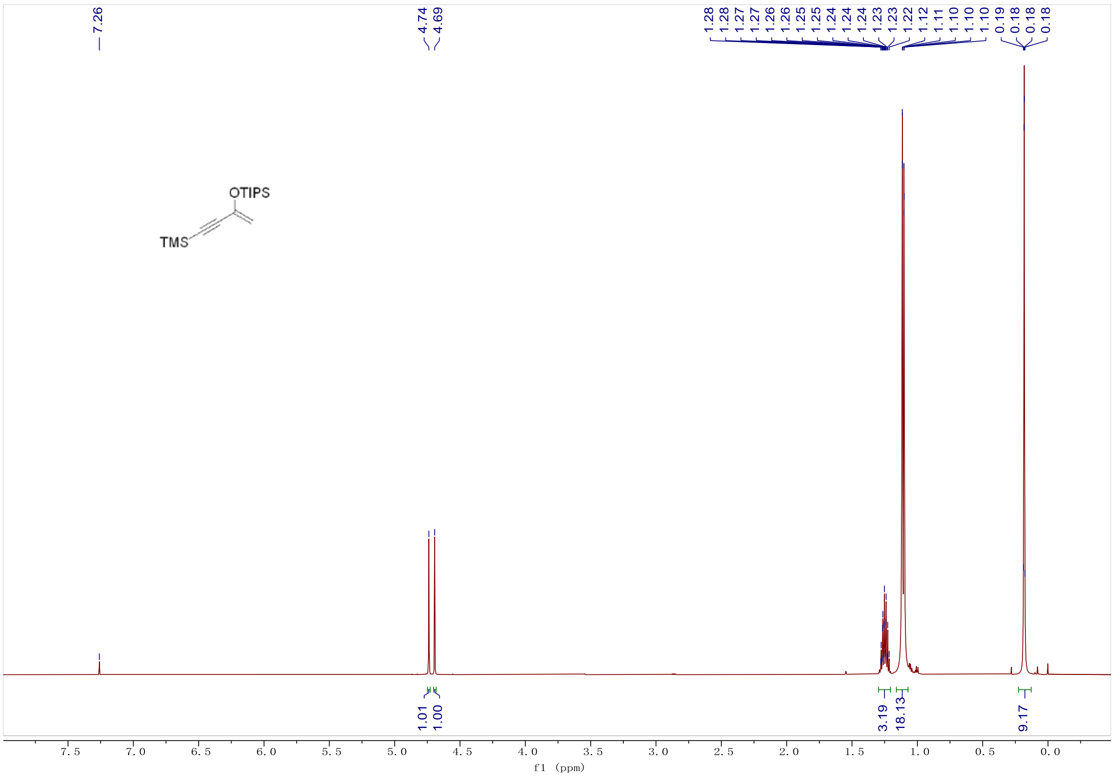

Supplementary Figure 18. ^{1}**H NMR** spectrum (600 MHz, $CDCl_3$) of **1v**

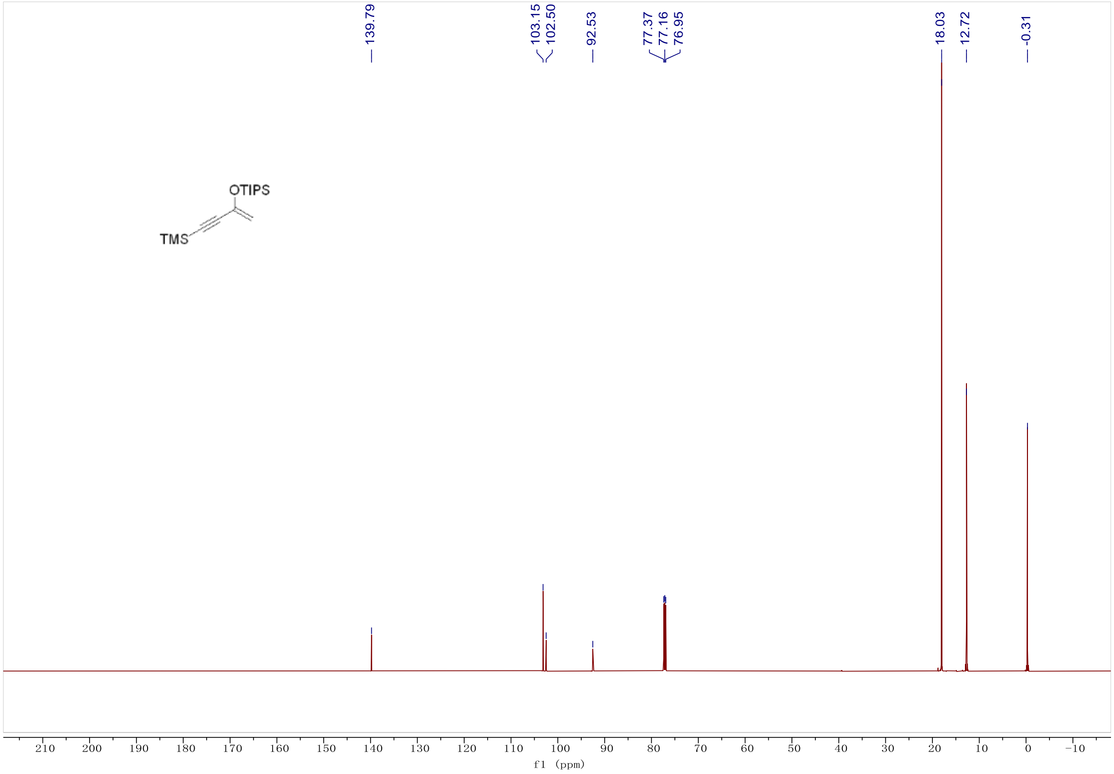

Supplementary Figure 19. ^{13}**C NMR** spectrum (150 MHz, $CDCl_3$) of **1v**

Compound 1x

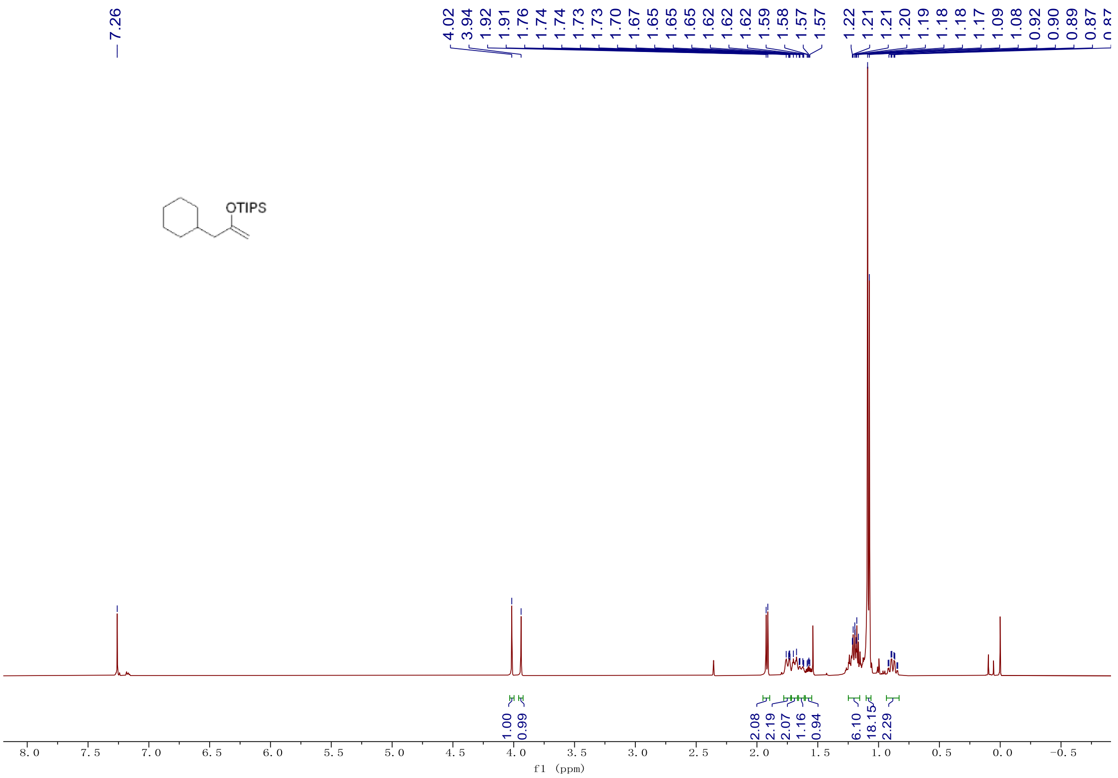

Supplementary Figure 20. ^{1}**H NMR** spectrum (500 MHz, $CDCl_3$) of **1x**

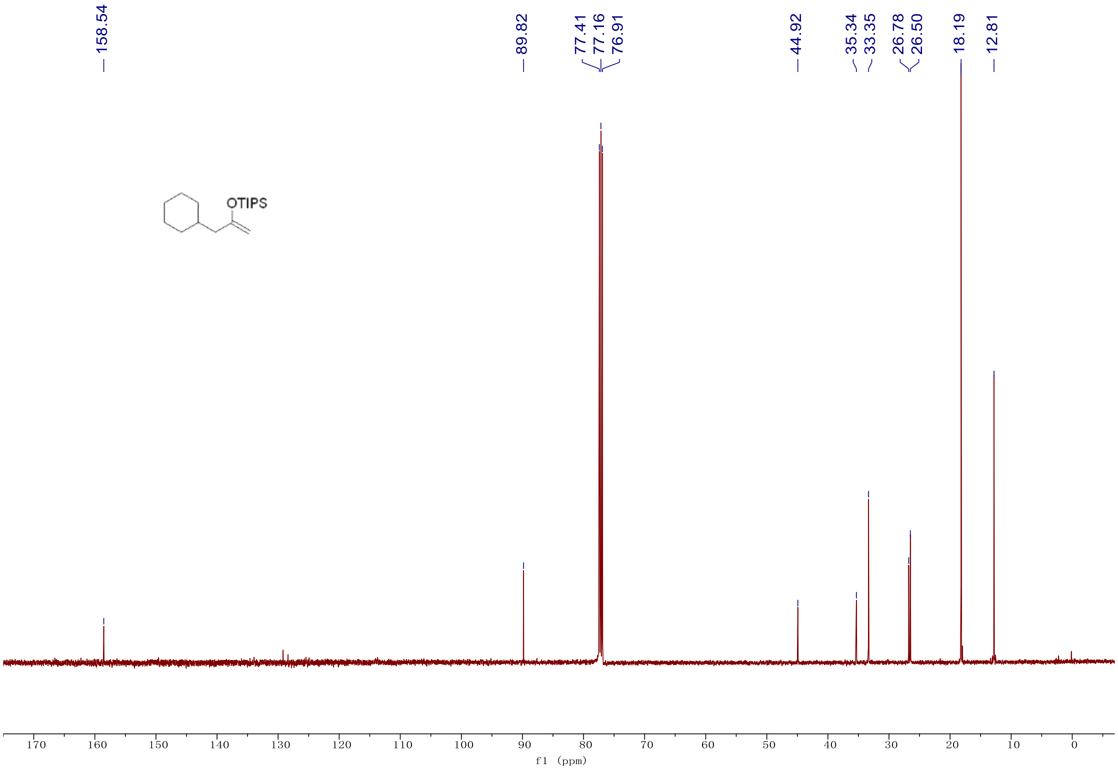

Supplementary Figure 21. ^{13}**C NMR** spectrum (125 MHz, $CDCl_3$) of **1x**

Compound 1y

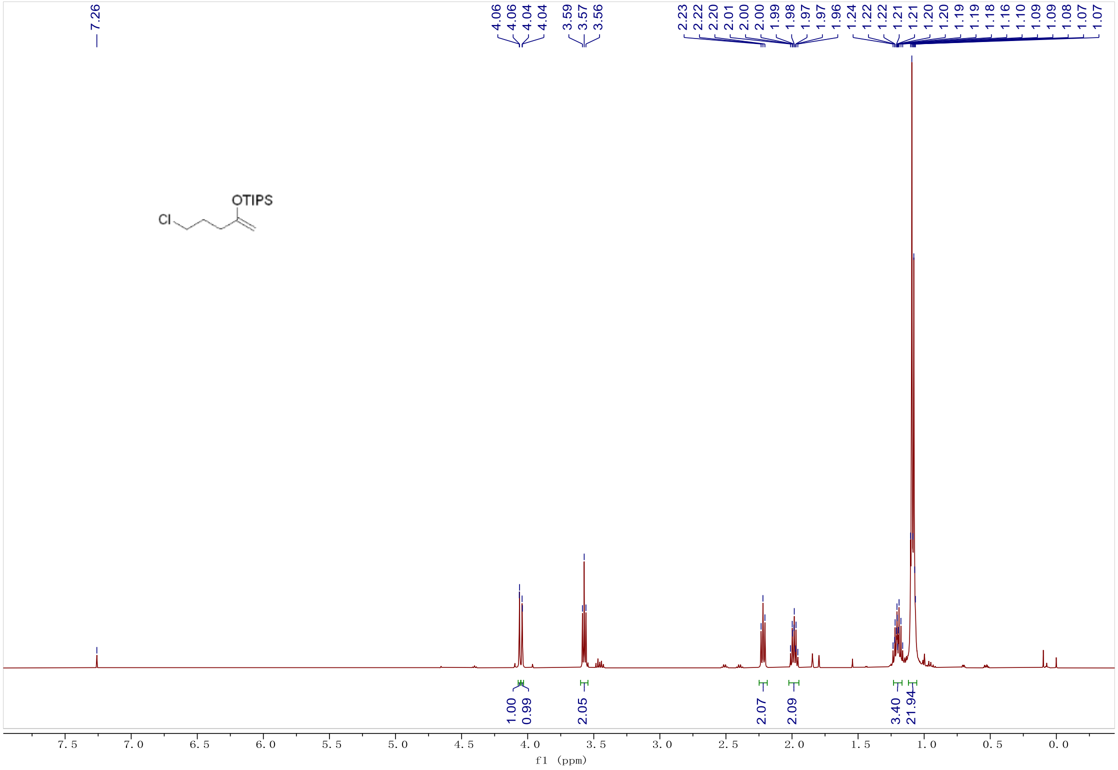

Supplementary Figure 22. ^{1}H NMR spectrum (500 MHz, $CDCl_3$) of **1y**

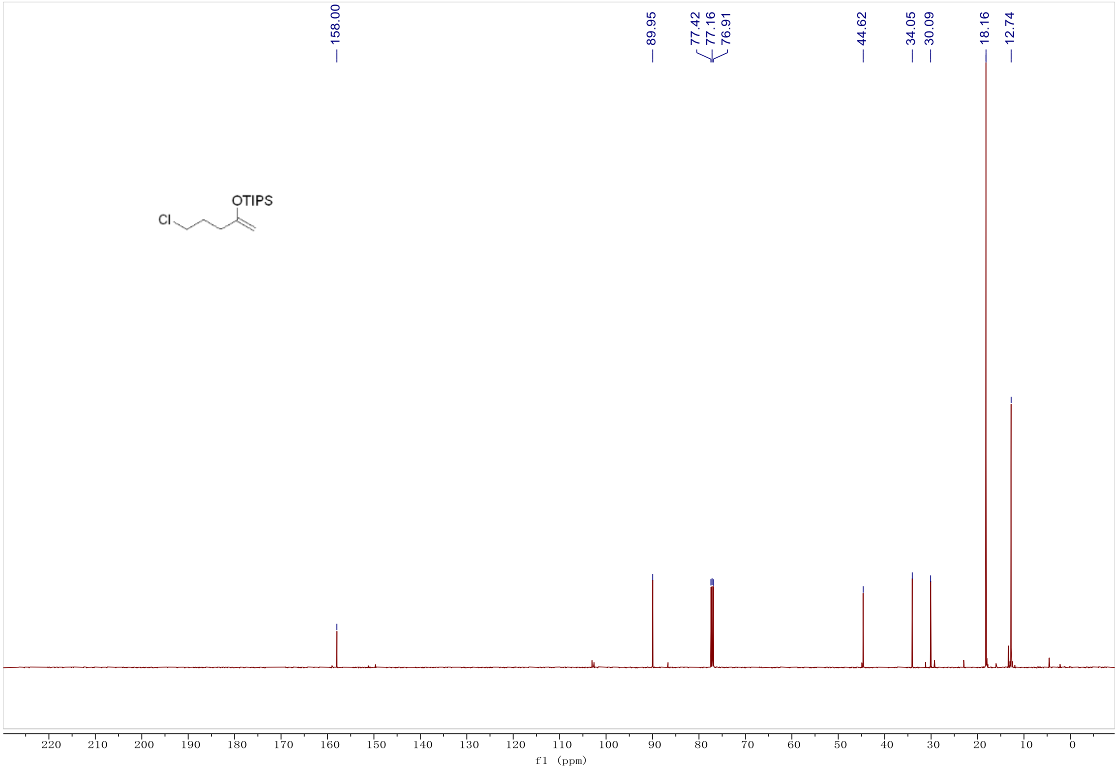

Supplementary Figure 23. ^{13}C NMR spectrum (125 MHz, $CDCl_3$) of **1y**

Compound 1z

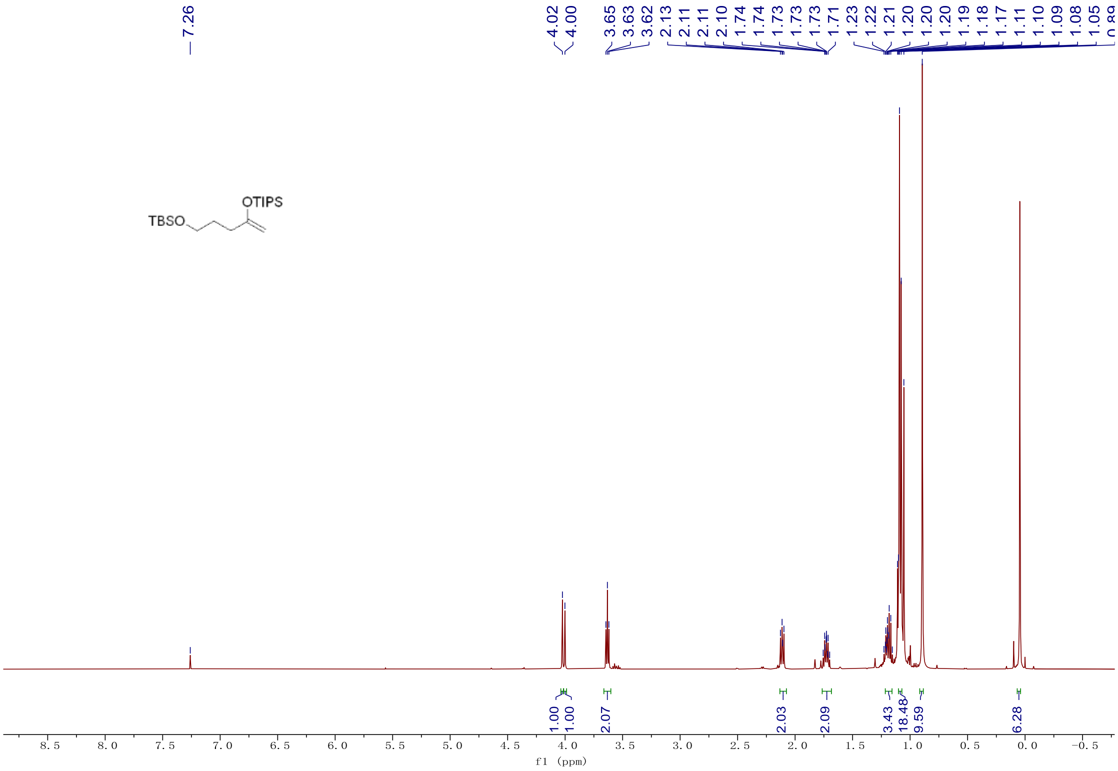

Supplementary Figure 24. ^{1}H NMR spectrum (500 MHz, $CDCl_3$) of **1z**

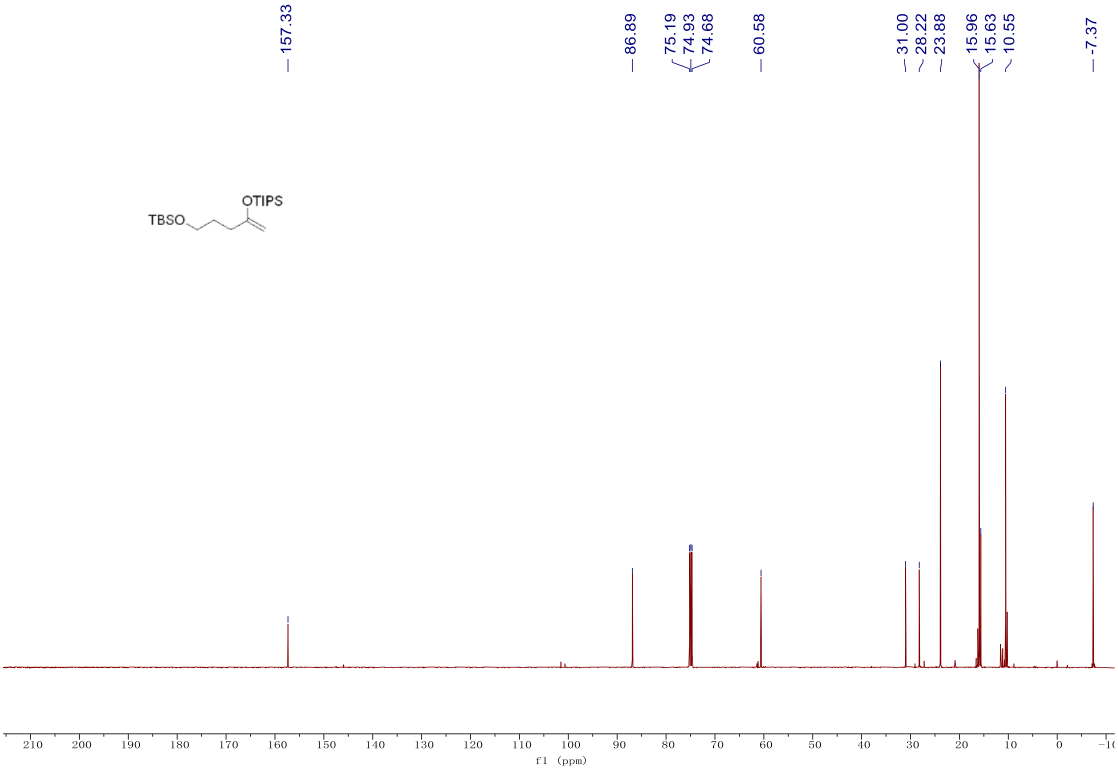

Supplementary Figure 25. ^{13}C NMR spectrum (125 MHz, $CDCl_3$) of **1z**

Compound 1aa

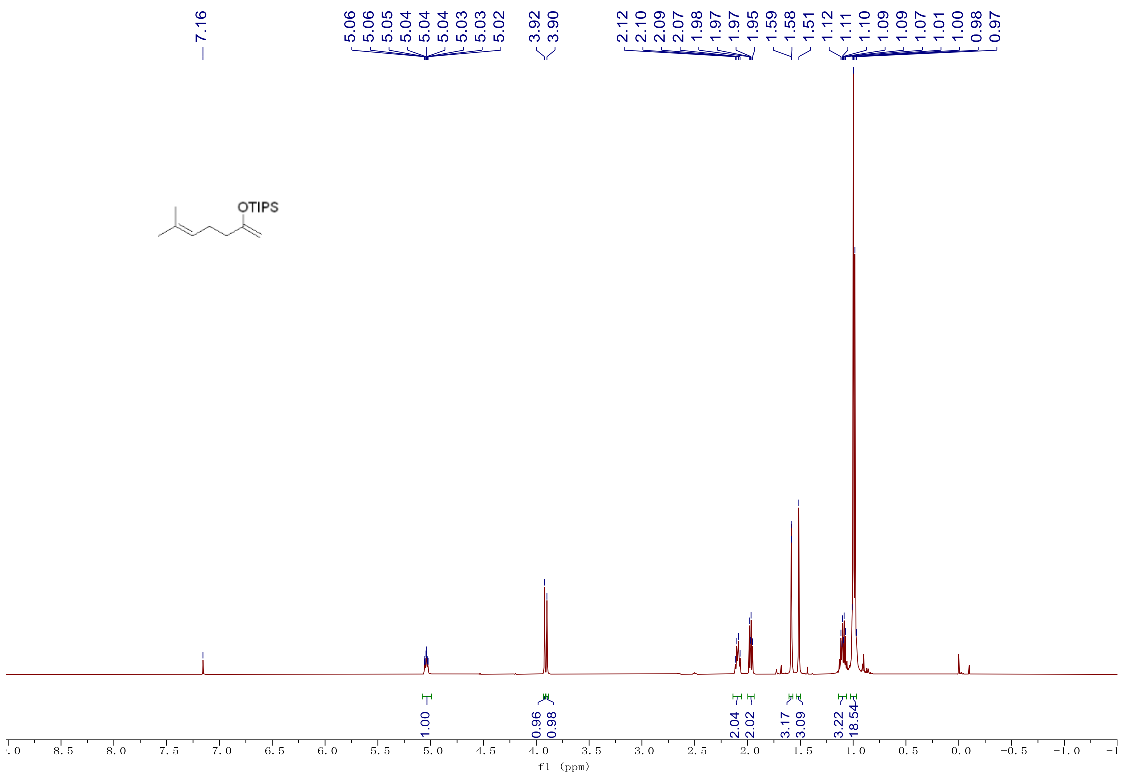

Supplementary Figure 26. **^{1}H NMR** spectrum (500 MHz, $CDCl_3$) of **1aa**

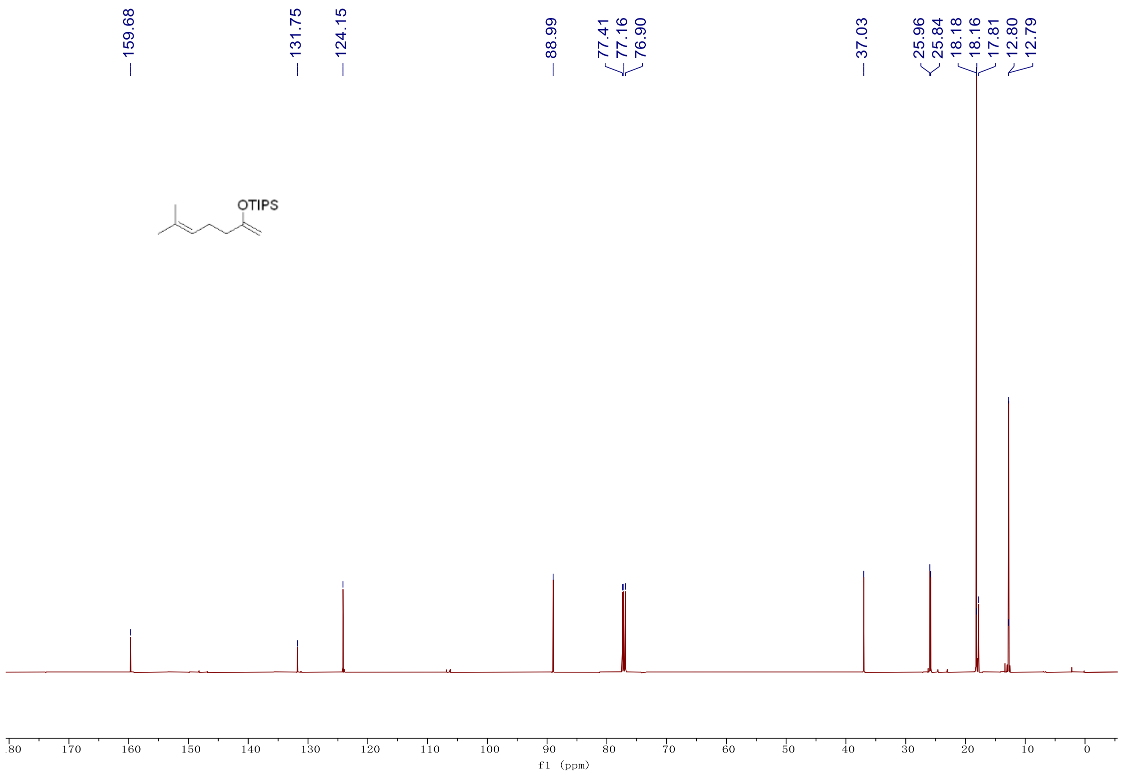

Supplementary Figure 27. **^{13}C NMR** spectrum (125 MHz, $CDCl_3$) of **1aa**

Compound 1ab

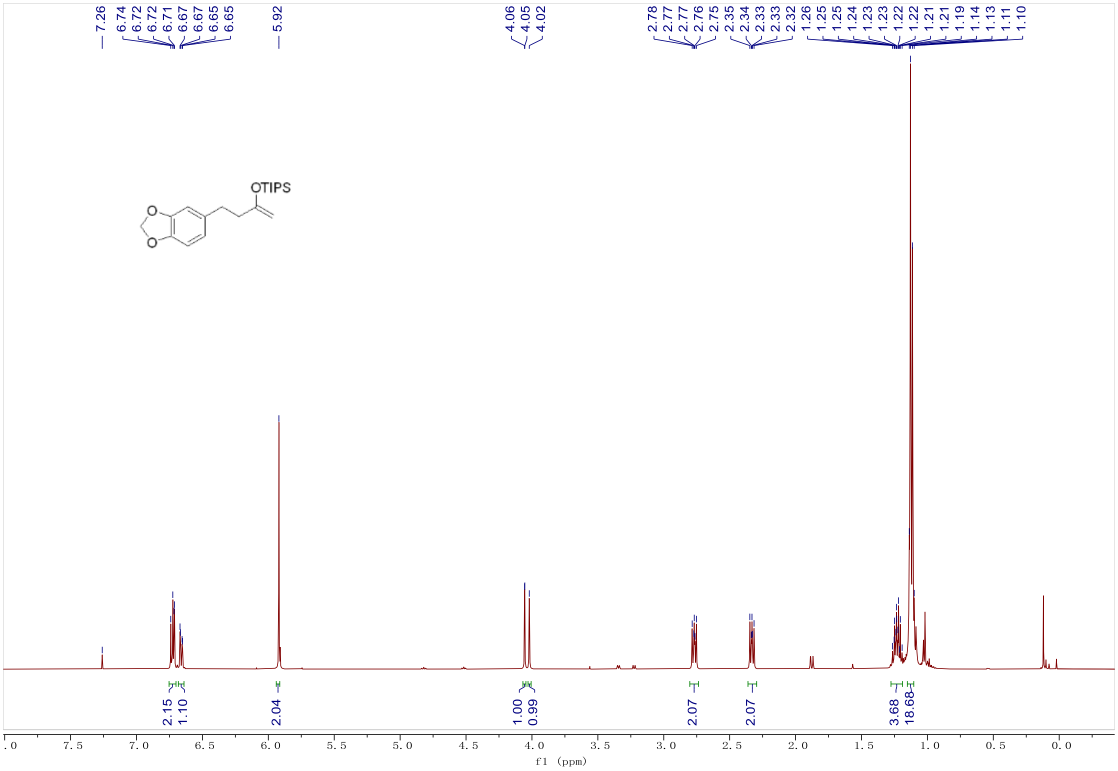

Supplementary Figure 28. ^{1}H NMR spectrum (500 MHz, $CDCl_3$) of **1ab**

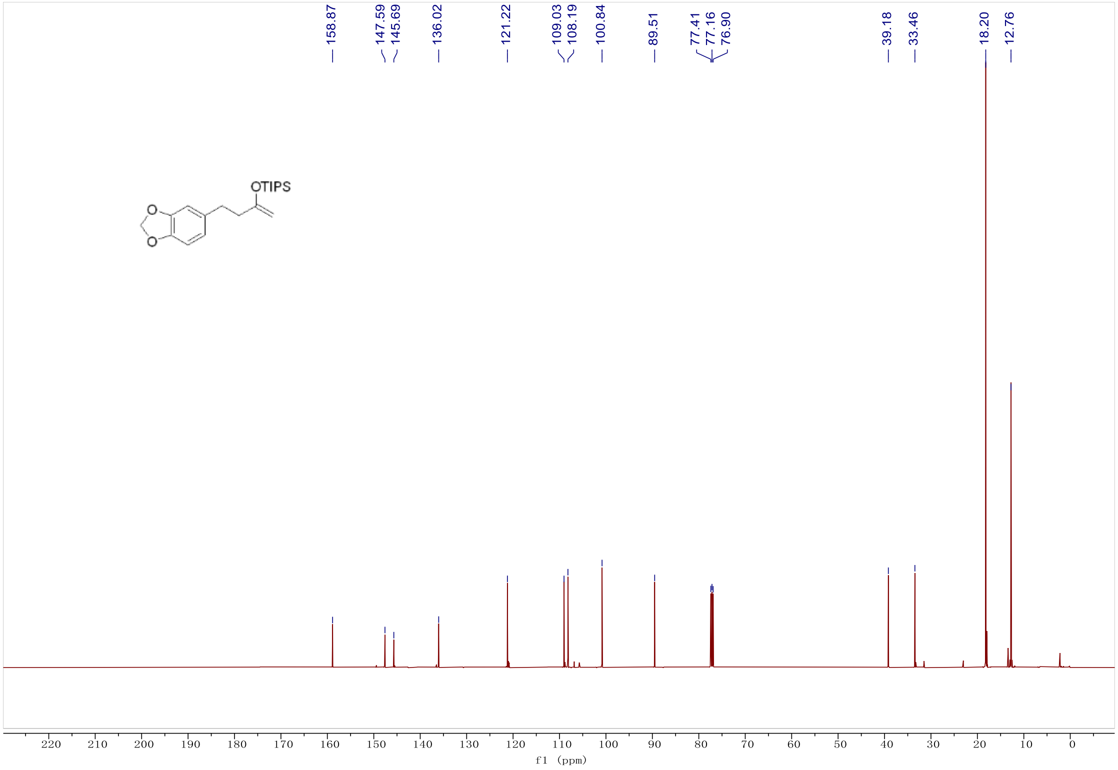

Supplementary Figure 29. ^{13}C NMR spectrum (125 MHz, $CDCl_3$) of **1ab**

Compound 1ac

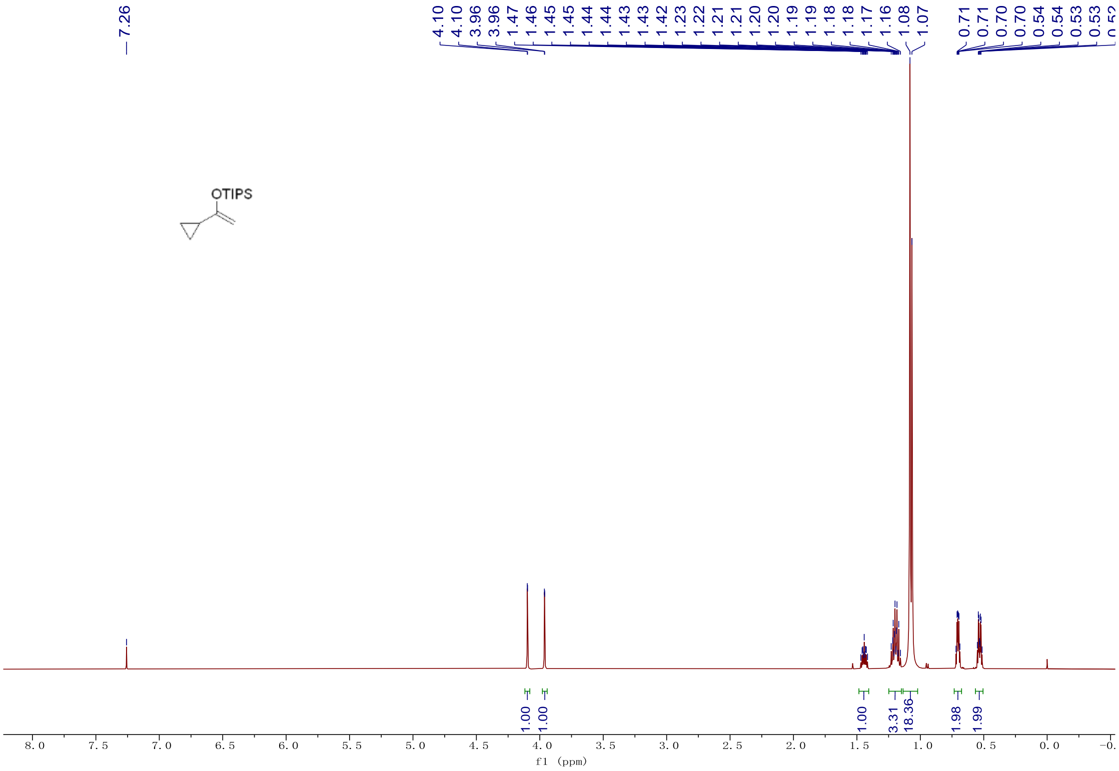

Supplementary Figure 30. ^{1}**H NMR** spectrum (500 MHz, $CDCl_3$) of **1ac**

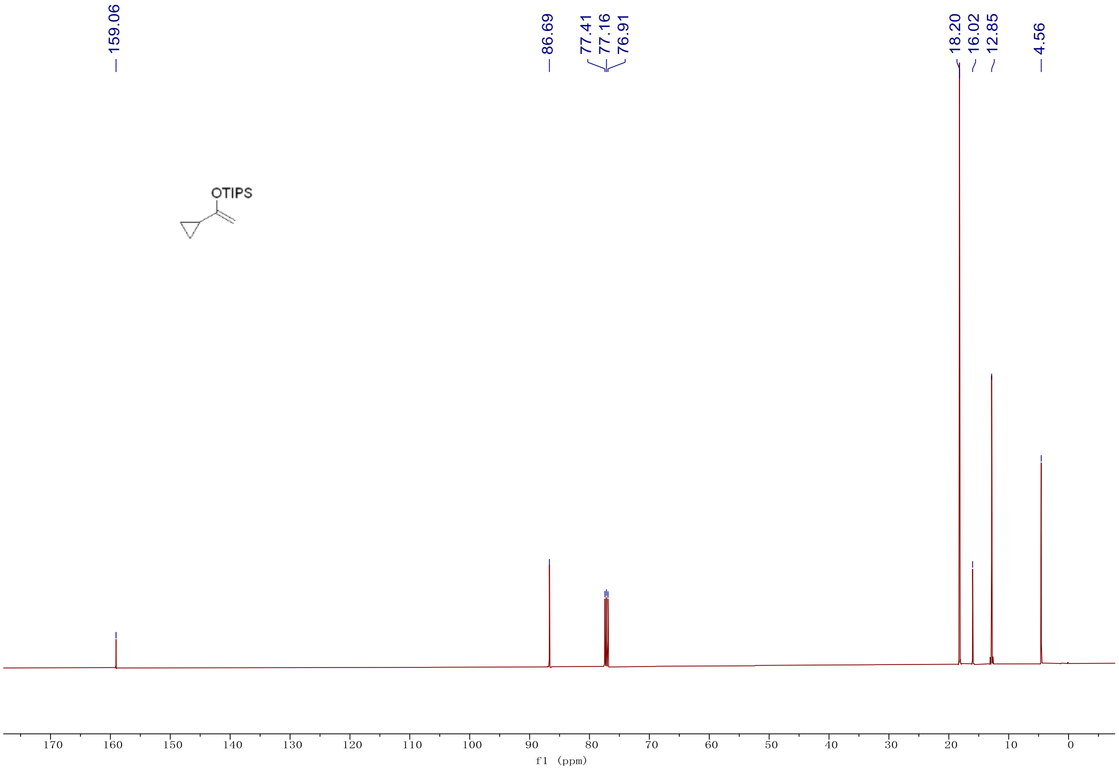

Supplementary Figure 31. ^{13}**C NMR** spectrum (125 MHz, $CDCl_3$) of **1ac**

Compound 1aj

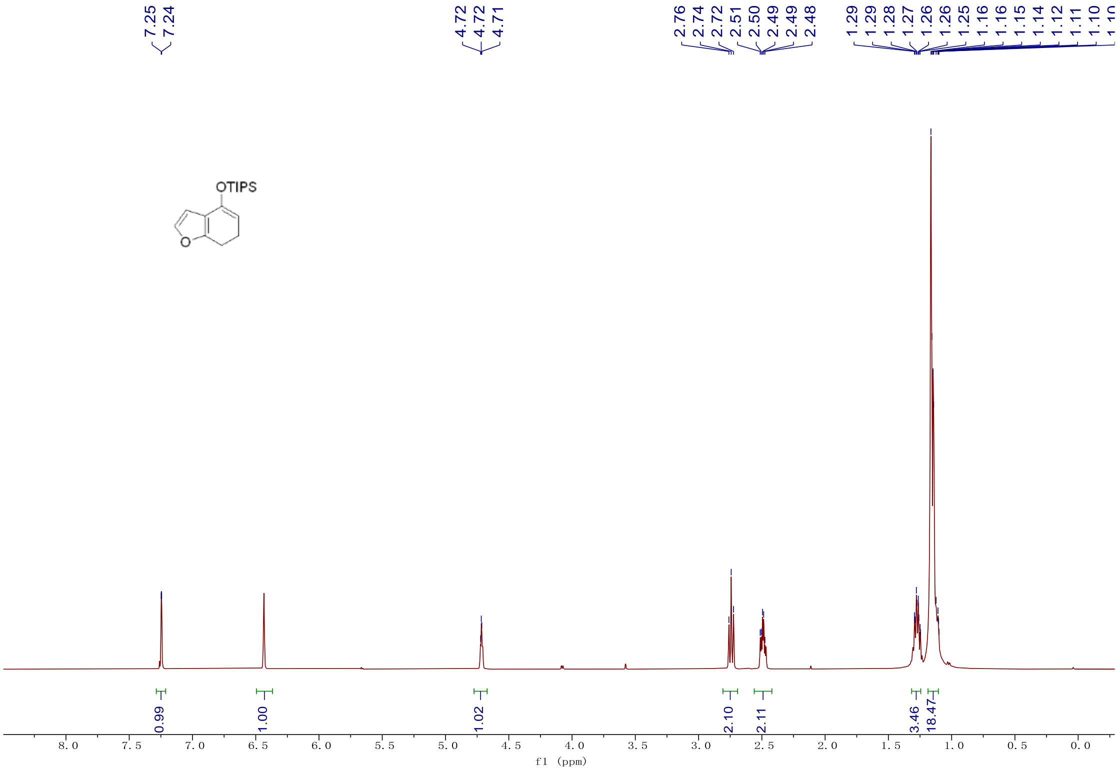

Supplementary Figure 32. ^{1}**H NMR** spectrum (500 MHz, $CDCl_3$) of **1aj**

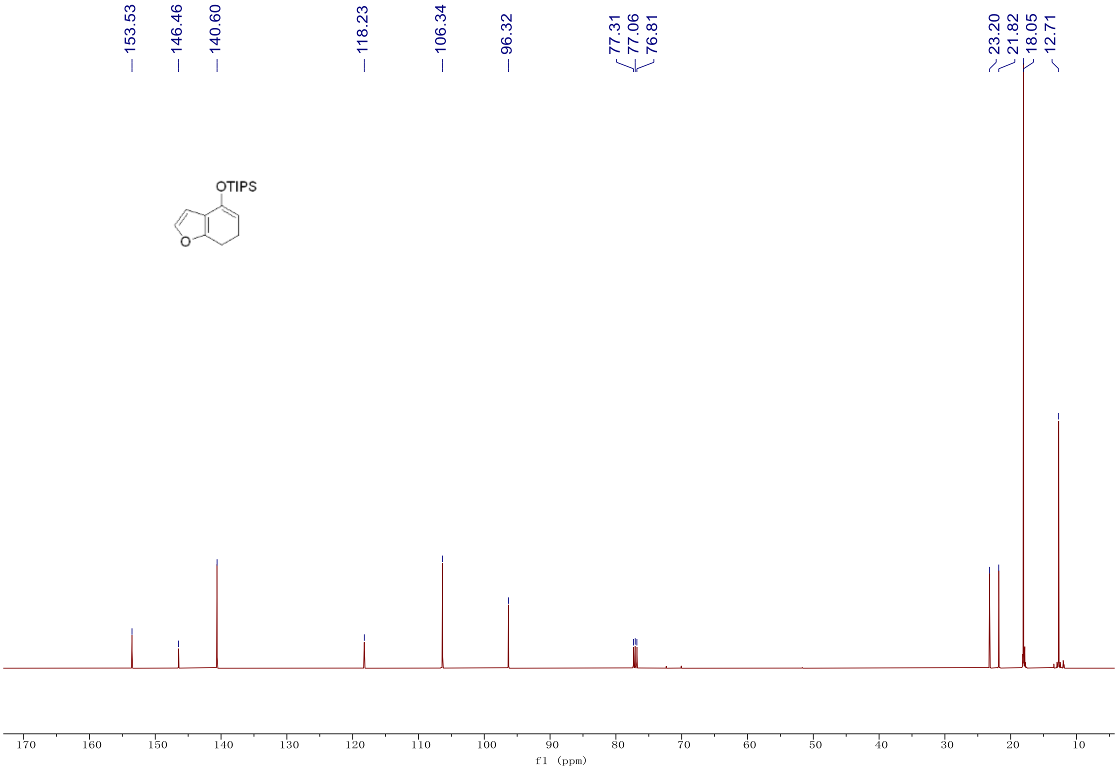

Supplementary Figure 33. ^{13}**C NMR** spectrum (125 MHz, $CDCl_3$) of **1aj**

Compound 1ak

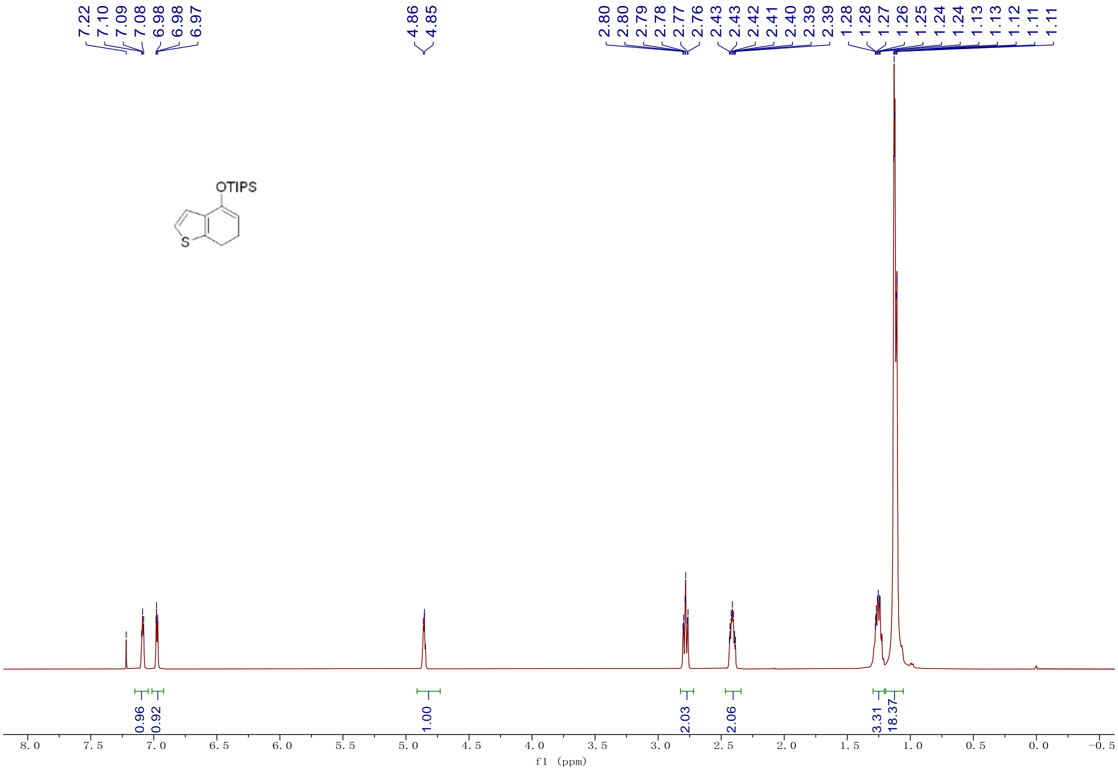

Supplementary Figure 34. ^{1}**H NMR** spectrum (500 MHz, $CDCl_3$) of **1ak**

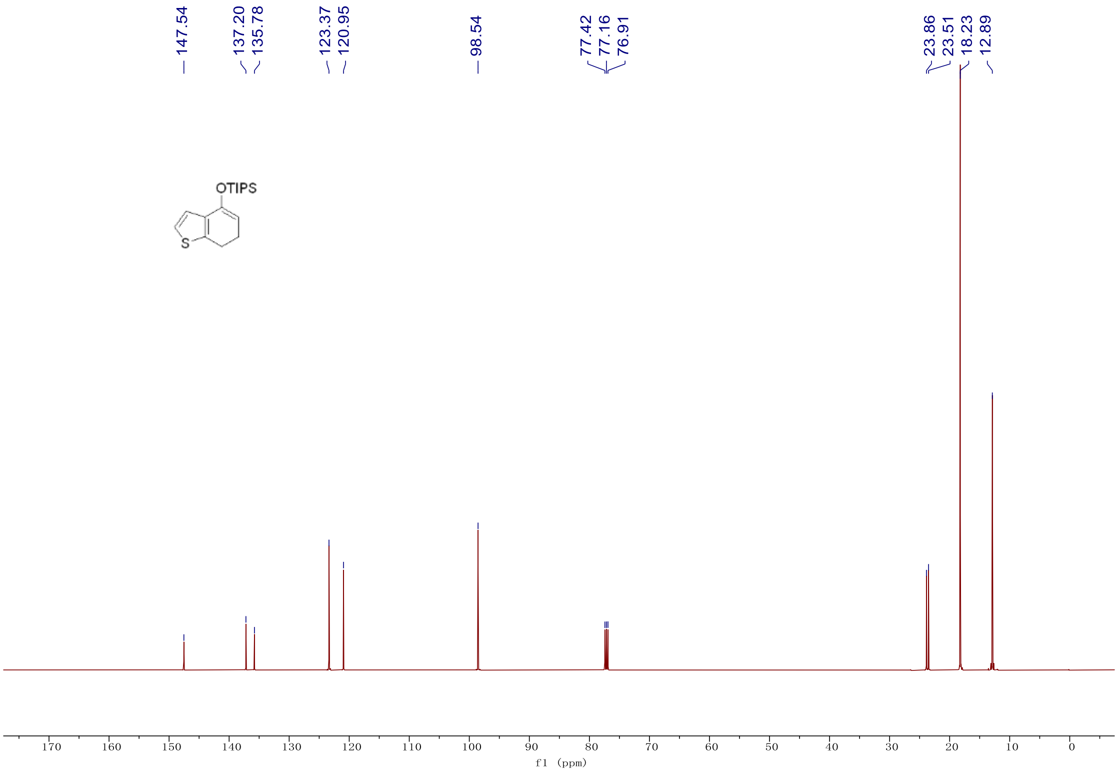

Supplementary Figure 35. ^{13}**C NMR** spectrum (125 MHz, $CDCl_3$) of **1ak**

Compound 1an

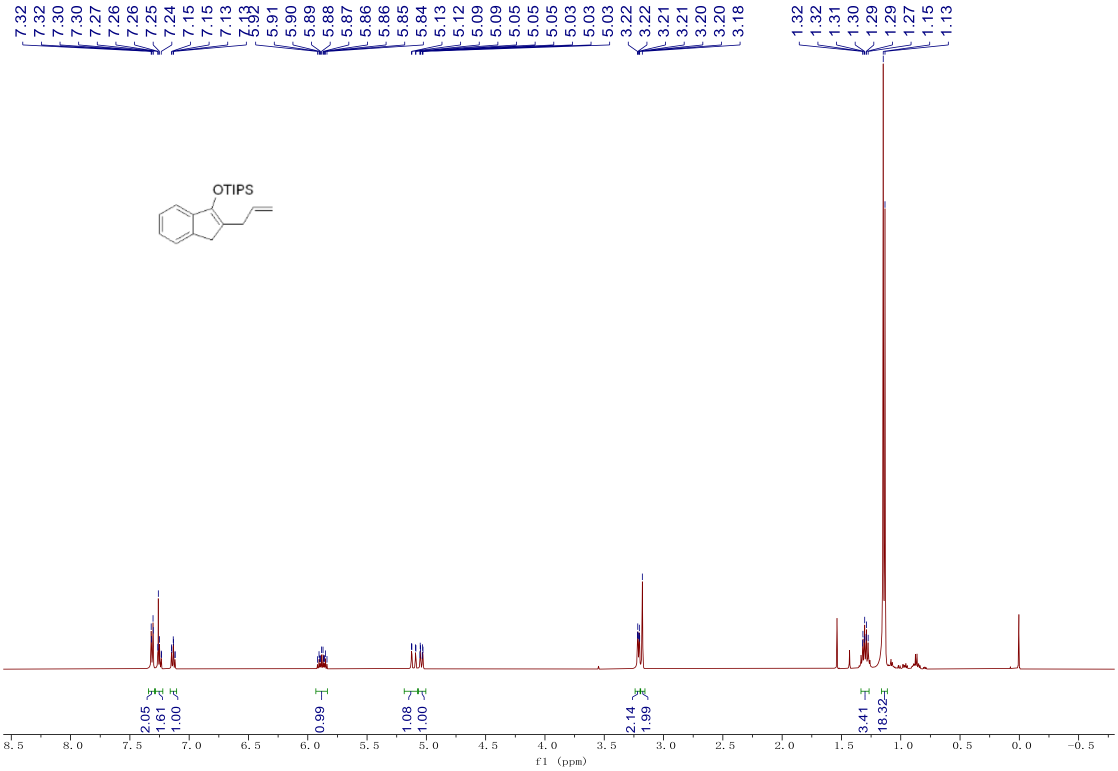

Supplementary Figure 36. ^{1}H NMR spectrum (500 MHz, $CDCl_3$) of **1an**

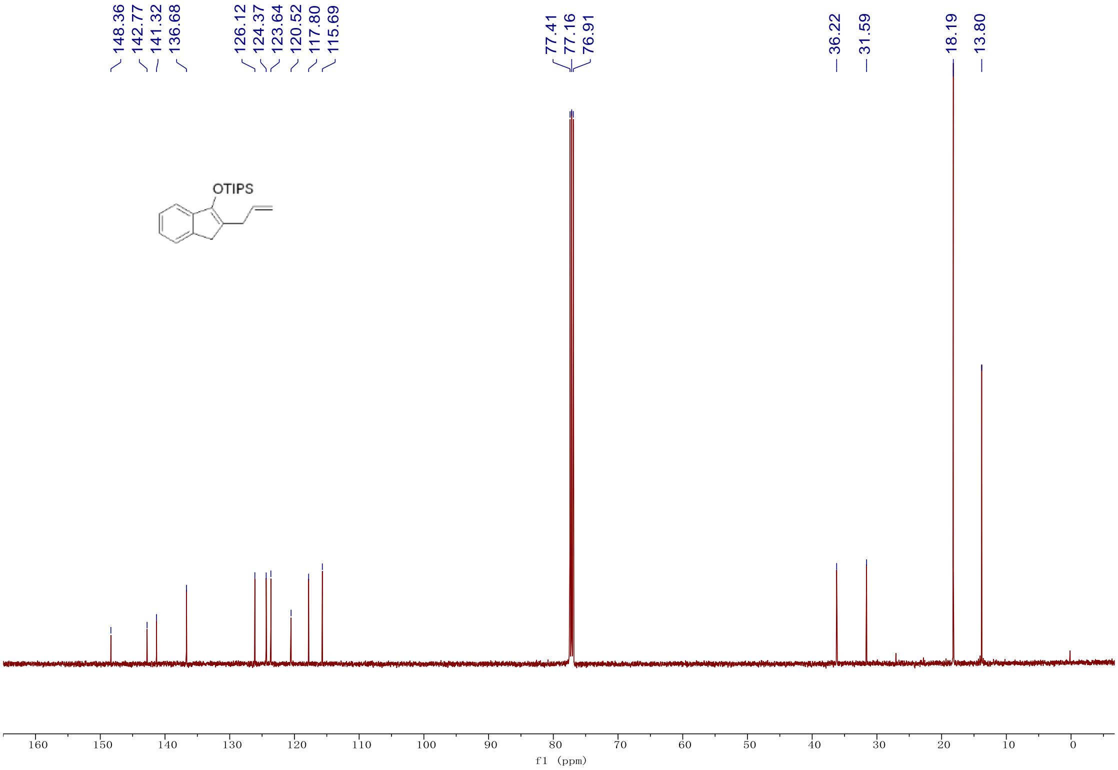

Supplementary Figure 37. ^{13}C NMR spectrum (125 MHz, $CDCl_3$) of **1an**

Compound 1ar

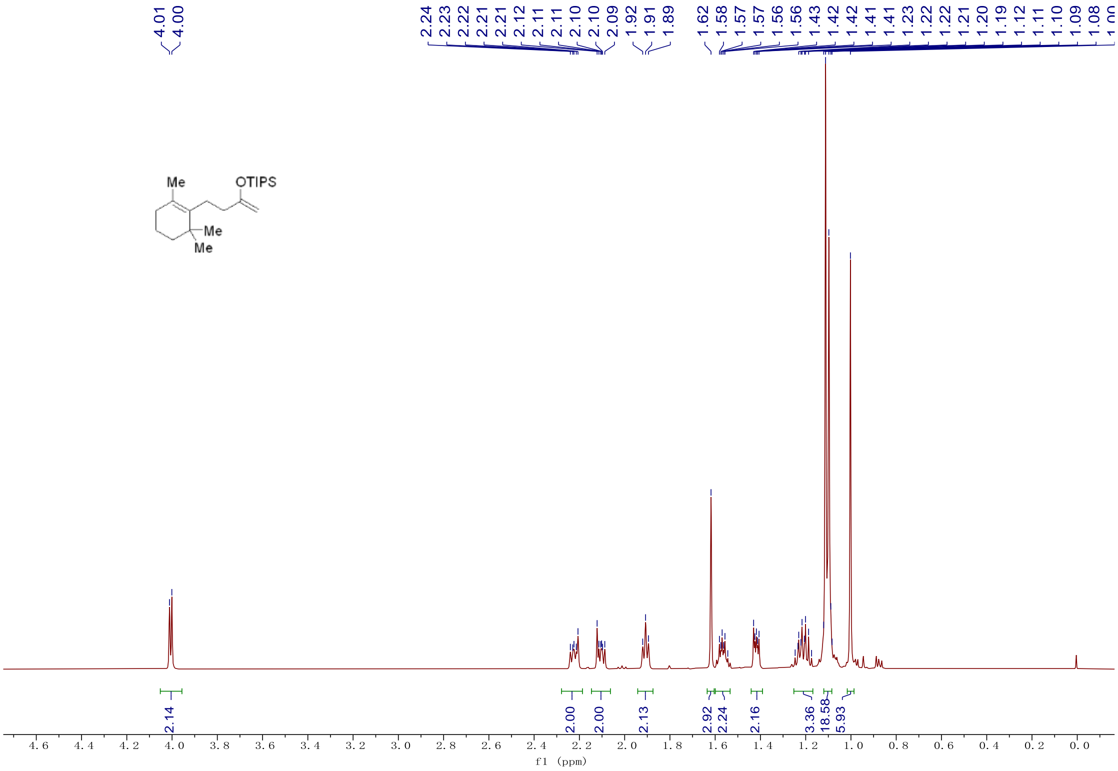

Supplementary Figure 38. ^{1}**H NMR** spectrum (500 MHz, $CDCl_3$) of **1ar**

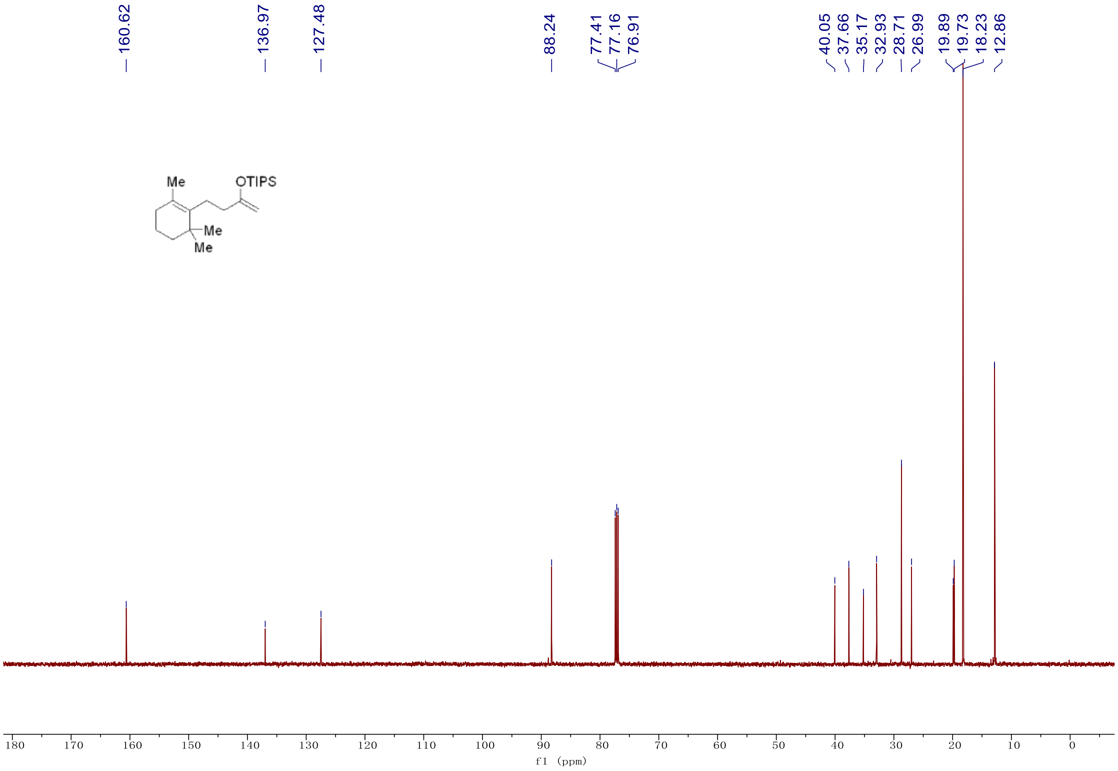

Supplementary Figure 39. ^{13}**C NMR** spectrum (125 MHz, $CDCl_3$) of **1ar**

Compound 1au

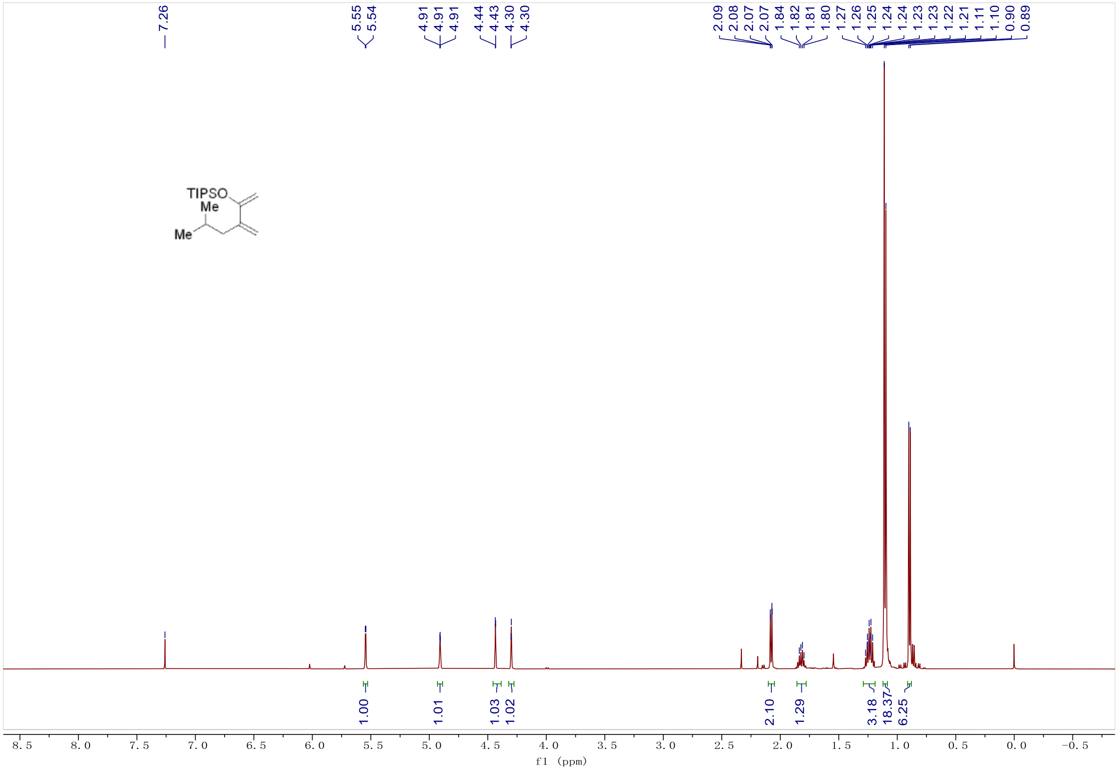

Supplementary Figure 40. ^{1}**H NMR** spectrum (500 MHz, $CDCl_3$) of **1au**

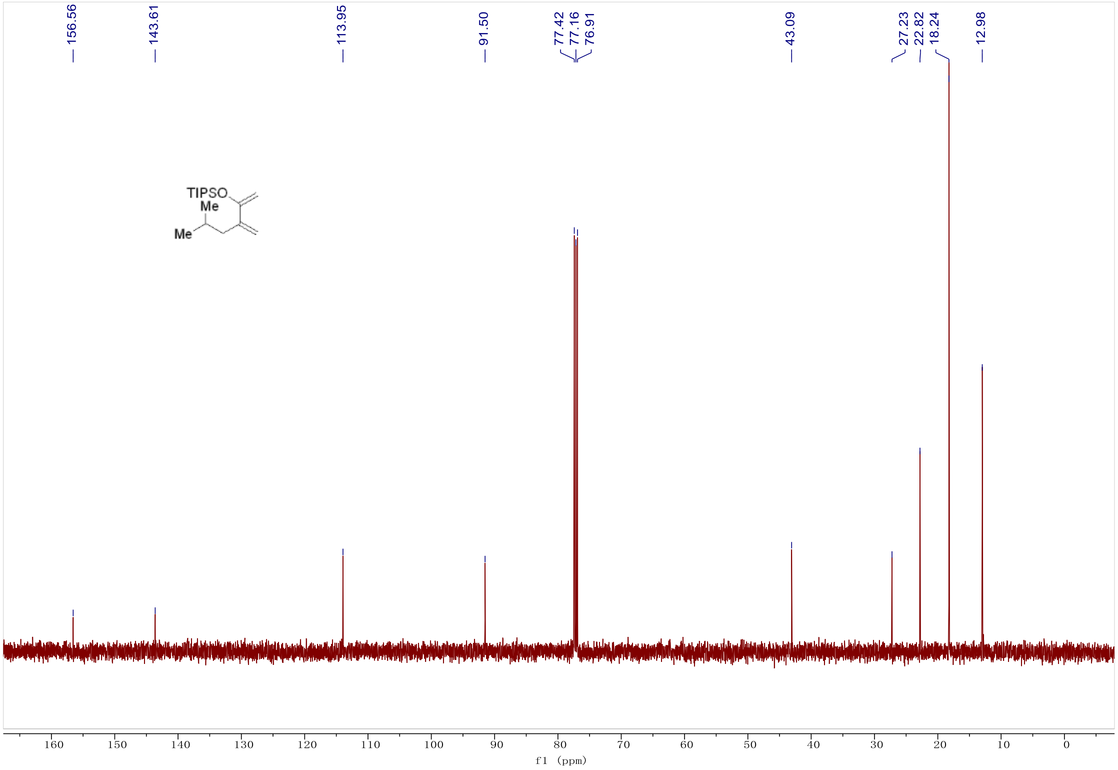

Supplementary Figure 41. ^{13}**C NMR** spectrum (125 MHz, $CDCl_3$) of **1au**

Compound 1ax

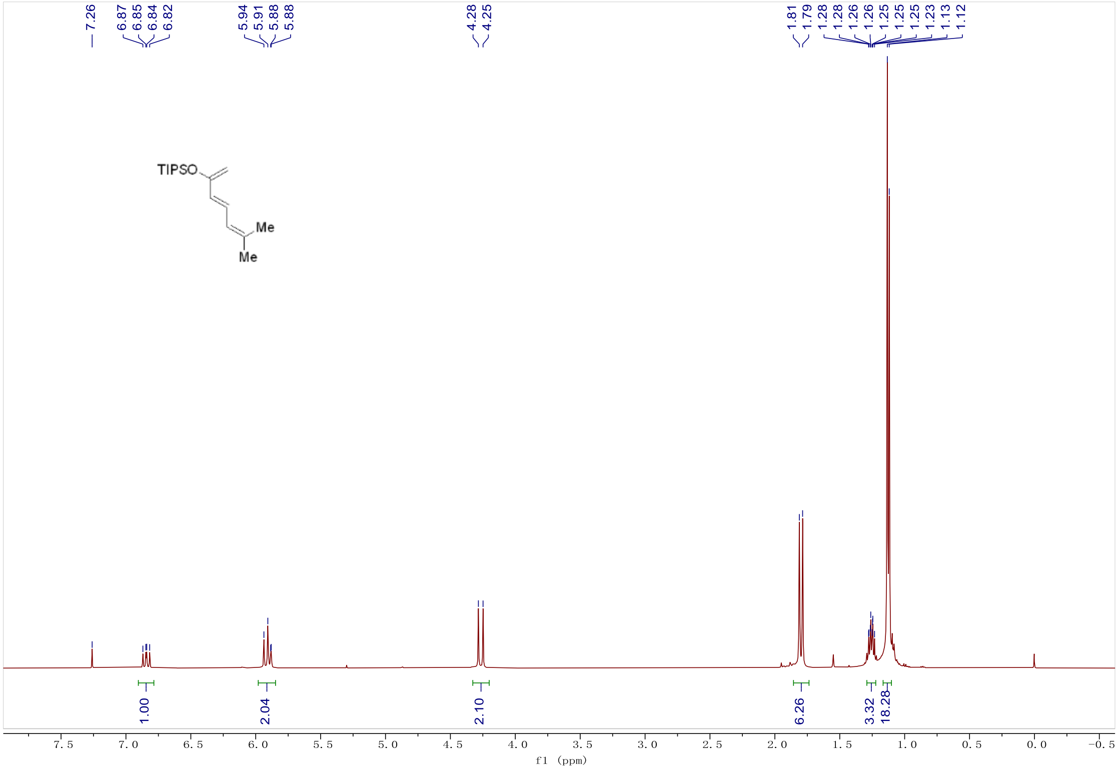

Supplementary Figure 42. ^{1}H NMR spectrum (500 MHz, $CDCl_3$) of **1ax**

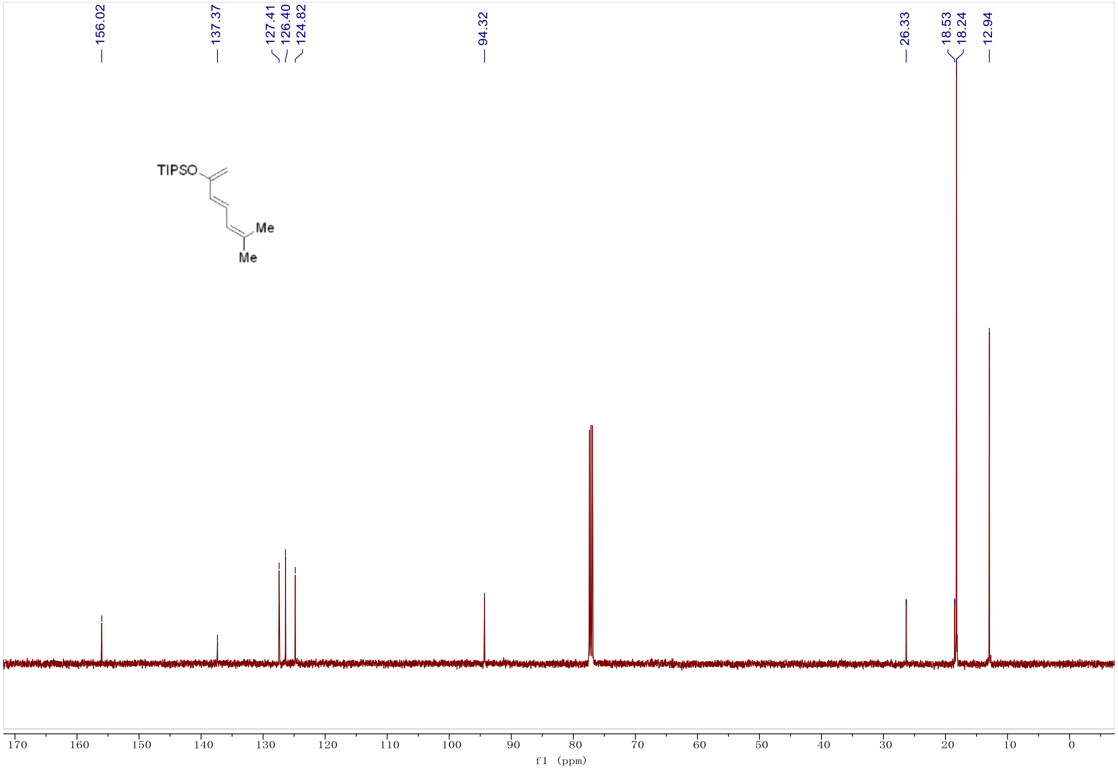

Supplementary Figure 43. ^{13}C NMR spectrum (125 MHz, $CDCl_3$) of **1ax**

Compound 1ba

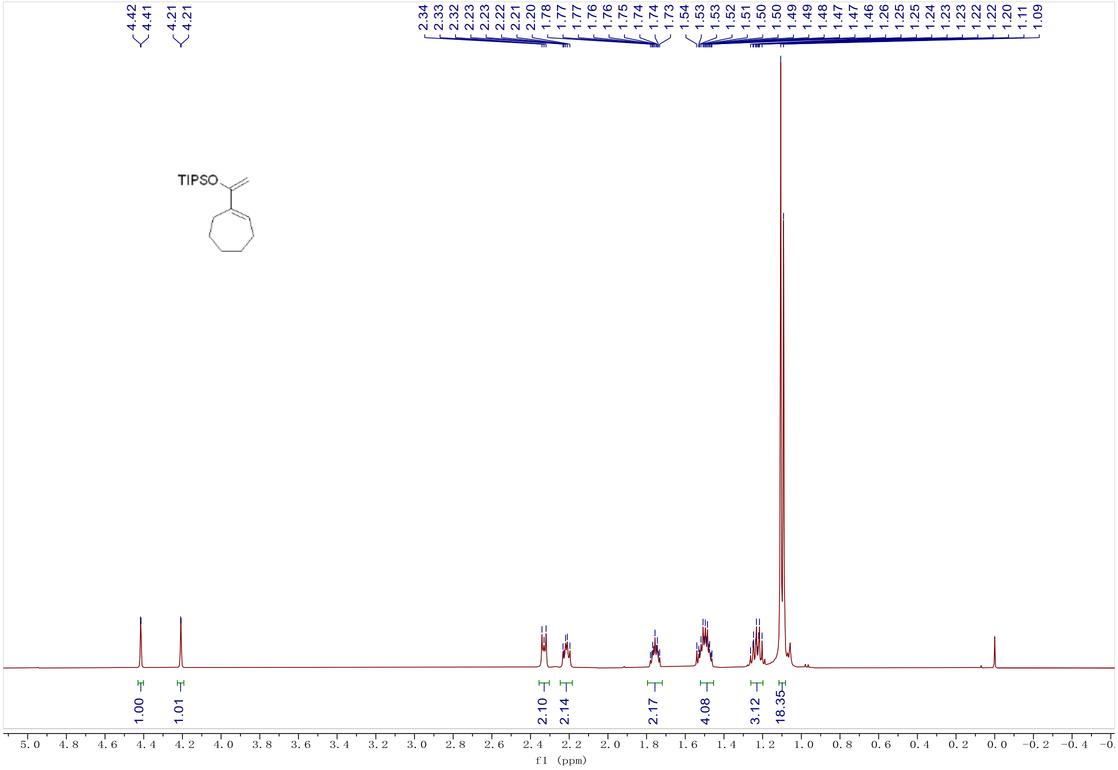

Supplementary Figure 44. ^{1}H NMR spectrum (500 MHz, $CDCl_3$) of **1ba**

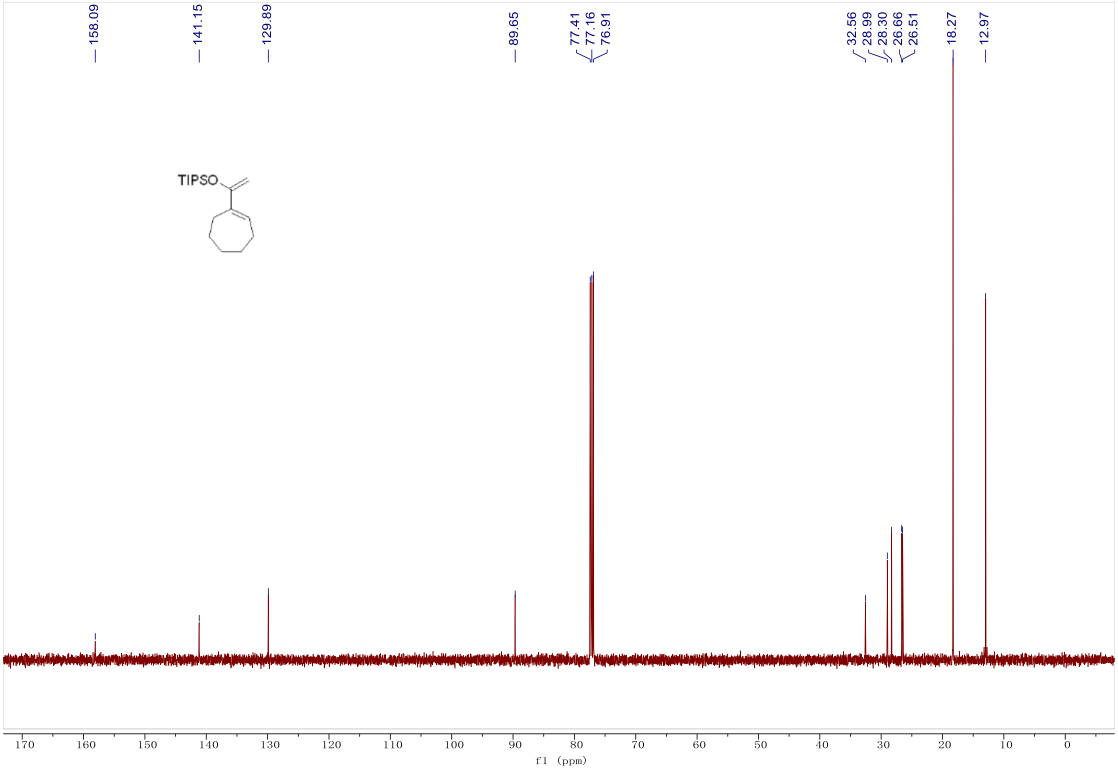

Supplementary Figure 45. ^{13}C NMR spectrum (125 MHz, $CDCl_3$) of **1ba**

Compound 1bb

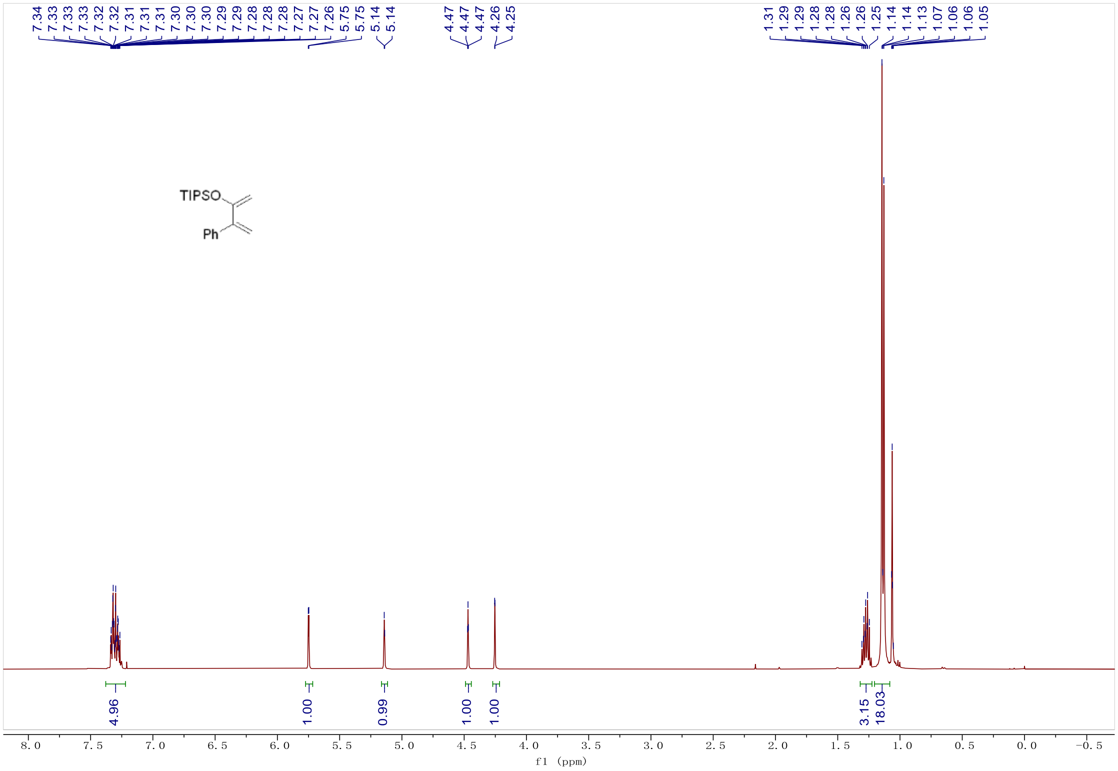

Supplementary Figure 46. ^{1}H NMR spectrum (500 MHz, $CDCl_3$) of **1bb**

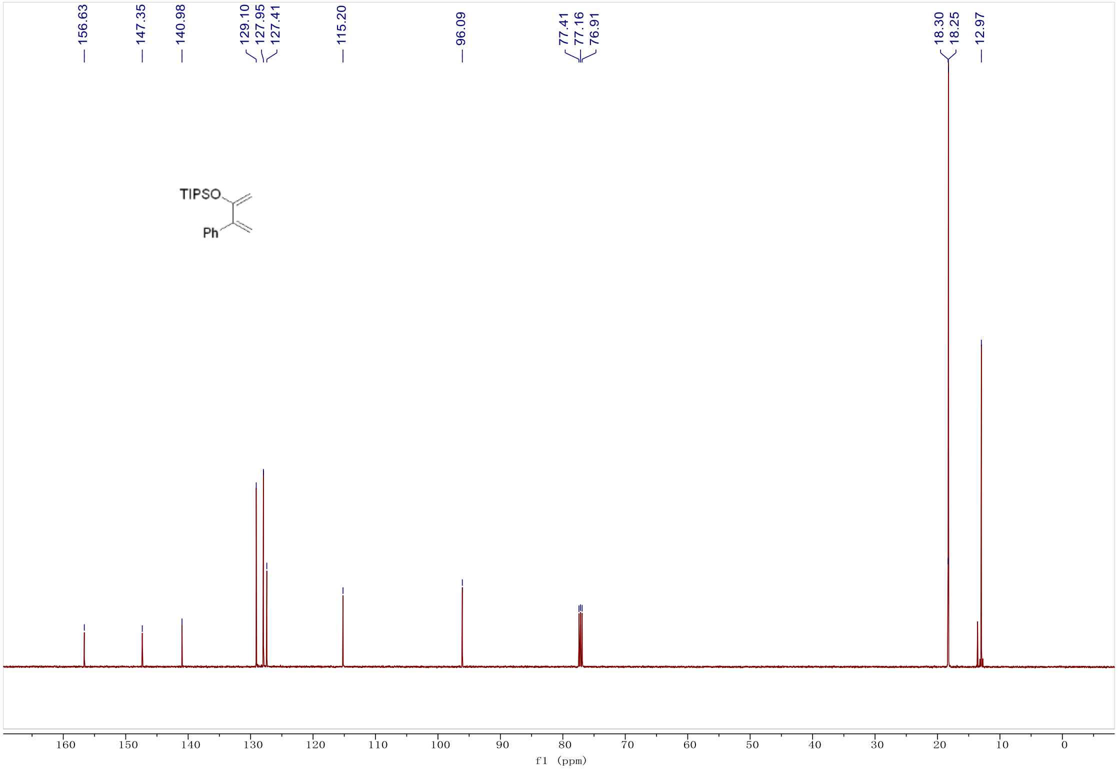

Supplementary Figure 47. ^{13}C NMR spectrum (125 MHz, $CDCl_3$) of **1bb**

Compound 3a

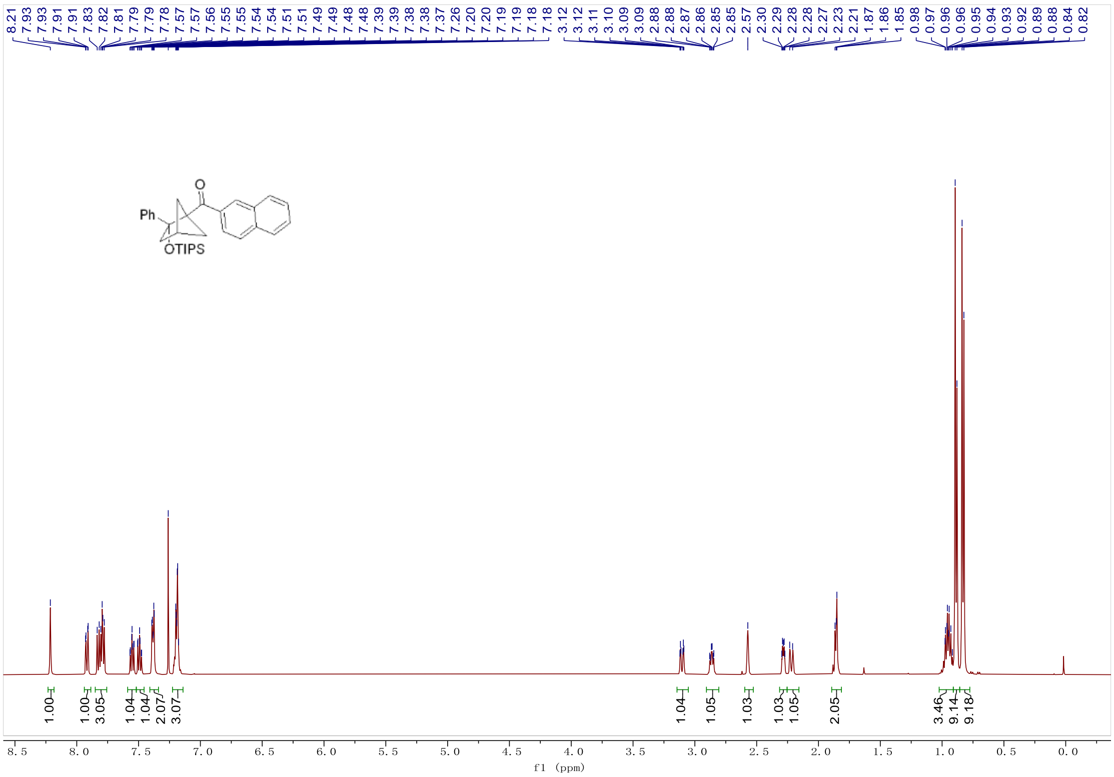

Supplementary Figure 48. ^{1}H NMR spectrum (500 MHz, $CDCl_3$) of **3a**

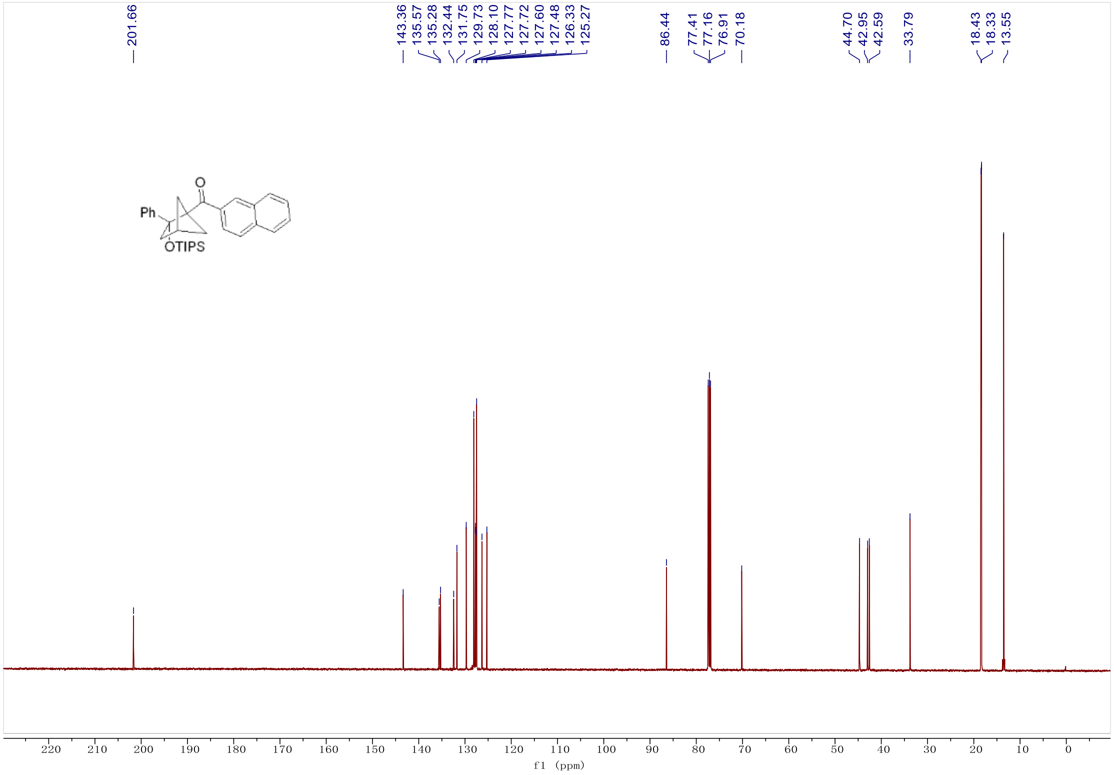

Supplementary Figure 49. ^{13}C NMR spectrum (125 MHz, $CDCl_3$) of **3a**

Compound 4

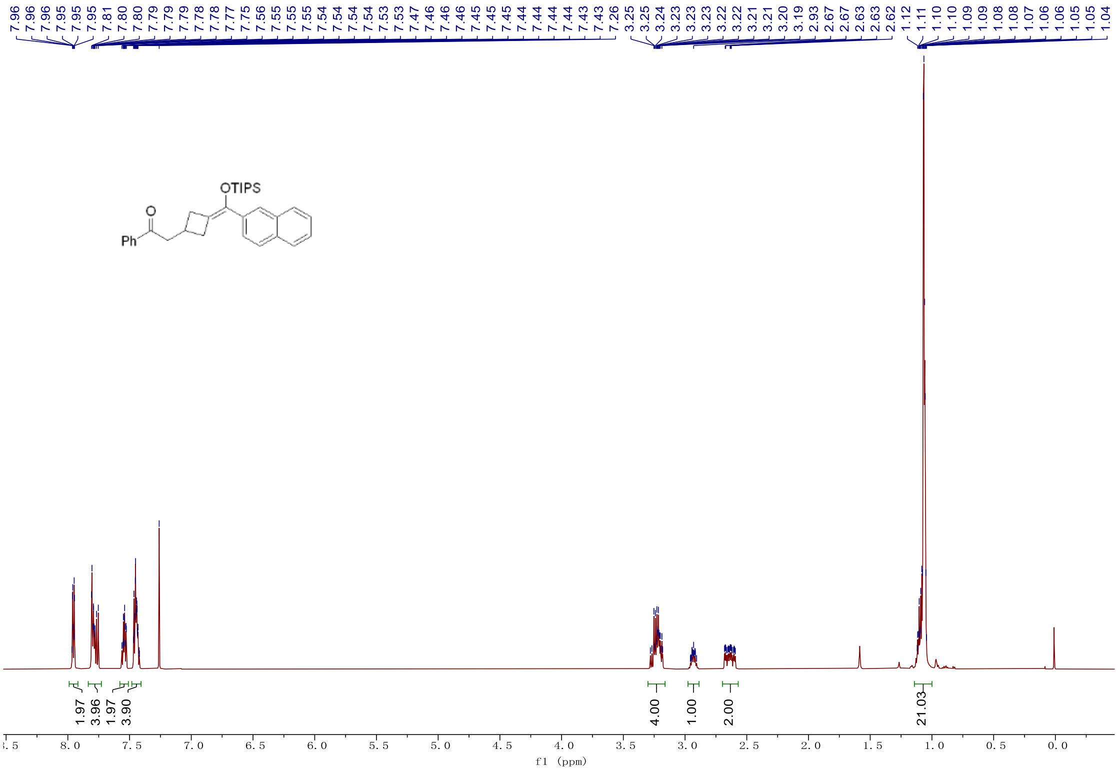

Supplementary Figure 50. ^{1}**H NMR** spectrum (500 MHz, $CDCl_3$) of **4**

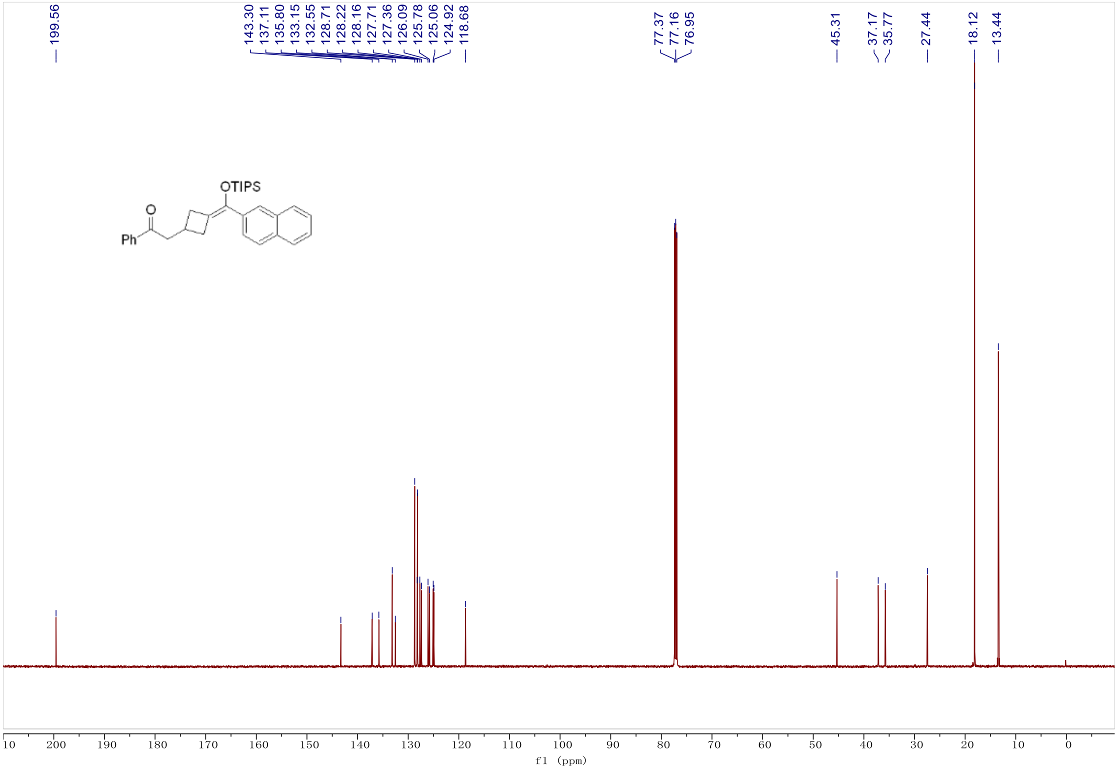

Supplementary Figure 51. ^{13}**C NMR** spectrum (125 MHz, $CDCl_3$) of **4**

Compound 3b

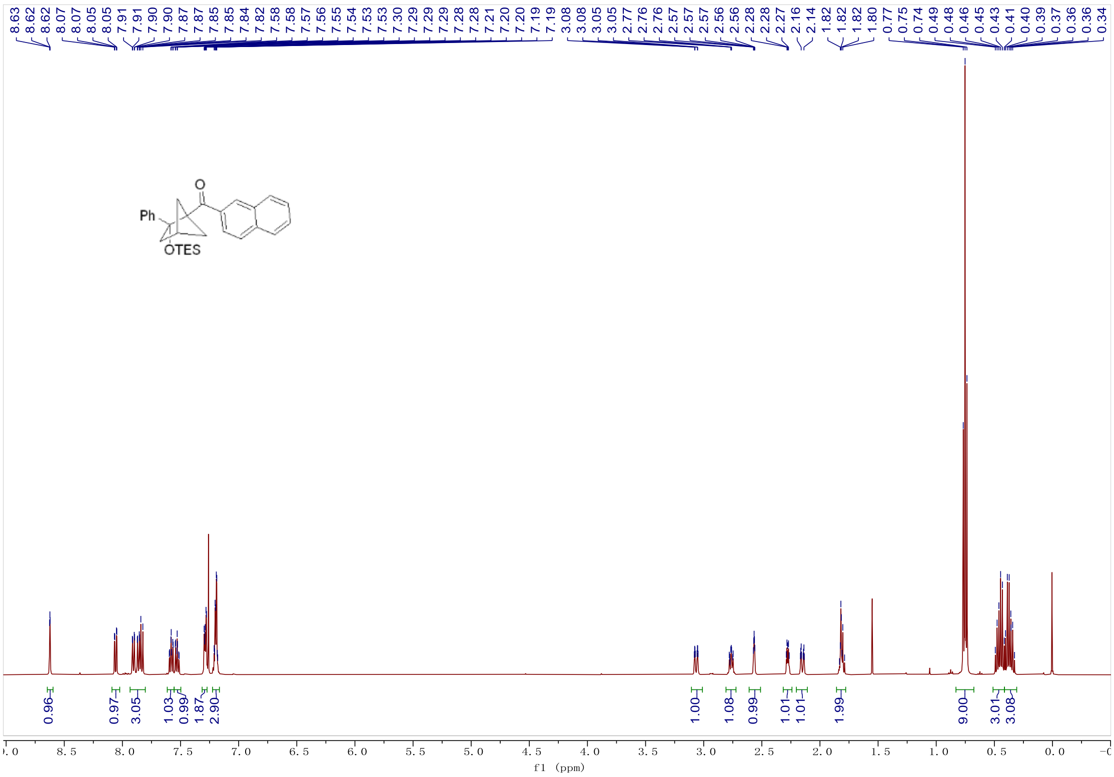

Supplementary Figure 52. ¹H NMR spectrum (500 MHz, CDCl3) of **3b**

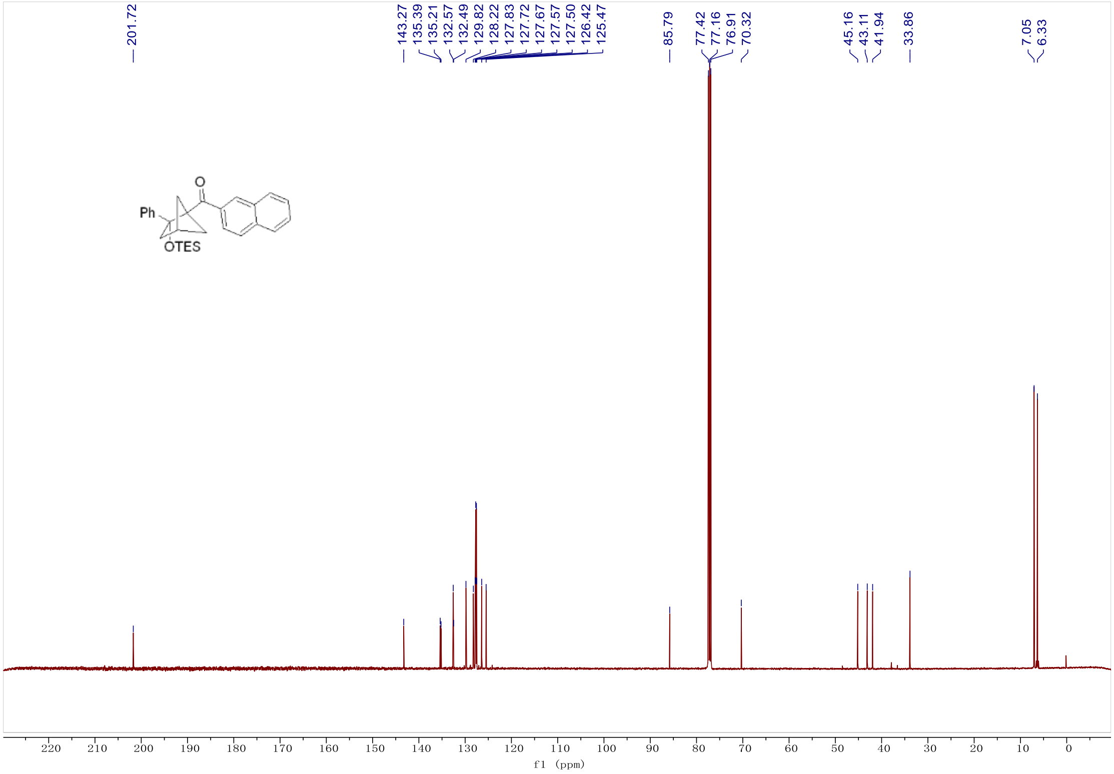

Supplementary Figure 53. ¹³C NMR spectrum (125 MHz, CDCl3) of **3b**

Compound 3c

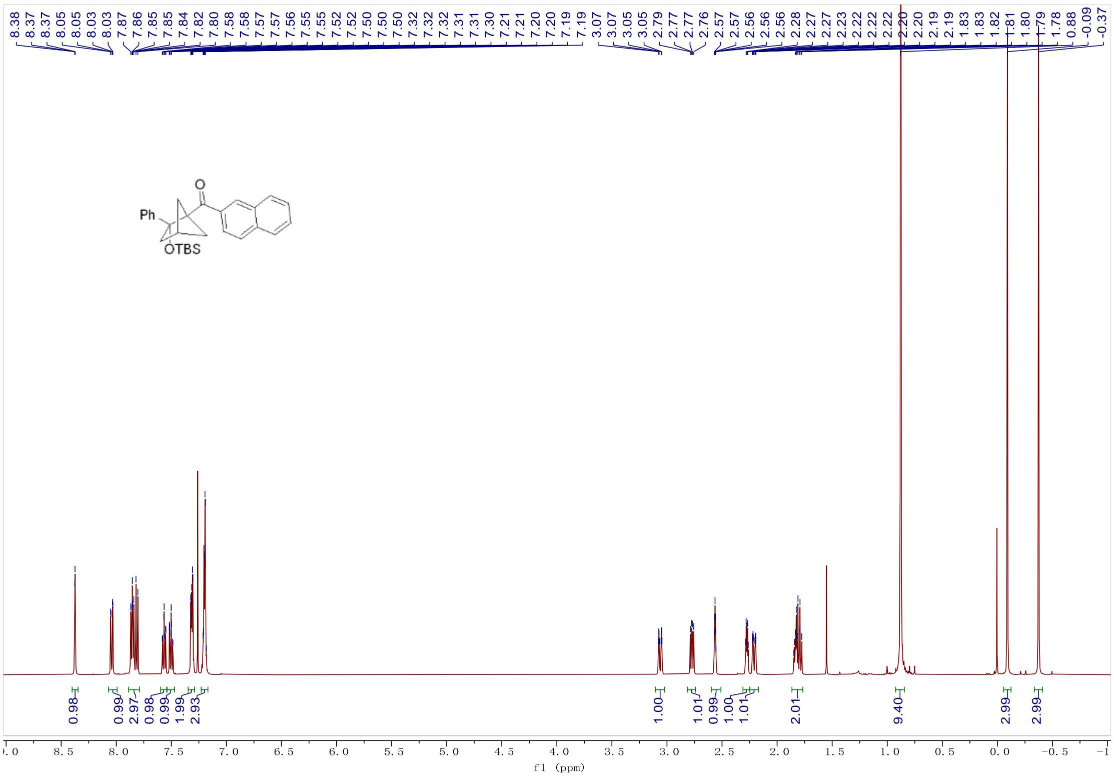

Supplementary Figure 54. ^{1}H NMR spectrum (500 MHz, $CDCl_3$) of **3c**

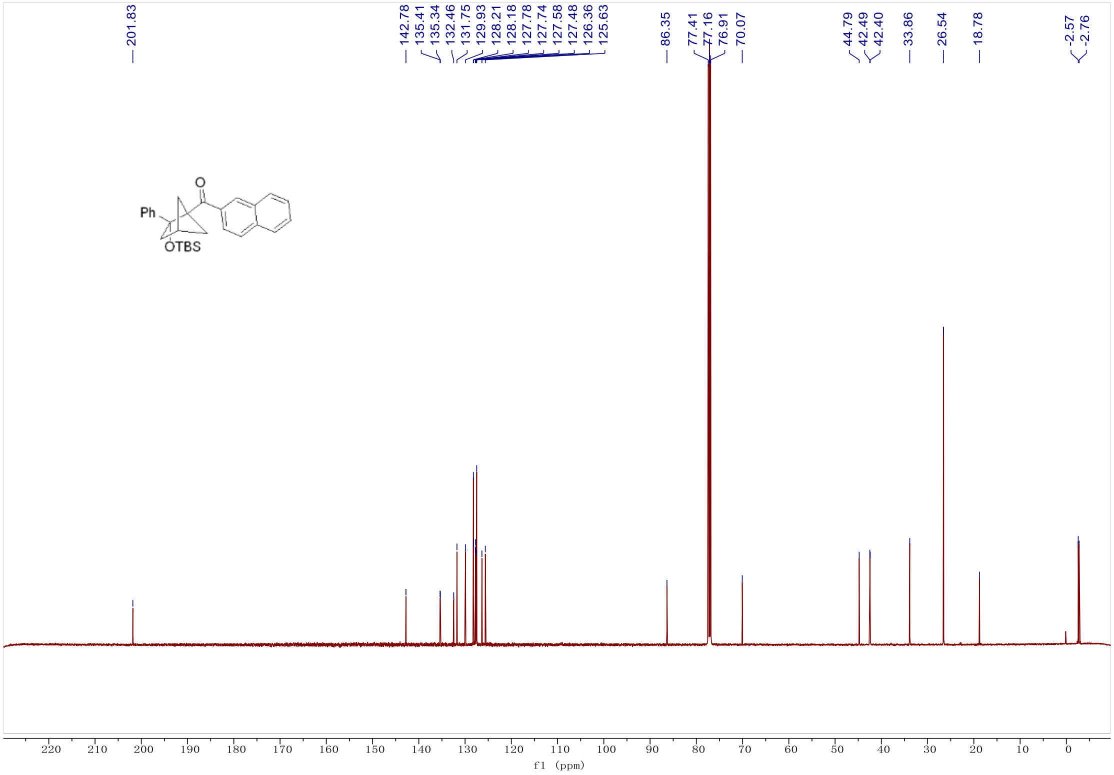

Supplementary Figure 55. ^{13}C NMR spectrum (125 MHz, $CDCl_3$) of **3c**

Compound 3d

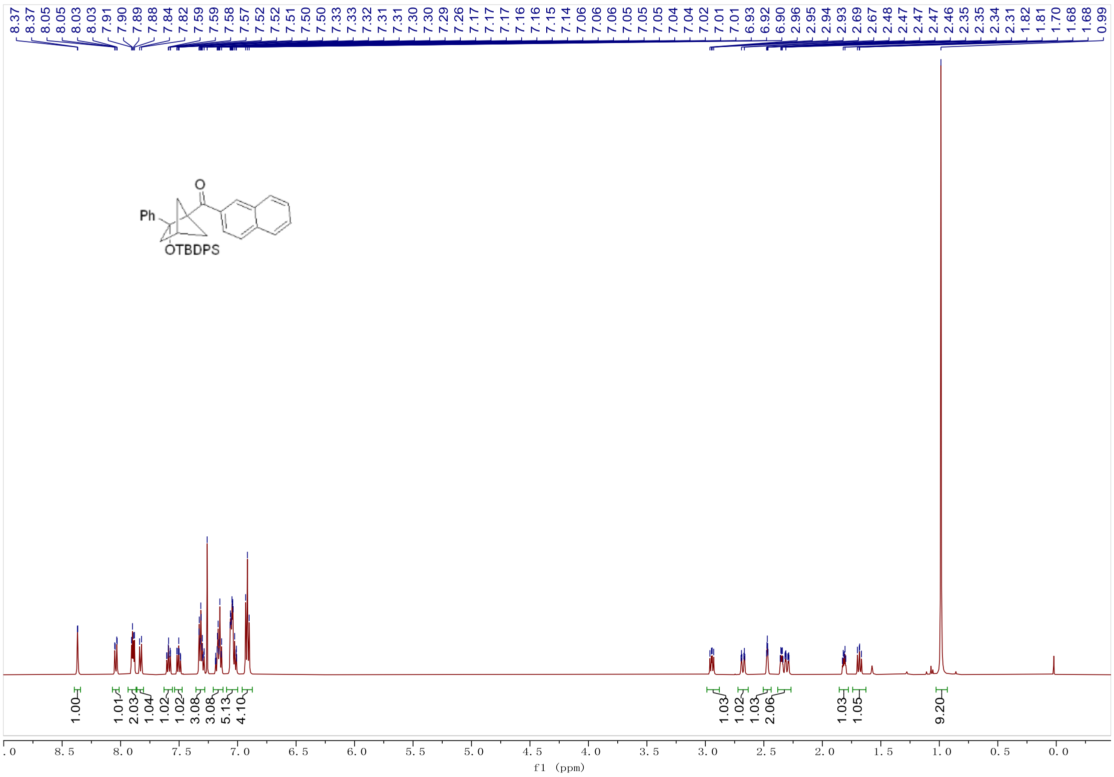

Supplementary Figure 56. ^{1}H NMR spectrum (500 MHz, $CDCl_3$) of **3d**

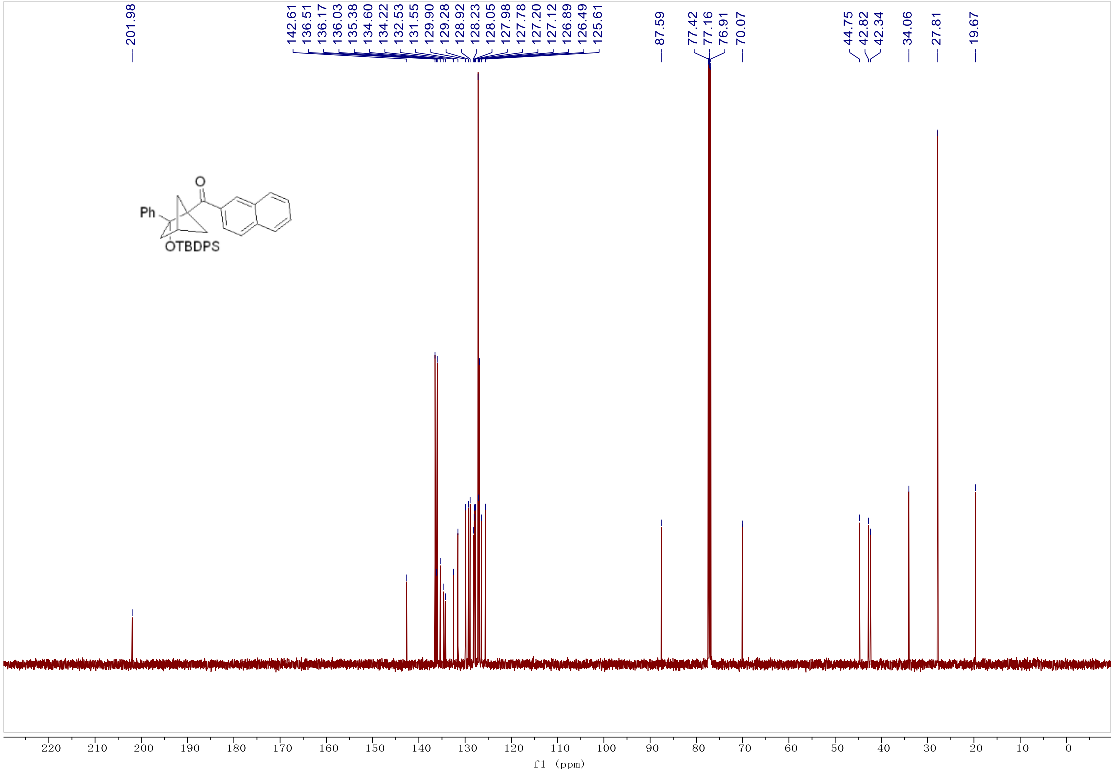

Supplementary Figure 57. ^{13}C NMR spectrum (125 MHz, $CDCl_3$) of **3d**

Compound 3e

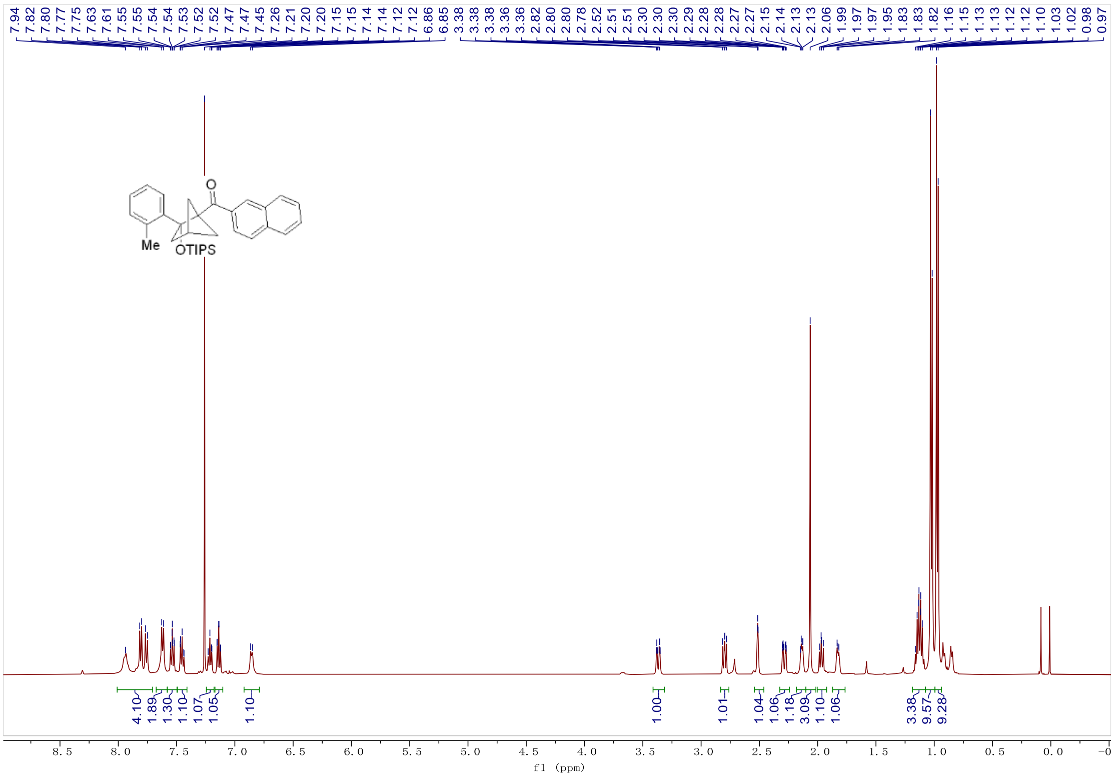

Supplementary Figure 58. **^{1}H NMR** spectrum (500 MHz, $CDCl_3$) of **3e**

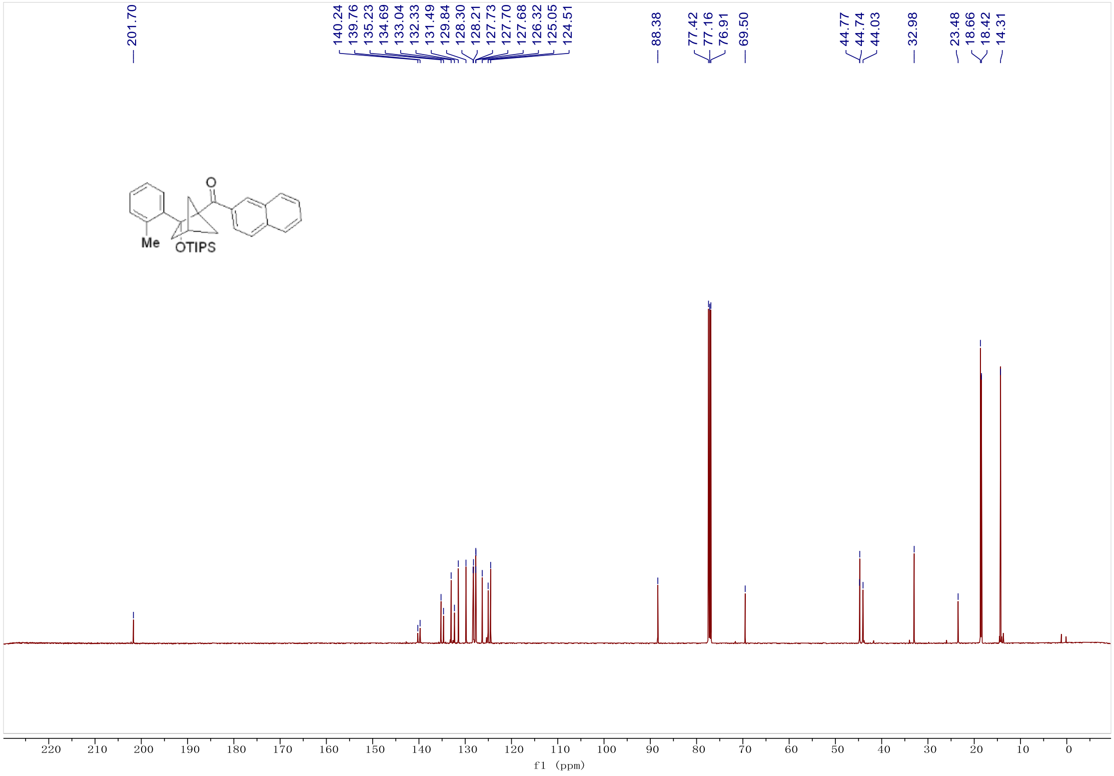

Supplementary Figure 59. **^{13}C NMR** spectrum (125 MHz, $CDCl_3$) of **3e**

Compound 3f

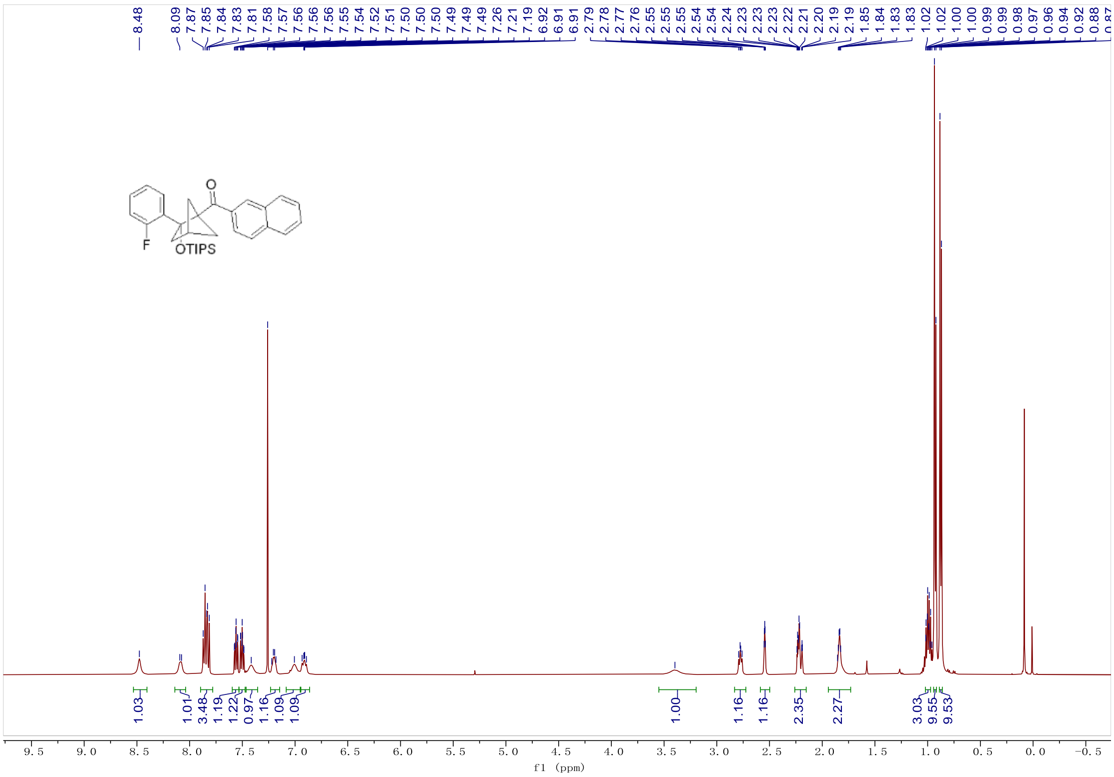

Supplementary Figure 60. ^{1}**H NMR** spectrum (500 MHz, $CDCl_3$) of **3f**

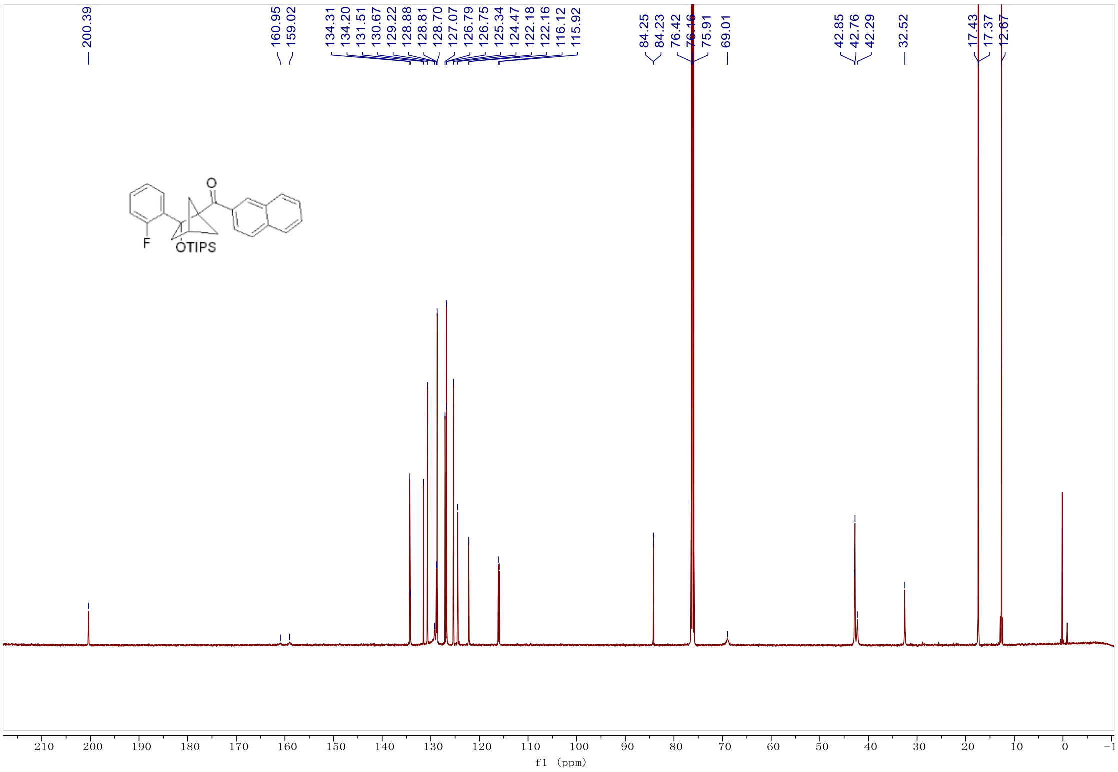

Supplementary Figure 61. ^{13}**C NMR** spectrum (125 MHz, $CDCl_3$) of **3f**

Compound 3f

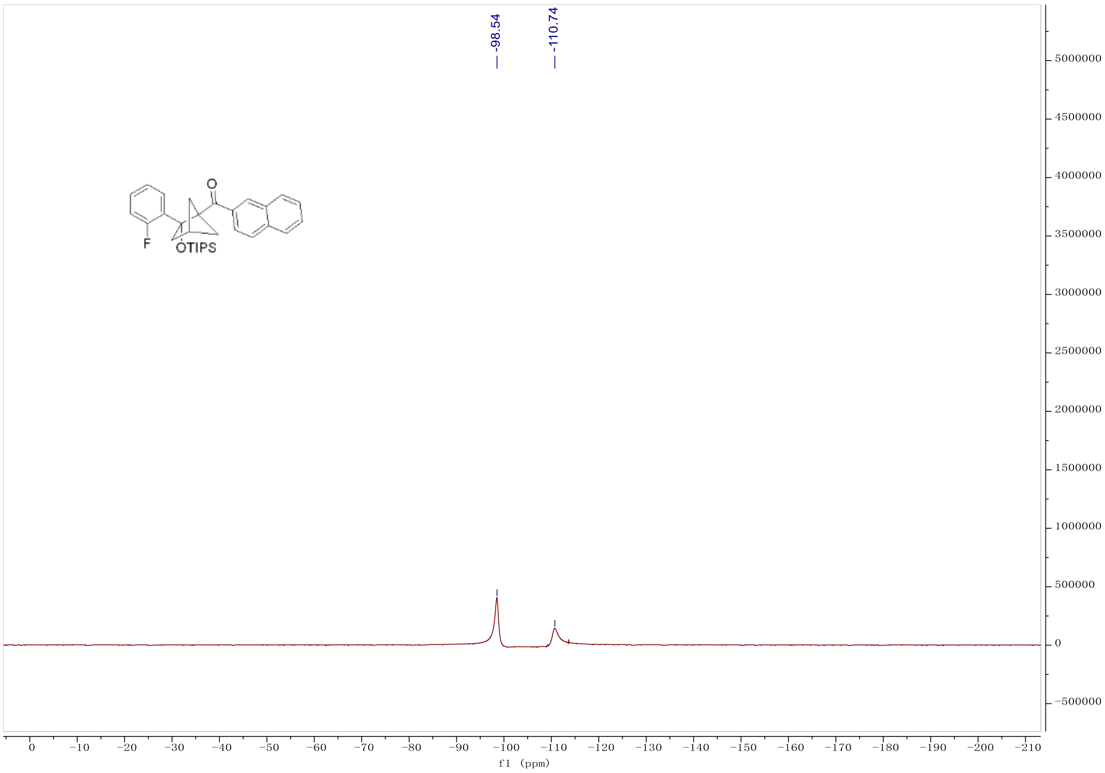

Supplementary Figure 62. ^{19}F NMR spectrum of (565 MHz, $CDCl_3$) of **3f**

Compound 3g

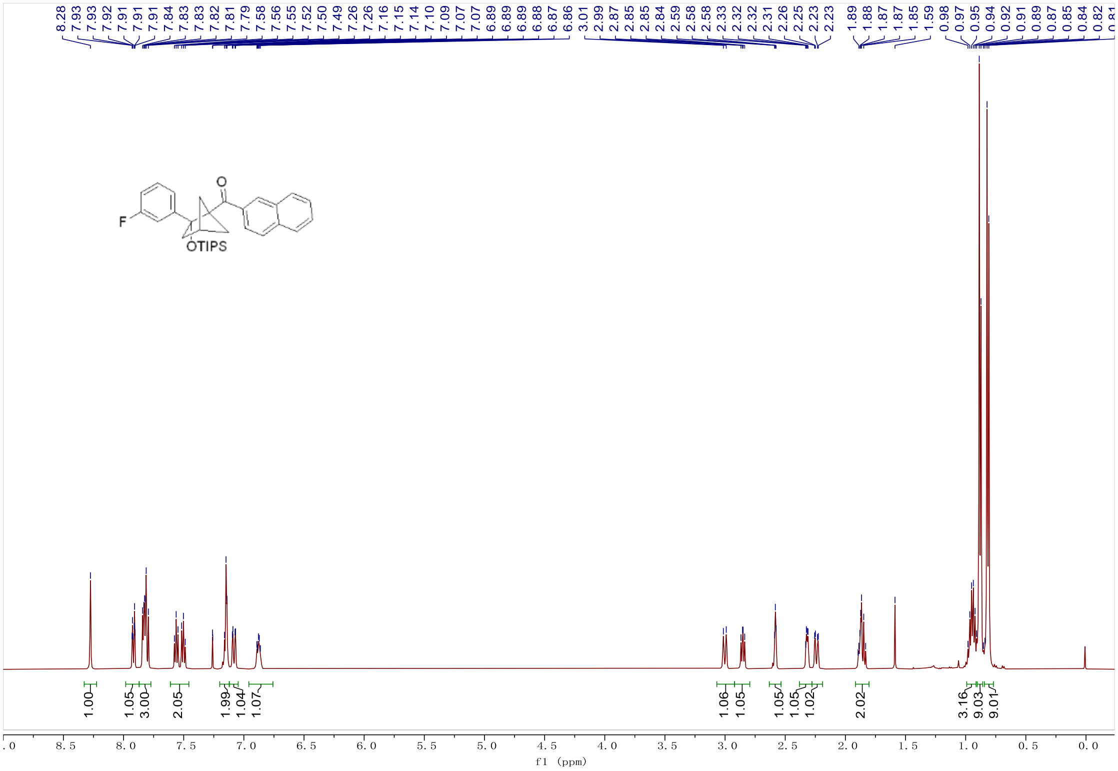

Supplementary Figure 63. ^{1}H NMR spectrum (500 MHz, $CDCl_3$) of **3g**

Compound 3g

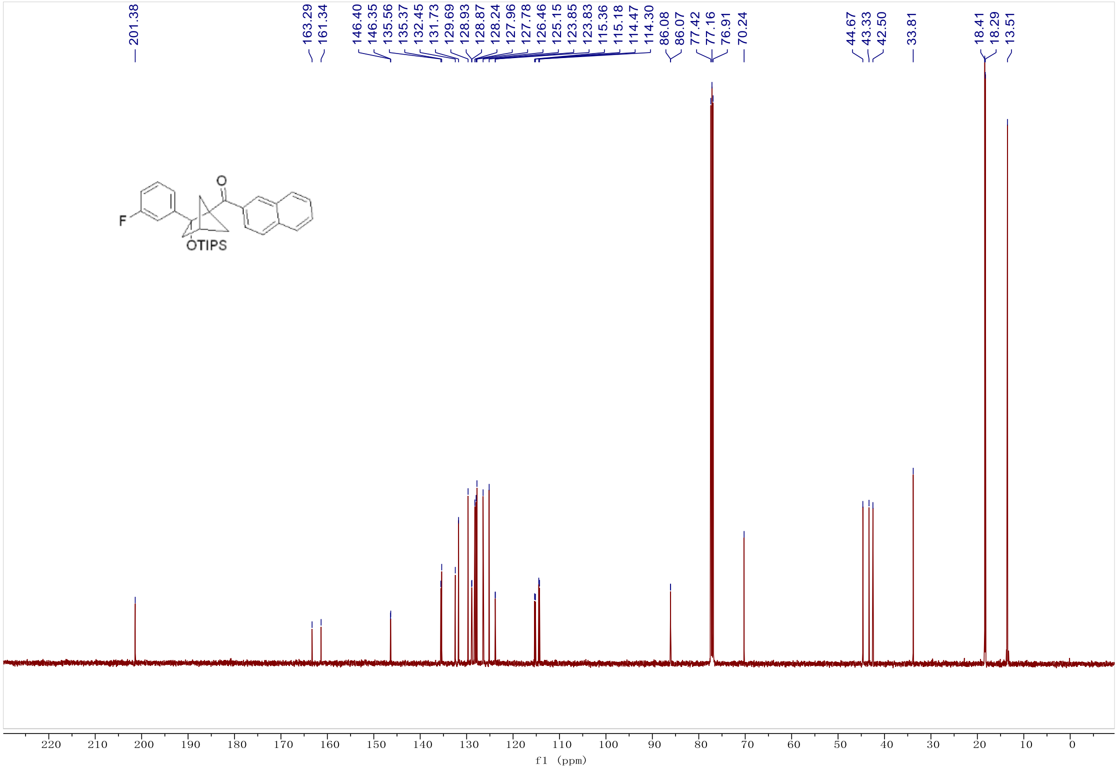

Supplementary Figure 64. ^{13}**C NMR** spectrum (125 MHz, $CDCl_3$) of **3g**

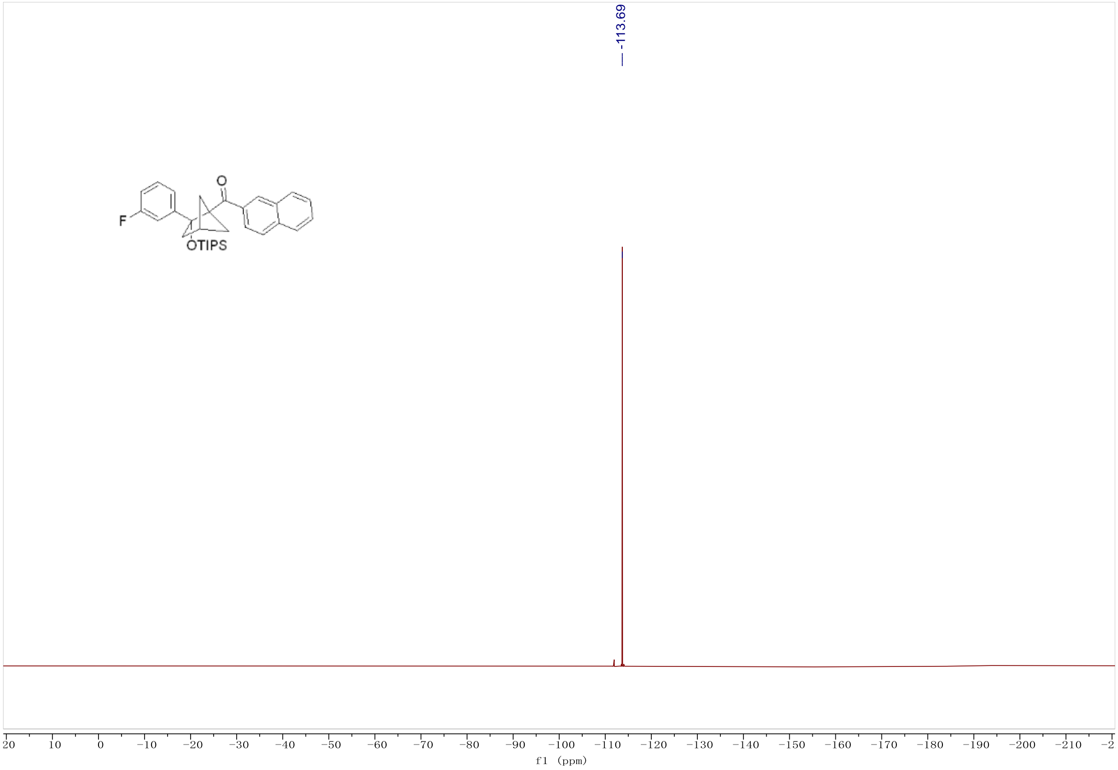

Supplementary Figure 65. ^{19}**F NMR** spectrum of (471 MHz, $CDCl_3$) of **3g**

Compound 3h

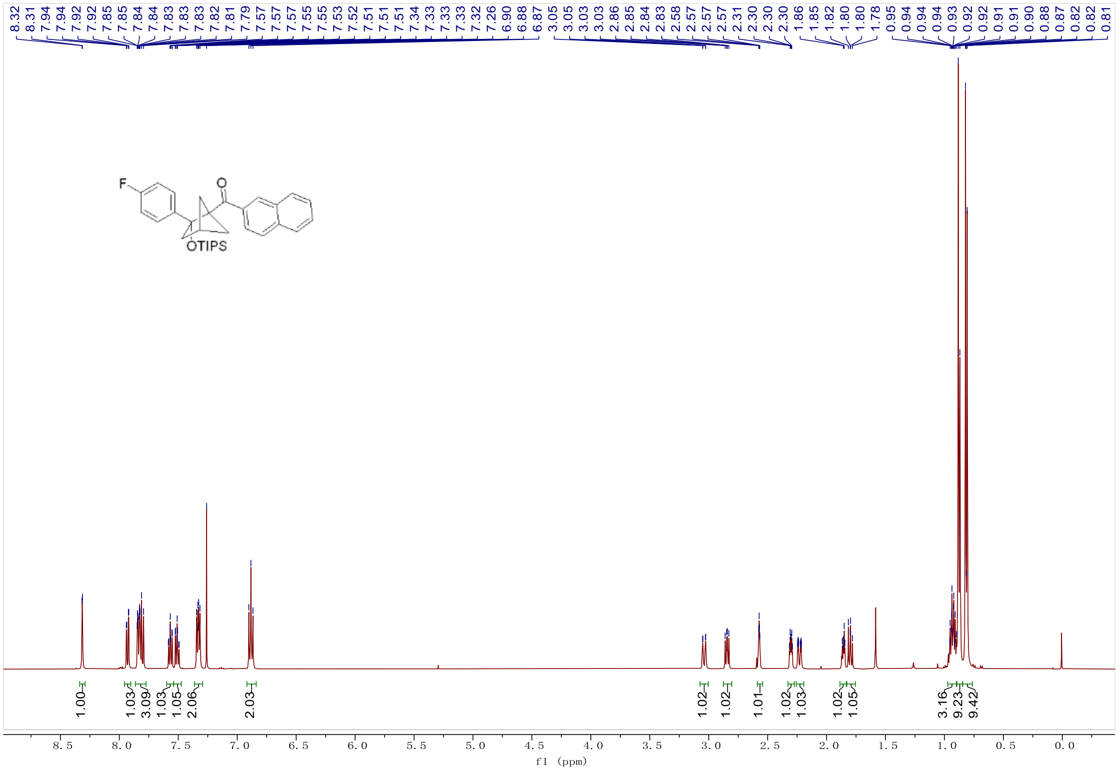

Supplementary Figure 66. ^{1}**H NMR** spectrum (500 MHz, $CDCl_3$) of **3h**

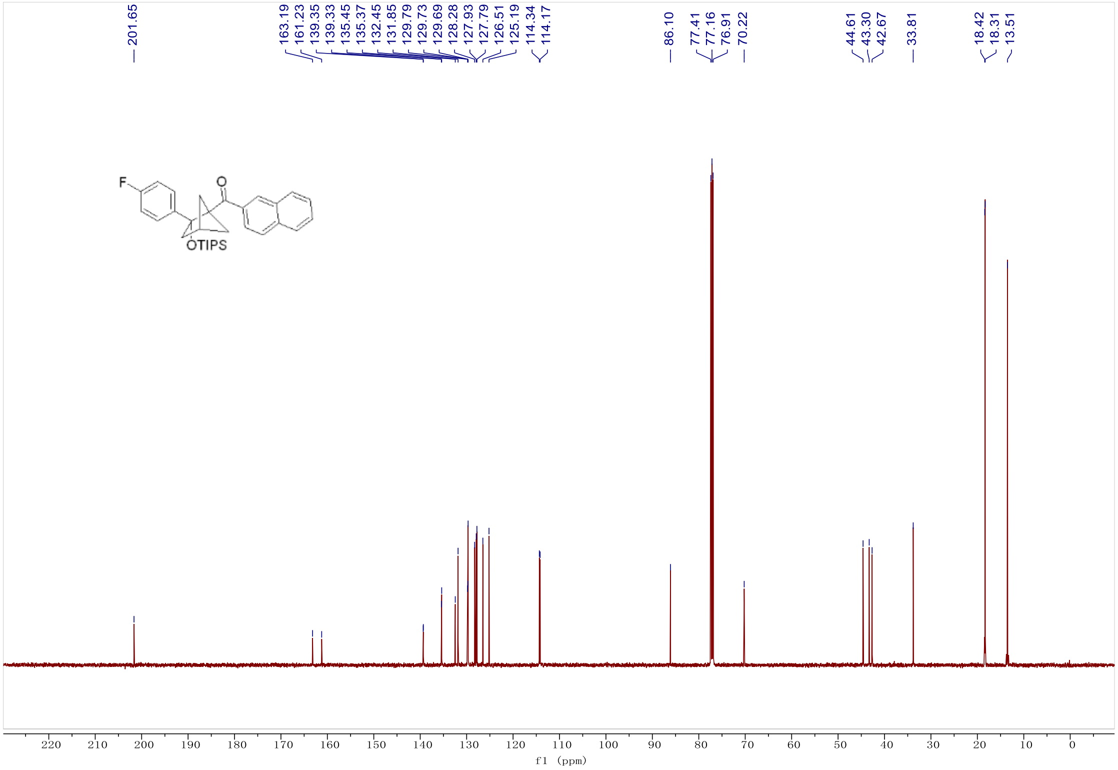

Supplementary Figure 67. ^{13}**C NMR** spectrum (125 MHz, $CDCl_3$) of **3h**

Compound 3h

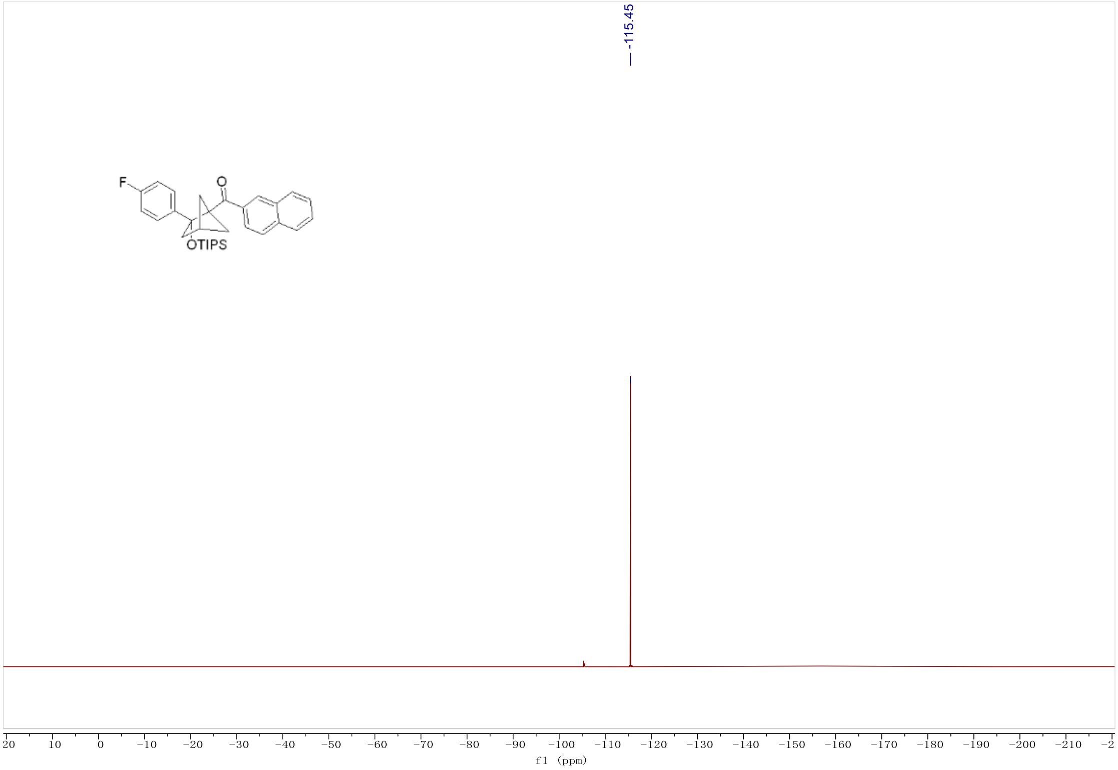

Supplementary Figure 68. ^{19}**F NMR** spectrum of (471 MHz, $CDCl_3$) of **3h**

Compound 3i

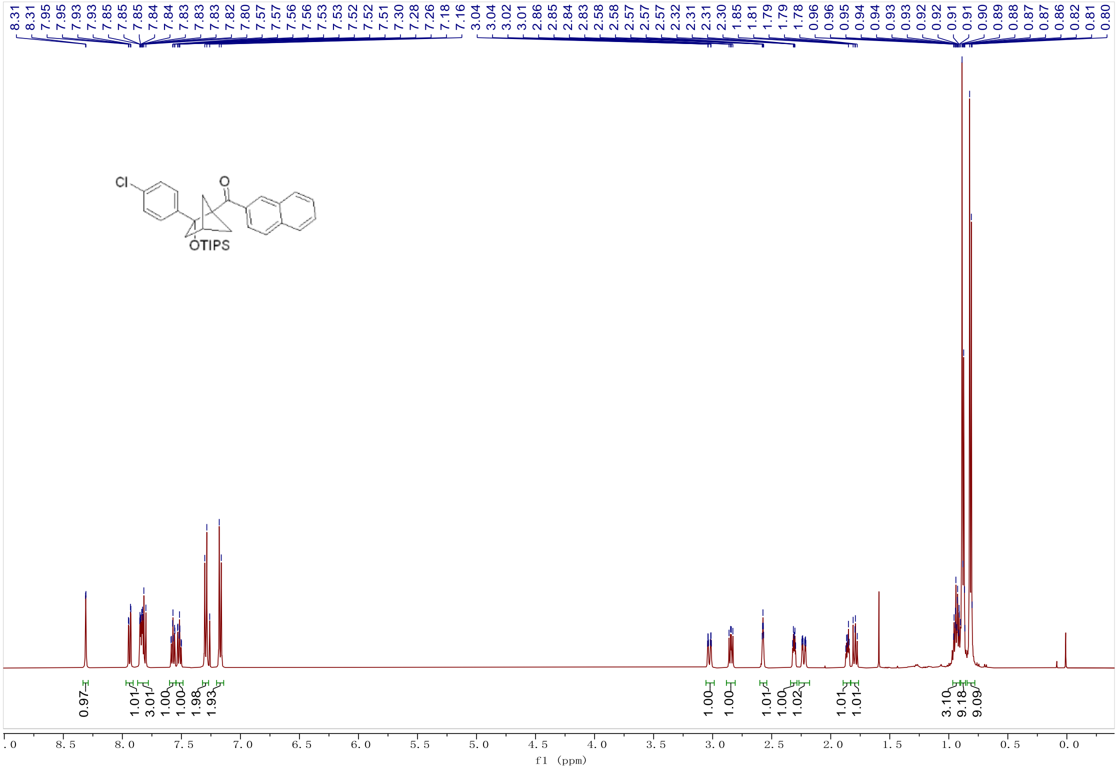

Supplementary Figure 69. ^{1}**H NMR** spectrum (500 MHz, $CDCl_3$) of **3i**

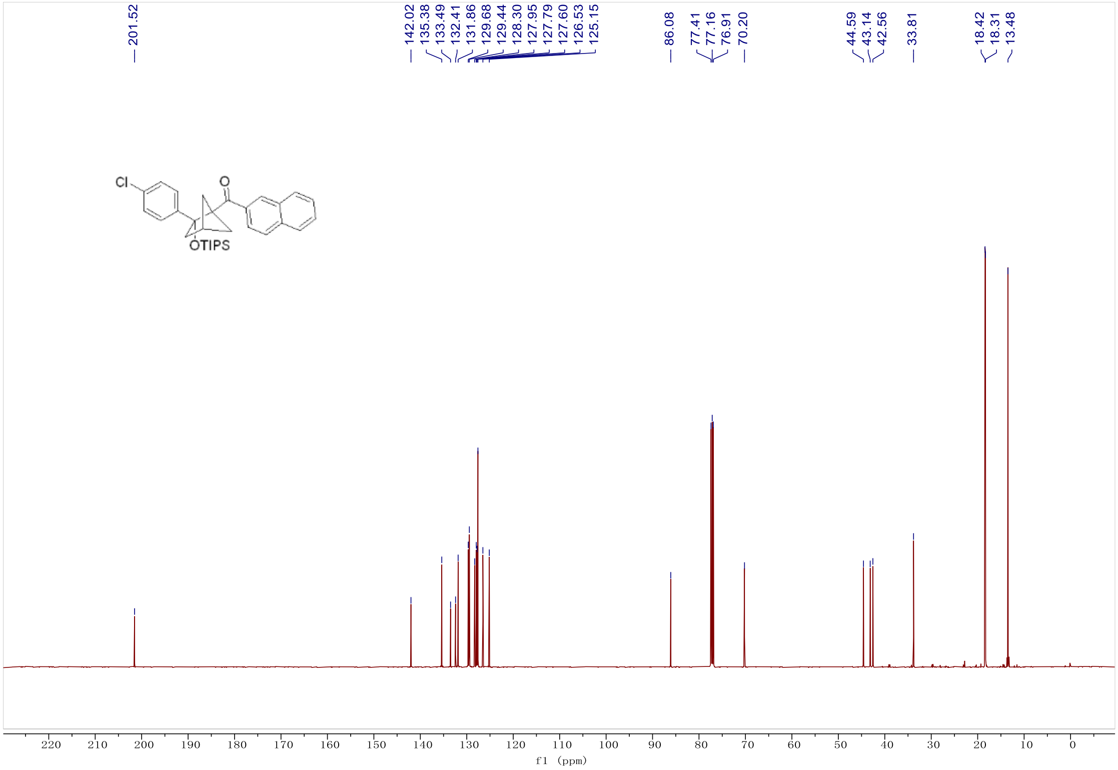

Supplementary Figure 70. ^{13}**C NMR** spectrum (125 MHz, $CDCl_3$) of **3i**

Compound 3j

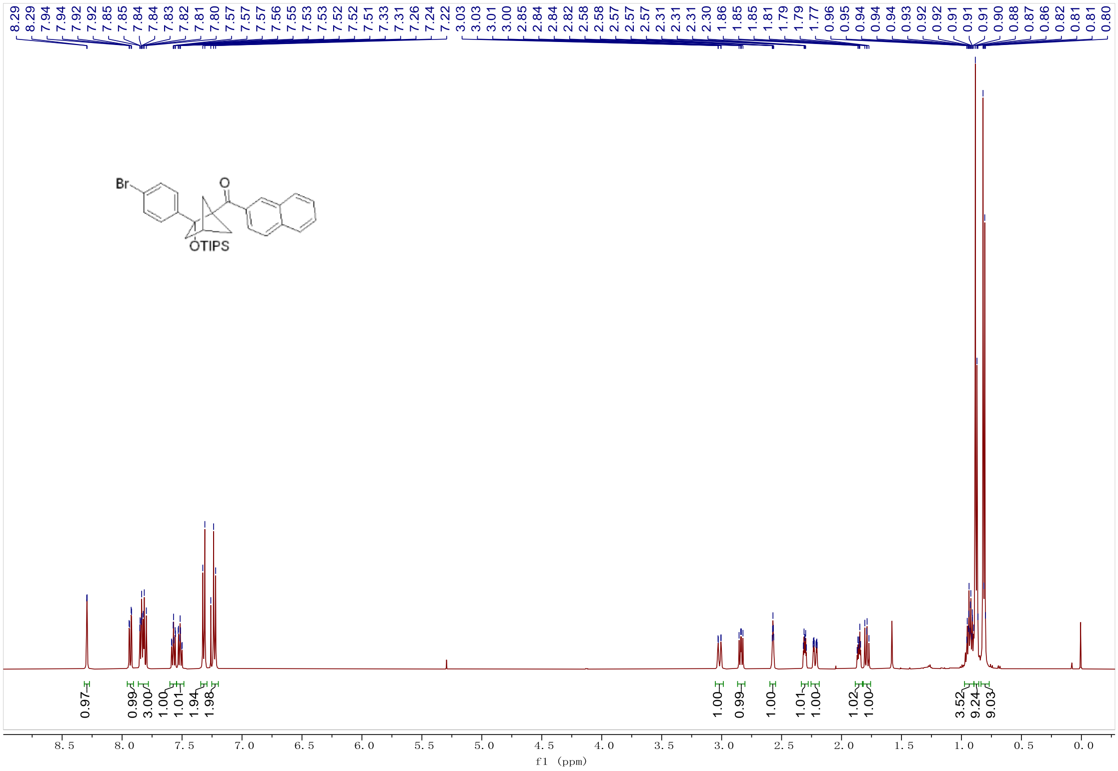

Supplementary Figure 71. ^{1}H NMR spectrum (500 MHz, $CDCl_3$) of **3j**

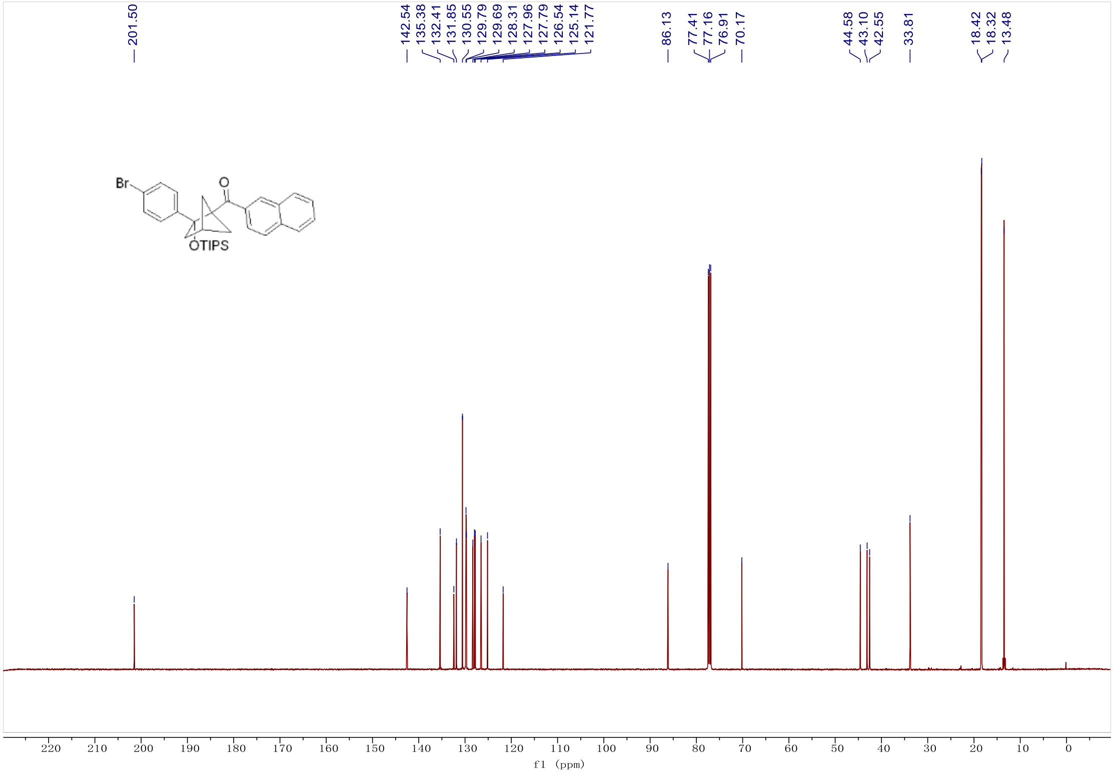

Supplementary Figure 72. ^{13}C NMR spectrum (125 MHz, $CDCl_3$) of **3j**

Compound 3k

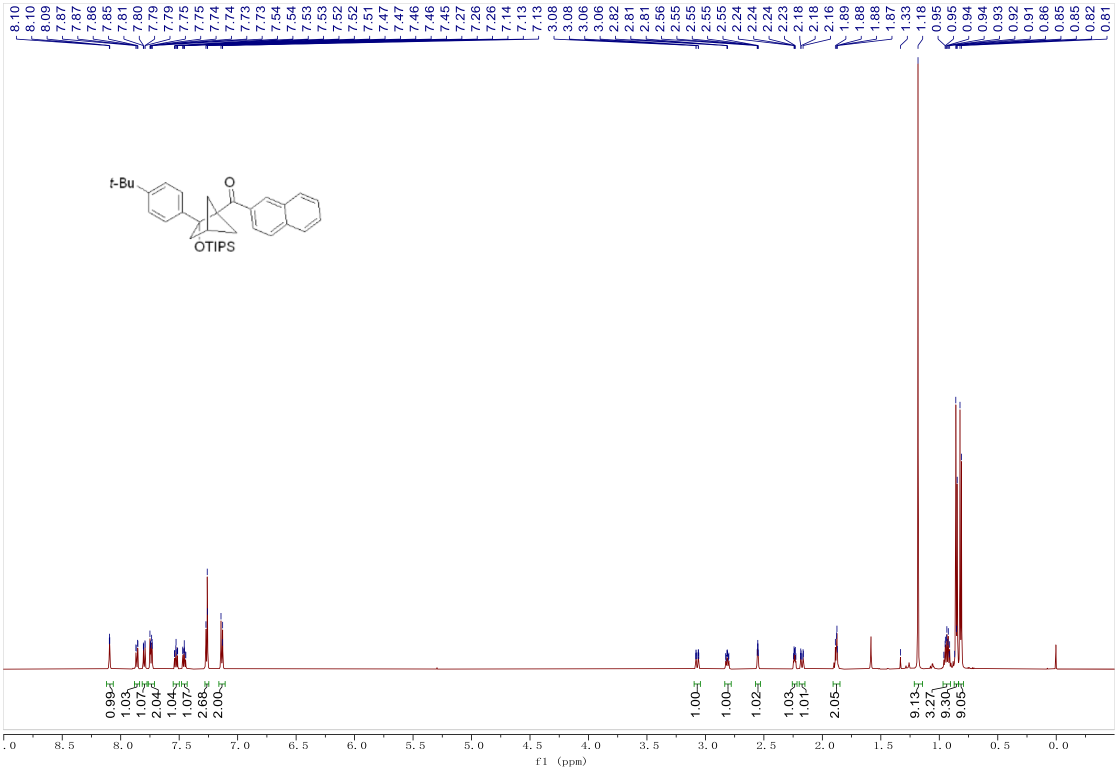

Supplementary Figure 73. ^{1}**H NMR** spectrum (600 MHz, $CDCl_3$) of **3k**

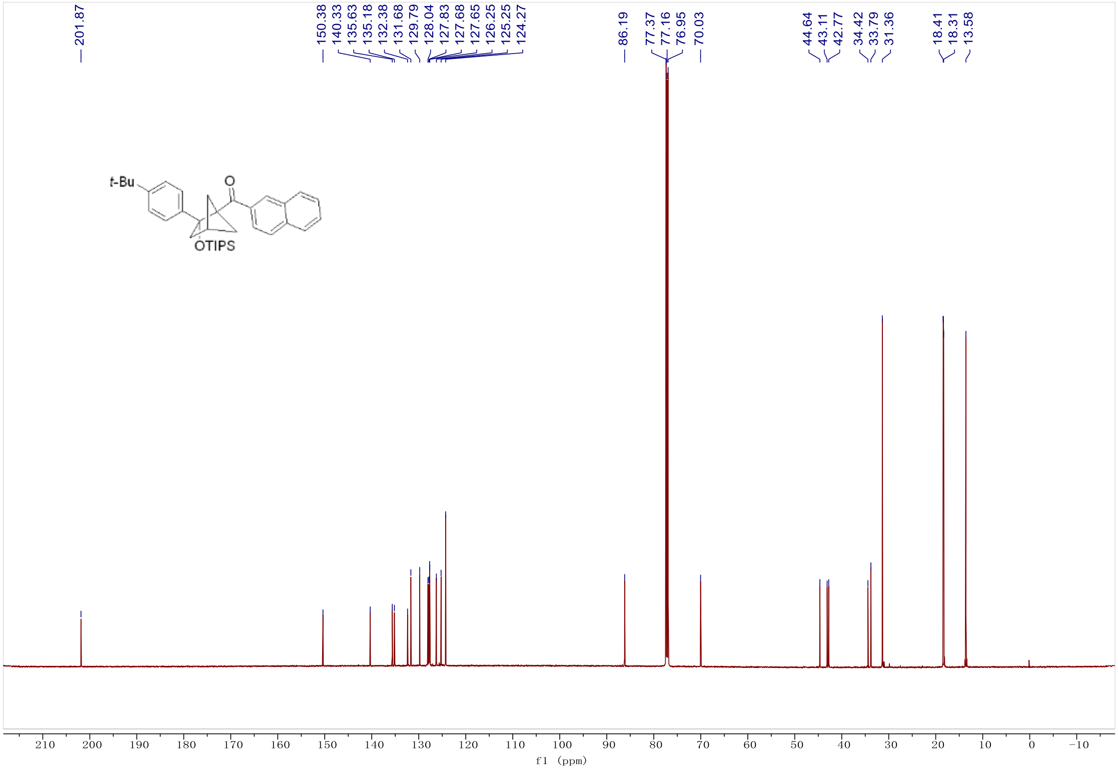

Supplementary Figure 74. ^{13}**C NMR** spectrum (150 MHz, $CDCl_3$) of **3k**

Compound 3l

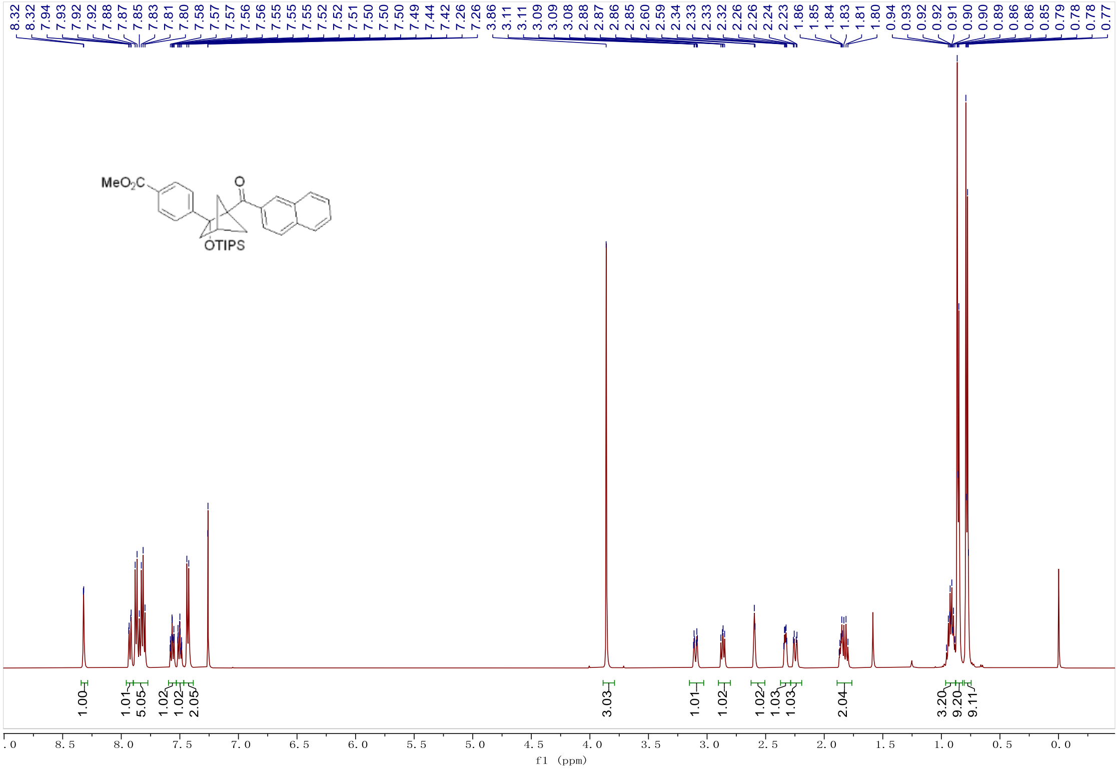

Supplementary Figure 75. ^{1}**H NMR** spectrum (500 MHz, $CDCl_3$) of **3l**

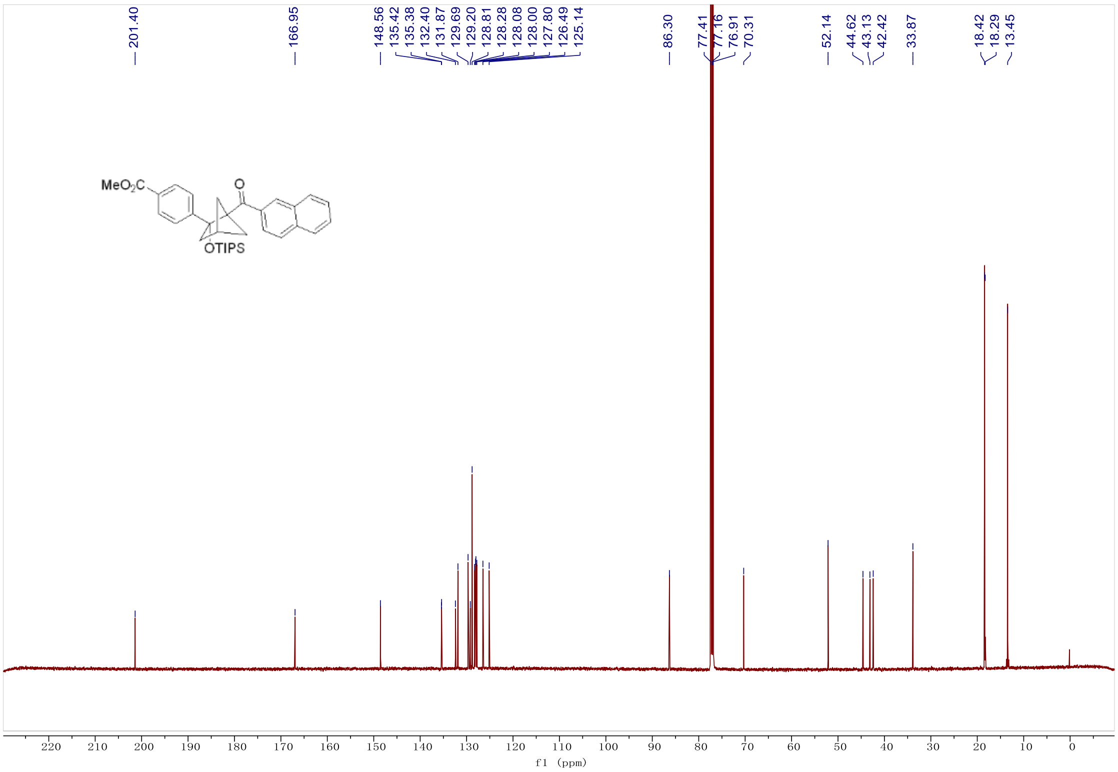

Supplementary Figure 76. ^{13}**C NMR** spectrum (125 MHz, $CDCl_3$) of **3l**

Compound 3m

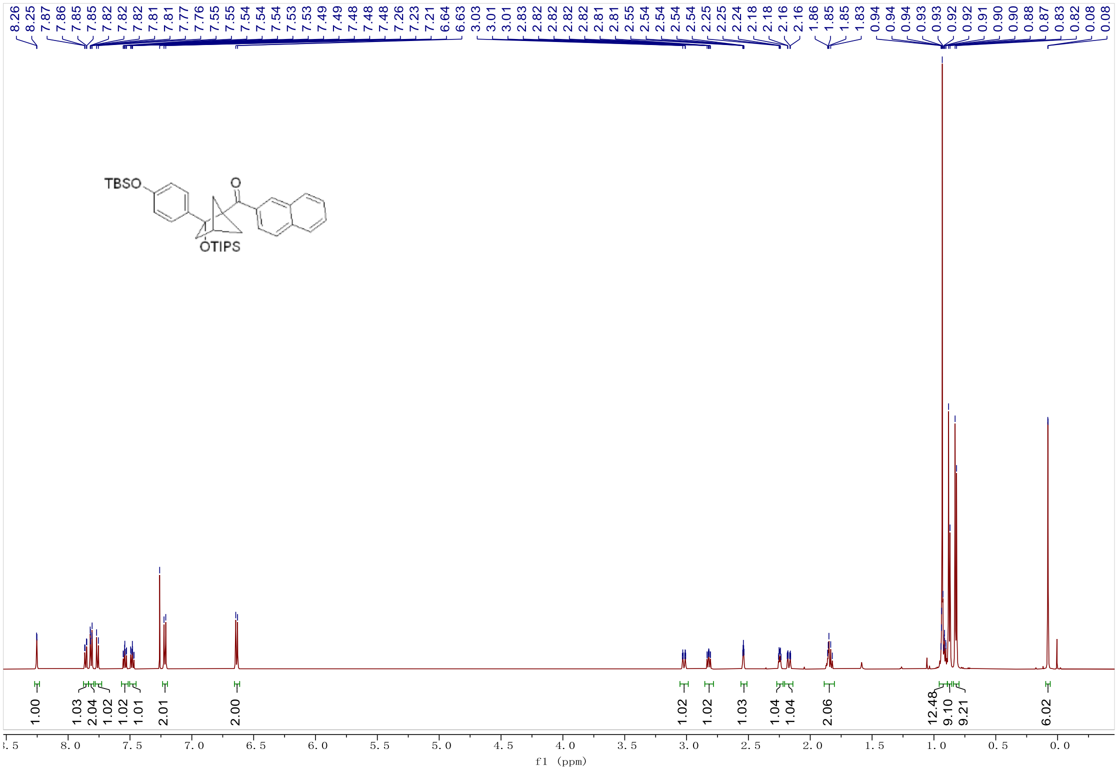

Supplementary Figure 77. ^{1}H NMR spectrum (600 MHz, $CDCl_3$) of **3m**

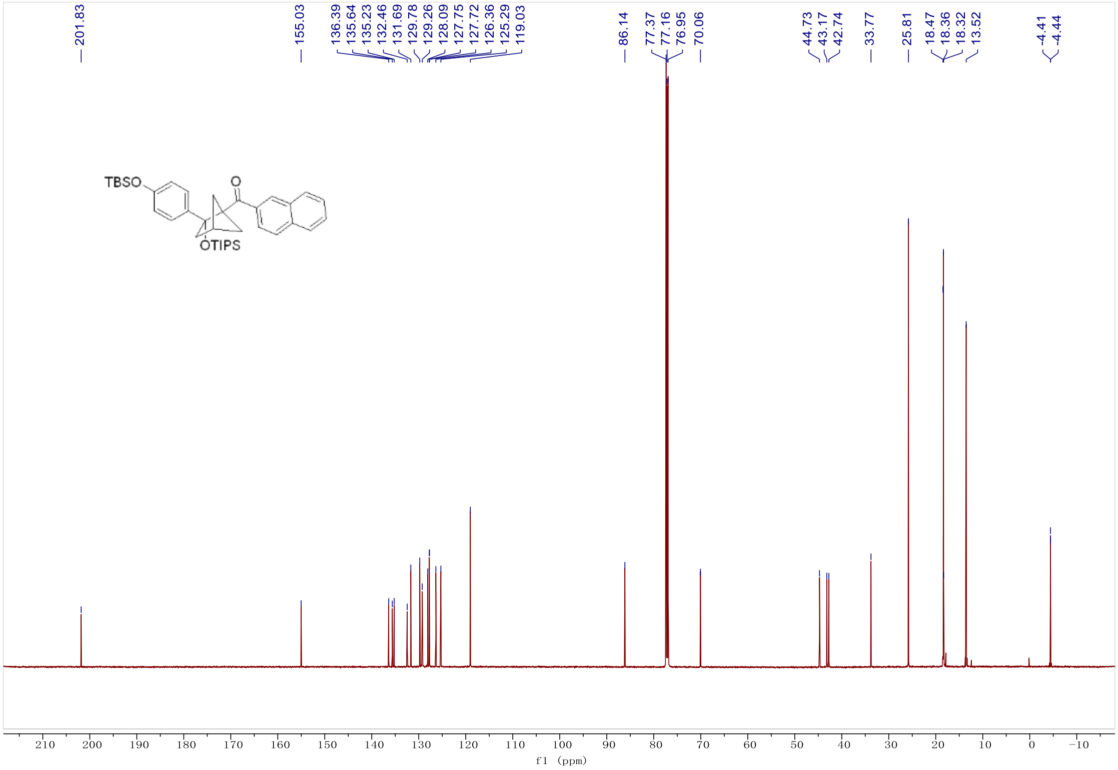

Supplementary Figure 78. ^{13}C NMR spectrum (150 MHz, $CDCl_3$) of **3m**

Compound 3n

^{1}H NMR (600 MHz, $CDCl_3$)

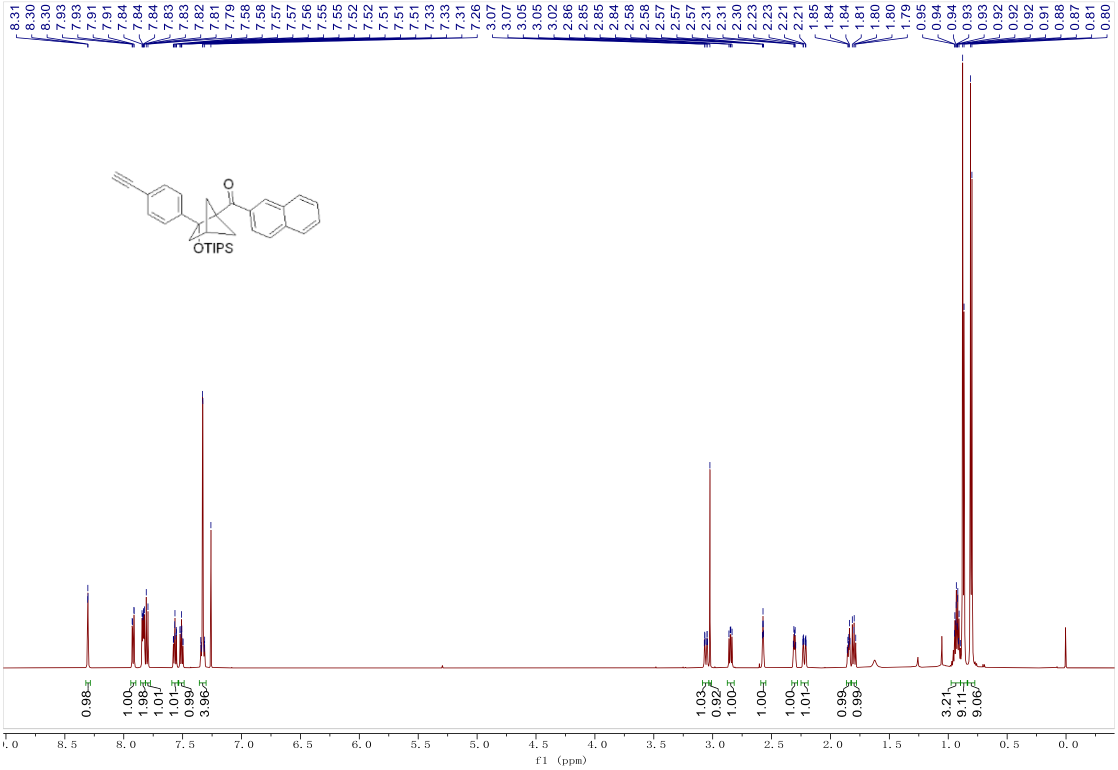

Supplementary Figure 79. ^{1}H NMR spectrum (600 MHz, $CDCl_3$) of **3n**

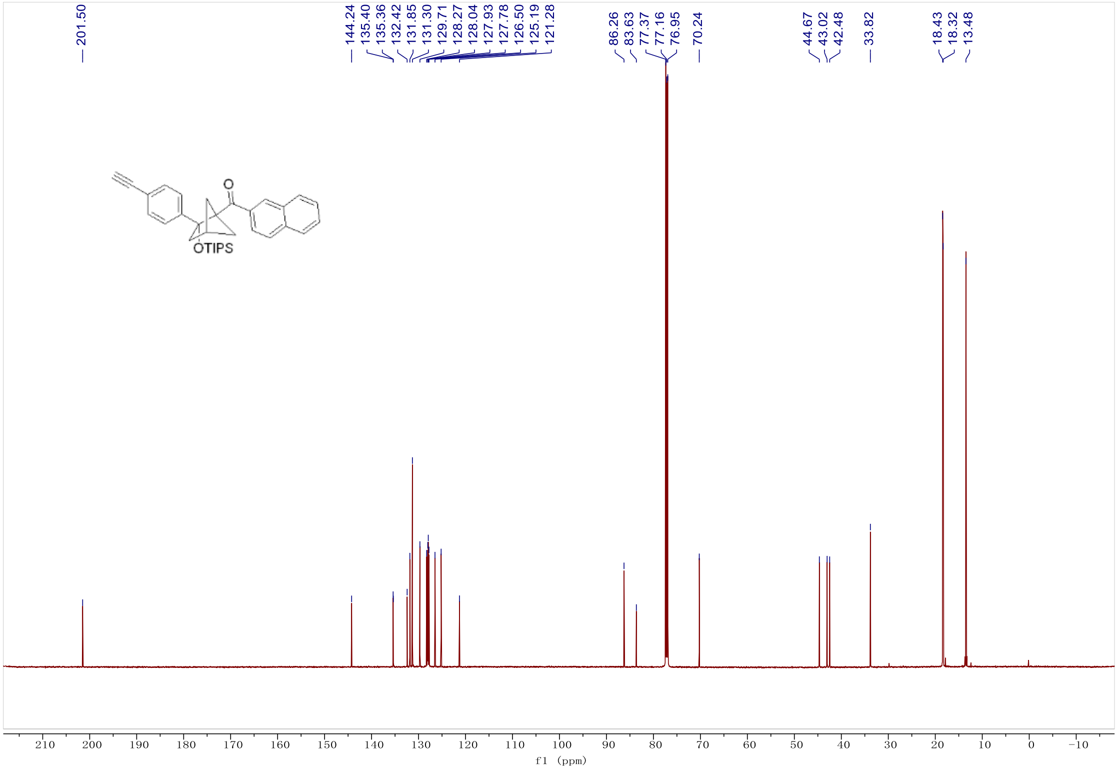

Supplementary Figure 80. ^{13}C NMR spectrum (150 MHz, $CDCl_3$) of **3n**

Compound 3o

^{1}H NMR (500 MHz, $CDCl_3$)

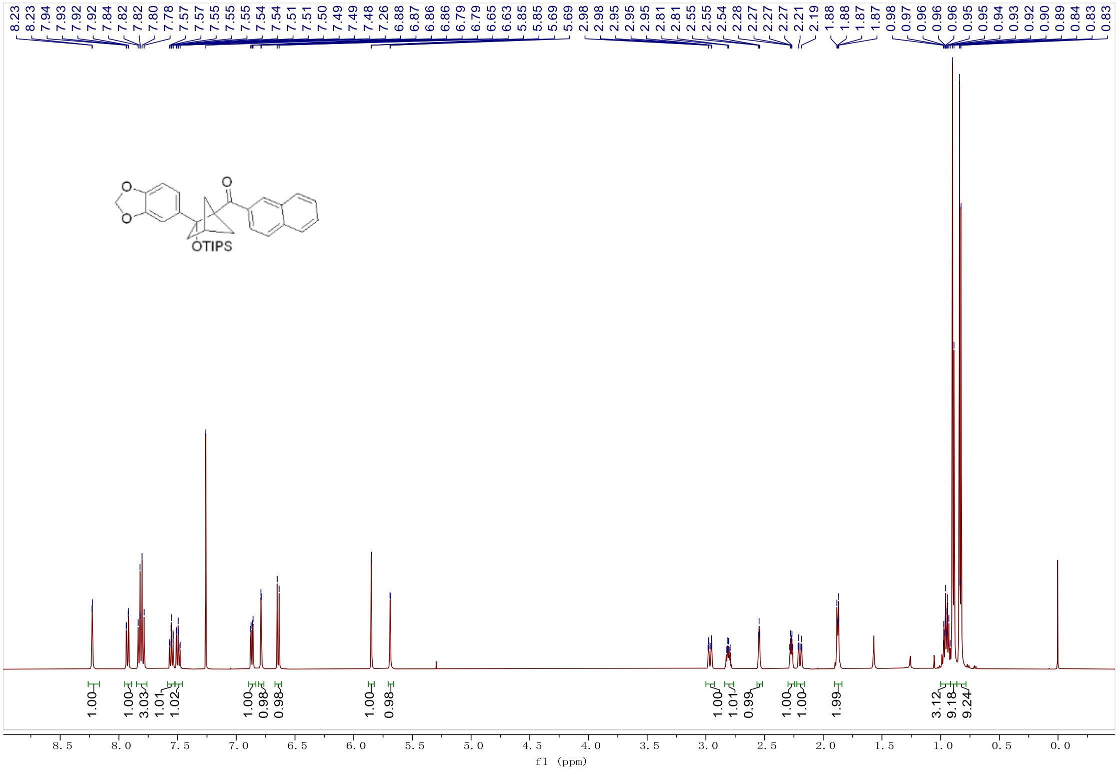

Supplementary Figure 81. **^{1}H NMR** spectrum (500 MHz, $CDCl_3$) of **3o**

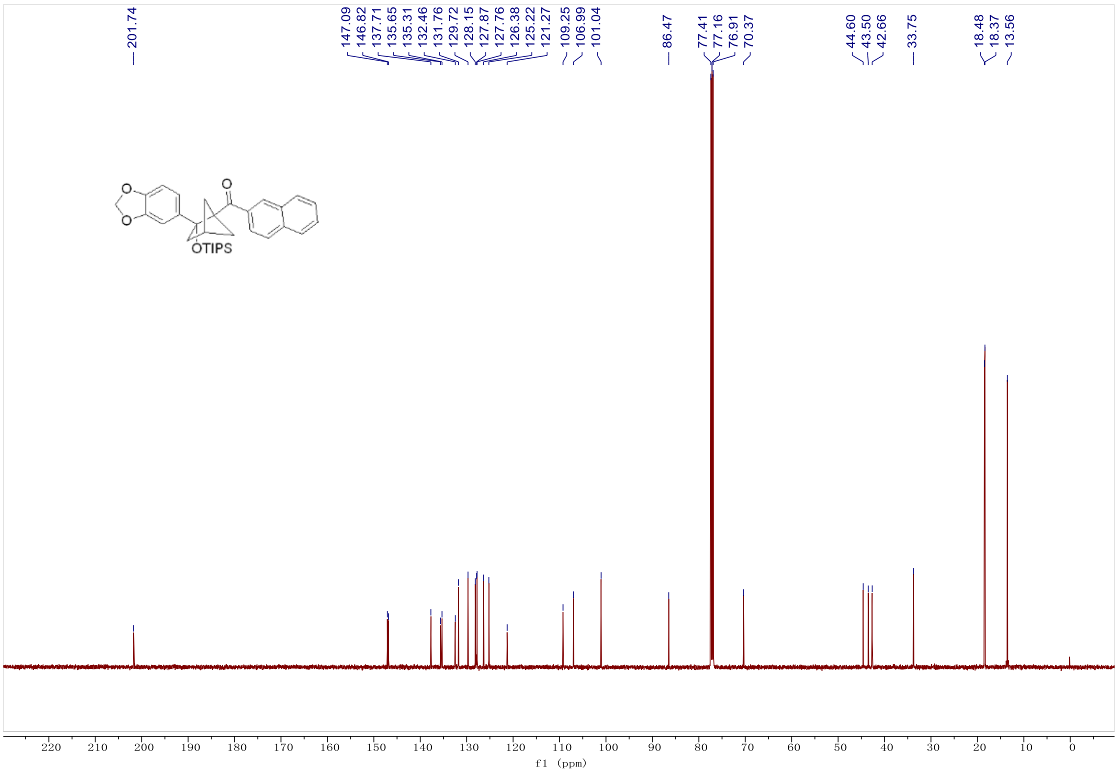

Supplementary Figure 82. **^{13}C NMR** spectrum (125 MHz, $CDCl_3$) of **3o**

Compound 3p

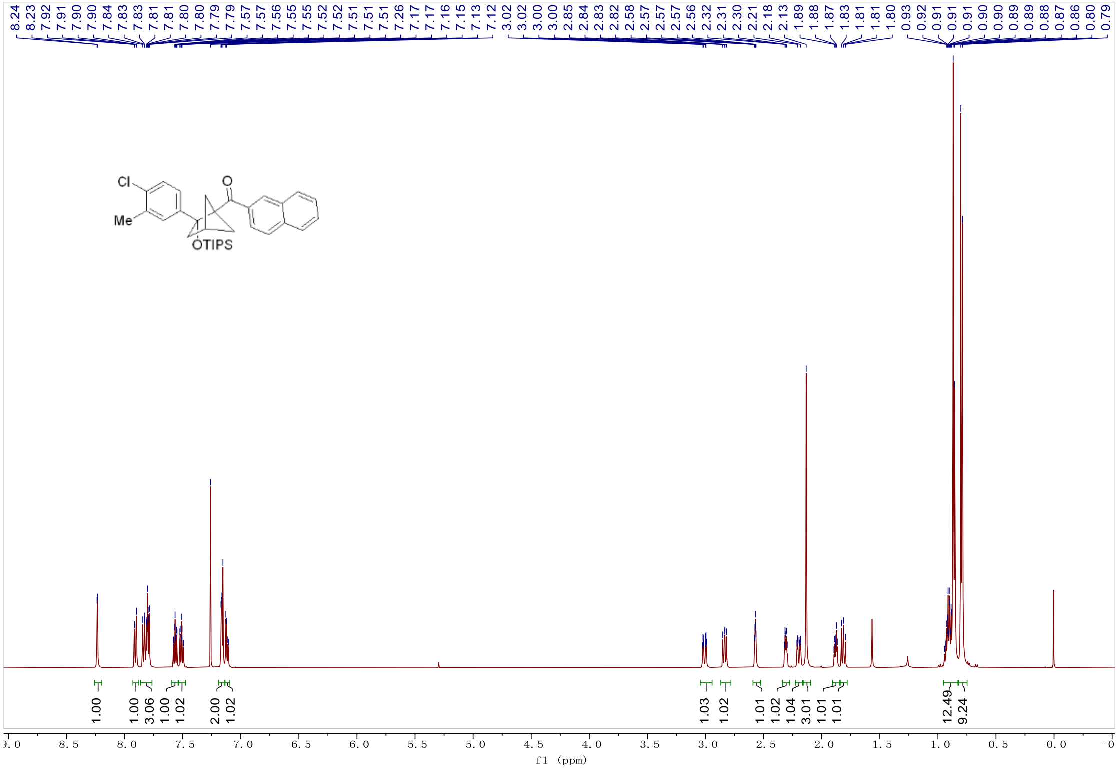

Supplementary Figure 83. ^{1}**H NMR** spectrum (500 MHz, $CDCl_3$) of **3p**

^{13}**C NMR** (125 MHz, $CDCl_3$)

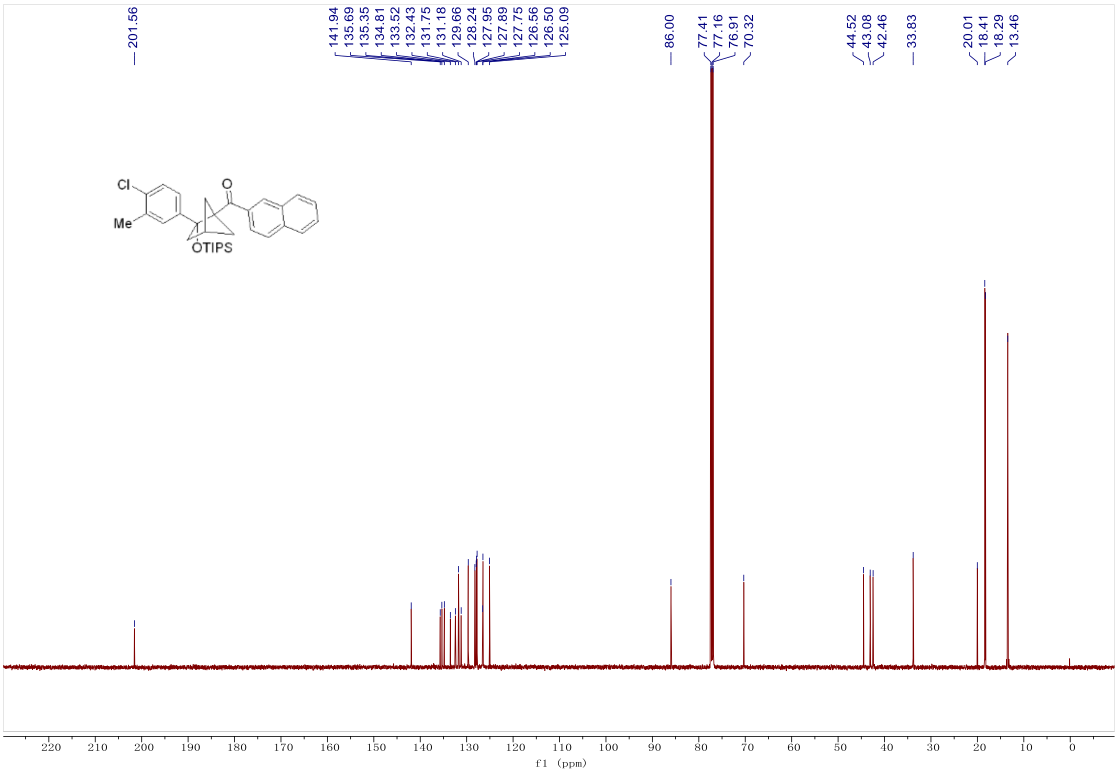

Supplementary Figure 84. ^{13}**C NMR** spectrum (125 MHz, $CDCl_3$) of **3p**

Compound 3q

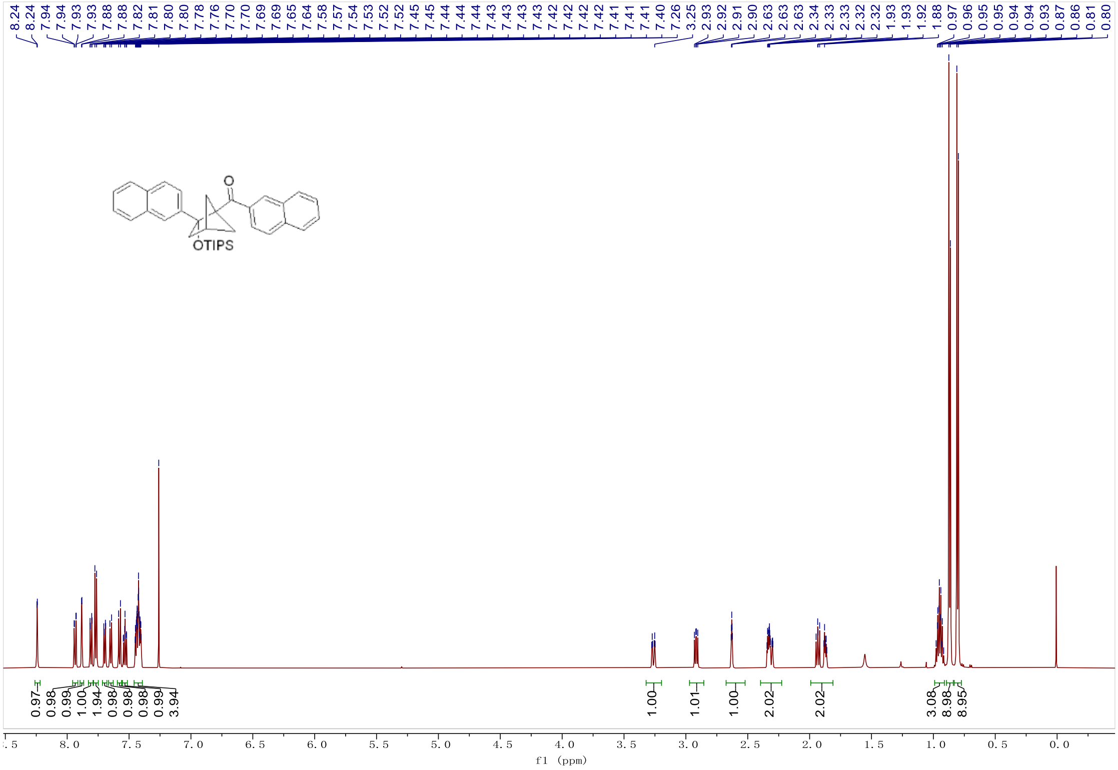

Supplementary Figure 85. ^{1}H NMR spectrum (600 MHz, $CDCl_3$) of **3q**

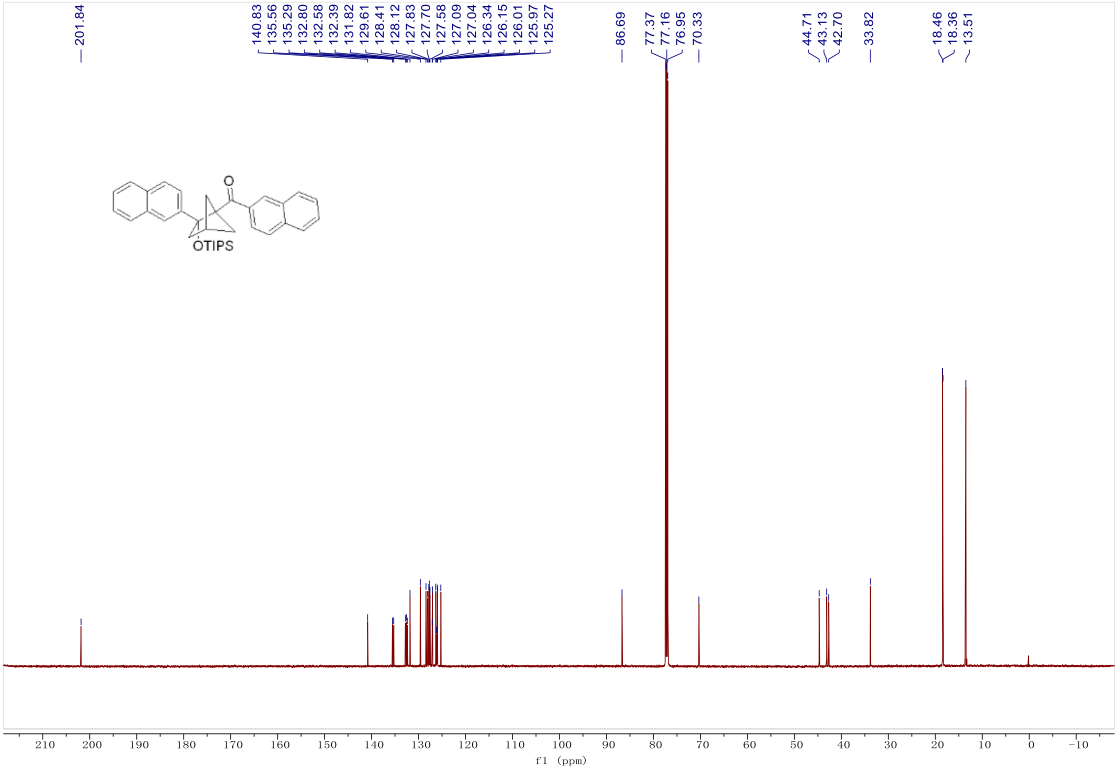

Supplementary Figure 86. ^{13}C NMR spectrum (150 MHz, $CDCl_3$) of **3q**

Compound 3r

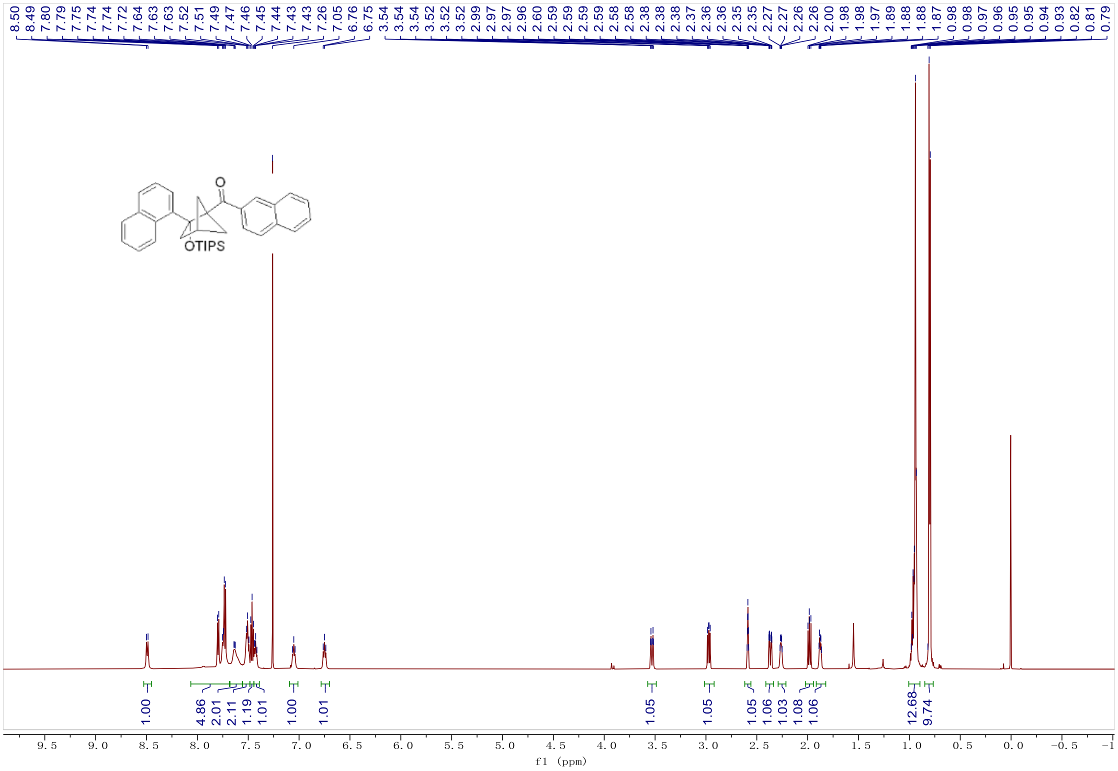

Supplementary Figure 87. ^{1}**H NMR** spectrum (600 MHz, $CDCl_3$) of **3r**

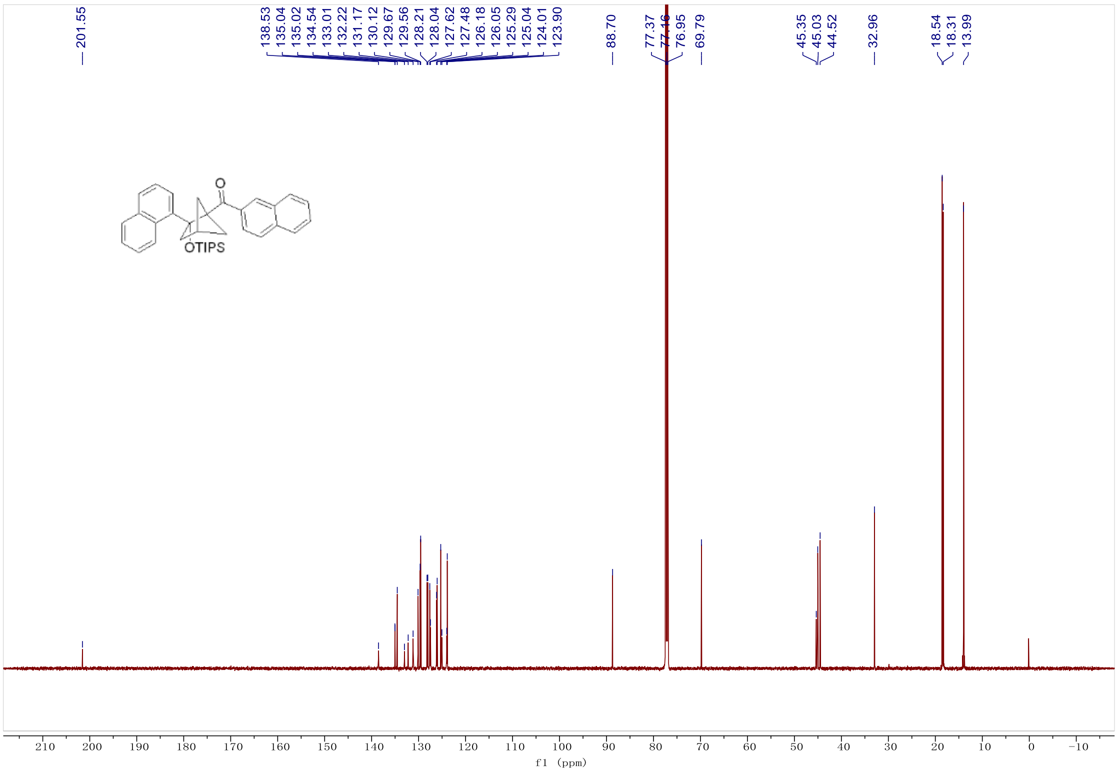

Supplementary Figure 88. ^{13}**C NMR** spectrum (150 MHz, $CDCl_3$) of **3r**

Compound 3s

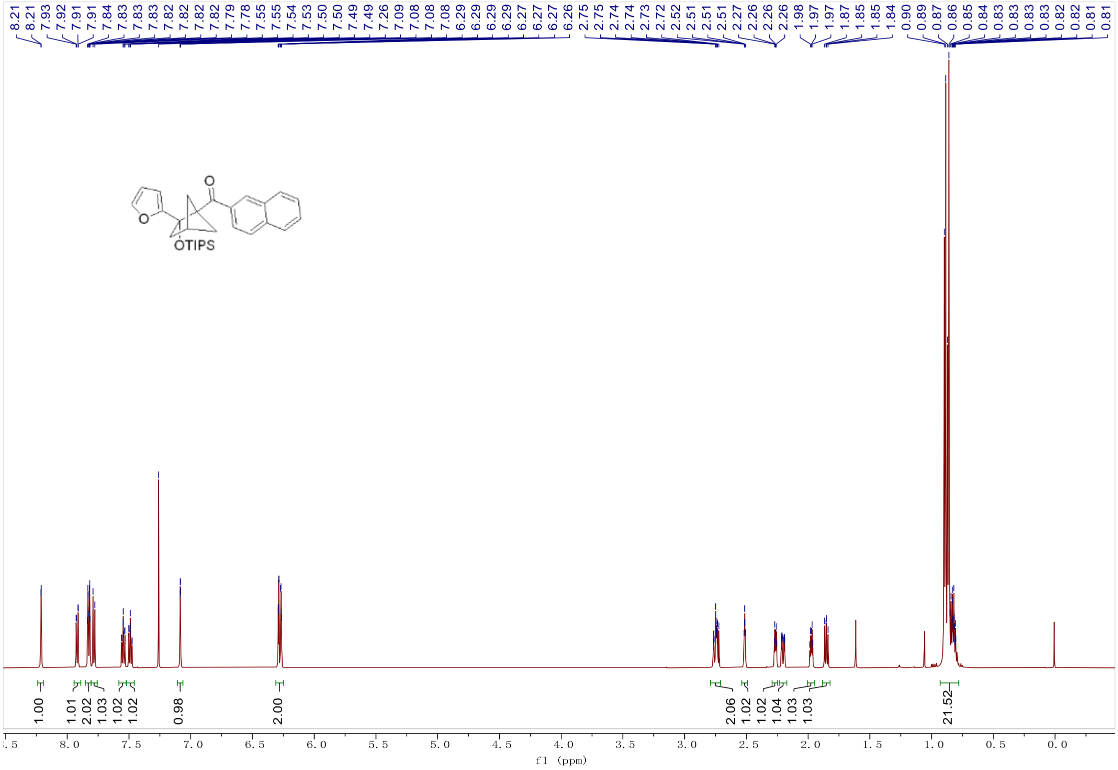

Supplementary Figure 89. ^{1}**H NMR** spectrum (600 MHz, $CDCl_3$) of **3s**

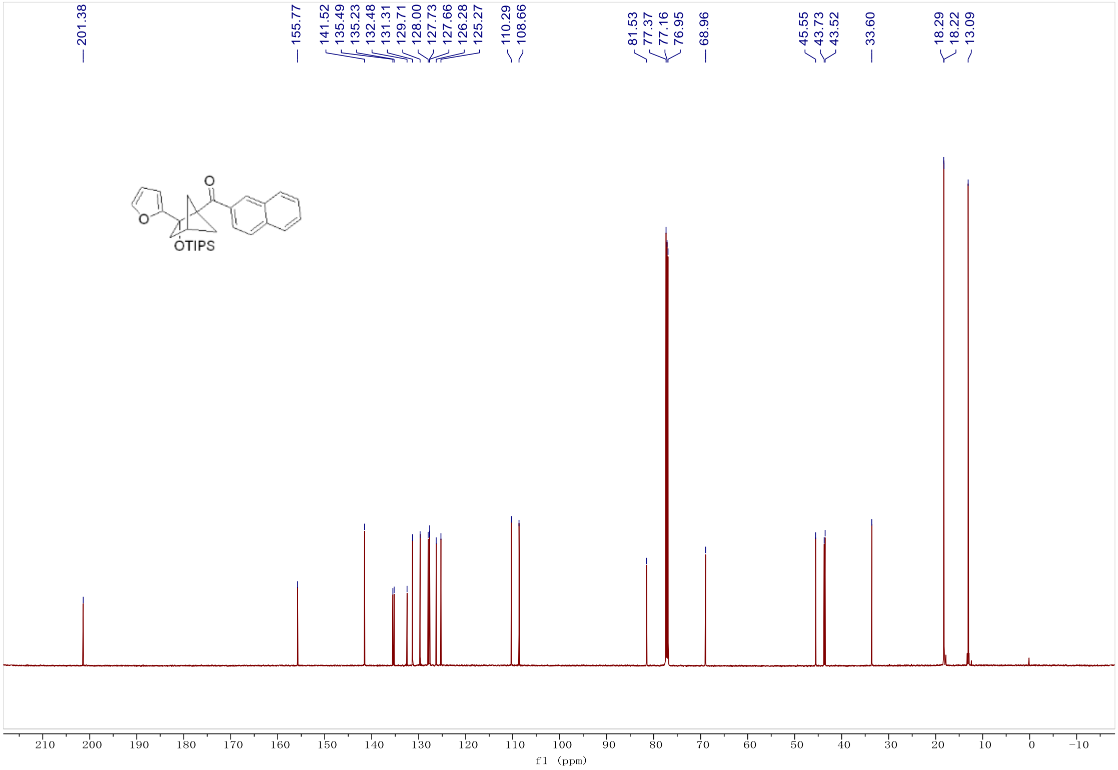

Supplementary Figure 90. ^{13}**C NMR** spectrum (150 MHz, $CDCl_3$) of **3s**

Compound 3t

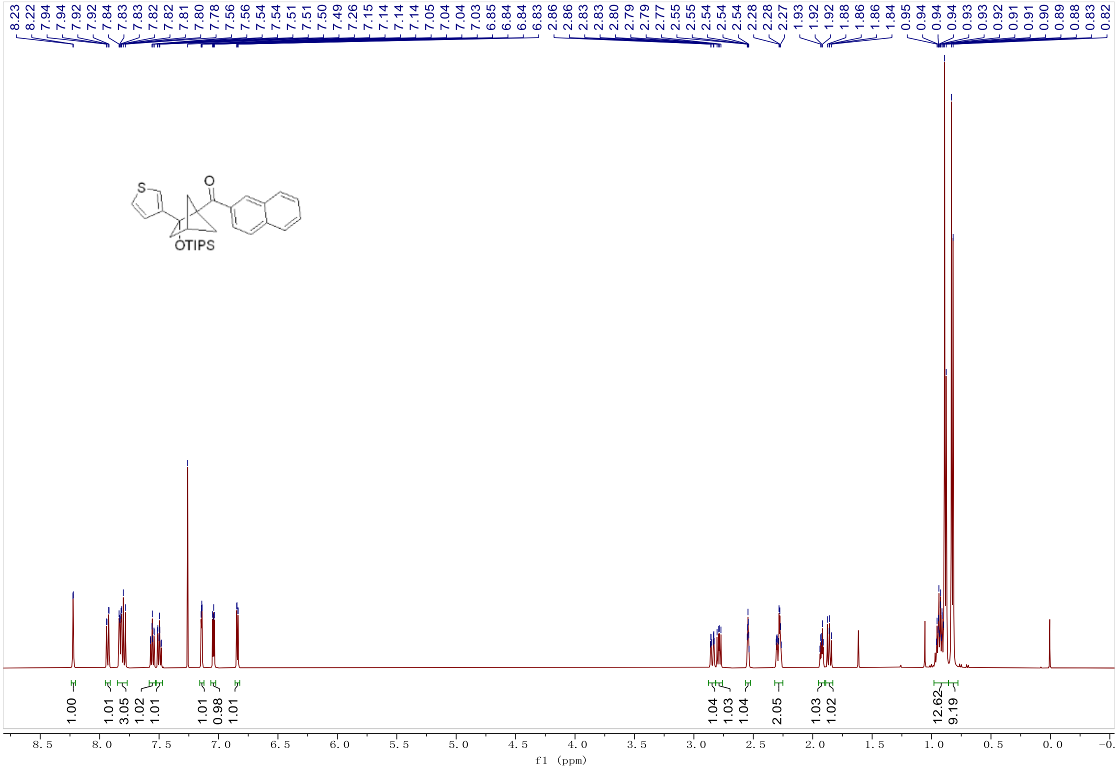

Supplementary Figure 91. ^{1}**H NMR** spectrum (500 MHz, $CDCl_3$) of **3t**

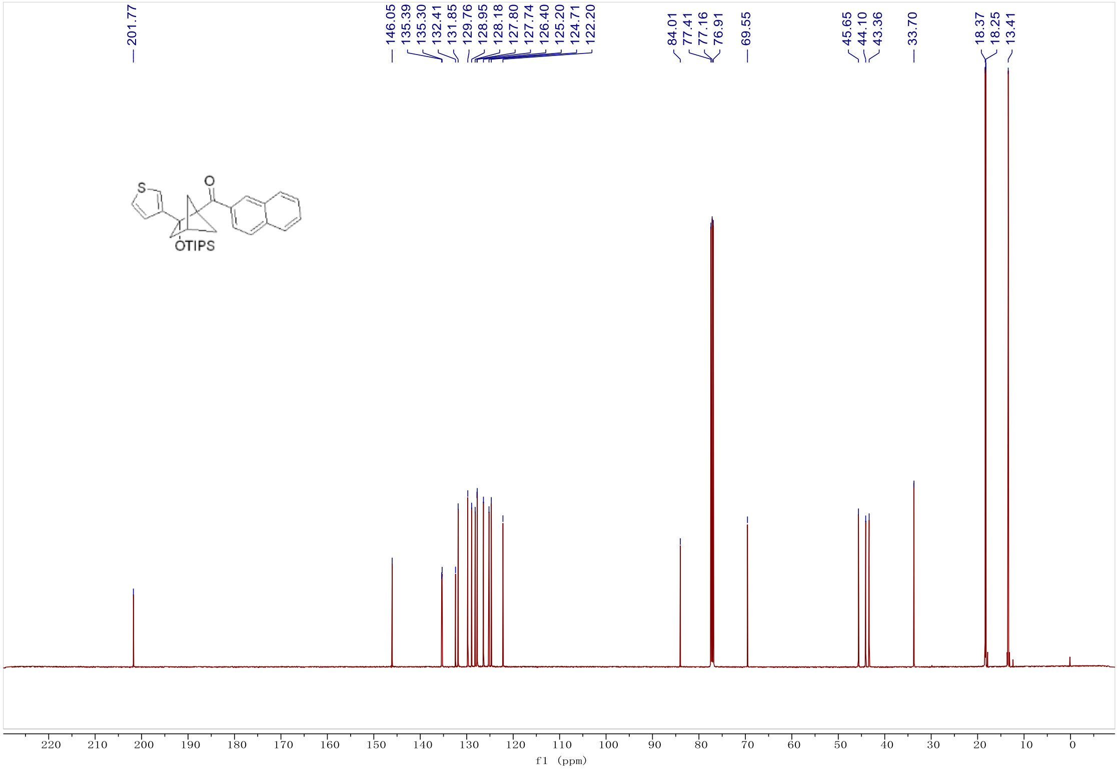

Supplementary Figure 92. ^{13}**C NMR** spectrum (125 MHz, $CDCl_3$) of **3t**

Compound 3u

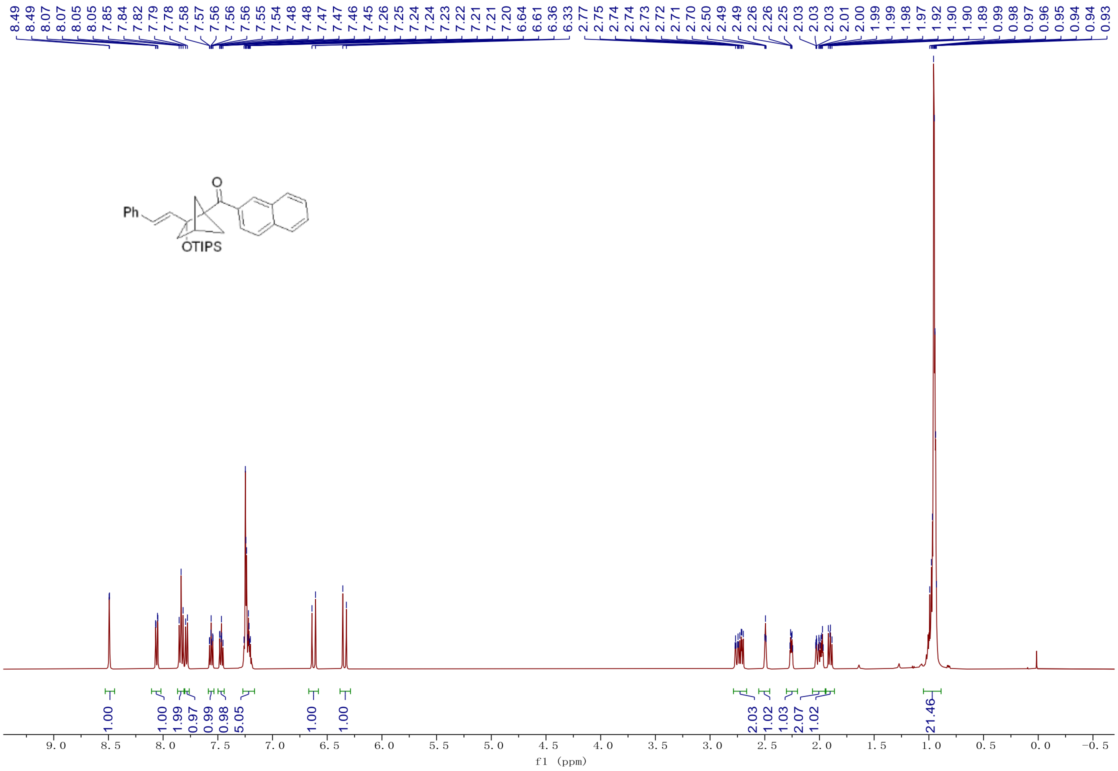

Supplementary Figure 93. ^{1}H NMR spectrum (500 MHz, $CDCl_3$) of **3u**

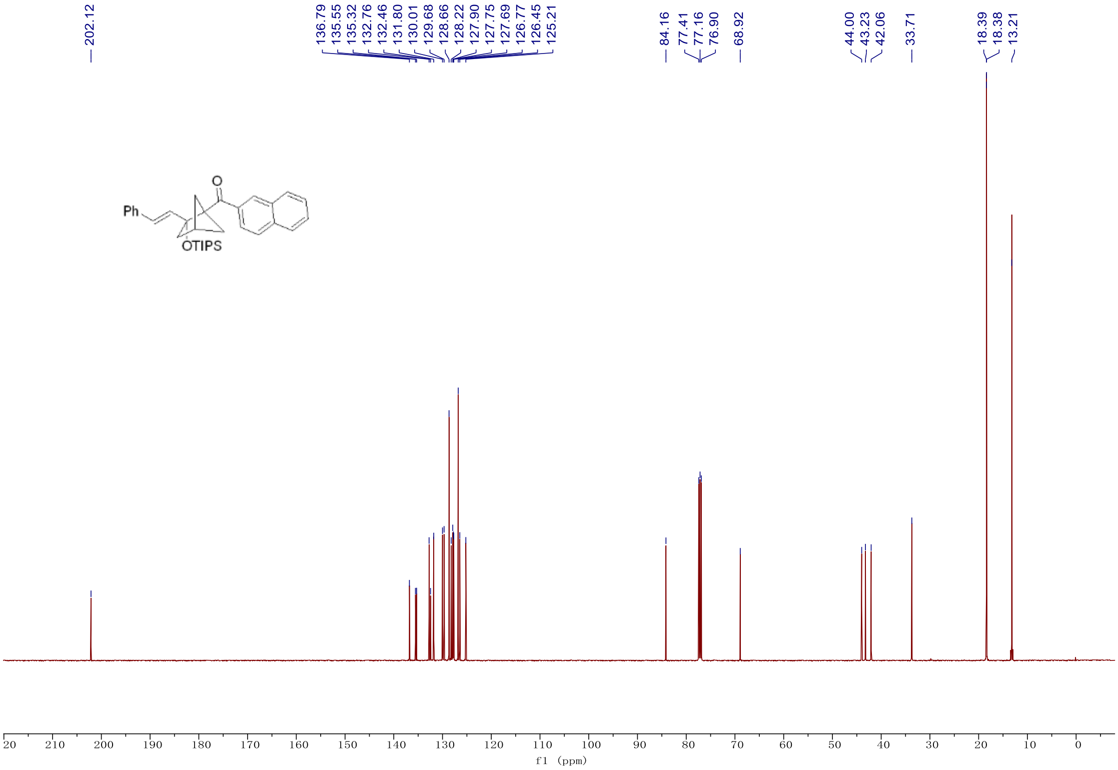

Supplementary Figure 94. ^{13}C NMR spectrum (125 MHz, $CDCl_3$) of **3u**

Compound 3v

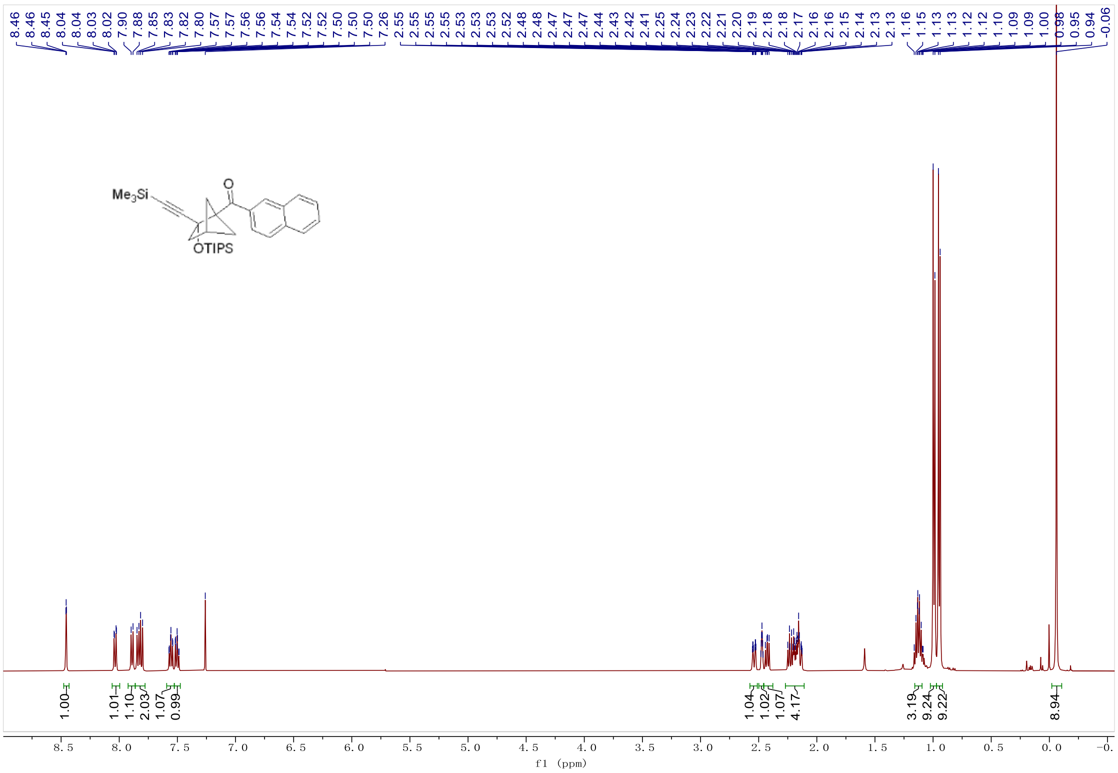

Supplementary Figure 95. ^{1}H NMR spectrum (500 MHz, $CDCl_3$) of **3v**

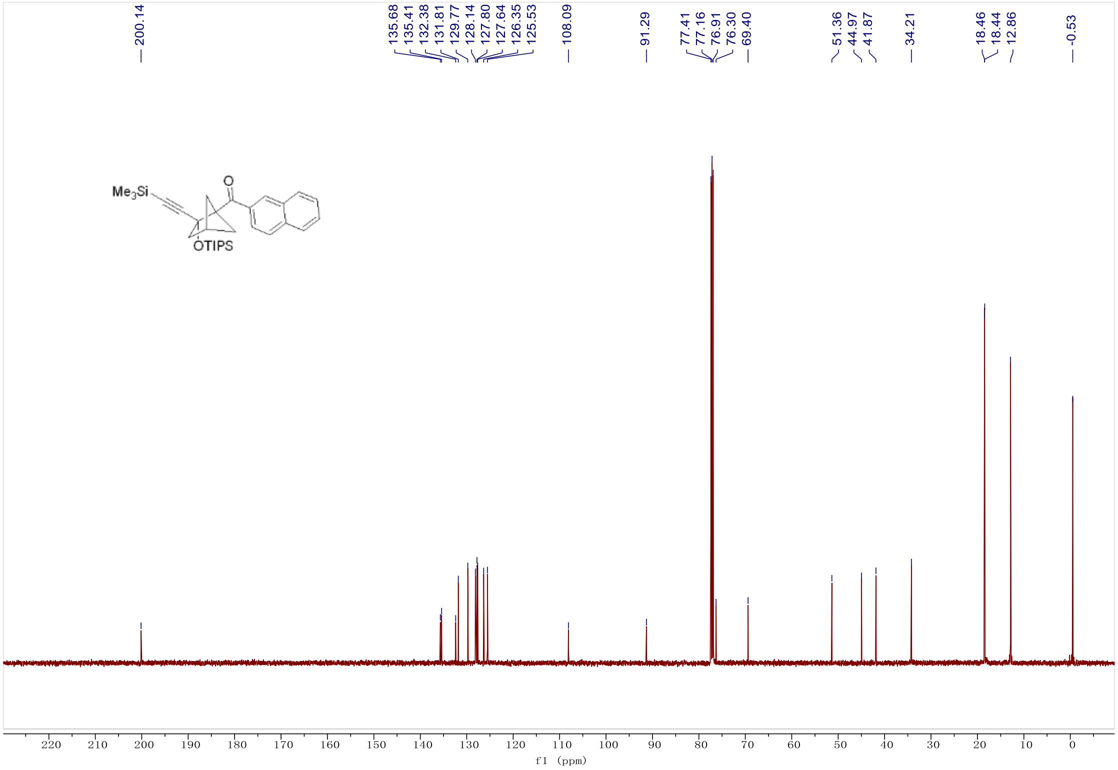

Supplementary Figure 96. ^{13}C NMR spectrum (125 MHz, $CDCl_3$) of **3v**

Compound 3w

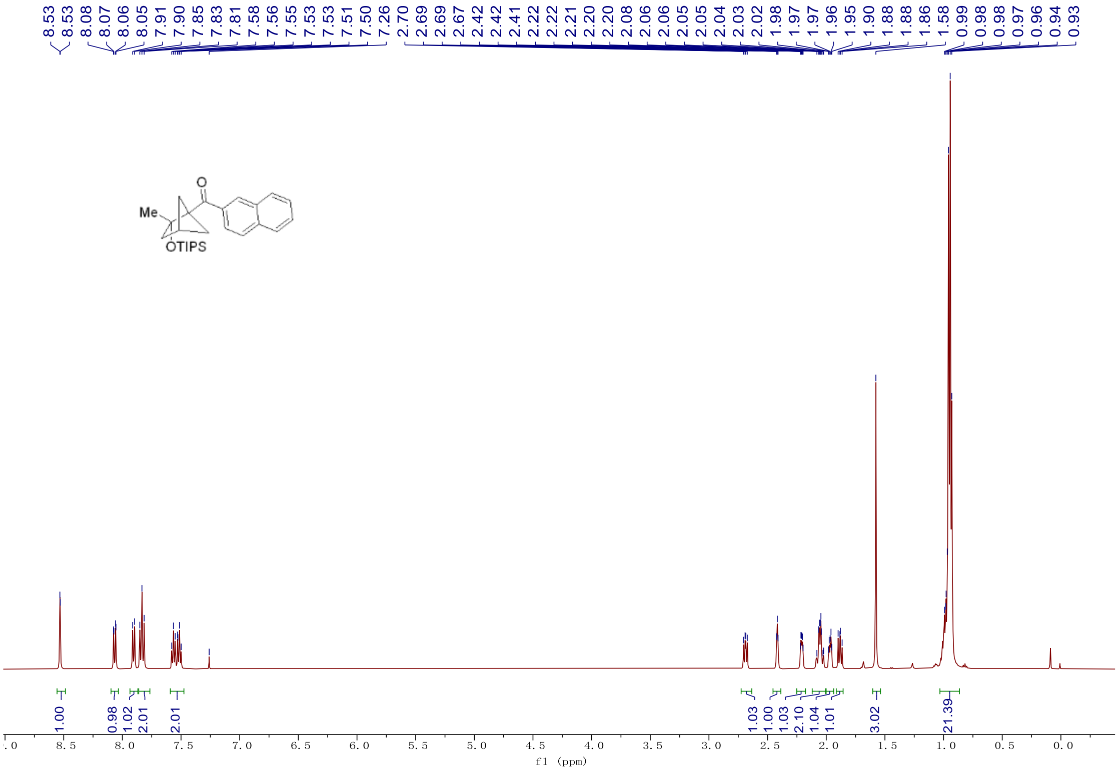

Supplementary Figure 97. **^{1}H NMR** spectrum (500 MHz, $CDCl_3$) of **3w**

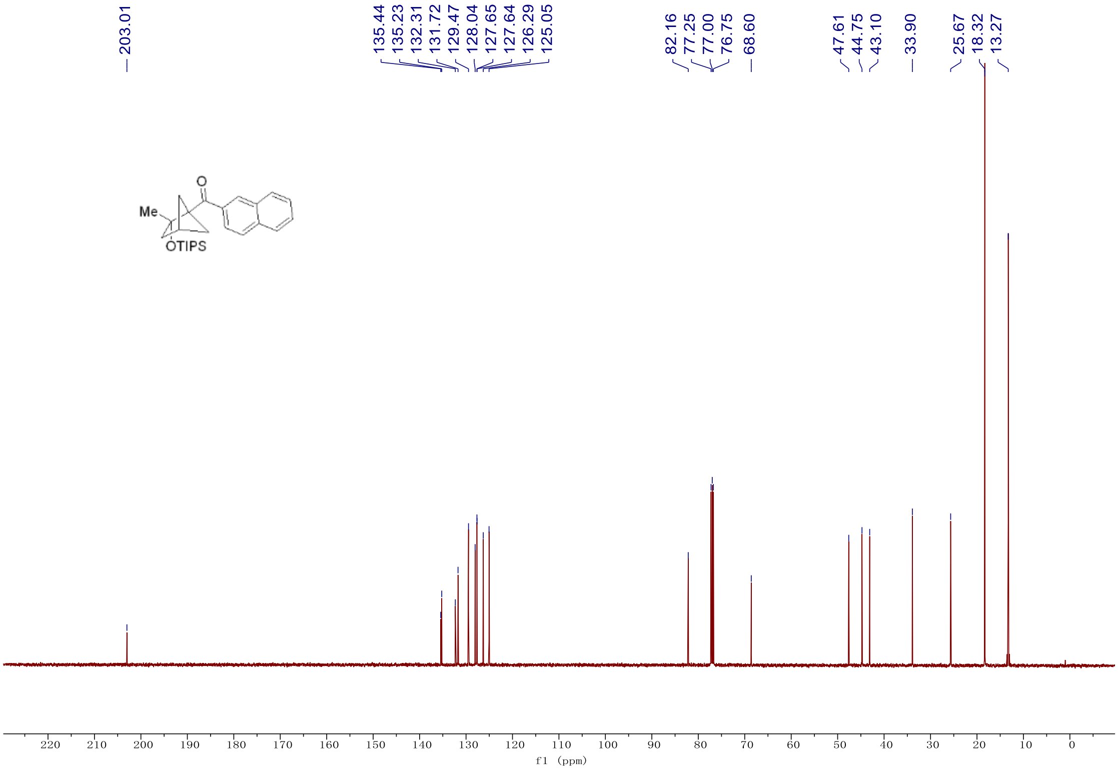

Supplementary Figure 98. **^{13}C NMR** spectrum (125 MHz, $CDCl_3$) of **3w**

Compound 3x

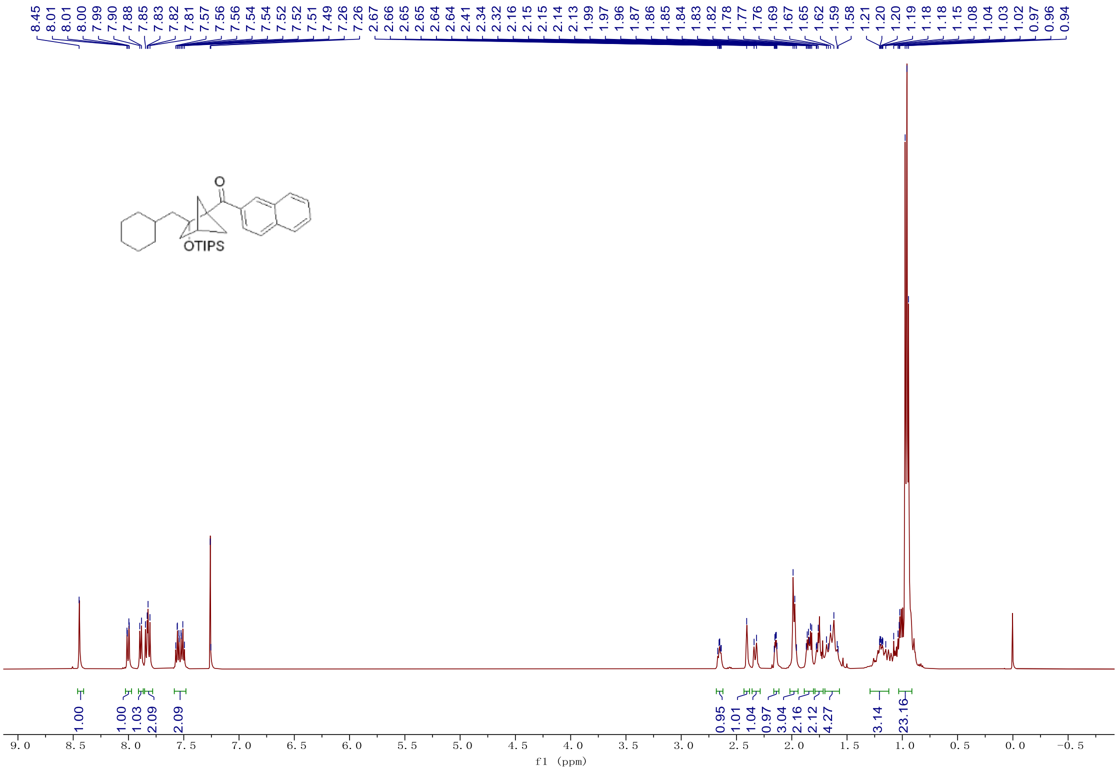

Supplementary Figure 99. ^{1}**H NMR** spectrum (500 MHz, $CDCl_3$) of **3x**

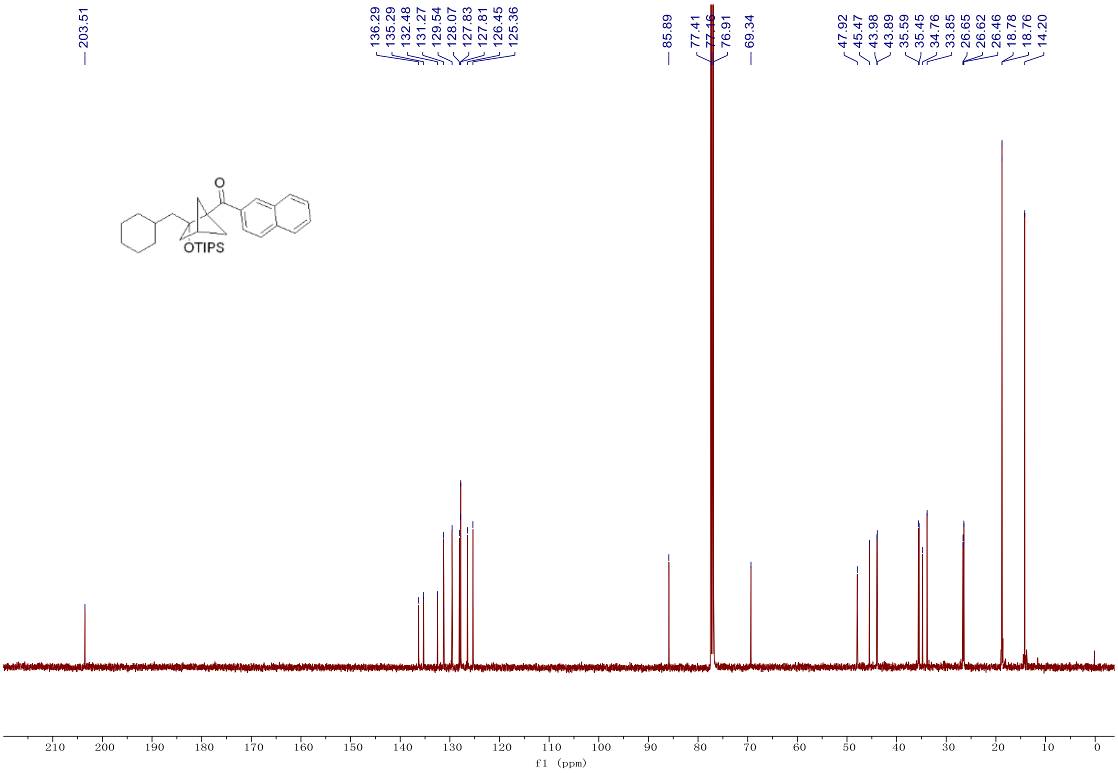

Supplementary Figure 100. ^{13}**C NMR** spectrum (125 MHz, $CDCl_3$) of **3x**

Compound 3y

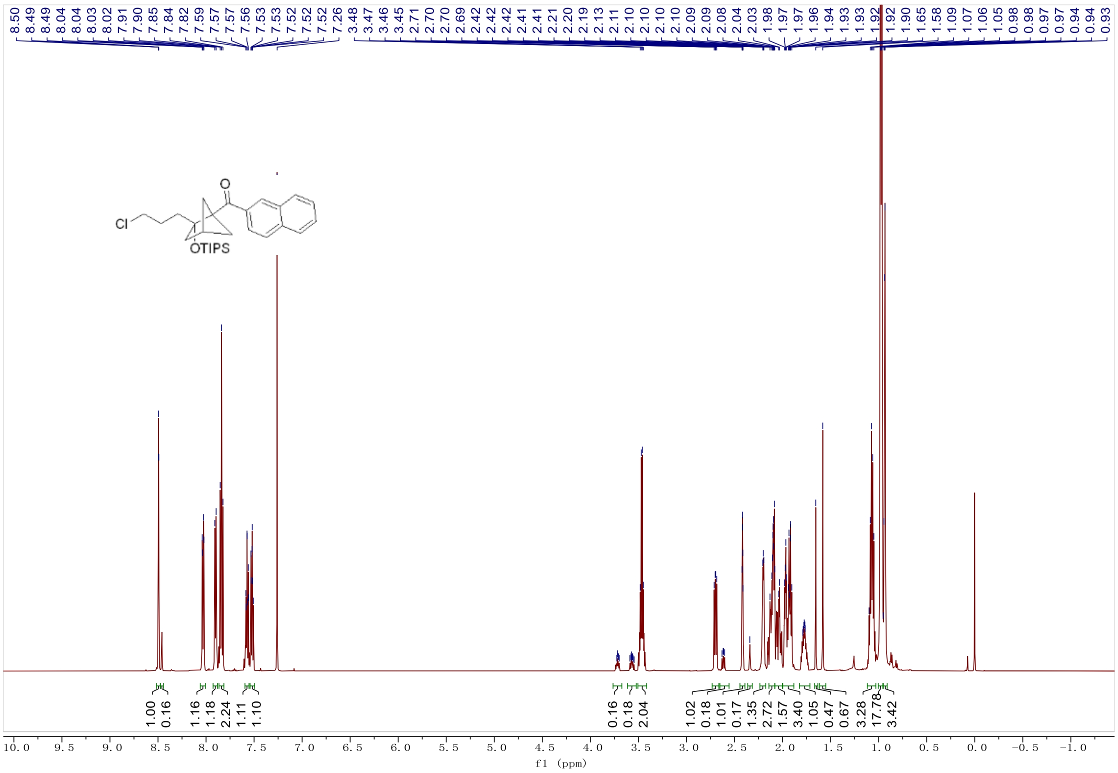

Supplementary Figure 101. **^{1}H NMR** spectrum (600 MHz, $CDCl_3$) of **3y**

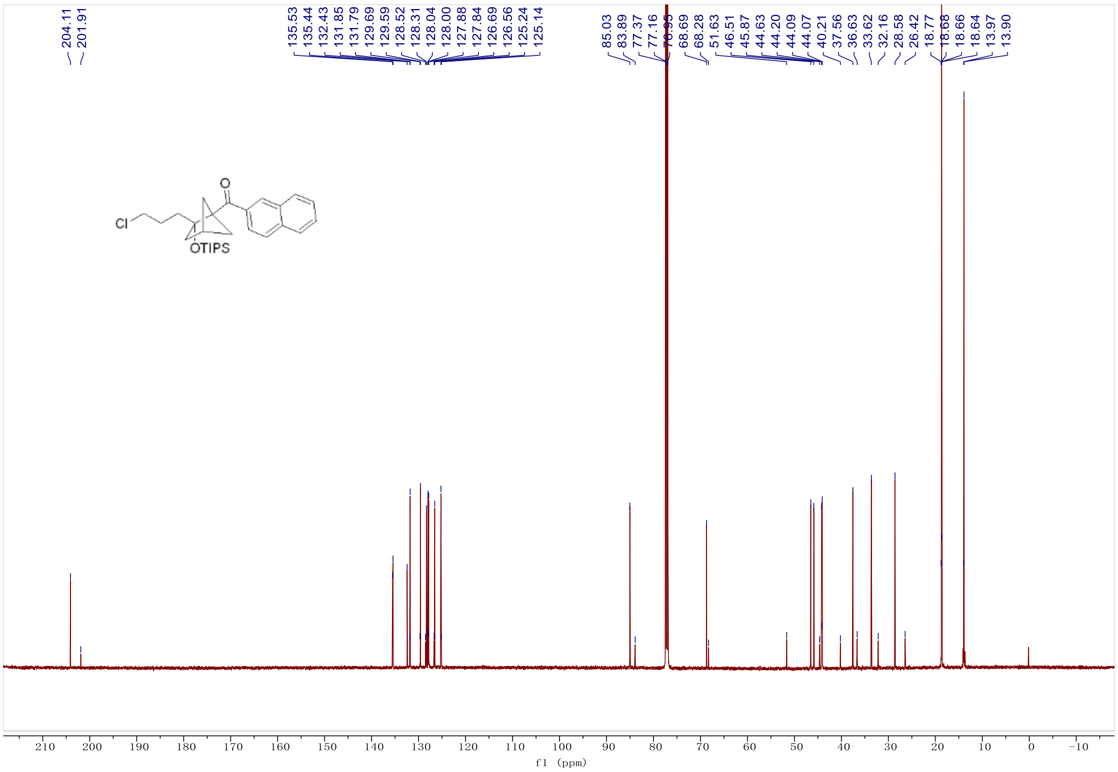

Supplementary Figure 102. **^{13}C NMR** spectrum (125 MHz, $CDCl_3$) of **3y**

Compound 3z

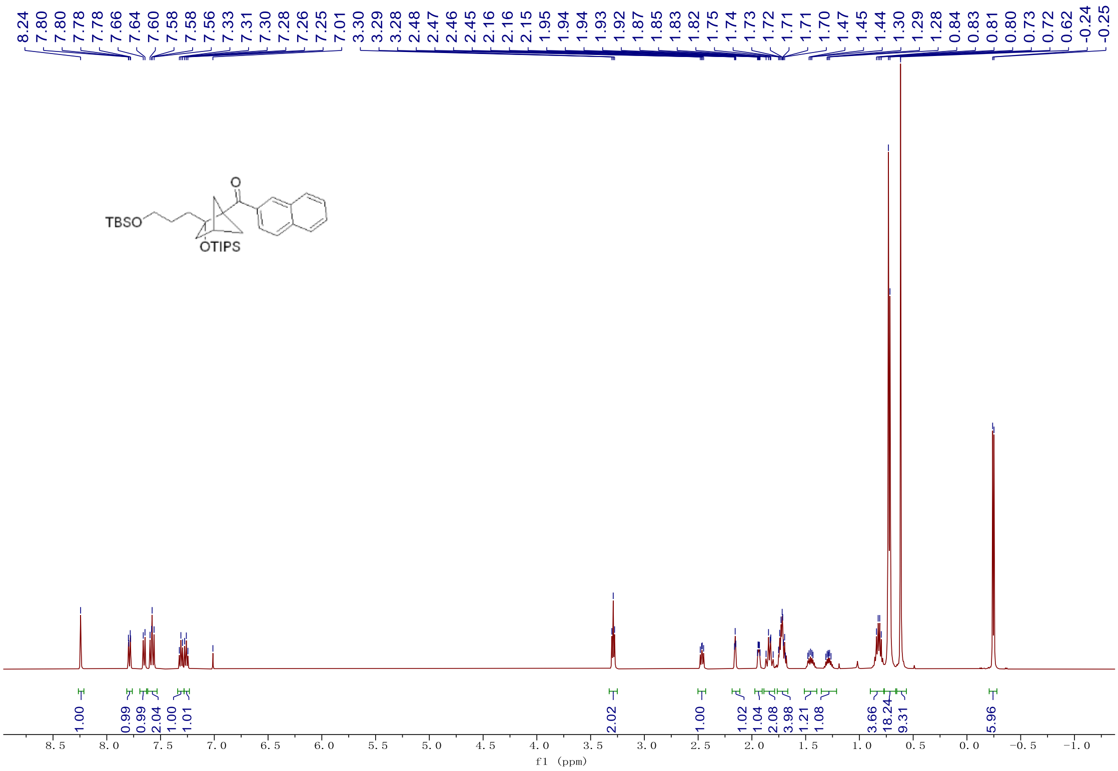

Supplementary Figure 103. **^{1}H NMR** spectrum (500 MHz, $CDCl_3$) of **3z**

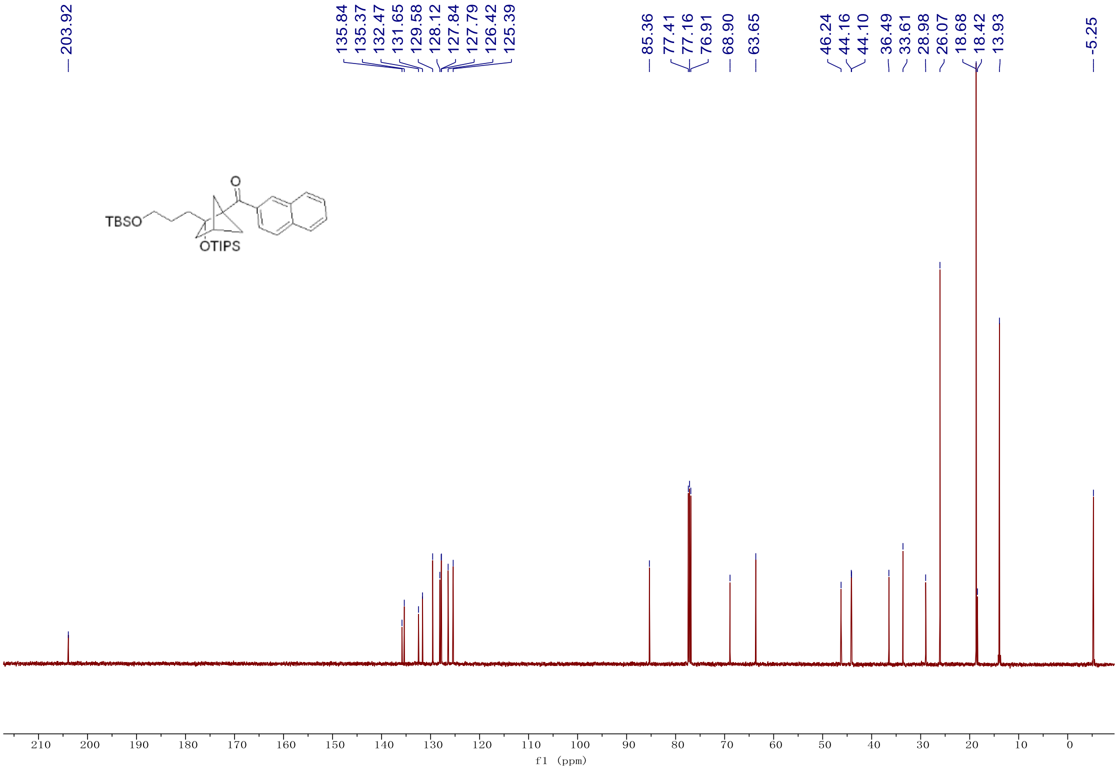

Supplementary Figure 104. **^{13}C NMR** spectrum (125 MHz, $CDCl_3$) of **3z**

Compound 3aa

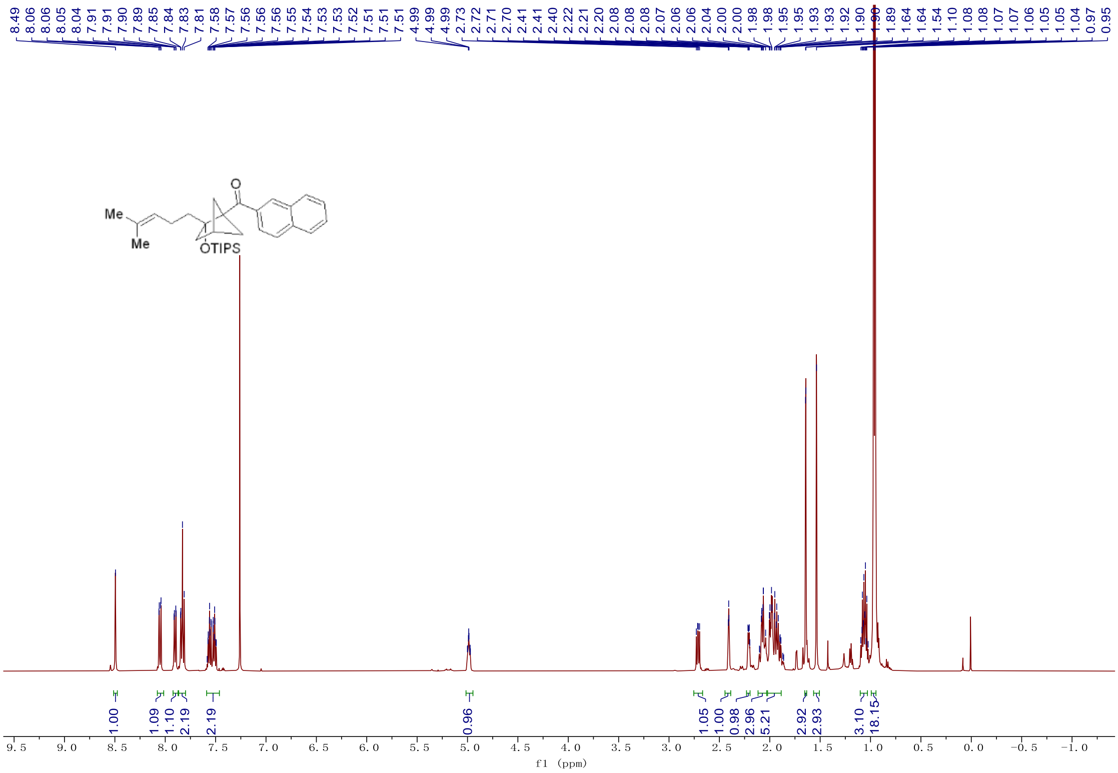

Supplementary Figure 105. **^{1}H NMR** spectrum (500 MHz, $CDCl_3$) of **3aa**

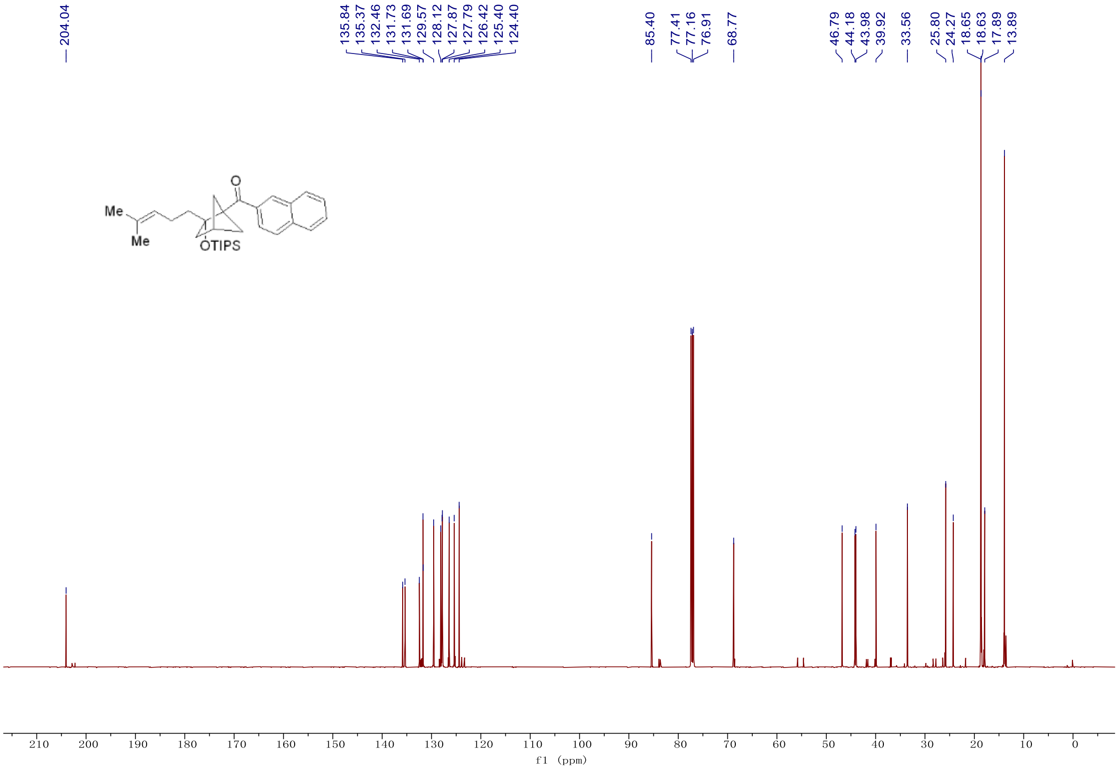

Supplementary Figure 106. **^{13}C NMR** spectrum (125 MHz, $CDCl_3$) of **3aa**

Compound 3ab

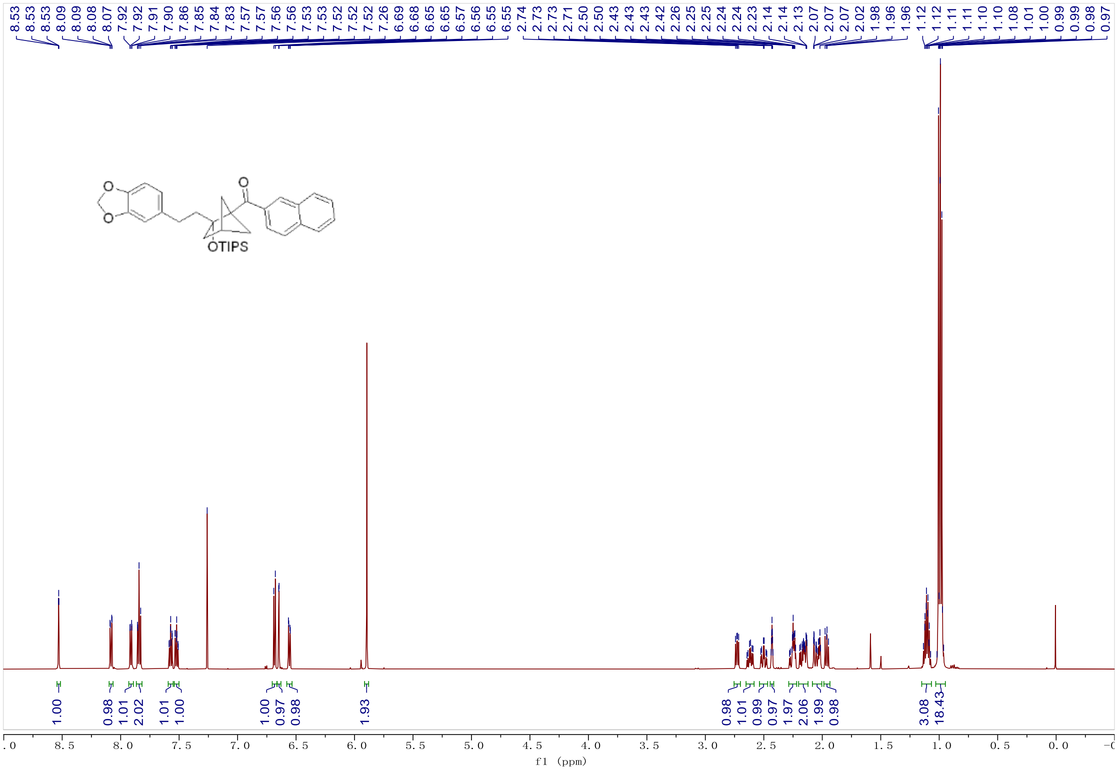

Supplementary Figure 107. **^{1}H NMR** spectrum (600 MHz, $CDCl_3$) of **3ab**

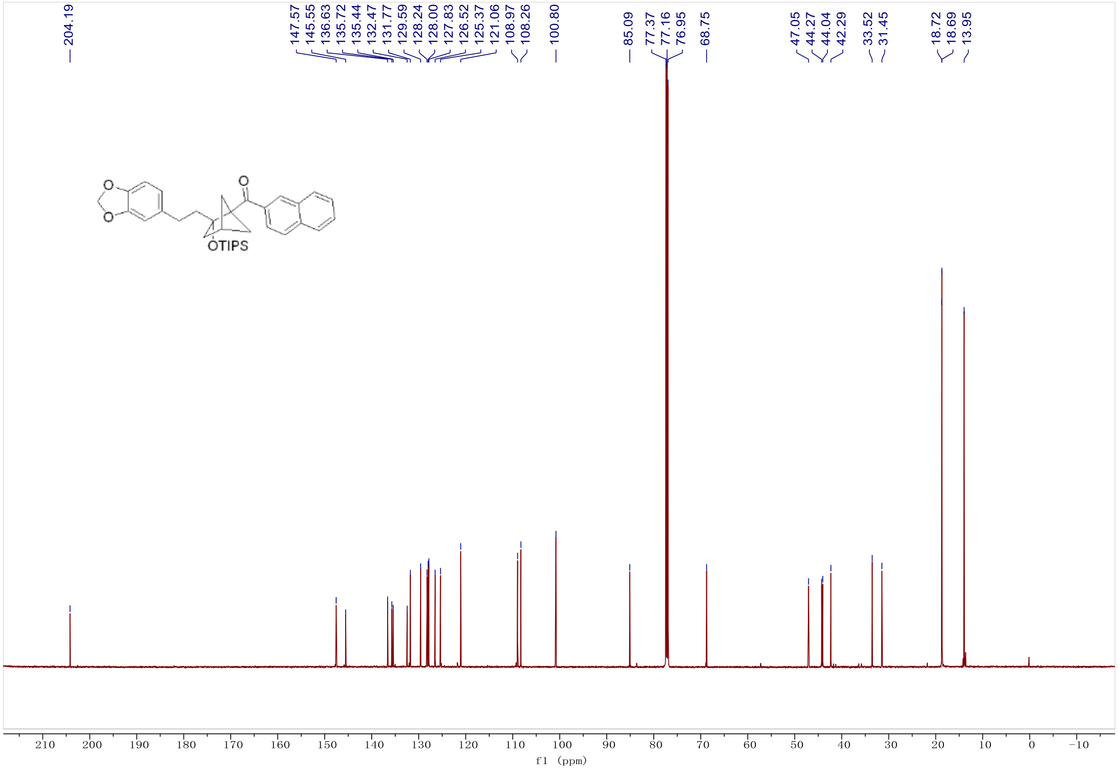

Supplementary Figure 108. **^{13}C NMR** spectrum (150 MHz, $CDCl_3$) of **3ab**

Compound 3ac

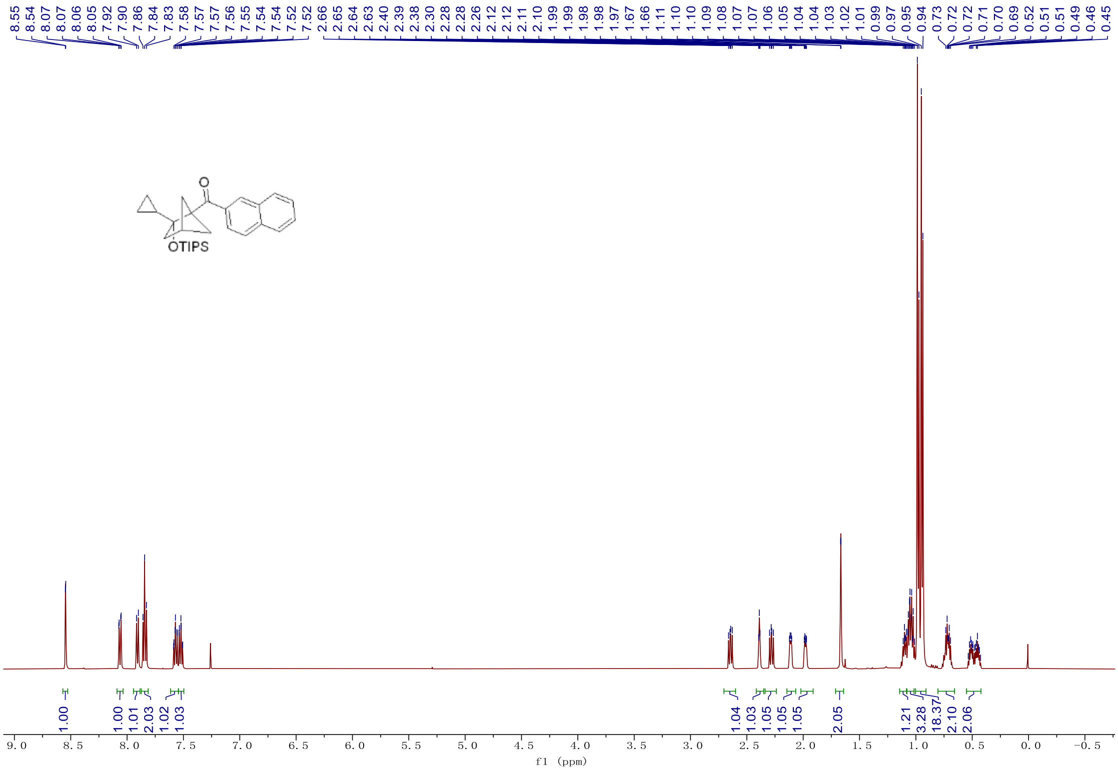

Supplementary Figure 109. **^{1}H NMR** spectrum (500 MHz, $CDCl_3$) of **3ac**

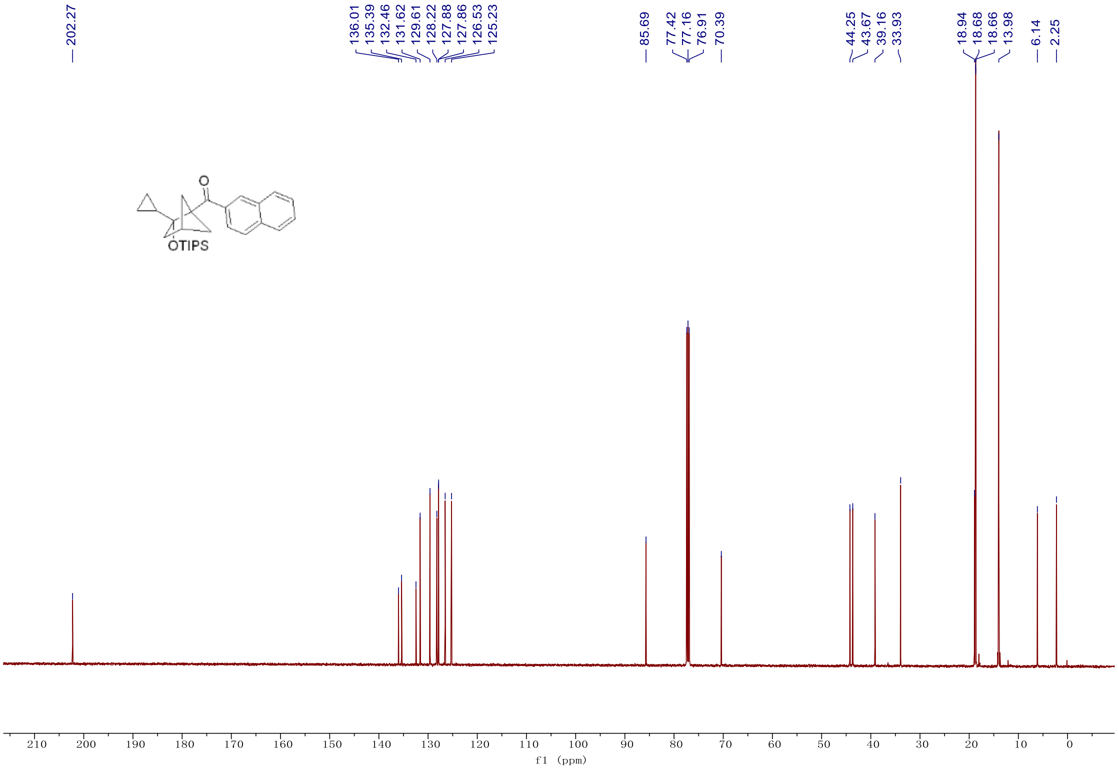

Supplementary Figure 110. **^{13}C NMR** spectrum (125 MHz, $CDCl_3$) of **3ac**

Compound 3ad

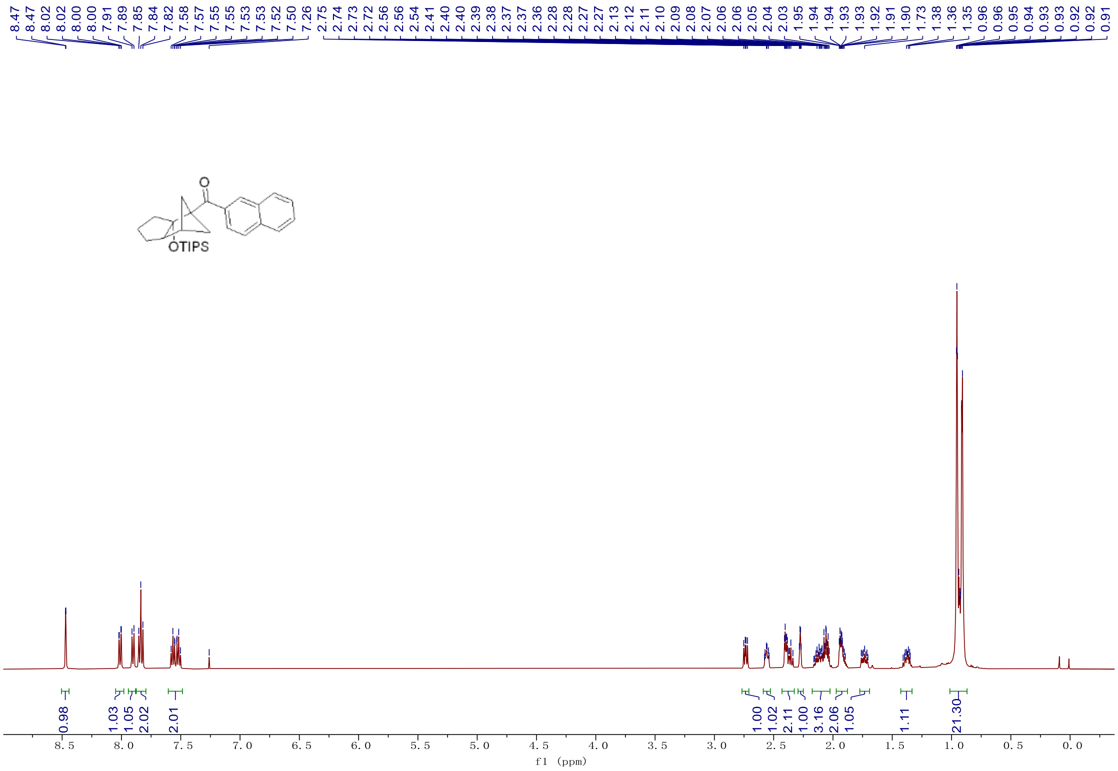

Supplementary Figure 111. ^{1}**H NMR** spectrum (500 MHz, $CDCl_3$) of **3ad**

^{13}**C NMR** (125 MHz, $CDCl_3$)

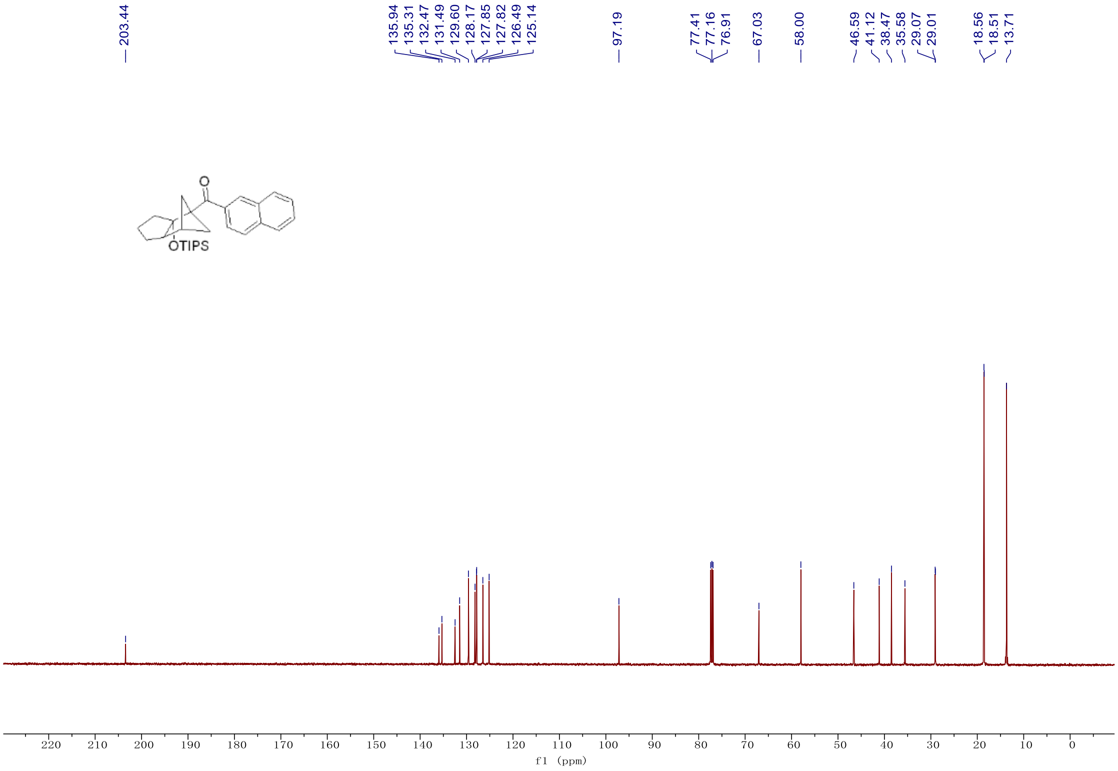

Supplementary Figure 112. ^{13}**C NMR** spectrum (125 MHz, $CDCl_3$) of **3ad**

Compound 3ae

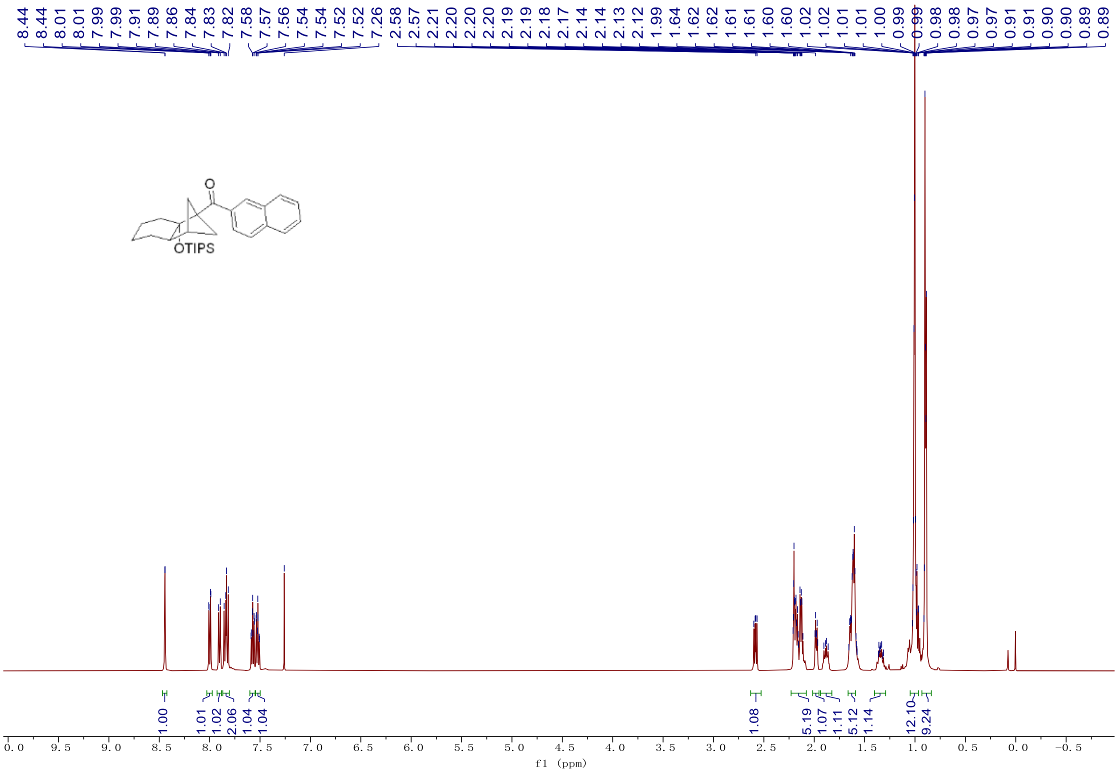

Supplementary Figure 113. ^{1}**H NMR** spectrum (500 MHz, $CDCl_3$) of **3ae**

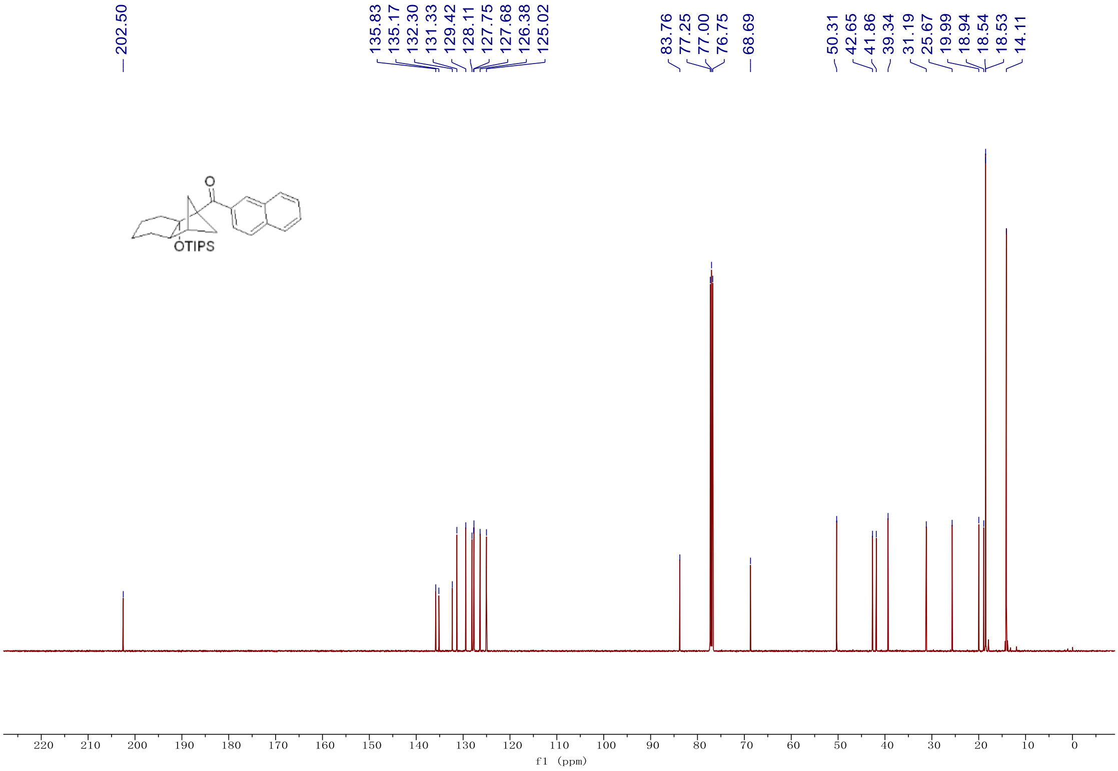

Supplementary Figure 114. ^{13}**C NMR** spectrum (125 MHz, $CDCl_3$) of **3ae**

Compound 3af

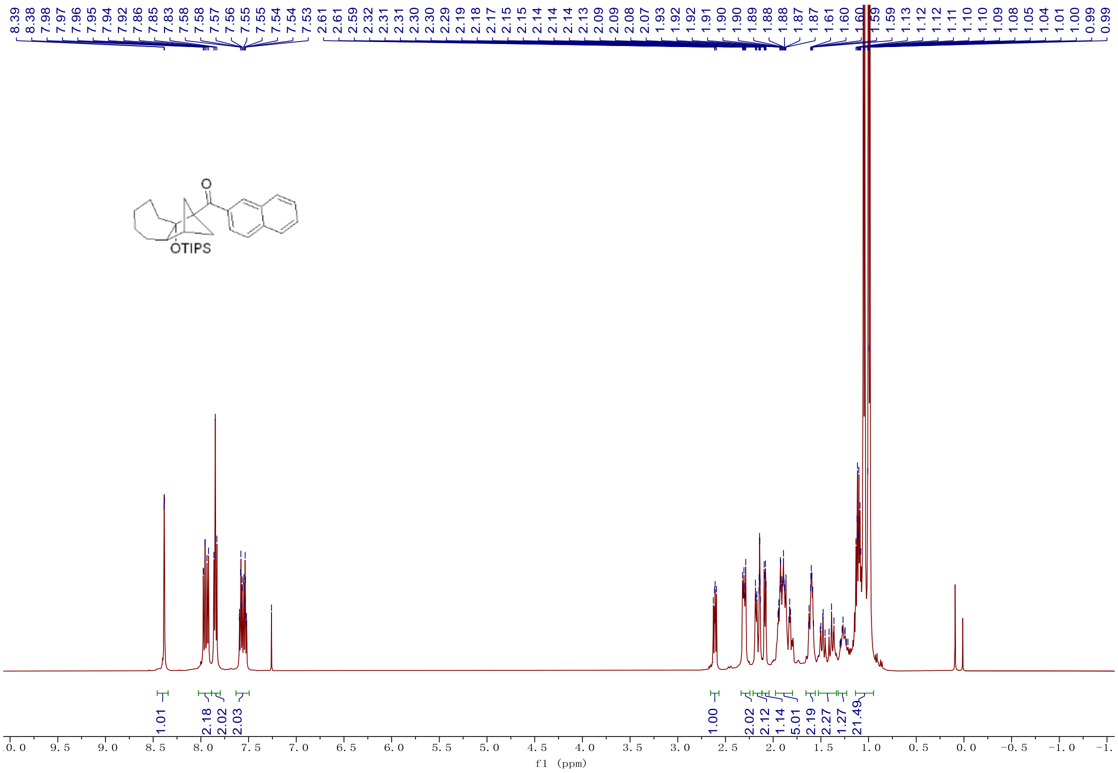

Supplementary Figure 115. ^{1}H NMR spectrum (500 MHz, $CDCl_3$) of **3af**

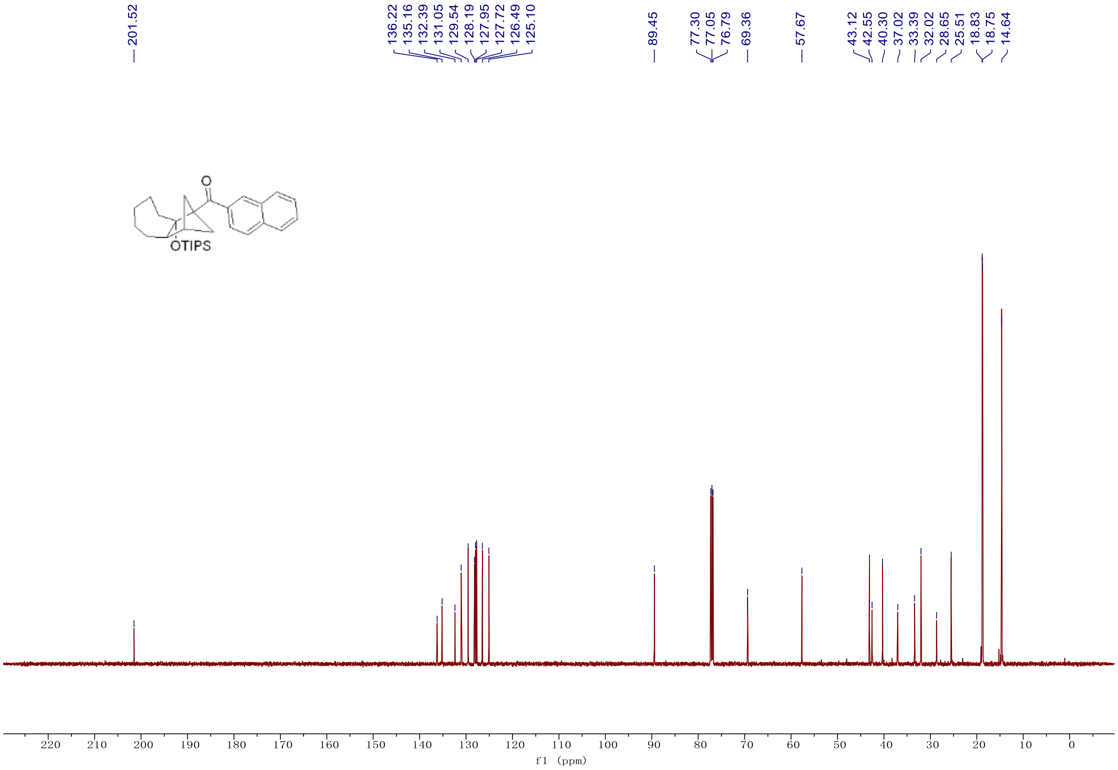

Supplementary Figure 116. ^{13}C NMR spectrum (125 MHz, $CDCl_3$) of **3af**

Compound 3ag

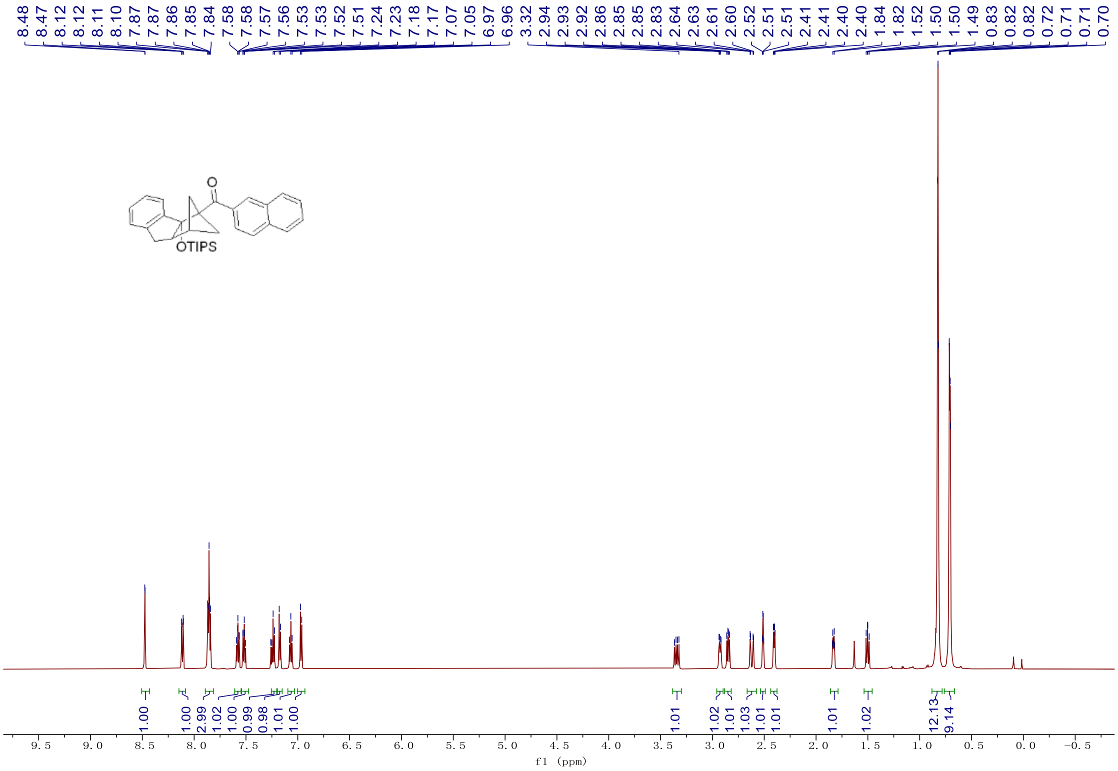

Supplementary Figure 117. ^{1}**H NMR** spectrum (600 MHz, $CDCl_3$) of **3ag**

^{13}**C NMR** (150 MHz, $CDCl_3$)

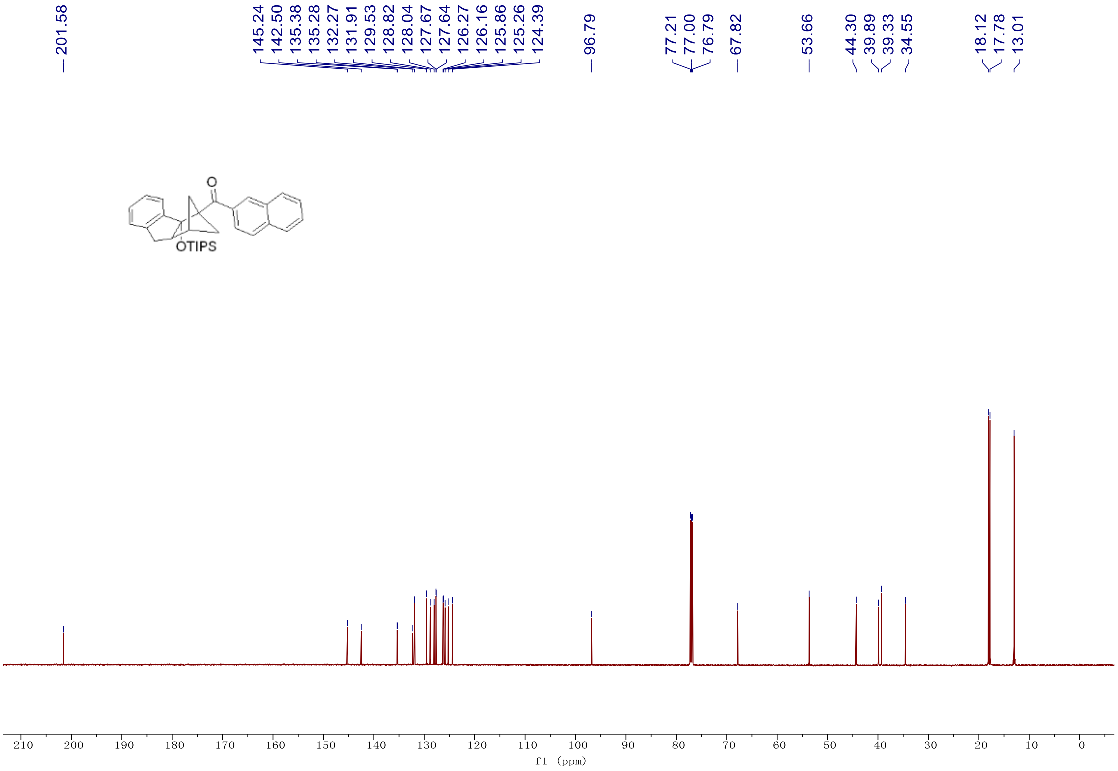

Supplementary Figure 118. ^{13}**C NMR** spectrum (150 MHz, $CDCl_3$) of **3ag**

Compound 3ah

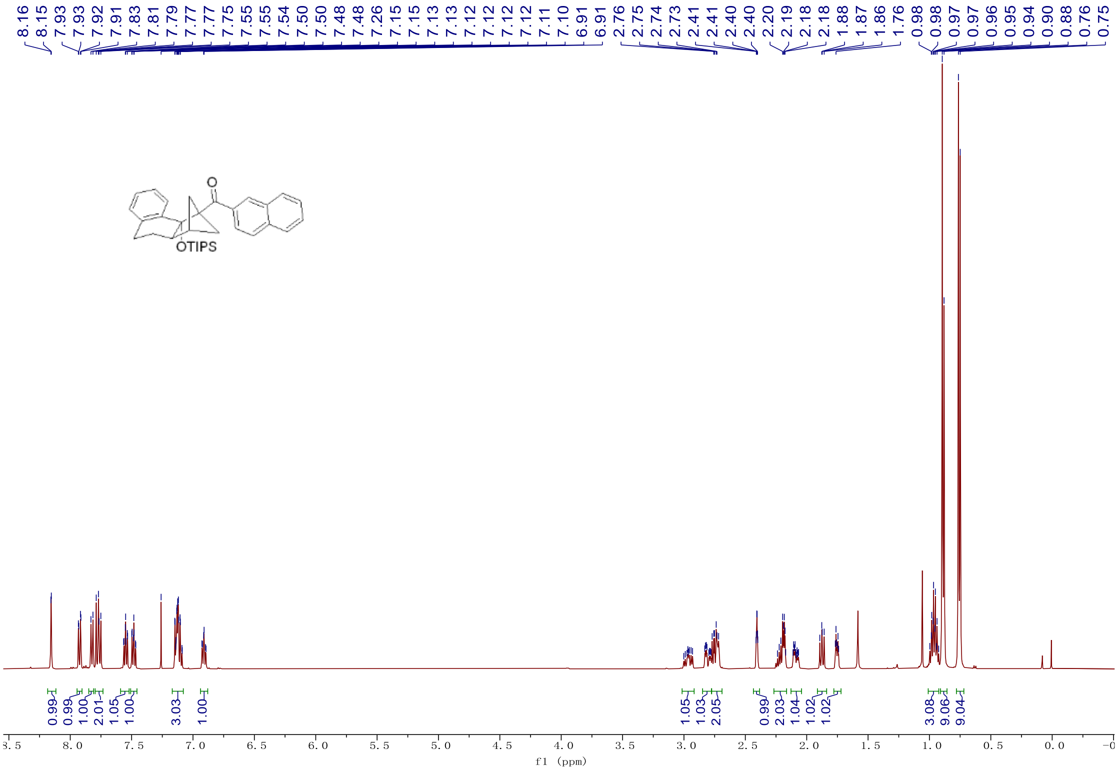

Supplementary Figure 119. ^{1}**H NMR** spectrum (500 MHz, $CDCl_3$) of **3ah**

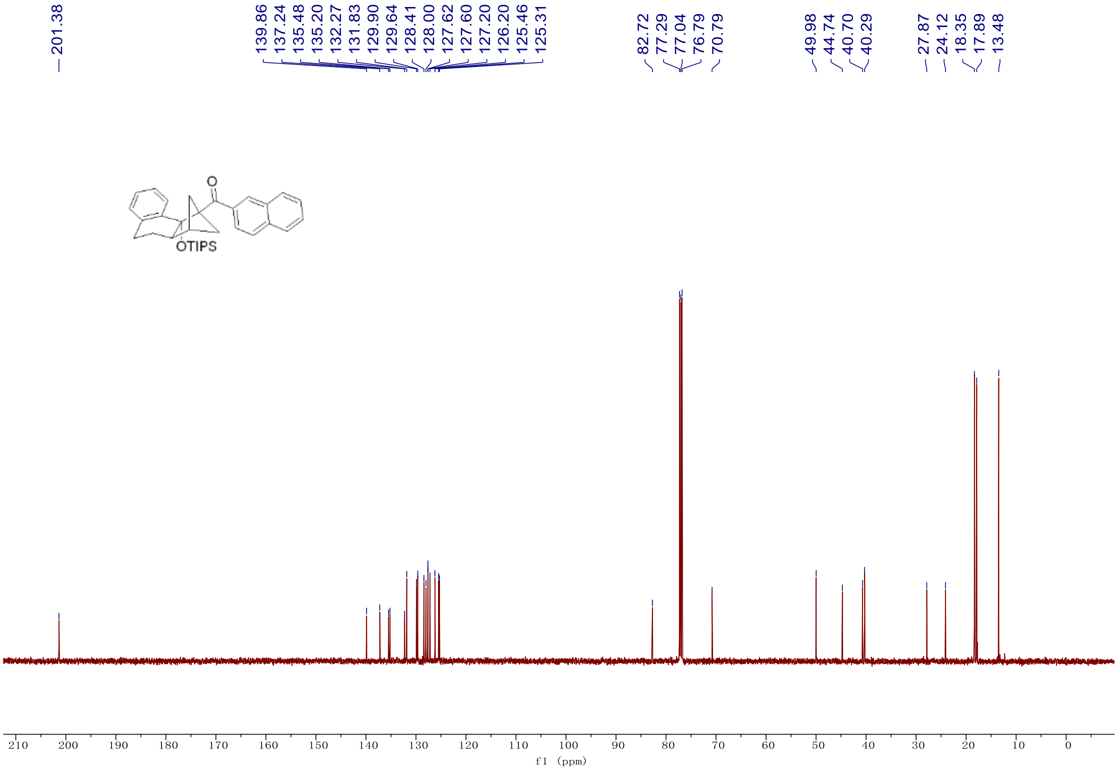

Supplementary Figure 120. ^{13}**C NMR** spectrum (125 MHz, $CDCl_3$) of **3ah**

Compound 3ai

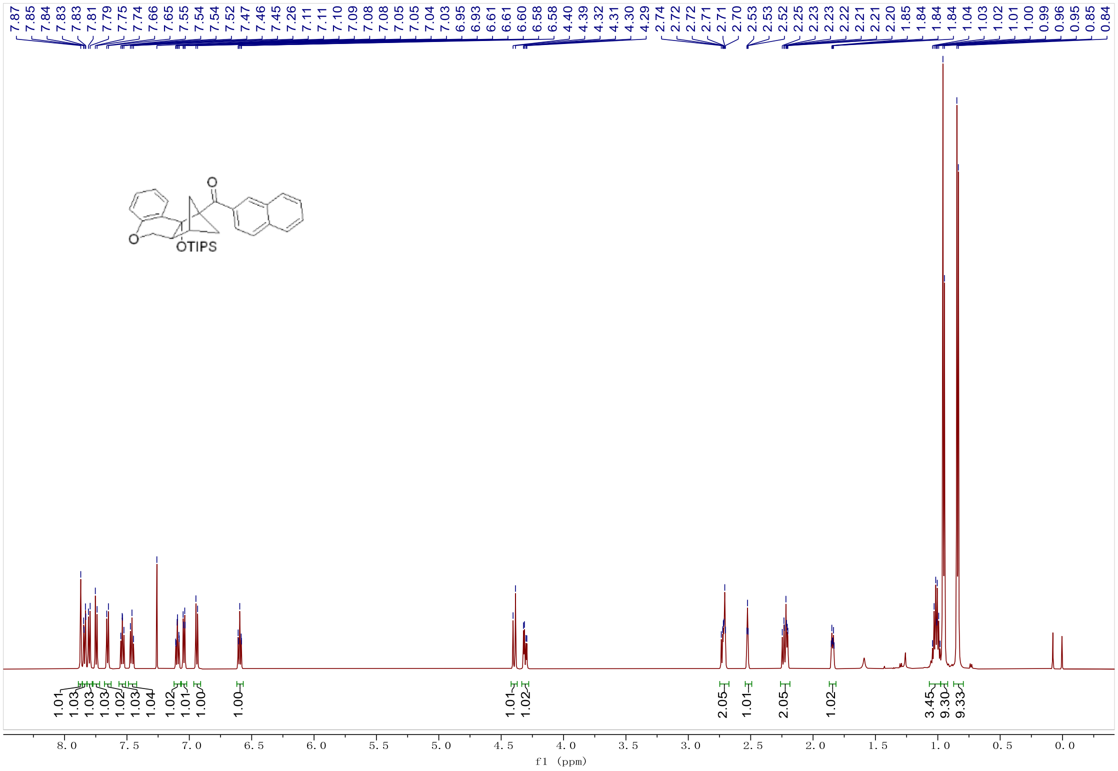

Supplementary Figure 121. ^{1}**H NMR** spectrum (600 MHz, $CDCl_3$) of **3ai**

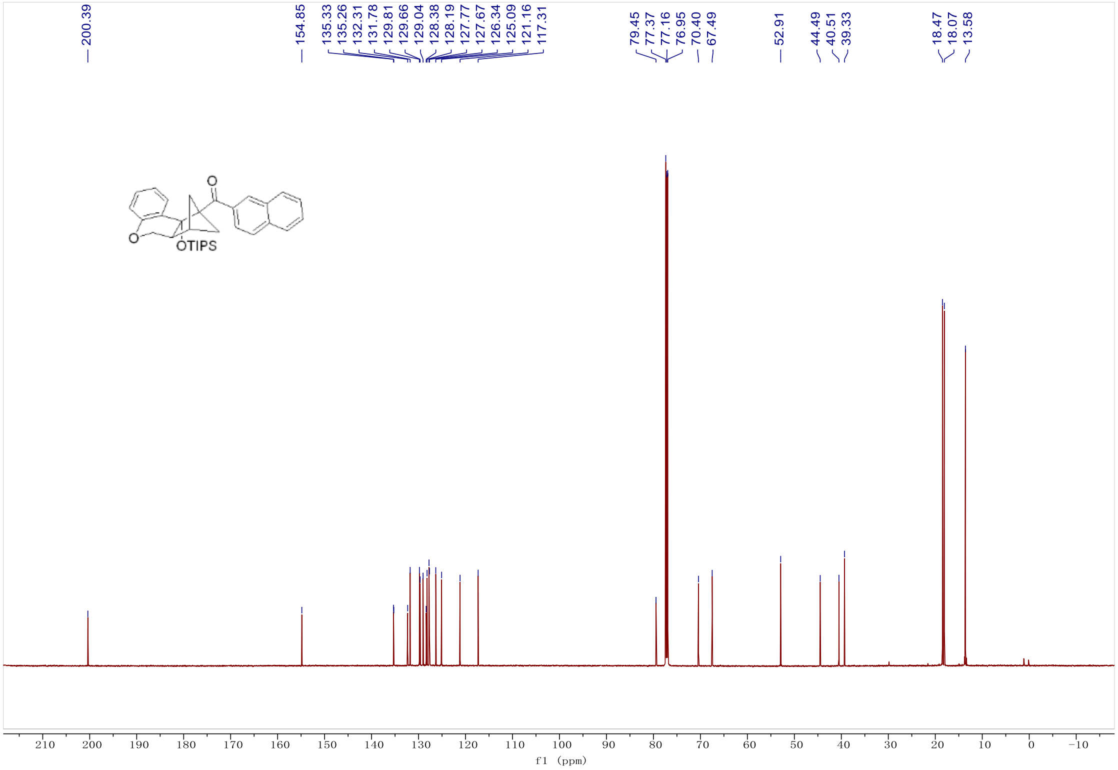

Supplementary Figure 122. ^{13}**C NMR** spectrum (150 MHz, $CDCl_3$) of **3ai**

Compound 3aj

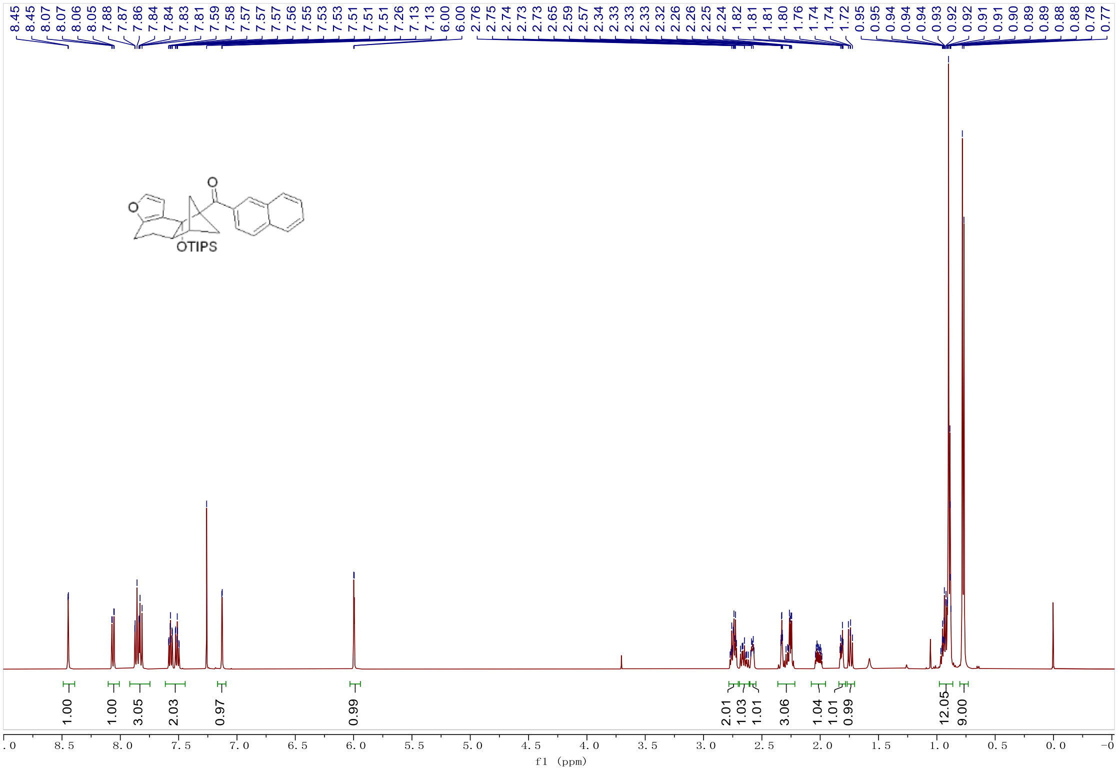

Supplementary Figure 123. ^{1}**H NMR** spectrum (500 MHz, $CDCl_3$) of **3aj**

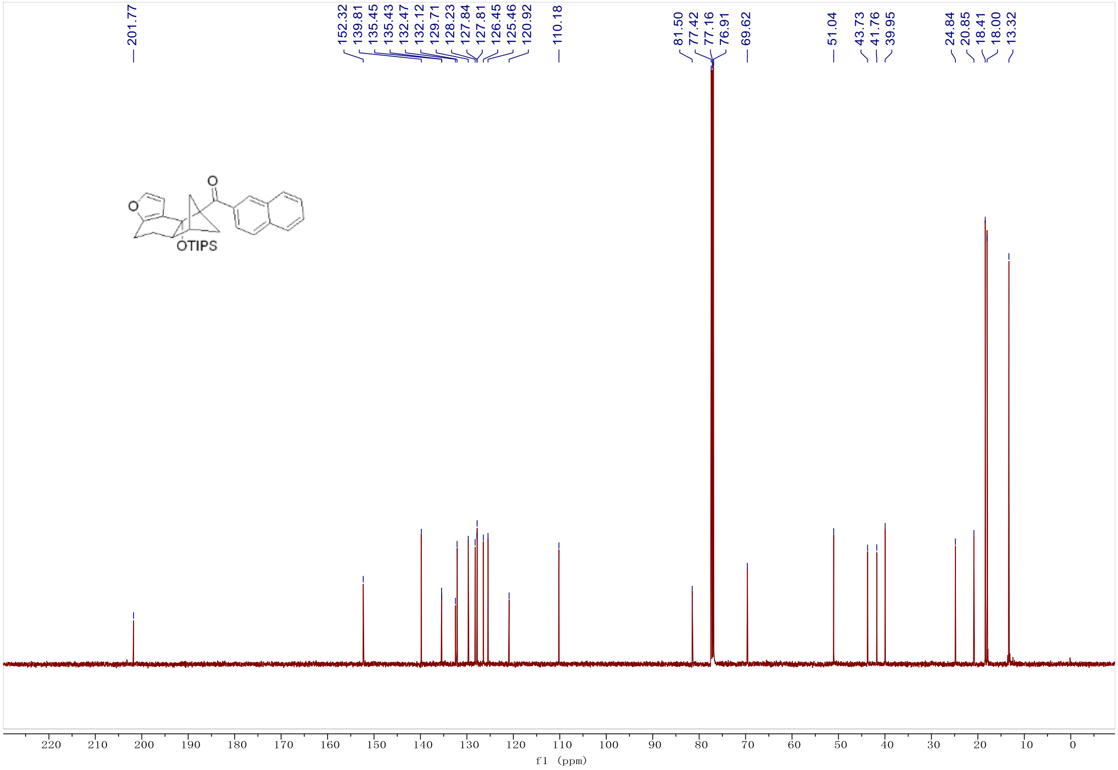

Supplementary Figure 124. ^{13}**C NMR** spectrum (125 MHz, $CDCl_3$) of **3aj**

Compound 3ak

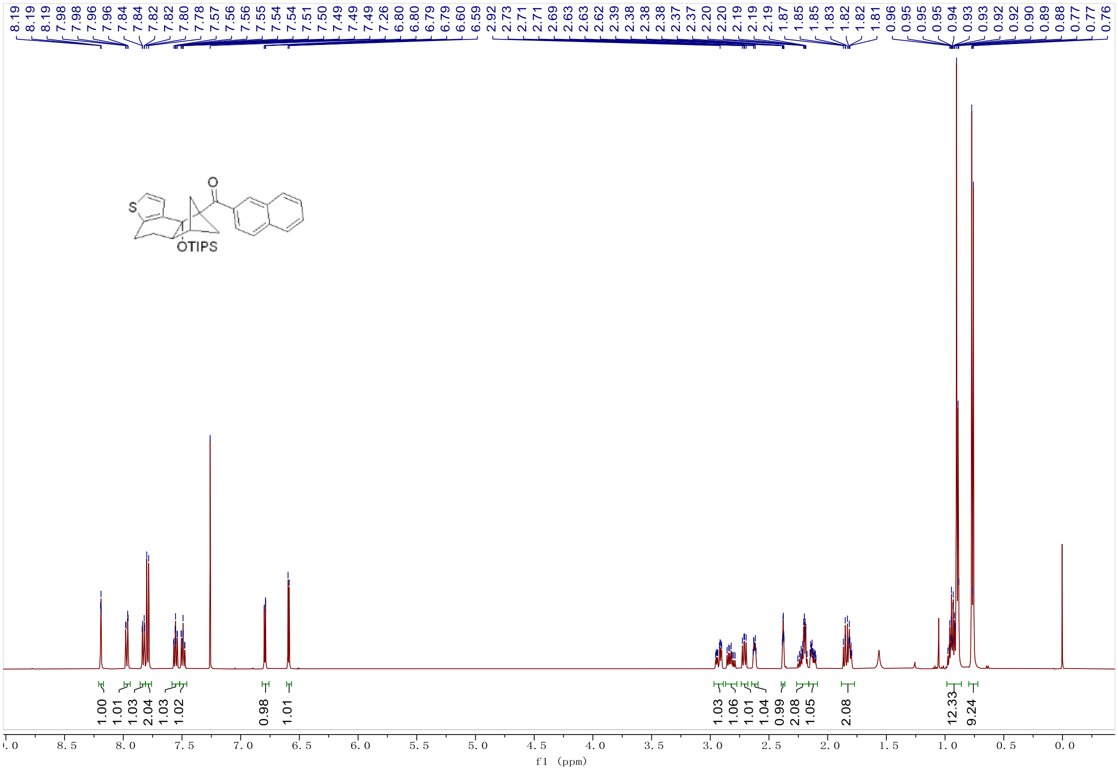

Supplementary Figure 125. ^{1}H NMR spectrum (500 MHz, $CDCl_3$) of **3ak**

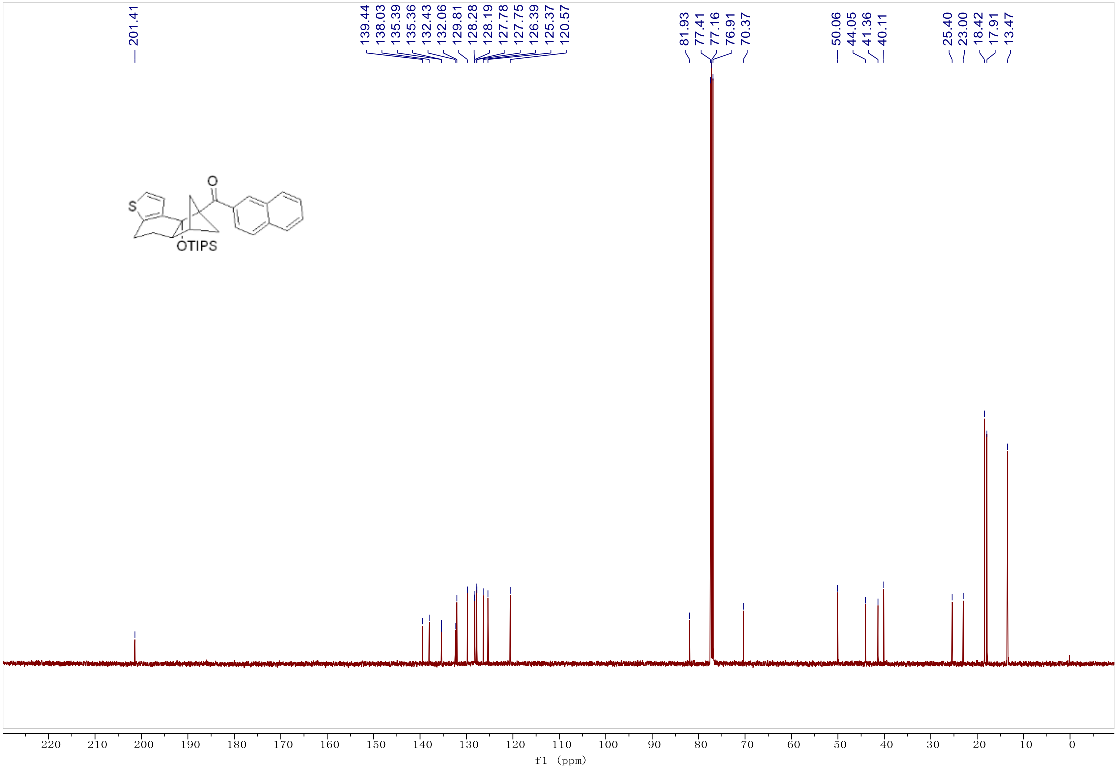

Supplementary Figure 126. ^{13}C NMR spectrum (125 MHz, $CDCl_3$) of **3ak**

Compound 3al

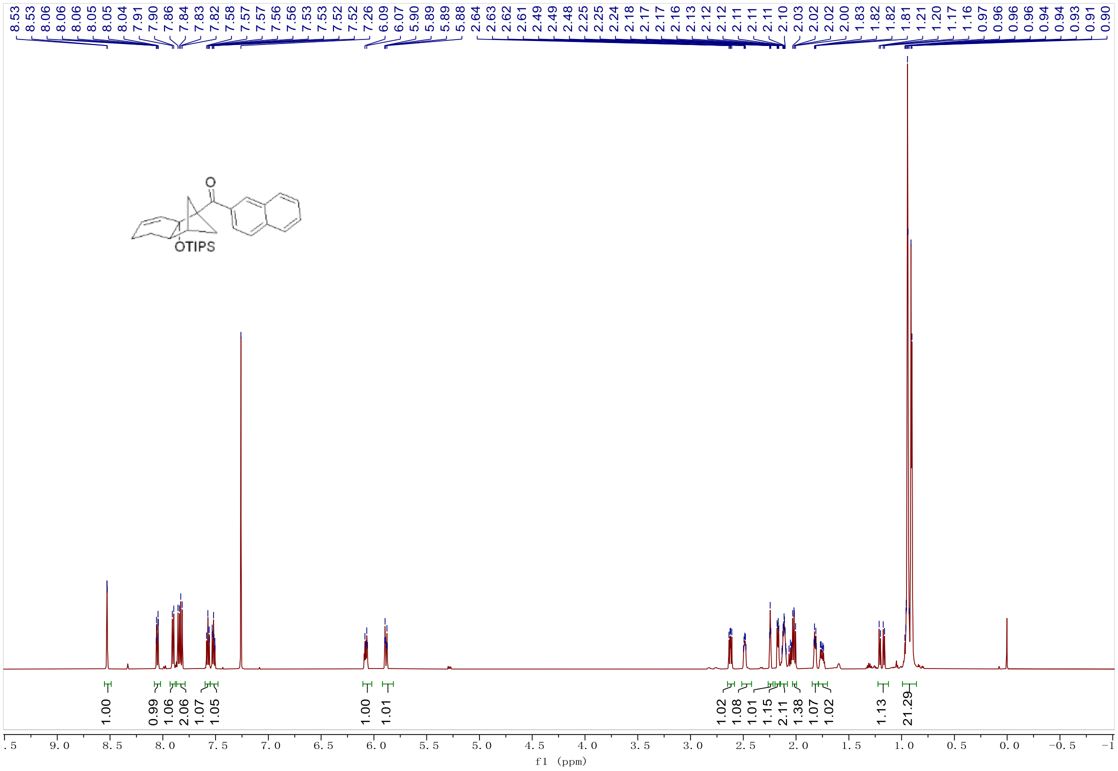

Supplementary Figure 127. ^{1}**H NMR** spectrum (600 MHz, $CDCl_3$) of **3al**

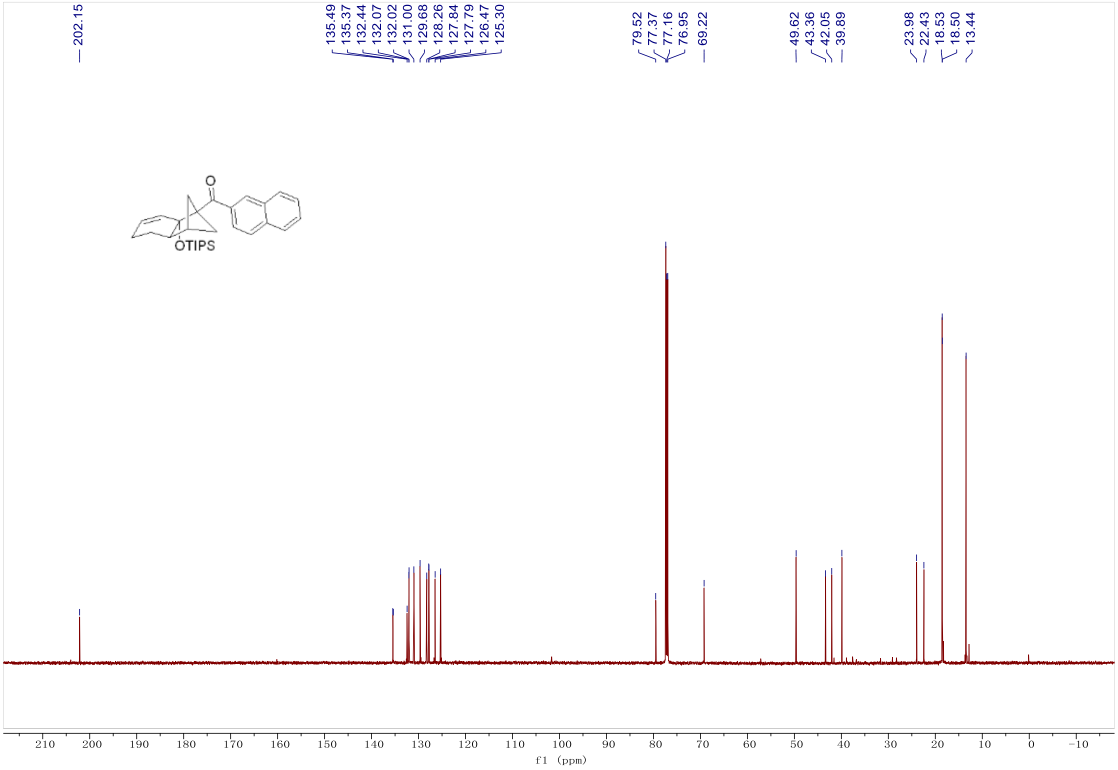

Supplementary Figure 128. ^{13}**C NMR** spectrum (150 MHz, $CDCl_3$) of **3al**

Compound 3am

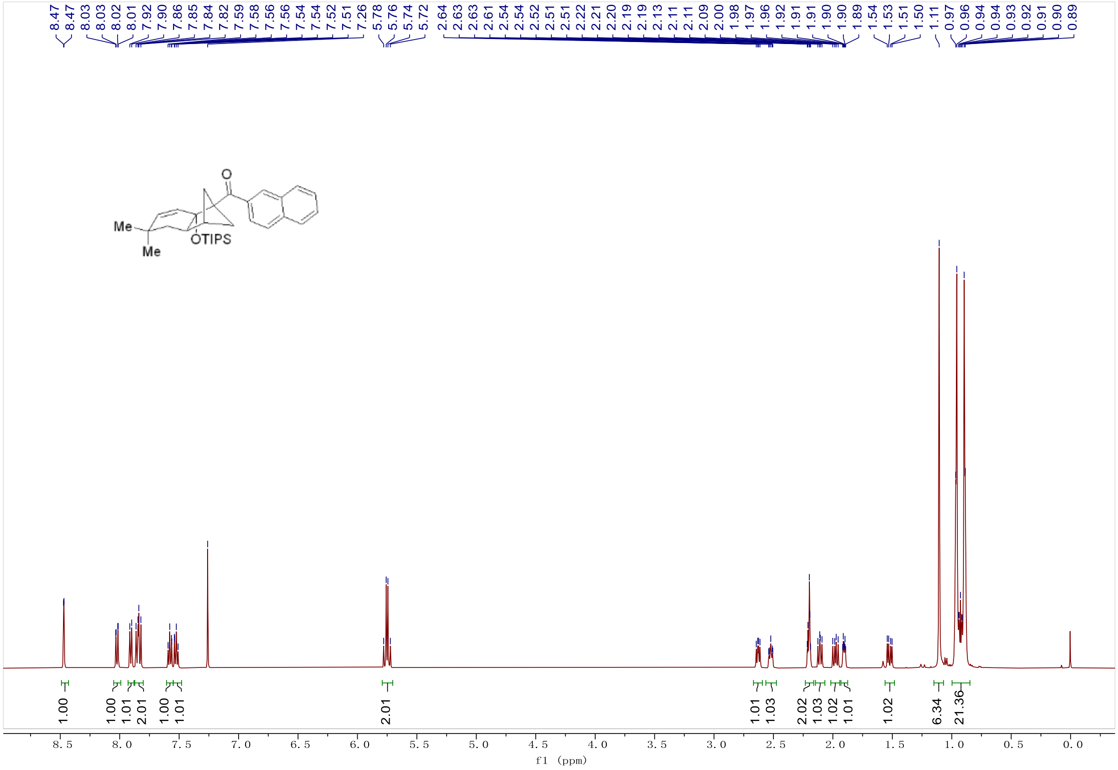

Supplementary Figure 129. ^{1}H NMR spectrum (500 MHz, $CDCl_3$) of **3am**

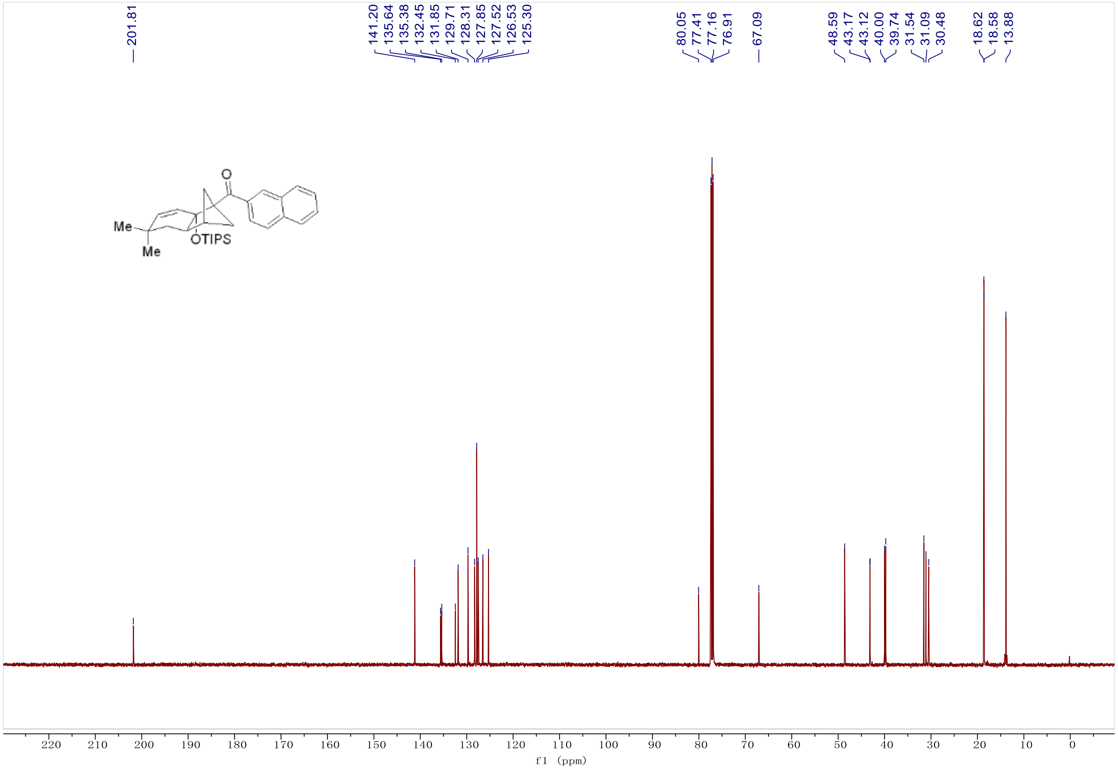

Supplementary Figure 130. ^{13}C NMR spectrum (125 MHz, $CDCl_3$) of **3am**

Compound 3an

^{1}H NMR (500 MHz, $CDCl_3$)

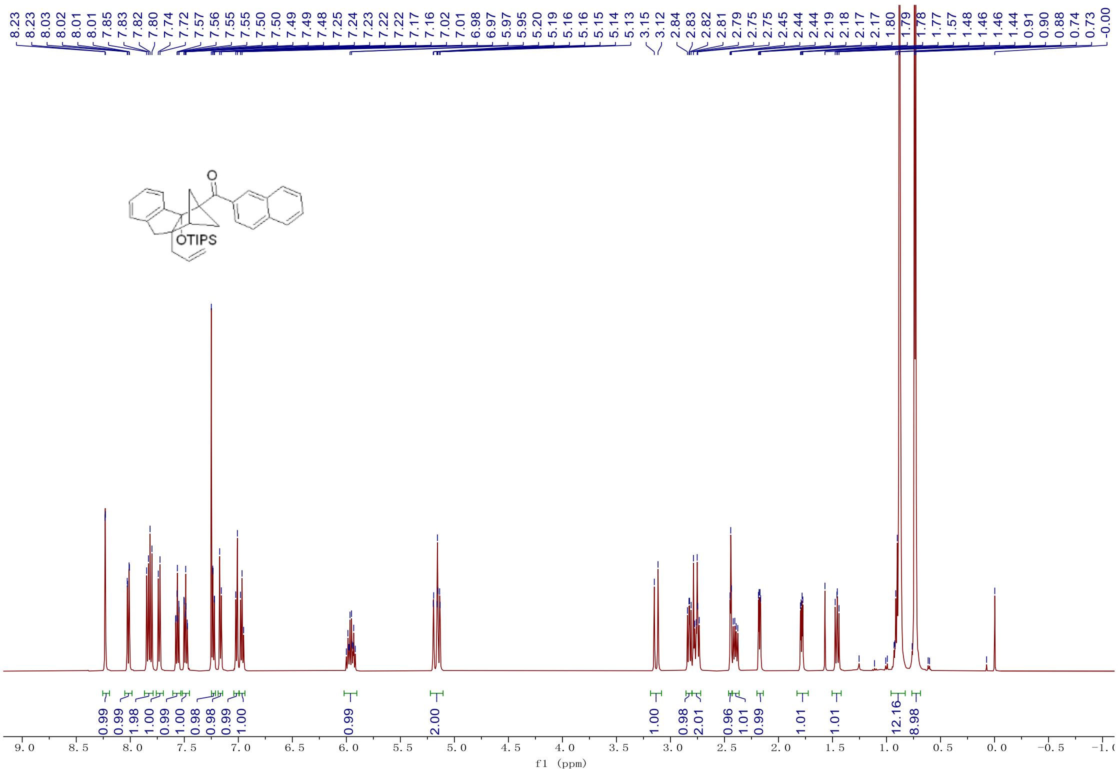

Supplementary Figure 131. ^{1}H NMR spectrum (500 MHz, $CDCl_3$) of **3an**

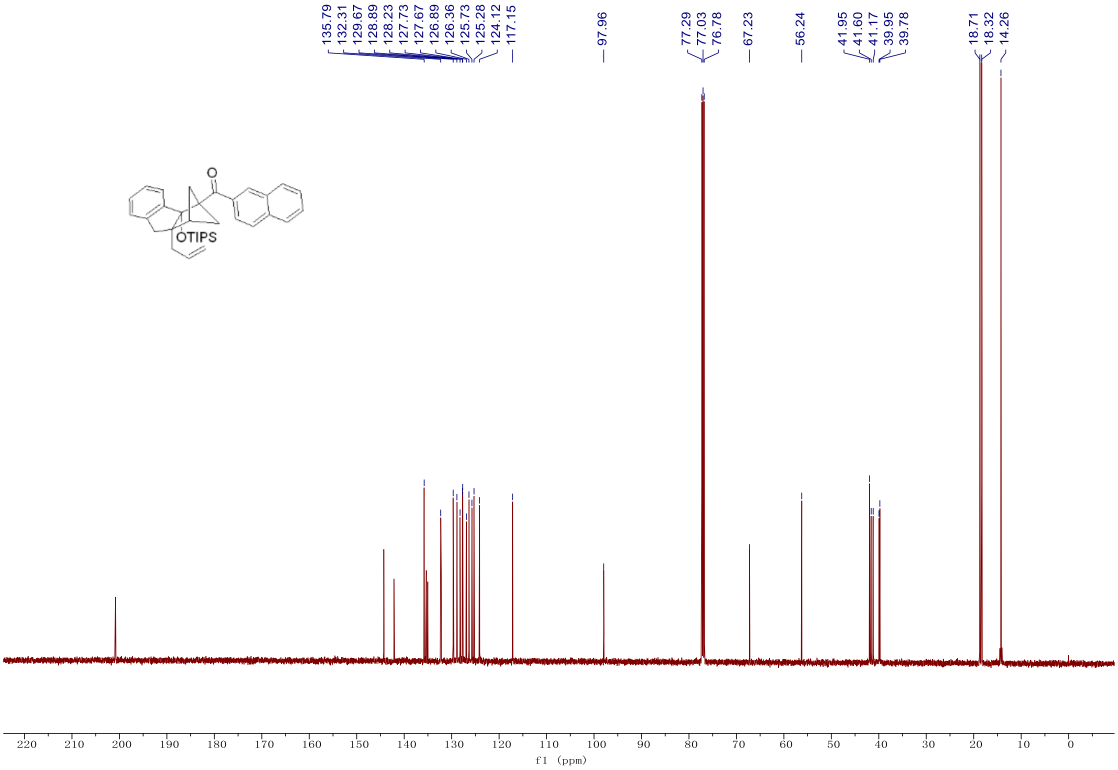

Supplementary Figure 132. ^{13}C NMR spectrum (125 MHz, $CDCl_3$) of **3an**

COSY NMR (500 MHz, $CDCl_3$)

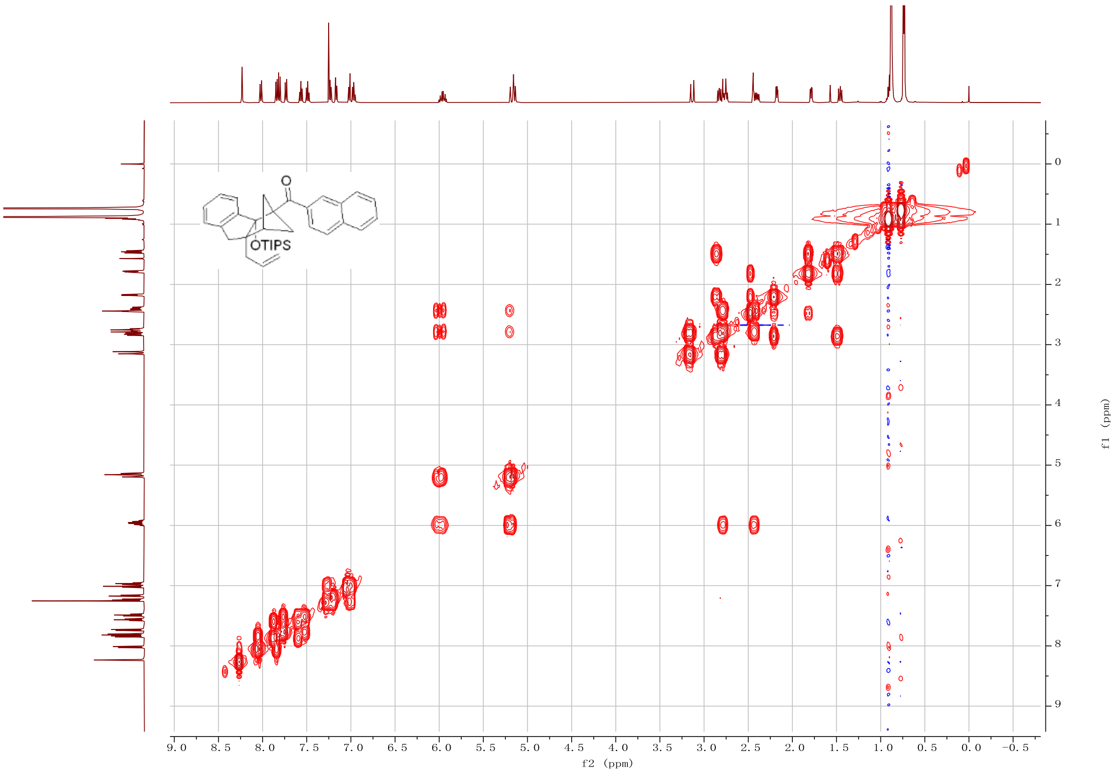

Supplementary Figure 133. COSY NMR spectrum (500 MHz, $CDCl_3$) of **3an**

HSQC NMR (500 MHz, $CDCl_3$)

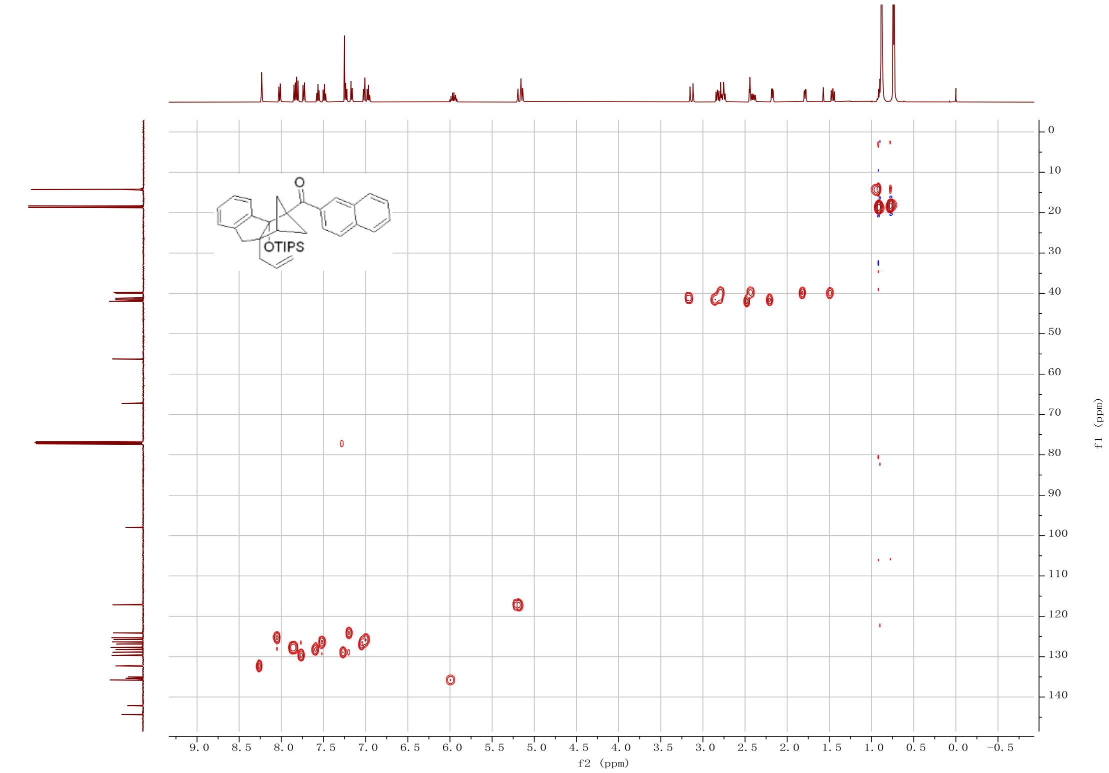

Supplementary Figure 134. HSQC NMR spectrum (500 MHz, $CDCl_3$) of **3an**

NOESY NMR (500 MHz, $CDCl_3$)

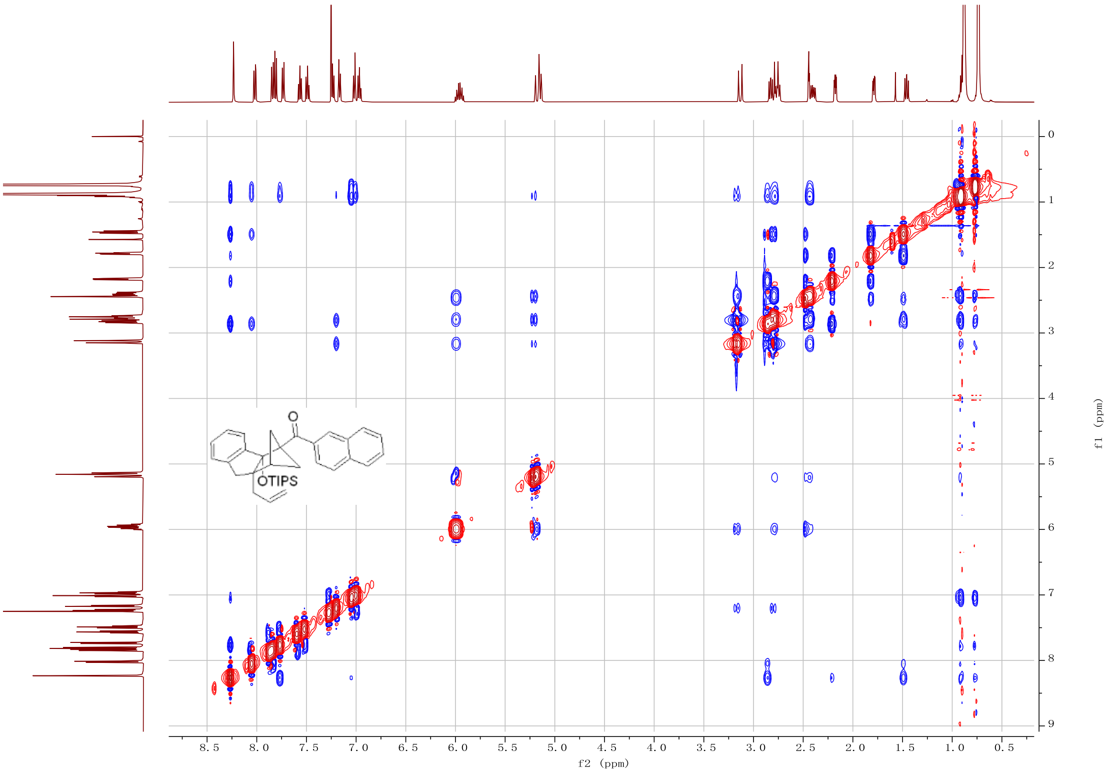

Supplementary Figure 135. NOESY NMR spectrum (500 MHz, $CDCl_3$) of **3an**

Compound 3ao

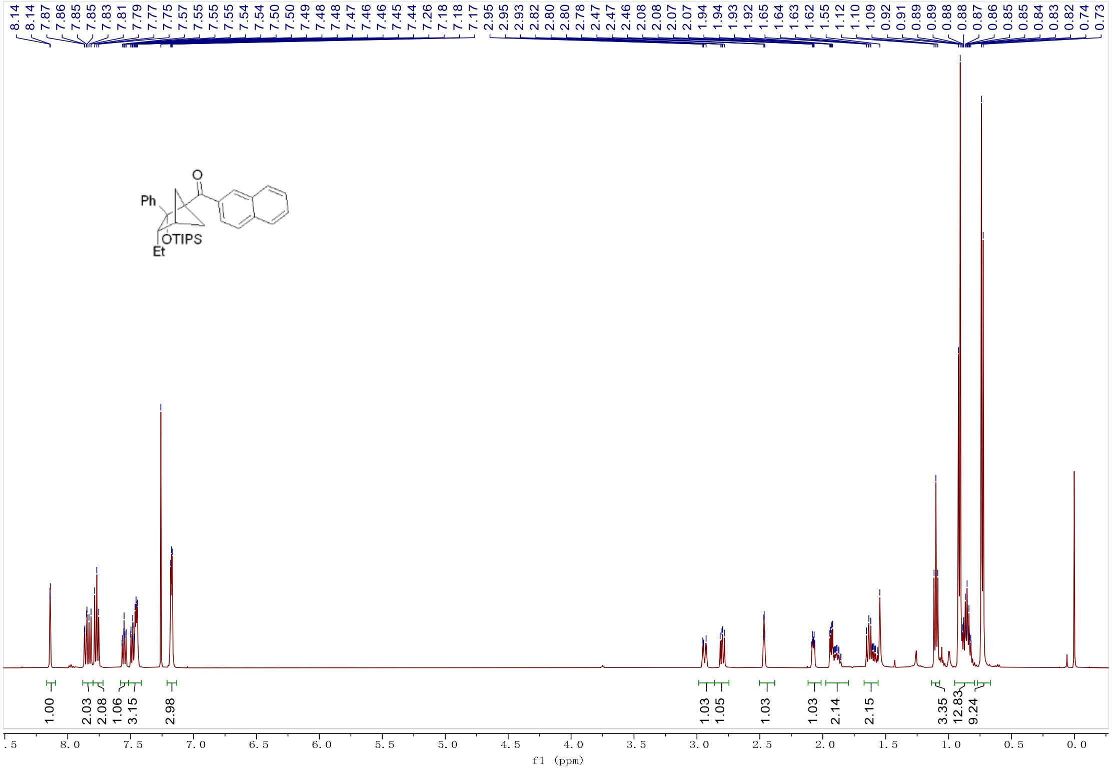

Supplementary Figure 136. ^{1}H NMR spectrum (500 MHz, CDCl$_3$) of **3ao**

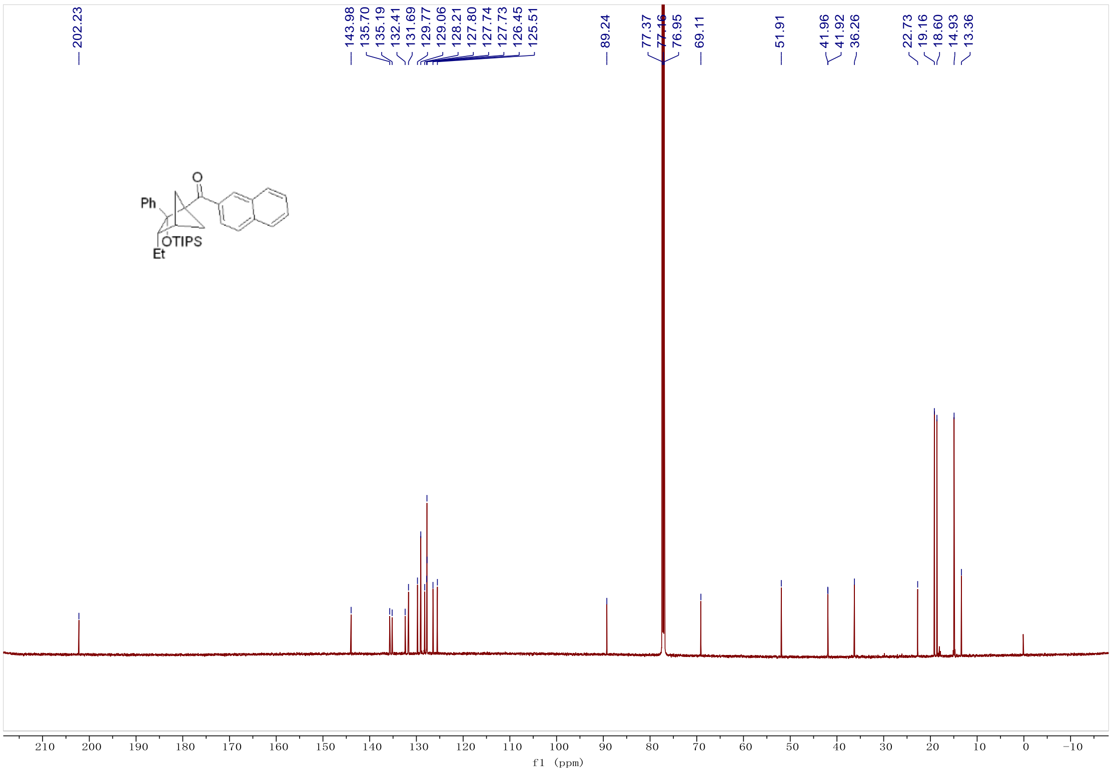

Supplementary Figure 137. ^{13}C NMR spectrum (150 MHz, CDCl$_3$) of **3ao**

COSY NMR (600 MHz, $CDCl_3$)

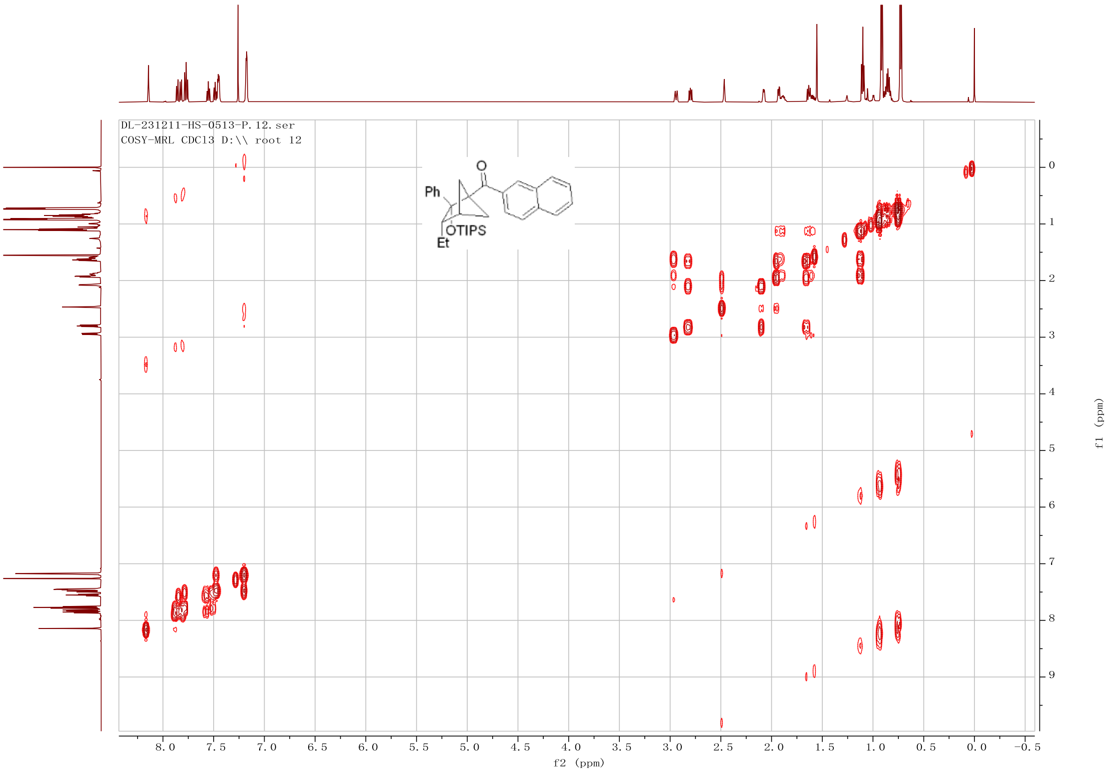

Supplementary Figure 138. COSY NMR spectrum (500 MHz, $CDCl_3$) of **3ao**

NOESY NMR (600 MHz, $CDCl_3$)

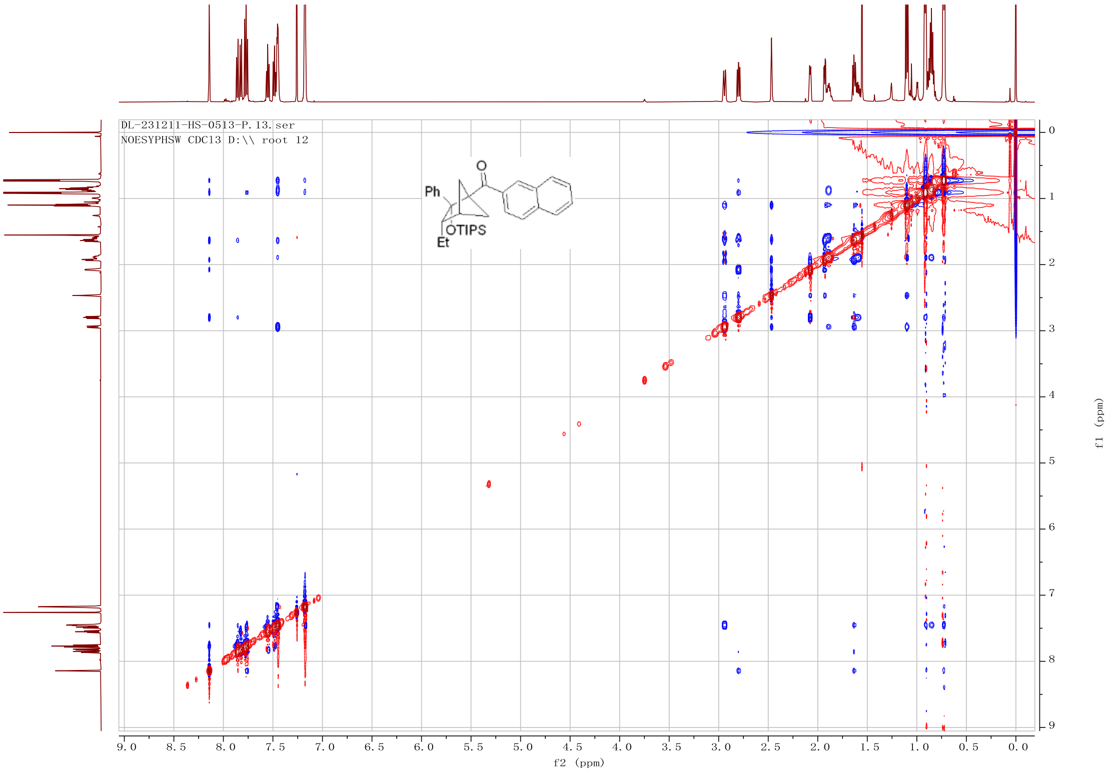

Supplementary Figure 139. NOESY NMR spectrum (500 MHz, $CDCl_3$) of **3ao**

Compound 3ap

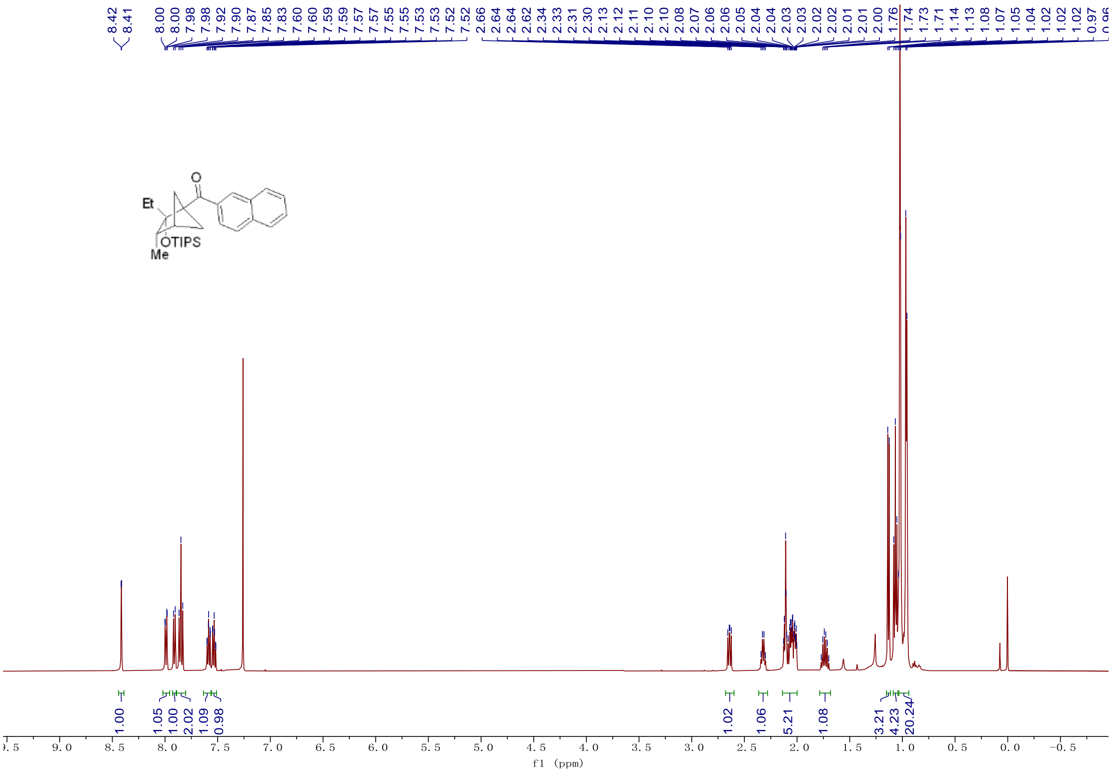

Supplementary Figure 140. ^{1}H NMR spectrum (500 MHz, $CDCl_3$) of 3ap

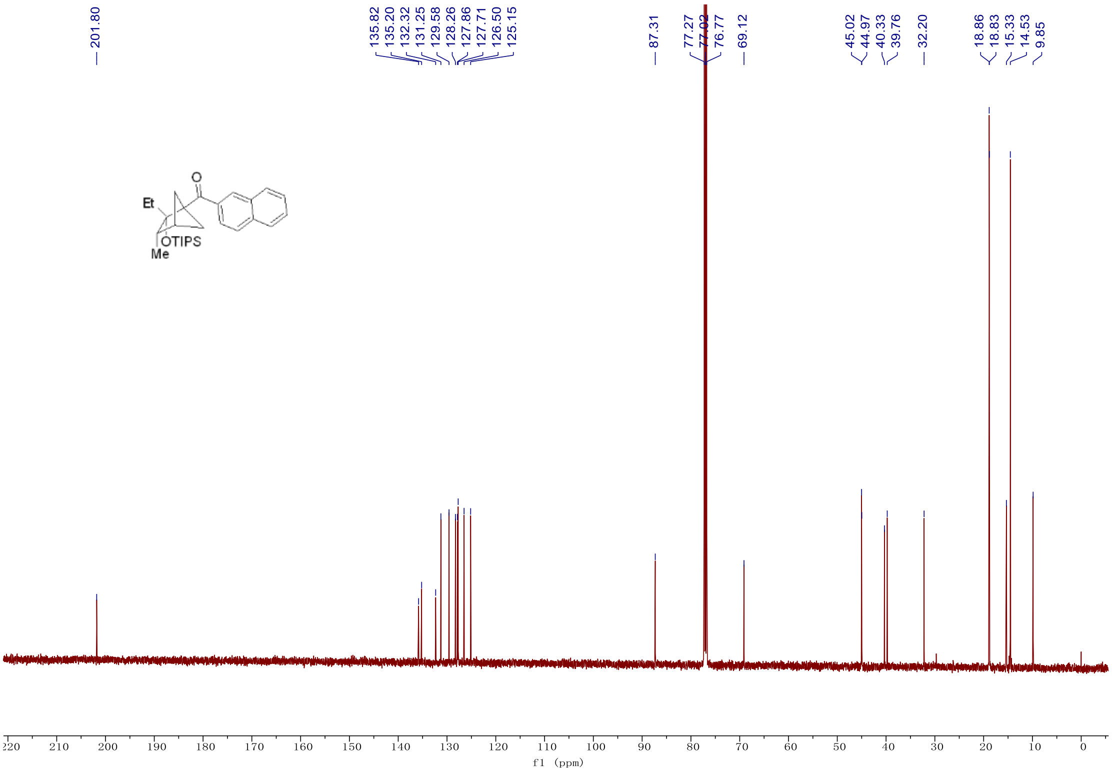

Supplementary Figure 141. ^{13}C NMR spectrum (125 MHz, $CDCl_3$) of 3ap

COSY NMR (500 MHz, $CDCl_3$)

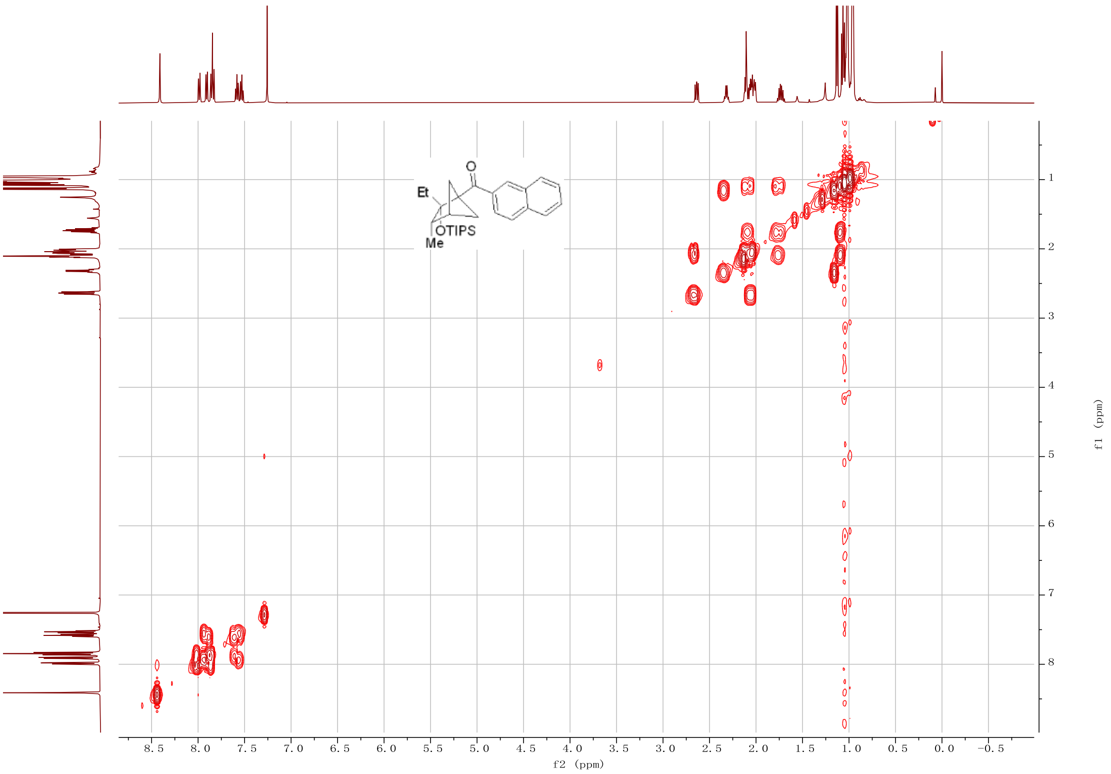

Supplementary Figure 142. COSY NMR spectrum (500 MHz, $CDCl_3$) of **3ap**

HSQC NMR (500 MHz, $CDCl_3$)

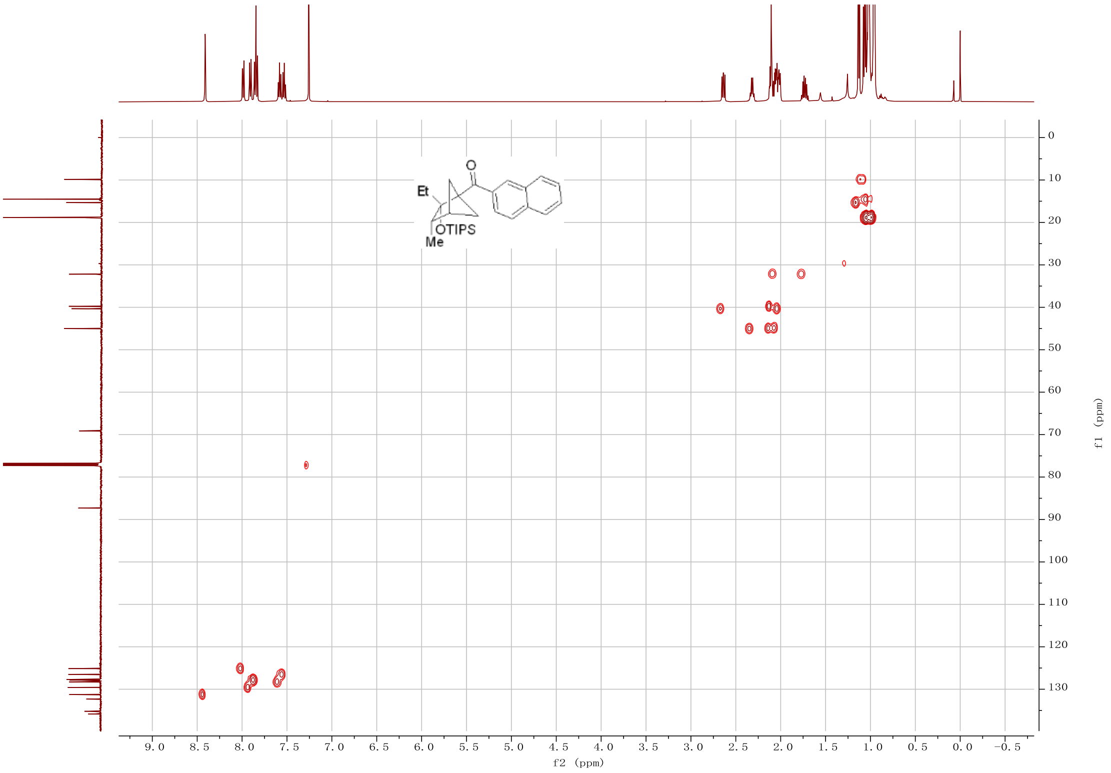

Supplementary Figure 143. HSQC NMR spectrum (500 MHz, $CDCl_3$) of **3ap**

NOESY NMR (500 MHz, $CDCl_3$)

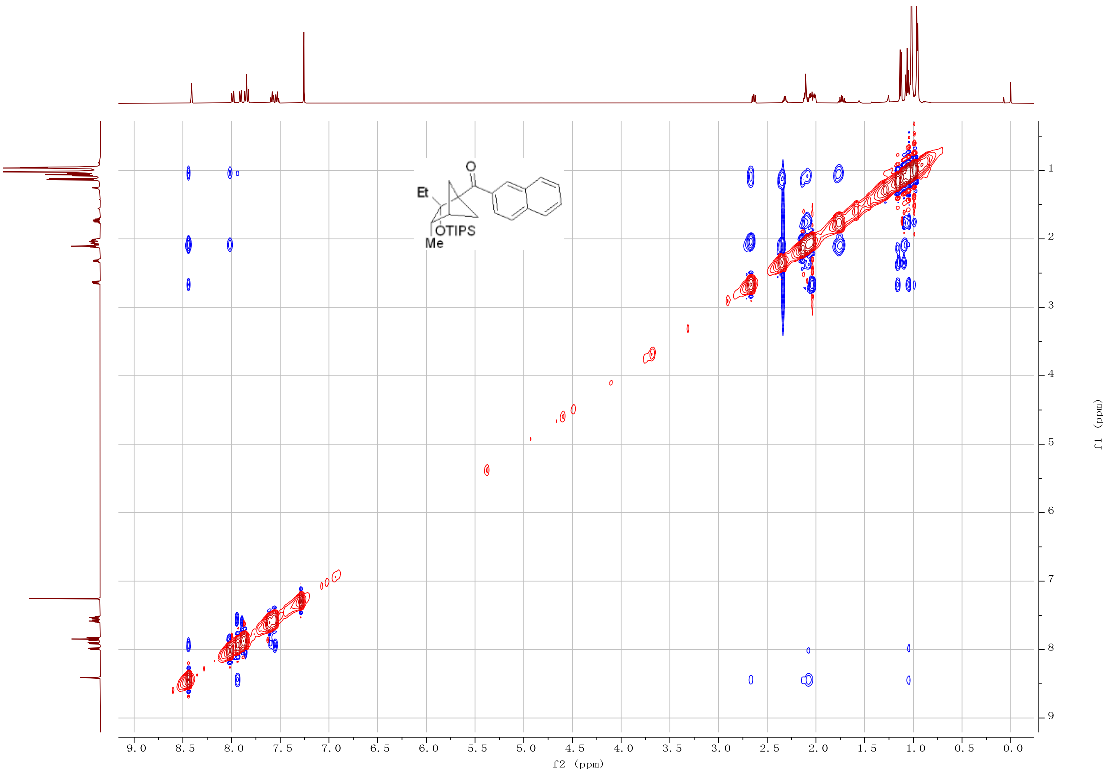

Supplementary Figure 144. NOESY NMR spectrum (500 MHz, $CDCl_3$) of **3ap**

Compound 3ap'

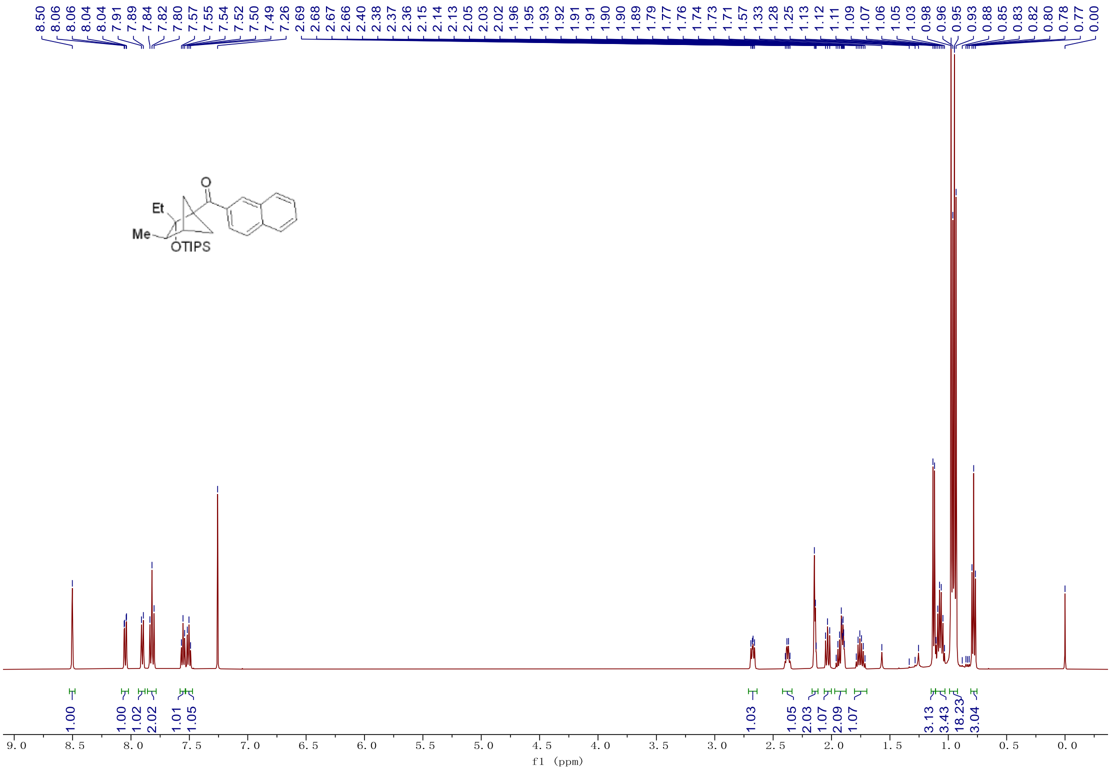

Supplementary Figure 145. ^{1}**H NMR** spectrum (500 MHz, $CDCl_3$) of **3ap'**

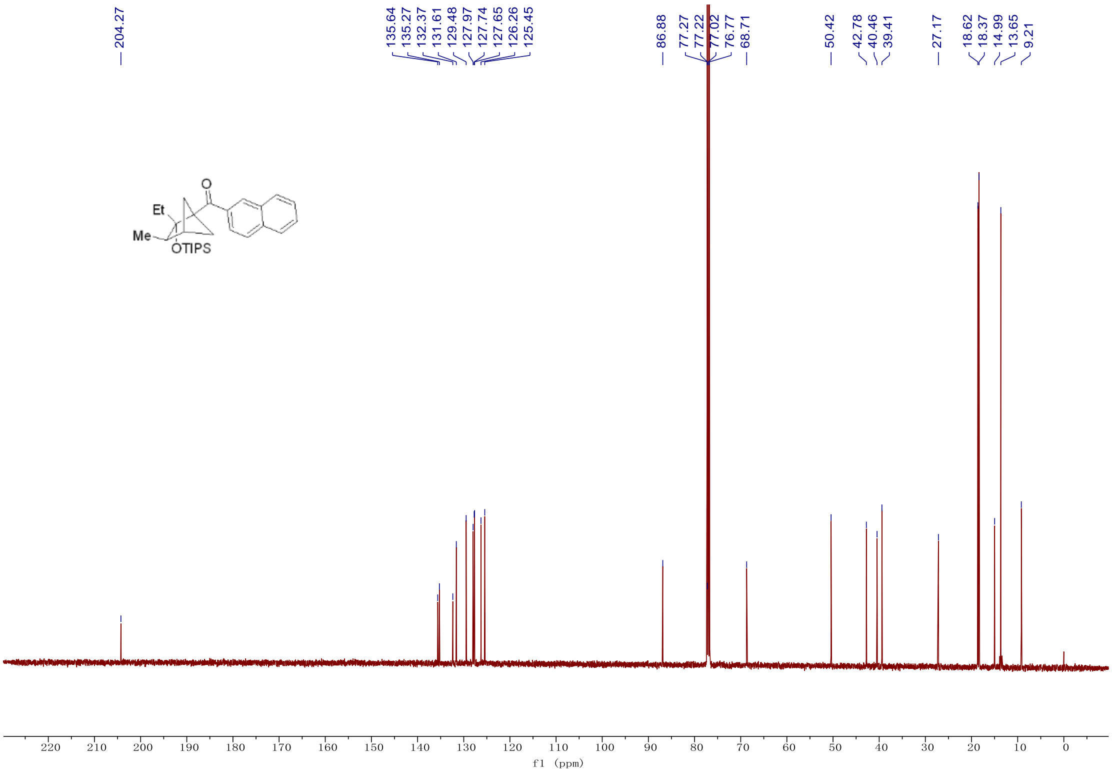

Supplementary Figure 146. ^{13}**C NMR** spectrum (125 MHz, $CDCl_3$) of **3ap'**

COSY NMR (500 MHz, $CDCl_3$)

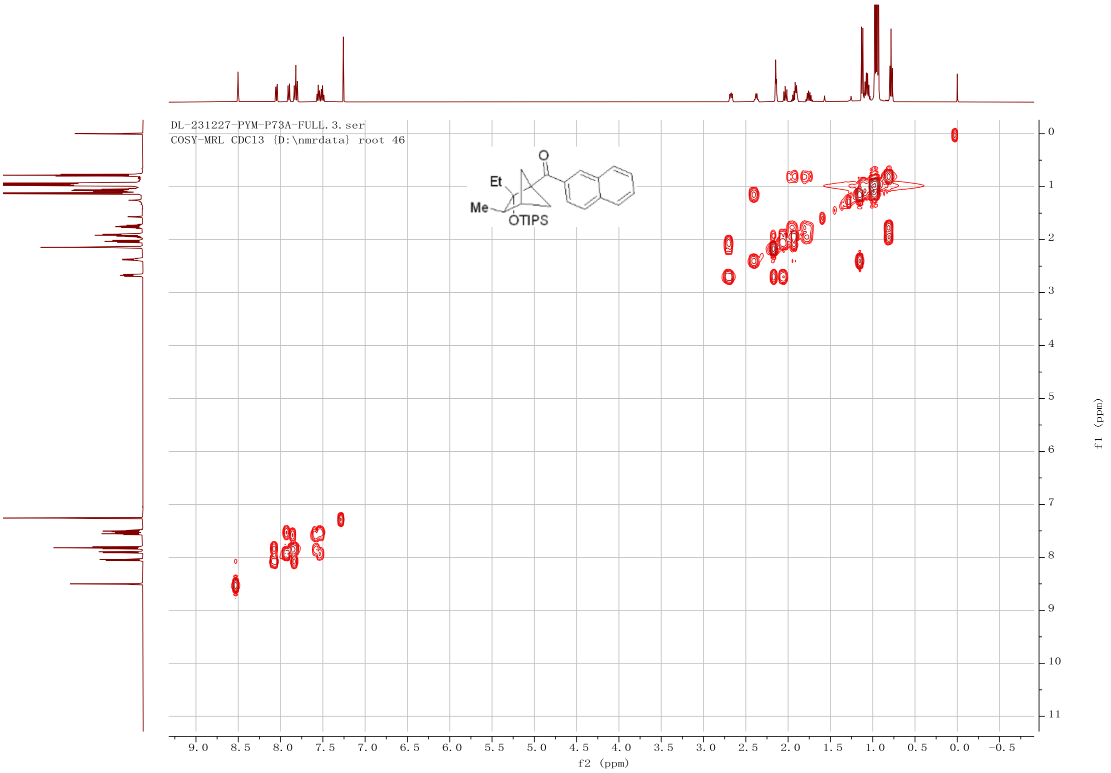

Supplementary Figure 147. COSY NMR spectrum (500 MHz, $CDCl_3$) of **3ap'**

HSQC NMR (500 MHz, $CDCl_3$)

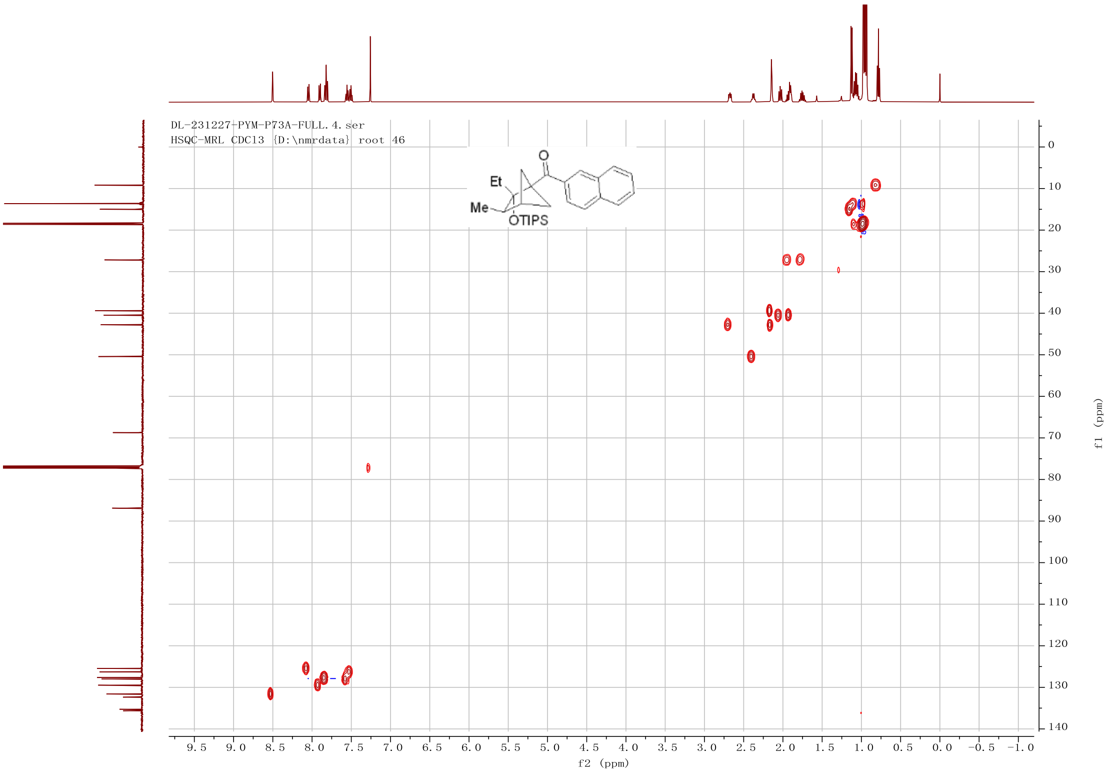

Supplementary Figure 148. HSQC NMR spectrum (500 MHz, $CDCl_3$) of **3ap'**

NOESY NMR (500 MHz, $CDCl_3$)

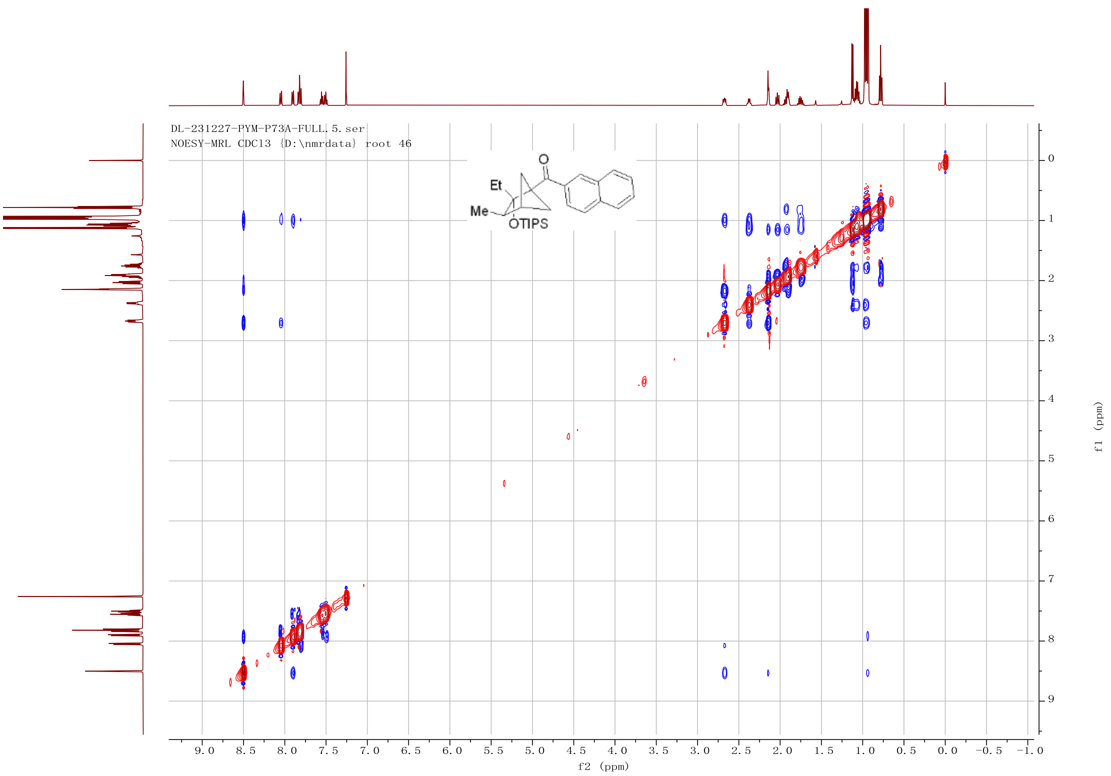

Supplementary Figure 149. NOESY NMR spectrum (500 MHz, $CDCl_3$) of **3ap'**

Compound 3aq

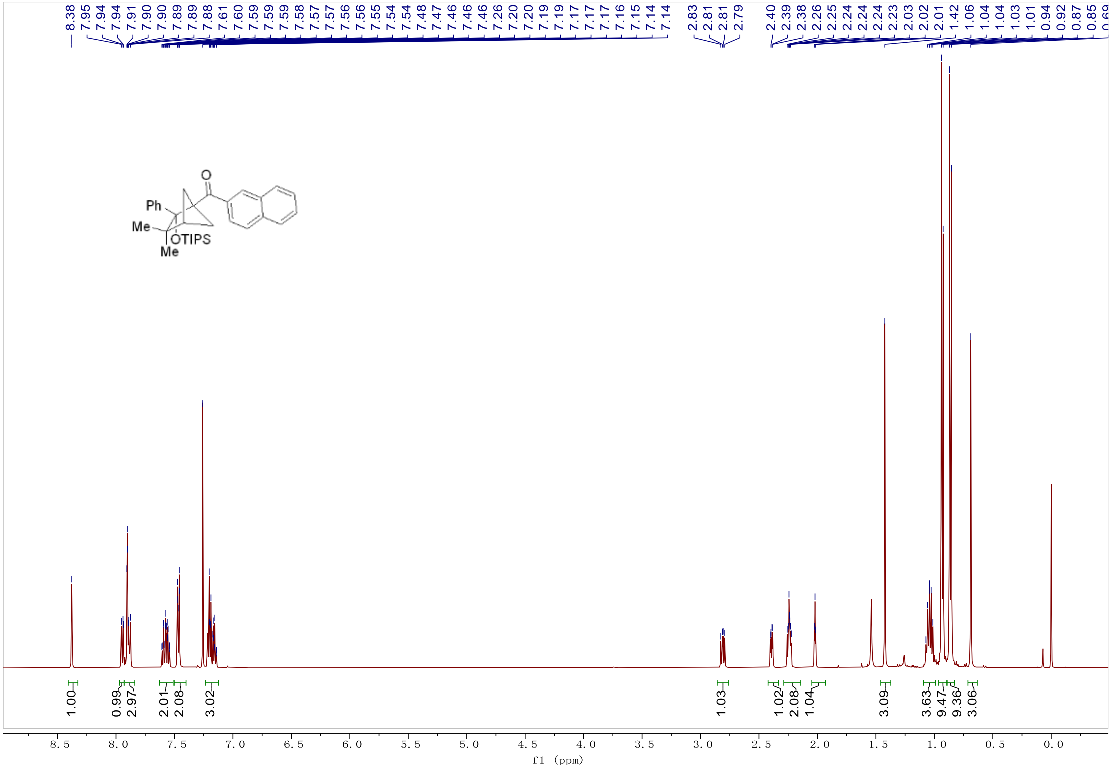

Supplementary Figure 150. ^{1}H NMR spectrum (500 MHz, $CDCl_3$) of **3aq**

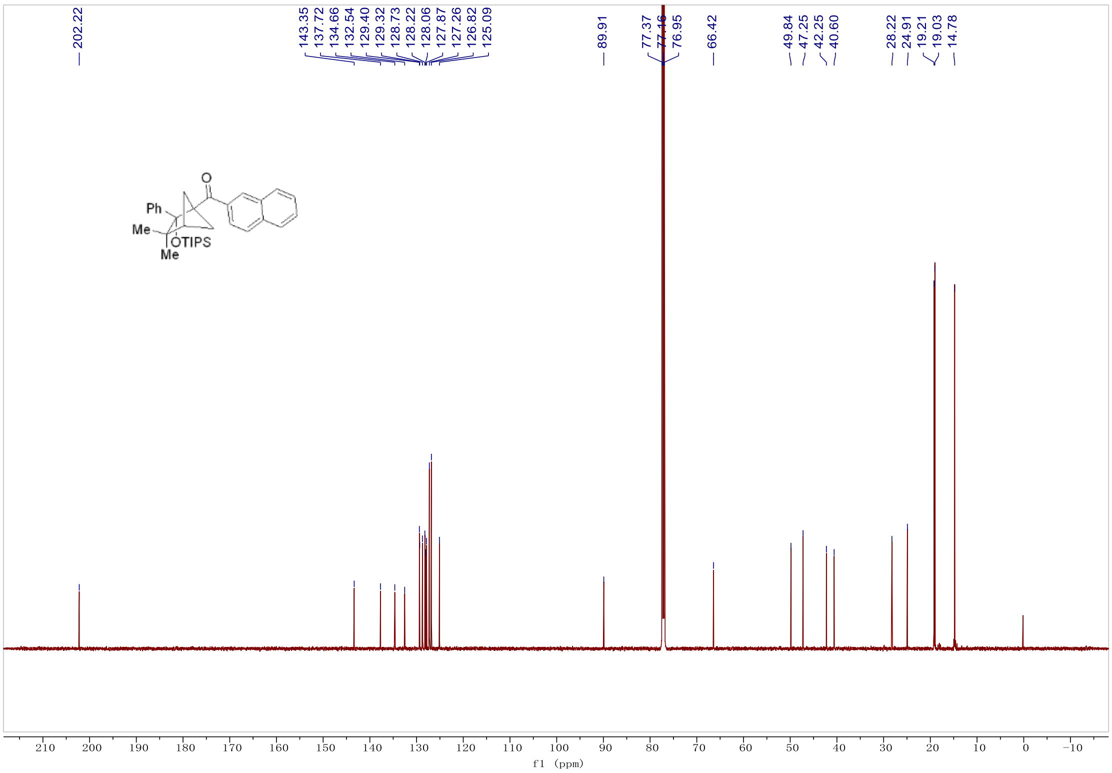

Supplementary Figure 151. ^{13}C NMR spectrum (150 MHz, $CDCl_3$) of **3aq**

Compound 3ba

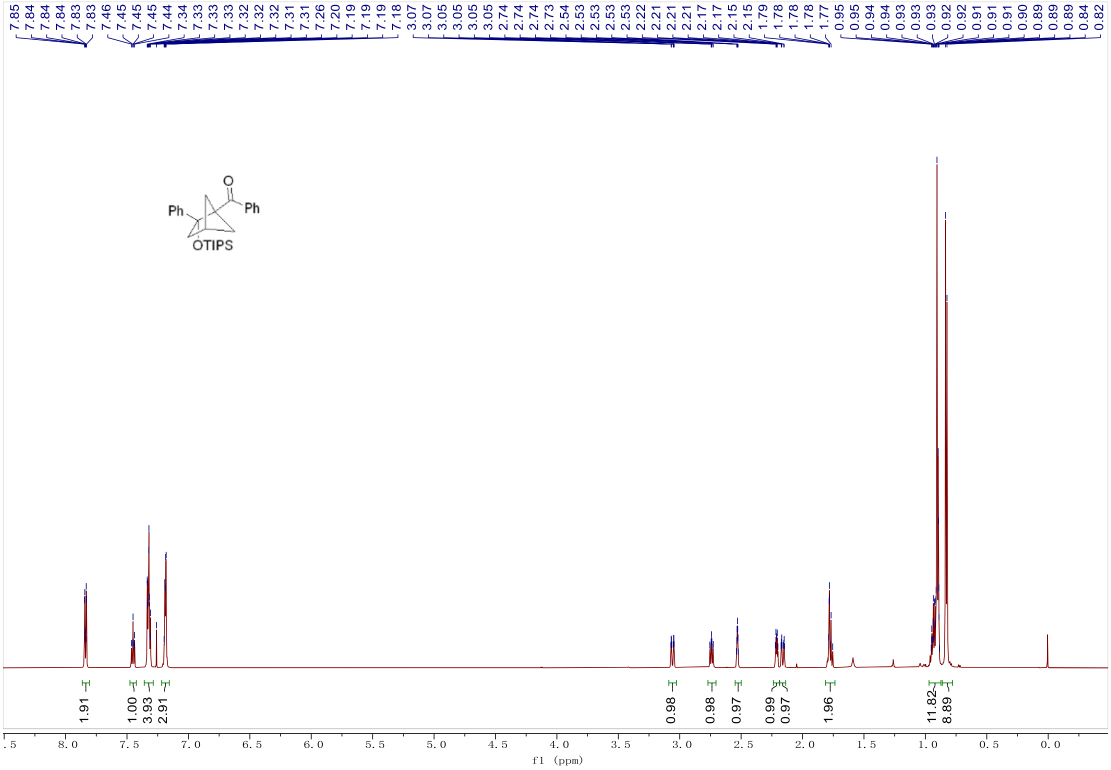

Supplementary Figure 152. ^{1}H NMR spectrum (600 MHz, $CDCl_3$) of **3ba**

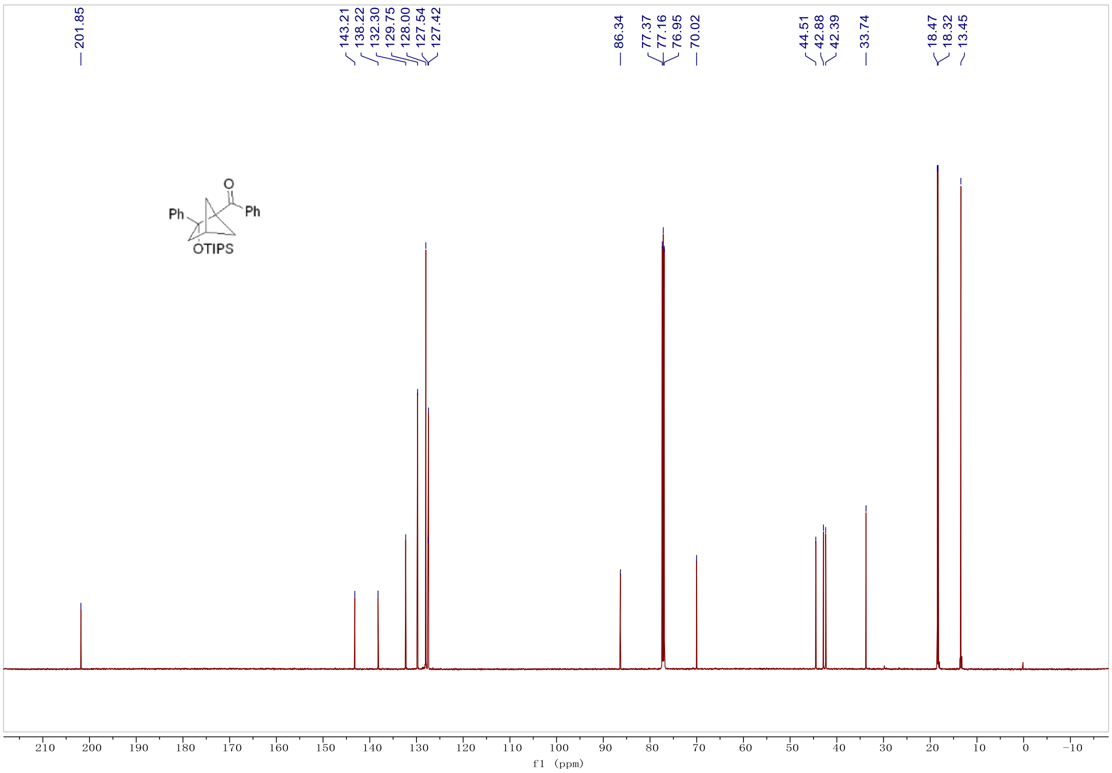

Supplementary Figure 153. ^{13}C NMR spectrum (150 MHz, $CDCl_3$) of **3ba**

Compound 3bb

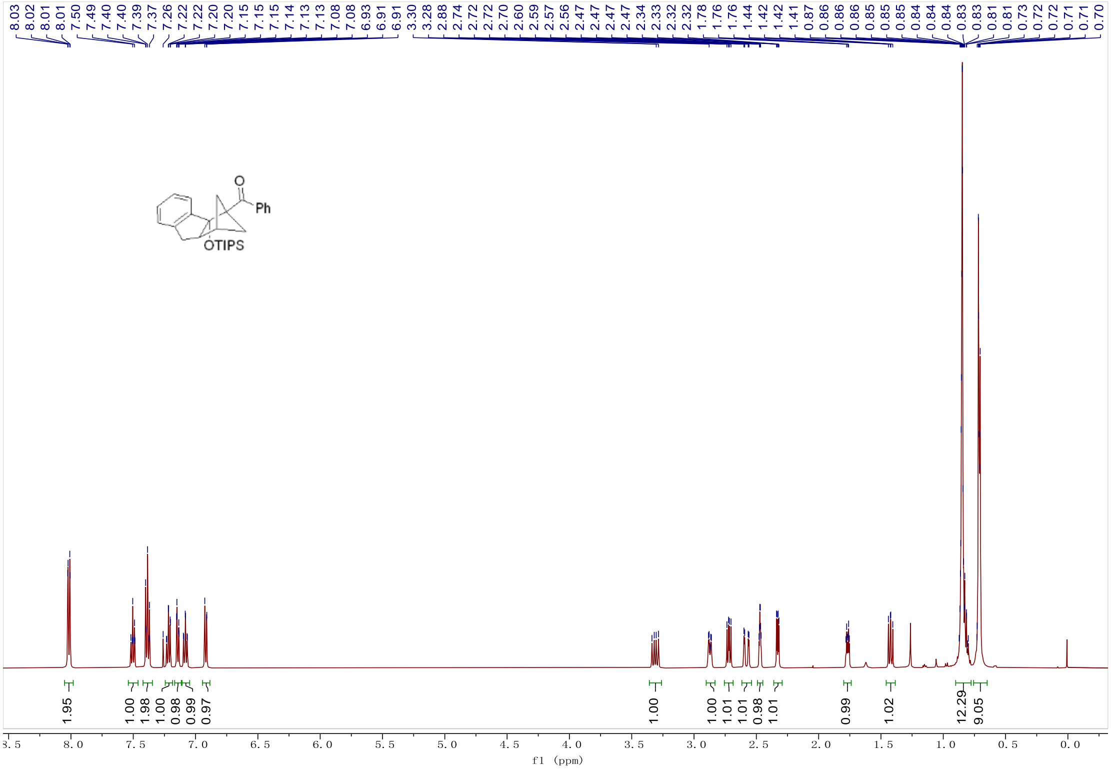

Supplementary Figure 154. ^{1}**H NMR** spectrum (500 MHz, $CDCl_3$) of **3bb**

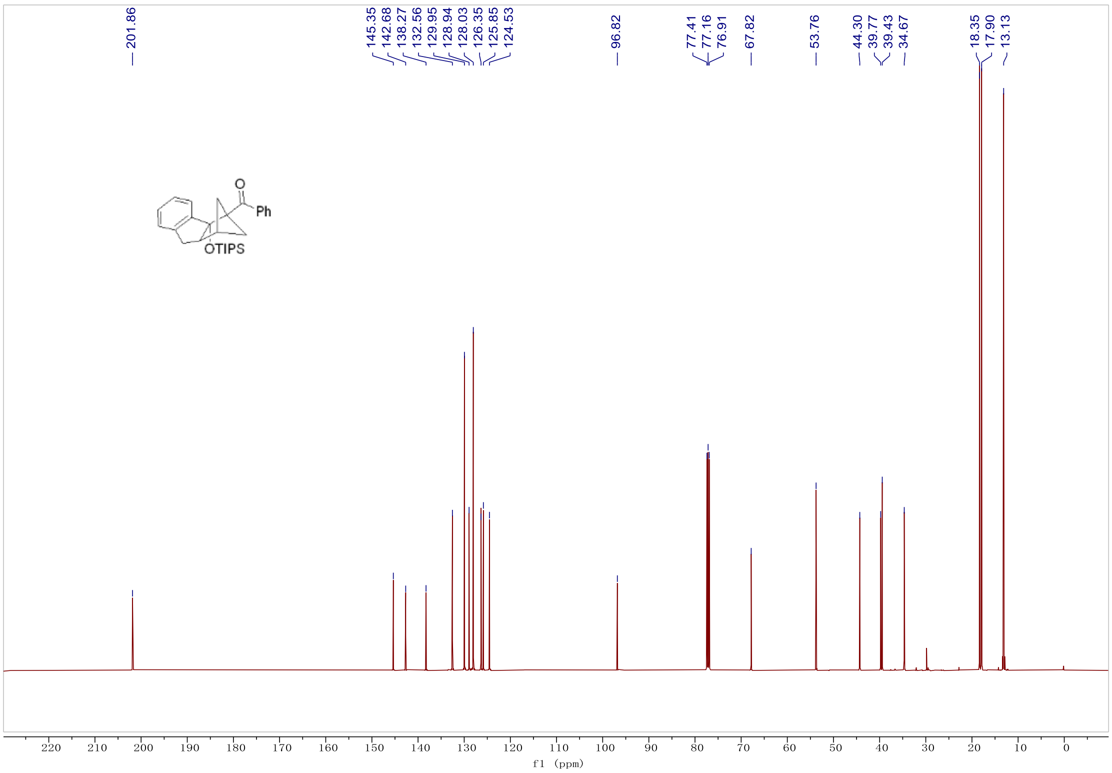

Supplementary Figure 155. ^{13}**C NMR** spectrum (125 MHz, $CDCl_3$) of **3bb**

Compound 3bc

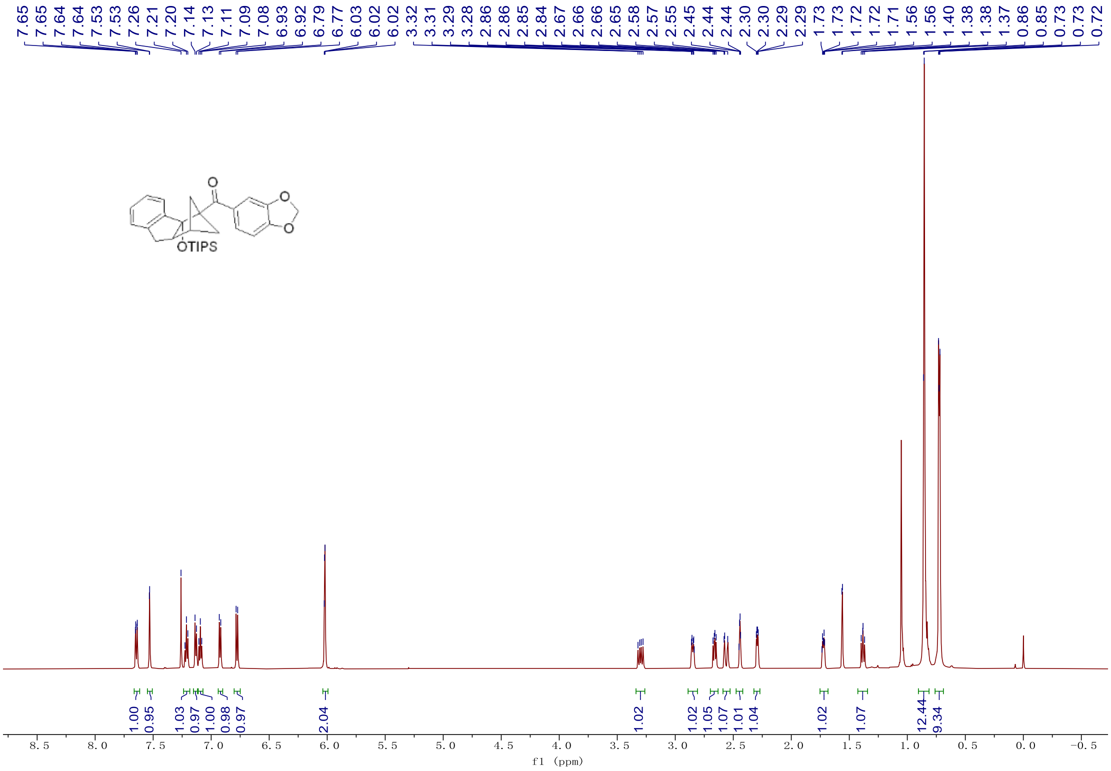

Supplementary Figure 156. ^{1}H NMR spectrum (600 MHz, $CDCl_3$) of **3bc**

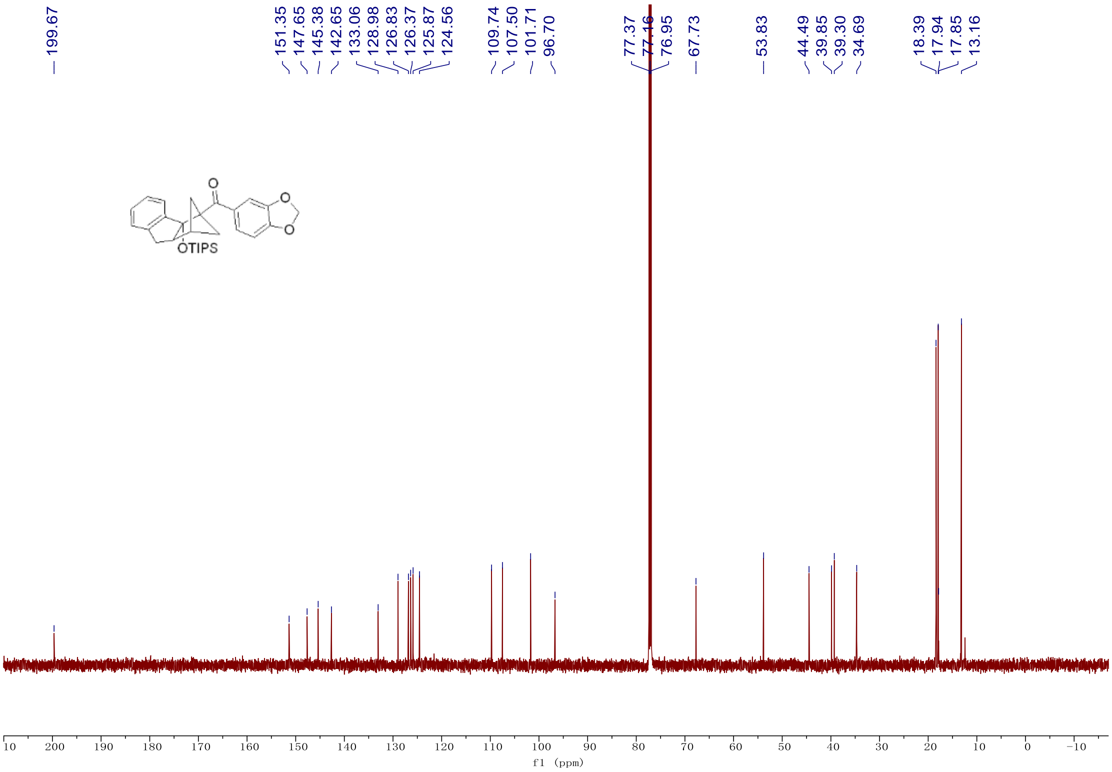

Supplementary Figure 157. ^{13}C NMR spectrum (150 MHz, $CDCl_3$) of **3bc**

Compound 3bd

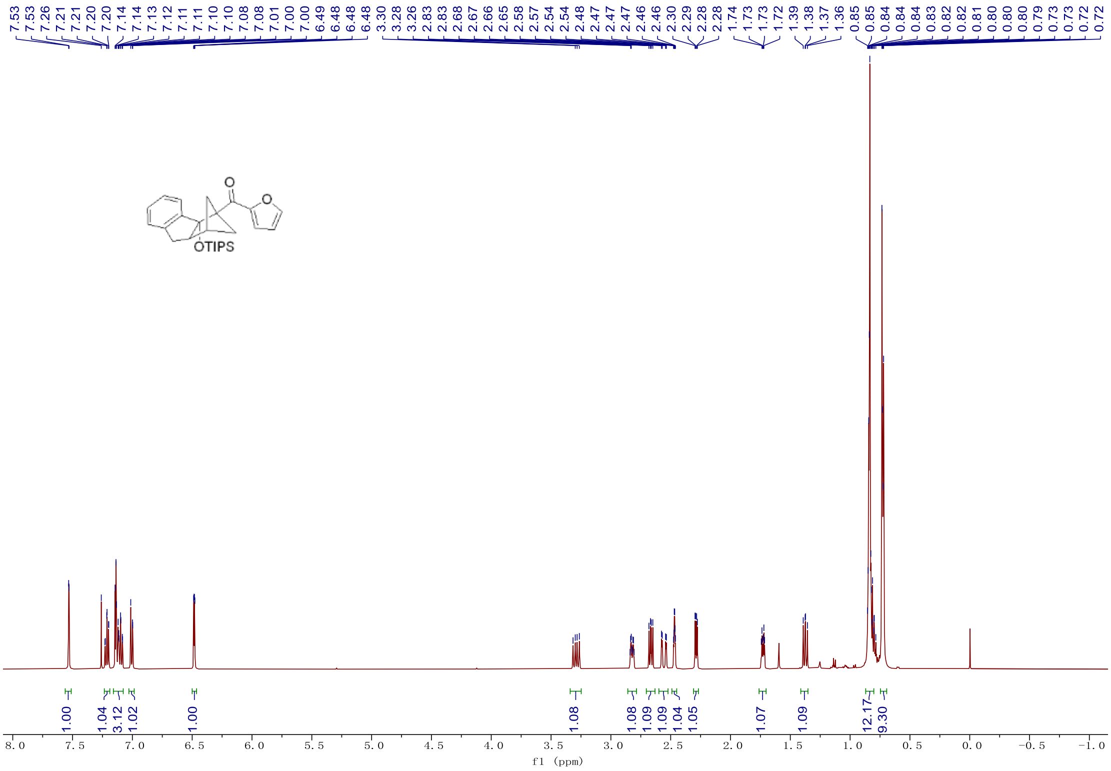

Supplementary Figure 158. ^{1}H NMR spectrum (500 MHz, CDCl$_3$) of **3bd**

^{13}C NMR (125 MHz, CDCl$_3$)

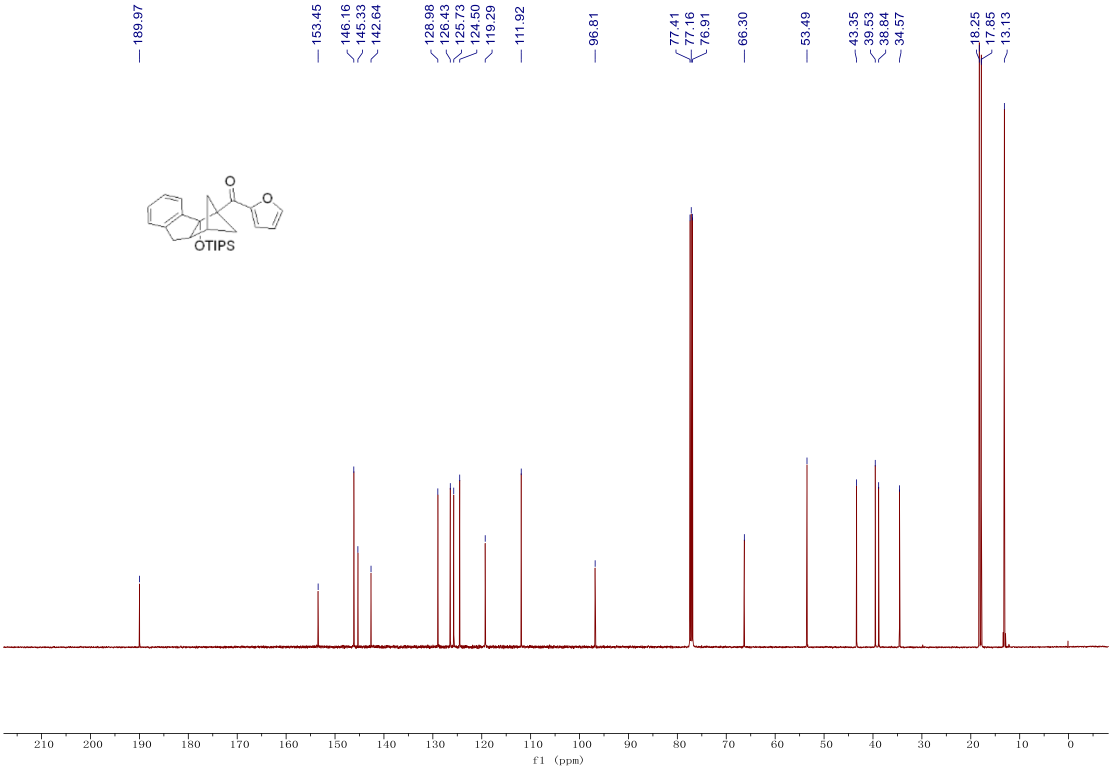

Supplementary Figure 159. ^{13}C NMR spectrum (150 MHz, CDCl$_3$) of **3bd**

Compound 3be

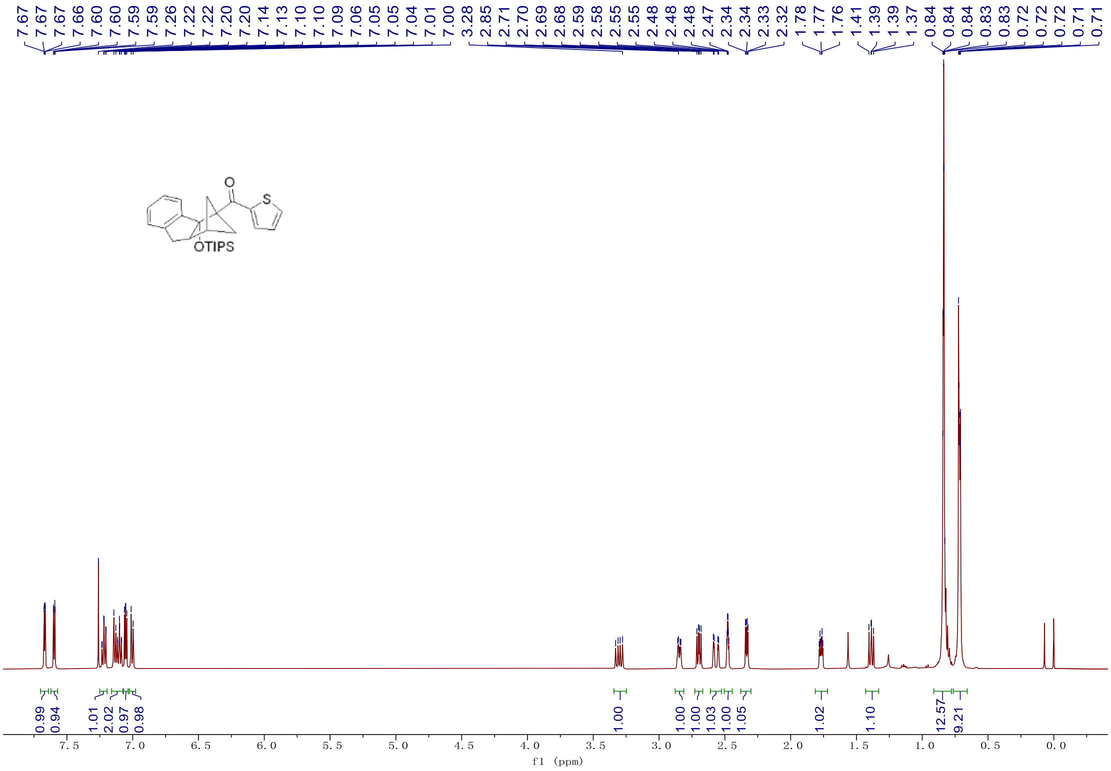

Supplementary Figure 160. ^{1}**H NMR** spectrum (500 MHz, $CDCl_3$) of **3be**

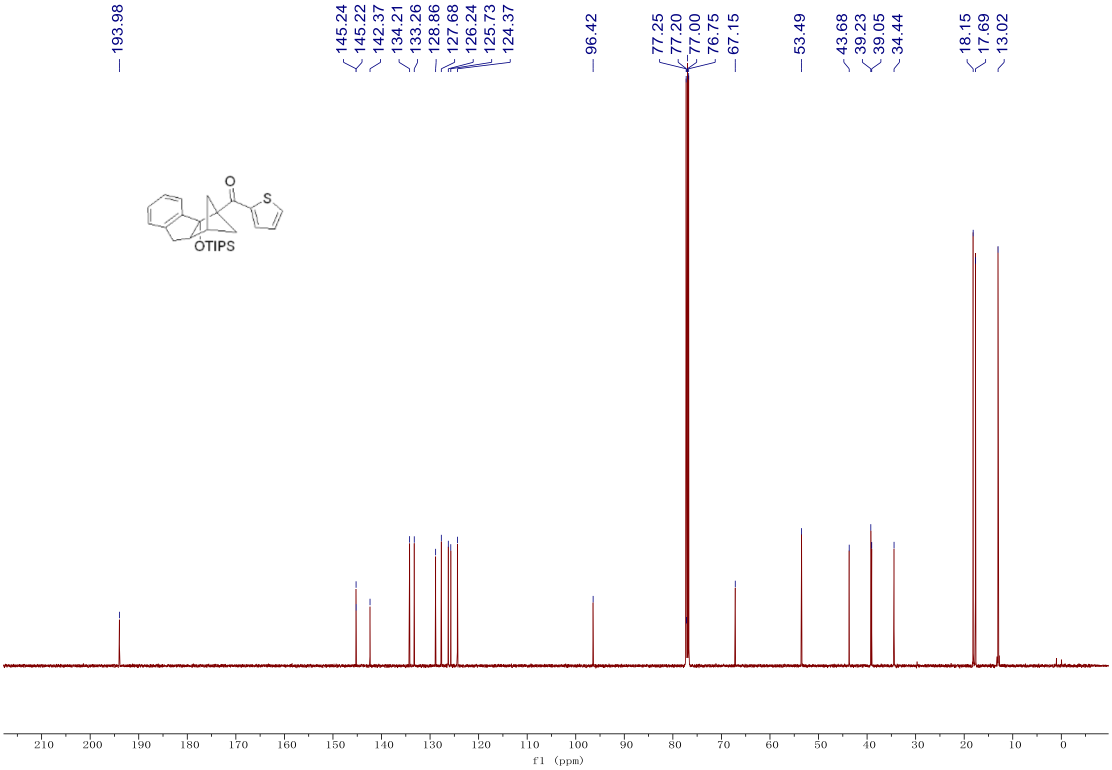

Supplementary Figure 161. ^{13}**C NMR** spectrum (125 MHz, $CDCl_3$) of **3be**

Compound 3bf

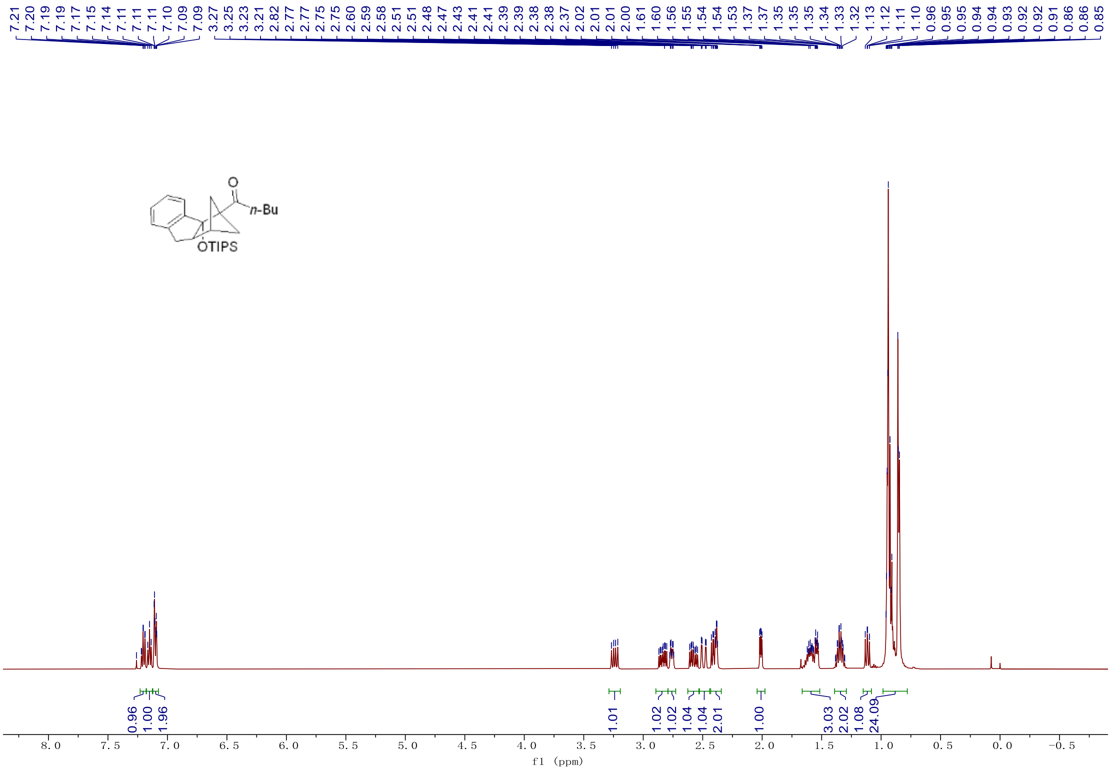

Supplementary Figure 162. ^{1}**H NMR** spectrum (500 MHz, $CDCl_3$) of **3bf**

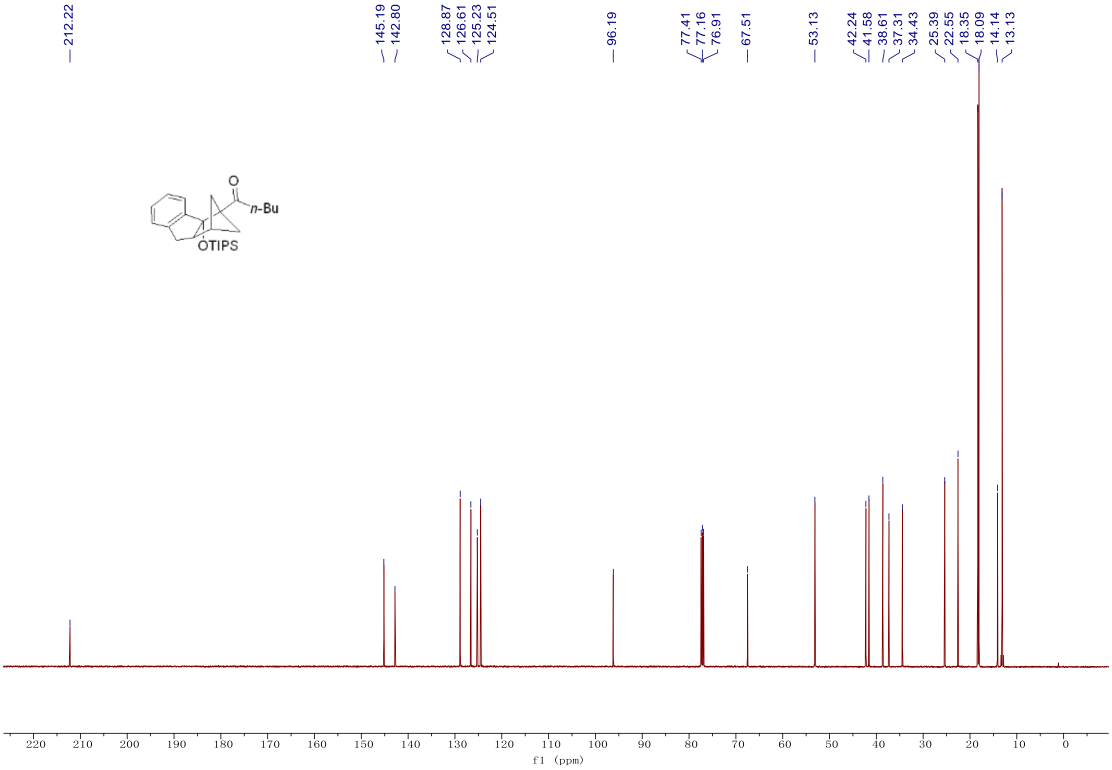

Supplementary Figure 163. ^{13}**C NMR** spectrum (125 MHz, $CDCl_3$) of **3bf**

Compound 3bg

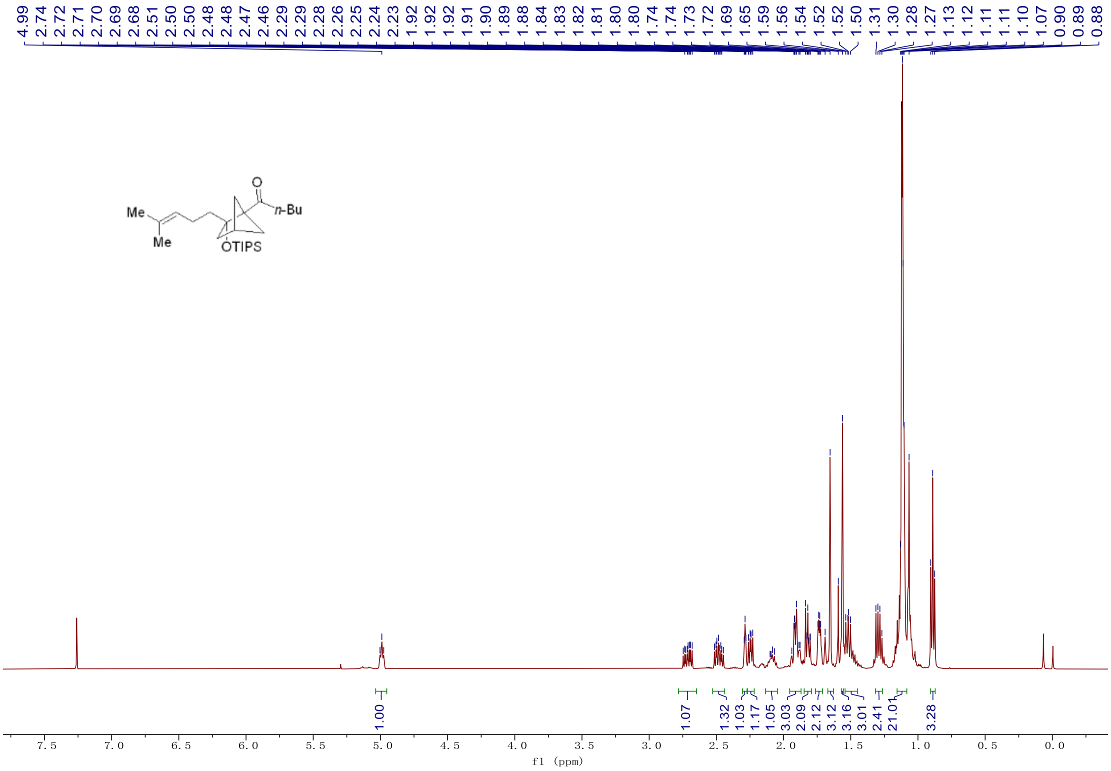

Supplementary Figure 164. **^{1}H NMR** spectrum (500 MHz, $CDCl_3$) of **3bg**

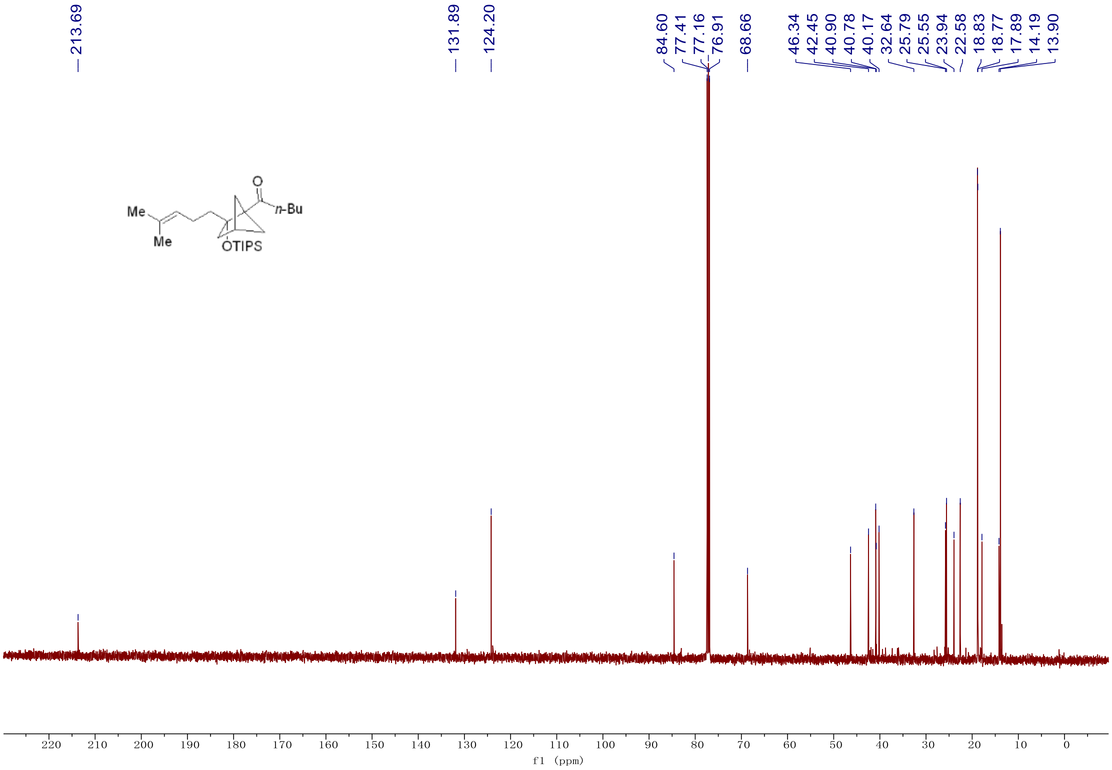

Supplementary Figure 165. **^{13}C NMR** spectrum (125 MHz, $CDCl_3$) of **3bg**

Compound 3bh

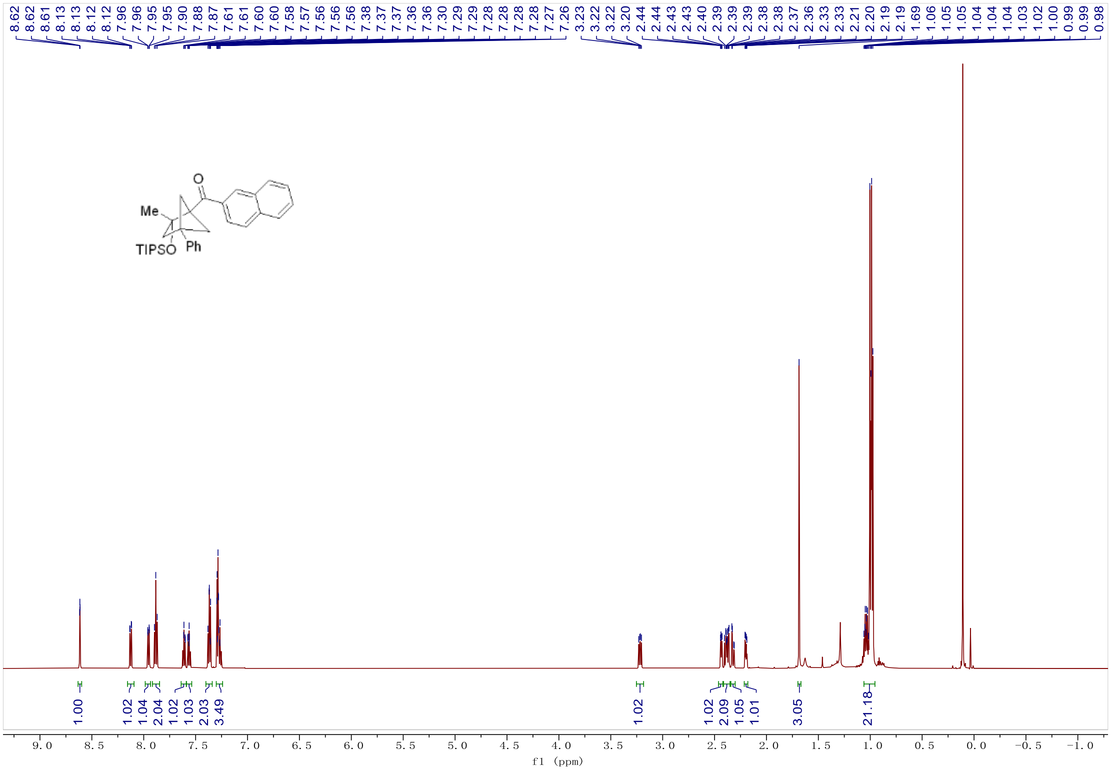

Supplementary Figure 166. **^{1}H NMR** spectrum (600 MHz, $CDCl_3$) of **3bh**

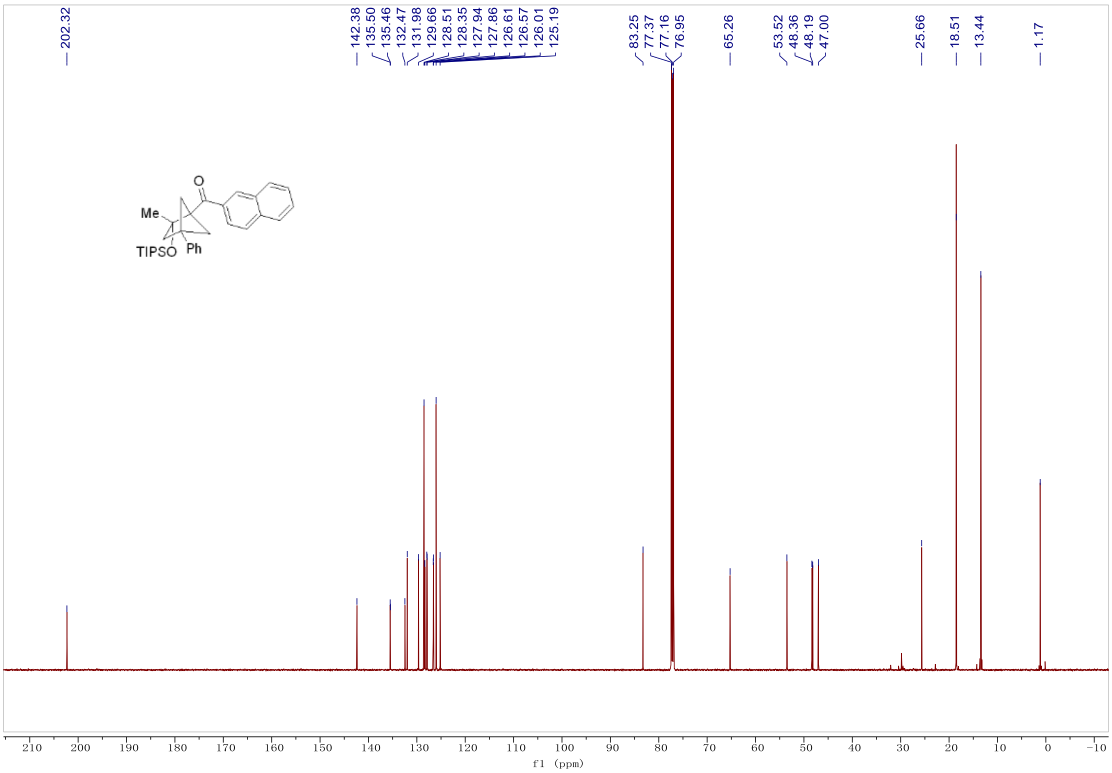

Supplementary Figure 167. **^{13}C NMR** spectrum (150 MHz, $CDCl_3$) of **3bh**

Compound 3bi

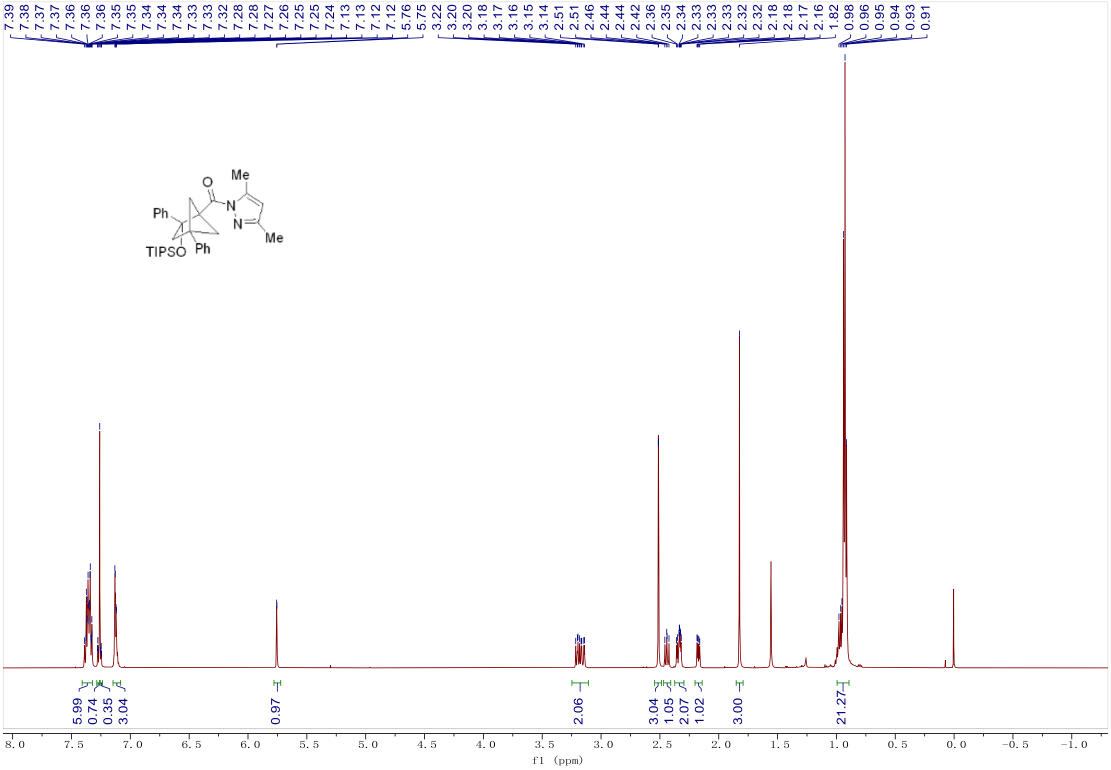

Supplementary Figure 168. ^{1}**H NMR** spectrum (500 MHz, $CDCl_3$) of **3bi**

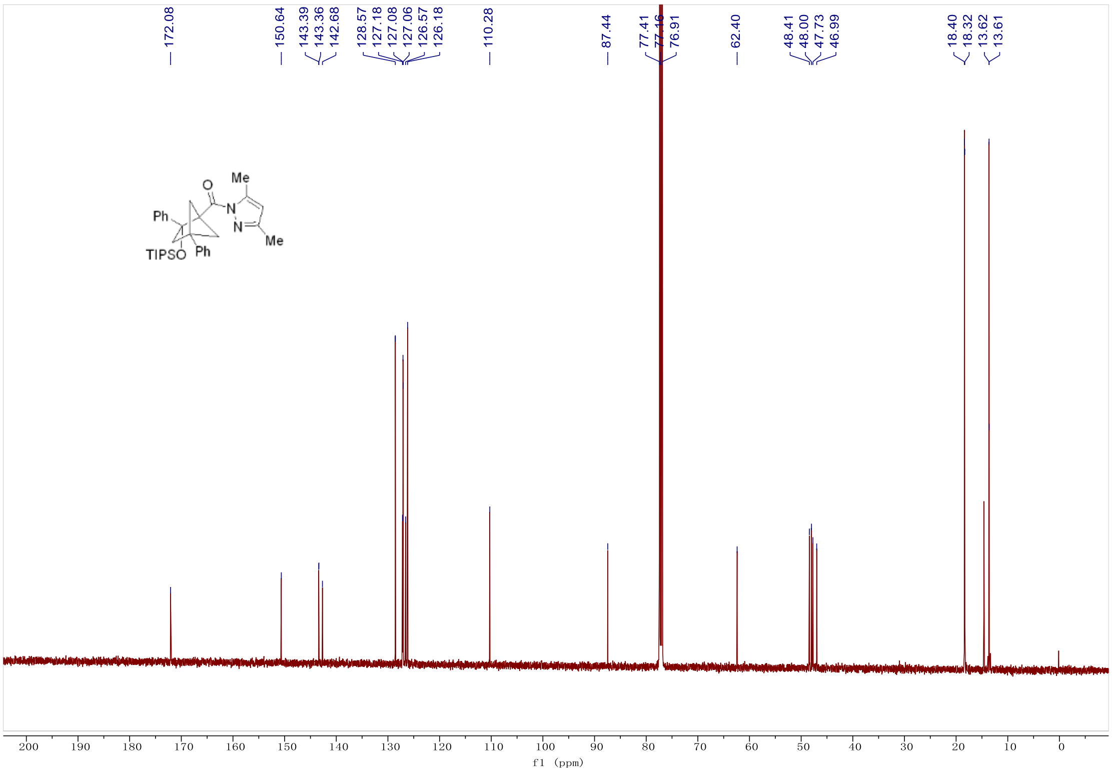

Supplementary Figure 169. ^{13}**C NMR** spectrum (125 MHz, $CDCl_3$) of **3bi**

Compound 5a

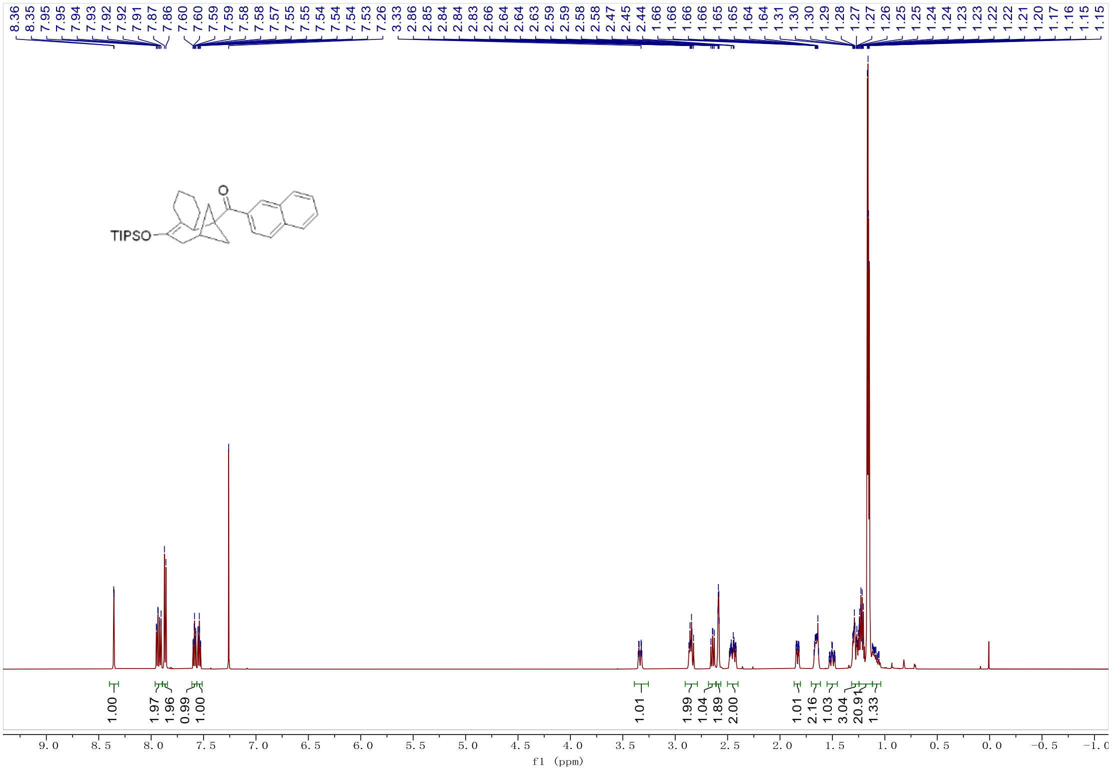

Supplementary Figure 170. **^{1}H NMR** spectrum (500 MHz, $CDCl_3$) of **5a**

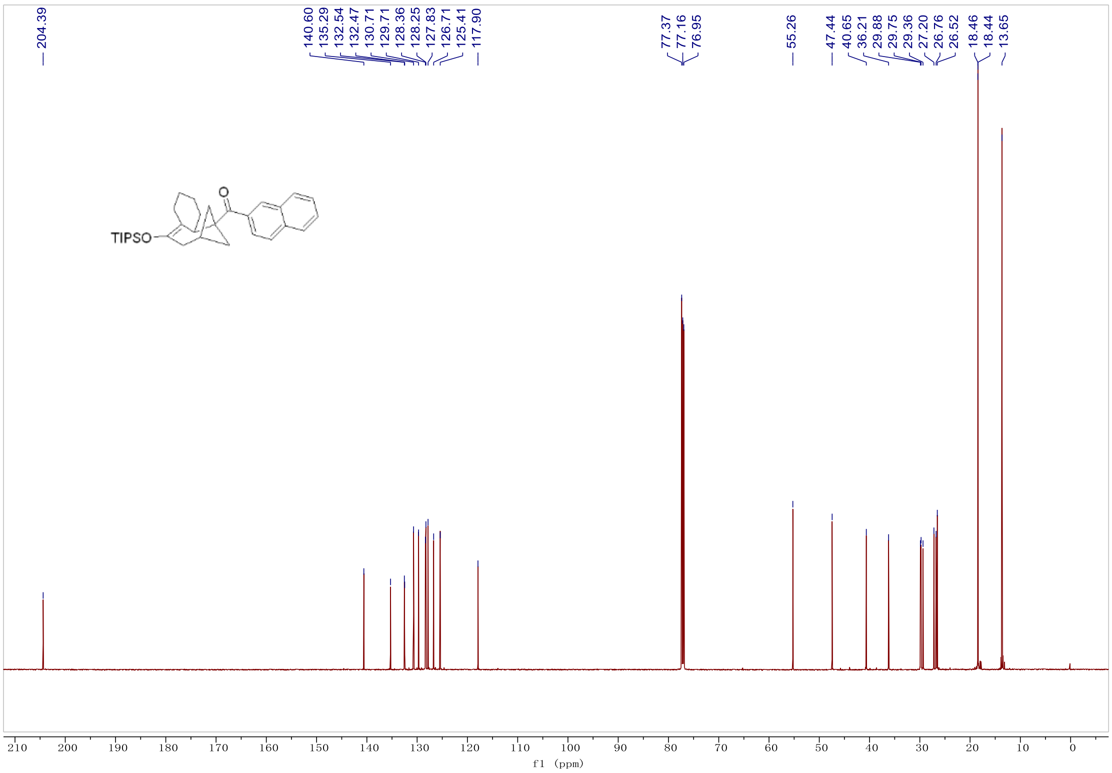

Supplementary Figure 171. **^{13}C NMR** spectrum (125 MHz, $CDCl_3$) of **5a**

COSY NMR

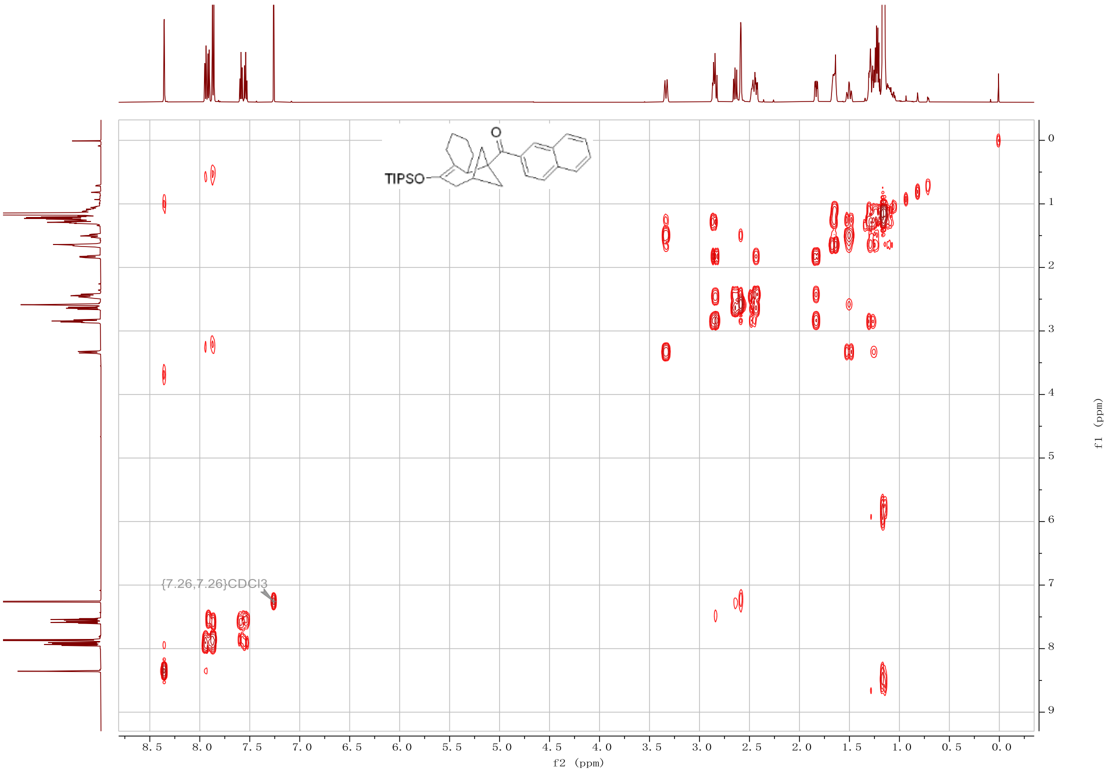

Supplementary Figure 172. COSY NMR spectrum (500 MHz, $CDCl_3$) of **5a**

HSQC NMR

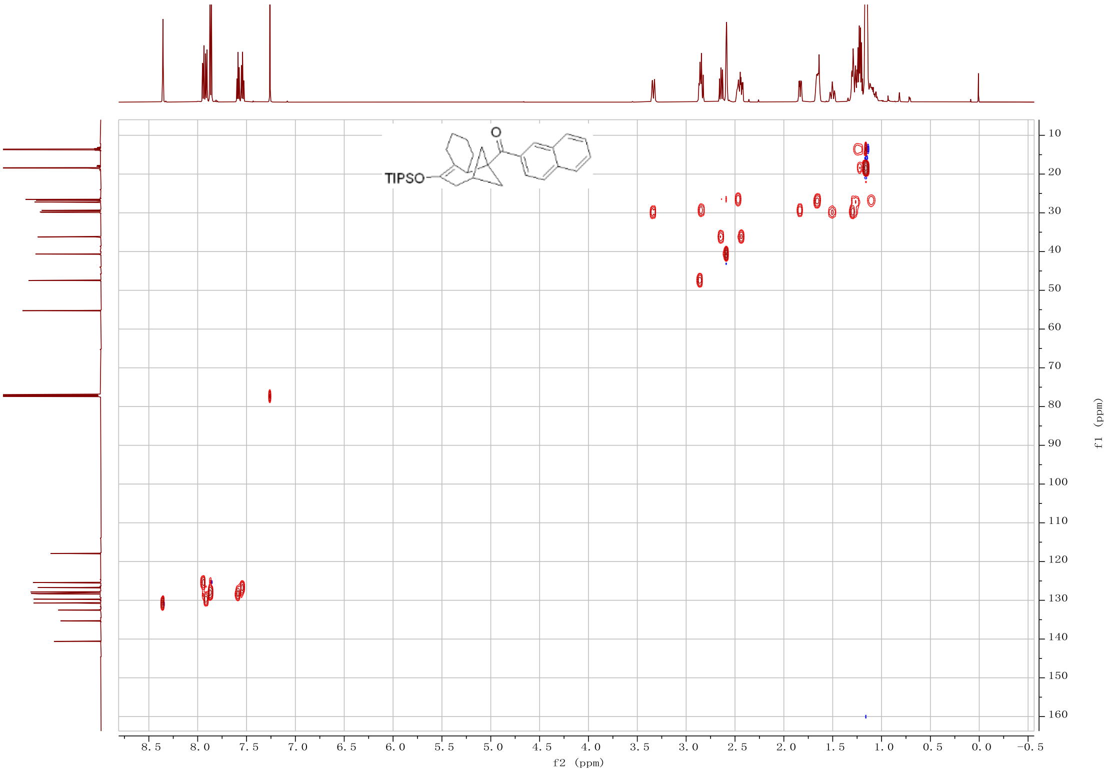

Supplementary Figure 173. HSQC NMR spectrum (500 MHz, $CDCl_3$) of **5a**

HMBC NMR

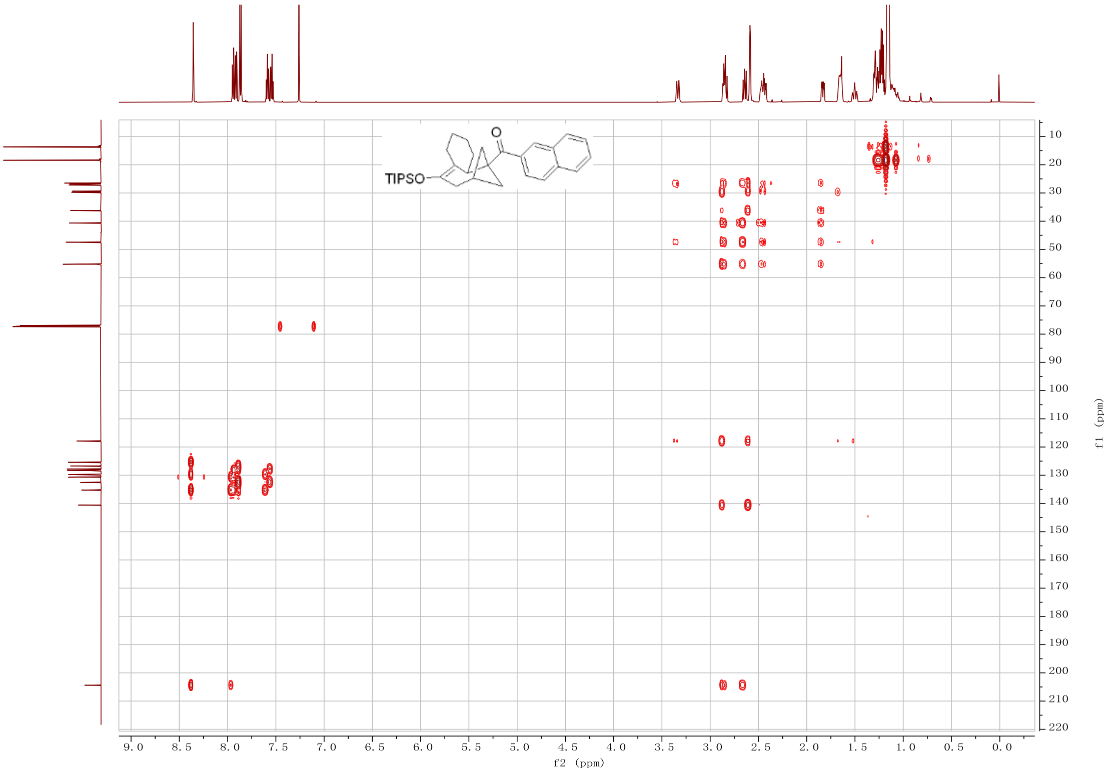

Supplementary Figure 174. HMBC NMR spectrum (500 MHz, $CDCl_3$) of **5a**

Compound 5b

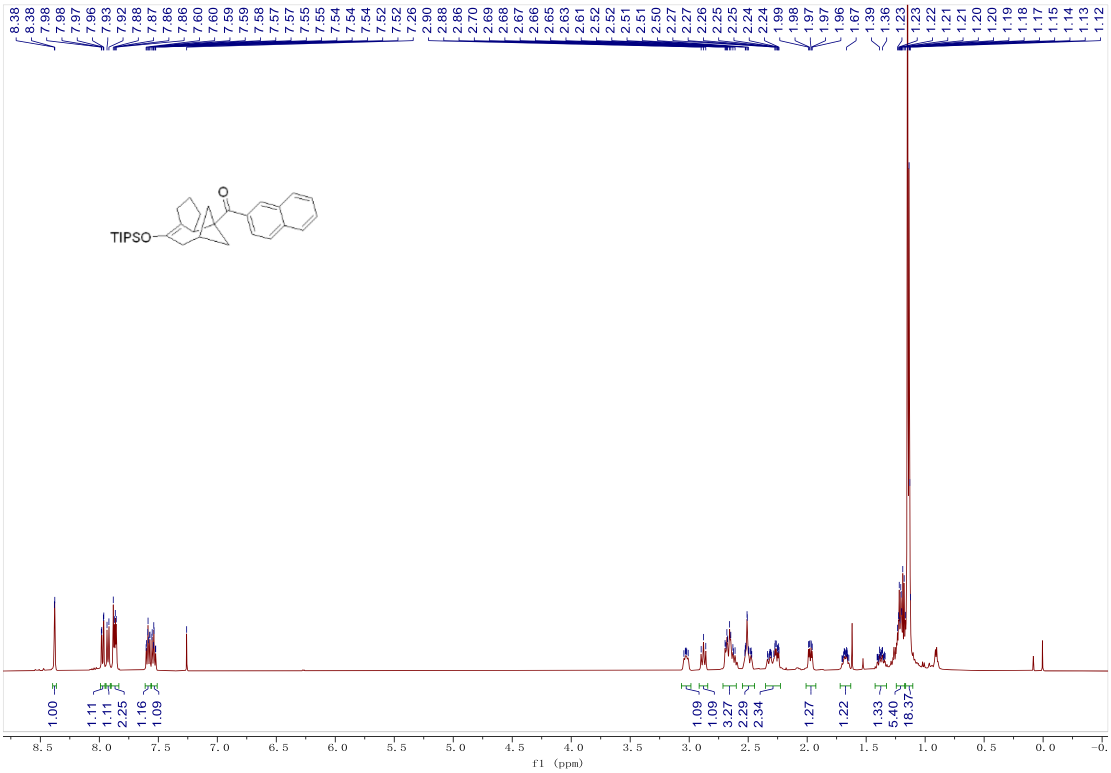

Supplementary Figure 175. ^{1}**H NMR** spectrum (500 MHz, $CDCl_3$) of **5b**

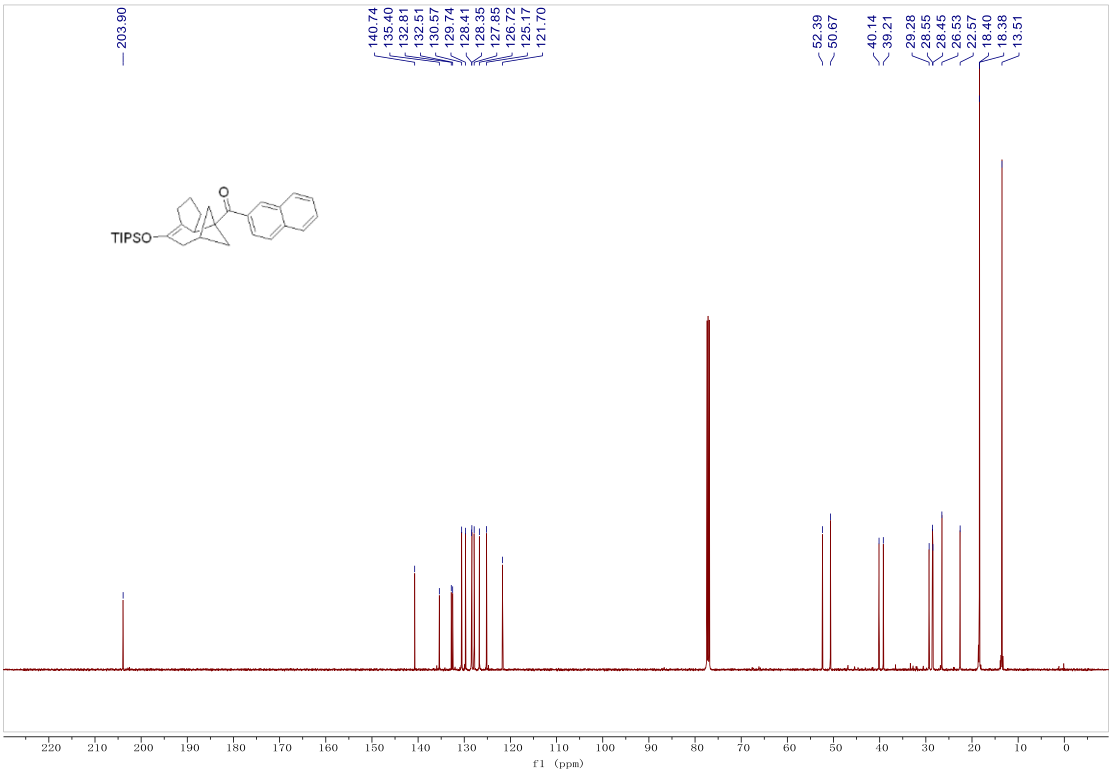

Supplementary Figure 176. ^{13}**C NMR** spectrum (125 MHz, $CDCl_3$) of **5b**

Compound 5c

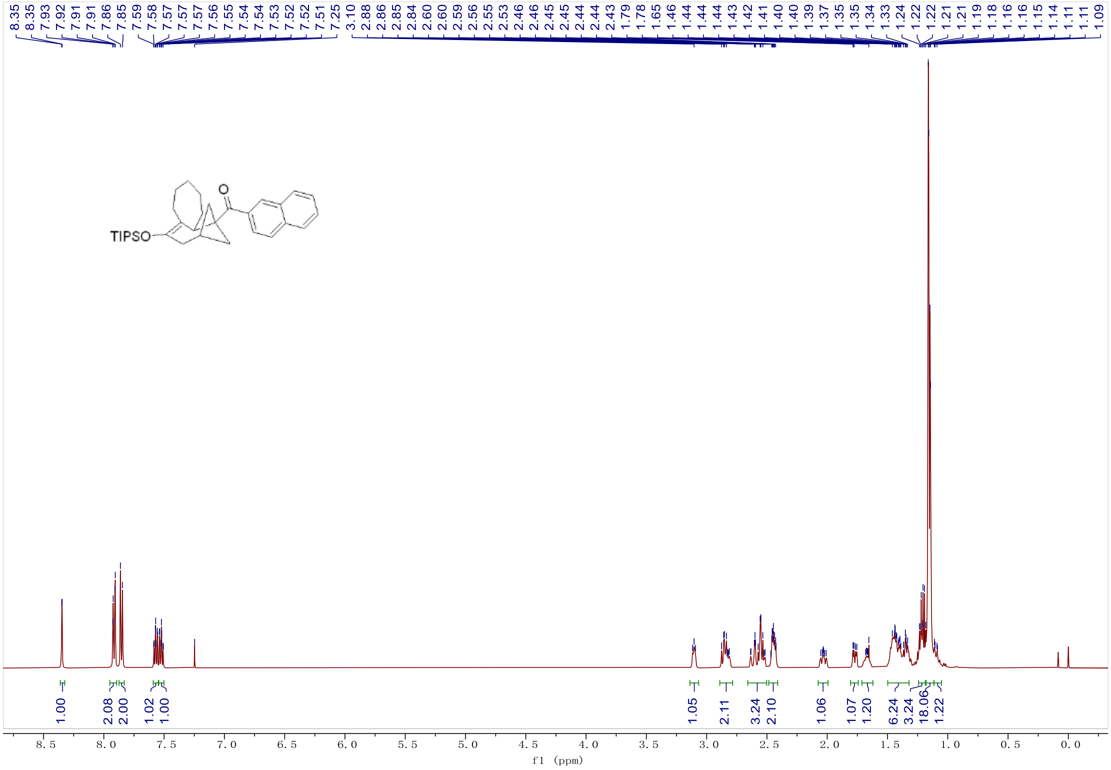

Supplementary Figure 177. ^{1}H NMR spectrum (500 MHz, $CDCl_3$) of **5c**

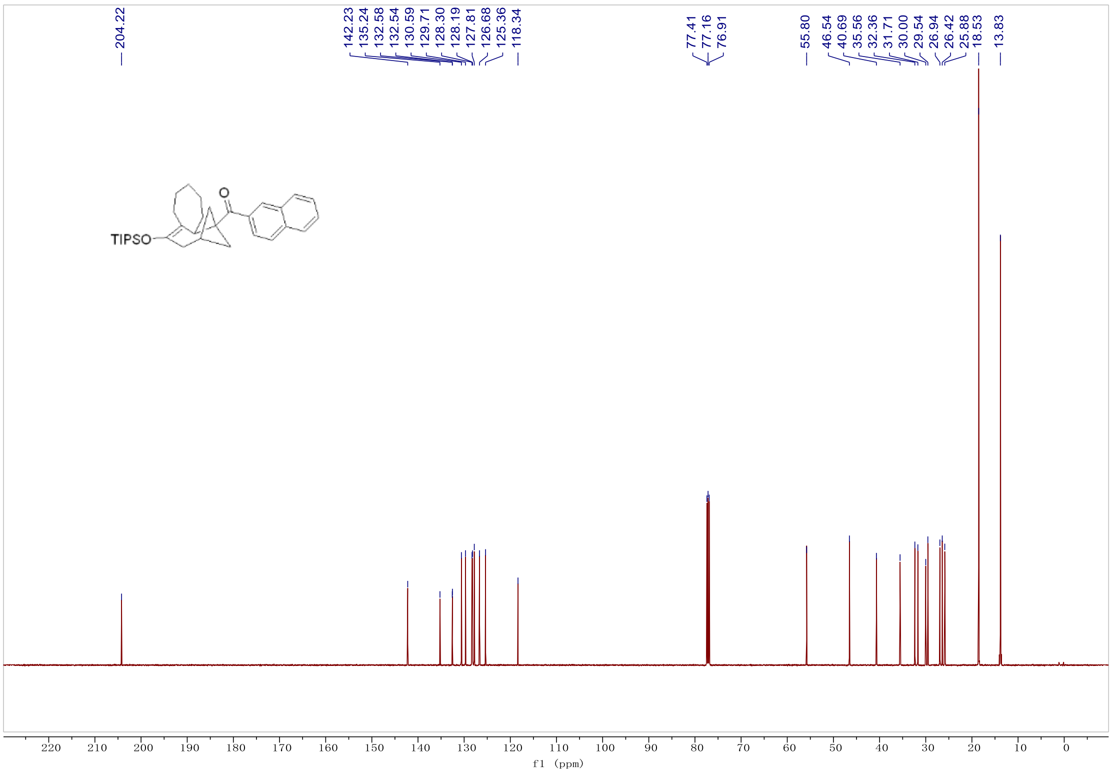

Supplementary Figure 178. ^{13}C NMR spectrum (125 MHz, $CDCl_3$) of **5c**

Compound 5d

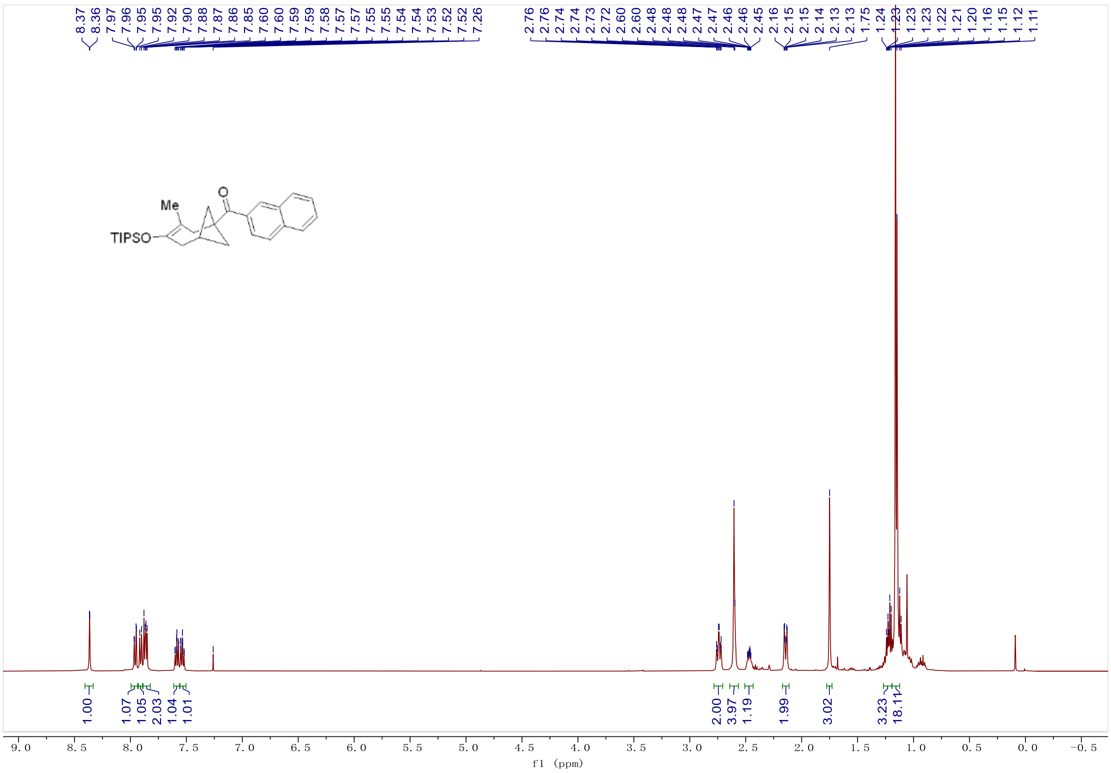

Supplementary Figure 179. ^{1}H NMR spectrum (500 MHz, $CDCl_3$) of **5d**

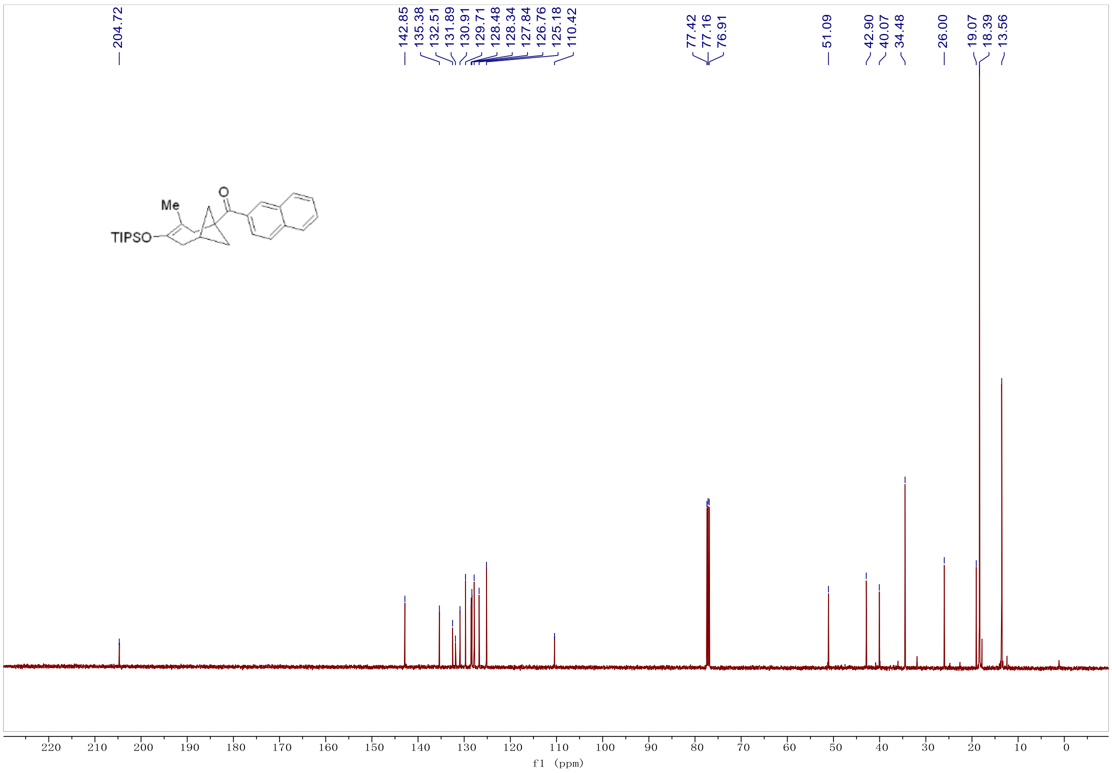

Supplementary Figure 180. ^{13}C NMR spectrum (125 MHz, $CDCl_3$) of **5d**

Compound 5e

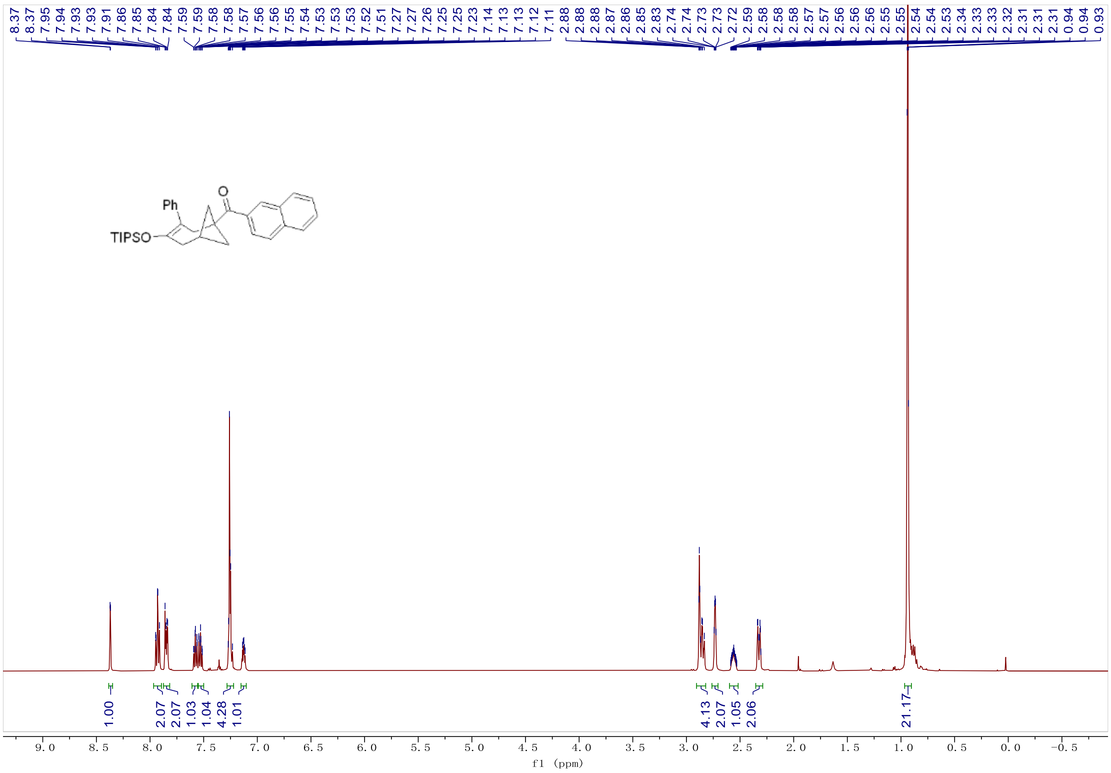

Supplementary Figure 181. ¹H NMR spectrum (500 MHz, $CDCl_3$) of **5e**

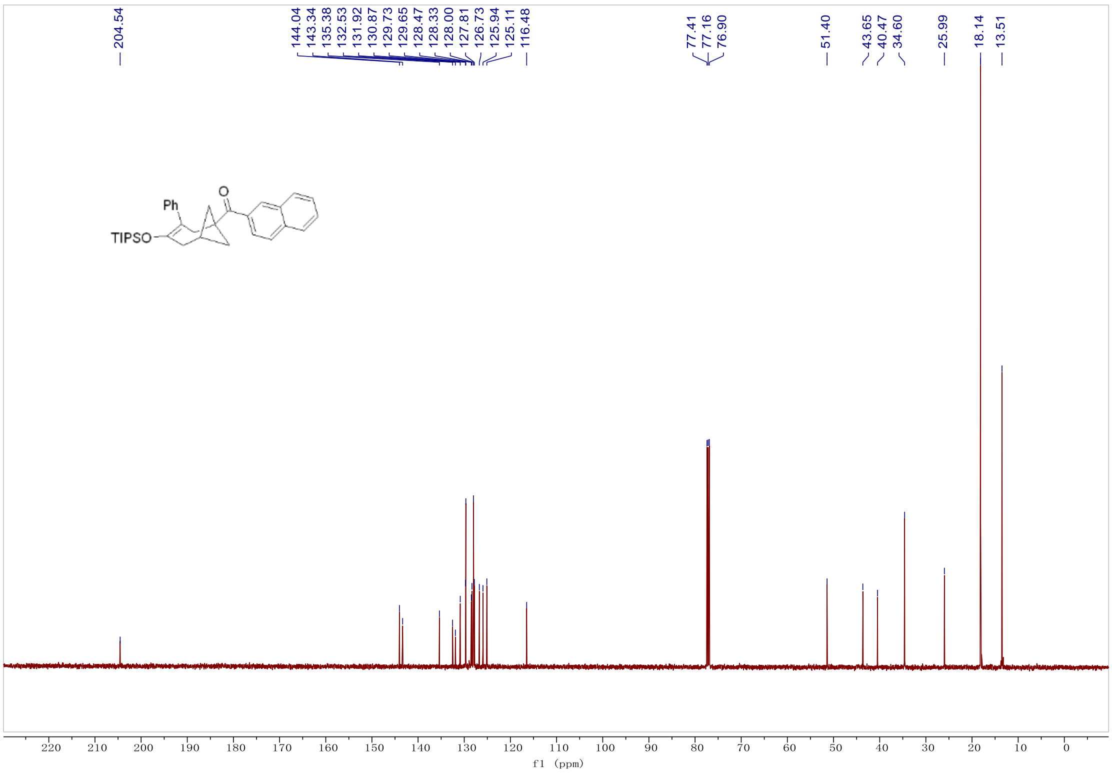

Supplementary Figure 182. ¹³C NMR spectrum (125 MHz, $CDCl_3$) of **5e**

Compound 5f

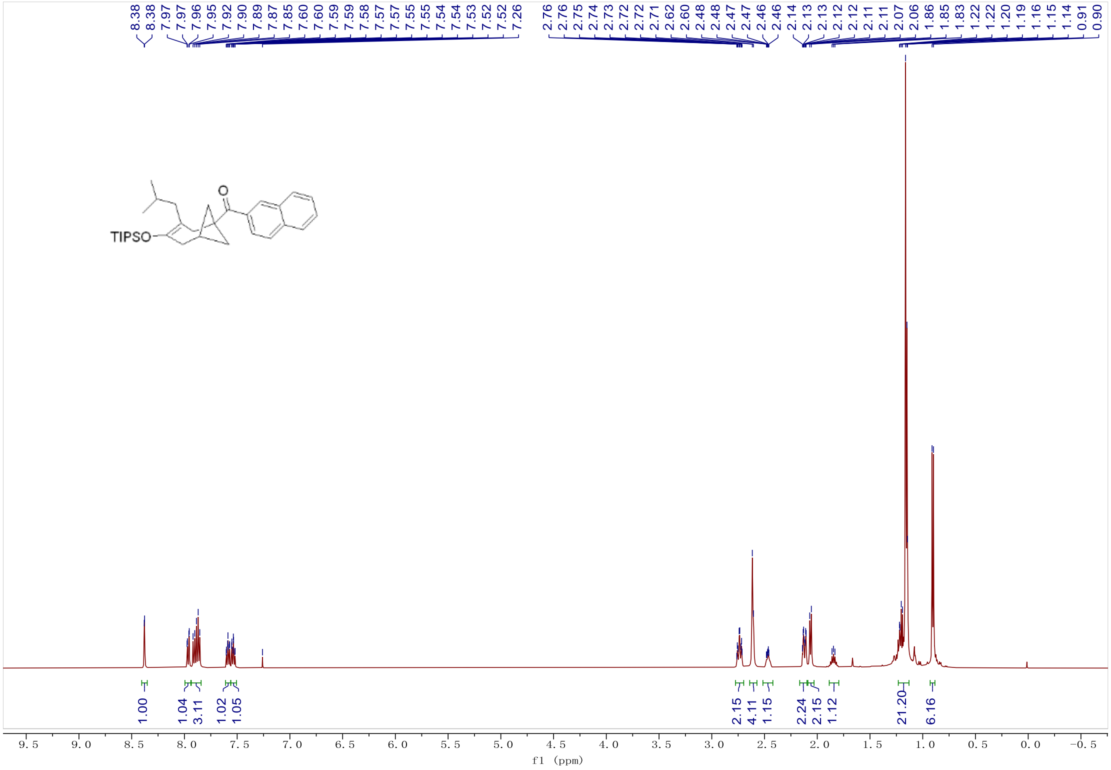

Supplementary Figure 183. **^{1}H NMR** spectrum (500 MHz, $CDCl_3$) of **5f**

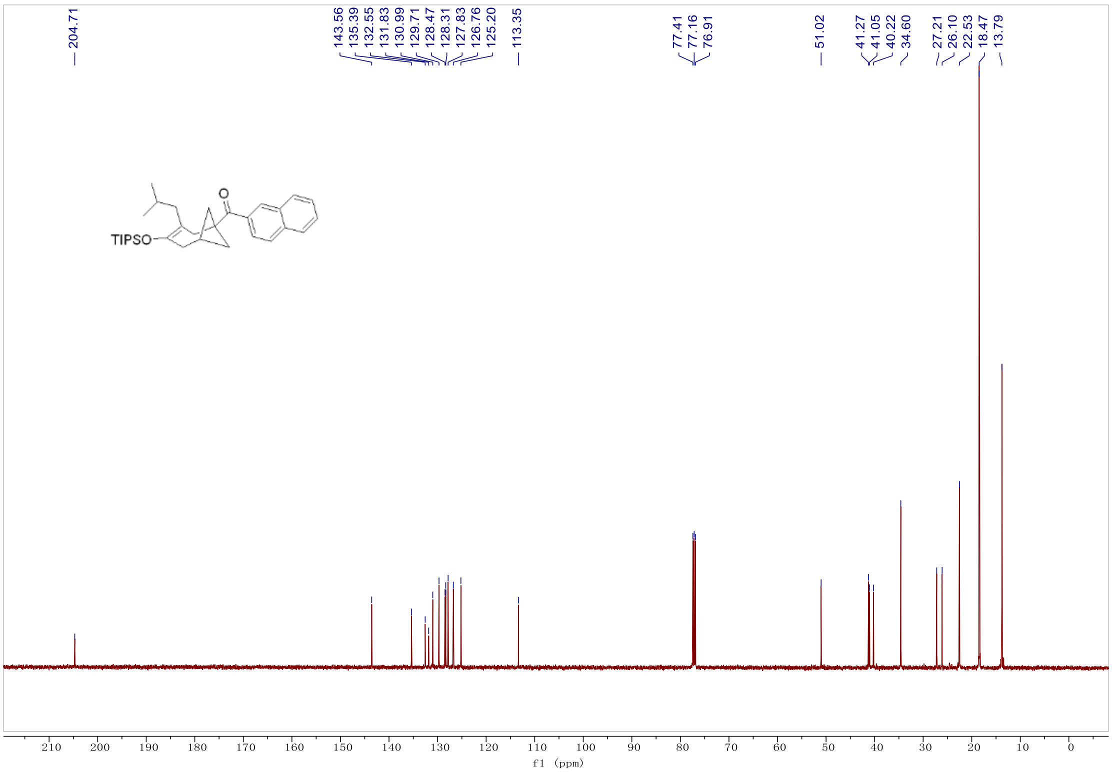

Supplementary Figure 184. **^{13}C NMR** spectrum (125 MHz, $CDCl_3$) of **5f**

Compound 5g

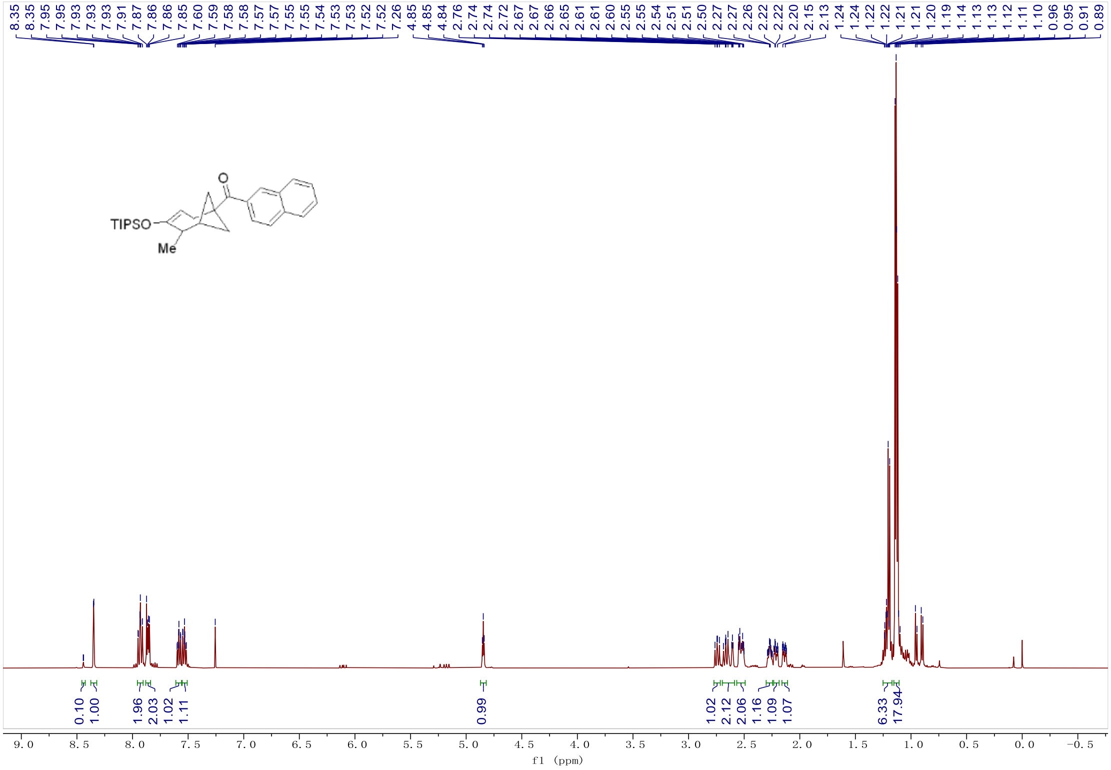

Supplementary Figure 185. ^{1}**H NMR** spectrum (500 MHz, $CDCl_3$) of **5g**

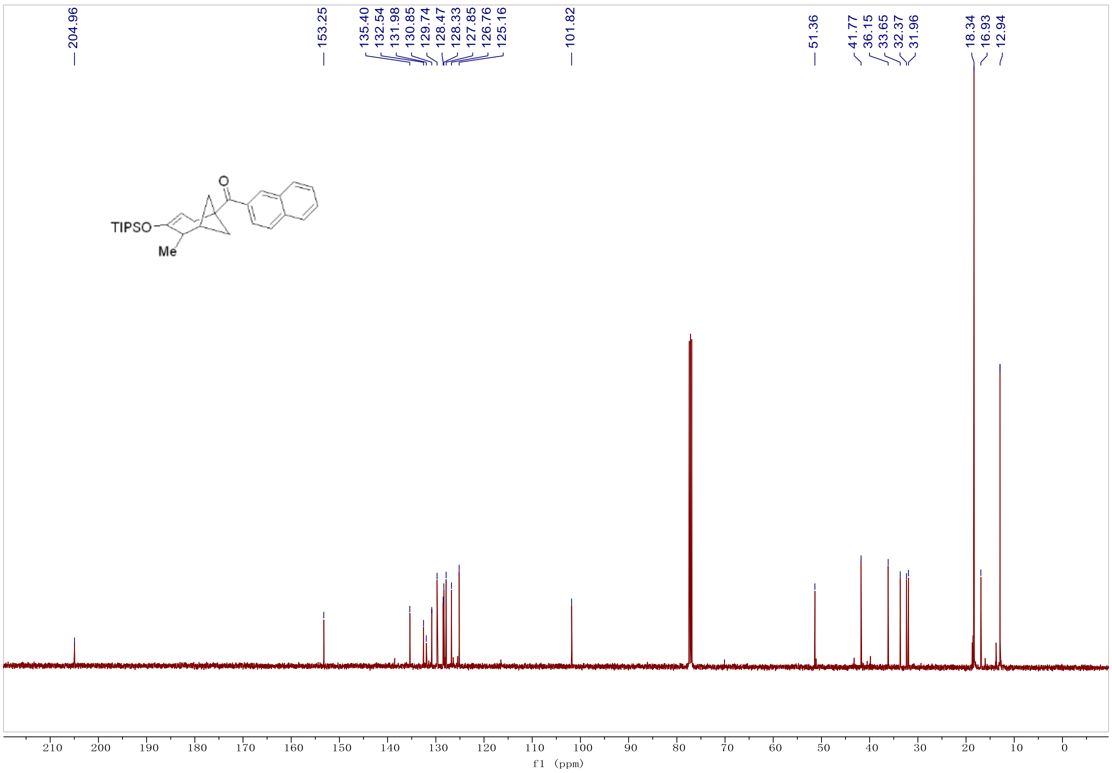

Supplementary Figure 186. ^{13}**C NMR** spectrum (125 MHz, $CDCl_3$) of **5g**

Compound 5h

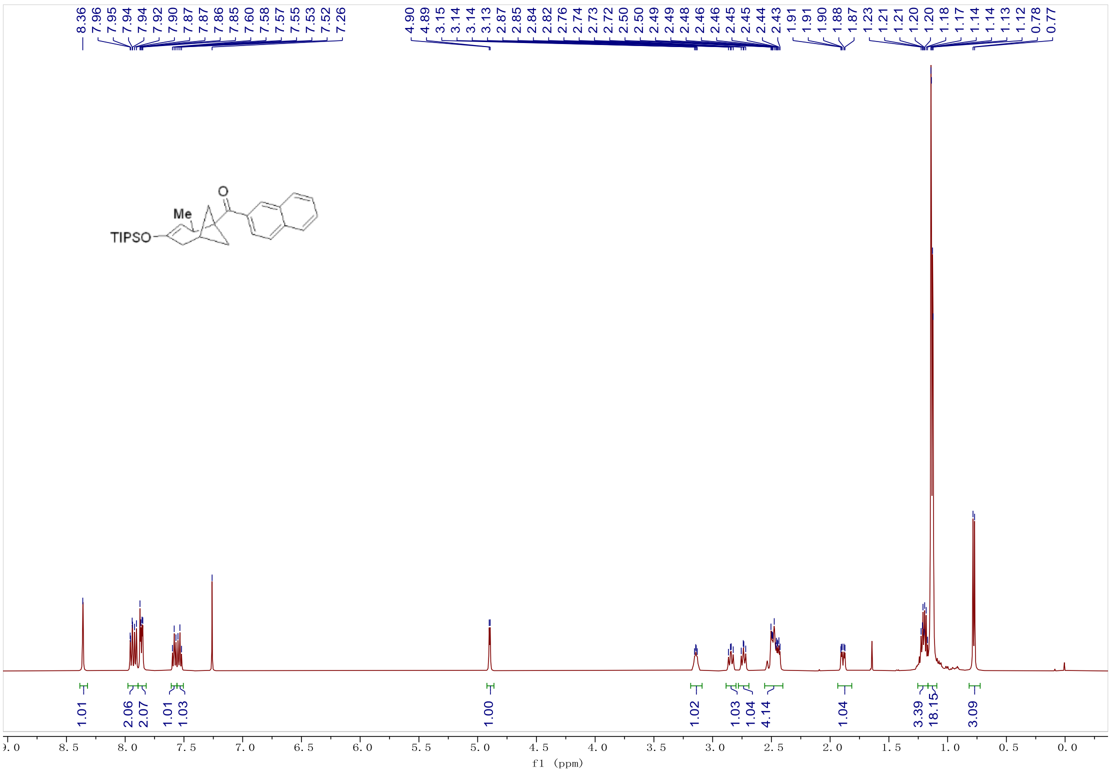

Supplementary Figure 187. ^{1}**H NMR** spectrum (500 MHz, $CDCl_3$) of **5h**

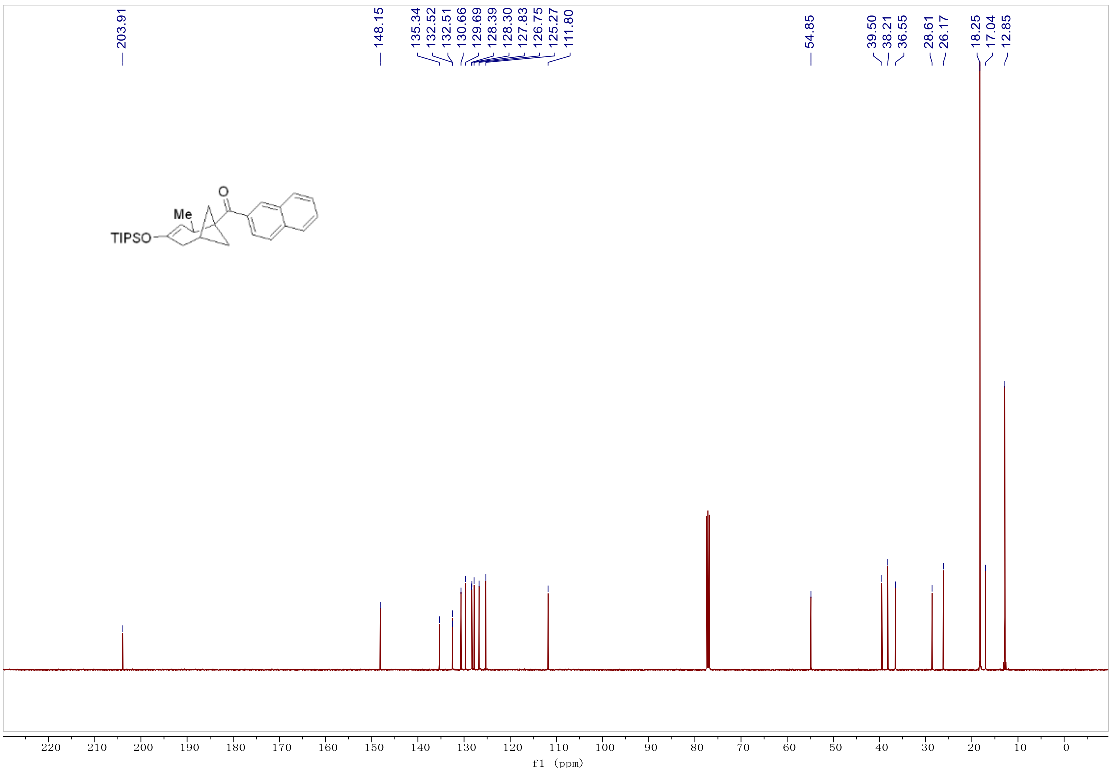

Supplementary Figure 188. ^{13}**C NMR** spectrum (125 MHz, $CDCl_3$) of **5h**

Compound 5i

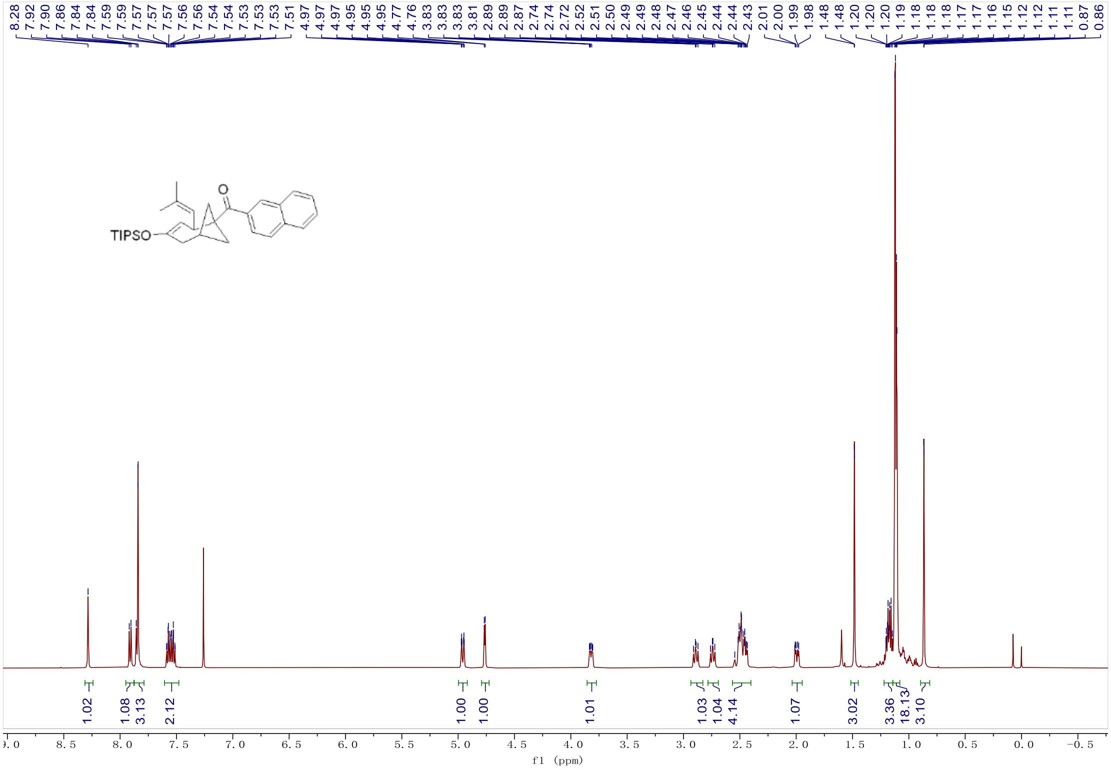

Supplementary Figure 189. ^{1}**H NMR** spectrum (500 MHz, $CDCl_3$) of **5i**

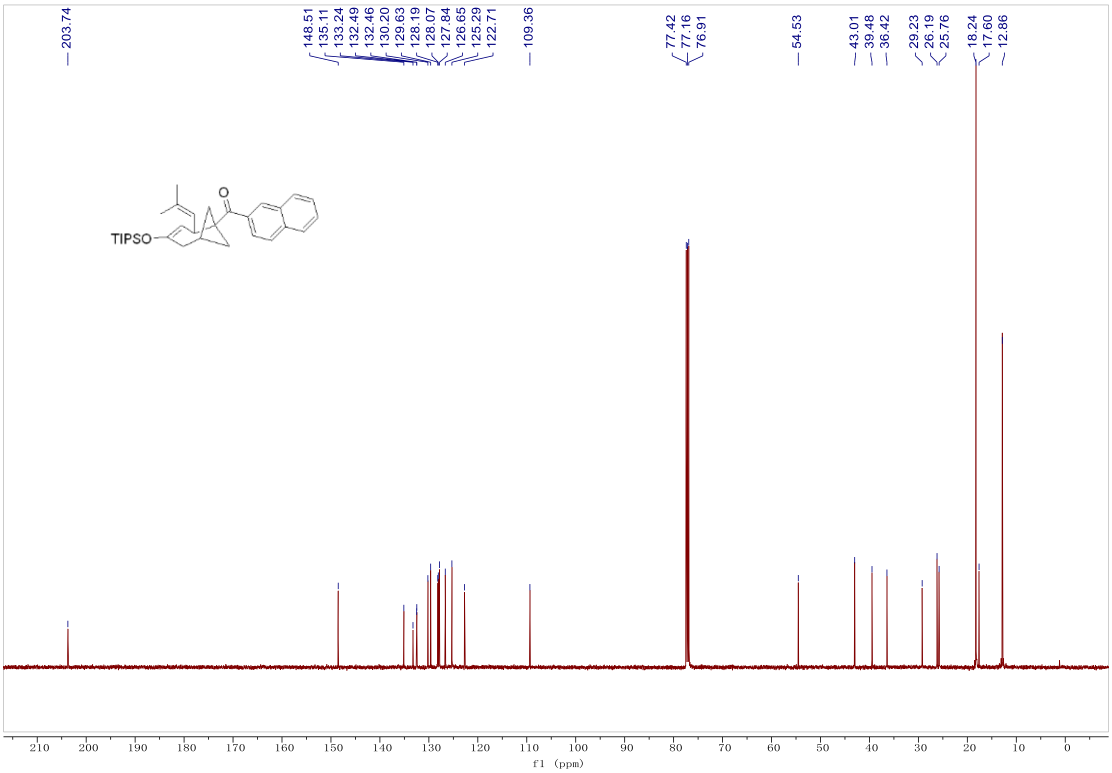

Supplementary Figure 190. ^{13}**C NMR** spectrum (125 MHz, $CDCl_3$) of **5i**

Compound 5j

^{1}H NMR (500 MHz, $CDCl_3$)

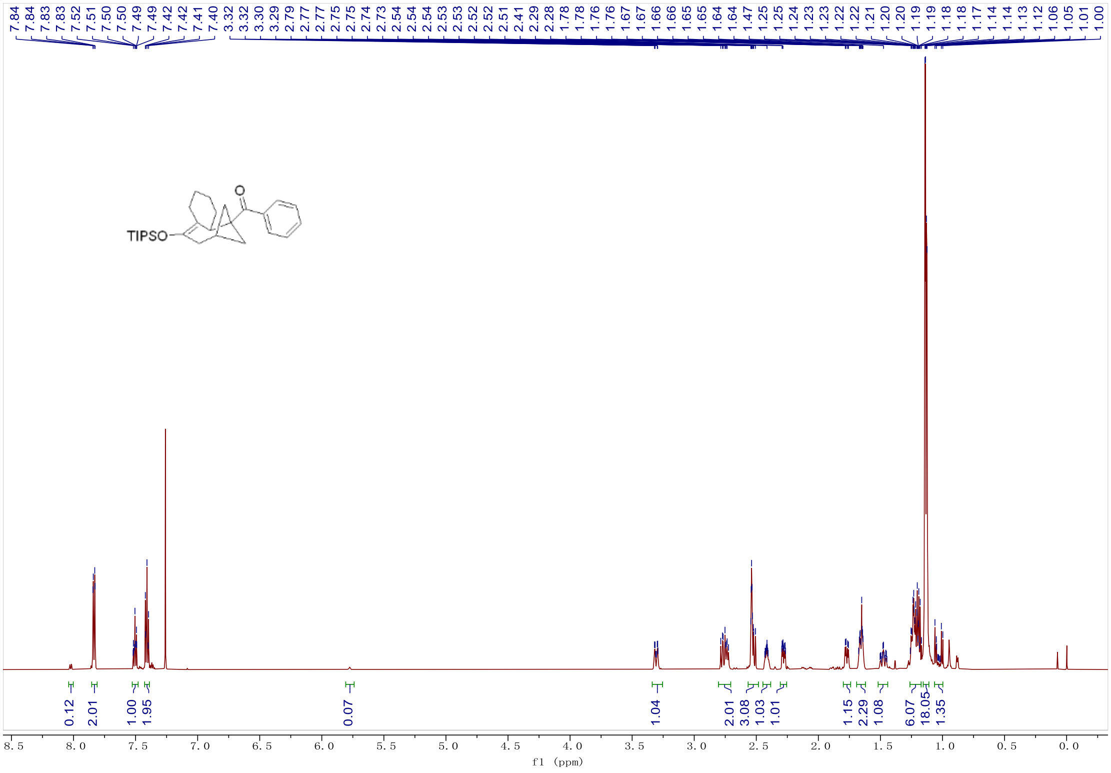

Supplementary Figure 191. ^{1}H NMR spectrum (500 MHz, $CDCl_3$) of **5j**

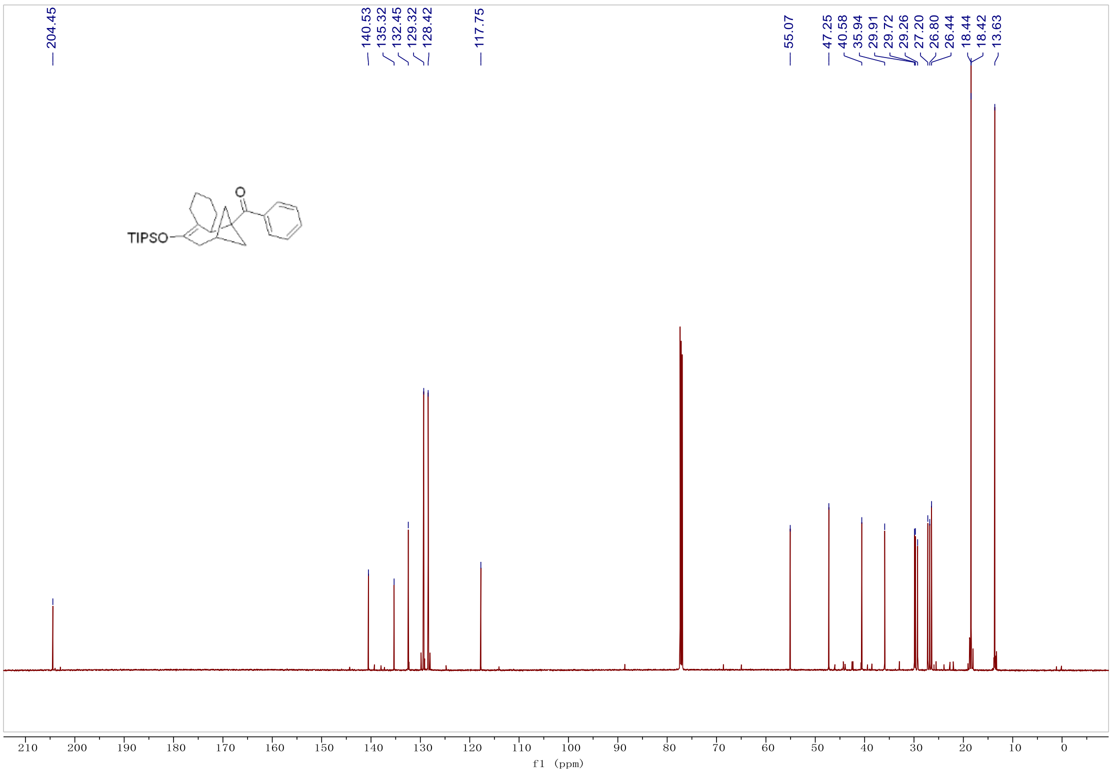

Supplementary Figure 192. ^{13}C NMR spectrum (125 MHz, $CDCl_3$) of **5j**

Compound 5k

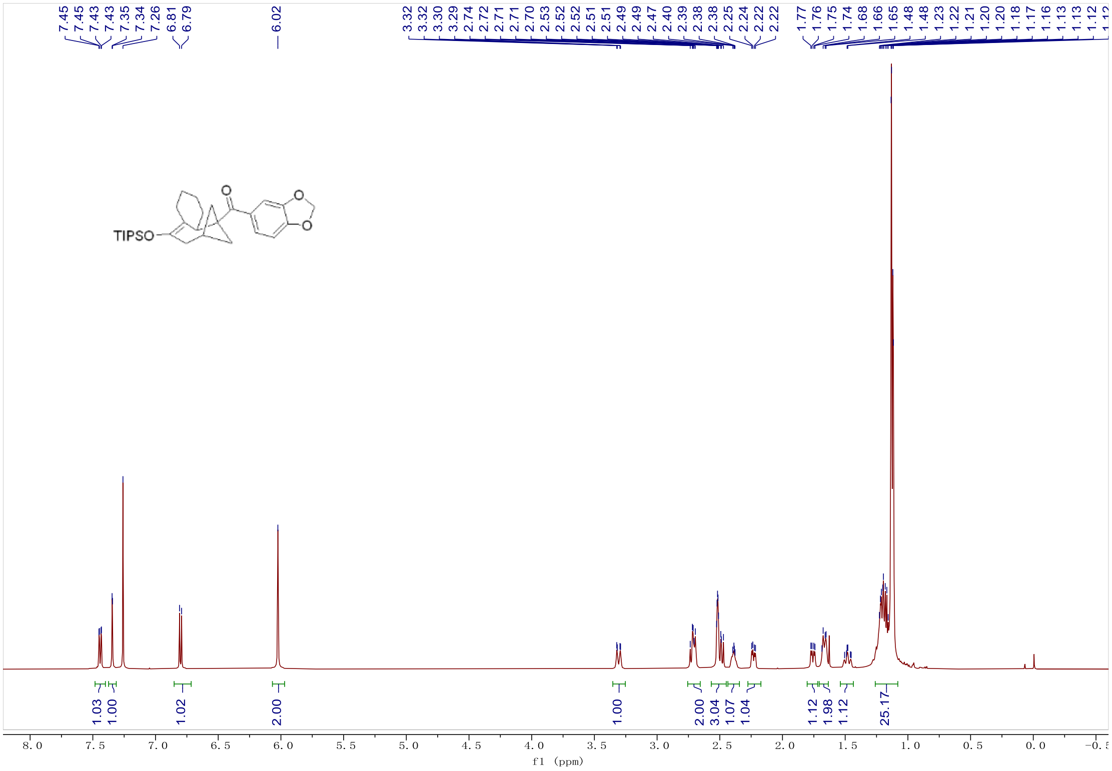

Supplementary Figure 193. ^{1}H NMR spectrum (500 MHz, $CDCl_3$) of **5k**

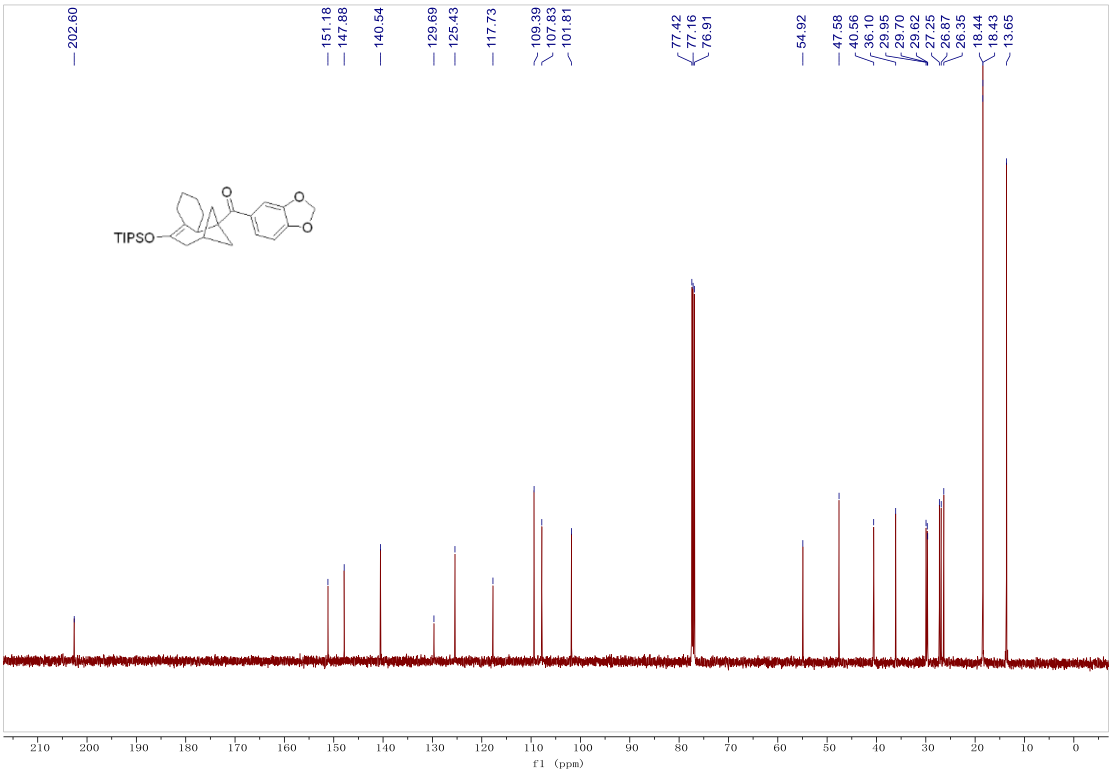

Supplementary Figure 194. ^{13}C NMR spectrum (125 MHz, $CDCl_3$) of **5k**

Compound 5l

^{1}H NMR (500 MHz, $CDCl_3$)

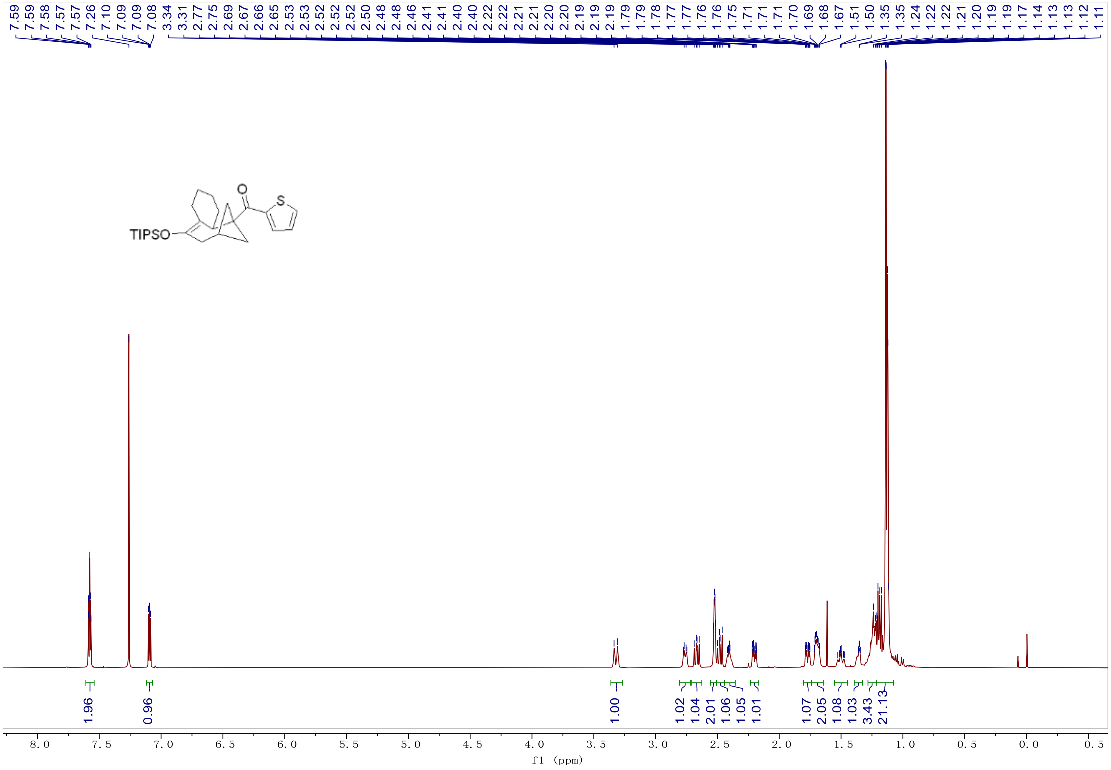

Supplementary Figure 195. ^{1}H NMR spectrum (500 MHz, $CDCl_3$) of **5l**

^{13}C NMR (125 MHz, $CDCl_3$)

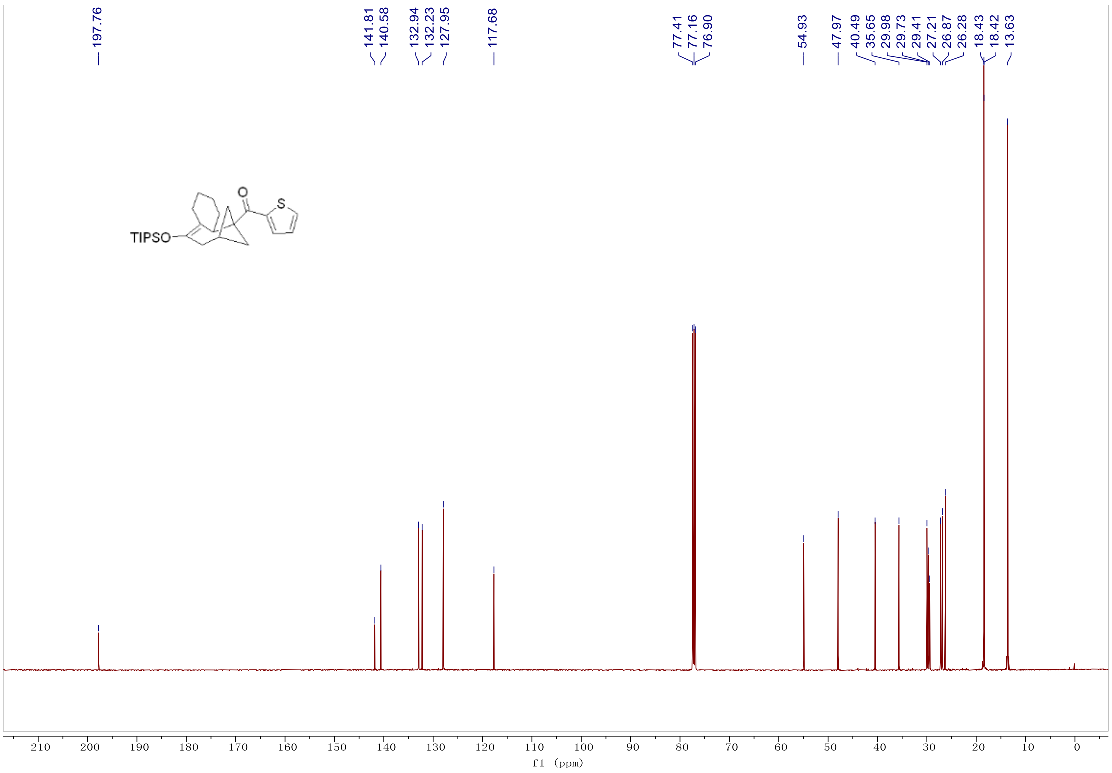

Supplementary Figure 196. ^{13}C NMR spectrum (125 MHz, $CDCl_3$) of **5l**

Compound 5m

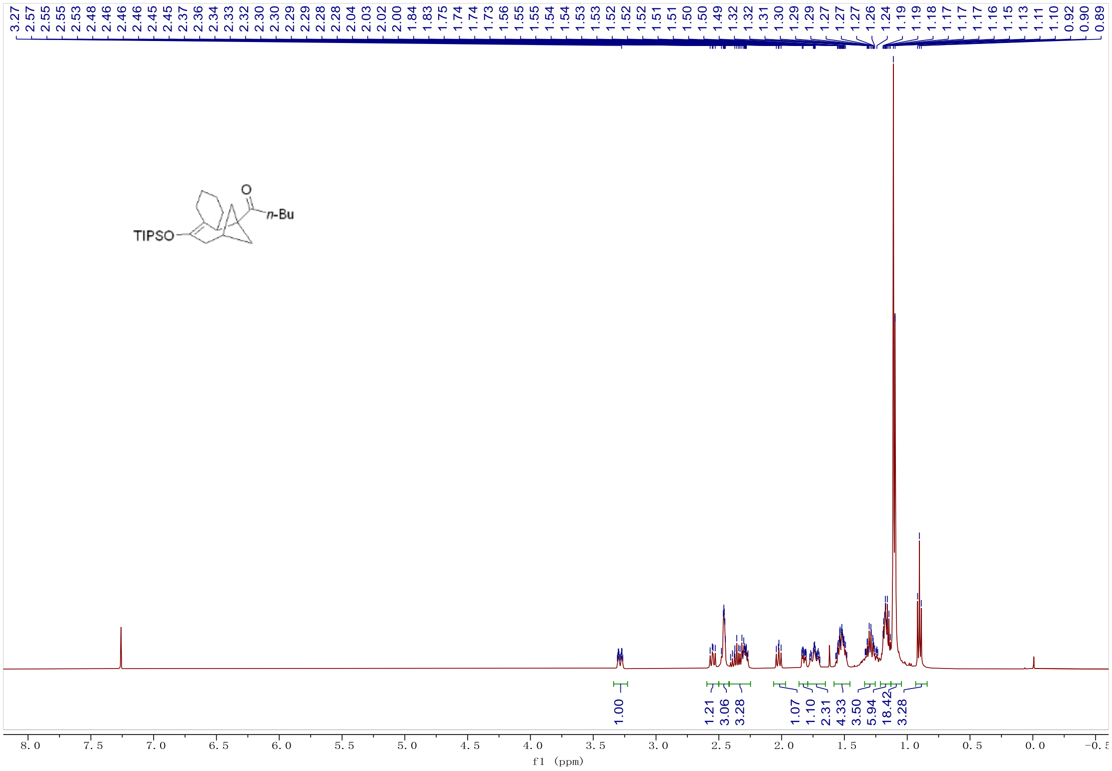

Supplementary Figure 197. ¹H NMR spectrum (500 MHz, $CDCl_3$) of **5m**

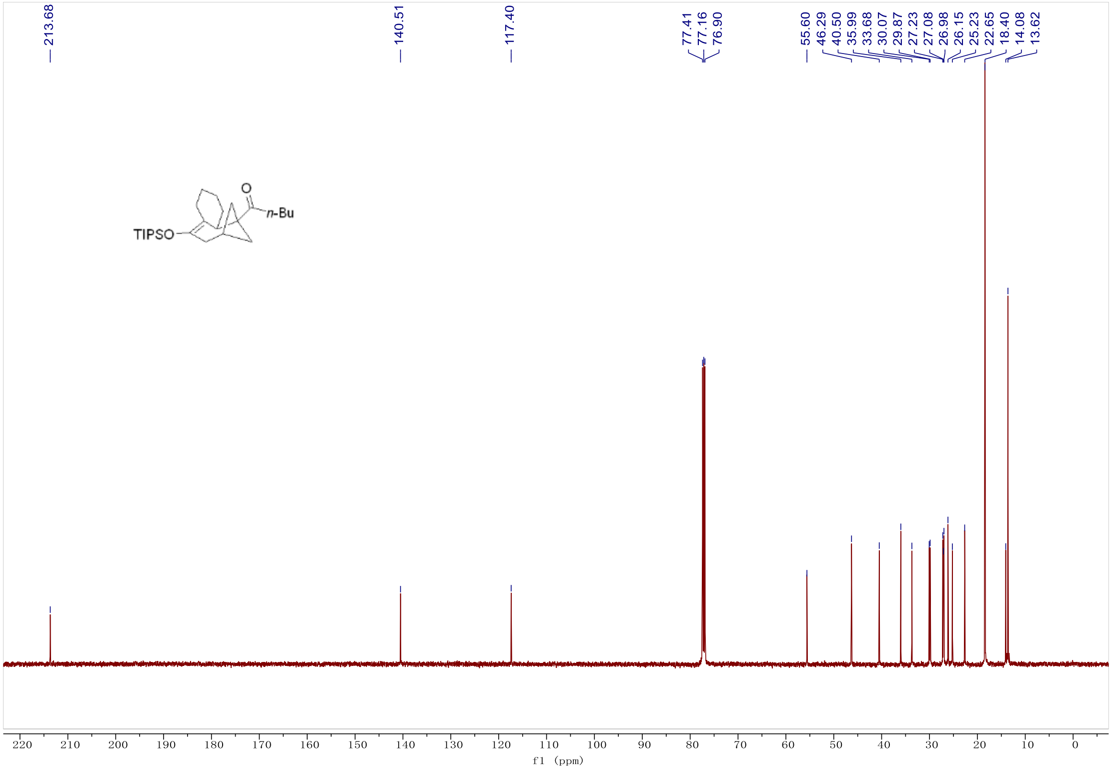

Supplementary Figure 198. ¹³C NMR spectrum (125 MHz, $CDCl_3$) of **5m**

Compound S12

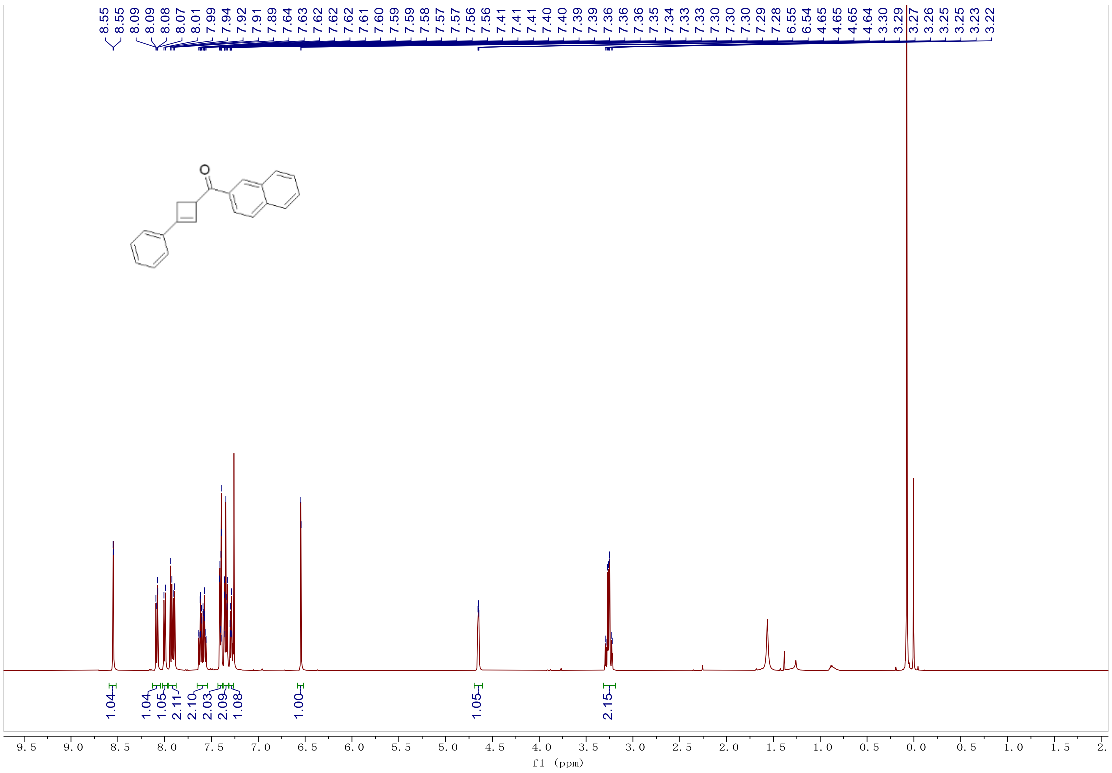

Supplementary Figure 199. ^{1}H NMR spectrum (500 MHz, $CDCl_3$) of **S12**

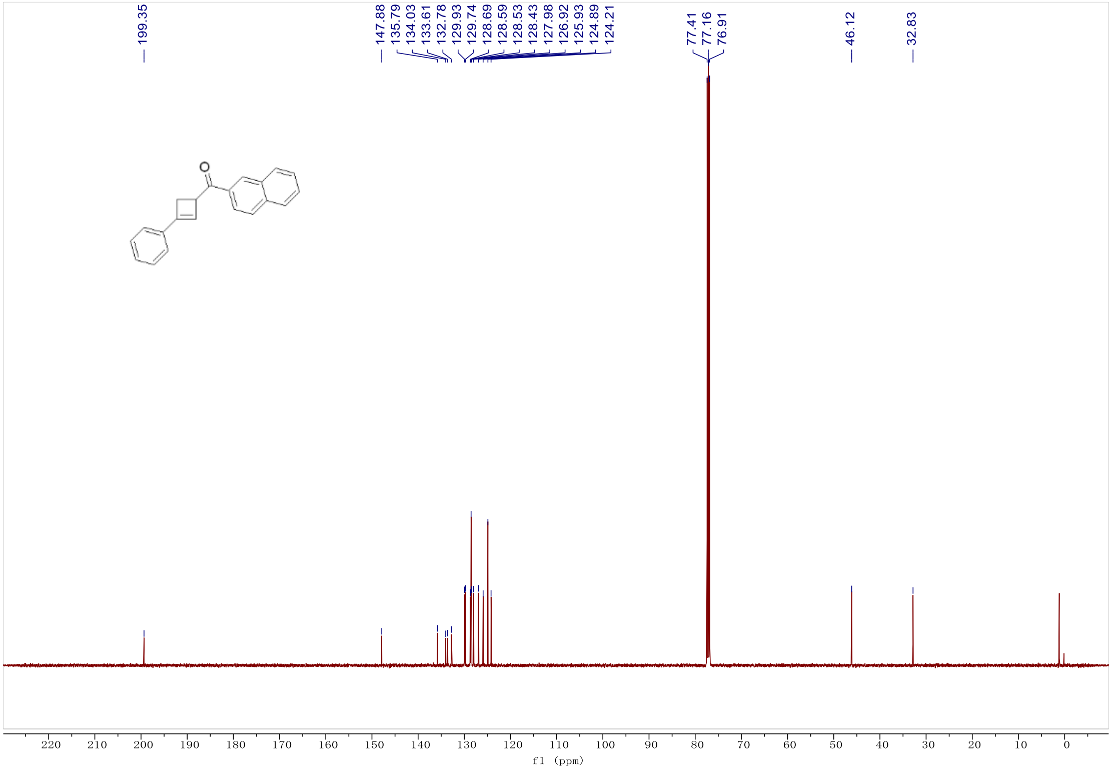

Supplementary Figure 200. ^{13}C NMR spectrum (125 MHz, $CDCl_3$) of **S12**

Compound 6

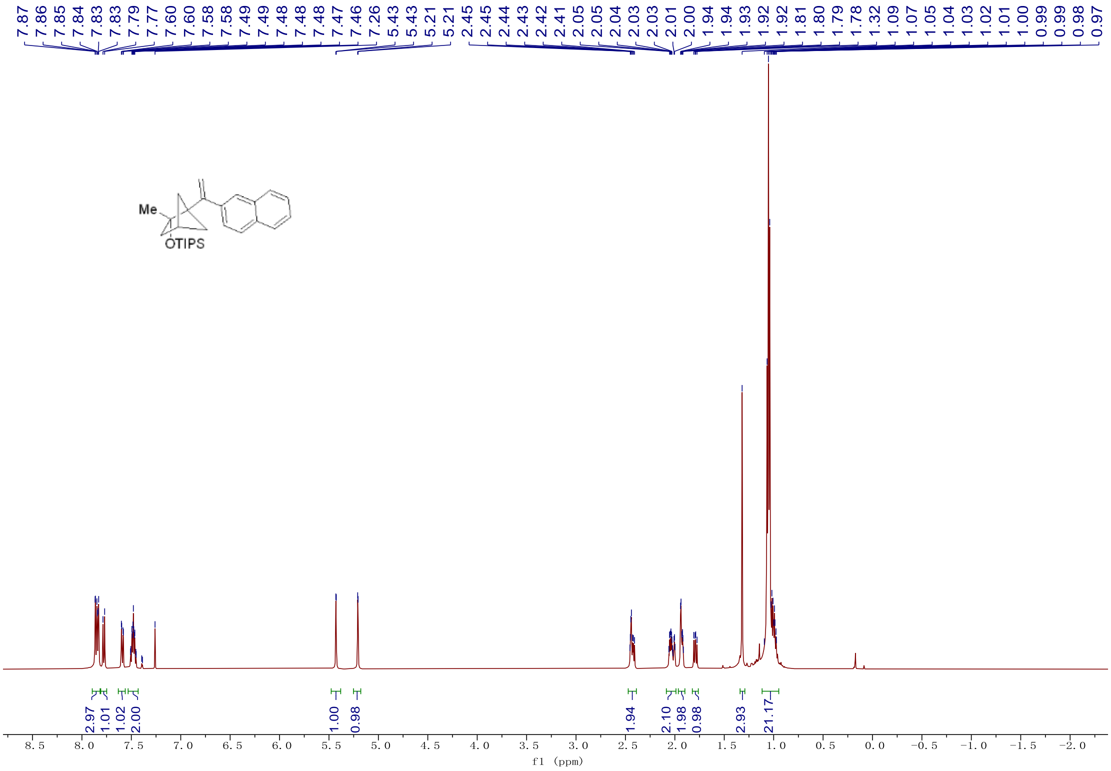

Supplementary Figure 201. ^{1}H NMR spectrum (500 MHz, $CDCl_3$) of **6**

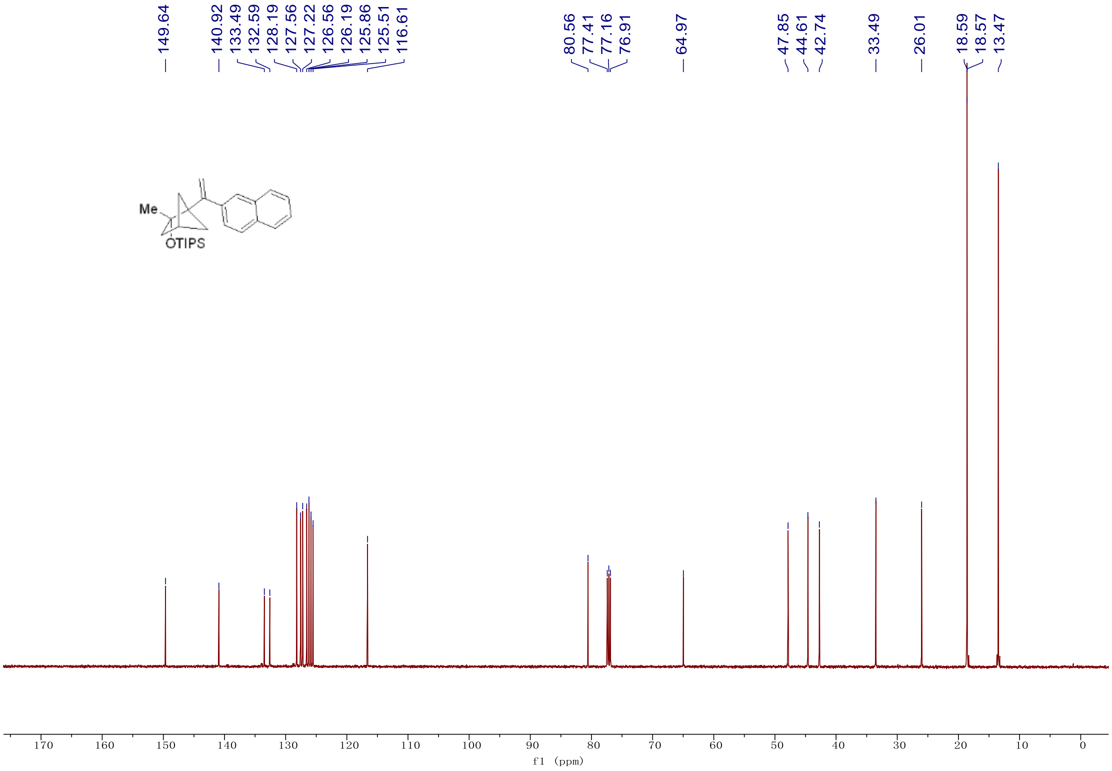

Supplementary Figure 202. ^{13}C NMR spectrum (125 MHz, $CDCl_3$) of **6**

Compound 7

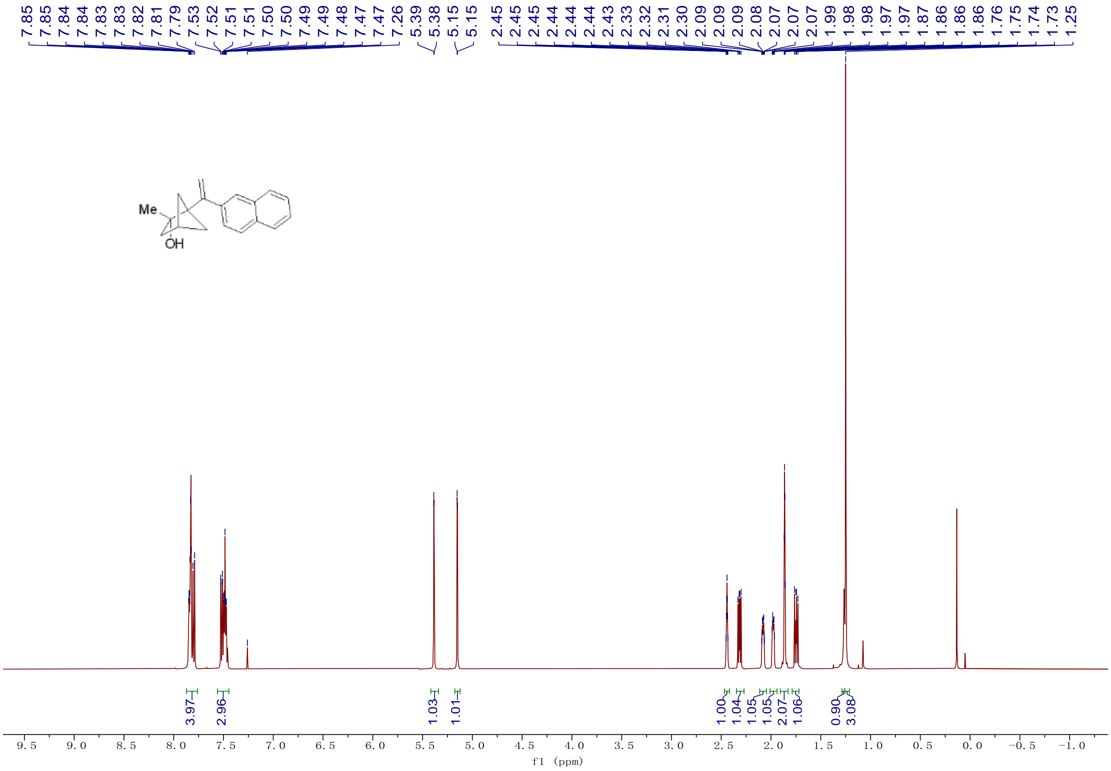

Supplementary Figure 203. ^{1}H NMR spectrum (500 MHz, $CDCl_3$) of **7**

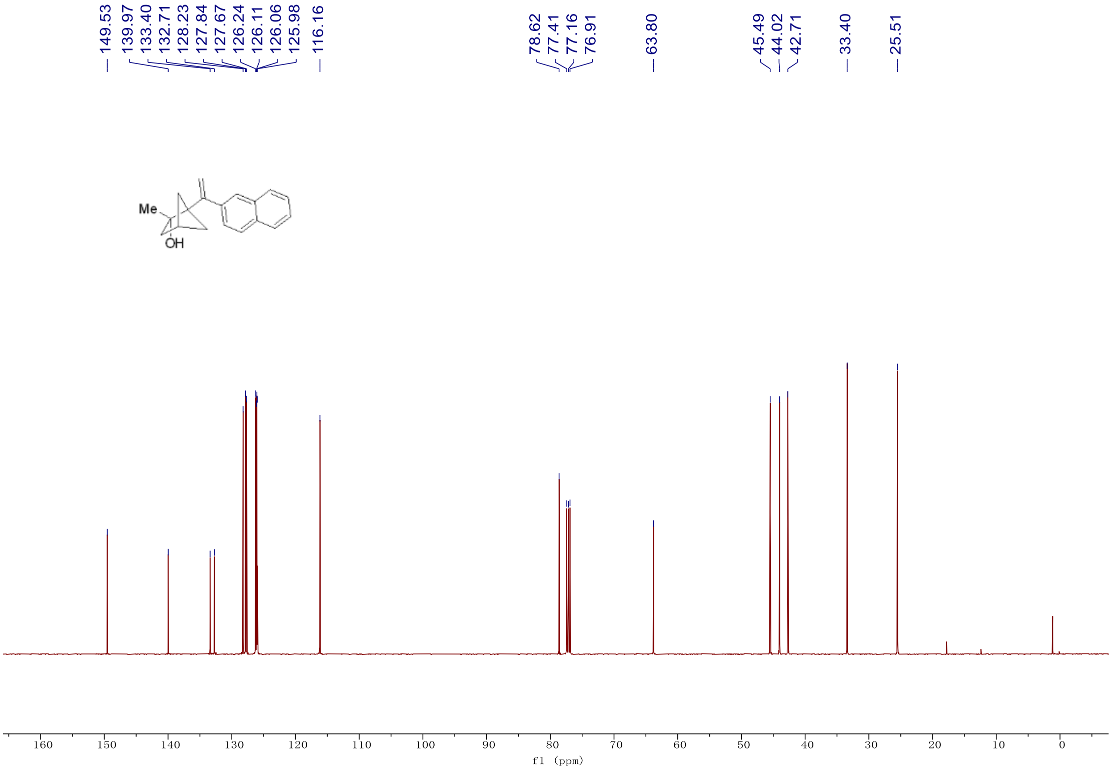

Supplementary Figure 204. ^{13}C NMR spectrum (125 MHz, $CDCl_3$) of **7**

Compound 8

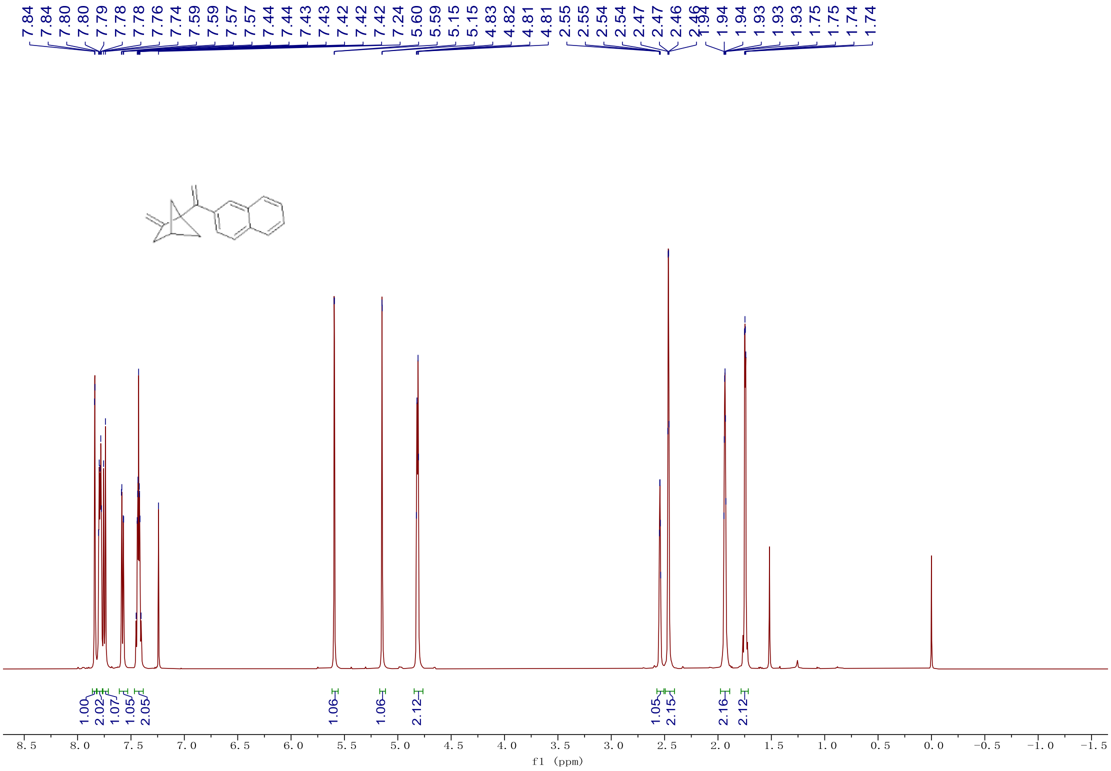

Supplementary Figure 205. ^{1}H NMR spectrum (500 MHz, $CDCl_3$) of **8**

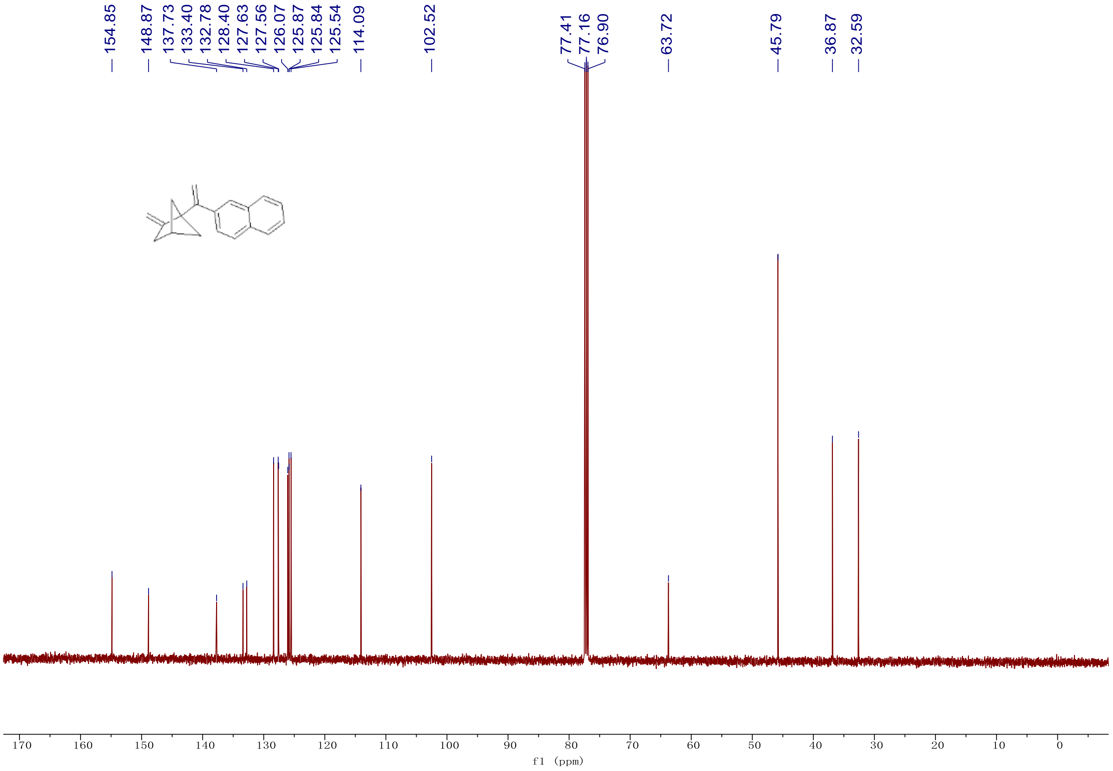

Supplementary Figure 206. ^{13}C NMR spectrum (125 MHz, $CDCl_3$) of **8**

Compound 9

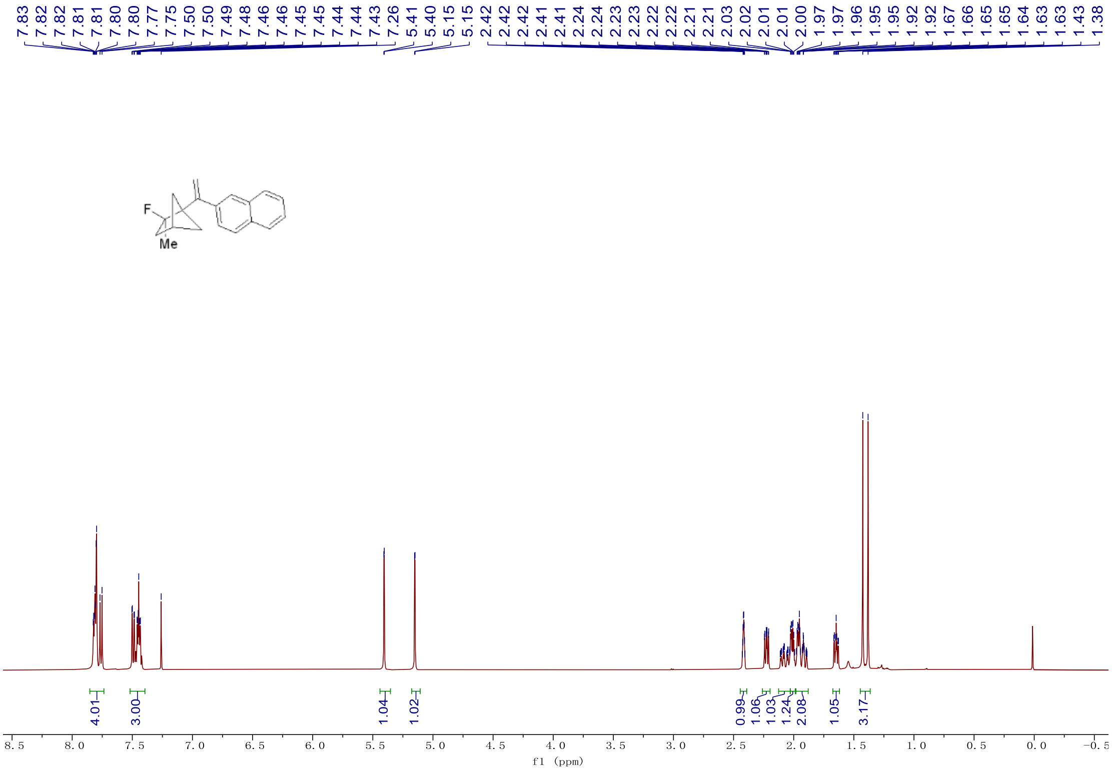

Supplementary Figure 207. ^{1}H NMR spectrum (500 MHz, $CDCl_3$) of **9**

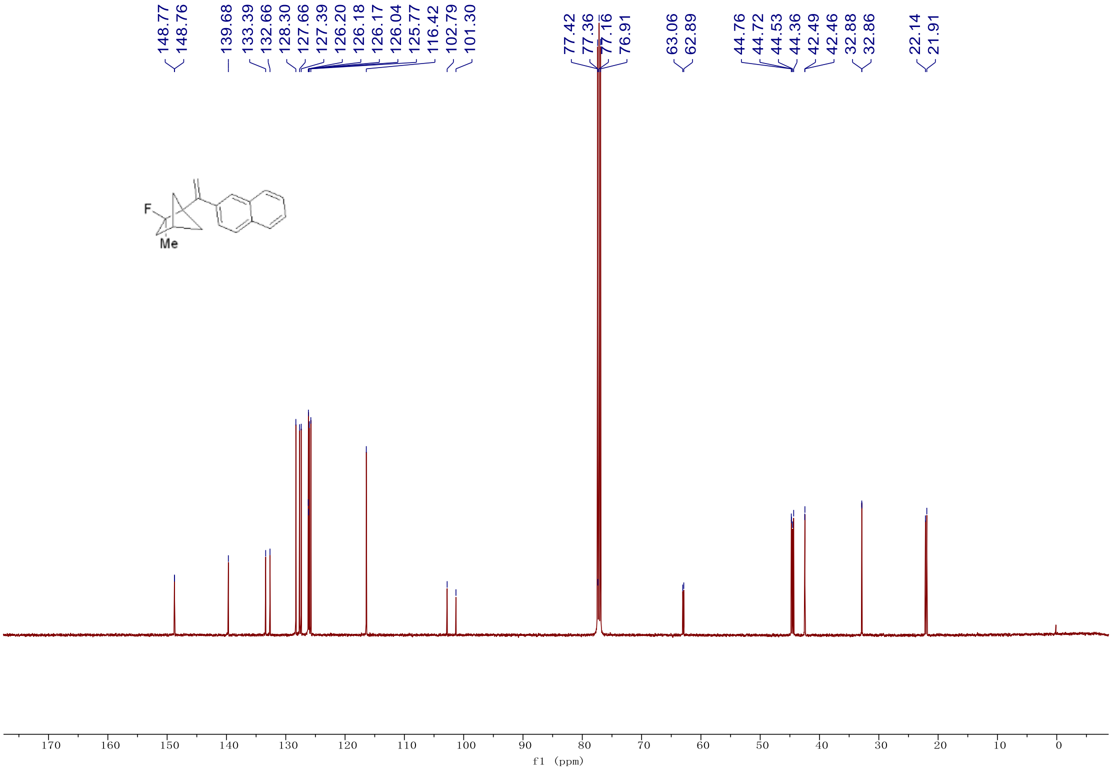

Supplementary Figure 208. ^{13}C NMR spectrum (125 MHz, $CDCl_3$) of **9**

Compound 9

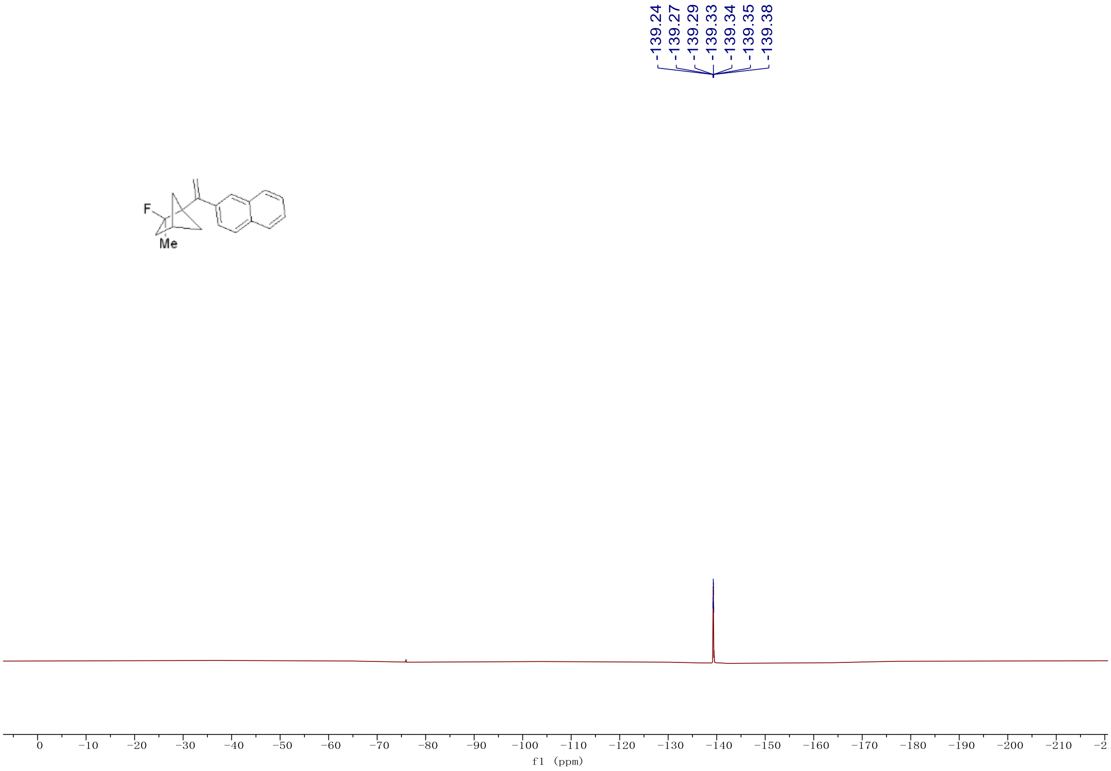

Supplementary Figure 209. ^{19}F NMR spectrum (471 MHz, $CDCl_3$) of **9**

Compound 10

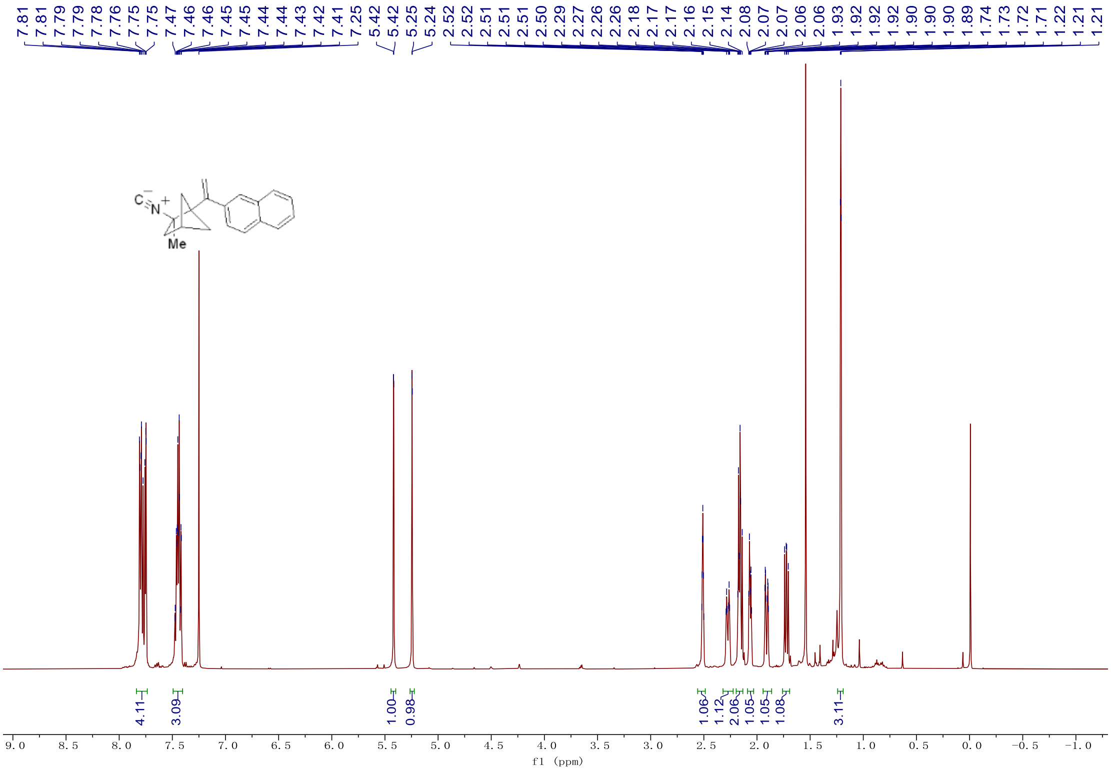

Supplementary Figure 210. ^{1}**H NMR** spectrum (500 MHz, $CDCl_3$) of **10**

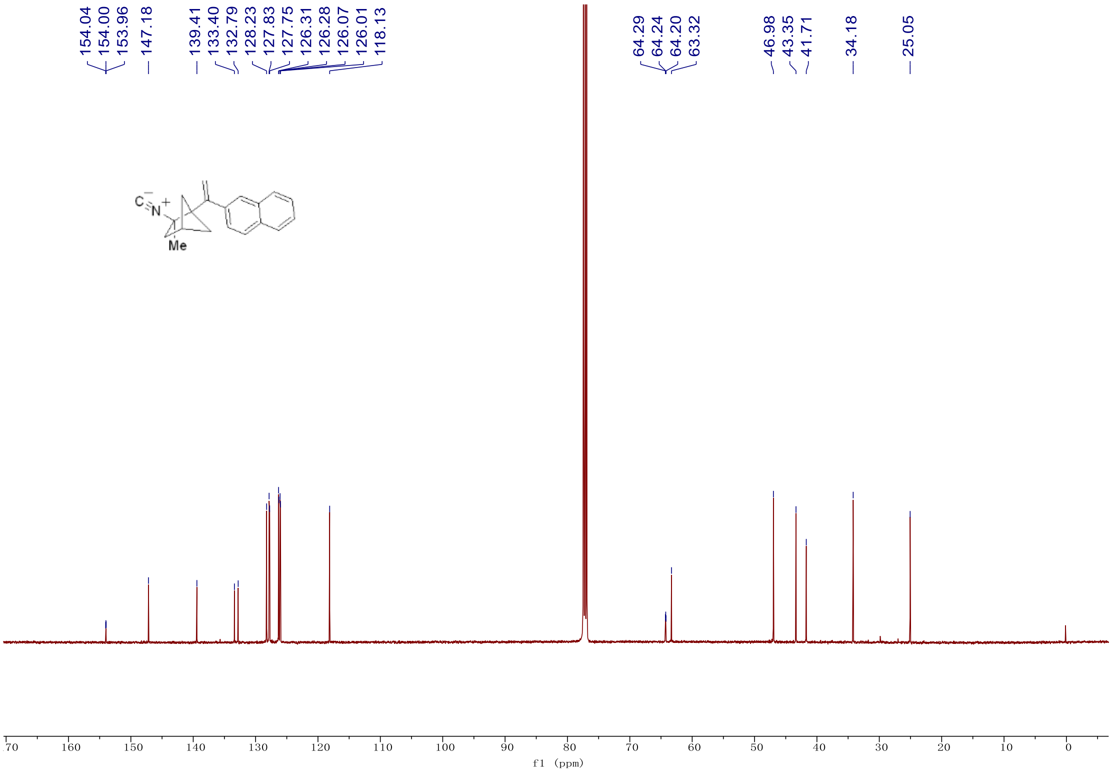

Supplementary Figure 211. ^{13}**C NMR** spectrum (125 MHz, $CDCl_3$) of **10**

Compound 11

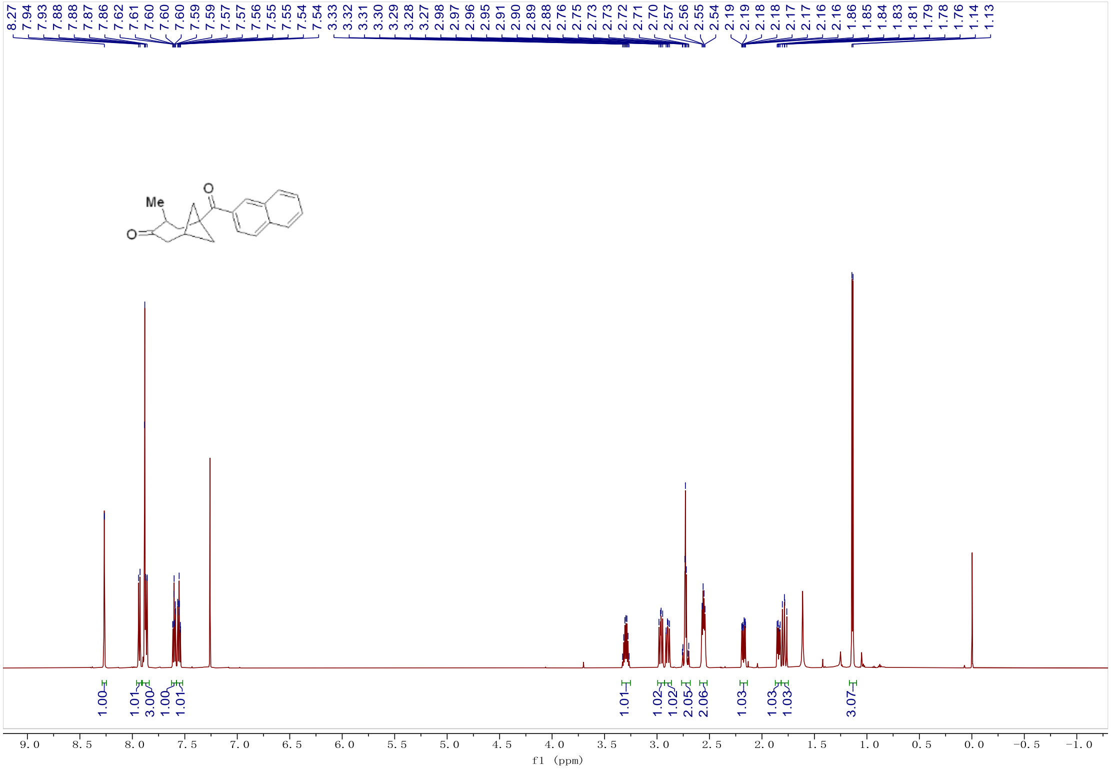

Supplementary Figure 212. ^{1}**H NMR** spectrum (600 MHz, $CDCl_3$) of **11**

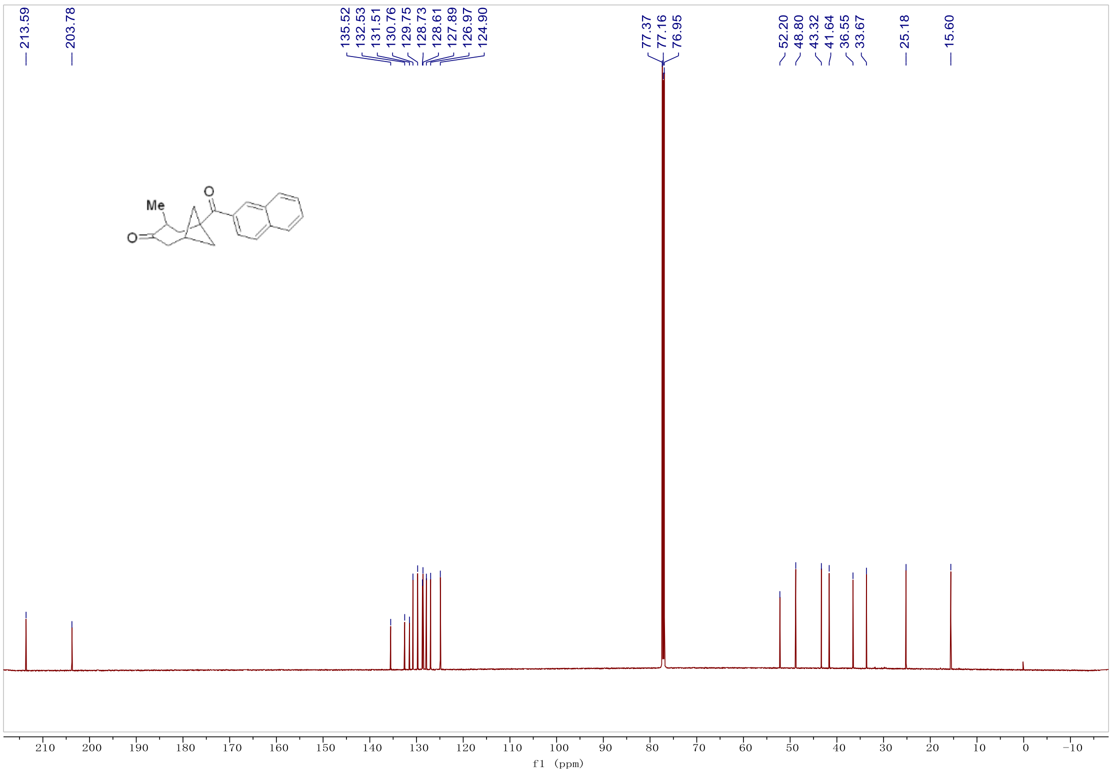

Supplementary Figure 213. ^{13}**C NMR** spectrum (150 MHz, $CDCl_3$) of **11**

Compound 12

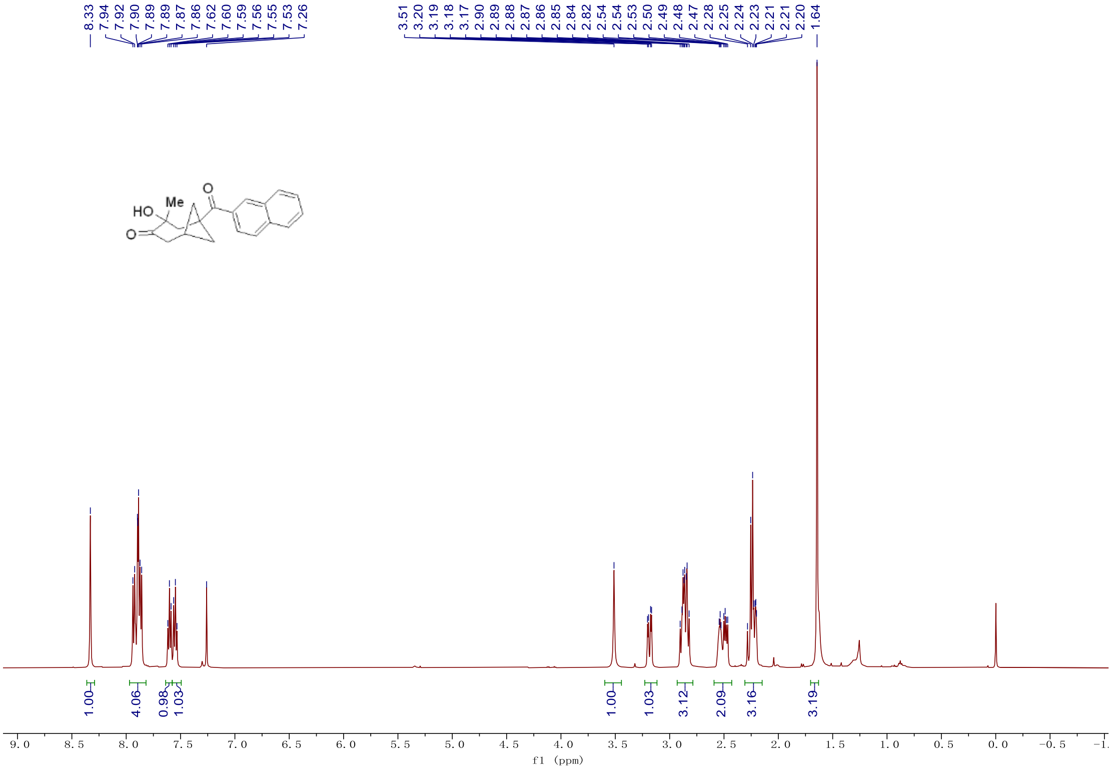

Supplementary Figure 214. ^{1}H NMR spectrum (500 MHz, $CDCl_3$) of **12**

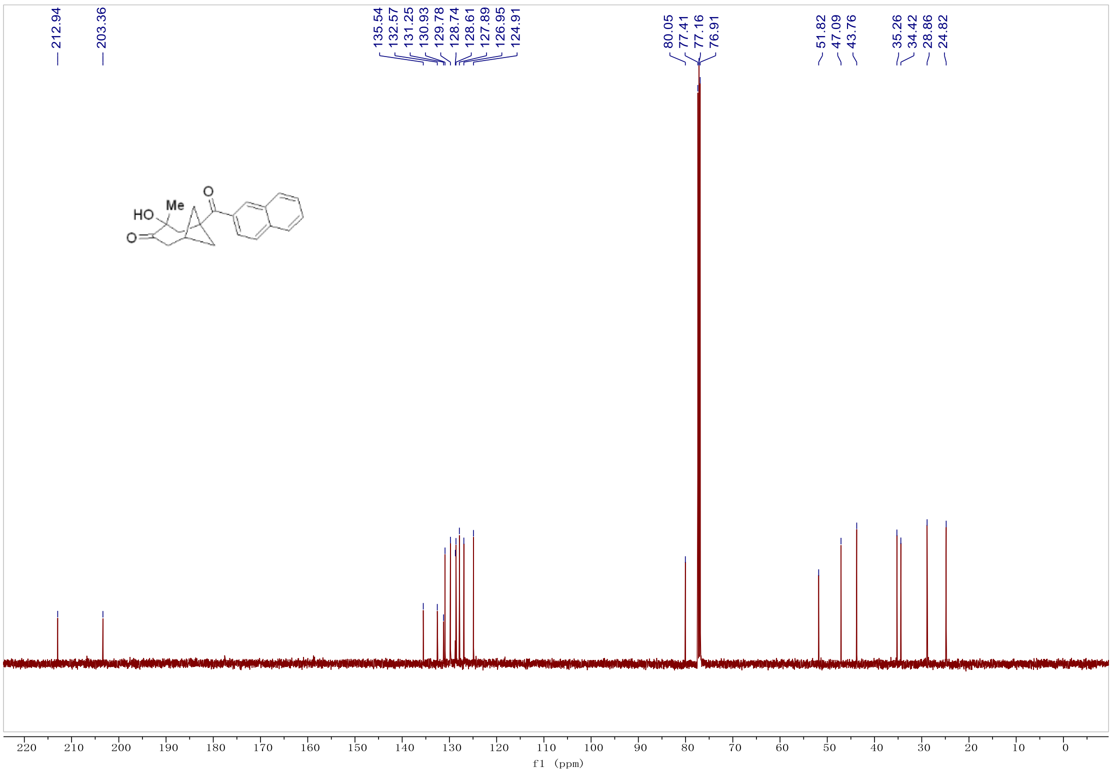

Supplementary Figure 215. ^{13}C NMR spectrum (125 MHz, $CDCl_3$) of **12**

Compound 13

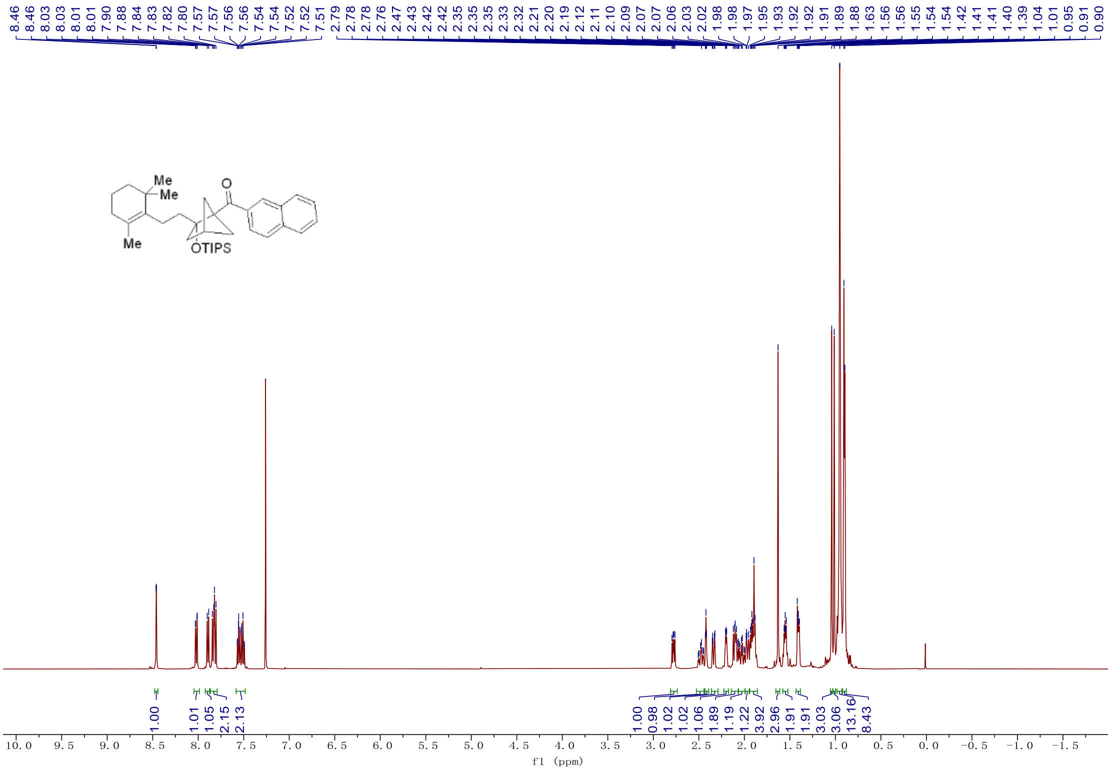

Supplementary Figure 216. ^{1}**H NMR** spectrum (500 MHz, $CDCl_3$) of **13**

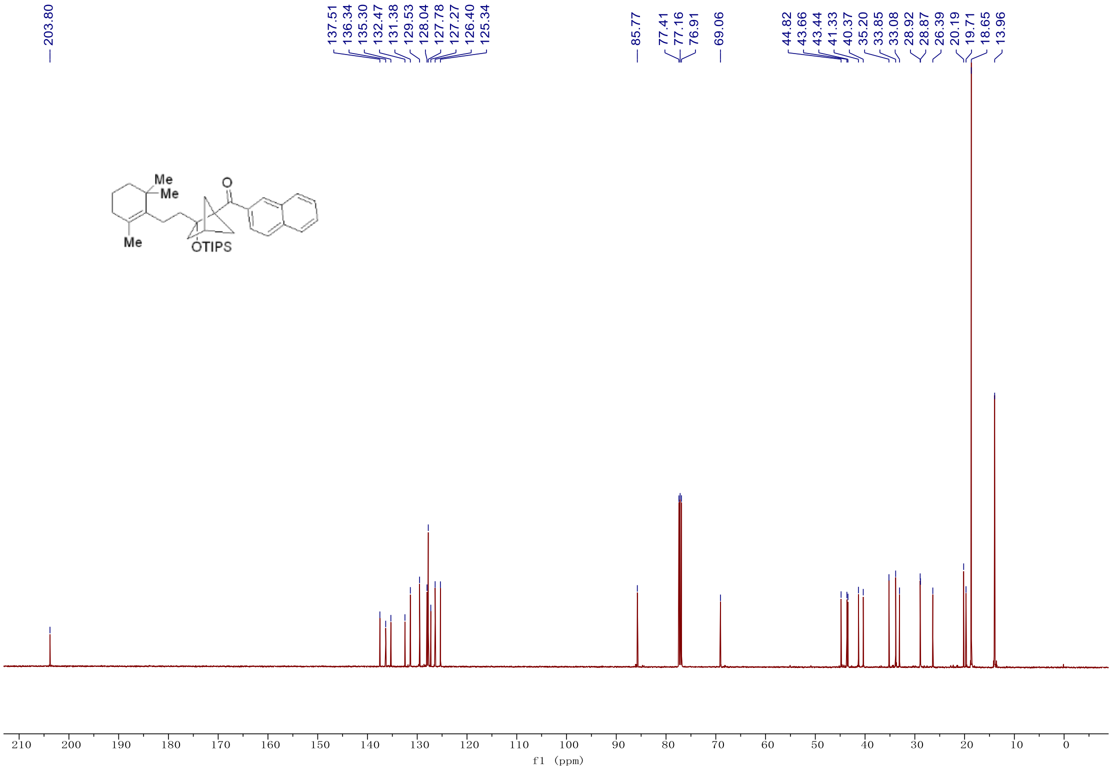

Supplementary Figure 217. ^{13}**C NMR** spectrum (125 MHz, $CDCl_3$) of **13**

Compound 14 (major isomer)

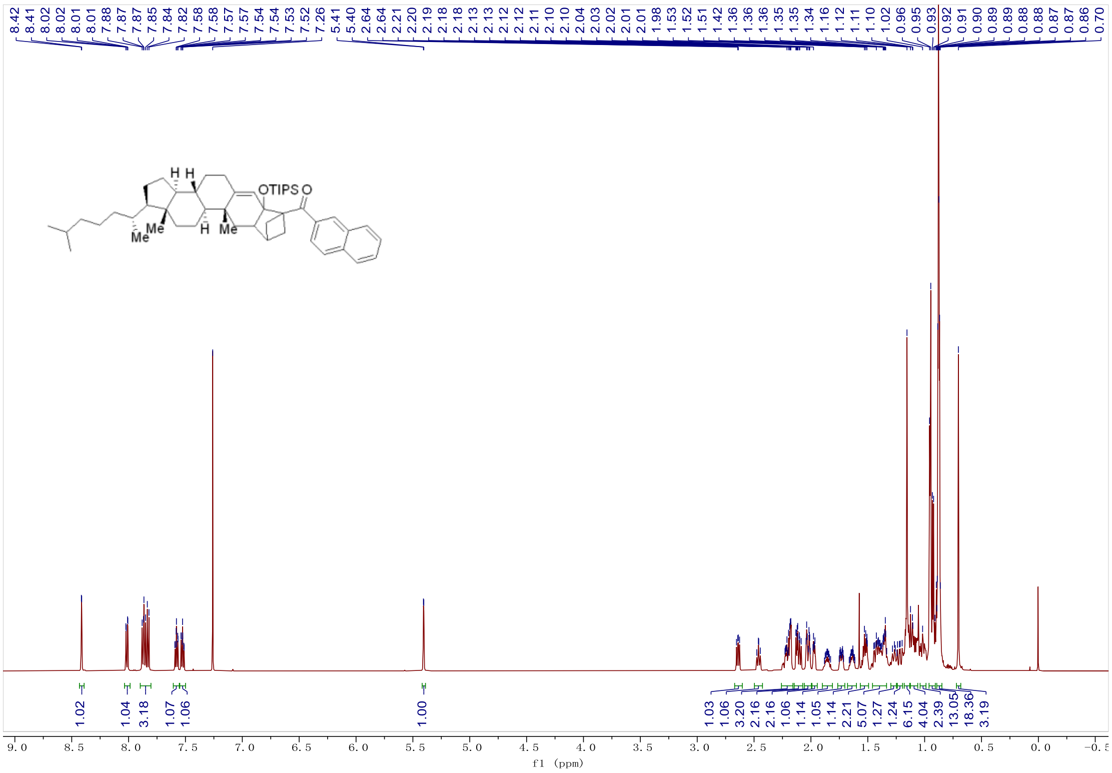

Supplementary Figure 218. ^{1}H NMR spectrum (600 MHz, $CDCl_3$) of **14 (major isomer)**

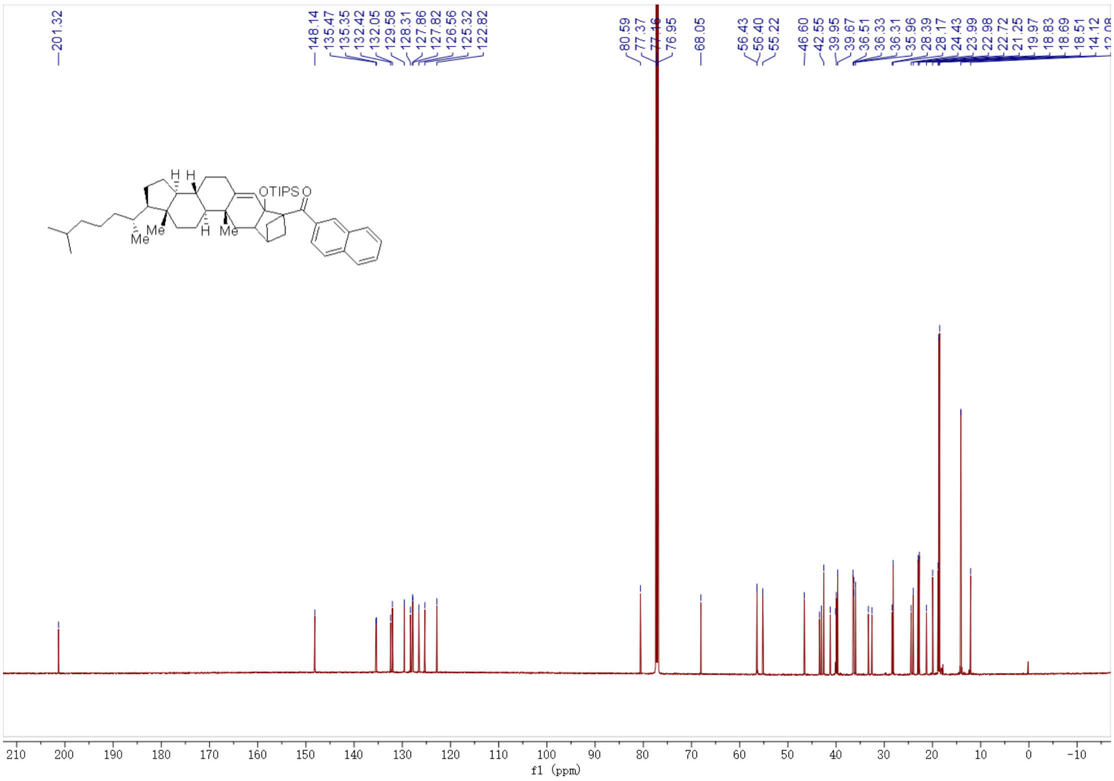

Supplementary Figure 219. ^{13}C NMR spectrum (150 MHz, $CDCl_3$) of **14 (major isomer)**

Compound 14 (minor isomer)

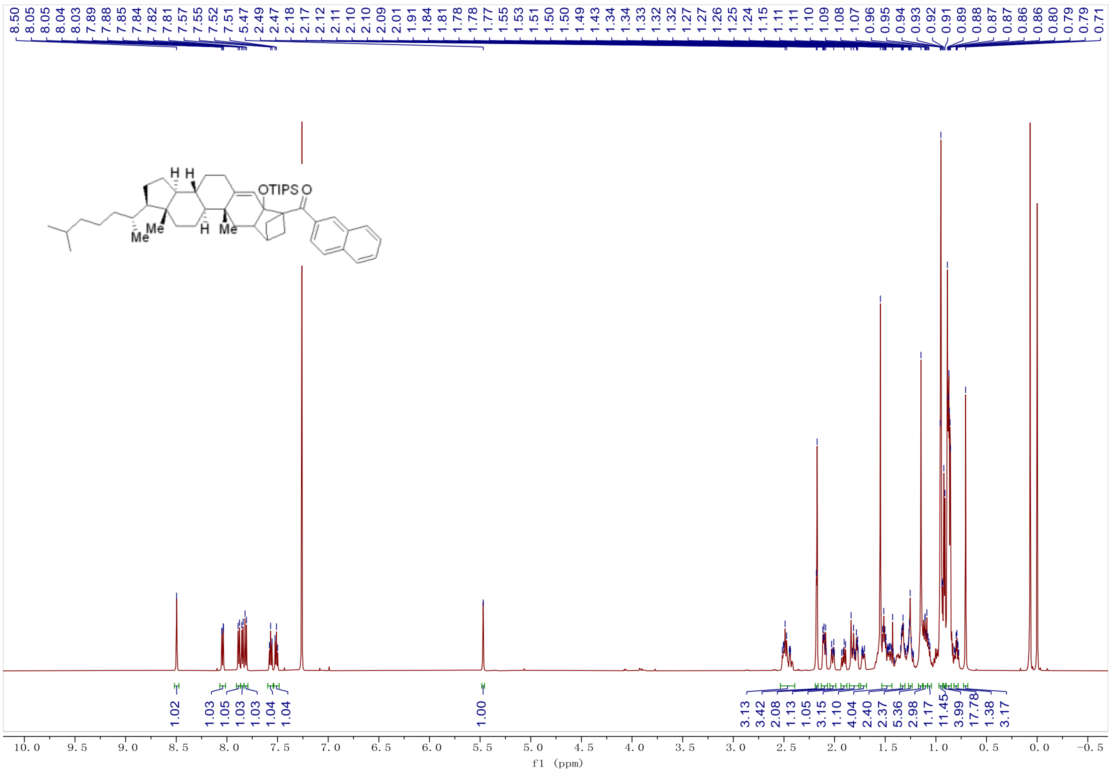

Supplementary Figure 220. ^{1}H NMR spectrum (600 MHz, CDCl$_3$) of **14 (minor isomer)**

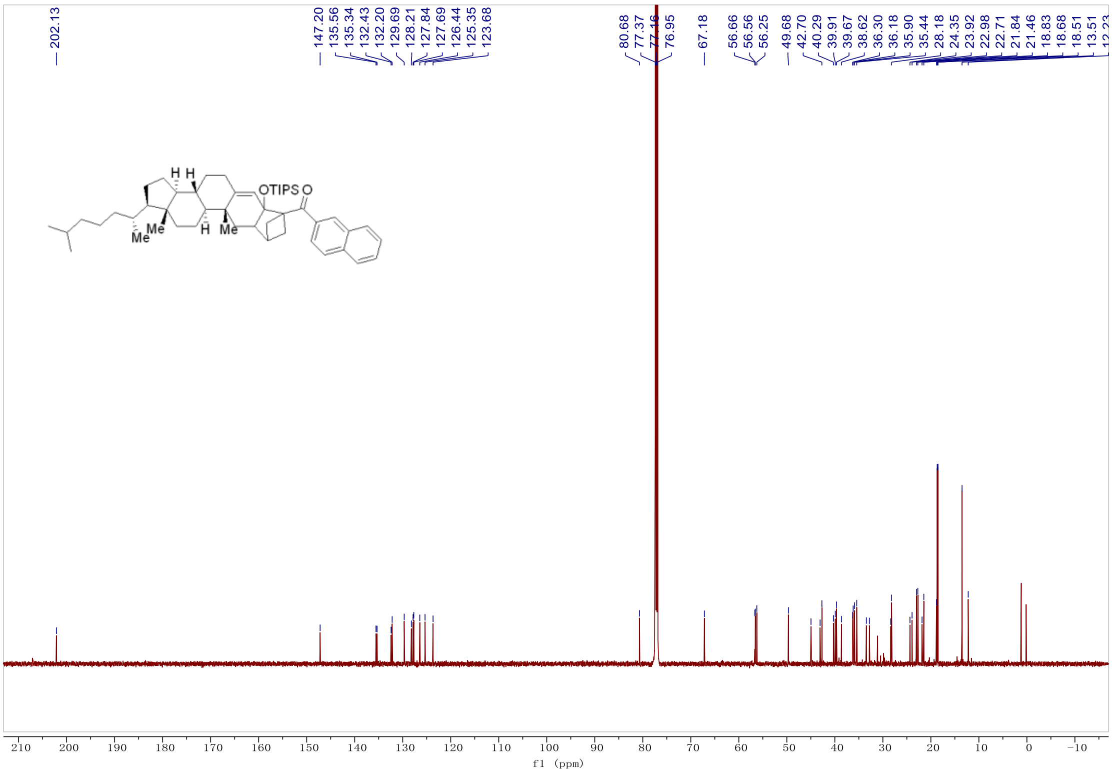

Supplementary Figure 221. ^{13}C NMR spectrum (150 MHz, CDCl$_3$) of **14 (minor isomer)**

5. Supplementary References

1. Zhao, J.-F., Tan, B.-H. & Loh, T.-P. In(III)-pybox complex catalyzed enantioselective Mukaiyama aldol reactions between polymeric or hydrated glyoxylates and enolsilanes derived from aryl ketones. *Chem. Sci.* **2**, 349-352, (2011).
2. de Nanteuil, F. & Waser, J. Catalytic [3+2] annulation of aminocyclopropanes for the enantiospecific synthesis of cyclopentylamines. *Angew. Chem. Int. Ed.* **50**, 12075-12079 (2011).
3. Ma, R. *et al.* Photoinduced trifluoromethylation with CF_3Br as a trifluoromethyl source: synthesis of α-CF_3-substituted ketones. *ACS Omega* **7**, 14357-14362 (2022).
4. Tanis, S. P. *et al.* Solvent and in situ catalyst preparation impacts upon Noyori reductions of aryl-chloromethyl ketones: application to syntheses of chiral 2-amino-1-aryl-ethanols. *Tetrahedron: Asymmetry* **17**, 2154-2182 (2006).
5. Romanski, S. *et al.* Iron dienylphosphate tricarbonyl complexes as water-soluble enzyme-triggered CO-releasing molecules (ET-CORMs). *Organometallics* **31**, 5800-5809 (2012).
6. Banoun, C., Bourdreux, F., Magnier, E. & Dagousset, G. Intermolecular C-O bond formation with alkoxyl radicals: photoredox-catalyzed α-alkoxylation of carbonyl compounds. *Org. Lett* **23**, 8926-8930 (2021).
7. Xu, L. & Shi, H. Ruthenium-catalyzed activation of nonpolar C−C bonds via π-coordination-enabled aromatization. *Angew. Chem. Int. Ed.* **62**, e202307285 (2023).
8. Khan, I., Reed-Berendt, B. G., Melen, R. L. & Morrill, L. C. FLP-catalyzed transfer hydrogenation of silyl enol ethers. *Angew. Chem. Int. Ed.* **57**, 12356-12359 (2018).
9. Mikami, K., Kawakami, Y., Akiyama, K. & Aikawa, K. Enantioselective catalysis of ketoester-ene reaction of silyl enol ether to construct quaternary carbons by chiral dicationic palladium(II) complexes. *J. Am. Chem. Soc.* **129**, 12950-12951 (2007).
10. Yu, J.-Q., Wu, H.-C. & Corey, E. J. $Pd(OH)_2$/C-mediated selective oxidation of silyl enol ethers by *tert*-butylhydroperoxide, a useful method for the conversion of ketones to α,β-enones or β-silyloxy-α,β-enones. *Org. Lett.* **7**, 1415-1417 (2005).
11. Ma, D., Martin, B. S., Gallagher, K. S., Saito, T. & Dai, M. One-carbon insertion and polarity inversion enabled a pyrrole strategy to the total syntheses of pyridine-containing lycopodium alkaloids: complanadine A and lycodine. *J. Am. Chem. Soc.* **143**, 16383-16387 (2021).
12. Schwartz, B. D., Zhang, M. Y., Attard, R. H., Gardiner, M. G. & Malins, L. R. Structurally diverse acyl bicyclobutanes: valuable strained electrophiles. *Chem. Eur. J.* **26**, 2808-2812 (2020).
13. Guo, R. *et al.* Strain-release [2π + 2σ] cycloadditions for the synthesis of bicyclo[2.1.1]hexanes initiated by energy transfer. *J. Am. Chem. Soc.* **144**, 7988-7994 (2022).
14. Ni, D. *et al.* Intermolecular formal cycloaddition of indoles with bicyclo[1.1.0]butanes by Lewis acid Catalysis. *Angew. Chem. Int. Ed.* **62**, e202308606 (2023).
15. Liang, Y., Paulus, F., Daniliuc, C. G. & Glorius, F. Catalytic formal [2π+2σ] cycloaddition of aldehydes with bicyclobutanes: expedient access to polysubstituted

2-oxabicyclo[2.1.1]hexanes. *Angew. Chem. Int. Ed.* **62**, e202305043, (2023).
16. Liu, H.-J., Wang, D.-X., Kim, J. B., Browne, E. N. C. & Wang, Y. Activated cyclooctenones are effective dienophiles. *Can. J. Chem*. **75**, 899-912 (1997).
17. Li, Y.-H. *et al.* Highly stereoselective Diels–Alder reactions catalyzed by diboronate complexes**. *Angew. Chem. Int. Ed.* **62**, e202303075, (2023).
18. Guzmán, P. E., Lian, Y. & Davies, H. M. L. Reversal of the regiochemistry in the Rhodium-catalyzed [4+3] cycloaddition between vinyldiazoacetates and dienes. *Angew. Chem. Int. Ed.* **53**, 13083-13087 (2014).
19. Pronin, S. V., Reiher, C. A. & Shenvi, R. A. Stereoinversion of tertiary alcohols to tertiary-alkyl isonitriles and amines. *Nature* **501**, 195-199 (2013).
20. Singleton, D. A. & Thomas, A. A. High-precision simultaneous determination of multiple small kinetic isotope effects at natural abundance. *J. Am. Chem. Soc.* **117**, 9357-9358 (1995).
21. Kwon, K.-H., Lee, D. W. & Yi, C. S. Chelate-assisted oxidative coupling reaction of arylamides and unactivated alkenes: mechanistic evidence for vinyl C−H Bond activation promoted by an electrophilic ruthenium hydride catalyst. *Organometallics* **29**, 5748-5750 (2010).
22. Bartelson, K. J., Singh, R. P., Foxman, B. M. & Deng, L. Catalytic asymmetric [4 + 2] additions with aliphatic nitroalkenes. *Chem. Sci.* **2**, 1940-1944 (2011).
